# Comprehensive assessment of sequence variation within the copy number variable defensin cluster on 8p23 by target enriched in-depth 454 sequencing

**DOI:** 10.1186/1471-2164-12-243

**Published:** 2011-05-18

**Authors:** Stefan Taudien, Karol Szafranski, Marius Felder, Marco Groth, Klaus Huse, Francesca Raffaelli, Andreas Petzold, Xinmin Zhang, Philip Rosenstiel, Jochen Hampe, Stefan Schreiber, Matthias Platzer

**Affiliations:** 1 Leibniz Institute for Age Research - Fritz Lipmann Institute, Jena, Germany; 2 Roche NimbleGen, Inc., Madison WI, USA; 3 Institute of Clinical Molecular Biology, Christian-Albrechts-University Kiel, Germany; 4 Dept. of General Intermal Medicine, Christian-Albrechts-University Kiel, Germany

## Abstract

**Background:**

In highly copy number variable (CNV) regions such as the human defensin gene locus, comprehensive assessment of sequence variations is challenging. PCR approaches are practically restricted to tiny fractions, and next-generation sequencing (NGS) approaches of whole individual genomes e.g. by the 1000 Genomes Project is confined by an affordable sequence depth. Combining target enrichment with NGS may represent a feasible approach.

**Results:**

As a proof of principle, we enriched a ~850 kb section comprising the CNV defensin gene cluster DEFB, the invariable DEFA part and 11 control regions from two genomes by sequence capture and sequenced it by 454 technology. 6,651 differences to the human reference genome were found. Comparison to HapMap genotypes revealed sensitivities and specificities in the range of 94% to 99% for the identification of variations.

Using error probabilities for rigorous filtering revealed 2,886 unique single nucleotide variations (SNVs) including 358 putative novel ones. DEFB CN determinations by haplotype ratios were in agreement with alternative methods.

**Conclusion:**

Although currently labor extensive and having high costs, target enriched NGS provides a powerful tool for the comprehensive assessment of SNVs in highly polymorphic CNV regions of individual genomes. Furthermore, it reveals considerable amounts of putative novel variations and simultaneously allows CN estimation.

## Background

The defensin gene locus at chromosome 8p23.1 is one of the most copy number (CN) variable regions of the human genome [1]. It harbors genes for different alpha- and ß-defensins, antimicrobial and cytotoxic peptides involved in the orchestrated defense of infection and cancer [2,3]. Both gene dosage (CN) and sequence variation (SNP) affect expression and may influence physiological and pathogenic processes. Hence, determining both types of variants is a prerequisite to further correlate genetic architecture and phenotype [4].

For such targets, assessment of SNPs and multisite variations (MSVs) - comprehensively called single nucleotide variations (SNVs) - simultaneously with reliable CN estimation is extraordinarily challenging. PCR based SNV assessment combined with CN estimation both by traditional Sanger and next-generation sequencing (NGS) is feasible [5-7] but covers only tiny parts of the region consisting of two clusters (DEFA and DEFB) spanning ~200 and ~235 kb, respectively.

Recently, NGS methods have allowed re-sequencing of complete individual human genomes [8-13]. On the other hand, costs are still too high for sequencing entire genomes when only CNV regions of several hundred kb are of interest.

A way to overcome this constriction may be sequence capture methods allowing the enrichment of selected target regions over 1000-fold above their normal fraction in the genome [14,15]. These techniques are either based on arrays [16-20] or in-solution enrichments [21,22].

As a proof of principle and to comprehensively assess the sequence variability in the 8p23 DEF region of human genomes with simultaneous estimation of DEFB cluster CNs, we enriched the DEF regions and 11 different *bona fide *single copy control regions (CTRL) from two cell-line derived individual DNAs by NimbleGen Sequence Capture Arrays and sequenced by 454/Roche. The 454/Roche technology was chosen as it provides the longest sequence reads among available NGS approaches which in turn allowed inference of CNs from haplotype ratios [6] simultaneously with the variability assessment.

## Methods

To investigate a "simple" as well as a "complex" case in terms of DEFB sequence variability we selected the samples NA12716 and NA12760, harboring 2 and 6 DEFB copies per diploid genome, respectively, as multiply determined by different methods [7,23-25]. Both genomes were genotyped by the International HapMap project [26], the corresponding cell lines were derived from male individuals of European origin and are publicly available. Next generation sequencing of these DNA samples was done in the framework of the 1000 Genomes Project (http://www.1000genomes.org/) in the low coverage group by the Solexa/Illumina and ABI/SOLiD platforms resulting in 24 Gb (NA12716) and 40 Gb (NA12760) raw data, respectively.

### Cell lines and DNAs

B-lymphocyte, EBV transformed cell lines GM12716 (CEPH Utah pedigree 1358, male, European origin) and GM12760 (CEPH Utah pedigree 1447, male, European origin) were purchased from the Coriell cell depository (http://ccr.coriell.org/). Both cell lines are part of the HapMap project HAPMAPPT01 (http://hapmap.ncbi.nlm.nih.gov/). Cell lines were cultured in RPMI1640 with GIBCO GlutaMAX™ (Invitrogen) with 10% Fetal Bovine Serum "GOLD" (PAA Laboratories, Austria) and 1.5% PenStrep (Roth, Karlsruhe, Germany) in 25 mL and 75 mL BD Falcon™ flasks at 37°C and 5% CO_2 _in a total amount of 10 mL and 30 mL, respectively. Cells were grown to a density of 1 × 10^6 ^cells/mL and split in a ratio of 1:3. From the cell lines, DNAs NA12716 and NA12760 were isolated using the QIAamp DNA-Blood Mini Kit (Qiagen, Germany).

### Sequence Capture Enrichments

Total amounts of 32 ug of DNA NA12716 (c = 265 ng/uL in TE) and 30 ug of DNA NA12760 (c = 150 ng/uL in TE), both with A^260^/A^280 ^≥1.8 and A^260^/A^230^≥1.9 (measured by Nanodrop) were enriched using a customer designed 385K 1-plex sequence capture array following the manufacturer's instructions (Roche NimbleGen, Madison, WI). Target regions, captured regions and chromosomal positions are listed in additional file 1.

### Target Enrichment

DNA was isolated from the cell lines and enriched on a customized 385K Sequence Capture Array, designed and produced by NimbleGen (Madison, WI, USA). On the array, 694 kb (82%) of the 850 kb target comprised by DEFA (195 kb), DEFB (234 kb) and 11 CTRL regions (421 kb) were tiled by probes (additional file 1). Within two of these CTRL genes (*ANGPT2*, *MFHAS1*), probe hybridization sites were previously defined for Multiplex Ligation Dependent Probe Amplification (MLPA) and used for calibration of DEFB CN estimation [27].

### 454 Sequencing

Shotgun sequencing libraries were prepared by the GS Titanium library preparation kit following the manufacturer's instructions (Roche Diagnostics). The single-stranded libraries were quantified by a qPCR assay [28] and processed utilizing the GS Titanium emPCR and XLR70t sequencing kit (Roche Diagnostics) according to the manufacturer's instructions. Sequencing was performed by three runs on a half 70 × 75 picotiter plate each (NA12716) and one run on a complete 70 × 75 picotiter plate (NA12760), respectively. Sequencing of the enriched DNA by GS FLX (Titanium, Roche) yielded ~450 Mb (NA12716) and ~300 Mb (NA12760) of raw sequence data. The raw sequence data outputs (reads, bp, average read lengths) are listed in additional file 2. All 454 sequences mapping to the CTRL, DEFA and DEFB target regions were deposited at the NCBI Short Read Archive (SRA, http://www.ncbi.nlm.nih.gov/Traces/home/) under the accession number SRA024359.

### Sequence Analysis

The 454 reads in the sff files from NA12716 and NA12760 were independently mapped by the GS Reference Mapper against the concatenated target regions as backbone with a minimum initial match of 92% and a minimal initial mapping length of 80 bp (target filtering). The fully and uniquely mapping sequences were then mapped again to the human genome (NCBI build 36, hg18) masked for all except the target regions (chromosomal filtering). To distinguish between known and putative novel SNVs the information about known SNP positions for the reference sequence (GoldenPath snp128.txt) were used. The SNVs classified as putative novel by these information were re-validated by alignment against the human reference SNP sequence data (ftp://ftp.ncbi.nih.gov/snp/organisms/human_9606/rs_fasta/) using Blat [29]. The Blat output was analyzed by an inhouse perl script.

### Error model

Sequencing errors that accumulate at particular positions may falsely indicate novel alleles. HapMap SNPs homozygous for the variant allele of the CTRL and DEFA regions (n = 248) were used to determine the average per-site error frequency in the aligned reads as 2.3%. A binomial distribution was then used to model the false positive rate in SNP/SNV detection, given the average error frequency (p) and local sequencing depth (n). The model was used to evaluate all putative allele calls reported by HCDiff by calculating the posterior probability of observing d identical deviations just as a result of error: . A threshold of P = 10^-3 ^was applied to remove likely false positives from the output of HCDiff.

### SNV verification by PCR/cloning/Sanger sequencing

SNV verification was performed using the cell line derived DNA without sequence capture enrichment. PCR primers are listed in additional file 3. PCR products were either directly sequenced using dye terminator chemistry (ABI3730xl) or after cloning into pCR2.1 TOPO vector (Invitrogen).

### Haplotyping and CN estimations

For haplotyping, all sequences covering the haplotyping candidate regions (HTCR) were extracted and re-assembled in GAP4 (Staden package). The GAP4 assemblies were manually inspected at the polymorphic positions and haplotypes were inferred by a proprietary script. CN estimations were done as previously described [5-7] by counting all sequences representing the same haplotype and calculating the ratio of read numbers.

## Results

### Identification of high confidence differences (HCDiff)

**Figure 1:**
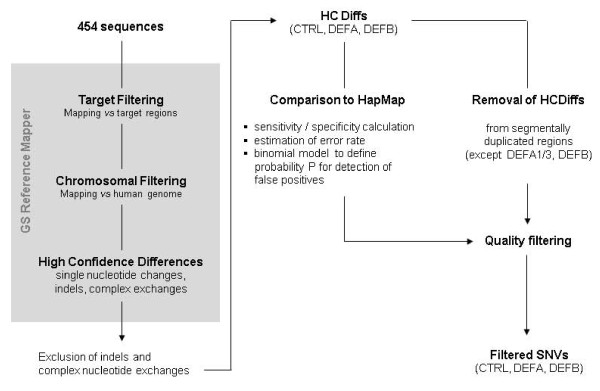
**Workflow for the identification of SNVs from 454 sequences generated after target enrichment by NimbleGen sequence capture of individual DNAs**.

The raw data obtained from 454 sequencing of the target enriched DNA (see Methods) were subjected to an analysis and filter process depicted in Figure 1. A first filtering was done by mapping the raw data against the target regions. The fractions of fully and uniquely mapping reads were 47% and 51% for NA12716 and NA12760 and 91% and 93% of the target regions were covered by at least 2 sequences, respectively. In relation to 850 kb target and 3 Gb human genome, the enrichment rates were ~470x and ~630x. The corresponding sequencing depths for CTRL, DEFA, and DEFB were 60x, 56x, 45x for NA12716 and 44x, 37x, 100x for NA12760, respectively. The target filtered sequences were then mapped by the Reference Mapper (Roche) against the human genome (NCBI build 36.1, UCSC hg18) in order to assign chromosomal positions and to provide high confidence differences (HCDiffs) to the reference. These differences included single nucleotide variations (SNVs), insertions/deletions (indels) and complex nucleotide exchanges. We further focused on SNVs by discarding indels and complex exchanges from the HCDiffs list. This resulted in 3,161 and 3,490 HCDiffs for the CTRL, DEFA and DEFB regions of NA12716 and NA12760, respectively.

### Evaluation of sensitivity, specificity and false discovery rate by comparison to HapMap SNPs

To estimate the accuracy of the SNV identification and to define reliable quality filter settings for the DEFB analyses, we compared the HCDiff data of the single-copy CTRL and DEFA regions with SNP data from the International HapMap project (http://hapmap.ncbi.nlm.nih.gov). We categorized the 1,388 and 1,251 HCDiffs for NA12716 and NA12760, respectively, by their variant's allele frequencies (VAF, frequency of the allele deviating from the hg18 reference). According to the Reference Mapper software, VAFs for HCDiffs by definition range between 10% and 100%. We therefore defined the categories as homozygous for the reference allele (10% ≤VAF <25%), heterozygous (25% ≤VAF ≤75%) and homozygous for the variant (75% <VAF ≤100%) (additional file 4). The corresponding HapMap data (Phase III, release 2, February 2009) for CTRL and DEFA of NA12716 and NA12760, comprised 1,337 and 1,322 genotypes, respectively. Comparing the HapMap data with our HCDiffs, we estimate that our approach has equal sensitivity and specificity for the identification of hetHCDiffs (94%-97%) and homvarHCDiffs (98%-99%) for *bona fide *single-copy loci with 2 copies per diploid genome (Table 1).

**Table 1:** Comparison of high confidence differences (HCDiffs) obtained from the CTRL and DEFA regions by sequence capture (SeqCap) with HapMap SNP genotypes.

	NA12716	NA12760		NA12716	NA12760
HapMap: heterozygous	244	263	SeqCap: heterozygous	711	646

SeqCap:			HapMap:		
homozygous for reference, no HCDiff	0	5	heterozygous	237	248
homozygous for reference	0	2	homozygous for variant	5	4
heterozygous	237	248	homozygous for reference	10	4
homozygous for variant	3	0			
not or poorly covered/alignment problem	4	8			

HapMap: homozygous for variant	254	217	SeqCap: homozygous for variant	526	468

SeqCap:			HapMap:		
homozygous for reference, no HCDiff	0	0	homozygous for variant	248	212
homozygous for reference	0	0	homozygous for reference	0	0
heterozygous	5	4	heterozygous	3	0
homozygous for variant	248	212			
not or poorly covered/alignment problem	1	1			

HapMap: homozygous for reference	839	842	SeqCap: homozygous for reference	151	137

SeqCap:			HapMap:		
homozygous for reference, no HCDiff	827	835	homozygous for variant	0	0
homozygous for reference	1	1	homozygous for reference	1	1
heterozygous	10	4	heterozygous	0	2
homozygous for variant	0	0			
not or poorly covered/alignment problem	1	2			

Sensitivity (heterozygous)	97,1%	94,3%	Specificity (heterozygous)	94,0%	96,9%
Sensitivity (homozygous)	98,4%	99,0%	Specificity (homozygous)	98,4%	98,6%

The SeqCap HCDiffs were categorized according to the variant's allele frequency (VAF) with the human genome sequence (hg18) as reference allele: homozygous for reference (VAF 10-24%); heterozygous (VAF 25-75%); homozygous for variant (VAF 76-100%).

In addition, we characterized the false discovery rate in more detail. It is caused by sequencing errors that accumulate at particular positions and falsely indicate novel alleles. Using 248 HapMap SNPs of the CTRL and DEFA regions of NA12716 which are homozygous for the variant allele we determined the average per-site error frequency in the alignment as 2.3%. This rate reflects the combined errors from sequencing and read mapping. A binomial distribution was then used to model the false positive rate in HCDiff detection, given the average error frequency (p = 0.023) and local sequencing depth (n) (see Materials and Methods). VAF distributions of sequenced HapMap sites and VAF distributions predicted by this binomial model showed good agreement (additional files 5, 6). Consequently, this model was used to assign the posterior error probability P to each putative allele call reported by HCDiff, and this was used for quality estimation of HCDiffs (see below).

### SNV assessment by filtering of HCDiffs

To obtain a reliable set of SNVs, the identified HCDiffs were successively subjected to two filter steps. First, by comparison to the hg18 reference, we re-checked our target regions for parts with more paralogs than annotated in the known copy number variable DEFA and DEFB regions at 8p23.1. We identified a 69 kb region in the DEFB cluster which overlaps an 80 kb low copy repeat (LCR type IV) [6]. LCR IV has additional paralogs at chromosomes 8 and 12. They are subjected to the enrichment together with the target regions due to sequence identities of up to 98% and hampered the SNV identification in the DEFB region. Consequently, we excluded all variations identified in this region, reducing the data set to 1,965 and 2,369 HCDiffs for NA12716 and NA12760, respectively, corresponding to 62% and 68% of the primary amount (additional file 7).

Then, as a sequence quality filtering, we applied our binomial distribution model (see above) to the reduced set of HCDiffs. Variants with probability values P >1 × 10^-3^, were considered error-prone and discarded, finally resulting in 1,919 and 2,265 HCDiffs (61% and 65%) for NA12716 and NA12760, respectively (additional file 8).

**Figure 2:**
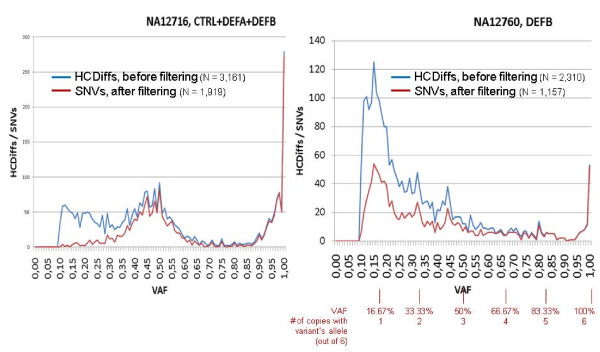
**Distribution of variant's allele frequencies (VAFs) before (HCDiffs) and after filtering (SNVs)**.

When plotting the HCDiffs according to their VAFs, the VAF distribution profile changes as a result of the filter process. For NA12716, due to the copy number of 2 for all target regions, HCDiffs should be spread around 50% VAF (heterozygous) and near 100% (homozygous for the variant). However, with the unfiltered HCDiffs, we observed a high amount of HCDiffs far below the expected peak at 50% VAF. In contrast, the plot of the filtered variations has exactly the anticipated pattern. Analogously, the curve for NA12760 CTRL+DEFA (2 copies) is changed after filtering, resulting in a shape similar to NA12716 (additional file 9). For NA12760 DEFB (6 copies) the diagram reflects the different VAFs from allele ratios of 1:5 to 6:0 (Figure 2).

We defined the set of 1,919 and 2,265 filtered HCDiffs as the final set of SNVs which was sub-classified by region and presence or absence from the SNP database (known/putative novel SNV). To distinguish between both categories we initially used the UCSC hg18 annotations and re-validated the variations classified as unknown by comparison to the more up-to-date dbSNP130 database (release 2009, April 30^th^). This revealed 574 known/41 putative novel SNVs in the CTRL regions, 604/27 in DEFA and 560/113 in DEFB of NA12716. For NA12760, 595/48 SNVs in CTRL, 428/37 in DEFA and 979/178 in DEFB were identified.

Similarly to the HapMap comparison (see above) we further classified the SNVs by VAF. For CTRL and DEFA in both samples as well as for DEFB in NA12716, SNVs with 25% ≤VAF ≤75% were regarded as heterozygous (het) and as homozygous for the variant (homvar) with 75% < VAF ≤100%. This revealed 1,165 het and 695 homvar SNVs (61% and 36% of all, respectively) for NA12716 and 610 het (55%) and 468 homvar (42%) for NA12760. For both samples, a fraction of ambiguous SNVs with 10% ≤VAF <25% remained, encompassing only ~3% of all SNVs, which can be regarded as measure of the false positive rate in the lower quarter of VAF. The SNVs from NA12760 DEFB (6 copies per diploid genome) were sub-classified by the number of copies deviating from the hg18 reference using the VAF ranges 10-24%/25-42%/43-58%/59-75%/76-92%/93-100%. This revealed 507, 257, 156, 88, 59 and 89 SNVs for the six classes from 1 out of 6 copies to 6 out of 6 copies (homvar).

**Figure 3:**
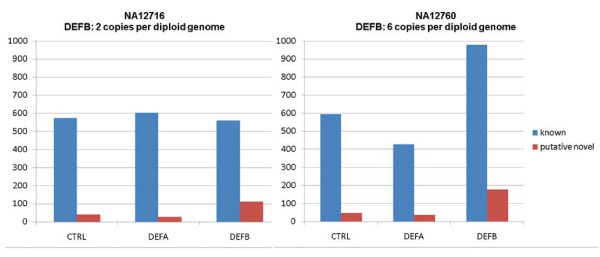
**Known and putative novel SNVs identified in the different target regions of NA12716 and NA12760**.

We also checked the final SNV sets for overlap between both DNAs, resulting in 771 known/77 putative novel unique SNVs in the CTRL regions, 701/61 in DEFA and 1,056/220 in DEFB (Table 2). A summary for the final SNV sets is shown in Figure 3, all HCDiffs and SNVs are listed in detail in additional files 10, 11 and 12.

**Table 2:** Known and putative novel (putnov) SNVs and SNV densities in CTRL, DEFA and DEFB of NA12716 and NA12760 after successive filtering of HCDiffs

	Total		CTRL			DEFA			DEFB		
	SNVs	SNVs per kb	known	putnov	SNVs per kb	known	putnov	SNVs per kb	known	putnov	SNVs per kb
NA12716	1.919	2,3	574	41	1,5	604	27	3,2	560	113	4,1
NA12760	2.265	2,7	595	48	1,5	428	37	2,4	979	178	7,0

unique SNVs	2.886		771	77		701	61		1.056	220	
			848			762			1.276		

### Experimental validation of SNV candidates

To test the reliability of SNV identification by our pipeline we experimentally verified 22 HCDiffs from the CTRL, DEFA, and DEFB regions of NA12760. All investigated HCDiffs are putative novel variations. The set comprised HCDiffs with sequencing depths ranging from 17 to 120 reads and VAFs between 13% and 97%.

The SNV flanking regions were amplified from the total non-enriched DNA NA12760 and the PCR products were sequenced by the Sanger method. Furthermore, 3 DEFA and 6 DEFB PCR products were also subcloned and the clones were sequenced. For 19 HCDiffs with VAFs between 17% and 76%, the presence of two alleles could be confirmed. For 2 HCDiffs with 96% and 97% VAF, respectively, the variant allele was found exclusively. The remaining 1 HCDiff (chr8:7,261,330) with a sequence depth of 104 and 13%VAF was found homozygous for the hg18 reference allele in the Sanger-sequenced PCR product as well as in 98 clones derived from it. Hence, 21 HCDiffs out of 22 (95%) were confirmed. For the HCDiffs which were additionally verified by cloning, the VAF values of cloning and 454 sequencing are in good agreement (additional file 13).

Comparison of the HapMap with our NA12716 data revealed 18 discrepancies which can be divided in three groups: (A) heterozygous/homozygous for the variant (HapMap/sequence capture; 3 cases); (B) homozygous for the variant/heterozygous (5 cases) and (C) homozygous for the reference/heterozygous (10 cases). We verified 14 of these variations by PCR and Sanger sequencing, confirming for 9 and 5 the sequence capture results or HapMap status, respectively. In addition to the 18 discrepancies, 6 HapMap SNVs were not resolved by the sequence capture experiment due to low sequence depth, low quality or alignment errors (Table 1).

### Haplotyping and CN estimations

With an average read length of >300 bp, in regions of high SNV densities at least a certain amount of GS FLX sequences should span two or more SNVs positions, allowing the inference of haplotypes. Furthermore, as previously shown [5-7], copy numbers can be estimated from the ratio of sequences representing the distinct haplotypes.

For this purpose we have selected from our alignments 13 haplotyping candidate regions (HTCRs) within DEFB of NA12716 and NA12760. Altogether, the HTCRs harbor 165 SNVs and span 10 kb (~16 SNVs/kb) with at least 9 SNVs/kb (additional files 14, 15). For the inference of haplotypes from HTCRs we postulated a minimal number of 5 sequences per haplotype. Consequently, haplotyping was done if the SNV combination was covered by ≥10 reads for NA12716 (2 copies per diploid genome) and ≥30 reads for NA12760 (6 copies). In total, 124 SNVs could be used to identify 188 haplotypes from combinations of 2 to 7 SNV (NA12716 22; NA12760 166 haplotypes).

At the loci with 2 copies per diploid genome, the ratio of reads representing the same haplotype should be 1:1. Indeed, we found for NA12716 DEFB 11 SNV combinations consisting of two haplotypes in a ratio of 172:173 reads (0.99:1). For NA12760 CTRL, 14 × 2 haplotypes were inferred with a total read ratio of 104:97 (1.07:1; additional file 16).

In the DEFB region of NA12760 (6 copies), 48 CN estimations could be done from 166 different haplotypes (on average 3.5 haplotypes per SNV combination), revealing between 4 and 8 copies per diploid genome with an average of 6.17 ± 1.06 (Table 3)

**Table 3:** DEFB cluster copy numbers (CN) per diploid genome of NA12760 estimated by the ratio of reads per haplotype

region/gene	reads	read numbers with different haplotypes	read ratio	CN
*DEFB4*	51	17:34	1:2 (3n)	6
	89	16:62:11	1:4:1	6

down	45	10:26:9	1:3:1	5
stream	35	11:14:4:6	2:3:1:1	7
*DEFB103*	102	25:12:19:13:12:21	2:1:1:1:1:2	8
	33	8:6:11:8	1:1:2:1	5
	64	9:8:47	1:1:4	6

*SPAG11*	38	15:23	2:3	5
	60	29:16:15	2:1:1	4
	100	50:16:34	3:1:2	6
	42	7:12:15:8	1:2:2:1	6
	63	22:11:12:18	2:1:1:2	6
	54	20:27:7	3:4:1	8
	37	20:10:7	3:2:1	6
	35	19:16	1:1 (2n)	6
	48	14:21:13	2:3:2	7
	30	4:21:5	1:5:1	7
	32	8:14:10	2:3:2	7
	48	7:22:19	1:3:3	7
	48	9:20:6:13	1:2:1:1	5
	62	13:18:13:7:11	2:2:2:1:1	8
	34	6:13:15	1:2:3	6
	33	12:21	1:2 (3n)	6
	40	6:8:26	1:1:4	6
	33	3:4:21:5	1:1:4:1	7
	53	12:33:8	1:3:1	5

*DEFB104*	36	17:7:12	3:1:2	6
	37	14:16:7	2:2:1	5
	53	10:17:17:9	1:2:2:1	6
	32	20:12	2:1 (3n)	6
	30	13:10:7	3:2:1	6
	30	14:6:6:4	3:1:1:1	6
	86	63:26	3:1	4
	62	6:8:21:27	1:1:3:3	8
	65	13:23:15:14	1:2:1:1	5
	31	6:6:6:5:8	1:1:1:1:2	6
	38	10:10:5:3:10	2:2:1:1:2	8
	29	6:7:16	1:1:3	5
	31	9:14:8	2:3:2	7
	33	6:27	1:5	6

*DEFB106*	100	32:12:18:38	2:1:1:2	6
	77	21:13:31:12	2:1:3:1	7
	30	8:22	1:3 (4n)	8
	63	20:8:22:13	2:1:2:1	6
	61	17:6:24:14	2:1:3:2	8
	39	7:5:27	1:1:4	6

*DEFB107*	28	5:5:5:13	1:1:1:3	6
	97	78:19	4:1	5

			total number of reads	2.397
			total number of CN estimations	48
			CN per diploid genome - average	6,17
			- max	8
			- min	4
			- STDEV	1,06

### SNVs in the putative DEFB4 promoter region

**Figure 4:**
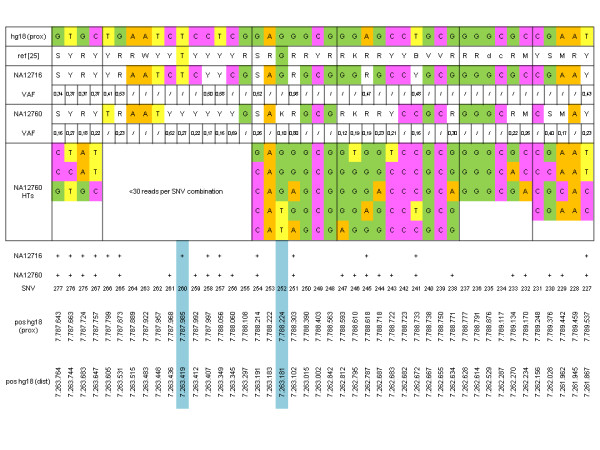
**SNVs in the putative DEFB4 promoter region ~1.9 kb upstream of the transcription start site inferred from the NA12716 and NA12760 sequence alignments with variants' allele frequencies (VAF) in comparison to the reference NCBI build 36.1 (hg18) and to variations identified by Groth et al**. [25]. Dash: homozygote for the hg18 allele NA12760 HTs: haplotypes from SNV combinations represented by >30 sequences (+): confirmation of polymorphisms identified in [25]; for SNV details see additional file18. SNVs 252 and 260 (blue) were found to be polymorphic in NA12716 and/or NA12760 by the present study but not in [25].

Recently we have identified haplotypes in the putative *DEFB4 *promoter region (hg18 chr8:7,261,867-7,263,764 and chr8:7,787,643-7,789,537) and have found an extraordinarily high density of ~26 SNVs per kb [25]. We therefore inspected the sequence alignments of NA12716 and NA12760 in this region (SNVs 227 to 277; additional file 17). In total, 13 out of 42 SNVs identified by Groth et al. were also found to be heterozygous or homozygous for the variant in NA12716. Analogously, we identified 24 such positions in NA12760 of which 12 are identical in both DNAs. Thus, for 25 out of 42 SNV positions (60%) previously identified in 9 individuals, the variant alleles were also found in at least one of the two genomes of NA12716 and NA12760 (Figure 4).

Two additional SNVs were found to be polymorphic in NA12760. One (SNV252, putative novel) showed 10%VAF, the second (SNV260, rs2737910) 27%VAF. In the same way as described above we also inferred haplotypes from the NA12760 putative *DEFB4 *promoter region (additional file 18).

## Discussion

CN polymorphisms are an experimental challenge with respect to their identification as well as for their genotyping in association or linkage studies [30]. Disease association analyses in CN variable loci are therefore currently restricted to the determination of CNs thereby often ignoring the variable sequence contents of the respective copies. The defensin locus on 8p23 is an extreme example of a large CN variable genomic locus (235 kb, 2 to at least 12 copies in individual genomes). Knowledge of the copies' variability is still fragmentary and we aimed with our study to describe it in more detail. The power of a combination of selective enrichment of target regions in the human genome and NGS has already been demonstrated [17,31-33], but such an approach has not been applied to CN variable loci so far.

By our sequence capture experiments using two individual genomic DNAs, the CTRL, DEFA and DEFB target regions with a total length of 850 Mb were enriched 600fold above their normal fraction in the whole human genome. Since this calculation is related to the entire target instead of the captured regions (82%), the enrichment rate is rather underestimated and in every case higher than determined by qPCR in the course of the enrichment process (~150fold, data not shown). Subsequent 454 sequencing of the enriched DNAs and mapping the sequences to the human genome resulted in CTRL, DEFA and DEFB sequence depths of 60, 56 and 45 (1.1:1.0:0.8) for NA12716 and 44, 37 and 100 (1.1:0.9:2.5) for NA12760, respectively. Although the sequencing depth ratios CTRL:DEFA:DEFB are not exactly 1:1:1 for NA12716 and 1:1:3 for NA12760 as expected from the DEFB CNs, the higher coverage of DEFB in NA12760 compared to NA12716 reflects the difference in DEFB CNs between the two DNAs and saturation of the array in case of high target region CNs. Furthermore, the CTRL:DEFA sequence depth ratio of ~1:1 for both DNAs indicates the single copy status (2 copies per diploid genome) of these regions.

### Quality estimation and filtering

Assessment of known and putative novel single nucleotide variants (SNVs) was performed on the base of the Roche Reference Mapper software providing high confidence differences (HCDiff) including chromosomal positions, sequence depths, variant's allele frequencies and SNP entry IDs. Comparison of the HCDiffs to HapMap data allowed the evaluation of the SNV identification accuracy, revealing a false-positive call rate of 2.3% per site. With sensitivities and specificities of ≥94% for the detection of heterozygote SNVs and >98% for homozygote SNVs we achieve similar values to those obtained by NimbleGen whole exome capture arrays combined with Illumina sequencing [34].

The second filter step for the obtained HCDiffs used a binomial distribution based model to distinguish with a given probability between real SNVs and nucleotide differences by PCR or sequencing errors. From the entire set of HCDiffs for NA12716 and NA12760, 4.6% and 5.5% have a probability value P >10^-3^, respectively. There are, however, remarkable differences between the excluded and remaining HCDiffs of the first filter step. Of the discarded variations from the segmentally duplicated region, 8.2% and 7.9% of the HCDiffs have P-values > 10^-3 ^in contrast to 2.3% and 4.4% for the retained ones. This suggests again that our filtering for HCDiffs in the segmentally duplicated DEFB region is reasonable.

With respect to the 4,184 SNVs identified in both DNAs and the applied quality threshold of P ≤ 10^-3 ^we expect 4-5 false positives which are sequencing artifacts. Under more stringent conditions (P ≤ 10^-4^) we would exclude them which in turn remove another 97 potentially real SNVs, thereof 25 (~7%) of the putative novel variations (data not shown).

### SNV densities and validation of putative novel SNVs

By our approach we identified 1,919 and 2,265 SNVs (het and homvar) in the CTRL, DEFA and DEFB regions for NA12716 and NA12760, respectively, corresponding to a mean density of 2.3 and 2.7 SNV/kb (Table 2). However, the SNV densities differ between the regions. With 1.5 SNV/kb for both DNAs, the invariable single copy CTRL genes have the lowest densities while they are somewhat higher for the DEFA cluster with 3.2 and 2.4 SNV/kb for NA12716 and NA12760, respectively. The highest density of 4.1 SNV/kb was observed for the DEFB cluster of NA12716. In contrast, mapping the identified DEFB SNVs of NA12760 to 3 DEFB copies per haploid genome results in a density of 2.3 SNV/kb. These findings reflect either a higher relatedness of NA12760 DEFA/B to the reference sequence, or lower sensitivity of the SNV detection in regions with high CN, or both. Sensitivity problems are expected in particular for rare alleles in NA12760 DEFB (1:5) with an expected VAF of 16.67% and an experimental VAF distribution between 10 and 25% which are selected against by our stringent filtering (Figure 2).

In both DNA samples we have identified 358 putative novel SNVs (Table 2, Figure 3). Their fraction with respect to all identified SNVs ranges between 4% and 8% in the CTRL and DEFA regions but 15% and 17% in DEFB. We compared our putative novel SNVs both with the most current dbSNP132 database (release 2010, September 23^rd^) and the data from the 1000 Genomes Project (status 2011, January 18^th^). The latter provided sequences for NA12716 and NA12760 corresponding to depths of ~8x and ~13x, respectively. Out of our 358 putative novel SNVs, 75 were meanwhile identified and submitted to dbSNP by the 1000 Genomes Project and another 93 by other groups. In contrast, 190 (53%) of our novel SNVs identified by targeted analysis in just two genomes remained undetected by the significantly larger efforts of others. This underlines the efficiency of our approach and is complemented by the fact that namely in the DEFB cluster only 44 positions were genotyped by HapMap [26].

There are no differences between known and putative novel SNVs with respect to sequence depth and quality. The mean sequence depth of the known SNVs is 59x (CTRL 49x, DEFA 54x, DEFB 71x) and 58x for the putative novel variations (CTRL 37x, DEFA 53x, DEFB 65x). For both known and putative novel SNVs more than 97% of the variants have P-values <10^-4^.

Out of the 2,886 SNVs identified in the two genomes, 48 are coding variations (34 non-synonymous, 14 synonymous). Thereof, in the DEFB cluster, 5 SNVs (4 non-synonymous, 1 synonymous) were found in the coding sequences of *DEFB104, DEFB106*, *DEFB105 *and *DEFB107*. None of these 5 SNVs are novel variations. In contrast, 220 putative novel SNVs were identified in the non-coding parts of the DEFB cluster. This indicates that sequence divergence between DEFB cluster paralogs rather evolves in non-coding than in protein-coding regions and protein dosage is realized by different expression levels of alleles. This is in accordance with Hardwick et al. [23] showing that the different levels of *DEFB103 *expression are not only the result of variable gene copy numbers but also controlled by rapidly evolving SNVs in regulatory regions."

By PCR and Sanger sequencing we confirmed 95% (21/22) of the putative novel SNVs. The unconfirmed SNV which was found to be homozygote for the hg18 allele by Sanger sequencing, belongs to a group of 5 variations with VAFs <25% in 454 sequencing. The VAF for the not confirmed SNV (13%) is in the range for 1 deviating copy out of 6 (16.67%) indicating the difficulty to distinguish between variations of this type and false positives of the applied method. Candidate SNVs with VAF ≥25% were all confirmed.

Although this study is focused on SNVs, polymorphic insertions/deletions (indels) may cause particular phenotypes and have to be assessed in future studies.

### Haplotypes and copy numbers

With average sequence lengths between 300 and 350 bp the obtained GS FLX sequences were suitable for the identification of haplotypes in regions with a certain SNV density. In total, 124 SNVs were informative for the identification of 188 different haplotypes in the DEFB regions of NA12716 and NA12760.

High sequencing depth and the calculation of read ratios is essential for haplotype-based CN estimation. In the present capture experiment, the sequencing depth was at the lower bound for a reliable CN determination. In the case of NA12716, 11 informative SNV combinations with an average of 31 reads per combination were available in DEFB. All of them exhibit 2 haplotypes per locus, strongly supporting the DEFB number of 2 per diploid genome as previously determined by other methods [23,24]. In contrast, read ratios were consistent with an expected 1:1 ratio of haplotypes only in 6 of the haplotyped regions. Nevertheless, artificially improving sequencing depth by summing up read counts for all loci resulted in a very robust haplotype ratio of 0.99:1.00 (172/173).

For NA12760 with a higher DEFB CN of 6, the average number of sequences per SNV combination was ~50. Again, this is below the requirements for reliable *de novo *CN estimation, in particular if the diplotype comprises more than 2 copies. As consequence, DEFB CN estimations for NA12760 by individual haplotype regions ranged between 4 and 8. However, due to the higher number of reads per haplotyped region, 48 CN estimations were possible with an average ~6 copies per diploid genome as previously determined by alternative methods [7,23,24]. Since consistent CN estimations were made from SNVs spread over the entire DEFB region (*DEFB4, DEFB103, SPAG11, DEFB104, DEFB106 *and *DEFB107*) our haplotyping results also confirm the concordance of CNs for all genes within the cluster [35].

### Completeness of SNV identification

In total, 82% of the CTRL, DEFA and DEFB target regions were tiled by sequence capture probes in our assay. The remaining untiled parts are located in repetitive elements and/or regions with segmental duplications. Sequences and therefore HCDiffs from such regions however can be obtained from enriched DNA fragments captured by probes at the edges of non-repetitive/non-duplicated regions. Due to the uniqueness of the fragment in its non-repetitive part, the HCDiffs outside of it should be correctly assigned to their chromosomal position and we regarded them as reliable variations. Indeed, 510 (12%) out of 4,184 SNVs from both DNAs are located within ± 200 bp around tiled regions and 466 (91%) thereof are embedded in repetitive elements.

While captured fragments extending the tiled regions increase the fraction of sequence covered target parts, it is decreased by ineffective probes. The probe efficiencies are hard to estimate but are reflected by regions not or poorly covered by 454 sequences despite being tiled. For example, the mean sequence depth of the *DEFA1A3 *genes, spanning ~41 kb and located in the 178-kb-DEFA cluster is underrepresented in both capture experiments, with only 7x compared to 37x for the entire cluster in NA12760.

Due to the described opposite effects and because the true number of nucleotide differences between the target regions of the investigated DNAs and the hg18 reference is unknown, we cannot exactly quantify the completeness of our SNV identification with respect to the whole target regions. We can, however, give an estimation based on the sequence depths. In the sequence alignments of NA12716 and NA12760, 87% and 91% of the target positions are covered by more than 5 sequences, respectively (data not shown). HCDiff identification by the Reference Mapper software is based on a combination of flow signal and quality score and requires a minimum number of sequences, the presence of both forward and reverse reads covering the difference etc. The minimal requirement for classification of a position as HCDiff is the coverage by ≥3 sequences in both orientations with Phred quality ≥Q20 [36,37] representing the variant, unless there are ≥5 reads in just one orientation.

Therefore, as a conservative calculation, we estimate the completeness of our SNV identification in NA12716 to less than ~87% with respect to the entire target regions. Regarding the identified 1,919 SNVs this would mean that we have missed at least ~290 SNVs, probably located predominantly in repetitive regions.

With respect to the sensitivity of variation detection in the CN variable DEFB, we can assume the same 98% sensitivity for the tiled regions of the 2-copy sample NA12716 as it is with one copy per haploid genome genetically identical to the HapMap loci, from which the sensitivity measure was derived. This high sensitivity/specificity was achieved by eliminating all variations with a VAF <25%. Of the final 673 NA12716 DEFB SNVs only 7 (1%) show a VAF <25%. In contrast, 43% (492/1157) of the NA12760 DEFB SNVs exhibit a VAF <25%. These variations most likely are present in only one of the 6 DEFB cluster copies. Moreover, these variations are heavily selected against by our stringent quality filters (Figure 2). Thus, it has to be expected that the sensitivity of our approach significantly drops in regions with higher copy numbers. To estimate the sensitivity for the 6-copy NA12760 DEFB, we assume 687 SNVs (673 observed NA12716 SNVs divided by 0.98 sensitivity) per haploid DEFB. Thus we would expect in the case of NA12760 a 3-times higher overall number of 2,061 DEFB SNVs. Having observed 1,157 NA12760 DEFB SNVs demonstrate a sensitivity of 56% in a 6-copy case.

Mapping and analysis errors are another source of missing variations. For our identifications we solely used the high confidence differences (HCDiffs) extracted by the Reference Mapper software (Roche). Sequence differences in the alignment not fulfilling these requirements escape the SNV identification by our approach. In addition we observed a problem of the Reference Mapper detecting HCDiffs when two differences between 454 read and reference appear at adjacent positions. For example, SNV165 at chr8:7,277,507, used for haplotyping in NA12716 is located beside another polymorphism (SNV164, chr8:7,277,506) not identified as HCDiff (additional file 19). Although we expect only a minute portion of differences missed by this shortcoming it may reduce the completeness of SNV identification.

## Conclusion

By NGS of target enriched DNAs, individual SNPs and SNVs can be assessed even in highly polymorphic and CNV regions such as the human defensin gene locus. SNV identification is highly specific and reveals a considerable amount of putative novel individual polymorphisms. Furthermore it allows simultaneous CN estimations by haplotyping. The sensitivity of the method is limited by sequencing depth and stringency of data filtering. Moreover, the observed lower sensitivity of the SNV detection in samples with higher CN may reflect capture-probe saturation and indicates the need for adjustment of sequencing depth and CN. Despite the costs sequence capture/NGS of copy number variable regions is a valuable source for identifying the growing number of causative variations for phenotypes/diseases.

## Authors' contributions

ST, KS, KH, and MG designed and carried out the Sequence Capture experiments, FR performed the SNV verification.. ST, KS, MF and AP analyzed the data. MP, PR, JH and SS conceived of the study and participated in its design coordination, ST, MP, KS, KH and XZ wrote the manuscript. All authors read and approved the final manuscript.

## Supplementary Material

Additional file 1 **Target regions, tiled (captured) regions and chromosomal positions**. Lengths, chromosomal positions and description of the regions, targeted and tiled by Sequence Capture Click here for file

Additional file 2 **Results of sequencing and HCDiff identification**. 454 sequences, target filtered sequences, sequence depths and HCDiff numbers Click here for file

Additional file 3 **Verification of HCDiffs**. Verification of HCDiffs from NA12760 by PCR, direct sequencing and sequencing after cloning Click here for file

Additional file 4 **Categorization of HCDiffs by variant's allele frequency (VAF)**. Categorization of HCDiffs in the DEFA and CTRL regions of NA12716 and NA12760 by variant's allele frequency (VAF) Click here for file

Additional file 5 **Histogram of error densities**. Histogram of error densities Click here for file

Additional file 6 **Binomial simulation of allele calls from heterozygous sites**. The simulation accounts for the global error rate, and the distribution of local sequence coverage is taken from the real experiment Click here for file

Additional file 7 **Schematic view of the targeted regions DEFA and DEFB (arrows) and their overlap to low copy repeats (LCRs) according to the classification in Ref**. [6]. LCR I contains successive copies of the DEFA1/A3/T1 genes/pseudogenes and has no paralogs elsewhere in the human genome. LCR V has two paralogs, representing the two DEFB copies annotated in the hg18 reference genome. In contrast, LCR IV has additional paralogs with up to 98% nucleotide identity which are enriched together with the targeted DEFB cluster hampering the SNV identification in the LCR IV region. Therefore, all HCDiffs from the targeted part of the LCR IV region (~69 kb) were discarded. Click here for file

Additional file 8 **Filtering of HCDiffs**. Exclusion of 69-kb-segmentally duplicated region, quality filtering following the binomial distribution model, classification by region and variation type and overlap between NA12716 and NA12760 Click here for file

Additional file 9 **Variant's allele frequencies (VAF) before and after filtering**. Variant's allele frequencies (VAF) for the CTRL+DEFA regions of NA12760 before (1,180 HCDiffs) and after (1,108 SNVs) filtering Click here for file

Additional file 10 **HCDiffs identified from NA12716**. HCDiffs identified from NA12716 (CTRL, DEFA, DEFB, after exclusion of indels and complex nucleotide exchanges) Click here for file

Additional file 11 **HCDiffs identified from NA12760**. HCDiffs identified from NA12760 (CTRL, DEFA, DEFB, after exclusion of indels and complex nucleotide exchanges) Click here for file

Additional file 12 **SNVs identified from NA12716 and NA12760**. SNVs identified from NA12716 and NA12760 (CTRL, DEFA, DEFB), after filtering of HCDiffs following workflow in Figure 1 Click here for file

Additional file 13 **Verification of discrepancies Sequence Capture/HapMap**. PCR verification of 14 discrepancies Sequence Capture/HapMap and 6 HapMap SNPs not identified by Sequence Capture Click here for file

Additional file 14 **Haplotyping candidate regions (HTCR)**. Haplotyping candidate regions (HTCR) with high density of polymorphic positions used for haplotyping Click here for file

Additional file 15 **SNVs used for haplotyping within HTCRs**. List of all SNVs within the HTCRs which were used for haplotyping and CN estimation illustrated in additional file 14 Click here for file

Additional file 16 **DEFB haplotype inference and CN estimation**. CN estimation by calculation of the ratios of reads representing the different haplotypes within the HTCRs Click here for file

Additional file 17 **SNVs in the DEFB4 promoter region**. SNVs in the DEFB4 promoter region in comparison to previous data published in Ref. [25] Click here for file

Additional file 18 **DEFB4 Promoter region haplotype inference and CN estimation (NA12760)**. Haplotype calls (HC) and CN estimation based on distal cluster for NA12760/DEFB4 Promoter region (threshold: ≥30 reads) Click here for file

Additional file 19 **Adjacent polymorphisms of which only one is identified by runAssembly**. SNV165 at chr8:7,277,507, used for haplotyping in NA12716 is located beside another polymorphism (SNV164, chr8:7,277,506) not identified as HCDiff by the runAssembly software. Click here for file

## References

[B1] Hollox EJ Barber JC Brookes AJ Armour JA Defensins and the dynamic genome: what we can learn from structural variation at human chromosome band 8p23.1 Genome Res 2008 18 11 1686 1697 10.1101/gr.080945.108 18974263

[B2] Conejo-Garcia JR Benencia F Courreges MC Kang E Mohamed-Hadley A Buckanovich RJ Holtz DO Jenkins A Na H Zhang L Wagner DS Katsaros D Caroll R Coukos G Tumor-infiltrating dendritic cell precursors recruited by a beta-defensin contribute to vasculogenesis under the influence of Vegf-A Nat Med 2004 10 9 950 958 10.1038/nm1097 15334073

[B3] Lehrer RI Primate defensins Nat Rev Microbiol 2004 2 9 727 738 10.1038/nrmicro976 15372083

[B4] Fredman D White SJ Potter S Eichler EE Den Dunnen JT Brookes AJ Complex SNP-related sequence variation in segmental genome duplications Nat Genet 2004 36 8 861 866 10.1038/ng1401 15247918

[B5] Huse K Taudien S Groth M Rosenstiel P Szafranski K Hiller M Hampe J Junker K Schubert J Schreiber S Birkenmeier G Krawczak M Platzer M Genetic variants of the copy number polymorphic beta-defensin locus are associated with sporadic prostate cancer Tumour Biol 2008 29 2 83 92 10.1159/000135688 18515986

[B6] Taudien S Galgoczy P Huse K Reichwald K Schilhabel M Szafranski K Shimizu A Asakawa S Frankish A Loncarevic IF Shimizu N Siddiqui R Platzer M Polymorphic segmental duplications at 8p23.1 challenge the determination of individual defensin gene repertoires and the assembly of a contiguous human reference sequence BMC Genomics 2004 5 1 92 10.1186/1471-2164-5-92 15588320 PMC544879

[B7] Taudien S Groth M Huse K Petzold A Szafranski K Hampe J Rosenstiel P Schreiber S Platzer M Haplotyping and copy number estimation of the highly polymorphic human beta-defensin locus on 8p23 by 454 amplicon sequencing BMC Genomics 2010 11 252 10.1186/1471-2164-11-252 20403190 PMC2873476

[B8] Ahn SM Kim TH Lee S Kim D Ghang H Kim DS Kim BC Kim SY Kim WY Kim C Park D Lee YS Kim S Reja R Jho S Kim CG Cha JY Kim KH Lee B Bhak J Kim SJ The first Korean genome sequence and analysis: full genome sequencing for a socio-ethnic group Genome Res 2009 19 9 1622 1629 10.1101/gr.092197.109 19470904 PMC2752128

[B9] Bentley DR Balasubramanian S Swerdlow HP Smith GP Milton J Brown CG Hall KP Evers DJ Barnes CL Bignell HR Boutell JM Bryant J Carter RJ Keira Cheetham R Cox AJ Ellis DJ Flatbush MR Gormley NA Humphray SJ Irving LJ Karbelashvili MS Kirk SM Li H Liu X Maisinger KS Murray LJ Obradovic B Ost T Parkinson ML Pratt MR Accurate whole human genome sequencing using reversible terminator chemistry Nature 2008 456 7218 53 59 10.1038/nature07517 18987734 PMC2581791

[B10] Kim JI Ju YS Park H Kim S Lee S Yi JH Mudge J Miller NA Hong D Bell CJ Kim HS Chung IS Lee WC Lee JS Seo SH Yun JY Woo HN Lee H Suh D Lee S Kim HJ Yavartanoo M Kwak M Zheng Y Lee MK Park H Kim JY Gokcumen O Mills RE Zaranek AW A highly annotated whole-genome sequence of a Korean individual Nature 2009 460 7258 1011 1015 19587683 10.1038/nature08211 PMC2860965

[B11] Schuster SC Miller W Ratan A Tomsho LP Giardine B Kasson LR Harris RS Petersen DC Zhao F Qi J Alkan C Kidd JM Sun Y Drautz DI Bouffard P Muzny DM Reid JG Nazareth LV Wang Q Burhans R Riemer C Wittekindt NE Moorjani P Tindall EA Danko CG Teo WS Buboltz AM Zhang Z Ma Q Oosthuysen A Complete Khoisan and Bantu genomes from southern Africa Nature 2010 463 7283 943 947 10.1038/nature08795 20164927 PMC3890430

[B12] Wang J Wang W Li R Li Y Tian G Goodman L Fan W Zhang J Li J Zhang J Guo Y Feng B Li H Lu Y Fang X Liang H Du Z Li D Zhao Y Hu Y Yang Z Zheng H Hellmann I Inouye M Pool J Yi X Zhao J Duan J Zhou Y Qin J The diploid genome sequence of an Asian individual Nature 2008 456 7218 60 65 10.1038/nature07484 18987735 PMC2716080

[B13] Wheeler DA Srinivasan M Egholm M Shen Y Chen L McGuire A He W Chen YJ Makhijani V Roth GT Gomes X Tartaro K Niazi F Turcotte CL Irzyk GP Lupski JR Chinault C Song XZ Liu Y Yuan Y Nazareth L Qin X Muzny DM Margulies M Weinstock GM Gibbs RA Rothberg JM The complete genome of an individual by massively parallel DNA sequencing Nature 2008 452 7189 872 876 10.1038/nature06884 18421352

[B14] Okou DT Steinberg KM Middle C Cutler DJ Albert TJ Zwick ME Microarray-based genomic selection for high-throughput resequencing Nat Methods 2007 4 11 907 909 10.1038/nmeth1109 17934469

[B15] Porreca GJ Zhang K Li JB Xie B Austin D Vassallo SL LeProust EM Peck BJ Emig CJ Dahl F Gao Y Church GM Shendure J Multiplex amplification of large sets of human exons Nat Methods 2007 4 11 931 936 10.1038/nmeth1110 17934468

[B16] Albert TJ Molla MN Muzny DM Nazareth L Wheeler D Song X Richmond TA Middle CM Rodesch MJ Packard CJ Weinstock GM Gibbs RA Direct selection of human genomic loci by microarray hybridization Nat Methods 2007 4 11 903 905 10.1038/nmeth1111 17934467

[B17] Hodges E Xuan Z Balija V Kramer M Molla MN Smith SW Middle CM Rodesch MJ Albert TJ Hannon GJ McCombie WR Genome-wide in situ exon capture for selective resequencing Nat Genet 2007 39 12 1522 1527 10.1038/ng.2007.42 17982454

[B18] Schracke N Kornmeyer T Kranzle M Stahler PF Summerer D Beier M Specific sequence selection and next generation resequencing of 68 E. coli genes using HybSelect N Biotechnol 2009 26 5 229 233 10.1016/j.nbt.2009.08.013 19735750

[B19] Summerer D HybSelect: high-throughput access to genomic regions of interest for targeted next-generation sequencing Nature Methods 2009 6 v vi

[B20] Summerer D Wu H Haase B Cheng Y Schracke N Stahler CF Chee MS Stahler PF Beier M Microarray-based multicycle-enrichment of genomic subsets for targeted next-generation sequencing Genome Res 2009 19 9 1616 1621 10.1101/gr.091942.109 19638418 PMC2752126

[B21] Bainbridge MN Wang M Burgess DL Kovar C Rodesch MJ D'Ascenzo M Kitzman J Wu YQ Newsham I Richmond TA Jeddeloh JA Muzny D Albert TJ Gibbs RA Whole exome capture in solution with 3 Gbp of data Genome Biol 2010 11 6 R62 10.1186/gb-2010-11-6-r62 20565776 PMC2911110

[B22] Gnirke A Melnikov A Maguire J Rogov P LeProust EM Brockman W Fennell T Giannoukos G Fisher S Russ C Gabriel S Jaffe DB Lander ES Nusbaum C Solution hybrid selection with ultra-long oligonucleotides for massively parallel targeted sequencing Nat Biotechnol 2009 27 2 182 189 10.1038/nbt.1523 19182786 PMC2663421

[B23] Hardwick RJ Machado LR Zuccherato LW Antolinos S Xue Y Shawa N Gilman RH Cabrera L Berg DE Tyler-Smith C Kelly P Tarazona-Santos E Hollox EJ A worldwide analysis of beta-defensin copy number variation suggests recent selection of a high-expressing DEFB103 gene copy in East Asia Hum Mutat 2011 10.1002/humu.21491 PMC3263423 21387465

[B24] Armour JA Palla R Zeeuwen PL den Heijer M Schalkwijk J Hollox EJ Accurate, high-throughput typing of copy number variation using paralogue ratios from dispersed repeats Nucleic Acids Res 2007 35 3 e19 10.1093/nar/gkl1089 17175532 PMC1807953

[B25] Groth M Wiegand C Szafranski K Huse K Kramer M Rosenstiel P Schreiber S Norgauer J Platzer M Both copy number and sequence variations affect expression of human DEFB4 Genes Immun 2010 11 6 458 466 10.1038/gene.2010.19 20445567

[B26] The International HapMap Project http://hapmap.ncbi.nlm.nih.gov/

[B27] MRC Holland http://www.mrc-holland.com

[B28] Meyer M Briggs AW Maricic T Hober B Hoffner B Krause J Weihmann A Paabo S Hofreiter M From micrograms to picograms: quantitative PCR reduces the material demands of high-throughput sequencing Nucleic Acids Res 2008 36 1 e5 18084031 10.1093/nar/gkm1095 PMC2248761

[B29] Kent WJ BLAT-the BLAST-like alignment tool Genome Res 2002 12 4 656 664 11932250 10.1101/gr.229202 PMC187518

[B30] Brookes AJ Prince JA Genetic association analysis: lessons from the study of Alzheimers disease Mutat Res 2005 573 1-2 152 159 15829244 10.1016/j.mrfmmm.2004.08.017

[B31] Chmielecki J Peifer M Jia P Socci ND Hutchinson K Viale A Zhao Z Thomas RK Pao W Targeted next-generation sequencing of DNA regions proximal to a conserved GXGXXG signaling motif enables systematic discovery of tyrosine kinase fusions in cancer Nucleic Acids Res 2010 10.1093/nar/gkq579 PMC2978357 20587502

[B32] Kim DW Nam SH Kim RN Choi SH Park HS Whole human exome capture for high-throughput sequencing Genome 2010 53 7 568 574 10.1139/G10-025 20616878

[B33] Rehman AU Morell RJ Belyantseva IA Khan SY Boger ET Shahzad M Ahmed ZM Riazuddin S Khan SN Riazuddin S Friedman TB Targeted capture and next-generation sequencing identifies C9orf75, encoding taperin, as the mutated gene in nonsyndromic deafness DFNB79 Am J Hum Genet 2010 86 3 378 388 10.1016/j.ajhg.2010.01.030 20170899 PMC2833391

[B34] Choi M Scholl UI Ji W Liu T Tikhonova IR Zumbo P Nayir A Bakkaloglu A Ozen S Sanjad S Nelson-Williams C Farhi A Mane S Lifton RP Genetic diagnosis by whole exome capture and massively parallel DNA sequencing Proc Natl Acad Sci USA 2009 106 45 19096 19101 10.1073/pnas.0910672106 19861545 PMC2768590

[B35] Groth M Szafranski K Taudien S Huse K Mueller O Rosenstiel P Nygren AO Schreiber S Birkenmeier G Platzer M High-resolution mapping of the 8p23.1 beta-defensin cluster reveals strictly concordant copy number variation of all genes Hum Mutat 2008 29 10 1247 1254 10.1002/humu.20751 18470942

[B36] Ewing B Green P Base-calling of automated sequencer traces using phred. II. Error probabilities Genome Res 1998 8 3 186 194 9521922

[B37] Ewing B Hillier L Wendl MC Green P Base-calling of automated sequencer traces using phred. I. Accuracy assessment Genome Res 1998 8 3 175 185 9521921 10.1101/gr.8.3.175

