# Supplementary material for: Comprehensive assessment of sequence variation within the copy number variable defensin cluster on 8p23 by target enriched in-depth 454 sequencing
Source: BMC Genomics. 2011 May 18;12:243. doi: 10.1186/1471-2164-12-243 (PMC3118217; doi:10.1186/1471-2164-12-243)
Supplement: Additional file 1 — Target regions, tiled (captured) regions and chromosomal positions. Lengths, chromosomal positions and description of the regions, targeted and tiled by Sequence Capture [file 1471-2164-12-243-S1.PDF]

additional file 1: Target regions, tiled (captured) regions and chromosomal positions

region	target (bp)	tiled (bp)	f (tiled)	target positions (hg18)	description / gene
CTRL	63.612	55.690		chr8:6,344,581-6,408,192	*ANGPT2/MCPH1*
CTRL	26.998	24.337		chr8:6,653,450-6,680,447	*XKR5*
CTRL	64.000	51.918		chr8:8,212,668-8,276,667	*SGK223 (PRAGMIN)*
CTRL	109.133	86.933		chr8:8,679,409-8,788,541	*MFHAS1*
CTRL	25.613	18.747		chr8:11,737,443-11,763,055	*CTSB*
CTRL	33.587	32.228		chr8:12,623,777-12,657,363	*LONRF1*
CTRL	6.986	5.543		chr17:31,284,638-31,291,623	*LYZL6*
CTRL	7.723	5.899		chr19:59,864,936-59,872,658	*LILRB4*
CTRL	18.291	9.600		chr19:60,076,361-60,094,651	*FCAR*
CTRL	9.604	7.897		chr20:29,526,766-29,536,369	*REM1*
CTRL	55.130	39.804		chr20:29,565,902-29,621,031	*HM13*
all CTRL loci	420.677	338.596	0,80		
DEFA	195.276	179.652	0,92	chr8: 6,714,511-6,909,786	DEF cluster A
DEFB*)	234.499	175.346	0,75	chr8:7,156,778-7,391,276	distal DEF cluster B
other loci	2.187.563	1.343.744			
**all loci**	**3.038.015**	**2.037.338**	**0,67**		
**CTRL+DEFA+DEFB**	**850.452**	**693.594**	**0,82**		

*)	target (bp)	tiled (bp)	f (tiled)	target positions (hg18)	comment
DEFB_segdup	69.092	38.229	0,55	chr8:7,156,778-7,225,869	additional paralogs at chr4, chr8(12Mb), chr11
DEFB	165.407	137.117	0,83	chr8:7,225,870-7,391,276	
