# Supplementary material for: Comprehensive assessment of sequence variation within the copy number variable defensin cluster on 8p23 by target enriched in-depth 454 sequencing
Source: BMC Genomics. 2011 May 18;12:243. doi: 10.1186/1471-2164-12-243 (PMC3118217; doi:10.1186/1471-2164-12-243)
Supplement: Additional file 2 — Results of sequencing and HCDiff identification. 454 sequences, target filtered sequences, sequence depths and HCDiff numbers [file 1471-2164-12-243-S2.PDF]

additional file 2: Results of sequencing and HCDiff identification

A) 454 sequences

#	DNA	reads	bp	avlen (bp)
1	NA12716	1.497.580	450.621.018	301
2	NA12760	931.236	300.702.895	323

B) Target filtered sequences

#	DNA	targets	reads	bp	f (reads)	enr1)	depth1)	replicates	duplicates
1	NA12716	all	696.943	237.538.030	0,47	456	78	0,07%	22,03%
2	NA12760	all	475.254	170.500.794	0,51	501	56	0,06%	14,37%
1	NA12716	CTRL,DEFA,DEFB	199.462	68.732.181	0,13	470	81	n.d.	n.d.
2	NA12760	CTRL,DEFA,DEFB	167.102	60.499.569	0,18	633	71	n.d.	n.d.

1) based on total target length 3,058,638 bp (all targets) and 850,452 bp (DEFA,DEFB,CTRL) and 3 Gb genome size

C) Average sequencing depths with respect to captured lengths (DEFA,DEFB,CTRL)

#	DNA	CTRL	DEFA	DEFB
1	NA12716	59,9	56,0	45,3
2	NA12760	44,5	37,0	99,5

D) Target and chromosomal filtered sequences

#	DNA	reads	bp	f of target filtered
1	NA12716	691.348	235.983.109	0,992
2	NA12760	472.346	169.538.640	0,994

E) High confidence differences (HCDiffs), after exclusion of indels and complex nt exchanges

#	DNA	targets	HCDiffs
1	NA12716	all	21.306
2	NA12760	all	20.149
1	NA12716	CTRL,DEFA,DEFB	3.161
2	NA12760	CTRL,DEFA,DEFB	3.490
