# Supplementary material for: Comprehensive assessment of sequence variation within the copy number variable defensin cluster on 8p23 by target enriched in-depth 454 sequencing
Source: BMC Genomics. 2011 May 18;12:243. doi: 10.1186/1471-2164-12-243 (PMC3118217; doi:10.1186/1471-2164-12-243)
Supplement: Additional file 3 — Verification of HCDiffs. Verification of HCDiffs from NA12760 by PCR, direct sequencing and sequencing after cloning [file 1471-2164-12-243-S3.PDF]

**additional file3: Verification of HCDiffs**

het = heterozygote, signal from both reference and variant's alleles / hom = homozygote

| # | Region | chr | hg18 | Gene | 454 sequencing | | | | | PCR and Sanger sequencing | | | | | Primer (5'->3') | |
|---|---|---|---|---|---|---|---|---|---|---|---|---|---|---|---|---|
| | | | | | Ref | Var | #reads | VAF | P | direct | CL | alleles | VAF | confirm | forward | reverse |
| 1 | DEFA | chr8 | 6.737.740 | | T | G | 30 | 40% | 2,51E-012 | GT | 66 | 42T/24G | 36% | YES | TGCAGGTGTTAAATCTCTTG | CTGAGCCTGCTGTCCTC |
| 3 | | chr8 | 6.777.846 | | G | A | 64 | 41% | 1,00E-012 | AG | 5 | 4G/1A | | YES | GTTGTTTGGAGACCCTCAC | GAATGTAGAATCAGGGCAAG |
| 4 | | chr8 | 6.793.802 | | C | T | 34 | 50% | 1,00E-012 | CT | 25 | 14C/11T | 44% | YES | TTGAGGAAGAACACAGTGAC | TTTAGACAGTGACGGTGATG |
| 5 | | chr8 | 6.818.340 | | C | A | 37 | 76% | 1,00E-012 | AC | | | | YES | TTTTTCCAGCACTCCAC | AGGCATCAGAGGAGAAGTG |
| 6 | | chr8 | 6.871.262 | | C | T | 32 | 47% | 1,00E-012 | CT | | | | YES | GTGTCCTCCTTGAGGTCTG | CCAAGAACCCTTACAGTGAC |
| 7 | | chr8 | 6.888.529 | | G | C | 17 | 65% | 1,00E-012 | CG | | | | YES | ACATCGTTTTGACCTGGTAG | TAGACAGCTTGTGAGAGGTG |
| 1 | DEFB | chr8 | 7.261.330 | *DEFB4* | G | A | 104 | 13% | 1,32E-007 | GG | 98 | 98G/-- | --- | NO | CCCGCCTTCCTTCATTATAC | CCCACACTTTTAGACTGA |
| 2 | | chr8 | 7.262.687 | | C | T | 44 | 23% | 5,02E-008 | CT | 112 | 93C/19T | 17% | YES | GAACTTCTACGCCATTCTTCC | AGAGACACCAGAGACCTCACAC |
| 3 | | chr8 | 7.274.285 | *DEFB103* | G | T | 104 | 17% | 4,09E-011 | GT | 50 | 38G/12T | 24% | YES | TCAAATAACTCATCCCTTGC | CTCTGAGAATAGAAGCACAGG |
| 4 | | chr8 | 7.287.713 | | T | C | 36 | 58% | 1,00E-012 | CT | 93 | 29T/64C | 69% | YES | AGTGGAGGAGTCACAGGAG | TTAGCCTCCCAAATACCTG |
| 5 | | chr8 | 7.315.804 | *DEFB104* | C | A | 120 | 20% | 1,00E-012 | AC | 88 | 70C/18A | 20% | YES | TTCCATGTCACACTTATTTTC | AAGGAAGAAAAACCTTCCAG |
| 6 | | chr8 | 7.316.428 | *DEFB104* | C | T | 97 | 96% | 1,00E-012 | TT | | | | YES | AAGGACGTGGTGAGTAAATG | CTATCAGTGTGGCATGTGAG |
| 7 | | chr8 | 7.317.261 | *DEFB104* | C | A | 97 | 97% | 1,00E-012 | AA | | | | YES | GTAGGTGGAGGTTGAGTAGC | GTTTCTGTTCCAGCTTTGAC |
| 8 | | chr8 | 7.319.333 | *DEFB104* | T | C | 98 | 18% | 1,42E-011 | CT | 66 | 57T/9C | 14% | YES | GTGCTCACTCATATCCATTG | ATTAGGATTAAAGGGGATGC |
| 1 | CTRL | chr8 | 6.373.518 | *ANGPT2* | C | T | 60 | 53% | 1,00E-012 | CT | | | | YES | TAATTTTTAGCCTGGGATTTC | AACAATGCTGACTGACATTTG |
| 2 | | chr8 | 6.388.961 | *ANGPT2* | T | C | 65 | 58% | 1,00E-012 | CT | | | | YES | AGATTATTTAACGGGGCTATG | TCGTACTTGTTACACTTCCTACC |
| 3 | | chr8 | 8.695.314 | *MFHAS1* | T | A | 66 | 44% | 1,00E-012 | AT | | | | YES | AACAGCAAACTGCTACAAAAG | GGTGGGTTGGTTTACAGAC |
| 4 | | chr8 | 8.739.698 | *MFHAS1* | G | C | 72 | 47% | 1,00E-012 | CG | | | | YES | TATCCCCTGAACCTCTCTTAC | CTTGAAAATACAGCAACAACC |
| 5 | | chr8 | 8.743.837 | *MFHAS1* | A | T | 24 | 67% | 1,00E-012 | AT | | | | YES | TTAATCAAACAGTGCGAAGTC | TGTACCTTTACCCTGCTAGTG |
| 6 | | chr8 | 8.744.430 | *MFHAS1* | G | A | 34 | 32% | 1,76E-010 | AG | | | | YES | TGTAGCAAGTAATTCTGAAAGC | TCATTTCTTGACCAGGAGAC |
| 7 | | chr8 | 11.755.710 | *CTSB* | T | C | 32 | 47% | 1,00E-012 | CT | | | | YES | AGTCAAGTGCTTACATTTTGC | AAGTAGGGGAAGATGAAACTC |

22
