# Supplementary figures and images for: Comprehensive assessment of sequence variation within the copy number variable defensin cluster on 8p23 by target enriched in-depth 454 sequencing
Source: BMC Genomics. 2011 May 18;12:243. doi: 10.1186/1471-2164-12-243 (PMC3118217; doi:10.1186/1471-2164-12-243)

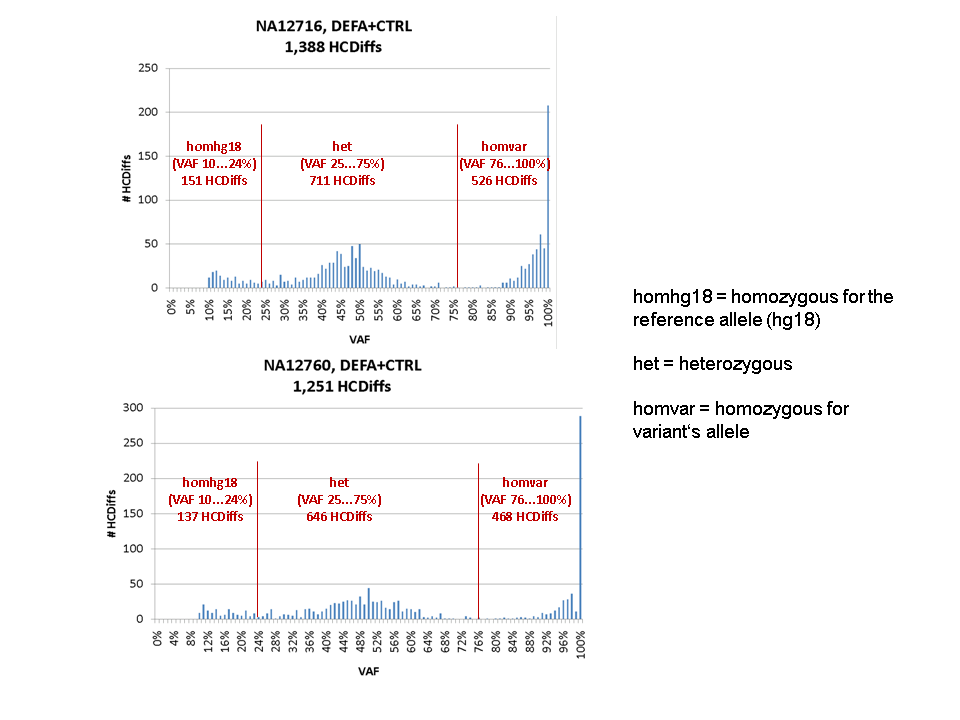

Supplement: Additional file 4 — Categorization of HCDiffs by variant's allele frequency (VAF). Categorization of HCDiffs in the DEFA and CTRL regions of NA12716 and NA12760 by variant's allele frequency (VAF) [file 1471-2164-12-243-S4.TIFF]

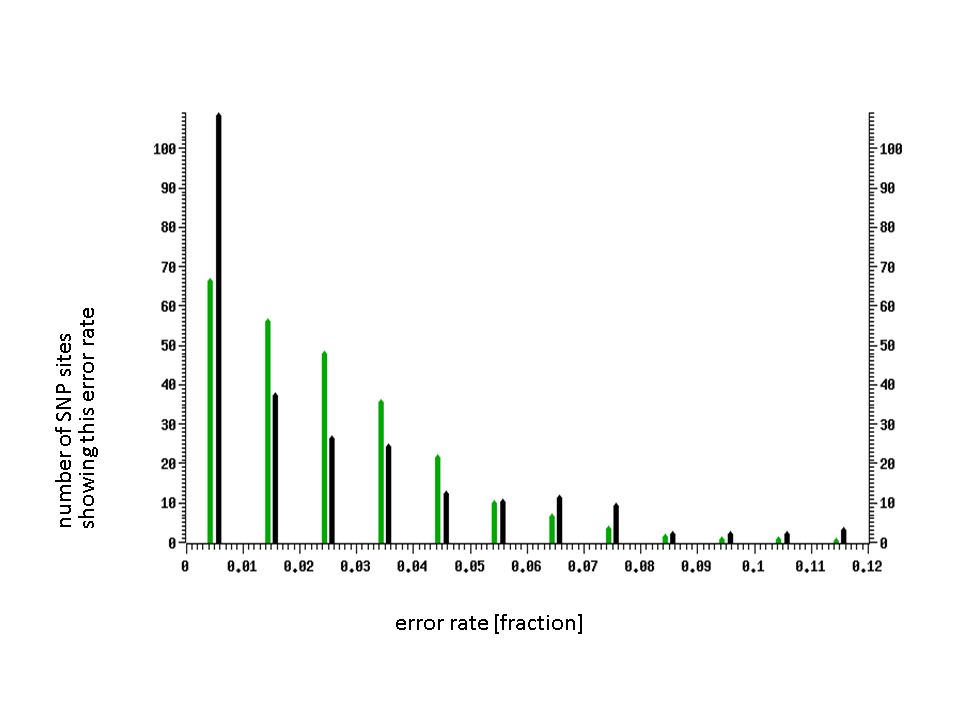

Supplement: Additional file 5 — Histogram of error densities. Histogram of error densities [file 1471-2164-12-243-S5.TIFF]

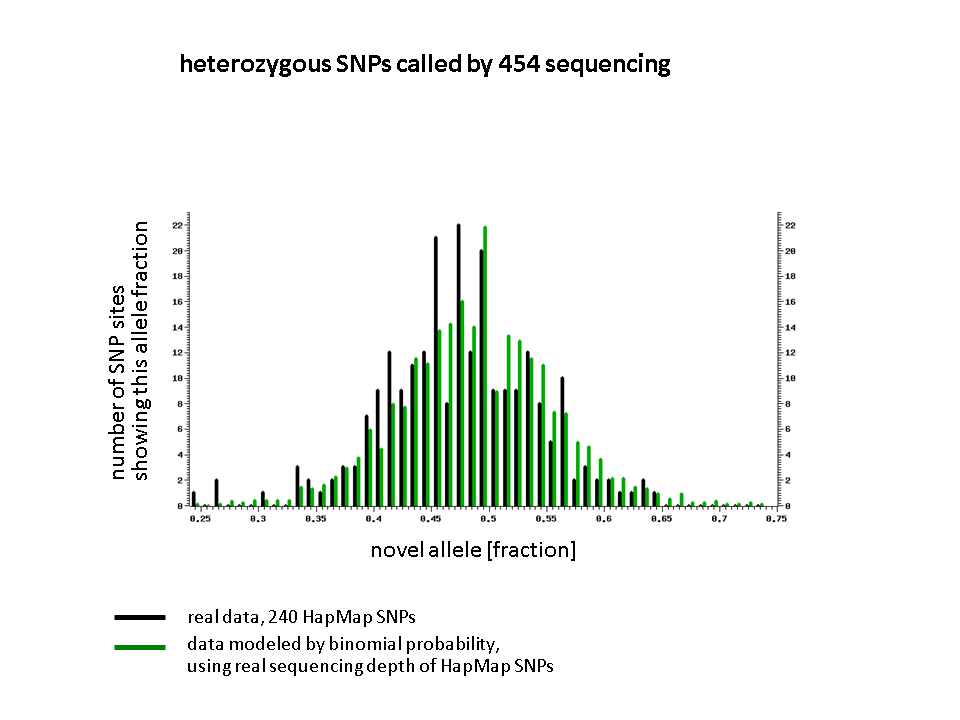

Supplement: Additional file 6 — Binomial simulation of allele calls from heterozygous sites. The simulation accounts for the global error rate, and the distribution of local sequence coverage is taken from the real experiment [file 1471-2164-12-243-S6.TIFF]

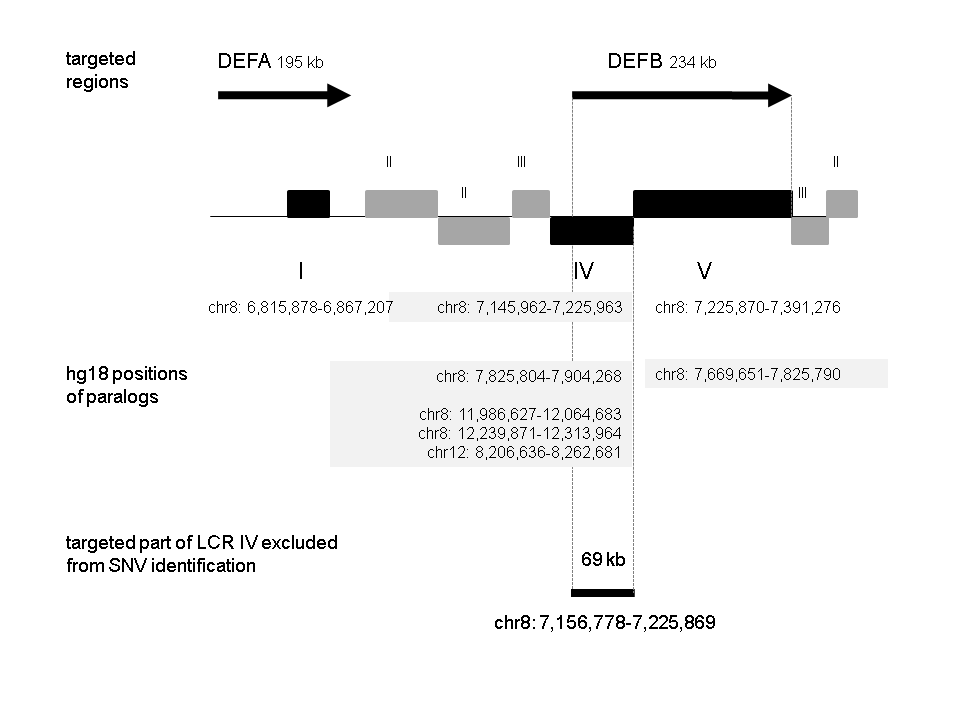

Supplement: Additional file 7 — Schematic view of the targeted regions DEFA and DEFB (arrows) and their overlap to low copy repeats (LCRs) according to the classification in Ref. [6]. LCR I contains successive copies of the DEFA1/A3/T1 genes/pseudogenes and has no paralogs elsewhere in the human genome. LCR V has two paralogs, representing the two DEFB copies annotated in the hg18 reference genome. In contrast, LCR IV has additional paralogs with up to 98% nucleotide identity which are enriched together with the targeted DEFB cluster hampering the SNV identification in the LCR IV region. Therefore, all HCDiffs from the targeted part of the LCR IV region (~69 kb) were discarded. [file 1471-2164-12-243-S7.TIFF]

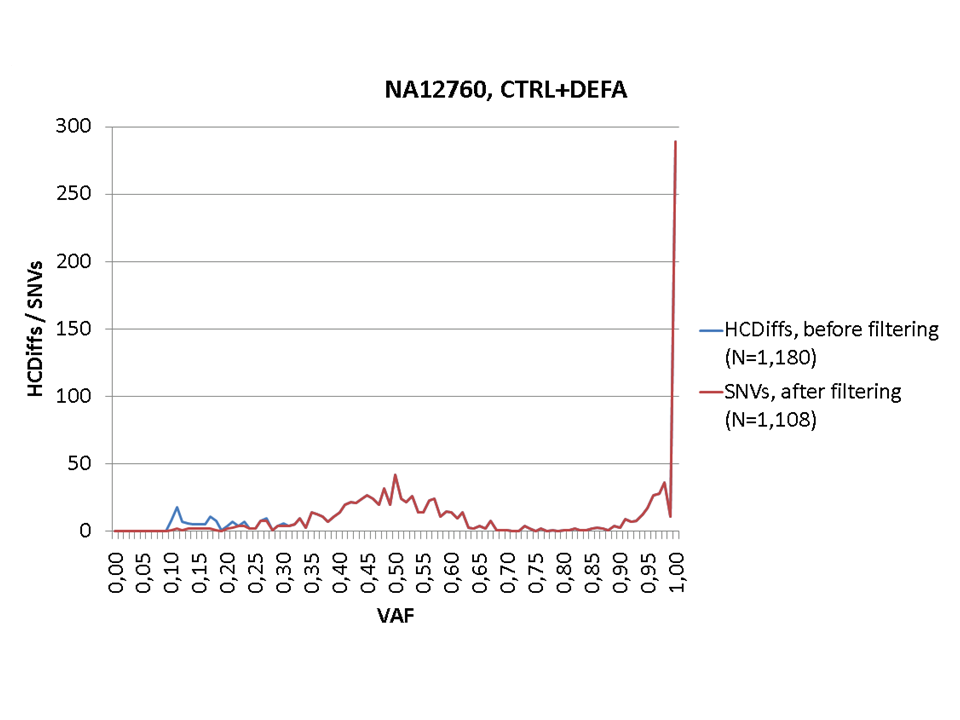

Supplement: Additional file 9 — Variant's allele frequencies (VAF) before and after filtering. Variant's allele frequencies (VAF) for the CTRL+DEFA regions of NA12760 before (1,180 HCDiffs) and after (1,108 SNVs) filtering [file 1471-2164-12-243-S9.TIFF]

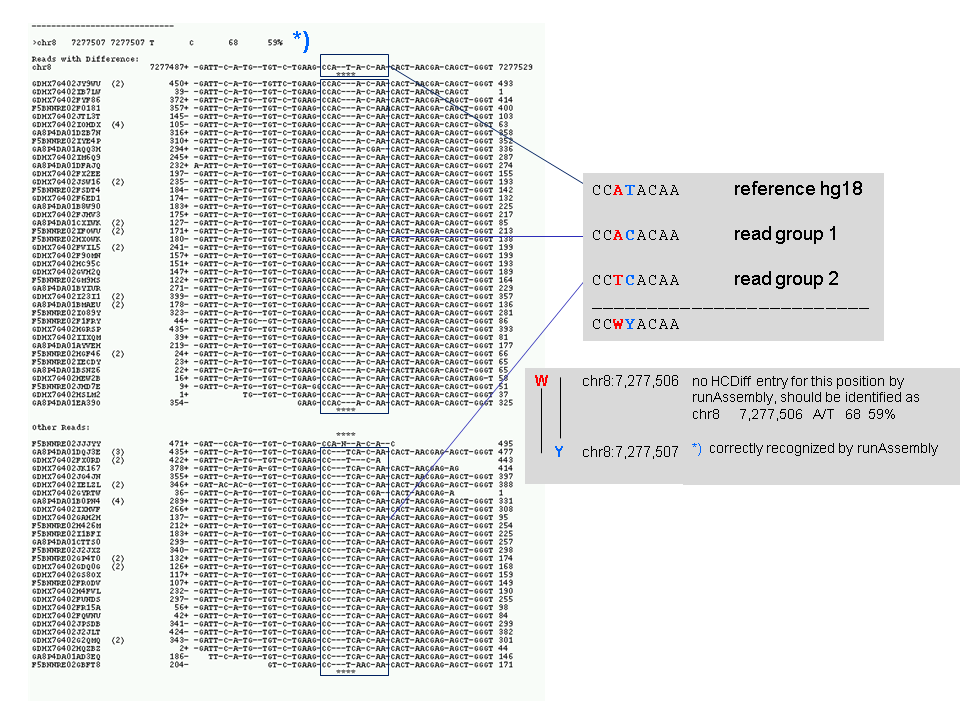

Supplement: Additional file 19 — Adjacent polymorphisms of which only one is identified by runAssembly. SNV165 at chr8:7,277,507, used for haplotyping in NA12716 is located beside another polymorphism (SNV164, chr8:7,277,506) not identified as HCDiff by the runAssembly software. [file 1471-2164-12-243-S19.TIFF]
