# Supplementary material for: Comprehensive assessment of sequence variation within the copy number variable defensin cluster on 8p23 by target enriched in-depth 454 sequencing
Source: BMC Genomics. 2011 May 18;12:243. doi: 10.1186/1471-2164-12-243 (PMC3118217; doi:10.1186/1471-2164-12-243)
Supplement: Additional file 8 — Filtering of HCDiffs. Exclusion of 69-kb-segmentally duplicated region, quality filtering following the binomial distribution model, classification by region and variation type and overlap between NA12716 and NA12760 [file 1471-2164-12-243-S8.PDF]

additional file 8: Filtering of HCDiffs

target region pos	gene/cluster	segdup paralogs	segdup affected region with HCDiffs to exclude	affected HCDiffs to exclude	
	DEFB	chr4, chr8 (12Mb), chr11	chr8:7,156,778-7,225,869	NA12716	1.196
				NA12760	1.121

HCDiff probability values by the binomial distribution model

	DNA	all	P<=10-3		P>10-3	
HCDiffs	NA12716	3.161	3.017	0,954	144	0,046
	NA12760	3.490	3.297	0,945	193	0,055
removed HCDiffs from DEFB region with segmental duplications beside the chr8p23.1 paralogs	NA12716	1.196	1.098	0,918	98	0,082
	NA12760	1.121	1.032	0,921	89	0,079
after removal	NA12716	1.965	1.919	0,977	46	0,023
	NA12760	2.369	2.265	0,956	104	0,044

Successive filtering of HCDiffs

HCDiffs	NA12716	3.161	100%
	NA12760	3.490	100%
after removal of HCDiffs from DEFB region with segmental duplications beside the chr8p23.1 paralogs	NA12716	1.965	62%
	NA12760	2.369	68%
after removal of HCDiffs with $P>10^{-3}$	NA12716	1.919	61%
	NA12760	2.265	65%

Sub-classification of SNVs by region and variation type

	region		ambiguous VAF 0.10-0.24	het VAF 0.25-0.75			homvar VAF 0.76-1.00		total
NA12716	CTRL	known	6	282			286		574
	CTRL	putnov	7	29			5		41
	DEFA	known	26	366			212		604
	DEFA	putnov	13	14			0		27
	DEFB	known	5	420			135		560
	DEFB	putnov	2	54			57		113
		total	59	1.165			695		1.919
		fraction	0,03	0,61			0,36		
NA12760	CTRL	known	4	356			235		595
	CTRL	putnov	6	39			3		48
	DEFA	known	13	189			226		428
	DEFA	putnov	7	26			4		37
		total	30	610			468		1.108
		fraction	0,03	0,55			0,42		
	DEFB	variant copies	1of6 VAF 0.10-0.24	2of6 0.25-0.42	3of6 0.43-0.58	4of6 0.59-0.75	5of6 0.76-0.92	6of6 0.93-1.00	total
		known	405	244	150	72	46	62	
		putnov	102	13	6	17	13	27	
		total	507	257	156	89	59	89	1.157
		fraction	0,438202247	0,22212619	0,134831	0,07692	0,0509939	0,07692	

Overlap of SNVs between NA12716 and NA12760

	Total	DEFA known	putnov	DEFB known	putnov	CTRL known	putnov
NA12716	1.919	604	27	560	113	574	41
NA12760	2.265	428	37	979	178	595	48
SNVs identical in both DNAs		331	3	483	71	398	12
SNVs exclusively in NA12716		273	24	77	42	176	29
SNVs exclusively in NA12760		97	34	496	107	197	36
unique SNVs		701	61	1.056	220	771	77
unique SNVs, total	2.886	762		1.276		848	
