# Supplementary material for: Comprehensive assessment of sequence variation within the copy number variable defensin cluster on 8p23 by target enriched in-depth 454 sequencing
Source: BMC Genomics. 2011 May 18;12:243. doi: 10.1186/1471-2164-12-243 (PMC3118217; doi:10.1186/1471-2164-12-243)
Supplement: Additional file 10 — HCDiffs identified from NA12716. HCDiffs identified from NA12716 (CTRL, DEFA, DEFB, after exclusion of indels and complex nucleotide exchanges) [file 1471-2164-12-243-S10.PDF]

additional file 10: HCDiffs identified from NA12716

dep_tot= sequence depth total / dep_var = sequence depth of variant / VAF = variant's allele frequency / aa = amino acid

region	segdup	hg18	pos	ref	var	dep_tot	VAF	var_type	dep_var	aa_ref	aa_var	strand	gene	SNPhg18	SNPhg18	SNPblat	P
reg_CTRL		chr17	31285944	A	G	75	49,00%	het	37	F	S	-3	LYZL6	rs9754			1,00E-012
reg_CTRL		chr17	31287000	G	C	8	37,00%	het	3			-	LYZL6	rs11654713			6,25E-004
reg_CTRL		chr19	59865309	A	G	36	47,00%	het	17					rs1749311			1,00E-012
reg_CTRL		chr19	59865333	A	G	39	100,00%	homvar	39					rs430978			1,00E-012
reg_CTRL		chr19	59865618	C	G	68	100,00%	homvar	68					rs413679			1,00E-012
reg_CTRL		chr19	59865626	G	C	67	97,00%	homvar	65					rs12609815			1,00E-012
reg_CTRL		chr19	59866025	C	T	41	44,00%	het	18			+	LILRB4	rs1654668			1,00E-012
reg_CTRL		chr19	59866821	G	T	3	100,00%	homvar	3	R	S	2	LILRB4	rs11574570			1,22E-005
reg_CTRL		chr19	59867552	C	T	23	30,00%	het	7	F	F	2	LILRB4	rs3745871			6,03E-007
reg_CTRL		chr19	59868074	A	G	24	100,00%	homvar	24	D	G	2	LILRB4	rs731170			1,00E-012
reg_CTRL		chr19	59868347	C	T	18	33,00%	het	6			+	LILRB4	rs1925241			2,17E-006
reg_CTRL		chr19	59868514	A	C	11	45,00%	het	5			+	LILRB4	rs1631746			2,65E-006
reg_CTRL		chr19	59868598	C	T	11	36,00%	het	4			+	LILRB4	rs1749316			8,11E-005
reg_CTRL		chr19	59869974	A	G	16	19,00%	ambig	3	N	D	2	LILRB4	rs11574576			5,44E-003
reg_CTRL		chr19	59870000	A	G	16	19,00%	ambig	3	E	E	2	LILRB4				5,44E-003
reg_CTRL		chr19	59870069	T	A	19	21,00%	ambig	4			+	LILRB4			rs393665	8,22E-004
reg_CTRL		chr19	59870138	C	T	21	14,00%	ambig	3			+	LILRB4	rs45561539	rs11574578		1,19E-002
reg_CTRL		chr19	59870148	T	C	21	38,00%	het	8			+	LILRB4				1,22E-008
reg_CTRL		chr19	59870157	G	T	21	24,00%	ambig	5			+	LILRB4				9,62E-005
reg_CTRL		chr19	59870171	C	T	20	30,00%	het	6			+	LILRB4				4,35E-006
reg_CTRL		chr19	59870190	A	G	20	50,00%	het	10			+	LILRB4				9,66E-012
reg_CTRL		chr19	59870239	A	G	19	47,00%	het	9			+	LILRB4				1,37E-010
reg_CTRL		chr19	59870279	C	G	21	24,00%	ambig	5			+	LILRB4				9,62E-005
reg_CTRL		chr19	59870298	A	G	22	45,00%	het	10			+	LILRB4				3,38E-011
reg_CTRL		chr19	59870306	G	A	23	17,00%	ambig	4			+	LILRB4				1,75E-003
reg_CTRL		chr19	59870313	C	T	23	17,00%	ambig	4			+	LILRB4				1,75E-003
reg_CTRL		chr19	59870327	T	A	24	29,00%	het	7			+	LILRB4			rs3865478	8,35E-007
reg_CTRL		chr19	59870329	T	C	24	29,00%	het	7			+	LILRB4				8,35E-007
reg_CTRL		chr19	59870332	T	C	24	33,00%	het	8			+	LILRB4				4,14E-008
reg_CTRL		chr19	59870428	G	A	28	39,00%	het	11			+	LILRB4			rs443874	2,64E-011
reg_CTRL		chr19	59870494	A	C	26	50,00%	het	13			+	LILRB4			rs3893859	1,00E-012
reg_CTRL		chr19	59870501	T	G	25	56,00%	het	14			+	LILRB4				1,00E-012
reg_CTRL		chr19	59870545	A	G	31	55,00%	het	17			+	LILRB4	rs11574582			1,00E-012
reg_CTRL		chr19	59870587	G	A	29	48,00%	het	14			+	LILRB4				1,00E-012
reg_CTRL		chr19	59870591	A	T	28	46,00%	het	13			+	LILRB4			rs62133430	1,00E-012
reg_CTRL		chr19	59870609	A	G	31	45,00%	het	14			+	LILRB4			rs73933940	1,00E-012
reg_CTRL		chr19	59870612	G	A	30	17,00%	ambig	5			+	LILRB4				5,67E-004
reg_CTRL		chr19	59870640	G	A	31	13,00%	ambig	4			+	LILRB4				5,36E-003
reg_CTRL		chr19	59870677	A	G	34	21,00%	ambig	7			+	LILRB4			rs685381	1,06E-005

reg_CTRL	chr19	59870745	G	C	37	14,00%	ambig	5			+	LILRB4				1,52E-003
reg_CTRL	chr19	59870769	G	A	38	11,00%	ambig	4			+	LILRB4				1,11E-002
reg_CTRL	chr19	59870794	G	A	36	61,00%	het	22			+	LILRB4	rs11574587			1,00E-012
reg_CTRL	chr19	59870797	A	G	35	29,00%	het	10			+	LILRB4			rs41308138	4,50E-009
reg_CTRL	chr19	59870823	T	C	35	43,00%	het	15			+	LILRB4			rs71365476	1,00E-012
reg_CTRL	chr19	59870834	G	A	35	66,00%	het	23			+	LILRB4				1,00E-012
reg_CTRL	chr19	59870860	A	G	41	34,00%	het	14			+	LILRB4				1,00E-012
reg_CTRL	chr19	59870864	T	C	40	45,00%	het	18			+	LILRB4				1,00E-012
reg_CTRL	chr19	59870909	C	T	40	10,00%	ambig	4	H	H	1	LILRB4				1,32E-002
reg_CTRL	chr19	59870940	A	G	37	19,00%	ambig	7	K	E	1	LILRB4	rs2764337	rs11574589		1,91E-005
reg_CTRL	chr19	59870957	A	T	35	34,00%	het	12	R	S	1	LILRB4			rs61738946	2,42E-011
reg_CTRL	chr19	59870988	C	T	37	30,00%	het	11	P	S	1	LILRB4			rs61743356	4,70E-010
reg_CTRL	chr19	59870996	T	C	35	11,00%	ambig	4	S	S	1	LILRB4				8,30E-003
reg_CTRL	chr19	59871028	C	T	36	25,00%	het	9	A	V	1	LILRB4	rs11574591			9,66E-008
reg_CTRL	chr19	59871029	A	G	36	39,00%	het	14	A	A	1	LILRB4	rs11574591			1,00E-012
reg_CTRL	chr19	59871041	A	G	36	47,00%	het	17	R	R	1	LILRB4	rs11574592			1,00E-012
reg_CTRL	chr19	59871057	G	A	36	17,00%	ambig	6			+	LILRB4				1,59E-004
reg_CTRL	chr19	59871112	C	A	28	25,00%	het	7			+	LILRB4			rs57804895	2,63E-006
reg_CTRL	chr19	59871189	T	G	35	17,00%	ambig	6	F	L	1	LILRB4			rs634222	1,35E-004
reg_CTRL	chr19	59871200	A	G	35	14,00%	ambig	5	Q	R	1	LILRB4	rs534749			1,17E-003
reg_CTRL	chr19	60076609	C	A	17	41,00%	het	7					rs11084374			5,41E-008
reg_CTRL	chr19	60077218	G	A	49	55,00%	het	27					rs17772004			1,00E-012
reg_CTRL	chr19	60077247	T	C	45	49,00%	het	22					rs12462181			1,00E-012
reg_CTRL	chr19	60077416	T	C	71	46,00%	het	33			+	FCAR	rs3816051			1,00E-012
reg_CTRL	chr19	60078436	C	G	28	39,00%	het	11			+	FCAR	rs11084376			2,64E-011
reg_CTRL	chr19	60078702	A	G	90	42,00%	het	38			+	FCAR	rs11084377			1,00E-012
reg_CTRL	chr19	60078732	A	G	88	42,00%	het	37			+	FCAR	rs8112766			1,00E-012
reg_CTRL	chr19	60081277	A	G	10	70,00%	het	7			+	FCAR	rs4806604			3,85E-010
reg_CTRL	chr19	60081476	T	C	16	50,00%	het	8			+	FCAR	rs4806605			8,62E-010
reg_CTRL	chr19	60082413	A	G	9	89,00%	homvar	8			+	FCAR	rs7257926			1,00E-012
reg_CTRL	chr19	60083518	A	T	18	50,00%	het	9			+	FCAR	rs6509905			7,33E-011
reg_CTRL	chr19	60084458	A	G	21	95,00%	homvar	20			+	FCAR	rs7259090			1,00E-012
reg_CTRL	chr19	60084567	C	T	16	100,00%	homvar	16			+	FCAR	rs7259347			1,00E-012
reg_CTRL	chr19	60084881	T	C	14	100,00%	homvar	14			+	FCAR	rs7248382	rs7247547		1,00E-012
reg_CTRL	chr19	60084977	A	G	12	100,00%	homvar	12			+	FCAR	rs12975418			1,00E-012
reg_CTRL	chr19	60085090	A	G	11	45,00%	het	5			+	FCAR	rs34764559			2,65E-006
reg_CTRL	chr19	60085431	A	G	41	100,00%	homvar	41			+	FCAR	rs4239591			1,00E-012
reg_CTRL	chr19	60085626	A	G	33	24,00%	ambig	8			+	FCAR	rs4806606			6,50E-007
reg_CTRL	chr19	60085864	G	A	20	30,00%	het	6			+	FCAR	rs10412499			4,35E-006
reg_CTRL	chr19	60086546	G	A	26	50,00%	het	13			+	FCAR	rs11671686			1,00E-012
reg_CTRL	chr19	60087161	C	G	33	55,00%	het	18			+	FCAR	rs28756208			1,00E-012
reg_CTRL	chr19	60087357	C	T	26	31,00%	het	8			+	FCAR	rs4806607			8,44E-008

reg_CTRL	chr19	60087394	A	G	26	27,00%	het	7			+	FCAR	rs4806608		1,52E-006
reg_CTRL	chr19	60087684	G	A	20	50,00%	het	10			+	FCAR	rs7260414		9,66E-012
reg_CTRL	chr19	60088124	G	A	8	100,00%	homvar	8			+	FCAR	rs10401687		1,00E-012
reg_CTRL	chr19	60088425	G	A	38	100,00%	homvar	38			+	FCAR	rs10402324		1,00E-012
reg_CTRL	chr19	60088712	A	G	71	99,00%	homvar	70	R	R	2	FCAR	rs1865096		1,00E-012
reg_CTRL	chr19	60088725	G	A	73	53,00%	het	39	D	N	2	FCAR	rs11666735		1,00E-012
reg_CTRL	chr19	60089029	A	G	37	100,00%	homvar	37			+	FCAR	rs1865097		1,00E-012
reg_CTRL	chr19	60089192	C	T	13	100,00%	homvar	13			+	FCAR	rs11666846		1,00E-012
reg_CTRL	chr19	60089829	T	C	14	71,00%	het	10			+	FCAR	rs12974530		1,00E-012
reg_CTRL	chr19	60089913	A	G	17	100,00%	homvar	17			+	FCAR	rs12972637		1,00E-012
reg_CTRL	chr19	60090047	T	C	27	100,00%	homvar	27			+	FCAR	rs12975083		1,00E-012
reg_CTRL	chr19	60090359	T	C	9	100,00%	homvar	9			+	FCAR		rs59223694	1,00E-012
reg_CTRL	chr19	60090376	C	T	9	100,00%	homvar	9			+	FCAR		rs59401716	1,00E-012
reg_CTRL	chr19	60090674	T	C	3	100,00%	homvar	3			+	FCAR	rs7258735		1,22E-005
reg_CTRL	chr19	60092982	A	G	72	58,00%	het	42	S	G	2	FCAR	rs16986050		1,00E-012
reg_CTRL	chr19	60093536	C	T	20	35,00%	het	7			+	FCAR	rs10413148		2,03E-007
reg_CTRL	chr19	60093544	T	G	20	35,00%	het	7			+	FCAR	rs10414707		2,03E-007
reg_CTRL	chr20	29527992	A	G	40	47,00%	het	19	H	R	1	REM1	rs1006459		1,00E-012
reg_CTRL	chr20	29530017	G	T	45	53,00%	het	24			+	REM1	rs717064		1,00E-012
reg_CTRL	chr20	29530975	C	T	86	42,00%	het	36			+	REM1	rs752841		1,00E-012
reg_CTRL	chr20	29530984	A	G	86	100,00%	homvar	86			+	REM1	rs215911		1,00E-012
reg_CTRL	chr20	29531413	T	C	23	13,00%	ambig	3			+	REM1			1,53E-002
reg_CTRL	chr20	29532051	T	C	49	47,00%	het	23			+	REM1	rs8120526		1,00E-012
reg_CTRL	chr20	29535505	C	T	62	52,00%	het	32			+	REM1	rs2233834		1,00E-012
reg_CTRL	chr20	29573824	T	C	30	10,00%	ambig	3			+	HM13			3,11E-002
reg_CTRL	chr20	29591769	T	G	75	97,00%	homvar	73			+	HM13	rs1555285		1,00E-012
reg_CTRL	chr20	29596516	T	A	58	71,00%	het	41			+	HM13			1,00E-012
reg_CTRL	chr20	29597668	T	C	8	37,00%	het	3			+	HM13			6,25E-004
reg_CTRL	chr20	29620213	C	T	85	15,00%	ambig	13			+	HM13			7,92E-008
reg_CTRL	chr8	6346527	C	T	9	78,00%	homvar	7			+	MCPH1	rs2916714		1,44E-010
reg_CTRL	chr8	6349118	A	G	29	90,00%	homvar	26			+	MCPH1	rs7841673		1,00E-012
reg_CTRL	chr8	6349757	A	G	29	10,00%	ambig	3			+	MCPH1			2,85E-002
reg_CTRL	chr8	6354204	C	T	85	98,00%	homvar	83			+	MCPH1			1,00E-012
reg_CTRL	chr8	6358884	C	T	77	95,00%	homvar	73			+	MCPH1	rs1982386		1,00E-012
reg_CTRL	chr8	6360207	G	A	70	90,00%	homvar	63			+	MCPH1	rs7816398		1,00E-012
reg_CTRL	chr8	6360907	T	C	72	100,00%	homvar	72			+	MCPH1	rs2009235		1,00E-012
reg_CTRL	chr8	6365605	G	A	65	98,00%	homvar	64			+	MCPH1	rs1960240		1,00E-012
reg_CTRL	chr8	6367073	G	A	30	97,00%	homvar	29			+	MCPH1	rs2515446		1,00E-012
reg_CTRL	chr8	6368638	C	G	11	100,00%	homvar	11			+	MCPH1	rs1989105		1,00E-012
reg_CTRL	chr8	6370028	C	T	67	97,00%	homvar	65			+	MCPH1	rs2959817		1,00E-012
reg_CTRL	chr8	6370280	A	G	23	91,00%	homvar	21			+	MCPH1	rs3020225		1,00E-012
reg_CTRL	chr8	6370714	A	T	61	89,00%	homvar	54			+	MCPH1	rs2922889		1,00E-012

reg_CTRL	chr8	6371007	A	G	64	92,00%	homvar	59			+	MCPH1	rs2515462	1,00E-012
reg_CTRL	chr8	6372214	C	T	57	96,00%	homvar	55			+	MCPH1	rs2959814	1,00E-012
reg_CTRL	chr8	6373087	T	A	13	100,00%	homvar	13			+	MCPH1	rs2442610	1,00E-012
reg_CTRL	chr8	6373247	T	G	42	98,00%	homvar	41			+	MCPH1	rs2515464	1,00E-012
reg_CTRL	chr8	6373270	G	C	44	100,00%	homvar	44			+	MCPH1	rs2515465	1,00E-012
reg_CTRL	chr8	6373612	C	T	97	97,00%	homvar	94			+	MCPH1	rs6990020	1,00E-012
reg_CTRL	chr8	6373694	A	G	90	97,00%	homvar	87			+	MCPH1	rs2515466	1,00E-012
reg_CTRL	chr8	6374188	C	G	89	98,00%	homvar	87			+	MCPH1	rs7825407	1,00E-012
reg_CTRL	chr8	6376528	G	T	33	100,00%	homvar	33			+	MCPH1	rs2515479	1,00E-012
reg_CTRL	chr8	6377297	C	G	69	99,00%	homvar	68	A	A	-1	ANGPT2	rs6559167	1,00E-012
reg_CTRL	chr8	6382432	G	A	43	58,00%	het	25			+	MCPH1		1,00E-012
reg_CTRL	chr8	6382971	T	A	14	43,00%	het	6			+	MCPH1	rs2959808	3,79E-007
reg_CTRL	chr8	6383317	G	A	75	45,00%	het	34			+	MCPH1	rs11989215	1,00E-012
reg_CTRL	chr8	6383428	G	A	78	50,00%	het	39			+	MCPH1	rs11989242	1,00E-012
reg_CTRL	chr8	6383749	C	T	67	49,00%	het	33			+	MCPH1	rs17623313	1,00E-012
reg_CTRL	chr8	6384278	G	A	53	41,00%	het	22			+	MCPH1	rs1375668	1,00E-012
reg_CTRL	chr8	6384313	G	A	49	39,00%	het	19			+	MCPH1	rs1989321	1,00E-012
reg_CTRL	chr8	6384394	G	T	49	43,00%	het	21			+	MCPH1	rs2897911	1,00E-012
reg_CTRL	chr8	6384406	G	C	48	37,00%	het	18			+	MCPH1	rs1823375	1,00E-012
reg_CTRL	chr8	6384513	C	A	24	25,00%	het	6			+	MCPH1	rs1823376	1,39E-005
reg_CTRL	chr8	6384555	T	G	20	25,00%	het	5			+	MCPH1	rs2408341	7,47E-005
reg_CTRL	chr8	6384627	C	T	19	53,00%	het	10			+	MCPH1	rs4263789	4,83E-012
reg_CTRL	chr8	6384716	A	T	15	40,00%	het	6			+	MCPH1	rs4376511	6,20E-007
reg_CTRL	chr8	6384838	A	T	44	41,00%	het	18			+	MCPH1	rs4455855	1,00E-012
reg_CTRL	chr8	6384861	C	T	48	44,00%	het	21			+	MCPH1	rs4991608	1,00E-012
reg_CTRL	chr8	6385735	A	G	76	54,00%	het	41			+	MCPH1	rs2922873	1,00E-012
reg_CTRL	chr8	6385973	T	C	46	52,00%	het	24			+	MCPH1	rs2922871	1,00E-012
reg_CTRL	chr8	6386346	G	T	43	49,00%	het	21			+	MCPH1	rs2959820	1,00E-012
reg_CTRL	chr8	6387110	A	G	17	29,00%	het	5			+	MCPH1		3,16E-005
reg_CTRL	chr8	6387274	T	G	28	43,00%	het	12			+	MCPH1	rs35742902	1,00E-012
reg_CTRL	chr8	6387413	G	A	46	46,00%	het	21			+	MCPH1	rs1988762	1,00E-012
reg_CTRL	chr8	6387489	C	A	48	48,00%	het	23			+	MCPH1	rs2515486	1,00E-012
reg_CTRL	chr8	6387856	C	A	52	63,00%	het	33			+	MCPH1	rs2515487	1,00E-012
reg_CTRL	chr8	6389049	C	T	87	99,00%	homvar	86			+	MCPH1	rs2442597	1,00E-012
reg_CTRL	chr8	6389775	G	A	115	50,00%	het	58			+	MCPH1	rs13250248	1,00E-012
reg_CTRL	chr8	6390400	A	G	61	100,00%	homvar	61			+	MCPH1	rs2442596	1,00E-012
reg_CTRL	chr8	6391248	T	C	67	100,00%	homvar	67			+	MCPH1	rs2044744	1,00E-012
reg_CTRL	chr8	6391663	G	C	81	99,00%	homvar	80			+	MCPH1	rs2515489	1,00E-012
reg_CTRL	chr8	6391722	A	G	81	99,00%	homvar	80			+	MCPH1	rs2442595	1,00E-012
reg_CTRL	chr8	6392028	A	T	67	100,00%	homvar	67			+	MCPH1	rs2442594	1,00E-012
reg_CTRL	chr8	6392149	T	C	57	98,00%	homvar	56			+	MCPH1	rs2442593	1,00E-012
reg_CTRL	chr8	6392271	T	C	56	82,00%	homvar	46			+	MCPH1	rs2515490	1,00E-012

reg_CTRL	chr8	6393016	C	T	71	100,00%	homvar	71			+	MCPH1	rs2442592		1,00E-012
reg_CTRL	chr8	6393121	G	A	83	100,00%	homvar	83			+	MCPH1	rs2515492		1,00E-012
reg_CTRL	chr8	6393980	C	A	120	98,00%	homvar	118			+	MCPH1	rs2515493		1,00E-012
reg_CTRL	chr8	6394533	T	C	53	100,00%	homvar	53			+	MCPH1	rs2515494		1,00E-012
reg_CTRL	chr8	6395624	A	T	58	84,00%	homvar	49			+	MCPH1	rs2442591		1,00E-012
reg_CTRL	chr8	6397787	C	T	61	52,00%	het	32			+	MCPH1	rs2515497		1,00E-012
reg_CTRL	chr8	6399828	G	C	49	100,00%	homvar	49			+	MCPH1	rs2515500		1,00E-012
reg_CTRL	chr8	6399852	G	C	50	70,00%	het	35			+	MCPH1	rs13262156		1,00E-012
reg_CTRL	chr8	6400140	G	A	67	100,00%	homvar	67			+	MCPH1	rs2515502		1,00E-012
reg_CTRL	chr8	6400377	A	G	24	96,00%	homvar	23			+	MCPH1	rs2922883		1,00E-012
reg_CTRL	chr8	6400446	T	C	25	96,00%	homvar	24			+	MCPH1	rs2515503		1,00E-012
reg_CTRL	chr8	6401094	A	T	50	12,00%	ambig	6			+	MCPH1			9,88E-004
reg_CTRL	chr8	6401634	C	T	40	90,00%	homvar	36			+	MCPH1	rs2515504		1,00E-012
reg_CTRL	chr8	6404255	A	G	38	100,00%	homvar	38			+	MCPH1	rs2515505		1,00E-012
reg_CTRL	chr8	6406668	T	G	52	92,00%	homvar	48			+	MCPH1	rs2515506		1,00E-012
reg_CTRL	chr8	6407942	A	G	86	99,00%	homvar	85			+	MCPH1	rs3739391		1,00E-012
reg_CTRL	chr8	6657680	A	G	110	49,00%	het	54			-	XKR5	rs12544341		1,00E-012
reg_CTRL	chr8	6657763	G	C	114	97,00%	homvar	111			-	XKR5	rs2615788		1,00E-012
reg_CTRL	chr8	6659973	G	T	52	62,00%	het	32			-	XKR5			1,00E-012
reg_CTRL	chr8	6660158	G	C	35	46,00%	het	16			-	XKR5			1,00E-012
reg_CTRL	chr8	6660662	A	G	33	58,00%	het	19			-	XKR5		rs58112583	1,00E-012
reg_CTRL	chr8	6660984	G	C	51	45,00%	het	23			-	XKR5	rs2738077		1,00E-012
reg_CTRL	chr8	6661868	A	G	48	54,00%	het	26			-	XKR5	rs9773025		1,00E-012
reg_CTRL	chr8	6669959	T	C	62	47,00%	het	29			-	XKR5			1,00E-012
reg_CTRL	chr8	6671547	C	T	97	54,00%	het	52			-	XKR5	rs2741083		1,00E-012
reg_CTRL	chr8	6672148	A	G	101	100,00%	homvar	101			-	XKR5	rs2980958		1,00E-012
reg_CTRL	chr8	6672229	T	G	106	100,00%	homvar	106			-	XKR5	rs2980957		1,00E-012
reg_CTRL	chr8	6672308	C	G	113	100,00%	homvar	113			-	XKR5	rs2980956		1,00E-012
reg_CTRL	chr8	6673095	C	A	55	49,00%	het	27			-	XKR5	rs2702924		1,00E-012
reg_CTRL	chr8	6673198	G	C	86	45,00%	het	39			-	XKR5	rs2741086		1,00E-012
reg_CTRL	chr8	6674201	C	T	55	35,00%	het	19			-	XKR5	rs2741087		1,00E-012
reg_CTRL	chr8	6675647	T	C	39	49,00%	het	19			-	XKR5	rs2741089		1,00E-012
reg_CTRL	chr8	6675775	G	C	43	51,00%	het	22			-	XKR5	rs2978903		1,00E-012
reg_CTRL	chr8	6676149	C	T	62	53,00%	het	33			-	XKR5			1,00E-012
reg_CTRL	chr8	6676673	T	C	69	96,00%	homvar	66			-	XKR5	rs2741091		1,00E-012
reg_CTRL	chr8	6676850	A	G	34	12,00%	ambig	4			-	XKR5			7,48E-003
reg_CTRL	chr8	6676904	A	G	31	16,00%	ambig	5			-	XKR5			6,64E-004
reg_CTRL	chr8	6677583	G	C	90	99,00%	homvar	89			-	XKR5	rs2978902		1,00E-012
reg_CTRL	chr8	6677625	G	C	83	99,00%	homvar	82			-	XKR5	rs2978901		1,00E-012
reg_CTRL	chr8	6677686	T	C	80	99,00%	homvar	79	M	V	-2	XKR5	rs2741098		1,00E-012
reg_CTRL	chr8	6678708	T	C	57	93,00%	homvar	53			-	XKR5	rs2978900		1,00E-012
reg_CTRL	chr8	6679008	C	G	64	98,00%	homvar	63			-	XKR5	rs2978899		1,00E-012

reg_CTRL	chr8	6679788	T	C	3	100,00%	homvar	3	-	XKR5	rs2978898		1,22E-005
reg_CTRL	chr8	6680072	G	C	3	100,00%	homvar	3	-	XKR5	rs2978897		1,22E-005
reg_DEFA	chr8	6714615	G	A	48	48,00%	het	23			rs2702829		1,00E-012
reg_DEFA	chr8	6715398	A	G	24	54,00%	het	13			rs2741123		1,00E-012
reg_DEFA	chr8	6715928	C	G	94	40,00%	het	38	-	DEFB1	rs2702885		1,00E-012
reg_DEFA	chr8	6715984	G	A	86	44,00%	het	38	-	DEFB1	rs2741124		1,00E-012
reg_DEFA	chr8	6716532	A	G	76	51,00%	het	39	-	DEFB1	rs2741125		1,00E-012
reg_DEFA	chr8	6716724	G	A	75	40,00%	het	30	-	DEFB1			1,00E-012
reg_DEFA	chr8	6717327	T	C	68	54,00%	het	37	-	DEFB1	rs2978874	rs5743482	1,00E-012
reg_DEFA	chr8	6717328	G	A	69	55,00%	het	38	-	DEFB1	rs5743482		1,00E-012
reg_DEFA	chr8	6717829	A	G	95	99,00%	homvar	94	-	DEFB1	rs2980928		1,00E-012
reg_DEFA	chr8	6717856	A	G	89	100,00%	homvar	89	-	DEFB1	rs2980927		1,00E-012
reg_DEFA	chr8	6717956	T	C	102	43,00%	het	44	-	DEFB1	rs2977779		1,00E-012
reg_DEFA	chr8	6717999	G	A	108	99,00%	homvar	107	-	DEFB1	rs2977778		1,00E-012
reg_DEFA	chr8	6718012	T	G	107	100,00%	homvar	107	-	DEFB1	rs2980926		1,00E-012
reg_DEFA	chr8	6718140	C	G	113	100,00%	homvar	113	-	DEFB1	rs2978872		1,00E-012
reg_DEFA	chr8	6718172	G	A	103	100,00%	homvar	103	-	DEFB1	rs2977777		1,00E-012
reg_DEFA	chr8	6718680	C	T	64	98,00%	homvar	63	-	DEFB1	rs2978870		1,00E-012
reg_DEFA	chr8	6718690	C	T	63	38,00%	het	24	-	DEFB1	rs2741126		1,00E-012
reg_DEFA	chr8	6718808	T	A	54	98,00%	homvar	53	-	DEFB1	rs2977776		1,00E-012
reg_DEFA	chr8	6718836	A	T	55	98,00%	homvar	54	-	DEFB1	rs2951854		1,00E-012
reg_DEFA	chr8	6718897	G	A	51	53,00%	het	27	-	DEFB1	rs2741127		1,00E-012
reg_DEFA	chr8	6718913	A	C	50	98,00%	homvar	49	-	DEFB1	rs2927345		1,00E-012
reg_DEFA	chr8	6718939	C	G	54	46,00%	het	25	-	DEFB1	rs5743467		1,00E-012
reg_DEFA	chr8	6719079	G	A	48	75,00%	het	36	-	DEFB1	rs11269568	rs9694202	1,00E-012
reg_DEFA	chr8	6719161	C	A	49	100,00%	homvar	49	-	DEFB1	rs2978869		1,00E-012
reg_DEFA	chr8	6719186	T	C	53	100,00%	homvar	53	-	DEFB1	rs2977774		1,00E-012
reg_DEFA	chr8	6719218	C	A	53	100,00%	homvar	53	-	DEFB1	rs2978868		1,00E-012
reg_DEFA	chr8	6719403	A	G	48	100,00%	homvar	48	-	DEFB1	rs2980924		1,00E-012
reg_DEFA	chr8	6719467	G	C	48	98,00%	homvar	47	-	DEFB1	rs5743465	rs34929240	1,00E-012
reg_DEFA	chr8	6719468	T	A	48	96,00%	homvar	46	-	DEFB1	rs34929240	rs5743464	1,00E-012
reg_DEFA	chr8	6719584	G	T	42	100,00%	homvar	42	-	DEFB1	rs5743463		1,00E-012
reg_DEFA	chr8	6719596	A	G	37	100,00%	homvar	37	-	DEFB1	rs5743462		1,00E-012
reg_DEFA	chr8	6719656	G	A	30	100,00%	homvar	30	-	DEFB1	rs2978867		1,00E-012
reg_DEFA	chr8	6719740	C	T	24	96,00%	homvar	23	-	DEFB1	rs2978866		1,00E-012
reg_DEFA	chr8	6720060	G	A	36	100,00%	homvar	36	-	DEFB1	rs2977773		1,00E-012
reg_DEFA	chr8	6720108	T	C	43	98,00%	homvar	42	-	DEFB1	rs2951855		1,00E-012
reg_DEFA	chr8	6720467	A	G	82	94,00%	homvar	77	-	DEFB1	rs2980923		1,00E-012
reg_DEFA	chr8	6720606	G	A	80	97,00%	homvar	78	-	DEFB1	rs2977772		1,00E-012
reg_DEFA	chr8	6720679	G	A	91	49,00%	het	45	-	DEFB1	rs2741129		1,00E-012
reg_DEFA	chr8	6721130	T	C	45	93,00%	homvar	42	-	DEFB1	rs2978864	rs5743441	1,00E-012
reg_DEFA	chr8	6721183	C	T	36	97,00%	homvar	35	-	DEFB1	rs5743440		1,00E-012

reg_DEFA	chr8	6721210	C	A	32	94,00%	homvar	30	-	DEFB1	rs5743439			1,00E-012
reg_DEFA	chr8	6721278	T	C	17	100,00%	homvar	17	-	DEFB1	rs5743437			1,00E-012
reg_DEFA	chr8	6721281	A	G	17	47,00%	het	8	-	DEFB1	rs2741130			1,60E-009
reg_DEFA	chr8	6721663	A	G	22	41,00%	het	9	-	DEFB1	rs2980922			6,96E-010
reg_DEFA	chr8	6721755	A	C	33	82,00%	homvar	27	-	DEFB1	rs2980921			1,00E-012
reg_DEFA	chr8	6722097	T	C	93	98,00%	homvar	91	-	DEFB1	rs2702945			1,00E-012
reg_DEFA	chr8	6722258	G	C	48	100,00%	homvar	48	-	DEFB1	rs2293960			1,00E-012
reg_DEFA	chr8	6722484	C	T	38	100,00%	homvar	38	-	DEFB1	rs2293959			1,00E-012
reg_DEFA	chr8	6722710	T	A	65	98,00%	homvar	64	-	DEFB1	rs2293958			1,00E-012
reg_DEFA	chr8	6722809	C	T	72	49,00%	het	35	-	DEFB1	rs11362			1,00E-012
reg_DEFA	chr8	6722833	C	G	74	100,00%	homvar	74	-	DEFB1	rs1800972			1,00E-012
reg_DEFA	chr8	6722841	C	T	71	49,00%	het	35	-	DEFB1	rs1799946			1,00E-012
reg_DEFA	chr8	6723179	A	T	65	48,00%	het	31			rs2738182			1,00E-012
reg_DEFA	chr8	6723399	C	T	52	44,00%	het	23			rs2741132			1,00E-012
reg_DEFA	chr8	6723423	G	C	46	46,00%	het	21			rs2702876	rs7838626		1,00E-012
reg_DEFA	chr8	6723465	G	T	42	48,00%	het	20			rs2741133			1,00E-012
reg_DEFA	chr8	6723477	G	C	43	98,00%	homvar	42			rs2702877			1,00E-012
reg_DEFA	chr8	6723483	T	C	46	100,00%	homvar	46			rs2977829			1,00E-012
reg_DEFA	chr8	6723520	G	A	49	43,00%	het	21			rs2741134			1,00E-012
reg_DEFA	chr8	6723531	C	T	50	44,00%	het	22			rs2978863			1,00E-012
reg_DEFA	chr8	6723664	G	A	57	46,00%	het	26			rs2741135			1,00E-012
reg_DEFA	chr8	6723906	A	T	43	42,00%	het	18			rs2738181			1,00E-012
reg_DEFA	chr8	6723908	C	T	42	40,00%	het	17			rs2738180			1,00E-012
reg_DEFA	chr8	6723927	T	G	39	36,00%	het	14			rs2738179			1,00E-012
reg_DEFA	chr8	6723930	A	C	39	36,00%	het	14			rs2738178			1,00E-012
reg_DEFA	chr8	6724030	C	A	25	48,00%	het	12					rs5743409	1,00E-012
reg_DEFA	chr8	6724531	G	A	48	52,00%	het	25			rs5743404			1,00E-012
reg_DEFA	chr8	6724606	A	G	48	48,00%	het	23			rs2741136			1,00E-012
reg_DEFA	chr8	6724645	A	G	46	48,00%	het	22			rs2741137			1,00E-012
reg_DEFA	chr8	6724992	A	G	15	40,00%	het	6			rs5743401			6,20E-007
reg_DEFA	chr8	6725055	G	A	12	33,00%	het	4			rs5743399			1,19E-004
reg_DEFA	chr8	6725193	C	T	14	36,00%	het	5			rs2472143			1,08E-005
reg_DEFA	chr8	6725224	G	A	15	33,00%	het	5			rs2977828			1,59E-005
reg_DEFA	chr8	6725291	G	C	31	48,00%	het	15			rs2702881			1,00E-012
reg_DEFA	chr8	6725524	T	C	27	33,00%	het	9			rs2738177			5,80E-009
reg_DEFA	chr8	6725600	G	T	25	60,00%	het	15			rs2741138			1,00E-012
reg_DEFA	chr8	6726157	A	G	7	43,00%	het	3			rs2741139			3,97E-004
reg_DEFA	chr8	6726180	A	G	7	43,00%	het	3			rs2741140			3,97E-004
reg_DEFA	chr8	6726302	T	C	14	29,00%	het	4			rs2702884			2,33E-004
reg_DEFA	chr8	6726851	T	C	89	43,00%	het	38			rs2738175			1,00E-012
reg_DEFA	chr8	6727011	G	A	71	52,00%	het	37			rs2741141			1,00E-012
reg_DEFA	chr8	6727167	C	T	61	52,00%	het	32			rs2977827			1,00E-012

reg_DEFA	chr8	6727332	G	A	97	49,00%	het	48	rs2978861			1,00E-012
reg_DEFA	chr8	6727393	T	C	100	53,00%	het	53	rs2978860			1,00E-012
reg_DEFA	chr8	6727484	C	T	98	53,00%	het	52	rs2977826			1,00E-012
reg_DEFA	chr8	6727601	C	T	94	50,00%	het	47	rs2977825			1,00E-012
reg_DEFA	chr8	6727790	G	A	38	42,00%	het	16			rs71214968	1,00E-012
reg_DEFA	chr8	6727818	G	A	33	55,00%	het	18	rs9692818	rs9694351		1,00E-012
reg_DEFA	chr8	6727819	T	C	34	71,00%	het	24	rs9694351			1,00E-012
reg_DEFA	chr8	6727833	A	C	36	50,00%	het	18	rs9694106			1,00E-012
reg_DEFA	chr8	6727846	A	G	39	74,00%	het	29	rs9694107			1,00E-012
reg_DEFA	chr8	6727902	A	G	59	66,00%	het	39	rs9694118			1,00E-012
reg_DEFA	chr8	6728005	G	T	69	65,00%	het	45	rs2978859			1,00E-012
reg_DEFA	chr8	6728154	C	T	66	61,00%	het	40	rs2977824			1,00E-012
reg_DEFA	chr8	6728378	A	G	54	48,00%	het	26	rs2741143			1,00E-012
reg_DEFA	chr8	6728397	C	T	57	49,00%	het	28	rs2738172			1,00E-012
reg_DEFA	chr8	6728537	T	C	50	44,00%	het	22	rs2738171			1,00E-012
reg_DEFA	chr8	6728715	T	G	38	39,00%	het	15	rs2978857			1,00E-012
reg_DEFA	chr8	6728719	A	G	38	39,00%	het	15	rs2951840			1,00E-012
reg_DEFA	chr8	6728764	C	T	32	37,00%	het	12	rs2980919			6,54E-012
reg_DEFA	chr8	6728770	T	C	31	42,00%	het	13	rs2978856			1,00E-012
reg_DEFA	chr8	6728867	G	C	17	35,00%	het	6	rs2978855			1,47E-006
reg_DEFA	chr8	6729141	A	G	14	50,00%	het	7	rs2977823			1,01E-008
reg_DEFA	chr8	6729311	T	C	37	49,00%	het	18	rs2978853			1,00E-012
reg_DEFA	chr8	6729458	C	T	51	45,00%	het	23	rs2977821			1,00E-012
reg_DEFA	chr8	6729520	C	T	57	47,00%	het	27	rs2951850			1,00E-012
reg_DEFA	chr8	6729801	G	A	100	49,00%	het	49	rs2978852			1,00E-012
reg_DEFA	chr8	6730195	A	T	51	51,00%	het	26				1,00E-012
reg_DEFA	chr8	6730978	A	G	36	100,00%	homvar	36	rs2738165			1,00E-012
reg_DEFA	chr8	6731383	T	C	40	42,00%	het	17	rs2738164			1,00E-012
reg_DEFA	chr8	6732145	G	T	34	50,00%	het	17			rs73517795	1,00E-012
reg_DEFA	chr8	6732419	C	T	69	42,00%	het	29				1,00E-012
reg_DEFA	chr8	6732626	C	T	96	56,00%	het	54	rs2738163			1,00E-012
reg_DEFA	chr8	6734051	A	G	117	48,00%	het	56	rs2741718			1,00E-012
reg_DEFA	chr8	6734149	G	A	108	100,00%	homvar	108	rs2978851			1,00E-012
reg_DEFA	chr8	6735296	G	A	73	56,00%	het	41	rs2741717			1,00E-012
reg_DEFA	chr8	6735747	A	C	64	55,00%	het	35	rs2738162			1,00E-012
reg_DEFA	chr8	6736007	C	T	57	39,00%	het	22	rs2738161			1,00E-012
reg_DEFA	chr8	6736585	C	T	80	54,00%	het	43	rs2738159			1,00E-012
reg_DEFA	chr8	6737079	G	A	62	100,00%	homvar	62	rs2738158			1,00E-012
reg_DEFA	chr8	6737661	T	C	68	56,00%	het	38	rs2738157			1,00E-012
reg_DEFA	chr8	6738298	A	G	29	100,00%	homvar	29	rs2978964			1,00E-012
reg_DEFA	chr8	6738591	G	A	51	100,00%	homvar	51	rs2951870			1,00E-012
reg_DEFA	chr8	6738844	T	C	75	55,00%	het	41	rs2702936			1,00E-012

reg_DEFA	chr8	6738879	C	T	77	44,00%	het	34	rs2738155		1,00E-012
reg_DEFA	chr8	6739237	A	G	105	50,00%	het	53	rs2702935		1,00E-012
reg_DEFA	chr8	6739310	T	G	96	36,00%	het	35	rs2738153		1,00E-012
reg_DEFA	chr8	6739495	T	A	111	46,00%	het	51	rs2738152		1,00E-012
reg_DEFA	chr8	6739788	T	C	78	55,00%	het	43	rs2741715		1,00E-012
reg_DEFA	chr8	6739842	C	T	79	57,00%	het	45	rs2738149		1,00E-012
reg_DEFA	chr8	6741958	C	A	79	99,00%	homvar	78	rs751009		1,00E-012
reg_DEFA	chr8	6742329	C	G	82	38,00%	het	31	rs2738148		1,00E-012
reg_DEFA	chr8	6742481	A	C	89	38,00%	het	34	rs2702911		1,00E-012
reg_DEFA	chr8	6743172	C	G	55	40,00%	het	22	rs2738145		1,00E-012
reg_DEFA	chr8	6743230	C	A	45	44,00%	het	20	rs2741056		1,00E-012
reg_DEFA	chr8	6743367	A	G	22	45,00%	het	10	rs2978963		3,38E-011
reg_DEFA	chr8	6743931	T	C	11	55,00%	het	6	rs2980959		6,19E-008
reg_DEFA	chr8	6744173	A	C	13	62,00%	het	8	rs9693852		9,13E-011
reg_DEFA	chr8	6744180	T	C	13	62,00%	het	8	rs13255719		9,13E-011
reg_DEFA	chr8	6744212	G	A	13	62,00%	het	8	rs2741057		9,13E-011
reg_DEFA	chr8	6745807	G	A	114	37,00%	het	42	rs2702929		1,00E-012
reg_DEFA	chr8	6745889	A	C	125	37,00%	het	46	rs2702930		1,00E-012
reg_DEFA	chr8	6746226	G	A	74	42,00%	het	31	rs2738143		1,00E-012
reg_DEFA	chr8	6746266	A	T	69	42,00%	het	29	rs2741060		1,00E-012
reg_DEFA	chr8	6746389	A	C	40	42,00%	het	17	rs2702931		1,00E-012
reg_DEFA	chr8	6746793	T	C	13	38,00%	het	5	rs2702932		7,10E-006
reg_DEFA	chr8	6746980	C	G	13	92,00%	homvar	12	rs2741061		1,00E-012
reg_DEFA	chr8	6747103	C	G	13	85,00%	homvar	11	rs2741062		1,00E-012
reg_DEFA	chr8	6747377	C	A	31	61,00%	het	19	rs2741714		1,00E-012
reg_DEFA	chr8	6747535	A	C	40	100,00%	homvar	40	rs2951867		1,00E-012
reg_DEFA	chr8	6747552	T	C	42	62,00%	het	26	rs2702933		1,00E-012
reg_DEFA	chr8	6747562	C	T	45	40,00%	het	18	rs2741064		1,00E-012
reg_DEFA	chr8	6747770	C	T	46	37,00%	het	17	rs2741065		1,00E-012
reg_DEFA	chr8	6747968	A	G	36	39,00%	het	14	rs2738142		1,00E-012
reg_DEFA	chr8	6748106	A	C	61	44,00%	het	27	rs2741713		1,00E-012
reg_DEFA	chr8	6748195	A	G	70	40,00%	het	28	rs2738141		1,00E-012
reg_DEFA	chr8	6748407	A	G	88	51,00%	het	45	rs2741068		1,00E-012
reg_DEFA	chr8	6748447	C	G	86	44,00%	het	38	rs2738140		1,00E-012
reg_DEFA	chr8	6748699	C	T	72	50,00%	het	36		rs55848359	1,00E-012
reg_DEFA	chr8	6748878	T	C	100	43,00%	het	43	rs20530		1,00E-012
reg_DEFA	chr8	6748942	A	G	122	46,00%	het	56	rs20528		1,00E-012
reg_DEFA	chr8	6748981	A	G	119	48,00%	het	57	rs20527		1,00E-012
reg_DEFA	chr8	6749191	G	A	102	49,00%	het	50	rs2741070		1,00E-012
reg_DEFA	chr8	6749195	A	C	101	51,00%	het	52	rs2702886		1,00E-012
reg_DEFA	chr8	6749262	T	G	88	41,00%	het	36	rs2738139		1,00E-012
reg_DEFA	chr8	6749839	A	C	29	48,00%	het	14	rs2741712		1,00E-012

reg_DEFA	chr8	6750400	G	C	42	50,00%	het	21	rs2741072		1,00E-012
reg_DEFA	chr8	6750581	T	C	78	99,00%	homvar	77	rs2981405		1,00E-012
reg_DEFA	chr8	6750651	A	G	92	53,00%	het	49	rs2741073		1,00E-012
reg_DEFA	chr8	6750694	A	G	94	46,00%	het	43	rs2738137		1,00E-012
reg_DEFA	chr8	6751071	C	T	69	45,00%	het	31	rs2741711		1,00E-012
reg_DEFA	chr8	6751139	A	T	62	53,00%	het	33	rs2702887		1,00E-012
reg_DEFA	chr8	6751207	C	T	58	98,00%	homvar	57	rs2741075		1,00E-012
reg_DEFA	chr8	6751396	T	C	52	100,00%	homvar	52	rs2741710		1,00E-012
reg_DEFA	chr8	6751654	C	T	22	55,00%	het	12	rs11782467		1,00E-012
reg_DEFA	chr8	6751657	C	G	20	55,00%	het	11	rs35621199		1,00E-012
reg_DEFA	chr8	6751684	T	C	19	63,00%	het	12	rs17078355		1,00E-012
reg_DEFA	chr8	6751724	G	A	12	67,00%	het	8	rs35809894		4,67E-011
reg_DEFA	chr8	6751748	T	A	12	67,00%	het	8			4,67E-011
reg_DEFA	chr8	6751750	G	A	11	64,00%	het	7			1,04E-009
reg_DEFA	chr8	6751808	T	C	11	55,00%	het	6	rs2980953		6,19E-008
reg_DEFA	chr8	6752098	G	A	11	45,00%	het	5	rs2741079		2,65E-006
reg_DEFA	chr8	6752323	C	T	25	48,00%	het	12	rs34412952		1,00E-012
reg_DEFA	chr8	6752361	T	C	36	44,00%	het	16	rs10085957		1,00E-012
reg_DEFA	chr8	6752442	G	C	51	45,00%	het	23	rs2702888		1,00E-012
reg_DEFA	chr8	6752546	A	G	83	42,00%	het	35	rs2702889		1,00E-012
reg_DEFA	chr8	6752550	A	G	84	43,00%	het	36	rs2741708		1,00E-012
reg_DEFA	chr8	6752578	C	T	91	43,00%	het	39	rs2702890		1,00E-012
reg_DEFA	chr8	6752677	C	T	109	43,00%	het	47		rs73194180	1,00E-012
reg_DEFA	chr8	6752728	A	G	107	40,00%	het	43	rs2702891		1,00E-012
reg_DEFA	chr8	6752872	G	A	108	45,00%	het	49	rs2702892		1,00E-012
reg_DEFA	chr8	6752904	A	G	109	47,00%	het	51	rs2702893		1,00E-012
reg_DEFA	chr8	6753127	A	G	69	38,00%	het	26	rs3888148		1,00E-012
reg_DEFA	chr8	6753189	A	C	52	48,00%	het	25	rs6605602		1,00E-012
reg_DEFA	chr8	6753230	G	T	46	39,00%	het	18	rs3888149		1,00E-012
reg_DEFA	chr8	6753242	C	G	44	41,00%	het	18	rs3888150		1,00E-012
reg_DEFA	chr8	6753261	C	G	46	39,00%	het	18	rs35516150		1,00E-012
reg_DEFA	chr8	6753275	T	C	49	39,00%	het	19	rs34494557		1,00E-012
reg_DEFA	chr8	6753286	C	T	49	59,00%	het	29			1,00E-012
reg_DEFA	chr8	6753328	C	G	50	32,00%	het	16	rs36092179		1,00E-012
reg_DEFA	chr8	6753376	A	G	54	35,00%	het	19	rs34166140		1,00E-012
reg_DEFA	chr8	6753385	C	G	55	33,00%	het	18	rs36021248		1,00E-012
reg_DEFA	chr8	6753589	G	C	64	44,00%	het	28	rs2951848		1,00E-012
reg_DEFA	chr8	6753656	C	T	63	38,00%	het	24	rs2981404		1,00E-012
reg_DEFA	chr8	6753661	C	G	67	42,00%	het	28	rs35504411		1,00E-012
reg_DEFA	chr8	6753664	C	G	73	42,00%	het	31	rs2981403		1,00E-012
reg_DEFA	chr8	6753731	G	A	82	44,00%	het	36	rs2951849		1,00E-012
reg_DEFA	chr8	6753760	C	T	79	47,00%	het	37	rs2981402		1,00E-012

reg_DEFA	chr8	6753858	A	G	61	36,00%	het	22				rs28533565		1,00E-012
reg_DEFA	chr8	6753882	T	C	57	33,00%	het	19				rs34987687		1,00E-012
reg_DEFA	chr8	6753915	G	A	47	26,00%	het	12				rs34087549		5,44E-010
reg_DEFA	chr8	6753932	C	T	41	24,00%	ambig	10				rs34661040		2,42E-008
reg_DEFA	chr8	6754046	A	C	25	24,00%	ambig	6					rs56174646	1,80E-005
reg_DEFA	chr8	6754602	C	T	85	48,00%	het	41				rs2702898		1,00E-012
reg_DEFA	chr8	6754803	G	A	89	53,00%	het	47				rs2702899		1,00E-012
reg_DEFA	chr8	6755659	A	G	81	48,00%	het	39				rs2702900		1,00E-012
reg_DEFA	chr8	6757796	A	G	55	53,00%	het	29				rs10216819		1,00E-012
reg_DEFA	chr8	6758791	T	C	53	100,00%	homvar	53				rs2981401		1,00E-012
reg_DEFA	chr8	6760323	T	G	62	53,00%	het	33				rs7461956		1,00E-012
reg_DEFA	chr8	6761824	G	A	102	45,00%	het	46				rs2738133		1,00E-012
reg_DEFA	chr8	6762080	C	T	61	52,00%	het	32				rs2741703		1,00E-012
reg_DEFA	chr8	6762169	C	T	45	64,00%	het	29				rs2741701		1,00E-012
reg_DEFA	chr8	6762964	G	A	65	100,00%	homvar	65				rs2741699		1,00E-012
reg_DEFA	chr8	6762978	C	A	64	94,00%	homvar	60				rs2741698		1,00E-012
reg_DEFA	chr8	6763134	A	G	43	56,00%	het	24				rs2741697		1,00E-012
reg_DEFA	chr8	6763299	T	A	58	45,00%	het	26				rs2741696		1,00E-012
reg_DEFA	chr8	6763722	G	A	59	100,00%	homvar	59				rs2741695		1,00E-012
reg_DEFA	chr8	6764461	A	G	29	100,00%	homvar	29				rs2741694		1,00E-012
reg_DEFA	chr8	6765176	G	A	63	48,00%	het	30				rs2738131		1,00E-012
reg_DEFA	chr8	6765363	G	A	58	98,00%	homvar	57				rs2702946		1,00E-012
reg_DEFA	chr8	6765676	A	G	53	57,00%	het	30				rs12545953		1,00E-012
reg_DEFA	chr8	6765994	T	C	30	100,00%	homvar	30				rs2738129		1,00E-012
reg_DEFA	chr8	6766076	A	C	43	98,00%	homvar	42				rs2738128		1,00E-012
reg_DEFA	chr8	6766524	T	C	66	100,00%	homvar	66				rs2978959		1,00E-012
reg_DEFA	chr8	6767164	C	T	58	48,00%	het	28				rs2738127		1,00E-012
reg_DEFA	chr8	6767542	G	A	91	54,00%	het	49				rs2738126		1,00E-012
reg_DEFA	chr8	6767654	T	C	69	48,00%	het	33				rs13275170		1,00E-012
reg_DEFA	chr8	6767778	G	T	39	100,00%	homvar	39				rs2738125		1,00E-012
reg_DEFA	chr8	6767823	A	C	31	29,00%	het	9				rs2738124		2,29E-008
reg_DEFA	chr8	6767996	T	C	14	50,00%	het	7				rs13276112		1,01E-008
reg_DEFA	chr8	6768647	T	A	46	50,00%	het	23				rs2702905		1,00E-012
reg_DEFA	chr8	6769030	T	C	51	41,00%	het	21				rs2738122		1,00E-012
reg_DEFA	chr8	6769096	T	C	40	25,00%	het	10				rs2738121		1,87E-008
reg_DEFA	chr8	6769681	A	G	97	49,00%	het	48		-	DEFA6	rs712276		1,00E-012
reg_DEFA	chr8	6770046	G	C	101	96,00%	homvar	97		-	DEFA6	rs2738120		1,00E-012
reg_DEFA	chr8	6770127	G	C	82	100,00%	homvar	82		-	DEFA6	rs2738119		1,00E-012
reg_DEFA	chr8	6770376	G	T	51	49,00%	het	25		-	DEFA6	rs2741691		1,00E-012
reg_DEFA	chr8	6771046	G	T	80	50,00%	het	40				rs11784359		1,00E-012
reg_DEFA	chr8	6771370	C	G	72	43,00%	het	31				rs4458901		1,00E-012
reg_DEFA	chr8	6771627	C	G	64	42,00%	het	27				rs2741690		1,00E-012

reg_DEFA	chr8	6771666	C	T	58	45,00%	het	26					rs2741689			1,00E-012
reg_DEFA	chr8	6772381	G	T	14	57,00%	het	8					rs2738118			2,09E-010
reg_DEFA	chr8	6773483	G	C	123	51,00%	het	63					rs2741686			1,00E-012
reg_DEFA	chr8	6774191	T	C	36	53,00%	het	19					rs3918350			1,00E-012
reg_DEFA	chr8	6774412	C	G	26	58,00%	het	15					rs34502430			1,00E-012
reg_DEFA	chr8	6774485	T	C	24	54,00%	het	13					rs2702938			1,00E-012
reg_DEFA	chr8	6774796	C	G	48	42,00%	het	20								1,00E-012
reg_DEFA	chr8	6774810	T	C	50	54,00%	het	27					rs2702939			1,00E-012
reg_DEFA	chr8	6775470	G	A	54	54,00%	het	29					rs2012832	rs2738114		1,00E-012
reg_DEFA	chr8	6775499	C	G	57	40,00%	het	23					rs2077406			1,00E-012
reg_DEFA	chr8	6775828	C	G	70	47,00%	het	33					rs2741684			1,00E-012
reg_DEFA	chr8	6775890	T	C	75	49,00%	het	37					rs2741683			1,00E-012
reg_DEFA	chr8	6776054	A	G	73	51,00%	het	37					rs2738111			1,00E-012
reg_DEFA	chr8	6776610	T	C	82	48,00%	het	39					rs2702855			1,00E-012
reg_DEFA	chr8	6776698	C	G	88	48,00%	het	42					rs3887306			1,00E-012
reg_DEFA	chr8	6777064	A	T	70	46,00%	het	32					rs2738109			1,00E-012
reg_DEFA	chr8	6777196	T	C	63	49,00%	het	31					rs2702858			1,00E-012
reg_DEFA	chr8	6777240	G	A	58	98,00%	homvar	57					rs2977818			1,00E-012
reg_DEFA	chr8	6777294	G	A	62	34,00%	het	21					rs13274891			1,00E-012
reg_DEFA	chr8	6777394	T	C	51	41,00%	het	21					rs13250769			1,00E-012
reg_DEFA	chr8	6777734	T	C	83	45,00%	het	37					rs2702861			1,00E-012
reg_DEFA	chr8	6777962	C	T	82	44,00%	het	36							rs56248548	1,00E-012
reg_DEFA	chr8	6778120	C	A	64	27,00%	het	17					rs13251447			1,00E-012
reg_DEFA	chr8	6778219	T	C	53	11,00%	ambig	6					rs2741682			1,35E-003
reg_DEFA	chr8	6778433	A	G	39	100,00%	homvar	39							rs59334047	1,00E-012
reg_DEFA	chr8	6779011	A	G	66	36,00%	het	24					rs13262140			1,00E-012
reg_DEFA	chr8	6779125	A	G	60	42,00%	het	25					rs2738106			1,00E-012
reg_DEFA	chr8	6779250	C	T	65	35,00%	het	23					rs13261705			1,00E-012
reg_DEFA	chr8	6779667	G	A	73	45,00%	het	33					rs2738104			1,00E-012
reg_DEFA	chr8	6779811	C	T	74	45,00%	het	33					rs13263461			1,00E-012
reg_DEFA	chr8	6779861	C	G	71	48,00%	het	34					rs13263510			1,00E-012
reg_DEFA	chr8	6779930	G	A	71	48,00%	het	34					rs2738103			1,00E-012
reg_DEFA	chr8	6780050	A	G	75	48,00%	het	36					rs13254588			1,00E-012
reg_DEFA	chr8	6780152	G	T	78	45,00%	het	35					rs13251814			1,00E-012
reg_DEFA	chr8	6780481	G	A	105	48,00%	het	50					rs2738102			1,00E-012
reg_DEFA	chr8	6780732	T	C	110	58,00%	het	64					rs2702867			1,00E-012
reg_DEFA	chr8	6780950	C	T	109	50,00%	het	55			-	DEFA4	rs736227			1,00E-012
reg_DEFA	chr8	6780991	A	G	105	46,00%	het	48	G	G	-2	DEFA4	rs2738100			1,00E-012
reg_DEFA	chr8	6781429	G	A	91	51,00%	het	46			-	DEFA4	rs2239668			1,00E-012
reg_DEFA	chr8	6781617	G	A	91	44,00%	het	40			-	DEFA4	rs2239667			1,00E-012
reg_DEFA	chr8	6782247	C	T	88	40,00%	het	35			-	DEFA4	rs4273874			1,00E-012
reg_DEFA	chr8	6782277	T	A	93	54,00%	het	50			-	DEFA4	rs2741679			1,00E-012

reg_DEFA	chr8	6783611	C	T	89	51,00%	het	45	rs2741676		1,00E-012
reg_DEFA	chr8	6784942	C	A	89	51,00%	het	45	rs2741675		1,00E-012
reg_DEFA	chr8	6785122	C	T	90	91,00%	homvar	82	rs2741674		1,00E-012
reg_DEFA	chr8	6786308	C	G	84	48,00%	het	40	rs2615772		1,00E-012
reg_DEFA	chr8	6786381	C	A	72	50,00%	het	36	rs2741673		1,00E-012
reg_DEFA	chr8	6786975	A	C	101	50,00%	het	51	rs3888293		1,00E-012
reg_DEFA	chr8	6787981	A	G	64	55,00%	het	35	rs2741672		1,00E-012
reg_DEFA	chr8	6788129	C	G	52	100,00%	homvar	52	rs2951853		1,00E-012
reg_DEFA	chr8	6788271	C	T	57	44,00%	het	25		rs56136595	1,00E-012
reg_DEFA	chr8	6789186	T	C	61	44,00%	het	27	rs41384045		1,00E-012
reg_DEFA	chr8	6789260	A	C	56	39,00%	het	22		rs61038944	1,00E-012
reg_DEFA	chr8	6789483	T	C	43	47,00%	het	20	rs2741669		1,00E-012
reg_DEFA	chr8	6790039	A	G	61	49,00%	het	30	rs12056400		1,00E-012
reg_DEFA	chr8	6790121	G	T	59	54,00%	het	32	rs12056552		1,00E-012
reg_DEFA	chr8	6790937	T	C	87	51,00%	het	44	rs2741668		1,00E-012
reg_DEFA	chr8	6791260	C	T	90	99,00%	homvar	89	rs2981396		1,00E-012
reg_DEFA	chr8	6792714	T	C	78	42,00%	het	33	rs2702878		1,00E-012
reg_DEFA	chr8	6792879	A	G	86	100,00%	homvar	86	rs2951844		1,00E-012
reg_DEFA	chr8	6793624	A	G	92	48,00%	het	44	rs2702879		1,00E-012
reg_DEFA	chr8	6793699	C	T	86	45,00%	het	39	rs2741665		1,00E-012
reg_DEFA	chr8	6793824	A	T	68	49,00%	het	33	rs2741663		1,00E-012
reg_DEFA	chr8	6794417	G	T	55	51,00%	het	28	rs2738088		1,00E-012
reg_DEFA	chr8	6794720	T	G	59	47,00%	het	28	rs2978955		1,00E-012
reg_DEFA	chr8	6795292	G	C	122	45,00%	het	55	rs2738086		1,00E-012
reg_DEFA	chr8	6795430	A	T	94	48,00%	het	45	rs2741661		1,00E-012
reg_DEFA	chr8	6796132	T	G	78	60,00%	het	47	rs2075836		1,00E-012
reg_DEFA	chr8	6797186	T	C	76	45,00%	het	34	rs2741660		1,00E-012
reg_DEFA	chr8	6797410	T	G	84	51,00%	het	43	rs2702907		1,00E-012
reg_DEFA	chr8	6797578	T	C	81	48,00%	het	39	rs2741659		1,00E-012
reg_DEFA	chr8	6798559	T	G	102	57,00%	het	58	rs2615787		1,00E-012
reg_DEFA	chr8	6798969	C	T	88	45,00%	het	40	rs2741658		1,00E-012
reg_DEFA	chr8	6799532	G	T	63	35,00%	het	22	rs2702850		1,00E-012
reg_DEFA	chr8	6799608	A	G	54	44,00%	het	24	rs2741657		1,00E-012
reg_DEFA	chr8	6799659	G	T	51	41,00%	het	21	rs2472562		1,00E-012
reg_DEFA	chr8	6799831	T	C	27	41,00%	het	11	rs2741653		1,60E-011
reg_DEFA	chr8	6800036	T	G	34	53,00%	het	18	rs2702851		1,00E-012
reg_DEFA	chr8	6800169	A	G	65	46,00%	het	30	rs2615771		1,00E-012
reg_DEFA	chr8	6800833	G	A	85	40,00%	het	34			1,00E-012
reg_DEFA	chr8	6800911	T	G	76	54,00%	het	41	rs2702852		1,00E-012
reg_DEFA	chr8	6802066	T	C	74	50,00%	het	37	rs2977812		1,00E-012
reg_DEFA	chr8	6802738	A	C	62	48,00%	het	30	rs2738082		1,00E-012
reg_DEFA	chr8	6802928	G	T	58	48,00%	het	28	rs2738081		1,00E-012

reg_DEFA	chr8	6803862	C	G	107	46,00%	het	49			rs10503361		1,00E-012
reg_DEFA	chr8	6804222	C	A	116	43,00%	het	50			rs2738079		1,00E-012
reg_DEFA	chr8	6804434	A	G	148	44,00%	het	65			rs2738078		1,00E-012
reg_DEFA	chr8	6805574	A	G	79	40,00%	het	32			rs2738071		1,00E-012
reg_DEFA	chr8	6805746	T	C	86	36,00%	het	31			rs2738069		1,00E-012
reg_DEFA	chr8	6805887	G	A	81	36,00%	het	29			rs2738068		1,00E-012
reg_DEFA	chr8	6806185	A	G	68	53,00%	het	36			rs2738067		1,00E-012
reg_DEFA	chr8	6806801	T	A	34	50,00%	het	17			rs2702853		1,00E-012
reg_DEFA	chr8	6807411	C	T	29	48,00%	het	14			rs2738066		1,00E-012
reg_DEFA	chr8	6807433	C	G	28	50,00%	het	14			rs6983753		1,00E-012
reg_DEFA	chr8	6807488	T	C	32	50,00%	het	16			rs6995959		1,00E-012
reg_DEFA	chr8	6807874	T	G	60	57,00%	het	34			rs12680018		1,00E-012
reg_DEFA	chr8	6808715	A	G	106	46,00%	het	49			rs4840647		1,00E-012
reg_DEFA	chr8	6808829	T	C	129	99,00%	homvar	128			rs2977793		1,00E-012
reg_DEFA	chr8	6809027	T	C	118	54,00%	het	64			rs2738058		1,00E-012
reg_DEFA	chr8	6809450	G	A	72	100,00%	homvar	72			rs2951869		1,00E-012
reg_DEFA	chr8	6809904	T	C	35	94,00%	homvar	33			rs2977789		1,00E-012
reg_DEFA	chr8	6809966	A	C	40	100,00%	homvar	40			rs2977788		1,00E-012
reg_DEFA	chr8	6810705	A	G	86	94,00%	homvar	81			rs2978951		1,00E-012
reg_DEFA	chr8	6810952	C	A	71	48,00%	het	34			rs2738046		1,00E-012
reg_DEFA	chr8	6810975	C	G	78	49,00%	het	38			rs2702912		1,00E-012
reg_DEFA	chr8	6810978	T	C	77	49,00%	het	38			rs2738045		1,00E-012
reg_DEFA	chr8	6811987	G	T	63	52,00%	het	33			rs2738168		1,00E-012
reg_DEFA	chr8	6812041	G	T	56	55,00%	het	31			rs13257112		1,00E-012
reg_DEFA	chr8	6812694	G	C	94	40,00%	het	38			rs2978950		1,00E-012
reg_DEFA	chr8	6812705	G	A	102	55,00%	het	56			rs6996047		1,00E-012
reg_DEFA	chr8	6812906	G	C	90	52,00%	het	47			rs11996346		1,00E-012
reg_DEFA	chr8	6812946	C	G	92	40,00%	het	37			rs4543566		1,00E-012
reg_DEFA	chr8	6813287	T	G	97	43,00%	het	42	-	DEFA10P	rs13278390		1,00E-012
reg_DEFA	chr8	6813295	A	G	93	41,00%	het	38	-	DEFA10P	rs13270884		1,00E-012
reg_DEFA	chr8	6813301	G	A	92	42,00%	het	39	-	DEFA10P	rs13267882		1,00E-012
reg_DEFA	chr8	6813385	T	G	74	38,00%	het	28	-	DEFA10P	rs13278672		1,00E-012
reg_DEFA	chr8	6813408	A	G	72	33,00%	het	24	-	DEFA10P	rs13271399		1,00E-012
reg_DEFA	chr8	6813420	C	G	72	29,00%	het	21	-	DEFA10P	rs13270339		1,00E-012
reg_DEFA	chr8	6813435	A	G	69	26,00%	het	18	-	DEFA10P	rs13271426		1,00E-012
reg_DEFA	chr8	6813460	C	T	65	20,00%	ambig	13	-	DEFA10P	rs13270374		2,71E-009
reg_DEFA	chr8	6813467	T	C	63	21,00%	ambig	13	-	DEFA10P	rs13278935		1,80E-009
reg_DEFA	chr8	6813491	A	G	54	17,00%	ambig	9	-	DEFA10P		rs71525777	3,75E-006
reg_DEFA	chr8	6813499	A	G	53	100,00%	homvar	53	-	DEFA10P	rs2978947		1,00E-012
reg_DEFA	chr8	6815354	T	C	38	13,00%	ambig	5					1,72E-003
reg_DEFA	chr8	6815365	C	G	40	17,00%	ambig	7					3,26E-005
reg_DEFA	chr8	6815387	C	T	44	18,00%	ambig	8					6,62E-006

reg_DEFA	chr8	6815411	G	A	43	16,00%	ambig	7						5,30E-005
reg_DEFA	chr8	6815430	A	C	46	22,00%	ambig	10						7,91E-008
reg_DEFA	chr8	6815437	C	A	47	23,00%	ambig	11						7,73E-009
reg_DEFA	chr8	6815442	G	C	48	23,00%	ambig	11						9,81E-009
reg_DEFA	chr8	6815446	T	G	48	23,00%	ambig	11						9,81E-009
reg_DEFA	chr8	6815845	G	A	72	35,00%	het	25			rs10088393			1,00E-012
reg_DEFA	chr8	6816495	G	T	35	57,00%	het	20			rs2738113			1,00E-012
reg_DEFA	chr8	6816665	C	A	36	47,00%	het	17			rs2738107			1,00E-012
reg_DEFA	chr8	6817036	T	C	106	17,00%	ambig	18			rs2738101			5,72E-011
reg_DEFA	chr8	6817114	C	T	136	15,00%	ambig	20			rs2615789			6,14E-011
reg_DEFA	chr8	6817149	T	A	135	13,00%	ambig	18			rs2738099			2,64E-009
reg_DEFA	chr8	6817203	C	T	118	11,00%	ambig	13			rs2702917			3,69E-006
reg_DEFA	chr8	6817334	A	G	106	16,00%	ambig	17			rs2738097			4,04E-010
reg_DEFA	chr8	6817394	C	G	101	16,00%	ambig	16			rs4841790			1,54E-009
reg_DEFA	chr8	6817412	C	T	95	15,00%	ambig	14			rs2978911			4,12E-008
reg_DEFA	chr8	6817429	A	G	86	13,00%	ambig	11			rs2738096			4,76E-006
reg_DEFA	chr8	6817438	G	A	82	13,00%	ambig	11			rs2978910			2,96E-006
reg_DEFA	chr8	6817564	T	C	57	23,00%	ambig	13			rs2738094			4,82E-010
reg_DEFA	chr8	6818357	G	A	43	44,00%	het	19			rs2738091			1,00E-012
reg_DEFA	chr8	6818361	C	T	43	44,00%	het	19			rs2738090	rs2447474		1,00E-012
reg_DEFA	chr8	6818374	C	T	36	50,00%	het	18			rs2702915	rs2738089		1,00E-012
reg_DEFA	chr8	6818375	G	A	36	47,00%	het	17			rs2738089			1,00E-012
reg_DEFA	chr8	6818397	C	T	25	48,00%	het	12			rs2927351			1,00E-012
reg_DEFA	chr8	6818535	T	A	69	38,00%	het	26			rs2615784	rs28647412		1,00E-012
reg_DEFA	chr8	6818566	A	C	87	53,00%	het	46					rs62488547	1,00E-012
reg_DEFA	chr8	6818614	A	G	103	38,00%	het	39			rs11305893			1,00E-012
reg_DEFA	chr8	6818706	G	C	125	50,00%	het	63			rs2702914			1,00E-012
reg_DEFA	chr8	6818828	C	A	112	25,00%	het	28						1,00E-012
reg_DEFA	chr8	6818877	C	A	101	43,00%	het	43			rs28694551	rs2927350		1,00E-012
reg_DEFA	chr8	6818898	A	G	97	44,00%	het	43			rs2927349			1,00E-012
reg_DEFA	chr8	6818919	C	T	92	42,00%	het	39			rs28693063			1,00E-012
reg_DEFA	chr8	6818941	A	G	89	43,00%	het	38			rs7460147			1,00E-012
reg_DEFA	chr8	6818949	G	A	90	42,00%	het	38						1,00E-012
reg_DEFA	chr8	6819003	C	T	79	58,00%	het	46			rs2977813			1,00E-012
reg_DEFA	chr8	6819005	A	G	78	44,00%	het	34			rs2978906			1,00E-012
reg_DEFA	chr8	6820626	C	A	180	50,00%	het	90					rs59243516	1,00E-012
reg_DEFA	chr8	6820983	G	A	96	93,00%	homvar	89			rs2615779			1,00E-012
reg_DEFA	chr8	6821065	G	A	50	22,00%	ambig	11						1,55E-008
reg_DEFA	chr8	6821169	T	C	9	89,00%	homvar	8			rs10108690			1,00E-012
reg_DEFA	chr8	6821178	A	G	9	89,00%	homvar	8			rs12381526			1,00E-012
reg_DEFA	chr8	6824570	C	A	22	95,00%	homvar	21	-	DEFA1B	rs2979395	rs3758132		1,00E-012
reg_DEFA	chr8	6824741	G	A	24	96,00%	homvar	23	-	DEFA1B			rs58955451	1,00E-012

reg_DEFA	chr8	6825349	C	T	108	32,00%	het	35					rs2978854		1,00E-012
reg_DEFA	chr8	6825357	G	A	107	33,00%	het	35					rs2951835		1,00E-012
reg_DEFA	chr8	6825678	A	G	32	97,00%	homvar	31					rs6993352		1,00E-012
reg_DEFA	chr8	6825754	C	T	17	88,00%	homvar	15					rs6986023		1,00E-012
reg_DEFA	chr8	6825821	C	T	7	71,00%	het	5							1,30E-007
reg_DEFA	chr8	6843886	T	A	206	11,00%	ambig	23			-	DEFA1B			6,44E-010
reg_DEFA	chr8	6844196	G	T	102	22,00%	ambig	22							1,00E-012
reg_DEFA	chr8	6844623	A	C	179	29,00%	het	52					rs28515027		1,00E-012
reg_DEFA	chr8	6844915	C	T	23	30,00%	het	7						rs71509223	6,03E-007
reg_DEFA	chr8	6846612	G	A	87	24,00%	ambig	21					rs4484718		1,00E-012
reg_DEFA	chr8	6846709	T	C	131	59,00%	het	77					rs3758131		1,00E-012
reg_DEFA	chr8	6859021	A	C	5	60,00%	het	3						rs71525791	1,18E-004
reg_DEFA	chr8	6859147	G	T	52	92,00%	homvar	48						rs2615778	1,00E-012
reg_DEFA	chr8	6859203	G	A	86	94,00%	homvar	81					rs28532282		1,00E-012
reg_DEFA	chr8	6859285	G	A	146	22,00%	ambig	32							1,00E-012
reg_DEFA	chr8	6859444	T	C	190	31,00%	het	59					rs2951846		1,00E-012
reg_DEFA	chr8	6859619	T	G	146	46,00%	het	67					rs2739221		1,00E-012
reg_DEFA	chr8	6859843	G	A	154	31,00%	het	48					rs2739220		1,00E-012
reg_DEFA	chr8	6859866	A	G	159	16,00%	ambig	25						rs4841796	1,00E-012
reg_DEFA	chr8	6860098	C	T	121	21,00%	ambig	25					rs10105775		1,00E-012
reg_DEFA	chr8	6860180	A	G	103	11,00%	ambig	11							2,72E-005
reg_DEFA	chr8	6860244	G	T	74	36,00%	het	27					rs10105163		1,00E-012
reg_DEFA	chr8	6860274	C	T	73	60,00%	het	44					rs9694309		1,00E-012
reg_DEFA	chr8	6861013	T	G	136	19,00%	ambig	26	D	A	-2	DEFA1	rs2230231		1,00E-012
reg_DEFA	chr8	6861144	T	G	180	16,00%	ambig	29			-	DEFA1B		rs4840655	1,00E-012
reg_DEFA	chr8	6861269	A	T	205	39,00%	het	80			-	DEFA1B	rs2702913		1,00E-012
reg_DEFA	chr8	6862522	A	G	15	100,00%	homvar	15			-	DEFA1B	rs4841798		1,00E-012
reg_DEFA	chr8	6863605	C	T	122	44,00%	het	54							1,00E-012
reg_DEFA	chr8	6864445	T	C	137	18,00%	ambig	25						rs62487509	1,00E-012
reg_DEFA	chr8	6864484	A	G	142	15,00%	ambig	21					rs4102677		2,43E-011
reg_DEFA	chr8	6864926	T	C	149	52,00%	het	77					rs2739218		1,00E-012
reg_DEFA	chr8	6864955	A	T	141	21,00%	ambig	30					rs9650669		1,00E-012
reg_DEFA	chr8	6864962	G	A	141	45,00%	het	63					rs2615770		1,00E-012
reg_DEFA	chr8	6865114	G	C	80	27,00%	het	22					rs4012963		1,00E-012
reg_DEFA	chr8	6865153	G	A	67	25,00%	het	17					rs4012962		1,00E-012
reg_DEFA	chr8	6865359	A	T	126	11,00%	ambig	14						rs7839709	1,43E-006
reg_DEFA	chr8	6865813	T	C	92	20,00%	ambig	18					rs11781199		1,25E-011
reg_DEFA	chr8	6865875	T	C	100	22,00%	ambig	22					rs11781205		1,00E-012
reg_DEFA	chr8	6865966	T	C	99	29,00%	het	29					rs11781229		1,00E-012
reg_DEFA	chr8	6866059	A	T	91	31,00%	het	28						rs56016462	1,00E-012
reg_DEFA	chr8	6866080	G	C	94	29,00%	het	27						rs55836016	1,00E-012
reg_DEFA	chr8	6866206	A	C	96	28,00%	het	27						rs55842276	1,00E-012

reg_DEFA	chr8	6866221	C	T	95	28,00%	het	27			1,00E-012
reg_DEFA	chr8	6866222	G	A	93	29,00%	het	27			1,00E-012
reg_DEFA	chr8	6866255	C	T	98	31,00%	het	30		rs73661330	1,00E-012
reg_DEFA	chr8	6866345	A	G	100	34,00%	het	34		rs59539636	1,00E-012
reg_DEFA	chr8	6866462	G	C	84	19,00%	ambig	16			9,04E-011
reg_DEFA	chr8	6866888	G	C	18	100,00%	homvar	18	rs35858635		1,00E-012
reg_DEFA	chr8	6866891	G	C	19	100,00%	homvar	19	rs34985860		1,00E-012
reg_DEFA	chr8	6866895	G	C	20	100,00%	homvar	20	rs35564068		1,00E-012
reg_DEFA	chr8	6866897	G	C	20	95,00%	homvar	19	rs35820601		1,00E-012
reg_DEFA	chr8	6866947	G	C	21	95,00%	homvar	20	rs4310228		1,00E-012
reg_DEFA	chr8	6869398	T	C	74	93,00%	homvar	69	rs4313182		1,00E-012
reg_DEFA	chr8	6869803	C	G	81	49,00%	het	40	rs883182		1,00E-012
reg_DEFA	chr8	6869887	A	T	98	99,00%	homvar	97	rs4481622		1,00E-012
reg_DEFA	chr8	6870176	T	G	97	99,00%	homvar	96	rs4314670		1,00E-012
reg_DEFA	chr8	6870220	G	T	97	99,00%	homvar	96	rs4332158		1,00E-012
reg_DEFA	chr8	6870259	G	A	89	97,00%	homvar	86	rs4332159		1,00E-012
reg_DEFA	chr8	6870678	C	A	90	43,00%	het	39	rs4448290		1,00E-012
reg_DEFA	chr8	6870686	G	C	90	97,00%	homvar	87	rs4469481		1,00E-012
reg_DEFA	chr8	6870701	A	G	89	99,00%	homvar	88	rs9774483		1,00E-012
reg_DEFA	chr8	6871265	A	G	65	100,00%	homvar	65	rs4840665		1,00E-012
reg_DEFA	chr8	6872288	T	C	83	40,00%	het	33	rs6984215		1,00E-012
reg_DEFA	chr8	6872534	A	C	66	100,00%	homvar	66	rs6605579		1,00E-012
reg_DEFA	chr8	6873321	T	A	112	46,00%	het	52	rs7820625		1,00E-012
reg_DEFA	chr8	6873505	A	G	133	50,00%	het	67	rs7816622		1,00E-012
reg_DEFA	chr8	6874265	A	T	65	100,00%	homvar	65	rs7821152		1,00E-012
reg_DEFA	chr8	6874382	G	A	52	44,00%	het	23	rs4403430		1,00E-012
reg_DEFA	chr8	6874473	T	G	64	100,00%	homvar	64	rs11137086		1,00E-012
reg_DEFA	chr8	6875301	C	T	42	52,00%	het	22		rs62487515	1,00E-012
reg_DEFA	chr8	6875366	G	A	40	95,00%	homvar	38	rs6982814		1,00E-012
reg_DEFA	chr8	6875538	C	A	68	46,00%	het	31		rs56230231	1,00E-012
reg_DEFA	chr8	6875544	T	C	69	51,00%	het	35		rs55851618	1,00E-012
reg_DEFA	chr8	6875673	C	G	73	52,00%	het	38		rs55660132	1,00E-012
reg_DEFA	chr8	6875975	A	G	60	55,00%	het	33		rs55740316	1,00E-012
reg_DEFA	chr8	6877006	A	T	42	95,00%	homvar	40	rs7011708		1,00E-012
reg_DEFA	chr8	6877045	G	A	44	57,00%	het	25	rs11776120		1,00E-012
reg_DEFA	chr8	6877291	A	G	43	56,00%	het	24	rs11786781		1,00E-012
reg_DEFA	chr8	6877358	G	C	41	100,00%	homvar	41	rs6993492		1,00E-012
reg_DEFA	chr8	6877487	T	C	42	64,00%	het	27	rs34825638		1,00E-012
reg_DEFA	chr8	6877608	G	A	48	100,00%	homvar	48	rs7824527		1,00E-012
reg_DEFA	chr8	6877988	G	T	46	98,00%	homvar	45	rs7825124		1,00E-012
reg_DEFA	chr8	6878312	T	C	22	100,00%	homvar	22	rs4490865		1,00E-012
reg_DEFA	chr8	6879022	T	G	78	96,00%	homvar	75	rs4841816		1,00E-012

reg_DEFA	chr8	6879381	C	G	109	50,00%	het	55	rs4433170		1,00E-012
reg_DEFA	chr8	6879503	C	G	73	49,00%	het	36	rs4300028		1,00E-012
reg_DEFA	chr8	6879828	A	G	47	98,00%	homvar	46	rs4602905		1,00E-012
reg_DEFA	chr8	6881033	C	T	19	100,00%	homvar	19	rs6605580		1,00E-012
reg_DEFA	chr8	6881147	G	A	16	75,00%	het	12	rs6605581		1,00E-012
reg_DEFA	chr8	6881163	G	A	17	94,00%	homvar	16	rs7014712		1,00E-012
reg_DEFA	chr8	6881365	G	T	49	98,00%	homvar	48	rs17078546		1,00E-012
reg_DEFA	chr8	6881460	G	A	55	96,00%	homvar	53	rs7015200		1,00E-012
reg_DEFA	chr8	6881573	G	A	77	56,00%	het	43	rs11775034		1,00E-012
reg_DEFA	chr8	6881816	T	C	98	99,00%	homvar	97	rs7004995		1,00E-012
reg_DEFA	chr8	6882227	G	T	36	92,00%	homvar	33	rs34333583		1,00E-012
reg_DEFA	chr8	6882354	T	A	20	90,00%	homvar	18	rs35708338		1,00E-012
reg_DEFA	chr8	6882359	G	T	18	100,00%	homvar	18	rs35866869		1,00E-012
reg_DEFA	chr8	6882374	T	C	14	100,00%	homvar	14	rs34268546		1,00E-012
reg_DEFA	chr8	6882401	G	C	13	100,00%	homvar	13	rs34599455		1,00E-012
reg_DEFA	chr8	6882406	T	A	13	38,00%	het	5		rs62488970	7,10E-006
reg_DEFA	chr8	6882583	C	G	17	47,00%	het	8		rs2515504	1,60E-009
reg_DEFA	chr8	6882620	T	C	18	56,00%	het	10	rs7009952		2,29E-012
reg_DEFA	chr8	6882669	G	T	16	69,00%	het	11	rs6982904		1,00E-012
reg_DEFA	chr8	6882937	G	C	43	98,00%	homvar	42	rs34219797		1,00E-012
reg_DEFA	chr8	6883103	C	T	32	94,00%	homvar	30	rs11994868		1,00E-012
reg_DEFA	chr8	6883131	T	C	32	97,00%	homvar	31	rs11985027		1,00E-012
reg_DEFA	chr8	6883151	T	G	29	97,00%	homvar	28	rs11985030		1,00E-012
reg_DEFA	chr8	6883232	T	C	24	100,00%	homvar	24	rs11985068		1,00E-012
reg_DEFA	chr8	6883286	T	C	26	100,00%	homvar	26	rs11985076		1,00E-012
reg_DEFA	chr8	6883469	A	C	52	94,00%	homvar	49	rs4841817		1,00E-012
reg_DEFA	chr8	6883493	G	T	56	96,00%	homvar	54	rs4840666		1,00E-012
reg_DEFA	chr8	6883556	G	C	73	96,00%	homvar	70	rs17078556		1,00E-012
reg_DEFA	chr8	6883571	T	G	77	97,00%	homvar	75	rs35196527		1,00E-012
reg_DEFA	chr8	6883577	A	G	79	97,00%	homvar	77	rs34725312		1,00E-012
reg_DEFA	chr8	6883643	C	T	82	98,00%	homvar	80	rs4621824		1,00E-012
reg_DEFA	chr8	6883724	A	G	93	98,00%	homvar	91	rs4345578		1,00E-012
reg_DEFA	chr8	6883754	T	C	95	98,00%	homvar	93	rs4342629		1,00E-012
reg_DEFA	chr8	6883823	G	C	98	100,00%	homvar	98	rs4342630		1,00E-012
reg_DEFA	chr8	6883831	G	C	99	100,00%	homvar	99	rs4342631		1,00E-012
reg_DEFA	chr8	6883942	C	T	86	98,00%	homvar	84	rs4549798		1,00E-012
reg_DEFA	chr8	6883982	A	C	84	98,00%	homvar	82	rs4342632		1,00E-012
reg_DEFA	chr8	6884098	A	T	91	52,00%	het	47	rs13256091		1,00E-012
reg_DEFA	chr8	6884242	G	T	75	97,00%	homvar	73	rs4601339		1,00E-012
reg_DEFA	chr8	6884293	G	C	72	53,00%	het	38	rs4367573		1,00E-012
reg_DEFA	chr8	6884304	G	T	69	99,00%	homvar	68	rs4601340		1,00E-012
reg_DEFA	chr8	6884370	C	A	62	100,00%	homvar	62	rs4642671		1,00E-012

reg_DEFA	chr8	6884762	G	C	66	45,00%	het	30	rs4840668		1,00E-012
reg_DEFA	chr8	6884922	G	A	69	45,00%	het	31	rs4240690		1,00E-012
reg_DEFA	chr8	6885295	C	T	48	40,00%	het	19	rs4418364		1,00E-012
reg_DEFA	chr8	6885310	C	T	47	96,00%	homvar	45	rs4546682		1,00E-012
reg_DEFA	chr8	6885444	G	A	44	59,00%	het	26	rs6981771		1,00E-012
reg_DEFA	chr8	6885525	C	T	65	52,00%	het	34	rs6996729		1,00E-012
reg_DEFA	chr8	6885553	C	T	66	98,00%	homvar	65	rs6996890		1,00E-012
reg_DEFA	chr8	6885601	C	A	65	48,00%	het	31	rs6996918		1,00E-012
reg_DEFA	chr8	6885642	A	G	70	46,00%	het	32	rs7004474		1,00E-012
reg_DEFA	chr8	6885716	T	A	77	45,00%	het	35	rs7009276		1,00E-012
reg_DEFA	chr8	6885754	C	T	77	47,00%	het	36	rs6997211		1,00E-012
reg_DEFA	chr8	6885796	T	G	72	96,00%	homvar	69	rs4841818		1,00E-012
reg_DEFA	chr8	6886141	A	G	95	96,00%	homvar	91	rs4358823		1,00E-012
reg_DEFA	chr8	6886408	T	C	85	47,00%	het	40	rs12716641		1,00E-012
reg_DEFA	chr8	6886563	G	A	70	50,00%	het	35	rs12716642		1,00E-012
reg_DEFA	chr8	6886619	G	A	58	52,00%	het	30	rs13274544		1,00E-012
reg_DEFA	chr8	6886896	C	A	43	40,00%	het	17	rs12716643		1,00E-012
reg_DEFA	chr8	6886899	C	T	42	38,00%	het	16	rs12716644		1,00E-012
reg_DEFA	chr8	6887255	A	C	54	94,00%	homvar	51	rs7843319		1,00E-012
reg_DEFA	chr8	6887285	C	G	57	96,00%	homvar	55	rs7836636		1,00E-012
reg_DEFA	chr8	6887373	C	T	64	92,00%	homvar	59	rs7836778		1,00E-012
reg_DEFA	chr8	6887405	A	C	70	93,00%	homvar	65	rs41514044	rs7843493	1,00E-012
reg_DEFA	chr8	6887848	C	A	86	93,00%	homvar	80	rs12674716		1,00E-012
reg_DEFA	chr8	6887922	G	T	75	87,00%	homvar	65	rs13250252		1,00E-012
reg_DEFA	chr8	6887979	C	G	71	96,00%	homvar	68	rs13252474		1,00E-012
reg_DEFA	chr8	6888029	T	A	65	94,00%	homvar	61	rs12716645		1,00E-012
reg_DEFA	chr8	6888052	G	A	62	95,00%	homvar	59	rs13257504		1,00E-012
reg_DEFA	chr8	6888345	T	G	28	96,00%	homvar	27	rs10108420		1,00E-012
reg_DEFA	chr8	6888430	G	A	24	96,00%	homvar	23	rs4841819		1,00E-012
reg_DEFA	chr8	6888510	C	T	24	92,00%	homvar	22	rs4841820		1,00E-012
reg_DEFA	chr8	6888714	G	C	45	93,00%	homvar	42	rs12716647		1,00E-012
reg_DEFA	chr8	6888737	C	G	46	96,00%	homvar	44	rs13261710		1,00E-012
reg_DEFA	chr8	6888783	C	A	60	97,00%	homvar	58	rs13261750		1,00E-012
reg_DEFA	chr8	6888786	A	G	61	97,00%	homvar	59	rs13262801		1,00E-012
reg_DEFA	chr8	6888796	G	A	60	90,00%	homvar	54	rs13259722		1,00E-012
reg_DEFA	chr8	6888800	T	G	63	90,00%	homvar	57	rs13270539		1,00E-012
reg_DEFA	chr8	6889029	C	G	78	99,00%	homvar	77	rs4304345		1,00E-012
reg_DEFA	chr8	6889141	C	T	71	97,00%	homvar	69	rs7017585		1,00E-012
reg_DEFA	chr8	6889250	C	G	69	94,00%	homvar	65	rs7017866		1,00E-012
reg_DEFA	chr8	6889297	T	C	63	97,00%	homvar	61	rs6992098		1,00E-012
reg_DEFA	chr8	6889488	T	G	70	94,00%	homvar	66	rs13279261		1,00E-012
reg_DEFA	chr8	6889853	A	T	61	93,00%	homvar	57	rs6988346		1,00E-012

reg_DEFA	chr8	6890032	C	G	63	90,00%	homvar	57	rs6981058		1,00E-012
reg_DEFA	chr8	6890057	T	A	63	95,00%	homvar	60	rs4841822		1,00E-012
reg_DEFA	chr8	6890122	T	C	65	94,00%	homvar	61	rs4841823		1,00E-012
reg_DEFA	chr8	6891017	C	A	60	98,00%	homvar	59	rs12682030		1,00E-012
reg_DEFA	chr8	6891039	G	T	66	91,00%	homvar	60	rs13269815		1,00E-012
reg_DEFA	chr8	6891182	A	G	69	91,00%	homvar	63	rs6998006		1,00E-012
reg_DEFA	chr8	6891217	C	G	63	92,00%	homvar	58	rs6990416		1,00E-012
reg_DEFA	chr8	6891225	T	G	61	93,00%	homvar	57	rs7002813		1,00E-012
reg_DEFA	chr8	6891463	G	T	78	96,00%	homvar	75	rs28480342		1,00E-012
reg_DEFA	chr8	6891605	A	C	89	97,00%	homvar	86	rs6998687		1,00E-012
reg_DEFA	chr8	6891649	G	A	80	96,00%	homvar	77	rs7014280		1,00E-012
reg_DEFA	chr8	6891711	C	A	78	94,00%	homvar	73	rs6991235		1,00E-012
reg_DEFA	chr8	6891919	A	G	60	97,00%	homvar	58	rs6999181		1,00E-012
reg_DEFA	chr8	6892865	T	C	34	88,00%	homvar	30	rs13257750		1,00E-012
reg_DEFA	chr8	6892895	C	G	34	88,00%	homvar	30	rs13249237		1,00E-012
reg_DEFA	chr8	6893048	T	C	22	95,00%	homvar	21	rs13265227		1,00E-012
reg_DEFA	chr8	6893329	G	A	16	94,00%	homvar	15	rs13255432		1,00E-012
reg_DEFA	chr8	6893366	A	G	20	100,00%	homvar	20	rs13258671		1,00E-012
reg_DEFA	chr8	6893449	C	T	16	100,00%	homvar	16	rs12675019	rs11137087	1,00E-012
reg_DEFA	chr8	6893450	A	G	16	100,00%	homvar	16	rs11137087		1,00E-012
reg_DEFA	chr8	6893571	A	T	17	94,00%	homvar	16	rs12677144		1,00E-012
reg_DEFA	chr8	6894199	A	G	13	92,00%	homvar	12	rs4841824		1,00E-012
reg_DEFA	chr8	6894251	G	A	17	100,00%	homvar	17	rs4841825		1,00E-012
reg_DEFA	chr8	6894335	C	T	23	96,00%	homvar	22	rs13267464		1,00E-012
reg_DEFA	chr8	6894359	A	G	27	100,00%	homvar	27	rs4841827		1,00E-012
reg_DEFA	chr8	6894414	T	C	28	93,00%	homvar	26	rs4841829		1,00E-012
reg_DEFA	chr8	6894855	C	A	21	100,00%	homvar	21	rs10867024		1,00E-012
reg_DEFA	chr8	6895055	T	G	30	100,00%	homvar	30	rs10780177		1,00E-012
reg_DEFA	chr8	6895465	T	G	52	94,00%	homvar	49	rs10503360		1,00E-012
reg_DEFA	chr8	6895593	G	A	48	96,00%	homvar	46	rs7007253		1,00E-012
reg_DEFA	chr8	6896339	C	G	16	100,00%	homvar	16	rs4446760		1,00E-012
reg_DEFA	chr8	6896851	C	T	69	93,00%	homvar	64	rs10867025		1,00E-012
reg_DEFA	chr8	6897044	G	C	74	93,00%	homvar	69	rs11137088		1,00E-012
reg_DEFA	chr8	6897177	C	G	76	95,00%	homvar	72	rs4451329		1,00E-012
reg_DEFA	chr8	6897235	C	T	72	94,00%	homvar	68	rs4344104		1,00E-012
reg_DEFA	chr8	6897291	T	C	75	95,00%	homvar	71	rs4392921		1,00E-012
reg_DEFA	chr8	6897555	T	G	76	96,00%	homvar	73	rs13271389		1,00E-012
reg_DEFA	chr8	6898076	G	A	84	94,00%	homvar	79	rs4841830		1,00E-012
reg_DEFA	chr8	6898613	T	G	72	99,00%	homvar	71	rs12680521		1,00E-012
reg_DEFA	chr8	6898728	C	T	56	98,00%	homvar	55	rs13273327		1,00E-012
reg_DEFA	chr8	6898818	A	G	34	100,00%	homvar	34	rs12680095		1,00E-012
reg_DEFA	chr8	6898847	C	T	30	97,00%	homvar	29	rs12678005		1,00E-012

reg_DEFA		chr8	6899381	G	A	53	98,00%	homvar	52					rs13279849		1,00E-012
reg_DEFA		chr8	6899982	G	A	54	96,00%	homvar	52					rs4645580		1,00E-012
reg_DEFA		chr8	6900441	G	A	61	93,00%	homvar	57	T	T	-2	DEFA5	rs2272719		1,00E-012
reg_DEFA		chr8	6901260	A	G	61	98,00%	homvar	60			-	DEFA5	rs10095331		1,00E-012
reg_DEFA		chr8	6902056	T	C	52	92,00%	homvar	48					rs4395911		1,00E-012
reg_DEFB	YES	chr8	7157719	T	C	38	13,00%	ambig	5							1,72E-003
reg_DEFB	YES	chr8	7157850	C	G	31	23,00%	ambig	7			+	DEFB109P1B			5,51E-006
reg_DEFB	YES	chr8	7157878	T	G	32	19,00%	ambig	6			+	DEFB109P1B	rs35717569		8,02E-005
reg_DEFB	YES	chr8	7157902	A	T	31	19,00%	ambig	6			+	DEFB109P1B		rs4089933	6,64E-005
reg_DEFB	YES	chr8	7158003	C	G	24	17,00%	ambig	4			+	DEFB109P1B			2,06E-003
reg_DEFB	YES	chr8	7158094	A	G	21	24,00%	ambig	5			+	DEFB109P1B			9,62E-005
reg_DEFB	YES	chr8	7158098	G	A	20	20,00%	ambig	4			+	DEFB109P1B			1,01E-003
reg_DEFB	YES	chr8	7158123	C	T	19	21,00%	ambig	4			+	DEFB109P1B			8,22E-004
reg_DEFB	YES	chr8	7158749	A	G	31	13,00%	ambig	4			+	DEFB109P1B			5,36E-003
reg_DEFB	YES	chr8	7158793	A	G	39	18,00%	ambig	7			+	DEFB109P1B			2,74E-005
reg_DEFB	YES	chr8	7158853	A	G	38	24,00%	ambig	9			+	DEFB109P1B			1,60E-007
reg_DEFB	YES	chr8	7158885	G	C	41	20,00%	ambig	8			+	DEFB109P1B			3,80E-006
reg_DEFB	YES	chr8	7159019	T	G	51	16,00%	ambig	8			+	DEFB109P1B	rs4403433		2,06E-005
reg_DEFB	YES	chr8	7159295	C	T	75	11,00%	ambig	8			+	DEFB109P1B	rs2979549		3,35E-004
reg_DEFB	YES	chr8	7159297	A	G	75	11,00%	ambig	8			+	DEFB109P1B	rs7008951		3,35E-004
reg_DEFB	YES	chr8	7159374	T	A	71	11,00%	ambig	8			+	DEFB109P1B	rs7013858		2,29E-004
reg_DEFB	YES	chr8	7159448	T	A	62	16,00%	ambig	10			+	DEFB109P1B	rs7013998		1,49E-006
reg_DEFB	YES	chr8	7159450	C	G	62	11,00%	ambig	7			+	DEFB109P1B			5,53E-004
reg_DEFB	YES	chr8	7159461	C	T	62	16,00%	ambig	10			+	DEFB109P1B	rs7001891		1,49E-006
reg_DEFB	YES	chr8	7159601	C	T	65	14,00%	ambig	9			+	DEFB109P1B			1,80E-005
reg_DEFB	YES	chr8	7159605	T	G	65	31,00%	het	20			+	DEFB109P1B			1,00E-012
reg_DEFB	YES	chr8	7159615	G	A	67	28,00%	het	19			+	DEFB109P1B			1,00E-012
reg_DEFB	YES	chr8	7159663	C	T	73	31,00%	het	23			+	DEFB109P1B			1,00E-012
reg_DEFB	YES	chr8	7159700	C	G	73	21,00%	ambig	15			+	DEFB109P1B			1,11E-010
reg_DEFB	YES	chr8	7159772	C	T	67	22,00%	ambig	15			+	DEFB109P1B			3,38E-011
reg_DEFB	YES	chr8	7159834	T	A	65	18,00%	ambig	12			+	DEFB109P1B	rs28509470		2,84E-008
reg_DEFB	YES	chr8	7159850	A	T	59	15,00%	ambig	9			+	DEFB109P1B			8,00E-006
reg_DEFB	YES	chr8	7159922	T	G	59	10,00%	ambig	6			+	DEFB109P1B			2,35E-003
reg_DEFB	YES	chr8	7161809	G	A	11	36,00%	het	4			+	DEFB109P1B		rs6468887	8,11E-005
reg_DEFB	YES	chr8	7161813	T	C	12	42,00%	het	5			+	DEFB109P1B			4,45E-006
reg_DEFB	YES	chr8	7161816	A	G	14	57,00%	het	8			+	DEFB109P1B			2,09E-010
reg_DEFB	YES	chr8	7161822	C	T	14	50,00%	het	7			+	DEFB109P1B			1,01E-008
reg_DEFB	YES	chr8	7161838	G	A	16	44,00%	het	7			+	DEFB109P1B		rs6470342	3,25E-008
reg_DEFB	YES	chr8	7161843	A	G	17	65,00%	het	11			+	DEFB109P1B			1,00E-012
reg_DEFB	YES	chr8	7161862	C	G	18	44,00%	het	8			+	DEFB109P1B		rs71526779	2,79E-009
reg_DEFB	YES	chr8	7161864	T	C	19	63,00%	het	12			+	DEFB109P1B		rs35703273	1,00E-012
reg_DEFB	YES	chr8	7161901	A	G	25	12,00%	ambig	3			+	DEFB109P1B			1,92E-002

reg_DEFB	YES	chr8	7161903	A	T	25	48,00%	het	12	+	DEFB109P1B			1,00E-012
reg_DEFB	YES	chr8	7161905	C	T	26	46,00%	het	12	+	DEFB109P1B			1,00E-012
reg_DEFB	YES	chr8	7161942	T	G	30	40,00%	het	12	+	DEFB109P1B			2,51E-012
reg_DEFB	YES	chr8	7161986	C	T	31	23,00%	ambig	7	+	DEFB109P1B			5,51E-006
reg_DEFB	YES	chr8	7162017	G	A	32	28,00%	het	9	+	DEFB109P1B			3,13E-008
reg_DEFB	YES	chr8	7162060	T	G	35	40,00%	het	14	+	DEFB109P1B			1,00E-012
reg_DEFB	YES	chr8	7162065	T	G	38	37,00%	het	14	+	DEFB109P1B			1,00E-012
reg_DEFB	YES	chr8	7162126	A	G	43	23,00%	ambig	10	+	DEFB109P1B			3,96E-008
reg_DEFB	YES	chr8	7162141	T	C	46	11,00%	ambig	5	+	DEFB109P1B			4,03E-003
reg_DEFB	YES	chr8	7162150	T	C	47	26,00%	het	12	+	DEFB109P1B			5,44E-010
reg_DEFB	YES	chr8	7162179	C	G	52	29,00%	het	15	+	DEFB109P1B			1,00E-012
reg_DEFB	YES	chr8	7162212	G	A	57	11,00%	ambig	6	+	DEFB109P1B			1,97E-003
reg_DEFB	YES	chr8	7162263	T	C	60	35,00%	het	21	+	DEFB109P1B			1,00E-012
reg_DEFB	YES	chr8	7162291	A	C	64	31,00%	het	20	+	DEFB109P1B			1,00E-012
reg_DEFB	YES	chr8	7162321	A	G	71	14,00%	ambig	10	+	DEFB109P1B			5,31E-006
reg_DEFB	YES	chr8	7162338	T	C	72	32,00%	het	23	+	DEFB109P1B			1,00E-012
reg_DEFB	YES	chr8	7162365	G	A	71	30,00%	het	21	+	DEFB109P1B			1,00E-012
reg_DEFB	YES	chr8	7162381	G	A	69	13,00%	ambig	9	+	DEFB109P1B			2,93E-005
reg_DEFB	YES	chr8	7162396	G	A	69	13,00%	ambig	9	+	DEFB109P1B			2,93E-005
reg_DEFB	YES	chr8	7162410	A	G	70	30,00%	het	21	+	DEFB109P1B			1,00E-012
reg_DEFB	YES	chr8	7162421	T	C	66	18,00%	ambig	12	+	DEFB109P1B			3,40E-008
reg_DEFB	YES	chr8	7162442	G	C	67	30,00%	het	20	+	DEFB109P1B			1,00E-012
reg_DEFB	YES	chr8	7162550	A	C	58	21,00%	ambig	12	+	DEFB109P1B			7,31E-009
reg_DEFB	YES	chr8	7162599	G	T	55	13,00%	ambig	7	+	DEFB109P1B			2,62E-004
reg_DEFB	YES	chr8	7162607	G	A	57	14,00%	ambig	8	+	DEFB109P1B			4,73E-005
reg_DEFB	YES	chr8	7162865	C	G	43	23,00%	ambig	10	+	DEFB109P1B	rs34406851		3,96E-008
reg_DEFB	YES	chr8	7162927	C	T	51	12,00%	ambig	6	+	DEFB109P1B			1,10E-003
reg_DEFB	YES	chr8	7162966	C	G	53	38,00%	het	20	+	DEFB109P1B			1,00E-012
reg_DEFB	YES	chr8	7163030	A	T	65	14,00%	ambig	9	+	DEFB109P1B			1,80E-005
reg_DEFB	YES	chr8	7163075	G	A	70	57,00%	het	40	+	DEFB109P1B			1,00E-012
reg_DEFB	YES	chr8	7163106	C	T	75	37,00%	het	28	+	DEFB109P1B			1,00E-012
reg_DEFB	YES	chr8	7163115	G	C	78	37,00%	het	29	+	DEFB109P1B			1,00E-012
reg_DEFB	YES	chr8	7163135	A	T	77	26,00%	het	20	+	DEFB109P1B			1,00E-012
reg_DEFB	YES	chr8	7163183	G	A	72	26,00%	het	19	+	DEFB109P1B			1,00E-012
reg_DEFB	YES	chr8	7163236	C	T	60	27,00%	het	16	+	DEFB109P1B			1,00E-012
reg_DEFB	YES	chr8	7163242	C	T	59	56,00%	het	33	+	DEFB109P1B			1,00E-012
reg_DEFB	YES	chr8	7163246	C	A	60	57,00%	het	34	+	DEFB109P1B			1,00E-012
reg_DEFB	YES	chr8	7163250	A	G	60	27,00%	het	16	+	DEFB109P1B			1,00E-012
reg_DEFB	YES	chr8	7163295	G	A	55	56,00%	het	31	+	DEFB109P1B			1,00E-012
reg_DEFB	YES	chr8	7163341	G	C	54	54,00%	het	29	+	DEFB109P1B			1,00E-012
reg_DEFB	YES	chr8	7163419	G	T	49	43,00%	het	21	+	DEFB109P1B		rs3988782	1,00E-012
reg_DEFB	YES	chr8	7163423	C	T	49	31,00%	het	15	+	DEFB109P1B		rs3988781	1,00E-012

reg_DEFB	YES	chr8	7163430	T	C	49	49,00%	het	24	+	DEFB109P1B		1,00E-012
reg_DEFB	YES	chr8	7163432	T	C	48	17,00%	ambig	8	+	DEFB109P1B		1,30E-005
reg_DEFB	YES	chr8	7163508	C	A	31	13,00%	ambig	4	+	DEFB109P1B		5,36E-003
reg_DEFB	YES	chr8	7163520	A	G	29	14,00%	ambig	4	+	DEFB109P1B		4,20E-003
reg_DEFB	YES	chr8	7163524	A	G	29	21,00%	ambig	6	+	DEFB109P1B	rs3890542	4,46E-005
reg_DEFB	YES	chr8	7164249	A	T	28	11,00%	ambig	3	+	DEFB109P1B		2,60E-002
reg_DEFB	YES	chr8	7164562	G	T	42	14,00%	ambig	6	+	DEFB109P1B	rs2719441	3,81E-004
reg_DEFB	YES	chr8	7164584	C	T	42	12,00%	ambig	5	+	DEFB109P1B		2,70E-003
reg_DEFB	YES	chr8	7164586	T	C	41	12,00%	ambig	5	+	DEFB109P1B		2,42E-003
reg_DEFB	YES	chr8	7164674	T	G	47	11,00%	ambig	5	+	DEFB109P1B		4,42E-003
reg_DEFB	YES	chr8	7164874	C	G	50	22,00%	ambig	11	+	DEFB109P1B		1,55E-008
reg_DEFB	YES	chr8	7164890	A	G	45	20,00%	ambig	9				7,54E-007
reg_DEFB	YES	chr8	7164975	C	T	55	42,00%	het	23			rs11786798	1,00E-012
reg_DEFB	YES	chr8	7165027	C	T	60	52,00%	het	31				1,00E-012
reg_DEFB	YES	chr8	7165051	A	G	63	43,00%	het	27				1,00E-012
reg_DEFB	YES	chr8	7165061	T	G	66	52,00%	het	34				1,00E-012
reg_DEFB	YES	chr8	7165101	T	C	67	18,00%	ambig	12			rs2740006	4,06E-008
reg_DEFB	YES	chr8	7165115	G	A	69	43,00%	het	30				1,00E-012
reg_DEFB	YES	chr8	7165121	T	C	70	11,00%	ambig	8				2,08E-004
reg_DEFB	YES	chr8	7165164	T	A	74	50,00%	het	37				1,00E-012
reg_DEFB	YES	chr8	7165167	G	C	74	12,00%	ambig	9				5,16E-005
reg_DEFB	YES	chr8	7165176	G	A	71	44,00%	het	31				1,00E-012
reg_DEFB	YES	chr8	7165207	A	T	65	52,00%	het	34				1,00E-012
reg_DEFB	YES	chr8	7165348	G	A	41	22,00%	ambig	9				3,24E-007
reg_DEFB	YES	chr8	7165400	C	A	37	16,00%	ambig	6				1,86E-004
reg_DEFB	YES	chr8	7165697	C	A	26	12,00%	ambig	3				2,13E-002
reg_DEFB	YES	chr8	7165708	C	T	25	16,00%	ambig	4				2,40E-003
reg_DEFB	YES	chr8	7165766	C	A	23	22,00%	ambig	5			rs3988809	1,53E-004
reg_DEFB	YES	chr8	7165798	C	T	21	29,00%	het	6				5,96E-006
reg_DEFB	YES	chr8	7165812	G	A	19	26,00%	het	5				5,71E-005
reg_DEFB	YES	chr8	7165831	T	G	20	25,00%	het	5			rs3988808	7,47E-005
reg_DEFB	YES	chr8	7167310	A	T	21	24,00%	ambig	5				9,62E-005
reg_DEFB	YES	chr8	7167370	T	G	16	37,00%	het	6			rs7835547	9,72E-007
reg_DEFB	YES	chr8	7167475	T	C	34	50,00%	het	17				1,00E-012
reg_DEFB	YES	chr8	7167493	A	G	38	61,00%	het	23				1,00E-012
reg_DEFB	YES	chr8	7167515	A	C	40	62,00%	het	25				1,00E-012
reg_DEFB	YES	chr8	7167647	A	G	44	64,00%	het	28			rs55915041	1,00E-012
reg_DEFB	YES	chr8	7167705	C	T	46	61,00%	het	28			rs62492162	1,00E-012
reg_DEFB	YES	chr8	7167774	T	A	54	54,00%	het	29				1,00E-012
reg_DEFB	YES	chr8	7167808	G	T	58	47,00%	het	27				1,00E-012
reg_DEFB	YES	chr8	7167817	T	C	59	54,00%	het	32				1,00E-012
reg_DEFB	YES	chr8	7167844	A	T	71	39,00%	het	28				1,00E-012

reg_DEFB	YES	chr8	7167864	A	G	68	46,00%	het	31				1,00E-012
reg_DEFB	YES	chr8	7167894	T	C	75	39,00%	het	29				1,00E-012
reg_DEFB	YES	chr8	7167922	A	C	77	30,00%	het	23				1,00E-012
reg_DEFB	YES	chr8	7167957	G	C	80	32,00%	het	26				1,00E-012
reg_DEFB	YES	chr8	7167983	G	A	82	34,00%	het	28				1,00E-012
reg_DEFB	YES	chr8	7167987	T	C	81	33,00%	het	27				1,00E-012
reg_DEFB	YES	chr8	7168028	C	T	88	25,00%	het	22				1,00E-012
reg_DEFB	YES	chr8	7168044	C	G	89	33,00%	het	29				1,00E-012
reg_DEFB	YES	chr8	7168073	T	G	87	25,00%	het	22				1,00E-012
reg_DEFB	YES	chr8	7168111	T	C	80	10,00%	ambig	8				5,21E-004
reg_DEFB	YES	chr8	7168178	T	C	75	32,00%	het	24				1,00E-012
reg_DEFB	YES	chr8	7168187	G	A	67	25,00%	het	17				1,00E-012
reg_DEFB	YES	chr8	7168243	T	G	61	21,00%	ambig	13				1,19E-009
reg_DEFB	YES	chr8	7168264	C	T	57	18,00%	ambig	10				6,65E-007
reg_DEFB	YES	chr8	7168267	T	A	56	12,00%	ambig	7				2,94E-004
reg_DEFB	YES	chr8	7168355	A	G	49	16,00%	ambig	8				1,52E-005
reg_DEFB	YES	chr8	7168356	T	G	51	14,00%	ambig	7				1,62E-004
reg_DEFB	YES	chr8	7168457	T	C	74	28,00%	het	21				1,00E-012
reg_DEFB	YES	chr8	7168499	G	A	74	32,00%	het	24				1,00E-012
reg_DEFB	YES	chr8	7168558	C	G	82	18,00%	ambig	15				6,32E-010
reg_DEFB	YES	chr8	7168569	C	T	84	19,00%	ambig	16				9,04E-011
reg_DEFB	YES	chr8	7168632	A	G	86	50,00%	het	43				1,00E-012
reg_DEFB	YES	chr8	7168694	T	C	85	27,00%	het	23				1,00E-012
reg_DEFB	YES	chr8	7168697	T	C	83	54,00%	het	45				1,00E-012
reg_DEFB	YES	chr8	7168738	C	T	81	52,00%	het	42				1,00E-012
reg_DEFB	YES	chr8	7168755	G	A	79	56,00%	het	44				1,00E-012
reg_DEFB	YES	chr8	7168766	C	A	78	56,00%	het	44			rs11786479	1,00E-012
reg_DEFB	YES	chr8	7168779	T	C	77	22,00%	ambig	17				5,67E-012
reg_DEFB	YES	chr8	7168791	A	C	76	62,00%	het	47				1,00E-012
reg_DEFB	YES	chr8	7168807	T	C	75	43,00%	het	32			rs11776779	1,00E-012
reg_DEFB	YES	chr8	7168824	A	G	75	28,00%	het	21				1,00E-012
reg_DEFB	YES	chr8	7168834	C	T	70	41,00%	het	29			rs11774389	1,00E-012
reg_DEFB	YES	chr8	7168916	T	C	67	12,00%	ambig	8				1,52E-004
reg_DEFB	YES	chr8	7168926	T	C	65	60,00%	het	39	rs3178039	rs34791945		1,00E-012
reg_DEFB	YES	chr8	7169004	T	A	59	24,00%	ambig	14				6,50E-011
reg_DEFB	YES	chr8	7169013	C	A	59	24,00%	ambig	14				6,50E-011
reg_DEFB	YES	chr8	7169050	T	G	48	31,00%	het	15			rs71509292	1,00E-012
reg_DEFB	YES	chr8	7169077	C	A	45	27,00%	het	12			rs10089913	3,12E-010
reg_DEFB	YES	chr8	7169868	G	A	72	14,00%	ambig	10				6,04E-006
reg_DEFB	YES	chr8	7169921	T	A	88	27,00%	het	24	rs3907269			1,00E-012
reg_DEFB	YES	chr8	7169973	C	T	106	13,00%	ambig	14			rs4109371	1,68E-007
reg_DEFB	YES	chr8	7169980	C	T	106	26,00%	het	28				1,00E-012

reg_DEFB	YES	chr8	7169982	C	T	107	27,00%	het	29			1,00E-012
reg_DEFB	YES	chr8	7169999	C	T	105	29,00%	het	30			1,00E-012
reg_DEFB	YES	chr8	7170000	G	A	109	16,00%	ambig	17			6,17E-010
reg_DEFB	YES	chr8	7170022	T	C	112	48,00%	het	54		rs4096457	1,00E-012
reg_DEFB	YES	chr8	7170056	C	G	119	52,00%	het	62	rs34518748		1,00E-012
reg_DEFB	YES	chr8	7170064	C	T	133	26,00%	het	35		rs4096458	1,00E-012
reg_DEFB	YES	chr8	7170105	G	A	146	27,00%	het	39	rs3907268		1,00E-012
reg_DEFB	YES	chr8	7170112	C	A	144	31,00%	het	45		rs62488805	1,00E-012
reg_DEFB	YES	chr8	7170135	A	G	152	18,00%	ambig	27		rs2739990	1,00E-012
reg_DEFB	YES	chr8	7170203	C	G	158	10,00%	ambig	16			9,18E-007
reg_DEFB	YES	chr8	7170213	T	C	163	26,00%	het	42			1,00E-012
reg_DEFB	YES	chr8	7170224	T	G	164	10,00%	ambig	16			1,51E-006
reg_DEFB	YES	chr8	7170282	G	A	146	81,00%	homvar	118	rs34361581		1,00E-012
reg_DEFB	YES	chr8	7170290	C	T	150	57,00%	het	86			1,00E-012
reg_DEFB	YES	chr8	7170304	C	T	152	23,00%	ambig	35			1,00E-012
reg_DEFB	YES	chr8	7170312	C	T	151	33,00%	het	50			1,00E-012
reg_DEFB	YES	chr8	7170317	C	T	152	21,00%	ambig	32			1,00E-012
reg_DEFB	YES	chr8	7170331	G	T	147	56,00%	het	82			1,00E-012
reg_DEFB	YES	chr8	7170340	G	A	142	20,00%	ambig	28			1,00E-012
reg_DEFB	YES	chr8	7170351	G	T	143	58,00%	het	83			1,00E-012
reg_DEFB	YES	chr8	7170384	T	A	143	34,00%	het	49			1,00E-012
reg_DEFB	YES	chr8	7170400	C	T	146	10,00%	ambig	15			1,66E-006
reg_DEFB	YES	chr8	7170412	G	A	148	21,00%	ambig	31			1,00E-012
reg_DEFB	YES	chr8	7170443	G	C	145	35,00%	het	51			1,00E-012
reg_DEFB	YES	chr8	7170446	A	C	145	11,00%	ambig	16		rs2739989	2,86E-007
reg_DEFB	YES	chr8	7170453	T	C	142	36,00%	het	51			1,00E-012
reg_DEFB	YES	chr8	7170458	C	A	143	20,00%	ambig	29			1,00E-012
reg_DEFB	YES	chr8	7170468	T	A	143	56,00%	het	80			1,00E-012
reg_DEFB	YES	chr8	7170492	T	A	135	20,00%	ambig	27			1,00E-012
reg_DEFB	YES	chr8	7170510	T	C	127	10,00%	ambig	13			8,33E-006
reg_DEFB	YES	chr8	7170518	A	T	122	17,00%	ambig	21			3,23E-012
reg_DEFB	YES	chr8	7170538	G	A	114	20,00%	ambig	23			1,00E-012
reg_DEFB	YES	chr8	7170546	T	A	113	14,00%	ambig	16			8,21E-009
reg_DEFB	YES	chr8	7170547	A	G	111	37,00%	het	41			1,00E-012
reg_DEFB	YES	chr8	7170569	T	A	104	37,00%	het	38			1,00E-012
reg_DEFB	YES	chr8	7170573	A	G	101	52,00%	het	53		rs71505181	1,00E-012
reg_DEFB	YES	chr8	7170645	T	C	74	46,00%	het	34			1,00E-012
reg_DEFB	YES	chr8	7170771	T	C	28	29,00%	het	8			1,61E-007
reg_DEFB	YES	chr8	7173577	A	C	27	22,00%	ambig	6			2,89E-005
reg_DEFB	YES	chr8	7173587	T	G	27	22,00%	ambig	6	rs34181137		2,89E-005
reg_DEFB	YES	chr8	7173717	C	T	44	34,00%	het	15			1,00E-012
reg_DEFB	YES	chr8	7173721	A	G	43	37,00%	het	16			1,00E-012

reg_DEFB	YES	chr8	7173725	C	T	44	36,00%	het	16			1,00E-012
reg_DEFB	YES	chr8	7173741	A	G	43	40,00%	het	17			1,00E-012
reg_DEFB	YES	chr8	7173771	T	G	45	42,00%	het	19			1,00E-012
reg_DEFB	YES	chr8	7173875	T	G	56	36,00%	het	20			1,00E-012
reg_DEFB	YES	chr8	7173894	T	C	56	37,00%	het	21		rs3976517	1,00E-012
reg_DEFB	YES	chr8	7173897	T	G	57	37,00%	het	21			1,00E-012
reg_DEFB	YES	chr8	7173926	A	G	55	38,00%	het	21		rs2864406	1,00E-012
reg_DEFB	YES	chr8	7173931	G	C	54	35,00%	het	19			1,00E-012
reg_DEFB	YES	chr8	7173970	G	T	54	35,00%	het	19			1,00E-012
reg_DEFB	YES	chr8	7173976	G	T	56	32,00%	het	18			1,00E-012
reg_DEFB	YES	chr8	7173991	A	G	55	24,00%	ambig	13			2,98E-010
reg_DEFB	YES	chr8	7174003	T	C	56	29,00%	het	16			1,00E-012
reg_DEFB	YES	chr8	7174006	T	C	56	29,00%	het	16		rs2864408	1,00E-012
reg_DEFB	YES	chr8	7174013	C	T	53	30,00%	het	16			1,00E-012
reg_DEFB	YES	chr8	7174047	C	T	51	98,00%	homvar	50		rs62488807	1,00E-012
reg_DEFB	YES	chr8	7174052	C	T	52	27,00%	het	14			2,32E-011
reg_DEFB	YES	chr8	7174097	C	T	60	22,00%	ambig	13			9,56E-010
reg_DEFB	YES	chr8	7174161	A	T	65	15,00%	ambig	10			2,33E-006
reg_DEFB	YES	chr8	7174164	T	C	64	14,00%	ambig	9			1,58E-005
reg_DEFB	YES	chr8	7174316	G	T	52	12,00%	ambig	6			1,22E-003
reg_DEFB	YES	chr8	7174321	A	G	52	12,00%	ambig	6			1,22E-003
reg_DEFB	YES	chr8	7174324	C	G	51	12,00%	ambig	6			1,10E-003
reg_DEFB	YES	chr8	7174355	T	A	49	16,00%	ambig	8		rs66483251	1,52E-005
reg_DEFB	YES	chr8	7174356	C	G	49	10,00%	ambig	5			5,30E-003
reg_DEFB	YES	chr8	7174375	A	C	50	18,00%	ambig	9		rs13260764	1,92E-006
reg_DEFB	YES	chr8	7174387	T	A	51	20,00%	ambig	10			2,23E-007
reg_DEFB	YES	chr8	7174394	A	C	49	20,00%	ambig	10			1,50E-007
reg_DEFB	YES	chr8	7174405	A	T	49	14,00%	ambig	7			1,25E-004
reg_DEFB	YES	chr8	7174432	T	C	48	21,00%	ambig	10			1,22E-007
reg_DEFB	YES	chr8	7174433	A	C	48	23,00%	ambig	11			9,81E-009
reg_DEFB	YES	chr8	7174459	A	T	51	24,00%	ambig	12			1,52E-009
reg_DEFB	YES	chr8	7174469	T	C	52	27,00%	het	14			2,32E-011
reg_DEFB	YES	chr8	7174509	G	A	54	30,00%	het	16			1,00E-012
reg_DEFB	YES	chr8	7174523	A	T	58	29,00%	het	17			1,00E-012
reg_DEFB	YES	chr8	7174577	C	A	53	21,00%	ambig	11			2,98E-008
reg_DEFB	YES	chr8	7174608	T	C	56	36,00%	het	20			1,00E-012
reg_DEFB	YES	chr8	7174659	A	T	48	37,00%	het	18			1,00E-012
reg_DEFB	YES	chr8	7174725	A	G	47	30,00%	het	14			4,96E-012
reg_DEFB	YES	chr8	7174734	G	A	45	27,00%	het	12			3,12E-010
reg_DEFB	YES	chr8	7174748	C	A	42	31,00%	het	13			1,74E-011
reg_DEFB	YES	chr8	7174758	G	A	41	29,00%	het	12			1,00E-010
reg_DEFB	YES	chr8	7174776	C	T	40	15,00%	ambig	6		rs7839403	2,90E-004

reg_DEFB	YES	chr8	7174795	G	A	39	31,00%	het	12			5,13E-011
reg_DEFB	YES	chr8	7174861	A	C	26	27,00%	het	7			1,52E-006
reg_DEFB	YES	chr8	7174867	G	T	27	22,00%	ambig	6			2,89E-005
reg_DEFB	YES	chr8	7174881	A	T	27	19,00%	ambig	5			3,40E-004
reg_DEFB	YES	chr8	7176301	T	C	16	19,00%	ambig	3			5,44E-003
reg_DEFB	YES	chr8	7176317	T	G	18	22,00%	ambig	4			6,61E-004
reg_DEFB	YES	chr8	7176337	A	G	20	20,00%	ambig	4			1,01E-003
reg_DEFB	YES	chr8	7176356	C	T	20	20,00%	ambig	4			1,01E-003
reg_DEFB	YES	chr8	7176372	A	G	24	17,00%	ambig	4			2,06E-003
reg_DEFB	YES	chr8	7176381	A	G	24	17,00%	ambig	4			2,06E-003
reg_DEFB	YES	chr8	7176384	A	G	24	17,00%	ambig	4			2,06E-003
reg_DEFB	YES	chr8	7176390	G	T	25	16,00%	ambig	4			2,40E-003
reg_DEFB	YES	chr8	7176395	A	G	25	16,00%	ambig	4			2,40E-003
reg_DEFB	YES	chr8	7176412	C	A	24	17,00%	ambig	4			2,06E-003
reg_DEFB	YES	chr8	7176428	A	T	24	17,00%	ambig	4			2,06E-003
reg_DEFB	YES	chr8	7176450	T	G	26	15,00%	ambig	4			2,79E-003
reg_DEFB	YES	chr8	7176472	T	G	27	15,00%	ambig	4			3,22E-003
reg_DEFB	YES	chr8	7176495	A	C	27	15,00%	ambig	4			3,22E-003
reg_DEFB	YES	chr8	7176523	A	G	26	15,00%	ambig	4			2,79E-003
reg_DEFB	YES	chr8	7176544	A	C	27	15,00%	ambig	4			3,22E-003
reg_DEFB	YES	chr8	7176556	C	T	27	11,00%	ambig	3			2,36E-002
reg_DEFB	YES	chr8	7177210	A	C	12	25,00%	het	3			2,29E-003
reg_DEFB	YES	chr8	7177266	G	T	9	33,00%	het	3			9,21E-004
reg_DEFB	YES	chr8	7177310	G	C	8	37,00%	het	3			6,25E-004
reg_DEFB	YES	chr8	7178422	T	C	17	24,00%	ambig	4			5,24E-004
reg_DEFB	YES	chr8	7178500	C	T	21	52,00%	het	11			1,00E-012
reg_DEFB	YES	chr8	7178506	T	A	21	52,00%	het	11			1,00E-012
reg_DEFB	YES	chr8	7178529	A	C	28	64,00%	het	18		rs9694780	1,00E-012
reg_DEFB	YES	chr8	7178569	A	C	36	53,00%	het	19			1,00E-012
reg_DEFB	YES	chr8	7178638	G	T	43	72,00%	het	31	rs34108245		1,00E-012
reg_DEFB	YES	chr8	7178675	G	A	45	67,00%	het	30			1,00E-012
reg_DEFB	YES	chr8	7178702	A	G	47	74,00%	het	35			1,00E-012
reg_DEFB	YES	chr8	7178706	C	T	45	80,00%	homvar	36			1,00E-012
reg_DEFB	YES	chr8	7178726	A	T	47	11,00%	ambig	5			4,42E-003
reg_DEFB	YES	chr8	7178746	A	G	52	73,00%	het	38			1,00E-012
reg_DEFB	YES	chr8	7178799	G	C	53	62,00%	het	33		rs9694759	1,00E-012
reg_DEFB	YES	chr8	7178804	G	C	54	72,00%	het	39			1,00E-012
reg_DEFB	YES	chr8	7178839	T	C	47	68,00%	het	32			1,00E-012
reg_DEFB	YES	chr8	7178844	A	G	45	67,00%	het	30			1,00E-012
reg_DEFB	YES	chr8	7178852	A	G	38	63,00%	het	24			1,00E-012
reg_DEFB	YES	chr8	7178861	G	T	38	68,00%	het	26			1,00E-012
reg_DEFB	YES	chr8	7178925	G	A	34	47,00%	het	16	rs34767109		1,00E-012

reg_DEFB	YES	chr8	7178949	G	A	32	37,00%	het	12			6,54E-012
reg_DEFB	YES	chr8	7178957	A	G	36	31,00%	het	11			3,56E-010
reg_DEFB	YES	chr8	7179104	T	C	20	15,00%	ambig	3	rs35175639		1,03E-002
reg_DEFB	YES	chr8	7179121	A	G	21	24,00%	ambig	5			9,62E-005
reg_DEFB	YES	chr8	7179124	C	T	21	19,00%	ambig	4			1,22E-003
reg_DEFB	YES	chr8	7179171	A	T	30	13,00%	ambig	4			4,76E-003
reg_DEFB	YES	chr8	7179183	T	A	30	37,00%	het	11			3,60E-011
reg_DEFB	YES	chr8	7179199	G	A	32	44,00%	het	14			1,00E-012
reg_DEFB	YES	chr8	7179203	A	G	32	44,00%	het	14			1,00E-012
reg_DEFB	YES	chr8	7179204	T	C	32	22,00%	ambig	7			6,91E-006
reg_DEFB	YES	chr8	7179213	C	G	32	44,00%	het	14			1,00E-012
reg_DEFB	YES	chr8	7179238	T	C	33	48,00%	het	16			1,00E-012
reg_DEFB	YES	chr8	7179263	T	A	30	50,00%	het	15			1,00E-012
reg_DEFB	YES	chr8	7179284	C	G	30	53,00%	het	16			1,00E-012
reg_DEFB	YES	chr8	7179324	G	T	31	45,00%	het	14			1,00E-012
reg_DEFB	YES	chr8	7179330	C	T	31	52,00%	het	16			1,00E-012
reg_DEFB	YES	chr8	7179332	G	A	31	52,00%	het	16			1,00E-012
reg_DEFB	YES	chr8	7179357	C	G	31	52,00%	het	16			1,00E-012
reg_DEFB	YES	chr8	7179396	A	G	29	10,00%	ambig	3			2,85E-002
reg_DEFB	YES	chr8	7179410	A	G	27	52,00%	het	14			1,00E-012
reg_DEFB	YES	chr8	7179416	T	G	27	52,00%	het	14			1,00E-012
reg_DEFB	YES	chr8	7179418	G	A	27	52,00%	het	14			1,00E-012
reg_DEFB	YES	chr8	7179439	A	C	24	54,00%	het	13			1,00E-012
reg_DEFB	YES	chr8	7179443	T	G	24	54,00%	het	13			1,00E-012
reg_DEFB	YES	chr8	7179461	T	C	22	50,00%	het	11			1,00E-012
reg_DEFB	YES	chr8	7179467	T	C	21	48,00%	het	10			1,84E-011
reg_DEFB	YES	chr8	7179473	G	T	22	45,00%	het	10			3,38E-011
reg_DEFB	YES	chr8	7179521	C	T	23	48,00%	het	11	rs34137692		1,66E-012
reg_DEFB	YES	chr8	7179529	A	G	23	48,00%	het	11			1,66E-012
reg_DEFB	YES	chr8	7179531	A	G	24	46,00%	het	11			3,07E-012
reg_DEFB	YES	chr8	7179558	G	T	25	44,00%	het	11		rs3882688	5,49E-012
reg_DEFB	YES	chr8	7179560	T	C	25	44,00%	het	11			5,49E-012
reg_DEFB	YES	chr8	7179563	A	G	25	44,00%	het	11			5,49E-012
reg_DEFB	YES	chr8	7179569	A	C	24	42,00%	het	10			6,17E-011
reg_DEFB	YES	chr8	7179597	T	G	17	29,00%	het	5			3,16E-005
reg_DEFB	YES	chr8	7179605	C	A	17	29,00%	het	5			3,16E-005
reg_DEFB	YES	chr8	7179612	T	C	14	29,00%	het	4			2,33E-004
reg_DEFB	YES	chr8	7179618	C	G	14	29,00%	het	4			2,33E-004
reg_DEFB	YES	chr8	7179933	A	T	14	86,00%	homvar	12		rs71511203	1,00E-012
reg_DEFB	YES	chr8	7180330	C	G	16	37,00%	het	6			9,72E-007
reg_DEFB	YES	chr8	7180392	T	C	20	40,00%	het	8			7,71E-009
reg_DEFB	YES	chr8	7180417	G	T	21	52,00%	het	11	rs34030924		1,00E-012

reg_DEFB	YES	chr8	7180486	T	C	25	40,00%	het	10			1,01E-010
reg_DEFB	YES	chr8	7180501	T	C	26	38,00%	het	10			1,61E-010
reg_DEFB	YES	chr8	7180513	T	G	25	52,00%	het	13			1,00E-012
reg_DEFB	YES	chr8	7180548	T	C	25	24,00%	ambig	6			1,80E-005
reg_DEFB	YES	chr8	7180550	G	C	25	36,00%	het	9			2,64E-009
reg_DEFB	YES	chr8	7180560	G	C	24	37,00%	het	9			1,72E-009
reg_DEFB	YES	chr8	7180565	T	G	25	36,00%	het	9			2,64E-009
reg_DEFB	YES	chr8	7180582	G	T	28	46,00%	het	13			1,00E-012
reg_DEFB	YES	chr8	7180595	G	C	29	48,00%	het	14			1,00E-012
reg_DEFB	YES	chr8	7180619	T	G	35	31,00%	het	11		rs71509297	2,52E-010
reg_DEFB	YES	chr8	7180635	T	G	37	30,00%	het	11		rs60601573	4,70E-010
reg_DEFB	YES	chr8	7180674	A	G	41	20,00%	ambig	8		rs2698948	3,80E-006
reg_DEFB	YES	chr8	7180678	C	A	41	12,00%	ambig	5			2,42E-003
reg_DEFB	YES	chr8	7180691	G	C	43	23,00%	ambig	10			3,96E-008
reg_DEFB	YES	chr8	7180700	A	C	45	24,00%	ambig	11			4,70E-009
reg_DEFB	YES	chr8	7180705	A	C	45	36,00%	het	16			1,00E-012
reg_DEFB	YES	chr8	7180711	A	G	45	24,00%	ambig	11			4,70E-009
reg_DEFB	YES	chr8	7180743	T	G	43	12,00%	ambig	5			2,99E-003
reg_DEFB	YES	chr8	7180752	C	G	38	13,00%	ambig	5			1,72E-003
reg_DEFB	YES	chr8	7180798	T	C	34	12,00%	ambig	4			7,48E-003
reg_DEFB	YES	chr8	7180844	G	A	31	13,00%	ambig	4			5,36E-003
reg_DEFB	YES	chr8	7180862	C	T	32	12,00%	ambig	4			6,02E-003
reg_DEFB	YES	chr8	7180866	A	C	32	12,00%	ambig	4			6,02E-003
reg_DEFB	YES	chr8	7180894	C	G	32	12,00%	ambig	4			6,02E-003
reg_DEFB	YES	chr8	7181101	C	T	29	90,00%	homvar	26	rs36114896		1,00E-012
reg_DEFB	YES	chr8	7181236	G	A	32	87,00%	homvar	28	rs35740946		1,00E-012
reg_DEFB	YES	chr8	7183521	T	C	24	25,00%	het	6			1,39E-005
reg_DEFB	YES	chr8	7183526	T	C	24	25,00%	het	6			1,39E-005
reg_DEFB	YES	chr8	7183610	A	T	26	19,00%	ambig	5			2,83E-004
reg_DEFB	YES	chr8	7183643	G	C	31	19,00%	ambig	6			6,64E-005
reg_DEFB	YES	chr8	7183653	A	G	32	16,00%	ambig	5			7,72E-004
reg_DEFB	YES	chr8	7183716	C	A	32	12,00%	ambig	4			6,02E-003
reg_DEFB	YES	chr8	7183727	C	A	33	12,00%	ambig	4			6,72E-003
reg_DEFB	YES	chr8	7184391	T	A	28	14,00%	ambig	4			3,68E-003
reg_DEFB	YES	chr8	7184454	A	G	26	19,00%	ambig	5			2,83E-004
reg_DEFB	YES	chr8	7184493	T	C	26	35,00%	het	9		rs55780142	3,95E-009
reg_DEFB	YES	chr8	7184501	A	G	25	36,00%	het	9			2,64E-009
reg_DEFB	YES	chr8	7184535	C	T	24	37,00%	het	9			1,72E-009
reg_DEFB	YES	chr8	7184657	G	A	21	29,00%	het	6			5,96E-006
reg_DEFB	YES	chr8	7184672	T	G	19	21,00%	ambig	4			8,22E-004
reg_DEFB	YES	chr8	7184688	C	T	19	79,00%	homvar	15			1,00E-012
reg_DEFB	YES	chr8	7184693	A	T	19	21,00%	ambig	4			8,22E-004

reg_DEFB	YES	chr8	7184705	T	G	19	21,00%	ambig	4		8,22E-004
reg_DEFB	YES	chr8	7184711	A	G	19	21,00%	ambig	4	rs3867443	8,22E-004
reg_DEFB	YES	chr8	7184731	A	C	22	27,00%	het	6		8,04E-006
reg_DEFB	YES	chr8	7184756	G	C	23	26,00%	het	6		1,07E-005
reg_DEFB	YES	chr8	7184796	T	C	25	32,00%	het	8		5,97E-008
reg_DEFB	YES	chr8	7184831	T	C	27	30,00%	het	8		1,18E-007
reg_DEFB	YES	chr8	7184868	C	T	31	16,00%	ambig	5	rs3989687	6,64E-004
reg_DEFB	YES	chr8	7184873	G	C	31	32,00%	het	10		1,18E-009
reg_DEFB	YES	chr8	7184892	A	G	29	34,00%	het	10		5,76E-010
reg_DEFB	YES	chr8	7184916	C	G	32	19,00%	ambig	6	rs28393679	8,02E-005
reg_DEFB	YES	chr8	7184967	C	T	31	32,00%	het	10		1,18E-009
reg_DEFB	YES	chr8	7184970	G	C	31	32,00%	het	10		1,18E-009
reg_DEFB	YES	chr8	7184985	C	T	29	17,00%	ambig	5		4,82E-004
reg_DEFB	YES	chr8	7185070	G	A	26	23,00%	ambig	6		2,29E-005
reg_DEFB	YES	chr8	7185097	A	G	26	42,00%	het	11	rs72496220	9,51E-012
reg_DEFB	YES	chr8	7185117	C	T	25	20,00%	ambig	5	rs60248625	2,33E-004
reg_DEFB	YES	chr8	7185138	G	T	25	20,00%	ambig	5	rs61044488	2,33E-004
reg_DEFB	YES	chr8	7185210	C	T	28	18,00%	ambig	5	rs13256402	4,06E-004
reg_DEFB	YES	chr8	7185212	C	T	28	14,00%	ambig	4		3,68E-003
reg_DEFB	YES	chr8	7185241	A	G	26	19,00%	ambig	5	rs13252616	2,83E-004
reg_DEFB	YES	chr8	7185256	G	A	25	36,00%	het	9		2,64E-009
reg_DEFB	YES	chr8	7185288	G	A	22	27,00%	het	6		8,04E-006
reg_DEFB	YES	chr8	7185340	A	T	18	22,00%	ambig	4	rs71509297	6,61E-004
reg_DEFB	YES	chr8	7185346	T	C	19	21,00%	ambig	4	rs61681254	8,22E-004
reg_DEFB	YES	chr8	7185388	A	G	21	14,00%	ambig	3		1,19E-002
reg_DEFB	YES	chr8	7185402	G	T	22	14,00%	ambig	3		1,35E-002
reg_DEFB	YES	chr8	7185422	T	G	23	13,00%	ambig	3		1,53E-002
reg_DEFB	YES	chr8	7185448	T	C	22	14,00%	ambig	3		1,35E-002
reg_DEFB	YES	chr8	7186692	A	G	32	100,00%	homvar	32	rs71507397	1,00E-012
reg_DEFB	YES	chr8	7186729	A	C	36	17,00%	ambig	6		1,59E-004
reg_DEFB	YES	chr8	7186753	G	T	40	22,00%	ambig	9	rs62636811	2,58E-007
reg_DEFB	YES	chr8	7186832	A	C	43	14,00%	ambig	6	rs62636813	4,35E-004
reg_DEFB	YES	chr8	7187793	G	T	44	18,00%	ambig	8		6,62E-006
reg_DEFB	YES	chr8	7187829	T	C	52	19,00%	ambig	10		2,71E-007
reg_DEFB	YES	chr8	7187910	G	T	56	21,00%	ambig	12		4,78E-009
reg_DEFB	YES	chr8	7187917	G	A	57	21,00%	ambig	12		5,92E-009
reg_DEFB	YES	chr8	7187918	C	T	57	23,00%	ambig	13		4,82E-010
reg_DEFB	YES	chr8	7187931	A	G	58	21,00%	ambig	12		7,31E-009
reg_DEFB	YES	chr8	7187943	T	C	60	22,00%	ambig	13		9,56E-010
reg_DEFB	YES	chr8	7187951	A	G	58	22,00%	ambig	13		6,09E-010
reg_DEFB	YES	chr8	7187955	T	G	58	22,00%	ambig	13		6,09E-010
reg_DEFB	YES	chr8	7187975	T	A	57	23,00%	ambig	13		4,82E-010

reg_DEFB	YES	chr8	7187998	C	T	59	22,00%	ambig	13			7,64E-010
reg_DEFB	YES	chr8	7187999	A	G	59	22,00%	ambig	13			7,64E-010
reg_DEFB	YES	chr8	7188045	G	C	60	18,00%	ambig	11			1,15E-007
reg_DEFB	YES	chr8	7188087	T	C	54	15,00%	ambig	8		rs71507400	3,17E-005
reg_DEFB	YES	chr8	7188090	G	C	54	13,00%	ambig	7			2,34E-004
reg_DEFB	YES	chr8	7188096	C	T	54	13,00%	ambig	7			2,34E-004
reg_DEFB	YES	chr8	7188105	T	C	55	13,00%	ambig	7			2,62E-004
reg_DEFB	YES	chr8	7188112	A	C	55	13,00%	ambig	7			2,62E-004
reg_DEFB	YES	chr8	7188124	C	T	55	13,00%	ambig	7			2,62E-004
reg_DEFB	YES	chr8	7188129	T	C	53	13,00%	ambig	7			2,08E-004
reg_DEFB	YES	chr8	7188148	G	T	51	14,00%	ambig	7			1,62E-004
reg_DEFB	YES	chr8	7188157	A	C	49	20,00%	ambig	10		rs6990411	1,50E-007
reg_DEFB	YES	chr8	7188181	A	G	49	12,00%	ambig	6			8,86E-004
reg_DEFB	YES	chr8	7188183	A	T	48	12,00%	ambig	6			7,93E-004
reg_DEFB	YES	chr8	7188670	C	A	50	12,00%	ambig	6			9,88E-004
reg_DEFB	YES	chr8	7188683	A	G	48	15,00%	ambig	7			1,10E-004
reg_DEFB	YES	chr8	7188696	G	A	49	14,00%	ambig	7			1,25E-004
reg_DEFB	YES	chr8	7188705	A	G	53	13,00%	ambig	7			2,08E-004
reg_DEFB	YES	chr8	7188707	A	T	53	17,00%	ambig	9			3,19E-006
reg_DEFB	YES	chr8	7188724	C	T	54	13,00%	ambig	7		rs3988914	2,34E-004
reg_DEFB	YES	chr8	7188736	A	G	54	13,00%	ambig	7			2,34E-004
reg_DEFB	YES	chr8	7188785	C	T	48	19,00%	ambig	9			1,34E-006
reg_DEFB	YES	chr8	7188793	G	T	48	19,00%	ambig	9			1,34E-006
reg_DEFB	YES	chr8	7188797	A	G	48	31,00%	het	15			1,00E-012
reg_DEFB	YES	chr8	7188803	G	T	48	19,00%	ambig	9			1,34E-006
reg_DEFB	YES	chr8	7188819	T	G	46	20,00%	ambig	9			9,18E-007
reg_DEFB	YES	chr8	7188823	T	C	46	17,00%	ambig	8			9,36E-006
reg_DEFB	YES	chr8	7188856	A	C	39	44,00%	het	17			1,00E-012
reg_DEFB	YES	chr8	7188876	A	G	36	25,00%	het	9			9,66E-008
reg_DEFB	YES	chr8	7188887	G	A	35	49,00%	het	17			1,00E-012
reg_DEFB	YES	chr8	7188934	A	T	33	27,00%	het	9			4,21E-008
reg_DEFB	YES	chr8	7188958	C	A	33	24,00%	ambig	8		rs34459218	6,50E-007
reg_DEFB	YES	chr8	7188981	C	G	34	50,00%	het	17		rs9987086	1,00E-012
reg_DEFB	YES	chr8	7188989	A	T	34	26,00%	het	9		rs36162872	5,61E-008
reg_DEFB	YES	chr8	7189009	T	A	30	47,00%	het	14		rs62493294	1,00E-012
reg_DEFB	YES	chr8	7189013	C	G	28	11,00%	ambig	3			2,60E-002
reg_DEFB	YES	chr8	7189032	C	G	20	20,00%	ambig	4			1,01E-003
reg_DEFB	YES	chr8	7189037	A	T	19	37,00%	het	7		rs6986093	1,34E-007
reg_DEFB	YES	chr8	7189054	A	G	15	27,00%	het	4		rs4733735	3,12E-004
reg_DEFB	YES	chr8	7189204	T	C	8	50,00%	het	4		rs71525470	1,82E-005
reg_DEFB	YES	chr8	7189210	T	A	7	57,00%	het	4		rs71514680	9,26E-006
reg_DEFB	YES	chr8	7189248	A	G	9	56,00%	het	5		rs55983859	7,51E-007

reg_DEFB	YES	chr8	7189264	T	G	11	36,00%	het	4	rs41480145			8,11E-005
reg_DEFB	YES	chr8	7189314	C	A	17	29,00%	het	5			rs71522940	3,16E-005
reg_DEFB	YES	chr8	7189338	G	A	19	32,00%	het	6			rs62507166	3,10E-006
reg_DEFB	YES	chr8	7189349	C	G	20	30,00%	het	6			rs813630	4,35E-006
reg_DEFB	YES	chr8	7189475	G	A	22	23,00%	ambig	5				1,22E-004
reg_DEFB	YES	chr8	7189479	C	A	22	23,00%	ambig	5			rs7830327	1,22E-004
reg_DEFB	YES	chr8	7189483	G	T	22	23,00%	ambig	5			rs2204808	1,22E-004
reg_DEFB	YES	chr8	7189491	T	G	22	23,00%	ambig	5			rs7833507	1,22E-004
reg_DEFB	YES	chr8	7189540	C	G	24	21,00%	ambig	5				1,90E-004
reg_DEFB	YES	chr8	7189575	A	G	20	20,00%	ambig	4				1,01E-003
reg_DEFB	YES	chr8	7189590	T	A	18	22,00%	ambig	4			rs10955320	6,61E-004
reg_DEFB	YES	chr8	7189611	C	G	18	17,00%	ambig	3				7,66E-003
reg_DEFB	YES	chr8	7191450	A	G	10	40,00%	het	4				5,26E-005
reg_DEFB	YES	chr8	7191452	A	G	11	27,00%	het	3				1,75E-003
reg_DEFB	YES	chr8	7191518	C	A	22	18,00%	ambig	4				1,47E-003
reg_DEFB	YES	chr8	7191671	C	T	58	36,00%	het	21				1,00E-012
reg_DEFB	YES	chr8	7191773	T	C	64	27,00%	het	17			rs55699527	1,00E-012
reg_DEFB	YES	chr8	7191776	C	A	64	28,00%	het	18				1,00E-012
reg_DEFB	YES	chr8	7191808	C	T	70	16,00%	ambig	11				5,90E-007
reg_DEFB	YES	chr8	7191810	G	A	70	19,00%	ambig	13				7,03E-009
reg_DEFB	YES	chr8	7191820	T	C	67	24,00%	ambig	16				6,34E-012
reg_DEFB	YES	chr8	7191873	T	C	57	12,00%	ambig	7				3,29E-004
reg_DEFB	YES	chr8	7193291	C	T	23	13,00%	ambig	3				1,53E-002
reg_DEFB	YES	chr8	7193339	A	G	26	15,00%	ambig	4				2,79E-003
reg_DEFB	YES	chr8	7193367	C	T	27	15,00%	ambig	4				3,22E-003
reg_DEFB	YES	chr8	7193379	G	C	29	14,00%	ambig	4				4,20E-003
reg_DEFB	YES	chr8	7193412	A	G	26	27,00%	het	7				1,52E-006
reg_DEFB	YES	chr8	7193428	A	G	28	14,00%	ambig	4				3,68E-003
reg_DEFB	YES	chr8	7193449	C	T	28	11,00%	ambig	3		rs2698920		2,60E-002
reg_DEFB	YES	chr8	7193455	C	T	28	11,00%	ambig	3				2,60E-002
reg_DEFB	YES	chr8	7193462	T	C	29	31,00%	het	9				1,19E-008
reg_DEFB	YES	chr8	7193467	A	G	28	14,00%	ambig	4				3,68E-003
reg_DEFB	YES	chr8	7193474	G	A	28	14,00%	ambig	4				3,68E-003
reg_DEFB	YES	chr8	7193490	T	C	26	35,00%	het	9				3,95E-009
reg_DEFB	YES	chr8	7193496	C	T	25	12,00%	ambig	3				1,92E-002
reg_DEFB	YES	chr8	7193556	A	G	24	12,00%	ambig	3				1,72E-002
reg_DEFB	YES	chr8	7193576	A	C	22	14,00%	ambig	3				1,35E-002
reg_DEFB	YES	chr8	7193593	C	G	22	14,00%	ambig	3				1,35E-002
reg_DEFB	YES	chr8	7193966	C	A	49	20,00%	ambig	10				1,50E-007
reg_DEFB	YES	chr8	7194072	A	G	64	53,00%	het	34			rs71511216	1,00E-012
reg_DEFB	YES	chr8	7194133	G	A	79	15,00%	ambig	12			rs71518600	2,68E-007
reg_DEFB	YES	chr8	7194174	G	A	82	12,00%	ambig	10	rs2719612			1,96E-005

reg_DEFB	YES	chr8	7194214	A	G	81	11,00%	ambig	9			1,06E-004
reg_DEFB	YES	chr8	7194267	C	G	81	53,00%	het	43	rs28413957		1,00E-012
reg_DEFB	YES	chr8	7194497	T	A	96	19,00%	ambig	18		rs4109383	9,81E-012
reg_DEFB	YES	chr8	7194523	T	C	91	12,00%	ambig	11			8,29E-006
reg_DEFB	YES	chr8	7194566	G	A	95	17,00%	ambig	16			6,05E-010
reg_DEFB	YES	chr8	7194629	G	C	97	10,00%	ambig	10			8,42E-005
reg_DEFB	YES	chr8	7194683	C	G	108	13,00%	ambig	14			2,13E-007
reg_DEFB	YES	chr8	7194714	T	C	134	27,00%	het	36		rs71511217	1,00E-012
reg_DEFB	YES	chr8	7194761	A	C	151	15,00%	ambig	23			4,21E-012
reg_DEFB	YES	chr8	7194793	C	G	172	13,00%	ambig	22			1,17E-010
reg_DEFB	YES	chr8	7194795	C	T	172	12,00%	ambig	21			6,86E-010
reg_DEFB	YES	chr8	7194912	C	T	173	28,00%	het	48			1,00E-012
reg_DEFB	YES	chr8	7194919	A	C	173	24,00%	ambig	42			1,00E-012
reg_DEFB	YES	chr8	7194944	A	G	169	19,00%	ambig	32			1,00E-012
reg_DEFB	YES	chr8	7194961	G	T	168	40,00%	het	67			1,00E-012
reg_DEFB	YES	chr8	7194995	G	A	165	11,00%	ambig	18			6,34E-008
reg_DEFB	YES	chr8	7195016	A	C	169	10,00%	ambig	17			4,67E-007
reg_DEFB	YES	chr8	7195039	T	C	164	29,00%	het	48			1,00E-012
reg_DEFB	YES	chr8	7195053	C	T	164	57,00%	het	93		rs4406416	1,00E-012
reg_DEFB	YES	chr8	7195058	T	A	167	32,00%	het	53			1,00E-012
reg_DEFB	YES	chr8	7195060	T	C	168	12,00%	ambig	20			2,67E-009
reg_DEFB	YES	chr8	7195091	T	G	162	15,00%	ambig	24			2,65E-012
reg_DEFB	YES	chr8	7195109	T	C	158	14,00%	ambig	22	rs4118268		2,86E-011
reg_DEFB	YES	chr8	7195125	A	T	145	35,00%	het	51			1,00E-012
reg_DEFB	YES	chr8	7195130	A	T	146	21,00%	ambig	31			1,00E-012
reg_DEFB	YES	chr8	7195137	T	C	142	48,00%	het	68	rs2698983		1,00E-012
reg_DEFB	YES	chr8	7195138	G	A	142	35,00%	het	50			1,00E-012
reg_DEFB	YES	chr8	7195192	G	A	135	26,00%	het	35			1,00E-012
reg_DEFB	YES	chr8	7195194	C	T	134	27,00%	het	36			1,00E-012
reg_DEFB	YES	chr8	7195256	T	C	116	11,00%	ambig	13			3,04E-006
reg_DEFB	YES	chr8	7195278	G	C	118	11,00%	ambig	13			3,69E-006
reg_DEFB	YES	chr8	7195310	T	C	121	15,00%	ambig	18			4,34E-010
reg_DEFB	YES	chr8	7195438	C	T	120	31,00%	het	37	rs2684172		1,00E-012
reg_DEFB	YES	chr8	7195583	G	T	116	15,00%	ambig	17			1,66E-009
reg_DEFB	YES	chr8	7195614	C	A	107	12,00%	ambig	13			1,21E-006
reg_DEFB	YES	chr8	7195621	C	A	106	23,00%	ambig	24		rs2739953	1,00E-012
reg_DEFB	YES	chr8	7195701	A	G	89	30,00%	het	27			1,00E-012
reg_DEFB	YES	chr8	7195707	C	A	88	10,00%	ambig	9			2,00E-004
reg_DEFB	YES	chr8	7195712	G	A	89	16,00%	ambig	14			1,75E-008
reg_DEFB	YES	chr8	7195721	T	C	86	47,00%	het	40			1,00E-012
reg_DEFB	YES	chr8	7195754	G	A	91	13,00%	ambig	12			1,28E-006
reg_DEFB	YES	chr8	7195766	A	T	93	23,00%	ambig	21			1,00E-012

reg_DEFB	YES	chr8	7195768	C	T	93	23,00%	ambig	21			1,00E-012
reg_DEFB	YES	chr8	7195788	T	C	88	23,00%	ambig	20			1,00E-012
reg_DEFB	YES	chr8	7195875	C	G	92	26,00%	het	24	rs9644813		1,00E-012
reg_DEFB	YES	chr8	7195903	C	T	87	55,00%	het	48			1,00E-012
reg_DEFB	YES	chr8	7195918	A	G	86	53,00%	het	46			1,00E-012
reg_DEFB	YES	chr8	7195928	C	G	83	24,00%	ambig	20			1,00E-012
reg_DEFB	YES	chr8	7196045	T	C	81	39,00%	het	32			1,00E-012
reg_DEFB	YES	chr8	7196080	G	T	90	29,00%	het	26			1,00E-012
reg_DEFB	YES	chr8	7196137	G	A	82	16,00%	ambig	13			5,10E-008
reg_DEFB	YES	chr8	7196166	T	A	64	22,00%	ambig	14			1,90E-010
reg_DEFB	YES	chr8	7196370	A	G	89	56,00%	het	50	rs11786963		1,00E-012
reg_DEFB	YES	chr8	7196542	A	G	96	58,00%	het	56	rs2719605		1,00E-012
reg_DEFB	YES	chr8	7196637	G	T	129	10,00%	ambig	13			9,88E-006
reg_DEFB	YES	chr8	7196648	G	A	127	13,00%	ambig	17			6,79E-009
reg_DEFB	YES	chr8	7196689	T	C	125	10,00%	ambig	13			6,99E-006
reg_DEFB	YES	chr8	7197406	A	G	23	22,00%	ambig	5	rs7005358		1,53E-004
reg_DEFB	YES	chr8	7197460	T	C	16	25,00%	het	4	rs6990088		4,08E-004
reg_DEFB	YES	chr8	7198085	C	G	57	39,00%	het	22	rs2719598		1,00E-012
reg_DEFB	YES	chr8	7198279	G	A	45	16,00%	ambig	7		rs71511218	7,17E-005
reg_DEFB	YES	chr8	7198657	T	A	19	32,00%	het	6			3,10E-006
reg_DEFB	YES	chr8	7198660	A	G	20	40,00%	het	8			7,71E-009
reg_DEFB	YES	chr8	7198710	C	T	29	21,00%	ambig	6			4,46E-005
reg_DEFB	YES	chr8	7198835	T	C	62	16,00%	ambig	10			1,49E-006
reg_DEFB	YES	chr8	7198855	C	T	68	43,00%	het	29			1,00E-012
reg_DEFB	YES	chr8	7198887	G	A	77	19,00%	ambig	15			2,48E-010
reg_DEFB	YES	chr8	7198888	C	A	77	19,00%	ambig	15			2,48E-010
reg_DEFB	YES	chr8	7198917	G	T	80	10,00%	ambig	8			5,21E-004
reg_DEFB	YES	chr8	7198932	G	T	81	14,00%	ambig	11			2,62E-006
reg_DEFB	YES	chr8	7198953	A	G	85	44,00%	het	37			1,00E-012
reg_DEFB	YES	chr8	7198957	G	A	86	19,00%	ambig	16			1,31E-010
reg_DEFB	YES	chr8	7198985	C	T	92	18,00%	ambig	17			4,72E-011
reg_DEFB	YES	chr8	7198997	C	T	92	42,00%	het	39			1,00E-012
reg_DEFB	YES	chr8	7199006	A	T	93	43,00%	het	40			1,00E-012
reg_DEFB	YES	chr8	7199022	G	T	95	41,00%	het	39			1,00E-012
reg_DEFB	YES	chr8	7199050	C	A	101	15,00%	ambig	15			1,23E-008
reg_DEFB	YES	chr8	7199055	A	T	104	17,00%	ambig	18			4,09E-011
reg_DEFB	YES	chr8	7199057	G	A	105	39,00%	het	41		rs2739951	1,00E-012
reg_DEFB	YES	chr8	7199070	T	G	102	15,00%	ambig	15			1,41E-008
reg_DEFB	YES	chr8	7199090	C	T	99	22,00%	ambig	22			1,00E-012
reg_DEFB	YES	chr8	7199114	G	A	94	11,00%	ambig	10			6,44E-005
reg_DEFB	YES	chr8	7199209	A	G	89	17,00%	ambig	15		rs2698933	2,04E-009
reg_DEFB	YES	chr8	7199246	T	G	100	17,00%	ambig	17	rs2719594		1,56E-010

reg_DEFB	YES	chr8	7199260	T	G	105	13,00%	ambig	14							1,49E-007
reg_DEFB	YES	chr8	7199565	T	C	78	15,00%	ambig	12					rs41494048		2,32E-007
reg_DEFB	YES	chr8	7199580	G	A	70	14,00%	ambig	10					rs2719592		4,66E-006
reg_DEFB	YES	chr8	7199703	G	A	40	20,00%	ambig	8						rs4452804	3,12E-006
reg_DEFB	YES	chr8	7200170	C	T	9	44,00%	het	4					rs11137098		3,21E-005
reg_DEFB	YES	chr8	7201007	C	G	83	51,00%	het	42						rs59917219	1,00E-012
reg_DEFB	YES	chr8	7201053	T	C	81	49,00%	het	40						rs71511221	1,00E-012
reg_DEFB	YES	chr8	7201387	C	T	83	34,00%	het	28					rs9720953		1,00E-012
reg_DEFB	YES	chr8	7201430	A	T	100	11,00%	ambig	11							2,05E-005
reg_DEFB	YES	chr8	7201552	G	A	108	12,00%	ambig	13							1,35E-006
reg_DEFB	YES	chr8	7201662	A	G	118	13,00%	ambig	15							1,03E-007
reg_DEFB	YES	chr8	7201669	G	A	120	25,00%	het	30							1,00E-012
reg_DEFB	YES	chr8	7201678	C	G	125	24,00%	ambig	30							1,00E-012
reg_DEFB	YES	chr8	7201691	C	T	120	22,00%	ambig	26							1,00E-012
reg_DEFB	YES	chr8	7201816	C	T	112	18,00%	ambig	20							5,18E-012
reg_DEFB	YES	chr8	7201817	G	T	112	18,00%	ambig	20							5,18E-012
reg_DEFB	YES	chr8	7201824	A	T	109	10,00%	ambig	11							4,61E-005
reg_DEFB	YES	chr8	7201852	T	C	105	11,00%	ambig	12							5,89E-006
reg_DEFB	YES	chr8	7202739	T	C	49	57,00%	het	28					rs9720695		1,00E-012
reg_DEFB	YES	chr8	7202754	C	T	49	45,00%	het	22						rs55799624	1,00E-012
reg_DEFB	YES	chr8	7202856	T	C	56	46,00%	het	26					rs9693661		1,00E-012
reg_DEFB	YES	chr8	7203283	T	A	53	13,00%	ambig	7	L	L	-1	ZNF705G			2,08E-004
reg_DEFB	YES	chr8	7203333	C	T	57	14,00%	ambig	8	E	K	-1	ZNF705G			4,73E-005
reg_DEFB	YES	chr8	7203336	T	G	56	12,00%	ambig	7	T	P	-1	ZNF705G			2,94E-004
reg_DEFB	YES	chr8	7203350	C	T	56	11,00%	ambig	6	R	H	-1	ZNF705G			1,80E-003
reg_DEFB	YES	chr8	7203356	G	C	54	11,00%	ambig	6	S	C	-1	ZNF705G			1,49E-003
reg_DEFB	YES	chr8	7203431	C	T	51	20,00%	ambig	10	R	H	-1	ZNF705G		rs59326007	2,23E-007
reg_DEFB	YES	chr8	7203440	T	C	49	12,00%	ambig	6	D	G	-1	ZNF705G			8,86E-004
reg_DEFB	YES	chr8	7203449	G	A	49	12,00%	ambig	6	S	L	-1	ZNF705G			8,86E-004
reg_DEFB	YES	chr8	7203472	C	A	50	12,00%	ambig	6	E	D	-1	ZNF705G			9,88E-004
reg_DEFB	YES	chr8	7203505	T	G	48	15,00%	ambig	7			-	ZNF705G			1,10E-004
reg_DEFB	YES	chr8	7203509	A	T	48	10,00%	ambig	5			-	ZNF705G			4,85E-003
reg_DEFB	YES	chr8	7203870	G	A	35	40,00%	het	14			-	ZNF705G	rs12682338		1,00E-012
reg_DEFB	YES	chr8	7204218	A	T	52	25,00%	het	13			-	ZNF705G			1,52E-010
reg_DEFB	YES	chr8	7204226	C	T	56	12,00%	ambig	7			-	ZNF705G			2,94E-004
reg_DEFB	YES	chr8	7204281	C	G	66	12,00%	ambig	8			-	ZNF705G			1,37E-004
reg_DEFB	YES	chr8	7204289	C	T	65	12,00%	ambig	8			-	ZNF705G			1,23E-004
reg_DEFB	YES	chr8	7204299	T	A	66	14,00%	ambig	9			-	ZNF705G			2,04E-005
reg_DEFB	YES	chr8	7204317	G	A	66	21,00%	ambig	14			-	ZNF705G			2,94E-010
reg_DEFB	YES	chr8	7204321	A	T	65	23,00%	ambig	15			-	ZNF705G			2,10E-011
reg_DEFB	YES	chr8	7204354	A	G	75	13,00%	ambig	10			-	ZNF705G			8,77E-006
reg_DEFB	YES	chr8	7204417	C	T	84	19,00%	ambig	16			-	ZNF705G			9,04E-011

reg_DEFB	YES	chr8	7204442	A	G	79	16,00%	ambig	13			-	ZNF705G			3,22E-008
reg_DEFB	YES	chr8	7204457	T	C	77	26,00%	het	20			-	ZNF705G			1,00E-012
reg_DEFB	YES	chr8	7204497	T	C	77	53,00%	het	41			-	ZNF705G		rs55661827	1,00E-012
reg_DEFB	YES	chr8	7204504	A	C	77	23,00%	ambig	18			-	ZNF705G			1,00E-012
reg_DEFB	YES	chr8	7204572	G	A	73	11,00%	ambig	8	D	D	-2	ZNF705G			2,78E-004
reg_DEFB	YES	chr8	7204582	G	A	71	45,00%	het	32	T	I	-2	ZNF705G		rs56023905	1,00E-012
reg_DEFB	YES	chr8	7204701	A	G	65	35,00%	het	23			-	ZNF705G		rs56111220	1,00E-012
reg_DEFB	YES	chr8	7204731	G	T	76	12,00%	ambig	9			-	ZNF705G			6,39E-005
reg_DEFB	YES	chr8	7204796	A	G	88	23,00%	ambig	20			-	ZNF705G			1,00E-012
reg_DEFB	YES	chr8	7204809	T	G	88	37,00%	het	33			-	ZNF705G			1,00E-012
reg_DEFB	YES	chr8	7204869	A	T	108	46,00%	het	50			-	ZNF705G			1,00E-012
reg_DEFB	YES	chr8	7204899	A	G	110	27,00%	het	30			-	ZNF705G			1,00E-012
reg_DEFB	YES	chr8	7204956	C	T	117	25,00%	het	29			-	ZNF705G			1,00E-012
reg_DEFB	YES	chr8	7204963	G	A	115	24,00%	ambig	28			-	ZNF705G			1,00E-012
reg_DEFB	YES	chr8	7205020	T	C	110	28,00%	het	31			-	ZNF705G			1,00E-012
reg_DEFB	YES	chr8	7205055	A	G	101	18,00%	ambig	18			-	ZNF705G			2,43E-011
reg_DEFB	YES	chr8	7205169	T	C	72	35,00%	het	25	S	G	-2	ZNF705G			1,00E-012
reg_DEFB	YES	chr8	7205192	A	T	61	29,00%	het	18	V	E	-2	ZNF705G			1,00E-012
reg_DEFB	YES	chr8	7205201	T	C	59	27,00%	het	16	E	G	-2	ZNF705G			1,00E-012
reg_DEFB	YES	chr8	7205205	T	G	58	47,00%	het	27	R	R	-2	ZNF705G	rs9721188		1,00E-012
reg_DEFB	YES	chr8	7205247	A	C	57	44,00%	het	25	S	A	-2	ZNF705G	rs3989701		1,00E-012
reg_DEFB	YES	chr8	7205528	G	A	91	21,00%	ambig	19			-	ZNF705G			1,00E-012
reg_DEFB	YES	chr8	7205551	T	C	100	12,00%	ambig	12			-	ZNF705G			3,52E-006
reg_DEFB	YES	chr8	7205569	T	A	108	22,00%	ambig	24			-	ZNF705G			1,00E-012
reg_DEFB	YES	chr8	7205590	C	T	110	30,00%	het	33			-	ZNF705G			1,00E-012
reg_DEFB	YES	chr8	7205625	A	T	110	16,00%	ambig	18			-	ZNF705G			8,84E-011
reg_DEFB	YES	chr8	7205630	C	G	112	16,00%	ambig	18			-	ZNF705G			1,20E-010
reg_DEFB	YES	chr8	7205634	A	T	111	16,00%	ambig	18			-	ZNF705G			1,03E-010
reg_DEFB	YES	chr8	7205677	C	A	124	43,00%	het	53			-	ZNF705G			1,00E-012
reg_DEFB	YES	chr8	7205696	A	G	130	17,00%	ambig	22			-	ZNF705G			1,00E-012
reg_DEFB	YES	chr8	7205712	G	A	136	17,00%	ambig	23			-	ZNF705G			1,00E-012
reg_DEFB	YES	chr8	7205738	T	G	142	18,00%	ambig	26			-	ZNF705G			1,00E-012
reg_DEFB	YES	chr8	7205746	C	T	141	48,00%	het	68			-	ZNF705G			1,00E-012
reg_DEFB	YES	chr8	7205753	C	T	143	69,00%	het	99			-	ZNF705G	rs3958831		1,00E-012
reg_DEFB	YES	chr8	7205758	A	G	145	15,00%	ambig	22			-	ZNF705G			1,38E-011
reg_DEFB	YES	chr8	7205760	T	C	143	35,00%	het	50			-	ZNF705G			1,00E-012
reg_DEFB	YES	chr8	7205778	A	G	145	21,00%	ambig	30			-	ZNF705G	rs3989700		1,00E-012
reg_DEFB	YES	chr8	7205887	C	G	121	12,00%	ambig	15			-	ZNF705G			1,44E-007
reg_DEFB	YES	chr8	7205901	G	A	117	14,00%	ambig	16			-	ZNF705G			1,37E-008
reg_DEFB	YES	chr8	7205911	C	T	113	32,00%	het	36			-	ZNF705G			1,00E-012
reg_DEFB	YES	chr8	7205938	T	G	112	31,00%	het	35			-	ZNF705G			1,00E-012
reg_DEFB	YES	chr8	7205976	T	C	94	20,00%	ambig	19			-	ZNF705G			1,00E-012

reg_DEFB	YES	chr8	7205999	T	C	94	14,00%	ambig	13			-	ZNF705G		2,66E-007
reg_DEFB	YES	chr8	7206008	C	G	91	13,00%	ambig	12			-	ZNF705G		1,28E-006
reg_DEFB	YES	chr8	7206031	C	G	89	48,00%	het	43			-	ZNF705G	rs3989699	1,00E-012
reg_DEFB	YES	chr8	7206042	C	T	92	40,00%	het	37	G	S	-1	ZNF705G	rs3989698	1,00E-012
reg_DEFB	YES	chr8	7206162	G	C	106	57,00%	het	60	L	V	-1	ZNF705G	rs3989697	1,00E-012
reg_DEFB	YES	chr8	7206207	T	C	124	18,00%	ambig	22			-	ZNF705G		1,00E-012
reg_DEFB	YES	chr8	7206251	C	A	142	14,00%	ambig	20			-	ZNF705G		1,37E-010
reg_DEFB	YES	chr8	7206282	G	A	149	15,00%	ambig	22			-	ZNF705G		8,46E-012
reg_DEFB	YES	chr8	7206307	C	G	151	17,00%	ambig	26			-	ZNF705G		1,00E-012
reg_DEFB	YES	chr8	7206323	C	A	156	17,00%	ambig	27			-	ZNF705G		1,00E-012
reg_DEFB	YES	chr8	7206369	G	C	154	29,00%	het	45			-	ZNF705G		1,00E-012
reg_DEFB	YES	chr8	7206389	A	G	158	35,00%	het	55			-	ZNF705G		1,00E-012
reg_DEFB	YES	chr8	7206391	A	G	156	15,00%	ambig	23			-	ZNF705G		8,56E-012
reg_DEFB	YES	chr8	7206401	G	A	155	19,00%	ambig	29			-	ZNF705G	rs11986853	1,00E-012
reg_DEFB	YES	chr8	7206449	A	G	150	21,00%	ambig	32			-	ZNF705G		1,00E-012
reg_DEFB	YES	chr8	7206561	C	T	121	34,00%	het	41			-	ZNF705G		1,00E-012
reg_DEFB	YES	chr8	7206566	G	A	122	17,00%	ambig	21			-	ZNF705G	rs3989696	3,23E-012
reg_DEFB	YES	chr8	7206569	T	C	123	24,00%	ambig	30			-	ZNF705G		1,00E-012
reg_DEFB	YES	chr8	7206574	C	G	126	44,00%	het	55			-	ZNF705G		1,00E-012
reg_DEFB	YES	chr8	7206587	T	C	126	16,00%	ambig	20			-	ZNF705G		1,87E-011
reg_DEFB	YES	chr8	7206596	C	T	125	48,00%	het	60			-	ZNF705G		1,00E-012
reg_DEFB	YES	chr8	7206634	A	G	120	23,00%	ambig	28			-	ZNF705G		1,00E-012
reg_DEFB	YES	chr8	7206666	A	G	117	13,00%	ambig	15			-	ZNF705G		9,21E-008
reg_DEFB	YES	chr8	7206679	C	T	116	16,00%	ambig	19			-	ZNF705G		3,30E-011
reg_DEFB	YES	chr8	7206722	G	A	106	19,00%	ambig	20			-	ZNF705G		1,00E-012
reg_DEFB	YES	chr8	7206730	T	C	106	17,00%	ambig	18			-	ZNF705G		5,72E-011
reg_DEFB	YES	chr8	7206756	C	A	102	15,00%	ambig	15			-	ZNF705G		1,41E-008
reg_DEFB	YES	chr8	7206785	C	A	95	13,00%	ambig	12			-	ZNF705G		2,04E-006
reg_DEFB	YES	chr8	7206819	T	C	93	52,00%	het	48			-	ZNF705G		1,00E-012
reg_DEFB	YES	chr8	7206830	T	C	93	23,00%	ambig	21			-	ZNF705G		1,00E-012
reg_DEFB	YES	chr8	7206852	A	G	94	51,00%	het	48			-	ZNF705G		1,00E-012
reg_DEFB	YES	chr8	7206930	C	T	101	35,00%	het	35			-	ZNF705G		1,00E-012
reg_DEFB	YES	chr8	7206932	A	G	100	12,00%	ambig	12			-	ZNF705G		3,52E-006
reg_DEFB	YES	chr8	7206944	A	T	97	20,00%	ambig	19			-	ZNF705G		3,14E-012
reg_DEFB	YES	chr8	7206994	A	G	87	10,00%	ambig	9			-	ZNF705G		1,83E-004
reg_DEFB	YES	chr8	7206999	C	T	85	35,00%	het	30			-	ZNF705G		1,00E-012
reg_DEFB	YES	chr8	7207022	C	G	82	12,00%	ambig	10			-	ZNF705G		1,96E-005
reg_DEFB	YES	chr8	7207028	A	T	81	33,00%	het	27			-	ZNF705G		1,00E-012
reg_DEFB	YES	chr8	7207045	T	A	78	14,00%	ambig	11			-	ZNF705G		1,79E-006
reg_DEFB	YES	chr8	7207076	C	T	82	17,00%	ambig	14			-	ZNF705G		5,87E-009
reg_DEFB	YES	chr8	7207091	A	G	84	11,00%	ambig	9			-	ZNF705G		1,40E-004
reg_DEFB	YES	chr8	7207152	A	G	90	16,00%	ambig	14			-	ZNF705G		2,03E-008

reg_DEFB	YES	chr8	7207187	T	C	93	33,00%	het	31	-	ZNF705G		1,00E-012
reg_DEFB	YES	chr8	7207189	C	G	95	15,00%	ambig	14	-	ZNF705G		4,12E-008
reg_DEFB	YES	chr8	7207228	G	A	93	26,00%	het	24	-	ZNF705G		1,00E-012
reg_DEFB	YES	chr8	7207234	G	A	91	15,00%	ambig	14	-	ZNF705G		2,35E-008
reg_DEFB	YES	chr8	7207252	G	A	93	14,00%	ambig	13	-	ZNF705G		2,35E-007
reg_DEFB	YES	chr8	7207260	T	C	92	14,00%	ambig	13	-	ZNF705G		2,06E-007
reg_DEFB	YES	chr8	7207297	C	T	82	17,00%	ambig	14	-	ZNF705G		5,87E-009
reg_DEFB	YES	chr8	7207342	T	C	88	12,00%	ambig	11	-	ZNF705G		5,97E-006
reg_DEFB	YES	chr8	7208147	G	C	14	29,00%	het	4			rs3989692	2,33E-004
reg_DEFB	YES	chr8	7208307	C	T	12	25,00%	het	3			rs9773545	2,29E-003
reg_DEFB	YES	chr8	7208353	G	A	16	37,00%	het	6			rs12544086	9,72E-007
reg_DEFB	YES	chr8	7208440	T	G	21	38,00%	het	8			rs3989691	1,22E-008
reg_DEFB	YES	chr8	7208546	T	G	12	50,00%	het	6			rs12542313	1,21E-007
reg_DEFB	YES	chr8	7208550	C	T	11	45,00%	het	5			rs12549121	2,65E-006
reg_DEFB	YES	chr8	7208586	G	T	16	25,00%	het	4				4,08E-004
reg_DEFB	YES	chr8	7208589	G	C	17	29,00%	het	5				3,16E-005
reg_DEFB	YES	chr8	7208591	A	G	17	24,00%	ambig	4				5,24E-004
reg_DEFB	YES	chr8	7208614	A	G	23	17,00%	ambig	4				1,75E-003
reg_DEFB	YES	chr8	7208657	A	G	32	41,00%	het	13				1,00E-012
reg_DEFB	YES	chr8	7208678	A	G	37	24,00%	ambig	9				1,25E-007
reg_DEFB	YES	chr8	7208696	T	G	41	15,00%	ambig	6				3,33E-004
reg_DEFB	YES	chr8	7208717	C	A	46	13,00%	ambig	6				6,30E-004
reg_DEFB	YES	chr8	7208731	T	C	48	21,00%	ambig	10				1,22E-007
reg_DEFB	YES	chr8	7208781	A	G	63	16,00%	ambig	10				1,74E-006
reg_DEFB	YES	chr8	7208794	G	A	64	17,00%	ambig	11				2,30E-007
reg_DEFB	YES	chr8	7208816	C	A	70	17,00%	ambig	12				6,75E-008
reg_DEFB	YES	chr8	7208837	A	C	74	47,00%	het	35				1,00E-012
reg_DEFB	YES	chr8	7208881	C	T	88	42,00%	het	37				1,00E-012
reg_DEFB	YES	chr8	7208887	A	T	95	17,00%	ambig	16				6,05E-010
reg_DEFB	YES	chr8	7208898	C	T	101	32,00%	het	32				1,00E-012
reg_DEFB	YES	chr8	7208906	T	C	103	17,00%	ambig	18				3,44E-011
reg_DEFB	YES	chr8	7208911	A	T	103	11,00%	ambig	11				2,72E-005
reg_DEFB	YES	chr8	7208920	C	T	107	21,00%	ambig	22				1,00E-012
reg_DEFB	YES	chr8	7208925	A	T	108	19,00%	ambig	21				1,00E-012
reg_DEFB	YES	chr8	7208940	T	C	106	20,00%	ambig	21				1,00E-012
reg_DEFB	YES	chr8	7208951	T	G	106	40,00%	het	42				1,00E-012
reg_DEFB	YES	chr8	7208956	A	G	109	52,00%	het	57				1,00E-012
reg_DEFB	YES	chr8	7208958	A	T	110	54,00%	het	59				1,00E-012
reg_DEFB	YES	chr8	7208996	C	T	116	12,00%	ambig	14				5,19E-007
reg_DEFB	YES	chr8	7209021	A	G	133	24,00%	ambig	32				1,00E-012
reg_DEFB	YES	chr8	7209090	C	T	131	38,00%	het	50				1,00E-012
reg_DEFB	YES	chr8	7209103	T	C	127	26,00%	het	33				1,00E-012

reg_DEFB	YES	chr8	7209105	C	T	127	59,00%	het	75			1,00E-012
reg_DEFB	YES	chr8	7209110	G	A	127	39,00%	het	50			1,00E-012
reg_DEFB	YES	chr8	7209126	A	G	117	61,00%	het	71			1,00E-012
reg_DEFB	YES	chr8	7209183	A	G	102	13,00%	ambig	13			6,97E-007
reg_DEFB	YES	chr8	7209200	T	C	96	33,00%	het	32			1,00E-012
reg_DEFB	YES	chr8	7209211	A	G	92	11,00%	ambig	10			5,35E-005
reg_DEFB	YES	chr8	7209224	T	C	93	19,00%	ambig	18			1,51E-011
reg_DEFB	YES	chr8	7209238	A	G	87	20,00%	ambig	17			1,83E-011
reg_DEFB	YES	chr8	7209244	A	G	88	11,00%	ambig	10			3,64E-005
reg_DEFB	YES	chr8	7209275	C	A	75	12,00%	ambig	9			5,75E-005
reg_DEFB	YES	chr8	7209286	T	C	74	38,00%	het	28			1,00E-012
reg_DEFB	YES	chr8	7209380	A	C	59	15,00%	ambig	9			8,00E-006
reg_DEFB	YES	chr8	7209405	C	A	59	36,00%	het	21			1,00E-012
reg_DEFB	YES	chr8	7209417	T	G	60	13,00%	ambig	8			6,90E-005
reg_DEFB	YES	chr8	7209477	C	T	59	29,00%	het	17			1,00E-012
reg_DEFB	YES	chr8	7209495	G	C	58	69,00%	het	40	rs10107229		1,00E-012
reg_DEFB	YES	chr8	7209500	G	A	58	34,00%	het	20			1,00E-012
reg_DEFB	YES	chr8	7209543	C	G	58	34,00%	het	20			1,00E-012
reg_DEFB	YES	chr8	7209552	T	A	59	22,00%	ambig	13			7,64E-010
reg_DEFB	YES	chr8	7209570	T	A	67	12,00%	ambig	8			1,52E-004
reg_DEFB	YES	chr8	7209584	G	T	71	38,00%	het	27			1,00E-012
reg_DEFB	YES	chr8	7209625	T	C	81	17,00%	ambig	14			4,97E-009
reg_DEFB	YES	chr8	7209658	A	T	85	22,00%	ambig	19	rs12675743		1,00E-012
reg_DEFB	YES	chr8	7209708	A	G	100	36,00%	het	36			1,00E-012
reg_DEFB	YES	chr8	7209711	T	C	102	35,00%	het	36			1,00E-012
reg_DEFB	YES	chr8	7209802	C	A	118	20,00%	ambig	24			1,00E-012
reg_DEFB	YES	chr8	7209809	T	C	118	18,00%	ambig	21			1,00E-012
reg_DEFB	YES	chr8	7209834	T	G	123	48,00%	het	59			1,00E-012
reg_DEFB	YES	chr8	7209838	T	G	124	21,00%	ambig	26	rs10097913		1,00E-012
reg_DEFB	YES	chr8	7209849	A	G	124	30,00%	het	37			1,00E-012
reg_DEFB	YES	chr8	7209884	A	T	122	49,00%	het	60			1,00E-012
reg_DEFB	YES	chr8	7209889	A	G	122	43,00%	het	52			1,00E-012
reg_DEFB	YES	chr8	7209936	G	A	120	16,00%	ambig	19			4,87E-011
reg_DEFB	YES	chr8	7209960	A	G	110	15,00%	ambig	17			7,14E-010
reg_DEFB	YES	chr8	7209962	T	C	110	22,00%	ambig	24	rs12676217		1,00E-012
reg_DEFB	YES	chr8	7209970	C	A	108	14,00%	ambig	15			3,10E-008
reg_DEFB	YES	chr8	7210033	T	C	81	20,00%	ambig	16			5,95E-011
reg_DEFB	YES	chr8	7210112	A	T	52	65,00%	het	34	rs12681401		1,00E-012
reg_DEFB	YES	chr8	7210190	G	A	25	64,00%	het	16		rs71241132	1,00E-012
reg_DEFB	YES	chr8	7210229	A	G	15	40,00%	het	6			6,20E-007
reg_DEFB	YES	chr8	7210567	A	G	31	45,00%	het	14	rs9773734		1,00E-012
reg_DEFB	YES	chr8	7210635	T	A	36	33,00%	het	12	rs9774127		3,63E-011

reg_DEFB	YES	chr8	7210670	A	G	44	11,00%	ambig	5				3,31E-003
reg_DEFB	YES	chr8	7210673	C	T	45	11,00%	ambig	5				3,66E-003
reg_DEFB	YES	chr8	7210718	C	T	62	34,00%	het	21	rs9772086			1,00E-012
reg_DEFB	YES	chr8	7210734	A	C	70	23,00%	ambig	16				1,34E-011
reg_DEFB	YES	chr8	7210769	T	C	75	21,00%	ambig	16				1,68E-011
reg_DEFB	YES	chr8	7210821	A	G	81	20,00%	ambig	16				5,95E-011
reg_DEFB	YES	chr8	7210836	G	C	85	20,00%	ambig	17				1,22E-011
reg_DEFB	YES	chr8	7210874	C	T	90	12,00%	ambig	11				7,44E-006
reg_DEFB	YES	chr8	7210889	C	T	92	12,00%	ambig	11				9,22E-006
reg_DEFB	YES	chr8	7210912	C	A	97	29,00%	het	28				1,00E-012
reg_DEFB	YES	chr8	7210974	G	C	105	28,00%	het	29			rs55975306	1,00E-012
reg_DEFB	YES	chr8	7210988	G	C	103	19,00%	ambig	20	rs3989690			1,00E-012
reg_DEFB	YES	chr8	7211001	A	T	104	12,00%	ambig	12				5,33E-006
reg_DEFB	YES	chr8	7211096	A	G	70	10,00%	ambig	7				1,15E-003
reg_DEFB	YES	chr8	7211198	C	G	59	27,00%	het	16	rs3989724			1,00E-012
reg_DEFB	YES	chr8	7211286	C	T	49	41,00%	het	20	rs3989723			1,00E-012
reg_DEFB	YES	chr8	7211302	G	T	48	29,00%	het	14	rs3989722			6,85E-012
reg_DEFB	YES	chr8	7211344	G	A	55	33,00%	het	18	rs3989721			1,00E-012
reg_DEFB	YES	chr8	7211388	G	C	56	36,00%	het	20	rs3989720			1,00E-012
reg_DEFB	YES	chr8	7211474	G	T	57	26,00%	het	15				6,21E-012
reg_DEFB	YES	chr8	7211483	C	T	56	34,00%	het	19	rs3958830	rs10094171		1,00E-012
reg_DEFB	YES	chr8	7211500	A	G	52	23,00%	ambig	12	rs3989719			1,94E-009
reg_DEFB	YES	chr8	7211502	G	T	53	21,00%	ambig	11	rs3989718			2,98E-008
reg_DEFB	YES	chr8	7211511	T	C	53	21,00%	ambig	11				2,98E-008
reg_DEFB	YES	chr8	7211524	G	A	53	19,00%	ambig	10	rs3989717			3,27E-007
reg_DEFB	YES	chr8	7211532	C	T	50	36,00%	het	18	rs9720553			1,00E-012
reg_DEFB	YES	chr8	7211592	G	A	43	14,00%	ambig	6				4,35E-004
reg_DEFB	YES	chr8	7211652	C	A	40	10,00%	ambig	4				1,32E-002
reg_DEFB	YES	chr8	7211679	G	A	38	34,00%	het	13	rs9314626			3,69E-012
reg_DEFB	YES	chr8	7211764	C	T	44	32,00%	het	14	rs9314627			1,00E-012
reg_DEFB	YES	chr8	7211776	A	G	44	32,00%	het	14	rs9314628			1,00E-012
reg_DEFB	YES	chr8	7212059	T	C	50	32,00%	het	16	rs11984912			1,00E-012
reg_DEFB	YES	chr8	7212151	G	A	55	15,00%	ambig	8				3,63E-005
reg_DEFB	YES	chr8	7212189	C	T	52	35,00%	het	18	rs11994800			1,00E-012
reg_DEFB	YES	chr8	7212213	T	C	53	19,00%	ambig	10				3,27E-007
reg_DEFB	YES	chr8	7212223	A	G	52	25,00%	het	13				1,52E-010
reg_DEFB	YES	chr8	7212234	T	G	55	27,00%	het	15				3,47E-012
reg_DEFB	YES	chr8	7212276	G	A	61	15,00%	ambig	9				1,06E-005
reg_DEFB	YES	chr8	7212354	G	A	57	14,00%	ambig	8				4,73E-005
reg_DEFB	YES	chr8	7212402	G	A	52	31,00%	het	16				1,00E-012
reg_DEFB	YES	chr8	7212441	C	G	62	11,00%	ambig	7				5,53E-004
reg_DEFB	YES	chr8	7212461	A	C	70	37,00%	het	26				1,00E-012

reg_DEFB	YES	chr8	7212493	G	C	70	11,00%	ambig	8			2,08E-004
reg_DEFB	YES	chr8	7212506	C	T	75	48,00%	het	36	rs9720735		1,00E-012
reg_DEFB	YES	chr8	7212526	C	A	79	65,00%	het	51	rs9720736		1,00E-012
reg_DEFB	YES	chr8	7212533	C	T	77	47,00%	het	36			1,00E-012
reg_DEFB	YES	chr8	7212541	A	T	77	13,00%	ambig	10			1,11E-005
reg_DEFB	YES	chr8	7212544	A	C	78	40,00%	het	31			1,00E-012
reg_DEFB	YES	chr8	7212552	A	T	81	48,00%	het	39			1,00E-012
reg_DEFB	YES	chr8	7212572	G	C	85	21,00%	ambig	18			2,97E-012
reg_DEFB	YES	chr8	7212694	C	T	77	48,00%	het	37		rs55761444	1,00E-012
reg_DEFB	YES	chr8	7212712	A	G	83	39,00%	het	32			1,00E-012
reg_DEFB	YES	chr8	7212720	C	T	84	27,00%	het	23	rs9720745		1,00E-012
reg_DEFB	YES	chr8	7212726	G	A	86	47,00%	het	40			1,00E-012
reg_DEFB	YES	chr8	7212732	A	G	88	18,00%	ambig	16			1,88E-010
reg_DEFB	YES	chr8	7212764	G	T	96	17,00%	ambig	16			7,11E-010
reg_DEFB	YES	chr8	7212782	C	A	101	45,00%	het	45			1,00E-012
reg_DEFB	YES	chr8	7212873	A	G	96	19,00%	ambig	18			9,81E-012
reg_DEFB	YES	chr8	7212877	A	G	93	42,00%	het	39			1,00E-012
reg_DEFB	YES	chr8	7212889	T	G	93	23,00%	ambig	21			1,00E-012
reg_DEFB	YES	chr8	7212929	C	T	80	19,00%	ambig	15			4,38E-010
reg_DEFB	YES	chr8	7212969	G	C	89	42,00%	het	37			1,00E-012
reg_DEFB	YES	chr8	7212983	C	T	94	24,00%	ambig	23			1,00E-012
reg_DEFB	YES	chr8	7213048	G	T	97	39,00%	het	38			1,00E-012
reg_DEFB	YES	chr8	7213076	C	T	102	15,00%	ambig	15			1,41E-008
reg_DEFB	YES	chr8	7213087	C	T	105	16,00%	ambig	17			3,47E-010
reg_DEFB	YES	chr8	7213098	T	C	102	25,00%	het	26	rs9720878		1,00E-012
reg_DEFB	YES	chr8	7213114	A	C	105	27,00%	het	28			1,00E-012
reg_DEFB	YES	chr8	7213137	A	G	103	26,00%	het	27			1,00E-012
reg_DEFB	YES	chr8	7213139	C	T	102	48,00%	het	49			1,00E-012
reg_DEFB	YES	chr8	7213178	T	C	91	22,00%	ambig	20			1,00E-012
reg_DEFB	YES	chr8	7213182	G	T	89	11,00%	ambig	10			4,02E-005
reg_DEFB	YES	chr8	7213215	G	T	84	20,00%	ambig	17			9,95E-012
reg_DEFB	YES	chr8	7213222	A	G	86	30,00%	het	26			1,00E-012
reg_DEFB	YES	chr8	7213227	C	G	86	28,00%	het	24			1,00E-012
reg_DEFB	YES	chr8	7213245	A	T	83	41,00%	het	34			1,00E-012
reg_DEFB	YES	chr8	7213289	G	C	84	20,00%	ambig	17	rs41394946		9,95E-012
reg_DEFB	YES	chr8	7213305	A	G	81	42,00%	het	34			1,00E-012
reg_DEFB	YES	chr8	7213332	C	A	77	42,00%	het	32			1,00E-012
reg_DEFB	YES	chr8	7213393	C	T	70	44,00%	het	31			1,00E-012
reg_DEFB	YES	chr8	7213423	G	C	69	28,00%	het	19			1,00E-012
reg_DEFB	YES	chr8	7213445	C	T	62	68,00%	het	42	rs3958829		1,00E-012
reg_DEFB	YES	chr8	7213470	A	G	57	68,00%	het	39	rs9314629		1,00E-012
reg_DEFB	YES	chr8	7213488	T	C	53	23,00%	ambig	12			2,43E-009

reg_DEFB	YES	chr8	7213560	G	A	44	25,00%	het	11			3,64E-009
reg_DEFB	YES	chr8	7213575	A	C	45	42,00%	het	19			1,00E-012
reg_DEFB	YES	chr8	7213603	T	C	44	39,00%	het	17			1,00E-012
reg_DEFB	YES	chr8	7213631	T	C	44	34,00%	het	15			1,00E-012
reg_DEFB	YES	chr8	7213665	C	G	45	36,00%	het	16			1,00E-012
reg_DEFB	YES	chr8	7213666	A	G	45	27,00%	het	12			3,12E-010
reg_DEFB	YES	chr8	7213674	A	G	44	43,00%	het	19			1,00E-012
reg_DEFB	YES	chr8	7213693	A	G	42	48,00%	het	20			1,00E-012
reg_DEFB	YES	chr8	7213713	A	T	45	51,00%	het	23			1,00E-012
reg_DEFB	YES	chr8	7213766	T	A	49	61,00%	het	30			1,00E-012
reg_DEFB	YES	chr8	7213778	C	T	52	27,00%	het	14			2,32E-011
reg_DEFB	YES	chr8	7213785	G	A	50	16,00%	ambig	8			1,77E-005
reg_DEFB	YES	chr8	7213790	T	C	50	34,00%	het	17			1,00E-012
reg_DEFB	YES	chr8	7213820	A	G	52	63,00%	het	33			1,00E-012
reg_DEFB	YES	chr8	7213824	A	C	52	15,00%	ambig	8			2,39E-005
reg_DEFB	YES	chr8	7213831	C	T	54	19,00%	ambig	10			3,93E-007
reg_DEFB	YES	chr8	7213836	G	A	55	18,00%	ambig	10			4,70E-007
reg_DEFB	YES	chr8	7213845	G	A	59	61,00%	het	36			1,00E-012
reg_DEFB	YES	chr8	7213856	A	G	64	31,00%	het	20			1,00E-012
reg_DEFB	YES	chr8	7213870	G	C	63	29,00%	het	18			1,00E-012
reg_DEFB	YES	chr8	7213871	G	A	64	64,00%	het	41		rs1456304	1,00E-012
reg_DEFB	YES	chr8	7213935	C	T	62	29,00%	het	18			1,00E-012
reg_DEFB	YES	chr8	7213953	C	G	63	25,00%	het	16			2,20E-012
reg_DEFB	YES	chr8	7214009	C	T	70	16,00%	ambig	11			5,90E-007
reg_DEFB	YES	chr8	7214022	A	T	69	12,00%	ambig	8			1,88E-004
reg_DEFB	YES	chr8	7214034	C	G	66	71,00%	het	47			1,00E-012
reg_DEFB	YES	chr8	7214038	T	G	68	10,00%	ambig	7			9,68E-004
reg_DEFB	YES	chr8	7214039	C	T	68	28,00%	het	19		rs6997873	1,00E-012
reg_DEFB	YES	chr8	7214040	G	A	68	13,00%	ambig	9			2,61E-005
reg_DEFB	YES	chr8	7214050	T	C	69	33,00%	het	23			1,00E-012
reg_DEFB	YES	chr8	7214061	G	A	66	12,00%	ambig	8			1,37E-004
reg_DEFB	YES	chr8	7214102	G	A	65	17,00%	ambig	11			2,71E-007
reg_DEFB	YES	chr8	7214113	A	C	63	54,00%	het	34	rs3989716		1,00E-012
reg_DEFB	YES	chr8	7214129	G	A	64	19,00%	ambig	12		rs35534613	2,37E-008
reg_DEFB	YES	chr8	7214143	A	C	65	71,00%	het	46		rs62527852	1,00E-012
reg_DEFB	YES	chr8	7214164	T	C	65	52,00%	het	34	rs9693481		1,00E-012
reg_DEFB	YES	chr8	7214177	C	A	66	27,00%	het	18			1,00E-012
reg_DEFB	YES	chr8	7214179	A	G	65	22,00%	ambig	14			2,37E-010
reg_DEFB	YES	chr8	7214198	T	C	65	46,00%	het	30			1,00E-012
reg_DEFB	YES	chr8	7214199	G	A	65	23,00%	ambig	15			2,10E-011
reg_DEFB	YES	chr8	7214207	T	C	63	25,00%	het	16			2,20E-012
reg_DEFB	YES	chr8	7214212	A	G	63	22,00%	ambig	14			1,51E-010

reg_DEFB	YES	chr8	7214223	G	A	66	64,00%	het	42			1,00E-012
reg_DEFB	YES	chr8	7214240	A	G	63	75,00%	het	47	rs3989715		1,00E-012
reg_DEFB	YES	chr8	7214272	A	G	62	65,00%	het	40			1,00E-012
reg_DEFB	YES	chr8	7214289	G	A	60	20,00%	ambig	12			1,10E-008
reg_DEFB	YES	chr8	7214296	G	T	58	64,00%	het	37			1,00E-012
reg_DEFB	YES	chr8	7214308	C	G	56	61,00%	het	34		rs57788671	1,00E-012
reg_DEFB	YES	chr8	7214315	A	G	54	65,00%	het	35			1,00E-012
reg_DEFB	YES	chr8	7214316	C	T	53	28,00%	het	15			1,89E-012
reg_DEFB	YES	chr8	7214344	C	T	57	16,00%	ambig	9			5,97E-006
reg_DEFB	YES	chr8	7214351	G	C	58	29,00%	het	17			1,00E-012
reg_DEFB	YES	chr8	7214371	C	A	56	25,00%	het	14		rs60424581	3,00E-011
reg_DEFB	YES	chr8	7214375	C	T	58	16,00%	ambig	9	rs3989714		6,92E-006
reg_DEFB	YES	chr8	7214386	G	T	56	46,00%	het	26	rs3958828		1,00E-012
reg_DEFB	YES	chr8	7214405	T	A	56	71,00%	het	40			1,00E-012
reg_DEFB	YES	chr8	7214458	A	T	54	20,00%	ambig	11			3,66E-008
reg_DEFB	YES	chr8	7214483	G	A	50	14,00%	ambig	7			1,43E-004
reg_DEFB	YES	chr8	7214498	T	C	50	14,00%	ambig	7		rs72669900	1,43E-004
reg_DEFB	YES	chr8	7214519	T	C	55	40,00%	het	22			1,00E-012
reg_DEFB	YES	chr8	7214555	A	G	50	32,00%	het	16			1,00E-012
reg_DEFB	YES	chr8	7214557	T	G	51	14,00%	ambig	7		rs73198172	1,62E-004
reg_DEFB	YES	chr8	7214578	C	T	53	28,00%	het	15		rs12548551	1,89E-012
reg_DEFB	YES	chr8	7214598	C	G	54	31,00%	het	17			1,00E-012
reg_DEFB	YES	chr8	7214642	C	T	55	27,00%	het	15		rs72646358	3,47E-012
reg_DEFB	YES	chr8	7214643	A	G	55	27,00%	het	15			3,47E-012
reg_DEFB	YES	chr8	7214648	C	T	53	25,00%	het	13			1,80E-010
reg_DEFB	YES	chr8	7214649	A	G	53	23,00%	ambig	12			2,43E-009
reg_DEFB	YES	chr8	7214679	G	A	52	46,00%	het	24	rs3989713		1,00E-012
reg_DEFB	YES	chr8	7214684	C	A	51	24,00%	ambig	12			1,52E-009
reg_DEFB	YES	chr8	7214746	G	A	53	23,00%	ambig	12			2,43E-009
reg_DEFB	YES	chr8	7214782	C	A	52	21,00%	ambig	11			2,41E-008
reg_DEFB	YES	chr8	7214792	A	G	51	24,00%	ambig	12	rs3989712		1,52E-009
reg_DEFB	YES	chr8	7214844	G	A	41	15,00%	ambig	6			3,33E-004
reg_DEFB	YES	chr8	7214929	G	A	30	33,00%	het	10			8,17E-010
reg_DEFB	YES	chr8	7214947	A	G	27	30,00%	het	8	rs3989711		1,18E-007
reg_DEFB	YES	chr8	7215572	C	T	16	94,00%	homvar	15	rs9774404		1,00E-012
reg_DEFB	YES	chr8	7215746	G	A	18	89,00%	homvar	16	rs4840274		1,00E-012
reg_DEFB	YES	chr8	7215791	A	T	22	91,00%	homvar	20		rs71532134	1,00E-012
reg_DEFB	YES	chr8	7216165	G	T	15	73,00%	het	11	rs2719560		1,00E-012
reg_DEFB	YES	chr8	7216177	A	G	14	86,00%	homvar	12	rs2698852		1,00E-012
reg_DEFB	YES	chr8	7216193	C	A	15	87,00%	homvar	13		rs2739920	1,00E-012
reg_DEFB	YES	chr8	7216236	G	T	15	67,00%	het	10		rs2740655	1,00E-012
reg_DEFB	YES	chr8	7216359	T	C	17	24,00%	ambig	4			5,24E-004

reg_DEFB	YES	chr8	7216399	C	T	23	22,00%	ambig	5			1,53E-004
reg_DEFB	YES	chr8	7216407	C	G	24	21,00%	ambig	5			1,90E-004
reg_DEFB	YES	chr8	7216462	T	A	30	63,00%	het	19		rs62636820	1,00E-012
reg_DEFB	YES	chr8	7216464	G	T	30	27,00%	het	8			2,91E-007
reg_DEFB	YES	chr8	7216514	G	A	38	21,00%	ambig	8			2,07E-006
reg_DEFB	YES	chr8	7216559	A	C	45	11,00%	ambig	5	rs2739917		3,66E-003
reg_DEFB	YES	chr8	7216565	A	C	46	59,00%	het	27	rs2739916		1,00E-012
reg_DEFB	YES	chr8	7216579	G	A	50	20,00%	ambig	10			1,83E-007
reg_DEFB	YES	chr8	7216612	A	T	53	19,00%	ambig	10			3,27E-007
reg_DEFB	YES	chr8	7216647	T	C	54	22,00%	ambig	12			3,06E-009
reg_DEFB	YES	chr8	7216666	A	T	56	29,00%	het	16		rs2461004	1,00E-012
reg_DEFB	YES	chr8	7216727	T	C	70	21,00%	ambig	15			6,69E-011
reg_DEFB	YES	chr8	7216730	C	A	71	21,00%	ambig	15			8,33E-011
reg_DEFB	YES	chr8	7216732	C	G	71	21,00%	ambig	15			8,33E-011
reg_DEFB	YES	chr8	7216748	T	A	70	21,00%	ambig	15			6,69E-011
reg_DEFB	YES	chr8	7216765	A	G	70	20,00%	ambig	14			6,76E-010
reg_DEFB	YES	chr8	7216794	C	T	77	10,00%	ambig	8			4,02E-004
reg_DEFB	YES	chr8	7216859	T	C	90	19,00%	ambig	17			3,25E-011
reg_DEFB	YES	chr8	7217037	C	A	99	20,00%	ambig	20	rs3958825		1,00E-012
reg_DEFB	YES	chr8	7217057	T	C	94	21,00%	ambig	20			1,00E-012
reg_DEFB	YES	chr8	7217109	G	C	88	70,00%	het	62	rs2740653		1,00E-012
reg_DEFB	YES	chr8	7217116	G	A	92	26,00%	het	24			1,00E-012
reg_DEFB	YES	chr8	7217208	A	G	74	23,00%	ambig	17			2,76E-012
reg_DEFB	YES	chr8	7217227	T	C	76	21,00%	ambig	16			2,09E-011
reg_DEFB	YES	chr8	7217283	T	C	77	13,00%	ambig	10			1,11E-005
reg_DEFB	YES	chr8	7217293	C	T	75	12,00%	ambig	9			5,75E-005
reg_DEFB	YES	chr8	7217310	A	G	74	82,00%	homvar	61	rs2739913		1,00E-012
reg_DEFB	YES	chr8	7217386	C	T	85	13,00%	ambig	11			4,24E-006
reg_DEFB	YES	chr8	7217396	A	G	85	21,00%	ambig	18			2,97E-012
reg_DEFB	YES	chr8	7217412	G	A	90	37,00%	het	33	rs2739912		1,00E-012
reg_DEFB	YES	chr8	7217505	G	T	117	22,00%	ambig	26			1,00E-012
reg_DEFB	YES	chr8	7217535	T	C	132	14,00%	ambig	18			1,83E-009
reg_DEFB	YES	chr8	7217540	A	G	135	15,00%	ambig	20			5,35E-011
reg_DEFB	YES	chr8	7217544	T	A	133	59,00%	het	78	rs2740651		1,00E-012
reg_DEFB	YES	chr8	7217623	G	C	124	27,00%	het	33			1,00E-012
reg_DEFB	YES	chr8	7217638	C	A	124	17,00%	ambig	21			4,53E-012
reg_DEFB	YES	chr8	7217667	T	A	124	16,00%	ambig	20			1,37E-011
reg_DEFB	YES	chr8	7217831	C	A	105	10,00%	ambig	11			3,25E-005
reg_DEFB	YES	chr8	7217892	T	C	102	13,00%	ambig	13			6,97E-007
reg_DEFB	YES	chr8	7217922	G	A	100	11,00%	ambig	11			2,05E-005
reg_DEFB	YES	chr8	7217957	G	T	97	10,00%	ambig	10			8,42E-005
reg_DEFB	YES	chr8	7218052	C	A	80	10,00%	ambig	8			5,21E-004

reg_DEFB	YES	chr8	7218094	T	C	76	11,00%	ambig	8			3,67E-004
reg_DEFB	YES	chr8	7218187	G	A	60	97,00%	homvar	58	rs3877968		1,00E-012
reg_DEFB	YES	chr8	7218825	G	T	25	96,00%	homvar	24	rs2719558		1,00E-012
reg_DEFB	YES	chr8	7218845	T	C	24	96,00%	homvar	23			1,00E-012
reg_DEFB	YES	chr8	7218861	A	G	17	76,00%	homvar	13		rs2719557	1,00E-012
reg_DEFB	YES	chr8	7218900	C	T	18	94,00%	homvar	17		rs2739910	1,00E-012
reg_DEFB	YES	chr8	7218909	A	C	16	31,00%	het	5			2,27E-005
reg_DEFB	YES	chr8	7218970	C	G	13	85,00%	homvar	11		rs2951096	1,00E-012
reg_DEFB	YES	chr8	7218973	T	C	13	77,00%	homvar	10		rs2954054	1,00E-012
reg_DEFB	YES	chr8	7218979	C	T	14	43,00%	het	6			3,79E-007
reg_DEFB	YES	chr8	7219007	C	T	15	53,00%	het	8			4,39E-010
reg_DEFB	YES	chr8	7219008	A	G	15	93,00%	homvar	14			1,00E-012
reg_DEFB	YES	chr8	7219050	A	G	22	36,00%	het	8			1,88E-008
reg_DEFB	YES	chr8	7219065	T	C	24	37,00%	het	9			1,72E-009
reg_DEFB	YES	chr8	7219076	A	G	26	35,00%	het	9			3,95E-009
reg_DEFB	YES	chr8	7219078	T	G	26	88,00%	homvar	23			1,00E-012
reg_DEFB	YES	chr8	7219083	T	C	27	37,00%	het	10			2,51E-010
reg_DEFB	YES	chr8	7219176	C	T	41	44,00%	het	18			1,00E-012
reg_DEFB	YES	chr8	7219186	T	G	41	44,00%	het	18			1,00E-012
reg_DEFB	YES	chr8	7219191	A	G	41	51,00%	het	21		rs71509104	1,00E-012
reg_DEFB	YES	chr8	7219218	T	C	44	89,00%	homvar	39			1,00E-012
reg_DEFB	YES	chr8	7219236	G	C	43	47,00%	het	20			1,00E-012
reg_DEFB	YES	chr8	7219251	T	C	46	35,00%	het	16			1,00E-012
reg_DEFB	YES	chr8	7219280	T	C	45	47,00%	het	21			1,00E-012
reg_DEFB	YES	chr8	7219346	C	A	43	40,00%	het	17			1,00E-012
reg_DEFB	YES	chr8	7219348	C	A	43	79,00%	homvar	34			1,00E-012
reg_DEFB	YES	chr8	7219375	T	C	45	80,00%	homvar	36			1,00E-012
reg_DEFB	YES	chr8	7219429	G	T	39	82,00%	homvar	32			1,00E-012
reg_DEFB	YES	chr8	7219459	C	A	34	79,00%	homvar	27			1,00E-012
reg_DEFB	YES	chr8	7219479	A	G	30	53,00%	het	16			1,00E-012
reg_DEFB	YES	chr8	7219497	C	G	27	81,00%	homvar	22			1,00E-012
reg_DEFB	YES	chr8	7219501	A	T	26	58,00%	het	15			1,00E-012
reg_DEFB	YES	chr8	7219515	T	C	27	19,00%	ambig	5			3,40E-004
reg_DEFB	YES	chr8	7219548	C	A	24	54,00%	het	13			1,00E-012
reg_DEFB	YES	chr8	7219555	G	A	23	61,00%	het	14			1,00E-012
reg_DEFB	YES	chr8	7219557	C	A	23	17,00%	ambig	4			1,75E-003
reg_DEFB	YES	chr8	7219581	G	A	22	77,00%	homvar	17			1,00E-012
reg_DEFB	YES	chr8	7219618	C	T	20	95,00%	homvar	19	rs34236860		1,00E-012
reg_DEFB	YES	chr8	7219641	G	A	20	75,00%	het	15			1,00E-012
reg_DEFB	YES	chr8	7219667	T	C	20	85,00%	homvar	17			1,00E-012
reg_DEFB	YES	chr8	7219722	T	C	18	83,00%	homvar	15			1,00E-012
reg_DEFB	YES	chr8	7219728	A	T	17	82,00%	homvar	14			1,00E-012

reg_DEFB	YES	chr8	7219739	G	A	19	84,00%	homvar	16				1,00E-012
reg_DEFB	YES	chr8	7219745	T	A	19	84,00%	homvar	16				1,00E-012
reg_DEFB	YES	chr8	7219750	A	G	19	84,00%	homvar	16				1,00E-012
reg_DEFB	YES	chr8	7219778	T	G	19	95,00%	homvar	18				1,00E-012
reg_DEFB	YES	chr8	7219968	C	T	17	94,00%	homvar	16				1,00E-012
reg_DEFB	YES	chr8	7219996	T	C	18	72,00%	het	13				1,00E-012
reg_DEFB	YES	chr8	7220067	C	T	17	53,00%	het	9				5,40E-011
reg_DEFB	YES	chr8	7220069	G	A	17	53,00%	het	9				5,40E-011
reg_DEFB	YES	chr8	7220079	G	A	18	94,00%	homvar	17			rs34293419	1,00E-012
reg_DEFB	YES	chr8	7220082	C	T	18	50,00%	het	9				7,33E-011
reg_DEFB	YES	chr8	7220087	A	G	18	89,00%	homvar	16				1,00E-012
reg_DEFB	YES	chr8	7220114	C	T	18	39,00%	het	7				8,67E-008
reg_DEFB	YES	chr8	7220132	A	T	18	61,00%	het	11			rs62493693	1,00E-012
reg_DEFB	YES	chr8	7220184	T	C	20	75,00%	het	15				1,00E-012
reg_DEFB	YES	chr8	7220193	T	C	19	26,00%	het	5				5,71E-005
reg_DEFB	YES	chr8	7220216	T	G	19	89,00%	homvar	17			rs2139763	1,00E-012
reg_DEFB	YES	chr8	7220227	T	C	21	90,00%	homvar	19			rs2139762	1,00E-012
reg_DEFB	YES	chr8	7220232	C	A	22	59,00%	het	13				1,00E-012
reg_DEFB	YES	chr8	7220265	C	T	28	96,00%	homvar	27	rs34967312			1,00E-012
reg_DEFB	YES	chr8	7220273	G	A	28	57,00%	het	16				1,00E-012
reg_DEFB	YES	chr8	7220281	G	A	28	43,00%	het	12			rs62483102	1,00E-012
reg_DEFB	YES	chr8	7220288	A	G	32	59,00%	het	19				1,00E-012
reg_DEFB	YES	chr8	7220306	A	C	31	58,00%	het	18				1,00E-012
reg_DEFB	YES	chr8	7220320	G	A	31	58,00%	het	18				1,00E-012
reg_DEFB	YES	chr8	7220342	T	G	35	63,00%	het	22				1,00E-012
reg_DEFB	YES	chr8	7220395	A	G	45	22,00%	ambig	10				6,32E-008
reg_DEFB	YES	chr8	7220424	G	A	39	77,00%	homvar	30				1,00E-012
reg_DEFB	YES	chr8	7220499	T	G	37	86,00%	homvar	32	rs34788125			1,00E-012
reg_DEFB	YES	chr8	7220520	C	T	34	91,00%	homvar	31				1,00E-012
reg_DEFB	YES	chr8	7220593	C	G	46	93,00%	homvar	43				1,00E-012
reg_DEFB	YES	chr8	7220646	T	C	50	88,00%	homvar	44				1,00E-012
reg_DEFB	YES	chr8	7220701	C	T	48	10,00%	ambig	5				4,85E-003
reg_DEFB	YES	chr8	7220797	G	A	47	74,00%	het	35				1,00E-012
reg_DEFB	YES	chr8	7220811	A	G	50	92,00%	homvar	46				1,00E-012
reg_DEFB	YES	chr8	7220813	A	G	50	20,00%	ambig	10				1,83E-007
reg_DEFB	YES	chr8	7220891	A	T	41	29,00%	het	12				1,00E-010
reg_DEFB	YES	chr8	7220980	T	C	34	97,00%	homvar	33	rs4620307	rs36048429		1,00E-012
reg_DEFB	YES	chr8	7220984	G	A	34	12,00%	ambig	4	rs34205780			7,48E-003
reg_DEFB	YES	chr8	7221028	G	A	24	46,00%	het	11				3,07E-012
reg_DEFB	YES	chr8	7221049	A	T	24	46,00%	het	11				3,07E-012
reg_DEFB	YES	chr8	7221052	T	C	24	46,00%	het	11				3,07E-012
reg_DEFB	YES	chr8	7221062	C	T	23	26,00%	het	6				1,07E-005

reg_DEFB	YES	chr8	7221068	G	A	23	26,00%	het	6				1,07E-005
reg_DEFB	YES	chr8	7221069	T	C	23	26,00%	het	6				1,07E-005
reg_DEFB	YES	chr8	7221074	A	C	23	43,00%	het	10				3,67E-011
reg_DEFB	YES	chr8	7221076	T	C	23	22,00%	ambig	5				1,53E-004
reg_DEFB	YES	chr8	7221077	T	G	23	39,00%	het	9				1,10E-009
reg_DEFB	YES	chr8	7221079	C	G	23	26,00%	het	6				1,07E-005
reg_DEFB	YES	chr8	7221085	C	T	23	22,00%	ambig	5				1,53E-004
reg_DEFB	YES	chr8	7221088	T	C	23	39,00%	het	9				1,10E-009
reg_DEFB	YES	chr8	7221097	G	A	23	22,00%	ambig	5				1,53E-004
reg_DEFB	YES	chr8	7221131	C	T	19	68,00%	het	13				1,00E-012
reg_DEFB	YES	chr8	7221141	T	C	18	33,00%	het	6				2,17E-006
reg_DEFB	YES	chr8	7221147	G	A	17	18,00%	ambig	3				6,50E-003
reg_DEFB	YES	chr8	7221196	T	C	13	62,00%	het	8			rs2739907	9,13E-011
reg_DEFB	YES	chr8	7221235	G	T	12	58,00%	het	7				2,45E-009
reg_DEFB	YES	chr8	7221273	C	T	15	100,00%	homvar	15	rs35737531			1,00E-012
reg_DEFB	YES	chr8	7221351	C	T	38	55,00%	het	21			rs2463975	1,00E-012
reg_DEFB	YES	chr8	7221359	C	T	43	88,00%	homvar	38			rs2463974	1,00E-012
reg_DEFB	YES	chr8	7221372	A	G	49	63,00%	het	31	rs2466108			1,00E-012
reg_DEFB	YES	chr8	7221417	T	A	59	68,00%	het	40			rs3867441	1,00E-012
reg_DEFB	YES	chr8	7221447	A	T	69	20,00%	ambig	14				5,52E-010
reg_DEFB	YES	chr8	7221470	G	A	73	93,00%	homvar	68	rs2739906	rs34051729		1,00E-012
reg_DEFB	YES	chr8	7221507	G	A	76	75,00%	het	57	rs4118276			1,00E-012
reg_DEFB	YES	chr8	7221622	G	A	85	24,00%	ambig	20	rs2719550			1,00E-012
reg_DEFB	YES	chr8	7221627	G	A	83	18,00%	ambig	15				7,41E-010
reg_DEFB	YES	chr8	7221643	G	T	81	17,00%	ambig	14				4,97E-009
reg_DEFB	YES	chr8	7221650	C	A	81	15,00%	ambig	12				3,55E-007
reg_DEFB	YES	chr8	7221656	A	C	80	20,00%	ambig	16				4,86E-011
reg_DEFB	YES	chr8	7221673	A	G	81	25,00%	het	20	rs2719549	rs2698890		1,00E-012
reg_DEFB	YES	chr8	7221734	G	A	78	14,00%	ambig	11				1,79E-006
reg_DEFB	YES	chr8	7222236	A	G	46	11,00%	ambig	5				4,03E-003
reg_DEFB	YES	chr8	7222261	G	T	47	11,00%	ambig	5				4,42E-003
reg_DEFB	YES	chr8	7222272	A	G	44	11,00%	ambig	5				3,31E-003
reg_DEFB	YES	chr8	7222289	A	G	44	11,00%	ambig	5				3,31E-003
reg_DEFB	YES	chr8	7222307	G	A	41	78,00%	homvar	32	rs2739903			1,00E-012
reg_DEFB	YES	chr8	7222344	A	T	41	12,00%	ambig	5				2,42E-003
reg_DEFB	YES	chr8	7222348	A	T	42	14,00%	ambig	6	rs2719547			3,81E-004
reg_DEFB	YES	chr8	7222419	C	A	38	68,00%	het	26	rs2739902			1,00E-012
reg_DEFB	YES	chr8	7222458	G	T	31	29,00%	het	9				2,29E-008
reg_DEFB	YES	chr8	7222484	C	T	32	25,00%	het	8				5,03E-007
reg_DEFB	YES	chr8	7222502	G	T	30	90,00%	homvar	27	rs4118271			1,00E-012
reg_DEFB	YES	chr8	7222663	G	C	18	61,00%	het	11	rs2698889			1,00E-012
reg_DEFB	YES	chr8	7222843	A	G	14	100,00%	homvar	14			rs73356401	1,00E-012

reg_DEFB	YES	chr8	7222958	C	T	22	23,00%	ambig	5			1,22E-004
reg_DEFB	YES	chr8	7222997	A	C	30	23,00%	ambig	7			4,35E-006
reg_DEFB	YES	chr8	7223022	A	C	39	15,00%	ambig	6			2,52E-004
reg_DEFB	YES	chr8	7223030	C	T	39	26,00%	het	10			1,43E-008
reg_DEFB	YES	chr8	7223044	A	C	44	16,00%	ambig	7			6,18E-005
reg_DEFB	YES	chr8	7223079	C	A	53	13,00%	ambig	7			2,08E-004
reg_DEFB	YES	chr8	7223085	C	G	54	15,00%	ambig	8			3,17E-005
reg_DEFB	YES	chr8	7223090	T	C	54	15,00%	ambig	8			3,17E-005
reg_DEFB	YES	chr8	7223096	A	G	56	29,00%	het	16			1,00E-012
reg_DEFB	YES	chr8	7223106	G	A	59	19,00%	ambig	11			9,63E-008
reg_DEFB	YES	chr8	7223131	G	A	71	28,00%	het	20	rs2719542		1,00E-012
reg_DEFB	YES	chr8	7223176	G	A	87	16,00%	ambig	14			1,29E-008
reg_DEFB	YES	chr8	7223186	G	A	89	13,00%	ambig	12			1,01E-006
reg_DEFB	YES	chr8	7223191	C	A	90	13,00%	ambig	12			1,14E-006
reg_DEFB	YES	chr8	7223226	T	G	101	11,00%	ambig	11			2,26E-005
reg_DEFB	YES	chr8	7223235	G	T	102	13,00%	ambig	13			6,97E-007
reg_DEFB	YES	chr8	7223250	T	C	100	23,00%	ambig	23	rs2719541		1,00E-012
reg_DEFB	YES	chr8	7223255	G	C	102	15,00%	ambig	15			1,41E-008
reg_DEFB	YES	chr8	7223264	T	C	102	14,00%	ambig	14			1,03E-007
reg_DEFB	YES	chr8	7223266	G	A	104	21,00%	ambig	22			1,00E-012
reg_DEFB	YES	chr8	7223379	T	C	108	19,00%	ambig	21	rs2719540		1,00E-012
reg_DEFB	YES	chr8	7223384	T	C	108	19,00%	ambig	21			1,00E-012
reg_DEFB	YES	chr8	7223402	C	G	110	20,00%	ambig	22			1,00E-012
reg_DEFB	YES	chr8	7223407	A	C	112	16,00%	ambig	18			1,20E-010
reg_DEFB	YES	chr8	7223587	C	G	99	12,00%	ambig	12			3,17E-006
reg_DEFB	YES	chr8	7223593	G	T	99	17,00%	ambig	17			1,33E-010
reg_DEFB	YES	chr8	7223613	A	C	96	31,00%	het	30			1,00E-012
reg_DEFB	YES	chr8	7223632	C	A	93	11,00%	ambig	10			5,88E-005
reg_DEFB	YES	chr8	7223670	A	T	82	11,00%	ambig	9			1,16E-004
reg_DEFB	YES	chr8	7223680	C	A	77	13,00%	ambig	10			1,11E-005
reg_DEFB	YES	chr8	7223684	C	G	77	10,00%	ambig	8			4,02E-004
reg_DEFB	YES	chr8	7223710	C	G	72	11,00%	ambig	8			2,53E-004
reg_DEFB	YES	chr8	7223711	A	G	71	11,00%	ambig	8			2,29E-004
reg_DEFB	YES	chr8	7223720	A	C	67	10,00%	ambig	7			8,86E-004
reg_DEFB	YES	chr8	7223728	A	G	70	14,00%	ambig	10			4,66E-006
reg_DEFB	YES	chr8	7223736	A	G	72	35,00%	het	25		rs71511230	1,00E-012
reg_DEFB	YES	chr8	7223760	G	C	67	15,00%	ambig	10	rs2719539		3,10E-006
reg_DEFB	YES	chr8	7224337	C	T	74	100,00%	homvar	74	rs9720658		1,00E-012
reg_DEFB	YES	chr8	7224910	T	G	35	100,00%	homvar	35	rs10087379		1,00E-012
reg_DEFB	YES	chr8	7225003	A	T	38	100,00%	homvar	38	rs28624344		1,00E-012
reg_DEFB	YES	chr8	7225112	G	A	48	100,00%	homvar	48	rs4840741		1,00E-012
reg_DEFB	YES	chr8	7225243	G	C	107	20,00%	ambig	21			1,00E-012

reg_DEFB	YES	chr8	7225281	G	C	134	23,00%	ambig	31				1,00E-012
reg_DEFB	YES	chr8	7225316	A	C	148	27,00%	het	40	rs9720218			1,00E-012
reg_DEFB	YES	chr8	7225374	A	G	150	89,00%	homvar	134	rs4840742			1,00E-012
reg_DEFB	YES	chr8	7225405	A	G	155	18,00%	ambig	28				1,00E-012
reg_DEFB	YES	chr8	7225426	T	C	152	36,00%	het	55	rs9720354			1,00E-012
reg_DEFB	YES	chr8	7225443	C	T	152	20,00%	ambig	30				1,00E-012
reg_DEFB	YES	chr8	7225457	A	C	154	20,00%	ambig	31				1,00E-012
reg_DEFB	YES	chr8	7225462	T	C	153	37,00%	het	57				1,00E-012
reg_DEFB	YES	chr8	7225473	C	A	154	21,00%	ambig	32				1,00E-012
reg_DEFB	YES	chr8	7225488	G	A	151	17,00%	ambig	26				1,00E-012
reg_DEFB	YES	chr8	7225515	A	G	150	18,00%	ambig	27				1,00E-012
reg_DEFB	YES	chr8	7225527	T	C	146	18,00%	ambig	26				1,00E-012
reg_DEFB	YES	chr8	7225533	T	C	144	19,00%	ambig	27				1,00E-012
reg_DEFB	YES	chr8	7225561	A	C	136	27,00%	het	37				1,00E-012
reg_DEFB	YES	chr8	7225563	G	A	135	45,00%	het	61				1,00E-012
reg_DEFB	YES	chr8	7225570	G	C	134	48,00%	het	64				1,00E-012
reg_DEFB	YES	chr8	7225582	C	A	132	20,00%	ambig	26				1,00E-012
reg_DEFB	YES	chr8	7225613	C	T	107	21,00%	ambig	22				1,00E-012
reg_DEFB	YES	chr8	7225617	G	C	106	22,00%	ambig	23				1,00E-012
reg_DEFB	YES	chr8	7225642	G	T	86	47,00%	het	40				1,00E-012
reg_DEFB	YES	chr8	7225690	G	A	60	17,00%	ambig	10	rs2740645			1,09E-006
reg_DEFB	YES	chr8	7225701	T	C	52	13,00%	ambig	7				1,84E-004
reg_DEFB	YES	chr8	7225714	G	A	42	26,00%	het	11				2,12E-009
reg_DEFB	YES	chr8	7225735	C	T	38	13,00%	ambig	5				1,72E-003
reg_DEFB	YES	chr8	7225750	A	G	34	29,00%	het	10			rs58658719	3,28E-009
reg_DEFB		chr8	7226320	A	G	57	91,00%	homvar	52	rs2740644			1,00E-012
reg_DEFB		chr8	7226650	T	G	47	98,00%	homvar	46			rs62636823	1,00E-012
reg_DEFB		chr8	7227202	C	G	60	98,00%	homvar	59	rs4110303			1,00E-012
reg_DEFB		chr8	7227284	A	C	46	98,00%	homvar	45			rs71511231	1,00E-012
reg_DEFB		chr8	7227642	C	T	29	100,00%	homvar	29	rs3915363			1,00E-012
reg_DEFB		chr8	7227922	A	T	19	95,00%	homvar	18	rs28413583	rs5004605		1,00E-012
reg_DEFB		chr8	7230646	C	G	33	94,00%	homvar	31			rs4295681	1,00E-012
reg_DEFB		chr8	7230752	G	A	30	83,00%	homvar	25			rs71511233	1,00E-012
reg_DEFB		chr8	7230809	G	T	29	52,00%	het	15				1,00E-012
reg_DEFB		chr8	7231435	G	A	30	100,00%	homvar	30			rs71511234	1,00E-012
reg_DEFB		chr8	7231456	G	A	33	100,00%	homvar	33	rs2739878			1,00E-012
reg_DEFB		chr8	7231549	C	G	36	97,00%	homvar	35	rs2719513			1,00E-012
reg_DEFB		chr8	7232643	A	T	42	93,00%	homvar	39	rs3915354			1,00E-012
reg_DEFB		chr8	7232788	A	G	38	92,00%	homvar	35	rs2698867			1,00E-012
reg_DEFB		chr8	7233725	C	T	24	96,00%	homvar	23	rs2740632			1,00E-012
reg_DEFB		chr8	7235443	G	C	88	95,00%	homvar	84	rs9720375			1,00E-012
reg_DEFB		chr8	7235967	G	T	35	11,00%	ambig	4	rs2719506			8,30E-003

reg_DEFB	chr8	7235975	G	A	33	36,00%	het	12		rs71511240	1,03E-011
reg_DEFB	chr8	7236869	C	T	35	43,00%	het	15			1,00E-012
reg_DEFB	chr8	7238365	A	G	29	97,00%	homvar	28	rs9720329		1,00E-012
reg_DEFB	chr8	7240395	C	A	56	96,00%	homvar	54	rs13260072		1,00E-012
reg_DEFB	chr8	7240612	C	A	49	39,00%	het	19	rs2740609		1,00E-012
reg_DEFB	chr8	7240705	T	C	45	44,00%	het	20	rs7005466		1,00E-012
reg_DEFB	chr8	7240831	C	T	38	24,00%	ambig	9		rs71250725	1,60E-007
reg_DEFB	chr8	7240856	C	A	39	28,00%	het	11	rs2740608		8,84E-010
reg_DEFB	chr8	7241049	T	C	24	25,00%	het	6		rs2698963	1,39E-005
reg_DEFB	chr8	7241078	T	C	23	30,00%	het	7		rs71250726	6,03E-007
reg_DEFB	chr8	7241147	C	A	27	41,00%	het	11		rs2698964	1,60E-011
reg_DEFB	chr8	7241218	T	C	30	50,00%	het	15	rs7009957		1,00E-012
reg_DEFB	chr8	7241259	A	T	30	47,00%	het	14			1,00E-012
reg_DEFB	chr8	7241264	C	T	31	52,00%	het	16	rs4625055		1,00E-012
reg_DEFB	chr8	7241285	A	G	32	50,00%	het	16		rs3958824	1,00E-012
reg_DEFB	chr8	7241358	T	C	31	45,00%	het	14		rs2740157	1,00E-012
reg_DEFB	chr8	7241366	G	T	32	41,00%	het	13		rs2740606	1,00E-012
reg_DEFB	chr8	7241686	A	G	34	97,00%	homvar	33	rs4571754		1,00E-012
reg_DEFB	chr8	7241782	T	G	20	90,00%	homvar	18			1,00E-012
reg_DEFB	chr8	7241787	C	T	21	38,00%	het	8		rs2740605	1,22E-008
reg_DEFB	chr8	7242034	T	A	42	57,00%	het	24		rs71247376	1,00E-012
reg_DEFB	chr8	7242037	G	C	42	57,00%	het	24		rs71247376	1,00E-012
reg_DEFB	chr8	7242252	G	C	35	49,00%	het	17		rs3988892	1,00E-012
reg_DEFB	chr8	7242513	T	C	41	46,00%	het	19		rs3988890	1,00E-012
reg_DEFB	chr8	7242527	T	C	42	48,00%	het	20			1,00E-012
reg_DEFB	chr8	7242637	T	G	42	55,00%	het	23		rs3927359	1,00E-012
reg_DEFB	chr8	7242769	A	T	34	50,00%	het	17		rs72494256	1,00E-012
reg_DEFB	chr8	7242795	G	C	35	100,00%	homvar	35		rs3928107	1,00E-012
reg_DEFB	chr8	7242850	G	A	38	47,00%	het	18		rs2740749	1,00E-012
reg_DEFB	chr8	7242925	C	T	37	54,00%	het	20	rs2698913		1,00E-012
reg_DEFB	chr8	7243080	G	A	35	37,00%	het	13			1,00E-012
reg_DEFB	chr8	7243097	A	G	39	36,00%	het	14			1,00E-012
reg_DEFB	chr8	7243226	G	C	42	36,00%	het	15		rs56017112	1,00E-012
reg_DEFB	chr8	7243230	C	T	41	37,00%	het	15		rs2719607	1,00E-012
reg_DEFB	chr8	7243275	T	A	43	35,00%	het	15		rs2719604	1,00E-012
reg_DEFB	chr8	7243307	T	C	40	40,00%	het	16		rs2719603	1,00E-012
reg_DEFB	chr8	7243453	C	T	40	45,00%	het	18	rs4840743		1,00E-012
reg_DEFB	chr8	7243457	A	T	40	92,00%	homvar	37	rs4840275		1,00E-012
reg_DEFB	chr8	7243460	T	C	38	42,00%	het	16			1,00E-012
reg_DEFB	chr8	7243517	G	A	33	45,00%	het	15		rs2463985	1,00E-012
reg_DEFB	chr8	7243526	C	T	30	50,00%	het	15		rs2466114	1,00E-012
reg_DEFB	chr8	7243649	A	G	30	37,00%	het	11	rs2463984		3,60E-011

reg_DEFB	chr8	7243739	T	C	33	42,00%	het	14				1,00E-012
reg_DEFB	chr8	7243778	T	C	35	54,00%	het	19	rs2737539			1,00E-012
reg_DEFB	chr8	7243809	T	C	37	43,00%	het	16	rs2977410			1,00E-012
reg_DEFB	chr8	7243954	C	A	37	41,00%	het	15	rs2740150	rs34384791		1,00E-012
reg_DEFB	chr8	7243999	A	G	36	36,00%	het	13	rs2740149	rs35454996		1,00E-012
reg_DEFB	chr8	7244256	G	C	16	44,00%	het	7	rs2954087			3,25E-008
reg_DEFB	chr8	7244340	C	T	17	47,00%	het	8	rs2977415			1,60E-009
reg_DEFB	chr8	7244846	T	C	30	100,00%	homvar	30	rs34757760			1,00E-012
reg_DEFB	chr8	7244847	G	A	31	100,00%	homvar	31	rs34757760			1,00E-012
reg_DEFB	chr8	7244856	T	G	32	97,00%	homvar	31	rs35642932			1,00E-012
reg_DEFB	chr8	7245066	G	A	29	41,00%	het	12	rs2977405			1,00E-012
reg_DEFB	chr8	7245210	G	A	39	38,00%	het	15	rs2977408	rs2977409		1,00E-012
reg_DEFB	chr8	7245290	C	T	38	42,00%	het	16	rs35021319			1,00E-012
reg_DEFB	chr8	7245577	T	C	17	47,00%	het	8	rs2740148	rs4840745		1,60E-009
reg_DEFB	chr8	7245649	G	A	16	56,00%	het	9	rs2740146	rs4840746		2,54E-011
reg_DEFB	chr8	7245898	G	C	24	92,00%	homvar	22	rs2719573			1,00E-012
reg_DEFB	chr8	7246164	C	T	46	46,00%	het	21	rs2719567			1,00E-012
reg_DEFB	chr8	7246774	G	A	27	52,00%	het	14	rs2977411			1,00E-012
reg_DEFB	chr8	7247056	G	A	44	48,00%	het	21	rs2954062			1,00E-012
reg_DEFB	chr8	7247198	C	A	36	39,00%	het	14	rs2954061			1,00E-012
reg_DEFB	chr8	7247379	C	T	17	71,00%	het	12	rs2740143	rs34835930		1,00E-012
reg_DEFB	chr8	7247486	G	A	13	23,00%	ambig	3	rs34558211			2,93E-003
reg_DEFB	chr8	7247554	A	C	18	17,00%	ambig	3				7,66E-003
reg_DEFB	chr8	7247586	T	C	16	19,00%	ambig	3				5,44E-003
reg_DEFB	chr8	7247602	T	C	15	20,00%	ambig	3				4,50E-003
reg_DEFB	chr8	7247667	A	G	16	19,00%	ambig	3				5,44E-003
reg_DEFB	chr8	7247690	T	C	16	31,00%	het	5				2,27E-005
reg_DEFB	chr8	7247728	A	C	20	25,00%	het	5				7,47E-005
reg_DEFB	chr8	7247737	T	C	19	42,00%	het	8				4,72E-009
reg_DEFB	chr8	7247775	A	C	16	50,00%	het	8				8,62E-010
reg_DEFB	chr8	7247782	C	T	16	19,00%	ambig	3				5,44E-003
reg_DEFB	chr8	7247825	C	T	15	20,00%	ambig	3				4,50E-003
reg_DEFB	chr8	7247847	G	C	14	64,00%	het	9				4,45E-012
reg_DEFB	chr8	7247936	A	G	9	56,00%	het	5				7,51E-007
reg_DEFB	chr8	7247959	T	A	8	62,00%	het	5	rs2266498			3,40E-007
reg_DEFB	chr8	7247963	A	G	8	37,00%	het	3				6,25E-004
reg_DEFB	chr8	7248073	T	A	6	50,00%	het	3				2,31E-004
reg_DEFB	chr8	7249027	A	C	3	100,00%	homvar	3				1,22E-005
reg_DEFB	chr8	7249069	C	G	3	100,00%	homvar	3				1,22E-005
reg_DEFB	chr8	7249100	T	C	3	100,00%	homvar	3	rs2740138			1,22E-005
reg_DEFB	chr8	7249269	T	C	4	75,00%	het	3			rs62494626	4,78E-005
reg_DEFB	chr8	7249925	G	A	5	60,00%	het	3			rs62510592	1,18E-004

reg_DEFB	chr8	7250102	G	A	7	86,00%	homvar	6			rs62636833	1,02E-009
reg_DEFB	chr8	7250132	C	T	8	62,00%	het	5			rs4108895	3,40E-007
reg_DEFB	chr8	7250139	A	C	8	87,00%	homvar	7				3,21E-011
reg_DEFB	chr8	7250185	A	G	9	33,00%	het	3				9,21E-004
reg_DEFB	chr8	7250219	T	C	10	90,00%	homvar	9				1,00E-012
reg_DEFB	chr8	7250228	C	T	10	30,00%	het	3				1,29E-003
reg_DEFB	chr8	7250289	T	C	14	57,00%	het	8			rs35064420	2,09E-010
reg_DEFB	chr8	7250365	G	A	12	42,00%	het	5			rs9802024	4,45E-006
reg_DEFB	chr8	7250371	T	C	12	58,00%	het	7			rs71521097	2,45E-009
reg_DEFB	chr8	7250404	T	C	10	90,00%	homvar	9				1,00E-012
reg_DEFB	chr8	7250428	T	G	10	80,00%	homvar	8			rs28883954	4,25E-012
reg_DEFB	chr8	7250500	T	G	4	100,00%	homvar	4				2,80E-007
reg_DEFB	chr8	7252395	G	C	4	75,00%	het	3				4,78E-005
reg_DEFB	chr8	7252477	T	C	5	80,00%	homvar	4	rs2740113			1,37E-006
reg_DEFB	chr8	7252507	A	G	6	83,00%	homvar	5				3,79E-008
reg_DEFB	chr8	7252518	A	G	6	83,00%	homvar	5				3,79E-008
reg_DEFB	chr8	7252595	A	G	6	67,00%	het	4			rs4876565	4,04E-006
reg_DEFB	chr8	7252655	A	G	3	100,00%	homvar	3				1,22E-005
reg_DEFB	chr8	7253101	G	A	16	44,00%	het	7	rs2698826	rs3988849		3,25E-008
reg_DEFB	chr8	7253210	C	T	18	39,00%	het	7	rs3988850			8,67E-008
reg_DEFB	chr8	7253275	G	A	20	35,00%	het	7			rs2719571	2,03E-007
reg_DEFB	chr8	7253389	T	C	27	52,00%	het	14	rs2719572			1,00E-012
reg_DEFB	chr8	7254208	T	C	38	39,00%	het	15				1,00E-012
reg_DEFB	chr8	7254247	A	G	35	80,00%	homvar	28	rs3915368			1,00E-012
reg_DEFB	chr8	7254807	C	G	71	52,00%	het	37	rs2740741			1,00E-012
reg_DEFB	chr8	7255839	C	T	64	94,00%	homvar	60	rs3877964			1,00E-012
reg_DEFB	chr8	7256331	C	G	49	51,00%	het	25	rs2719545			1,00E-012
reg_DEFB	chr8	7256550	G	A	44	45,00%	het	20			rs3915496	1,00E-012
reg_DEFB	chr8	7256617	G	A	44	98,00%	homvar	43	rs28576922	rs3866483		1,00E-012
reg_DEFB	chr8	7256739	C	T	30	33,00%	het	10			rs7813724	8,17E-010
reg_DEFB	chr8	7256872	G	A	8	37,00%	het	3			rs71513067	6,25E-004
reg_DEFB	chr8	7257104	T	C	31	45,00%	het	14	rs2740095			1,00E-012
reg_DEFB	chr8	7257126	G	A	34	100,00%	homvar	34	rs4840277			1,00E-012
reg_DEFB	chr8	7257138	G	A	34	50,00%	het	17	rs7386020			1,00E-012
reg_DEFB	chr8	7257305	C	A	49	51,00%	het	25			rs71247231	1,00E-012
reg_DEFB	chr8	7258456	G	T	78	22,00%	ambig	17	rs2737914			7,16E-012
reg_DEFB	chr8	7258464	G	A	76	32,00%	het	24			rs72626630	1,00E-012
reg_DEFB	chr8	7259539	G	A	67	40,00%	het	27	rs41390446			1,00E-012
reg_DEFB	chr8	7259563	G	T	65	38,00%	het	25	rs41507446			1,00E-012
reg_DEFB	chr8	7259739	C	T	80	49,00%	het	39	rs2737531			1,00E-012
reg_DEFB	chr8	7260004	A	G	72	42,00%	het	30	rs2740090			1,00E-012
reg_DEFB	chr8	7260322	T	C	35	57,00%	het	20			rs71251804	1,00E-012

reg_DEFB	chr8	7260751	C	T	6	67,00%	het	4			4,04E-006
reg_DEFB	chr8	7261175	C	T	54	44,00%	het	24			1,00E-012
reg_DEFB	chr8	7261330	G	A	48	37,00%	het	18			1,00E-012
reg_DEFB	chr8	7261371	G	T	46	39,00%	het	18			1,00E-012
reg_DEFB	chr8	7261718	A	G	50	100,00%	homvar	50		rs71509106	1,00E-012
reg_DEFB	chr8	7261867	A	G	65	43,00%	het	28	rs2740086		1,00E-012
reg_DEFB	chr8	7262672	G	A	42	48,00%	het	20		rs71513122	1,00E-012
reg_DEFB	chr8	7262787	C	T	38	47,00%	het	18	rs3762040		1,00E-012
reg_DEFB	chr8	7263102	T	C	46	98,00%	homvar	45	rs4840278		1,00E-012
reg_DEFB	chr8	7263191	G	C	33	52,00%	het	17	rs3762052		1,00E-012
reg_DEFB	chr8	7263349	G	A	24	58,00%	het	14	rs3762051		1,00E-012
reg_DEFB	chr8	7263407	A	G	30	50,00%	het	15		rs71244071	1,00E-012
reg_DEFB	chr8	7263531	T	C	38	53,00%	het	20			1,00E-012
reg_DEFB	chr8	7263605	A	G	34	41,00%	het	14		rs56020554	1,00E-012
reg_DEFB	chr8	7263647	A	G	30	37,00%	het	11			3,60E-011
reg_DEFB	chr8	7263683	T	C	32	37,00%	het	12			6,54E-012
reg_DEFB	chr8	7263744	G	A	35	37,00%	het	13	rs2740083		1,00E-012
reg_DEFB	chr8	7263764	G	C	32	34,00%	het	11	rs2409862		8,20E-011
reg_DEFB	chr8	7263877	A	C	27	100,00%	homvar	27	rs6651513		1,00E-012
reg_DEFB	chr8	7263885	C	T	29	31,00%	het	9	rs3762038		1,19E-008
reg_DEFB	chr8	7263922	G	T	24	21,00%	ambig	5	rs3988843		1,90E-004
reg_DEFB	chr8	7264091	G	A	30	43,00%	het	13	rs3762037		1,00E-012
reg_DEFB	chr8	7264095	A	G	30	40,00%	het	12	rs3988844		2,51E-012
reg_DEFB	chr8	7264156	G	C	36	81,00%	homvar	29	rs2737536		1,00E-012
reg_DEFB	chr8	7264462	A	C	52	38,00%	het	20		rs71254898	1,00E-012
reg_DEFB	chr8	7264552	A	C	46	39,00%	het	18	rs2737538		1,00E-012
reg_DEFB	chr8	7264604	G	A	45	42,00%	het	19		rs71513120	1,00E-012
reg_DEFB	chr8	7264753	G	C	35	40,00%	het	14		rs71537819	1,00E-012
reg_DEFB	chr8	7264799	C	T	37	43,00%	het	16	rs3866482		1,00E-012
reg_DEFB	chr8	7264836	T	G	36	39,00%	het	14	rs4118281		1,00E-012
reg_DEFB	chr8	7264944	G	C	35	94,00%	homvar	33	rs4840753		1,00E-012
reg_DEFB	chr8	7264948	G	A	35	37,00%	het	13	rs3866481		1,00E-012
reg_DEFB	chr8	7265160	T	C	29	45,00%	het	13		rs71513119	1,00E-012
reg_DEFB	chr8	7265202	A	T	35	43,00%	het	15		rs71513118	1,00E-012
reg_DEFB	chr8	7265254	T	C	33	39,00%	het	13		rs71513117	1,00E-012
reg_DEFB	chr8	7265340	T	C	36	56,00%	het	20	rs2698832		1,00E-012
reg_DEFB	chr8	7265354	C	T	37	30,00%	het	11			4,70E-010
reg_DEFB	chr8	7265410	G	A	41	22,00%	ambig	9		rs71513116	3,24E-007
reg_DEFB	chr8	7265947	G	T	42	33,00%	het	14	rs3988845		1,00E-012
reg_DEFB	chr8	7265983	G	A	53	55,00%	het	29	rs3866480		1,00E-012
reg_DEFB	chr8	7266035	T	C	54	43,00%	het	23	rs3988846		1,00E-012
reg_DEFB	chr8	7266053	C	T	55	49,00%	het	27	rs2740731		1,00E-012

reg_DEFB	chr8	7266297	C	A	55	45,00%	het	25	rs3988829			1,00E-012
reg_DEFB	chr8	7266317	C	T	55	47,00%	het	26	rs3988830			1,00E-012
reg_DEFB	chr8	7266358	C	T	45	44,00%	het	20	rs3988831			1,00E-012
reg_DEFB	chr8	7266438	A	C	40	42,00%	het	17	rs3988832			1,00E-012
reg_DEFB	chr8	7266786	T	C	49	41,00%	het	20			rs71513115	1,00E-012
reg_DEFB	chr8	7266858	C	T	51	47,00%	het	24			rs71513114	1,00E-012
reg_DEFB	chr8	7267031	T	G	65	100,00%	homvar	65	rs7815816			1,00E-012
reg_DEFB	chr8	7267055	A	C	63	38,00%	het	24			rs71513113	1,00E-012
reg_DEFB	chr8	7267305	C	G	46	39,00%	het	18	rs2737543			1,00E-012
reg_DEFB	chr8	7267395	G	T	33	27,00%	het	9			rs71513110	4,21E-008
reg_DEFB	chr8	7267436	A	C	28	43,00%	het	12			rs71513109	1,00E-012
reg_DEFB	chr8	7267461	T	G	28	46,00%	het	13			rs71513108	1,00E-012
reg_DEFB	chr8	7267609	T	G	52	50,00%	het	26			rs71513107	1,00E-012
reg_DEFB	chr8	7267636	C	G	55	49,00%	het	27			rs71513106	1,00E-012
reg_DEFB	chr8	7267855	A	G	59	98,00%	homvar	58	rs3958991			1,00E-012
reg_DEFB	chr8	7268025	T	A	59	46,00%	het	27	rs28610011	rs3866479		1,00E-012
reg_DEFB	chr8	7268334	A	G	53	38,00%	het	20				1,00E-012
reg_DEFB	chr8	7268520	A	T	48	50,00%	het	24			rs71513105	1,00E-012
reg_DEFB	chr8	7268623	G	T	49	98,00%	homvar	48	rs4840754			1,00E-012
reg_DEFB	chr8	7268975	C	T	40	50,00%	het	20	rs3958992			1,00E-012
reg_DEFB	chr8	7269055	T	C	33	100,00%	homvar	33				1,00E-012
reg_DEFB	chr8	7269085	G	T	37	97,00%	homvar	36				1,00E-012
reg_DEFB	chr8	7269368	T	C	35	97,00%	homvar	34			rs71511302	1,00E-012
reg_DEFB	chr8	7269455	T	C	31	42,00%	het	13			rs2698835	1,00E-012
reg_DEFB	chr8	7269776	G	A	34	94,00%	homvar	32			rs2737547	1,00E-012
reg_DEFB	chr8	7269817	C	A	36	100,00%	homvar	36			rs62636842	1,00E-012
reg_DEFB	chr8	7269942	G	T	32	97,00%	homvar	31				1,00E-012
reg_DEFB	chr8	7269967	T	C	34	94,00%	homvar	32				1,00E-012
reg_DEFB	chr8	7270168	G	T	32	94,00%	homvar	30				1,00E-012
reg_DEFB	chr8	7270508	C	T	38	63,00%	het	24			rs71511299	1,00E-012
reg_DEFB	chr8	7270703	T	C	33	45,00%	het	15			rs71511298	1,00E-012
reg_DEFB	chr8	7270806	C	T	23	52,00%	het	12	rs2740728			1,00E-012
reg_DEFB	chr8	7270994	G	A	16	50,00%	het	8	rs2737548			8,62E-010
reg_DEFB	chr8	7271226	C	A	18	22,00%	ambig	4				6,61E-004
reg_DEFB	chr8	7271232	T	C	18	22,00%	ambig	4				6,61E-004
reg_DEFB	chr8	7271434	T	C	18	100,00%	homvar	18			rs4543549	1,00E-012
reg_DEFB	chr8	7271837	C	T	45	58,00%	het	26			rs62636845	1,00E-012
reg_DEFB	chr8	7271938	C	T	62	55,00%	het	34			rs62636846	1,00E-012
reg_DEFB	chr8	7272893	G	A	80	47,00%	het	38	rs3988902			1,00E-012
reg_DEFB	chr8	7272905	G	C	80	99,00%	homvar	79			rs71509107	1,00E-012
reg_DEFB	chr8	7272963	A	G	76	41,00%	het	31			rs71511249	1,00E-012
reg_DEFB	chr8	7273532	G	C	53	58,00%	het	31	rs3866478			1,00E-012

reg_DEFB	chr8	7273819	A	G	34	41,00%	het	14					1,00E-012
reg_DEFB	chr8	7274151	C	T	66	64,00%	het	42	-	DEFB103B		rs62636848	1,00E-012
reg_DEFB	chr8	7274576	T	C	26	42,00%	het	11	-	DEFB103B	rs3789865		9,51E-012
reg_DEFB	chr8	7274717	T	C	18	50,00%	het	9	-	DEFB103B	rs3789864		7,33E-011
reg_DEFB	chr8	7274787	G	C	20	40,00%	het	8	-	DEFB103B	rs3789862		7,71E-009
reg_DEFB	chr8	7275507	G	T	38	39,00%	het	15				rs71511250	1,00E-012
reg_DEFB	chr8	7275590	A	G	25	48,00%	het	12			rs4461923		1,00E-012
reg_DEFB	chr8	7275765	T	C	15	33,00%	het	5					1,59E-005
reg_DEFB	chr8	7275809	A	G	21	62,00%	het	13				rs71511253	1,00E-012
reg_DEFB	chr8	7275956	G	A	33	55,00%	het	18					1,00E-012
reg_DEFB	chr8	7276674	A	C	77	93,00%	homvar	72			rs2737554		1,00E-012
reg_DEFB	chr8	7277359	T	C	58	100,00%	homvar	58				rs71509108	1,00E-012
reg_DEFB	chr8	7277387	T	C	60	45,00%	het	27				rs71511255	1,00E-012
reg_DEFB	chr8	7277422	T	A	62	100,00%	homvar	62				rs71509109	1,00E-012
reg_DEFB	chr8	7277424	C	A	62	94,00%	homvar	58				rs71509110	1,00E-012
reg_DEFB	chr8	7277451	C	G	64	44,00%	het	28				rs71511256	1,00E-012
reg_DEFB	chr8	7277460	G	A	63	43,00%	het	27				rs71511257	1,00E-012
reg_DEFB	chr8	7277507	T	C	68	59,00%	het	40					1,00E-012
reg_DEFB	chr8	7277521	C	G	68	37,00%	het	25				rs71511259	1,00E-012
reg_DEFB	chr8	7277545	C	T	68	34,00%	het	23				rs71511260	1,00E-012
reg_DEFB	chr8	7277566	G	A	62	35,00%	het	22				rs71509111	1,00E-012
reg_DEFB	chr8	7277662	A	G	60	32,00%	het	19				rs4999975	1,00E-012
reg_DEFB	chr8	7277710	T	C	55	27,00%	het	15				rs4999974	3,47E-012
reg_DEFB	chr8	7277744	A	C	57	30,00%	het	17				rs71252681	1,00E-012
reg_DEFB	chr8	7277755	T	C	56	34,00%	het	19			rs4999973		1,00E-012
reg_DEFB	chr8	7277810	A	G	59	34,00%	het	20					1,00E-012
reg_DEFB	chr8	7277830	C	G	54	26,00%	het	14					1,74E-011
reg_DEFB	chr8	7278010	C	T	84	98,00%	homvar	82					1,00E-012
reg_DEFB	chr8	7278116	C	T	97	51,00%	het	49				rs4840303	1,00E-012
reg_DEFB	chr8	7278542	T	A	81	100,00%	homvar	81			rs41380147		1,00E-012
reg_DEFB	chr8	7278548	A	C	81	48,00%	het	39				rs4840304	1,00E-012
reg_DEFB	chr8	7278563	G	T	85	46,00%	het	39				rs9693075	1,00E-012
reg_DEFB	chr8	7278594	T	C	87	97,00%	homvar	84					1,00E-012
reg_DEFB	chr8	7278727	C	T	91	42,00%	het	38				rs71276791	1,00E-012
reg_DEFB	chr8	7279289	T	C	56	43,00%	het	24					1,00E-012
reg_DEFB	chr8	7279315	T	C	44	98,00%	homvar	43					1,00E-012
reg_DEFB	chr8	7279450	C	T	37	41,00%	het	15			rs4840306		1,00E-012
reg_DEFB	chr8	7279696	A	T	63	49,00%	het	31			rs2737899		1,00E-012
reg_DEFB	chr8	7279780	C	G	64	53,00%	het	34				rs71276797	1,00E-012
reg_DEFB	chr8	7279877	C	T	61	57,00%	het	35				rs71276798	1,00E-012
reg_DEFB	chr8	7280090	T	C	48	94,00%	homvar	45					1,00E-012
reg_DEFB	chr8	7280221	C	T	64	48,00%	het	31				rs71299143	1,00E-012

reg_DEFB	chr8	7280224	G	C	64	44,00%	het	28			1,00E-012
reg_DEFB	chr8	7280227	A	T	64	95,00%	homvar	61			1,00E-012
reg_DEFB	chr8	7280259	T	G	60	47,00%	het	28	rs2698836		1,00E-012
reg_DEFB	chr8	7280364	G	C	63	98,00%	homvar	62		rs2737897	1,00E-012
reg_DEFB	chr8	7280790	G	A	51	43,00%	het	22	rs2737896		1,00E-012
reg_DEFB	chr8	7281118	T	C	49	57,00%	het	28	rs2737895		1,00E-012
reg_DEFB	chr8	7281292	C	T	37	100,00%	homvar	37	rs4840279		1,00E-012
reg_DEFB	chr8	7281372	T	G	35	37,00%	het	13	rs4247403		1,00E-012
reg_DEFB	chr8	7281483	A	T	32	28,00%	het	9	rs2740074		3,13E-008
reg_DEFB	chr8	7281562	C	T	29	52,00%	het	15		rs2737894	1,00E-012
reg_DEFB	chr8	7281568	A	T	29	41,00%	het	12	rs2740073		1,00E-012
reg_DEFB	chr8	7281649	T	A	26	46,00%	het	12		rs71509112	1,00E-012
reg_DEFB	chr8	7281710	G	T	28	46,00%	het	13	rs2698838		1,00E-012
reg_DEFB	chr8	7282103	G	T	37	38,00%	het	14		rs71267734	1,00E-012
reg_DEFB	chr8	7282443	T	C	48	96,00%	homvar	46			1,00E-012
reg_DEFB	chr8	7282560	C	T	60	35,00%	het	21		rs71267735	1,00E-012
reg_DEFB	chr8	7282592	G	A	63	40,00%	het	25		rs71267736	1,00E-012
reg_DEFB	chr8	7282670	T	C	73	97,00%	homvar	71			1,00E-012
reg_DEFB	chr8	7282719	A	G	81	51,00%	het	41		rs2737556	1,00E-012
reg_DEFB	chr8	7282985	T	A	81	48,00%	het	39		rs2737892	1,00E-012
reg_DEFB	chr8	7283081	C	T	61	51,00%	het	31	rs3915374		1,00E-012
reg_DEFB	chr8	7283283	C	T	45	51,00%	het	23		rs71267737	1,00E-012
reg_DEFB	chr8	7283305	A	G	38	97,00%	homvar	37			1,00E-012
reg_DEFB	chr8	7283441	C	G	31	48,00%	het	15		rs71267738	1,00E-012
reg_DEFB	chr8	7283465	C	T	30	47,00%	het	14		rs71267739	1,00E-012
reg_DEFB	chr8	7283568	A	G	25	36,00%	het	9		rs7836098	2,64E-009
reg_DEFB	chr8	7283609	G	C	31	32,00%	het	10		rs71267741	1,18E-009
reg_DEFB	chr8	7283828	C	G	72	58,00%	het	42	rs3915371		1,00E-012
reg_DEFB	chr8	7283981	G	A	81	93,00%	homvar	75		rs3915370	1,00E-012
reg_DEFB	chr8	7284078	A	G	84	45,00%	het	38			1,00E-012
reg_DEFB	chr8	7284088	T	C	83	45,00%	het	37			1,00E-012
reg_DEFB	chr8	7284285	G	A	38	45,00%	het	17	rs2698842		1,00E-012
reg_DEFB	chr8	7284296	G	T	35	51,00%	het	18			1,00E-012
reg_DEFB	chr8	7284304	G	C	33	52,00%	het	17			1,00E-012
reg_DEFB	chr8	7284416	A	G	22	32,00%	het	7		rs71267742	4,28E-007
reg_DEFB	chr8	7284587	C	T	16	44,00%	het	7		rs71267744	3,25E-008
reg_DEFB	chr8	7284697	G	C	21	52,00%	het	11		rs71267745	1,00E-012
reg_DEFB	chr8	7284872	T	A	34	53,00%	het	18		rs71267746	1,00E-012
reg_DEFB	chr8	7284977	A	G	37	54,00%	het	20		rs71267747	1,00E-012
reg_DEFB	chr8	7285008	T	A	35	49,00%	het	17		rs71267748	1,00E-012
reg_DEFB	chr8	7285027	C	T	35	34,00%	het	12	rs2740069		2,42E-011
reg_DEFB	chr8	7285028	A	G	35	40,00%	het	14	rs2740069		1,00E-012

reg_DEFB	chr8	7285109	C	T	34	47,00%	het	16							rs71267749	1,00E-012
reg_DEFB	chr8	7285531	G	C	58	43,00%	het	25					rs2740068	rs2737887		1,00E-012
reg_DEFB	chr8	7285682	T	C	52	42,00%	het	22							rs2698845	1,00E-012
reg_DEFB	chr8	7285744	C	T	47	94,00%	homvar	44					rs3988838			1,00E-012
reg_DEFB	chr8	7285834	T	A	43	86,00%	homvar	37					rs4501606			1,00E-012
reg_DEFB	chr8	7285981	C	G	42	93,00%	homvar	39							rs3988839	1,00E-012
reg_DEFB	chr8	7286036	C	T	47	45,00%	het	21							rs71267752	1,00E-012
reg_DEFB	chr8	7286545	G	A	80	95,00%	homvar	76					rs2280948			1,00E-012
reg_DEFB	chr8	7286699	A	T	88	50,00%	het	44					rs2280947			1,00E-012
reg_DEFB	chr8	7286812	G	A	98	47,00%	het	46					rs2280946			1,00E-012
reg_DEFB	chr8	7286995	G	C	66	94,00%	homvar	62					rs2280945			1,00E-012
reg_DEFB	chr8	7287010	G	A	65	58,00%	het	38					rs2280944			1,00E-012
reg_DEFB	chr8	7287700	A	G	37	51,00%	het	19					rs2740064			1,00E-012
reg_DEFB	chr8	7287713	T	C	38	97,00%	homvar	37								1,00E-012
reg_DEFB	chr8	7287772	G	A	34	59,00%	het	20							rs71267754	1,00E-012
reg_DEFB	chr8	7287838	G	T	38	29,00%	het	11							rs71267755	6,48E-010
reg_DEFB	chr8	7288049	G	A	27	59,00%	het	16					rs2740063			1,00E-012
reg_DEFB	chr8	7288309	A	C	28	11,00%	ambig	3								2,60E-002
reg_DEFB	chr8	7288723	A	G	62	37,00%	het	23					rs2740060			1,00E-012
reg_DEFB	chr8	7288985	T	C	53	94,00%	homvar	50					rs4466423			1,00E-012
reg_DEFB	chr8	7289545	G	A	75	47,00%	het	35					rs2740059			1,00E-012
reg_DEFB	chr8	7289788	C	T	49	45,00%	het	22					rs2698849			1,00E-012
reg_DEFB	chr8	7289903	T	G	47	45,00%	het	21							rs71267756	1,00E-012
reg_DEFB	chr8	7290162	T	G	23	70,00%	het	16					rs2698850			1,00E-012
reg_DEFB	chr8	7290359	A	G	35	34,00%	het	12							rs71267757	2,42E-011
reg_DEFB	chr8	7290646	G	A	38	55,00%	het	21					rs2740053			1,00E-012
reg_DEFB	chr8	7291101	G	A	57	53,00%	het	30							rs71267758	1,00E-012
reg_DEFB	chr8	7291811	C	T	55	51,00%	het	28							rs71267759	1,00E-012
reg_DEFB	chr8	7292605	G	C	36	47,00%	het	17							rs71267763	1,00E-012
reg_DEFB	chr8	7292773	T	C	43	56,00%	het	24			-	SPAG11B			rs4626629	1,00E-012
reg_DEFB	chr8	7292820	C	G	40	60,00%	het	24			-	SPAG11B			rs2740047	1,00E-012
reg_DEFB	chr8	7292896	A	C	36	58,00%	het	21			-	SPAG11B	rs3901154			1,00E-012
reg_DEFB	chr8	7293741	A	G	9	67,00%	het	6			-	SPAG11B			rs2740041	1,17E-008
reg_DEFB	chr8	7293835	G	A	13	54,00%	het	7			-	SPAG11B	rs2740040			5,17E-009
reg_DEFB	chr8	7294502	C	G	43	56,00%	het	24			-	SPAG11B	rs2853664			1,00E-012
reg_DEFB	chr8	7294800	C	T	69	59,00%	het	41			-	SPAG11B	rs2853663			1,00E-012
reg_DEFB	chr8	7295003	A	G	66	50,00%	het	33			-	SPAG11B	rs2853661			1,00E-012
reg_DEFB	chr8	7295159	G	C	58	45,00%	het	26			-	SPAG11B			rs3915372	1,00E-012
reg_DEFB	chr8	7295311	T	A	57	42,00%	het	24			-	SPAG11B	rs2853660			1,00E-012
reg_DEFB	chr8	7295321	A	T	61	48,00%	het	29			-	SPAG11B	rs2737558			1,00E-012
reg_DEFB	chr8	7295796	T	C	74	97,00%	homvar	72	H	R	-2	SPAG11B	rs1042797			1,00E-012
reg_DEFB	chr8	7295813	A	C	68	49,00%	het	33	I	M	-2	SPAG11B	rs12063			1,00E-012

reg_DEFB	chr8	7295820	G	A	68	49,00%	het	33	P	L	-2	SPAG11B	rs2256100		1,00E-012
reg_DEFB	chr8	7295830	C	T	69	43,00%	het	30	D	N	-2	SPAG11B			1,00E-012
reg_DEFB	chr8	7295894	C	A	72	40,00%	het	29			-	SPAG11B	rs2853659		1,00E-012
reg_DEFB	chr8	7295903	G	A	72	40,00%	het	29			-	SPAG11B	rs2738036		1,00E-012
reg_DEFB	chr8	7295939	C	T	67	30,00%	het	20			-	SPAG11B		rs2737559	1,00E-012
reg_DEFB	chr8	7296080	T	C	62	35,00%	het	22	D	G	-2	SPAG11B	rs2738035		1,00E-012
reg_DEFB	chr8	7296085	A	G	61	38,00%	het	23	C	C	-2	SPAG11B		rs61749560	1,00E-012
reg_DEFB	chr8	7296092	C	T	61	38,00%	het	23	R	K	-2	SPAG11B		rs61749561	1,00E-012
reg_DEFB	chr8	7296116	C	T	61	36,00%	het	22	R	Q	-2	SPAG11B	rs2853658		1,00E-012
reg_DEFB	chr8	7296208	A	G	59	100,00%	homvar	59			-	SPAG11B	rs4840280		1,00E-012
reg_DEFB	chr8	7297645	A	T	80	51,00%	het	41			-	SPAG11B		rs71511266	1,00E-012
reg_DEFB	chr8	7298128	T	C	51	39,00%	het	20			-	SPAG11B		rs71511267	1,00E-012
reg_DEFB	chr8	7298525	T	G	54	96,00%	homvar	52			-	SPAG11B		rs62636856	1,00E-012
reg_DEFB	chr8	7298616	A	G	44	50,00%	het	22			-	SPAG11B	rs2738028		1,00E-012
reg_DEFB	chr8	7299529	T	C	64	59,00%	het	38			-	SPAG11B	rs2738025		1,00E-012
reg_DEFB	chr8	7299852	A	T	48	46,00%	het	22			-	SPAG11B		rs71235967	1,00E-012
reg_DEFB	chr8	7300354	A	G	20	15,00%	ambig	3			-	SPAG11B			1,03E-002
reg_DEFB	chr8	7301157	C	G	49	29,00%	het	14			-	SPAG11B		rs71235965	9,40E-012
reg_DEFB	chr8	7301270	A	G	48	44,00%	het	21			-	SPAG11B		rs71235963	1,00E-012
reg_DEFB	chr8	7301857	A	G	16	25,00%	het	4			-	SPAG11B	rs2740031		4,08E-004
reg_DEFB	chr8	7301986	G	A	16	50,00%	het	8			-	SPAG11B	rs2738017		8,62E-010
reg_DEFB	chr8	7302658	T	C	21	57,00%	het	12			-	SPAG11B	rs2740717		1,00E-012
reg_DEFB	chr8	7302765	C	T	26	92,00%	homvar	24			-	SPAG11B	rs4840756		1,00E-012
reg_DEFB	chr8	7303683	A	T	39	46,00%	het	18			-	SPAG11B	rs4532613		1,00E-012
reg_DEFB	chr8	7303698	G	A	38	55,00%	het	21			-	SPAG11B		rs71235959	1,00E-012
reg_DEFB	chr8	7303880	C	T	21	100,00%	homvar	21			-	SPAG11B		rs62641376	1,00E-012
reg_DEFB	chr8	7303899	C	G	17	100,00%	homvar	17			-	SPAG11B		rs71526141	1,00E-012
reg_DEFB	chr8	7303958	T	C	16	100,00%	homvar	16			-	SPAG11B		rs2740712	1,00E-012
reg_DEFB	chr8	7303987	G	A	21	100,00%	homvar	21			-	SPAG11B		rs34315736	1,00E-012
reg_DEFB	chr8	7303999	A	G	23	100,00%	homvar	23			-	SPAG11B		rs7464358	1,00E-012
reg_DEFB	chr8	7304356	C	G	68	44,00%	het	30			-	SPAG11B		rs71242685	1,00E-012
reg_DEFB	chr8	7304399	A	C	66	55,00%	het	36			-	SPAG11B		rs2853665	1,00E-012
reg_DEFB	chr8	7304490	A	G	63	59,00%	het	37			-	SPAG11B		rs2737566	1,00E-012
reg_DEFB	chr8	7304912	A	G	44	98,00%	homvar	43			-	SPAG11B	rs4840757		1,00E-012
reg_DEFB	chr8	7306469	G	T	68	96,00%	homvar	65			-	SPAG11B	rs4840282		1,00E-012
reg_DEFB	chr8	7306933	C	A	62	45,00%	het	28			-	SPAG11B			1,00E-012
reg_DEFB	chr8	7307198	T	C	38	100,00%	homvar	38			-	SPAG11B		rs62636859	1,00E-012
reg_DEFB	chr8	7307400	C	G	38	47,00%	het	18			-	SPAG11B	rs2740708		1,00E-012
reg_DEFB	chr8	7307943	G	A	97	97,00%	homvar	94			-	SPAG11B	rs2251705		1,00E-012
reg_DEFB	chr8	7308164	G	A	60	45,00%	het	27			-	SPAG11B	rs3817721		1,00E-012
reg_DEFB	chr8	7308331	A	G	50	54,00%	het	27			-	SPAG11B	rs2272769		1,00E-012
reg_DEFB	chr8	7308457	C	T	51	39,00%	het	20			-	SPAG11B		rs71242684	1,00E-012

reg_DEFB	chr8	7309001	C	T	50	44,00%	het	22					rs3762045		1,00E-012
reg_DEFB	chr8	7309292	G	A	63	49,00%	het	31					rs17149290		1,00E-012
reg_DEFB	chr8	7309624	T	G	76	50,00%	het	38					rs2738013		1,00E-012
reg_DEFB	chr8	7309975	G	A	57	100,00%	homvar	57							1,00E-012
reg_DEFB	chr8	7310156	A	C	54	57,00%	het	31					rs2740702		1,00E-012
reg_DEFB	chr8	7310241	G	T	50	90,00%	homvar	45					rs4840283		1,00E-012
reg_DEFB	chr8	7310265	T	A	47	55,00%	het	26					rs2740701		1,00E-012
reg_DEFB	chr8	7311441	C	T	46	96,00%	homvar	44							1,00E-012
reg_DEFB	chr8	7311522	A	T	38	100,00%	homvar	38							1,00E-012
reg_DEFB	chr8	7312534	T	C	57	54,00%	het	31						rs71242683	1,00E-012
reg_DEFB	chr8	7312649	A	G	69	97,00%	homvar	67					rs2737570		1,00E-012
reg_DEFB	chr8	7313500	C	G	77	53,00%	het	41					rs2737573		1,00E-012
reg_DEFB	chr8	7313761	A	G	72	53,00%	het	38					rs2737574		1,00E-012
reg_DEFB	chr8	7314038	C	T	66	52,00%	het	34					rs2740026		1,00E-012
reg_DEFB	chr8	7314399	C	A	74	43,00%	het	32					rs2740025		1,00E-012
reg_DEFB	chr8	7314602	G	C	94	51,00%	het	48						rs71242681	1,00E-012
reg_DEFB	chr8	7315223	G	A	84	42,00%	het	35						rs71242680	1,00E-012
reg_DEFB	chr8	7315394	C	T	101	38,00%	het	38	R	Q	-2	DEFB104A		rs71242679	1,00E-012
reg_DEFB	chr8	7315477	A	C	97	41,00%	het	40			-	DEFB104A		rs2740023	1,00E-012
reg_DEFB	chr8	7315533	G	A	83	96,00%	homvar	80			-	DEFB104A			1,00E-012
reg_DEFB	chr8	7315562	G	T	81	37,00%	het	30			-	DEFB104A	rs2740696		1,00E-012
reg_DEFB	chr8	7315640	A	G	70	49,00%	het	34			-	DEFB104A		rs71242678	1,00E-012
reg_DEFB	chr8	7315688	A	G	62	47,00%	het	29			-	DEFB104A		rs71242677	1,00E-012
reg_DEFB	chr8	7315783	T	C	49	43,00%	het	21			-	DEFB104A		rs71242676	1,00E-012
reg_DEFB	chr8	7316113	C	T	40	85,00%	homvar	34			-	DEFB104A		rs62639764	1,00E-012
reg_DEFB	chr8	7316116	A	C	38	45,00%	het	17			-	DEFB104A	rs2740021		1,00E-012
reg_DEFB	chr8	7316428	C	T	59	97,00%	homvar	57			-	DEFB104A			1,00E-012
reg_DEFB	chr8	7316983	C	T	59	37,00%	het	22			-	DEFB104A	rs2740019		1,00E-012
reg_DEFB	chr8	7317035	C	T	57	96,00%	homvar	55			-	DEFB104A		rs62639766	1,00E-012
reg_DEFB	chr8	7317157	G	A	56	36,00%	het	20			-	DEFB104A		rs62641371	1,00E-012
reg_DEFB	chr8	7317239	G	A	50	34,00%	het	17			-	DEFB104A	rs2737576		1,00E-012
reg_DEFB	chr8	7317261	C	A	49	98,00%	homvar	48			-	DEFB104A			1,00E-012
reg_DEFB	chr8	7317363	C	A	44	55,00%	het	24			-	DEFB104A	rs2740011		1,00E-012
reg_DEFB	chr8	7317396	C	G	42	31,00%	het	13			-	DEFB104A	rs2740010		1,74E-011
reg_DEFB	chr8	7317398	G	A	42	31,00%	het	13			-	DEFB104A	rs2740695		1,74E-011
reg_DEFB	chr8	7317588	G	A	27	56,00%	het	15			-	DEFB104A	rs6985641		1,00E-012
reg_DEFB	chr8	7317699	A	G	16	37,00%	het	6			-	DEFB104A	rs28681639		9,72E-007
reg_DEFB	chr8	7317749	T	G	11	55,00%	het	6			-	DEFB104A	rs2740007		6,19E-008
reg_DEFB	chr8	7317813	C	G	11	100,00%	homvar	11			-	DEFB104A	rs7001088		1,00E-012
reg_DEFB	chr8	7318591	C	T	24	29,00%	het	7			-	DEFB104A	rs2680505		8,35E-007
reg_DEFB	chr8	7318756	A	T	33	33,00%	het	11			-	DEFB104A	rs2739991		1,21E-010
reg_DEFB	chr8	7318925	T	C	46	59,00%	het	27			-	DEFB104A	rs2739988		1,00E-012

reg_DEFB	chr8	7319055	T	C	51	55,00%	het	28			-	DEFB104A	rs2739981			1,00E-012
reg_DEFB	chr8	7319063	T	C	53	55,00%	het	29			-	DEFB104A	rs2680506			1,00E-012
reg_DEFB	chr8	7319275	A	T	65	52,00%	het	34			-	DEFB104A	rs2739976			1,00E-012
reg_DEFB	chr8	7319456	T	C	64	42,00%	het	27			-	DEFB104A	rs2739969			1,00E-012
reg_DEFB	chr8	7319532	G	C	69	54,00%	het	37			-	DEFB104A			rs62639768	1,00E-012
reg_DEFB	chr8	7319556	A	G	67	34,00%	het	23			-	DEFB104A			rs2740692	1,00E-012
reg_DEFB	chr8	7319599	C	T	71	51,00%	het	36			-	DEFB104A			rs2739962	1,00E-012
reg_DEFB	chr8	7319638	C	T	63	51,00%	het	32			-	DEFB104A			rs2739960	1,00E-012
reg_DEFB	chr8	7319747	A	G	58	98,00%	homvar	57			-	DEFB104A	rs4259430			1,00E-012
reg_DEFB	chr8	7319771	T	C	64	98,00%	homvar	63			-	DEFB104A			rs17843872	1,00E-012
reg_DEFB	chr8	7319973	T	C	53	45,00%	het	24	I	V	-1	DEFB104B	rs2680507			1,00E-012
reg_DEFB	chr8	7320136	G	C	36	97,00%	homvar	35					rs28590291			1,00E-012
reg_DEFB	chr8	7320153	T	C	23	13,00%	ambig	3								1,53E-002
reg_DEFB	chr8	7320212	C	G	27	96,00%	homvar	26								1,00E-012
reg_DEFB	chr8	7320256	C	T	29	97,00%	homvar	28								1,00E-012
reg_DEFB	chr8	7320337	G	A	36	44,00%	het	16							rs2740691	1,00E-012
reg_DEFB	chr8	7320389	C	T	39	97,00%	homvar	38								1,00E-012
reg_DEFB	chr8	7320465	C	T	43	100,00%	homvar	43								1,00E-012
reg_DEFB	chr8	7320516	A	G	42	100,00%	homvar	42							rs71308312	1,00E-012
reg_DEFB	chr8	7320524	C	G	41	100,00%	homvar	41							rs71308312	1,00E-012
reg_DEFB	chr8	7320543	A	G	42	98,00%	homvar	41								1,00E-012
reg_DEFB	chr8	7320547	A	G	41	100,00%	homvar	41								1,00E-012
reg_DEFB	chr8	7320699	G	A	38	97,00%	homvar	37							rs71272074	1,00E-012
reg_DEFB	chr8	7320772	A	C	28	96,00%	homvar	27					rs2680508			1,00E-012
reg_DEFB	chr8	7320916	A	G	35	100,00%	homvar	35							rs61413127	1,00E-012
reg_DEFB	chr8	7320949	G	A	41	85,00%	homvar	35					rs2740690			1,00E-012
reg_DEFB	chr8	7320971	C	G	44	39,00%	het	17					rs2680509			1,00E-012
reg_DEFB	chr8	7321565	A	G	33	45,00%	het	15					rs2739944			1,00E-012
reg_DEFB	chr8	7321575	T	A	33	94,00%	homvar	31					rs2739943			1,00E-012
reg_DEFB	chr8	7321621	C	T	42	93,00%	homvar	39							rs2680510	1,00E-012
reg_DEFB	chr8	7322171	G	A	45	98,00%	homvar	44							rs2680512	1,00E-012
reg_DEFB	chr8	7322389	A	G	43	98,00%	homvar	42							rs2680515	1,00E-012
reg_DEFB	chr8	7322414	C	T	41	95,00%	homvar	39							rs2680516	1,00E-012
reg_DEFB	chr8	7322882	A	C	31	48,00%	het	15							rs6472800	1,00E-012
reg_DEFB	chr8	7322889	C	T	32	50,00%	het	16							rs4602913	1,00E-012
reg_DEFB	chr8	7322954	C	G	38	97,00%	homvar	37					rs28695206			1,00E-012
reg_DEFB	chr8	7323016	A	G	48	50,00%	het	24							rs2680518	1,00E-012
reg_DEFB	chr8	7323020	A	T	49	92,00%	homvar	45							rs2739908	1,00E-012
reg_DEFB	chr8	7323062	T	A	68	51,00%	het	35							rs2739905	1,00E-012
reg_DEFB	chr8	7323118	A	G	82	49,00%	het	40					rs2737579			1,00E-012
reg_DEFB	chr8	7323491	T	G	79	39,00%	het	31					rs2739894			1,00E-012
reg_DEFB	chr8	7323679	C	T	61	100,00%	homvar	61					rs28557092	rs41405850		1,00E-012

reg_DEFB	chr8	7324031	C	A	56	46,00%	het	26					rs4840760		1,00E-012
reg_DEFB	chr8	7325028	A	G	47	51,00%	het	24					rs6651517		1,00E-012
reg_DEFB	chr8	7325195	C	G	57	39,00%	het	22						rs71247896	1,00E-012
reg_DEFB	chr8	7325767	T	C	42	48,00%	het	20						rs62639773	1,00E-012
reg_DEFB	chr8	7326060	C	T	51	41,00%	het	21					rs2740125		1,00E-012
reg_DEFB	chr8	7326628	C	T	56	100,00%	homvar	56						rs62639774	1,00E-012
reg_DEFB	chr8	7327328	G	A	36	100,00%	homvar	36					rs2293956		1,00E-012
reg_DEFB	chr8	7327333	G	A	37	100,00%	homvar	37							1,00E-012
reg_DEFB	chr8	7327748	G	A	74	54,00%	het	40			-	DEFB106B	rs2244096		1,00E-012
reg_DEFB	chr8	7328290	T	C	50	50,00%	het	25			-	DEFB106B	rs2740081		1,00E-012
reg_DEFB	chr8	7328305	C	A	54	46,00%	het	25			-	DEFB106B		rs62641365	1,00E-012
reg_DEFB	chr8	7328700	C	T	22	100,00%	homvar	22			-	DEFB106B	rs28548808		1,00E-012
reg_DEFB	chr8	7329944	A	T	4	75,00%	het	3			-	DEFB106B	rs2740058		4,78E-005
reg_DEFB	chr8	7330145	A	T	4	100,00%	homvar	4			-	DEFB106B			2,80E-007
reg_DEFB	chr8	7330551	T	C	55	40,00%	het	22			-	DEFB106B	rs6605634		1,00E-012
reg_DEFB	chr8	7330574	C	G	56	37,00%	het	21			-	DEFB106B		rs62639775	1,00E-012
reg_DEFB	chr8	7330881	T	A	78	46,00%	het	36			-	DEFB106B		rs62639776	1,00E-012
reg_DEFB	chr8	7331216	C	G	41	46,00%	het	19			-	DEFB106B	rs2738000		1,00E-012
reg_DEFB	chr8	7331608	C	G	59	100,00%	homvar	59							1,00E-012
reg_DEFB	chr8	7332663	T	A	52	54,00%	het	28	I	N	1	DEFB105B		rs62639778	1,00E-012
reg_DEFB	chr8	7333168	G	C	42	100,00%	homvar	42			+	DEFB105A			1,00E-012
reg_DEFB	chr8	7334564	C	G	46	61,00%	het	28						rs62639779	1,00E-012
reg_DEFB	chr8	7334844	G	C	38	47,00%	het	18						rs62639780	1,00E-012
reg_DEFB	chr8	7334996	A	G	52	48,00%	het	25					rs2737595		1,00E-012
reg_DEFB	chr8	7335121	T	C	59	95,00%	homvar	56							1,00E-012
reg_DEFB	chr8	7335401	A	G	57	100,00%	homvar	57					rs2977418		1,00E-012
reg_DEFB	chr8	7335573	T	C	51	94,00%	homvar	48					rs4481626		1,00E-012
reg_DEFB	chr8	7336570	C	T	33	42,00%	het	14						rs62639783	1,00E-012
reg_DEFB	chr8	7336931	A	C	11	36,00%	het	4					rs2737601		8,11E-005
reg_DEFB	chr8	7337863	A	C	25	100,00%	homvar	25							1,00E-012
reg_DEFB	chr8	7338251	A	C	26	38,00%	het	10					rs2737989		1,61E-010
reg_DEFB	chr8	7338279	T	C	28	100,00%	homvar	28					rs2946448		1,00E-012
reg_DEFB	chr8	7338381	C	T	30	60,00%	het	18					rs2977421		1,00E-012
reg_DEFB	chr8	7338534	G	T	26	50,00%	het	13					rs2737604		1,00E-012
reg_DEFB	chr8	7338734	C	T	31	35,00%	het	11					rs2680559		5,47E-011
reg_DEFB	chr8	7338884	A	G	42	100,00%	homvar	42						rs62639786	1,00E-012
reg_DEFB	chr8	7339447	C	T	39	95,00%	homvar	37						rs62639787	1,00E-012
reg_DEFB	chr8	7339485	C	T	42	93,00%	homvar	39					rs2680561		1,00E-012
reg_DEFB	chr8	7339538	C	G	40	100,00%	homvar	40					rs2737606		1,00E-012
reg_DEFB	chr8	7339872	A	G	40	100,00%	homvar	40					rs2737608		1,00E-012
reg_DEFB	chr8	7339927	A	C	40	100,00%	homvar	40					rs2737609		1,00E-012
reg_DEFB	chr8	7340195	C	A	31	94,00%	homvar	29					rs2737988		1,00E-012

reg_DEFB	chr8	7340261	C	G	24	46,00%	het	11				rs62639790	3,07E-012
reg_DEFB	chr8	7340710	G	A	48	100,00%	homvar	48					1,00E-012
reg_DEFB	chr8	7341096	G	C	35	54,00%	het	19	+	DEFB107A	rs4355796		1,00E-012
reg_DEFB	chr8	7341300	T	C	19	89,00%	homvar	17	+	DEFB107A	rs12682203		1,00E-012
reg_DEFB	chr8	7341317	T	C	18	39,00%	het	7	+	DEFB107A	rs12682205		8,67E-008
reg_DEFB	chr8	7341325	A	G	18	39,00%	het	7	+	DEFB107A	rs12681807		8,67E-008
reg_DEFB	chr8	7341374	C	G	11	45,00%	het	5	+	DEFB107A		rs62639791	2,65E-006
reg_DEFB	chr8	7341377	A	G	11	45,00%	het	5	+	DEFB107A		rs73366541	2,65E-006
reg_DEFB	chr8	7341389	G	T	11	45,00%	het	5	+	DEFB107A	rs12675434		2,65E-006
reg_DEFB	chr8	7341479	T	G	10	50,00%	het	5	+	DEFB107A		rs6471470	1,47E-006
reg_DEFB	chr8	7341618	G	T	32	31,00%	het	10	+	DEFB107A		rs62641362	1,68E-009
reg_DEFB	chr8	7341639	C	T	35	37,00%	het	13	+	DEFB107A		rs62641361	1,00E-012
reg_DEFB	chr8	7342213	G	C	69	52,00%	het	36	+	DEFB107A		rs73199782	1,00E-012
reg_DEFB	chr8	7342346	G	A	67	40,00%	het	27	+	DEFB107A		rs66911494	1,00E-012
reg_DEFB	chr8	7343575	C	T	4	75,00%	het	3	+	DEFB107A		rs4538904	4,78E-005
reg_DEFB	chr8	7343596	C	T	4	100,00%	homvar	4	+	DEFB107A		rs9774358	2,80E-007
reg_DEFB	chr8	7343619	C	T	4	100,00%	homvar	4	+	DEFB107A	rs693031		2,80E-007
reg_DEFB	chr8	7343692	C	T	4	100,00%	homvar	4	+	DEFB107A		rs62515824	2,80E-007
reg_DEFB	chr8	7343696	T	G	4	75,00%	het	3	+	DEFB107A		rs10102892	4,78E-005
reg_DEFB	chr8	7343714	C	T	3	100,00%	homvar	3	+	DEFB107A	rs9720857		1,22E-005
reg_DEFB	chr8	7348303	A	G	11	91,00%	homvar	10	+	DEFB107A			1,00E-012
reg_DEFB	chr8	7348338	C	A	10	100,00%	homvar	10	+	DEFB107A			1,00E-012
reg_DEFB	chr8	7348349	T	C	10	50,00%	het	5	+	DEFB107A			1,47E-006
reg_DEFB	chr8	7348357	A	G	10	80,00%	homvar	8	+	DEFB107A			4,25E-012
reg_DEFB	chr8	7348411	A	C	9	56,00%	het	5	+	DEFB107A			7,51E-007
reg_DEFB	chr8	7348467	C	A	9	89,00%	homvar	8	+	DEFB107A			1,00E-012
reg_DEFB	chr8	7348683	A	G	4	100,00%	homvar	4	+	DEFB107A			2,80E-007
reg_DEFB	chr8	7348763	T	C	4	100,00%	homvar	4	+	DEFB107A			2,80E-007
reg_DEFB	chr8	7348942	T	C	5	80,00%	homvar	4	+	DEFB107A			1,37E-006
reg_DEFB	chr8	7349108	A	G	3	100,00%	homvar	3	+	DEFB107A			1,22E-005
reg_DEFB	chr8	7351384	G	T	3	100,00%	homvar	3	+	DEFB107A			1,22E-005
reg_DEFB	chr8	7351413	T	G	3	100,00%	homvar	3	+	DEFB107A			1,22E-005
reg_DEFB	chr8	7352362	G	T	20	95,00%	homvar	19	+	DEFB107A		rs2737986	1,00E-012
reg_DEFB	chr8	7352697	A	G	58	52,00%	het	30	+	DEFB107A	rs12155781		1,00E-012
reg_DEFB	chr8	7352969	T	C	34	94,00%	homvar	32	+	DEFB107A	rs12155828		1,00E-012
reg_DEFB	chr8	7353182	T	C	48	44,00%	het	21	+	DEFB107A	rs12155887		1,00E-012
reg_DEFB	chr8	7353385	A	G	73	48,00%	het	35	+	DEFB107A	rs2737983		1,00E-012
reg_DEFB	chr8	7353557	C	T	59	54,00%	het	32	+	DEFB107A	rs11775409		1,00E-012
reg_DEFB	chr8	7353946	C	T	57	47,00%	het	27	+	DEFB107A		rs62639796	1,00E-012
reg_DEFB	chr8	7353996	T	C	63	54,00%	het	34	+	DEFB107A	rs2737476		1,00E-012
reg_DEFB	chr8	7354227	T	C	85	99,00%	homvar	84	+	DEFB107A	rs2737477		1,00E-012
reg_DEFB	chr8	7354364	A	G	106	44,00%	het	47			rs4143089		1,00E-012

reg_DEFB	chr8	7354376	T	C	108	44,00%	het	48	rs4143090		1,00E-012
reg_DEFB	chr8	7354477	G	A	114	45,00%	het	51	rs4143091		1,00E-012
reg_DEFB	chr8	7354673	C	G	106	98,00%	homvar	104		rs62639797	1,00E-012
reg_DEFB	chr8	7355739	G	A	17	100,00%	homvar	17		rs71511273	1,00E-012
reg_DEFB	chr8	7356015	G	A	8	75,00%	het	6		rs66488835	3,99E-009
reg_DEFB	chr8	7357088	T	C	88	49,00%	het	43	rs2737481		1,00E-012
reg_DEFB	chr8	7357411	A	C	89	97,00%	homvar	86	rs4840763		1,00E-012
reg_DEFB	chr8	7357553	G	A	70	41,00%	het	29	rs2737979		1,00E-012
reg_DEFB	chr8	7357798	C	T	67	52,00%	het	35		rs2737483	1,00E-012
reg_DEFB	chr8	7357880	T	C	68	99,00%	homvar	67		rs71509114	1,00E-012
reg_DEFB	chr8	7357983	G	A	81	54,00%	het	44		rs2737978	1,00E-012
reg_DEFB	chr8	7358321	C	T	127	51,00%	het	65		rs71511276	1,00E-012
reg_DEFB	chr8	7358618	C	T	109	97,00%	homvar	106			1,00E-012
reg_DEFB	chr8	7358904	G	A	77	99,00%	homvar	76		rs62639798	1,00E-012
reg_DEFB	chr8	7358942	C	T	67	42,00%	het	28	rs2680433		1,00E-012
reg_DEFB	chr8	7359000	G	A	57	100,00%	homvar	57	rs11984588		1,00E-012
reg_DEFB	chr8	7359515	C	A	31	100,00%	homvar	31		rs71239493	1,00E-012
reg_DEFB	chr8	7359657	T	G	32	100,00%	homvar	32		rs62639800	1,00E-012
reg_DEFB	chr8	7360543	A	G	29	10,00%	ambig	3			2,85E-002
reg_DEFB	chr8	7360635	T	G	36	42,00%	het	15		rs71259275	1,00E-012
reg_DEFB	chr8	7361108	A	G	35	49,00%	het	17		rs62639801	1,00E-012
reg_DEFB	chr8	7361125	T	C	31	58,00%	het	18		rs62639802	1,00E-012
reg_DEFB	chr8	7361343	A	C	24	37,00%	het	9			1,72E-009
reg_DEFB	chr8	7361697	C	T	41	46,00%	het	19		rs67213127	1,00E-012
reg_DEFB	chr8	7361701	G	T	42	90,00%	homvar	38		rs62639803	1,00E-012
reg_DEFB	chr8	7361941	A	G	24	42,00%	het	10			6,17E-011
reg_DEFB	chr8	7362037	C	G	24	50,00%	het	12		rs66478539	1,00E-012
reg_DEFB	chr8	7362076	A	G	22	45,00%	het	10		rs71230560	3,38E-011
reg_DEFB	chr8	7362278	G	A	17	47,00%	het	8		rs71230561	1,60E-009
reg_DEFB	chr8	7362561	G	C	22	41,00%	het	9		rs71230562	6,96E-010
reg_DEFB	chr8	7362617	A	G	28	89,00%	homvar	25		rs71213914	1,00E-012
reg_DEFB	chr8	7362619	G	A	29	41,00%	het	12			1,00E-012
reg_DEFB	chr8	7362633	A	G	31	45,00%	het	14		rs71213915	1,00E-012
reg_DEFB	chr8	7362640	A	G	31	45,00%	het	14		rs71213915	1,00E-012
reg_DEFB	chr8	7362978	C	T	36	61,00%	het	22		rs62639805	1,00E-012
reg_DEFB	chr8	7363131	C	T	36	50,00%	het	18		rs66602902	1,00E-012
reg_DEFB	chr8	7363844	A	T	39	59,00%	het	23		rs2737488	1,00E-012
reg_DEFB	chr8	7363941	A	G	36	64,00%	het	23		rs2680438	1,00E-012
reg_DEFB	chr8	7364280	A	G	64	45,00%	het	29		rs71228231	1,00E-012
reg_DEFB	chr8	7364478	C	G	72	99,00%	homvar	71	rs2680440		1,00E-012
reg_DEFB	chr8	7364562	G	T	74	50,00%	het	37		rs4590459	1,00E-012
reg_DEFB	chr8	7364725	T	A	64	50,00%	het	32	rs4556104		1,00E-012

reg_DEFB	chr8	7365153	T	C	53	28,00%	het	15		rs71228230	1,89E-012
reg_DEFB	chr8	7365196	A	G	57	32,00%	het	18		rs67575026	1,00E-012
reg_DEFB	chr8	7365938	G	A	86	47,00%	het	40		rs71511278	1,00E-012
reg_DEFB	chr8	7366130	G	T	72	46,00%	het	33		rs71511279	1,00E-012
reg_DEFB	chr8	7366324	G	A	61	49,00%	het	30	rs4584163		1,00E-012
reg_DEFB	chr8	7366488	A	G	38	42,00%	het	16	rs4263787		1,00E-012
reg_DEFB	chr8	7366494	A	C	38	97,00%	homvar	37	rs4270988		1,00E-012
reg_DEFB	chr8	7366516	C	G	38	45,00%	het	17	rs4446761		1,00E-012
reg_DEFB	chr8	7366531	T	G	38	47,00%	het	18	rs4335141		1,00E-012
reg_DEFB	chr8	7367473	C	A	55	35,00%	het	19	rs4440657		1,00E-012
reg_DEFB	chr8	7367818	T	C	12	100,00%	homvar	12		rs7842766	1,00E-012
reg_DEFB	chr8	7367967	G	A	10	40,00%	het	4			5,26E-005
reg_DEFB	chr8	7368330	G	T	58	50,00%	het	29			1,00E-012
reg_DEFB	chr8	7368407	T	G	71	48,00%	het	34		rs4599836	1,00E-012
reg_DEFB	chr8	7368542	T	A	73	55,00%	het	40		rs71511292	1,00E-012
reg_DEFB	chr8	7369171	A	G	11	27,00%	het	3			1,75E-003
reg_DEFB	chr8	7369173	T	C	11	27,00%	het	3			1,75E-003
reg_DEFB	chr8	7369180	T	C	11	27,00%	het	3			1,75E-003
reg_DEFB	chr8	7369184	T	A	11	27,00%	het	3			1,75E-003
reg_DEFB	chr8	7369708	C	T	84	46,00%	het	39	rs725058		1,00E-012
reg_DEFB	chr8	7370010	A	G	67	40,00%	het	27			1,00E-012
reg_DEFB	chr8	7370028	T	G	60	45,00%	het	27	rs2680484		1,00E-012
reg_DEFB	chr8	7370060	T	C	61	39,00%	het	24			1,00E-012
reg_DEFB	chr8	7370074	C	A	57	51,00%	het	29		rs62639806	1,00E-012
reg_DEFB	chr8	7370610	A	G	82	40,00%	het	33		rs2680482	1,00E-012
reg_DEFB	chr8	7371475	C	T	59	41,00%	het	24	rs4840766		1,00E-012
reg_DEFB	chr8	7372793	T	C	88	43,00%	het	38			1,00E-012
reg_DEFB	chr8	7373254	C	T	27	59,00%	het	16	rs1807385		1,00E-012
reg_DEFB	chr8	7374467	T	C	54	44,00%	het	24			1,00E-012
reg_DEFB	chr8	7375518	G	A	49	45,00%	het	22	rs4840769		1,00E-012
reg_DEFB	chr8	7375596	C	A	45	49,00%	het	22	rs11786478		1,00E-012
reg_DEFB	chr8	7375818	C	T	30	30,00%	het	9		rs71264915	1,66E-008
reg_DEFB	chr8	7376276	A	C	43	42,00%	het	18			1,00E-012
reg_DEFB	chr8	7376945	T	G	29	45,00%	het	13		rs71249123	1,00E-012
reg_DEFB	chr8	7377165	A	G	25	48,00%	het	12		rs71249124	1,00E-012
reg_DEFB	chr8	7377464	C	A	28	100,00%	homvar	28			1,00E-012
reg_DEFB	chr8	7378147	C	T	23	48,00%	het	11	rs2977689		1,66E-012
reg_DEFB	chr8	7378654	T	C	13	100,00%	homvar	13		rs71249125	1,00E-012
reg_DEFB	chr8	7379026	G	A	11	91,00%	homvar	10		rs67847292	1,00E-012
reg_DEFB	chr8	7379048	T	C	11	100,00%	homvar	11		rs71249126	1,00E-012
reg_DEFB	chr8	7379519	C	T	15	27,00%	het	4		rs62639811	3,12E-004
reg_DEFB	chr8	7380428	G	A	41	100,00%	homvar	41			1,00E-012

reg_DEFB	chr8	7380733	A	G	68	53,00%	het	36	rs2954331			1,00E-012
reg_DEFB	chr8	7380984	G	C	89	46,00%	het	41	rs2977404			1,00E-012
reg_DEFB	chr8	7381451	A	G	65	48,00%	het	31	rs2977687			1,00E-012
reg_DEFB	chr8	7381476	G	A	60	97,00%	homvar	58	rs2737939			1,00E-012
reg_DEFB	chr8	7381963	G	A	36	31,00%	het	11	rs4311672			3,56E-010
reg_DEFB	chr8	7382091	A	C	42	90,00%	homvar	38	rs2737936			1,00E-012
reg_DEFB	chr8	7382305	C	G	45	38,00%	het	17	rs2737499			1,00E-012
reg_DEFB	chr8	7382406	C	A	36	50,00%	het	18	rs4461922			1,00E-012
reg_DEFB	chr8	7382462	C	T	46	52,00%	het	24	rs4633079			1,00E-012
reg_DEFB	chr8	7382473	T	G	45	51,00%	het	23			rs62639815	1,00E-012
reg_DEFB	chr8	7382556	T	C	42	55,00%	het	23			rs62639816	1,00E-012
reg_DEFB	chr8	7382833	G	A	77	100,00%	homvar	77				1,00E-012
reg_DEFB	chr8	7383272	A	C	65	54,00%	het	35			rs67373661	1,00E-012
reg_DEFB	chr8	7383406	C	T	58	55,00%	het	32	rs4504661			1,00E-012
reg_DEFB	chr8	7383498	T	C	52	42,00%	het	22	rs4392927			1,00E-012
reg_DEFB	chr8	7383738	A	G	50	52,00%	het	26	rs2977686			1,00E-012
reg_DEFB	chr8	7383983	A	G	43	51,00%	het	22	rs2977685			1,00E-012
reg_DEFB	chr8	7384348	A	C	89	60,00%	het	53	rs2737932			1,00E-012
reg_DEFB	chr8	7385749	G	A	49	55,00%	het	27	rs2737931			1,00E-012
reg_DEFB	chr8	7385987	T	C	54	44,00%	het	24	rs2946446			1,00E-012
reg_DEFB	chr8	7386060	T	A	47	47,00%	het	22			rs62639820	1,00E-012
reg_DEFB	chr8	7386182	T	G	54	54,00%	het	29	rs2737502			1,00E-012
reg_DEFB	chr8	7386319	T	A	55	64,00%	het	35			rs62639821	1,00E-012
reg_DEFB	chr8	7386396	G	A	56	66,00%	het	37			rs62639822	1,00E-012
reg_DEFB	chr8	7386594	C	T	58	24,00%	ambig	14	rs2017780			5,05E-011
reg_DEFB	chr8	7386641	T	C	50	72,00%	het	36	rs2737503			1,00E-012
reg_DEFB	chr8	7386669	A	G	46	65,00%	het	30			rs62639824	1,00E-012
reg_DEFB	chr8	7386935	A	G	76	50,00%	het	38			rs62639825	1,00E-012
reg_DEFB	chr8	7387038	C	A	90	44,00%	het	40	rs3175182			1,00E-012
reg_DEFB	chr8	7387328	T	G	83	46,00%	het	38	rs4840284			1,00E-012
reg_DEFB	chr8	7387420	C	G	68	44,00%	het	30	rs2258428			1,00E-012
reg_DEFB	chr8	7388003	A	G	26	46,00%	het	12	rs2737508			1,00E-012
reg_DEFB	chr8	7388132	C	G	27	48,00%	het	13	rs2946444			1,00E-012
reg_DEFB	chr8	7388276	T	C	26	12,00%	ambig	3				2,13E-002
reg_DEFB	chr8	7389499	A	G	72	50,00%	het	36	rs2075896			1,00E-012
reg_DEFB	chr8	7390072	C	G	87	51,00%	het	44	rs2946443			1,00E-012
reg_DEFB	chr8	7390501	T	A	100	47,00%	het	47	rs4840774	rs2737514		1,00E-012
reg_DEFB	chr8	7390503	C	G	101	50,00%	het	51	rs4840775			1,00E-012
reg_DEFB	chr8	7390657	G	A	83	59,00%	het	49			rs2737889	1,00E-012
reg_DEFB	chr8	7390776	G	A	79	96,00%	homvar	76	rs2737515			1,00E-012
reg_DEFB	chr8	7390893	A	T	63	43,00%	het	27			rs62639831	1,00E-012
reg_DEFB	chr8	7391064	T	G	39	100,00%	homvar	39	rs4840776			1,00E-012

reg_DEFB	chr8	7391199	G	C	14	29,00%	het	4					rs2737885			2,33E-004
reg_DEFB	chr8	7391201	A	T	14	64,00%	het	9							rs2954332	4,45E-012
reg_CTRL	chr8	8213964	C	T	18	44,00%	het	8	A	T	-1	PRAGMIN	rs12549973			2,79E-009
reg_CTRL	chr8	8214235	A	G	58	31,00%	het	18			-	PRAGMIN	rs6601691			1,00E-012
reg_CTRL	chr8	8214527	A	G	68	49,00%	het	33			-	PRAGMIN	rs6996347			1,00E-012
reg_CTRL	chr8	8214897	A	C	74	54,00%	het	40			-	PRAGMIN	rs4840922			1,00E-012
reg_CTRL	chr8	8215271	G	A	68	44,00%	het	30			-	PRAGMIN	rs2980503			1,00E-012
reg_CTRL	chr8	8215510	T	C	78	54,00%	het	42			-	PRAGMIN	rs13276576			1,00E-012
reg_CTRL	chr8	8215633	T	C	87	100,00%	homvar	87			-	PRAGMIN	rs1548198			1,00E-012
reg_CTRL	chr8	8217059	C	G	31	55,00%	het	17			-	PRAGMIN	rs4840923			1,00E-012
reg_CTRL	chr8	8217969	G	C	61	48,00%	het	29			-	PRAGMIN	rs2102866			1,00E-012
reg_CTRL	chr8	8218156	G	A	32	56,00%	het	18			-	PRAGMIN	rs12674854			1,00E-012
reg_CTRL	chr8	8218928	G	C	86	44,00%	het	38			-	PRAGMIN	rs867757			1,00E-012
reg_CTRL	chr8	8218968	A	T	89	56,00%	het	50			-	PRAGMIN	rs2945898			1,00E-012
reg_CTRL	chr8	8219680	G	C	54	48,00%	het	26			-	PRAGMIN	rs939069			1,00E-012
reg_CTRL	chr8	8221093	T	C	8	50,00%	het	4			-	PRAGMIN	rs2945899			1,82E-005
reg_CTRL	chr8	8223577	T	C	55	45,00%	het	25			-	PRAGMIN	rs2979208			1,00E-012
reg_CTRL	chr8	8223747	A	T	58	57,00%	het	33			-	PRAGMIN	rs2976910			1,00E-012
reg_CTRL	chr8	8223842	C	T	53	41,00%	het	22			-	PRAGMIN	rs10099374			1,00E-012
reg_CTRL	chr8	8224196	A	G	38	11,00%	ambig	4			-	PRAGMIN				1,11E-002
reg_CTRL	chr8	8224207	G	T	37	11,00%	ambig	4			-	PRAGMIN				1,01E-002
reg_CTRL	chr8	8224229	G	T	38	29,00%	het	11			-	PRAGMIN	rs2945900			6,48E-010
reg_CTRL	chr8	8224912	A	G	82	56,00%	het	46			-	PRAGMIN	rs2979210			1,00E-012
reg_CTRL	chr8	8224960	T	C	76	57,00%	het	43			-	PRAGMIN	rs2945901			1,00E-012
reg_CTRL	chr8	8225163	A	G	41	41,00%	het	17			-	PRAGMIN	rs2980498	rs2945902		1,00E-012
reg_CTRL	chr8	8225164	T	C	41	41,00%	het	17			-	PRAGMIN	rs2945902			1,00E-012
reg_CTRL	chr8	8225230	T	C	45	49,00%	het	22			-	PRAGMIN	rs2945903			1,00E-012
reg_CTRL	chr8	8225641	G	A	78	50,00%	het	39			-	PRAGMIN	rs2979211			1,00E-012
reg_CTRL	chr8	8225874	G	A	87	55,00%	het	48			-	PRAGMIN	rs2979212			1,00E-012
reg_CTRL	chr8	8225954	G	A	93	56,00%	het	52			-	PRAGMIN	rs2979213			1,00E-012
reg_CTRL	chr8	8225986	A	G	98	57,00%	het	56			-	PRAGMIN	rs2976917			1,00E-012
reg_CTRL	chr8	8226005	A	G	97	53,00%	het	51			-	PRAGMIN	rs2976918			1,00E-012
reg_CTRL	chr8	8226193	A	G	100	55,00%	het	55			-	PRAGMIN	rs2979215			1,00E-012
reg_CTRL	chr8	8226315	T	C	101	48,00%	het	48			-	PRAGMIN	rs2979216			1,00E-012
reg_CTRL	chr8	8226494	G	A	100	26,00%	het	26			-	PRAGMIN	rs2979217			1,00E-012
reg_CTRL	chr8	8226774	G	C	87	41,00%	het	36			-	PRAGMIN	rs2979218			1,00E-012
reg_CTRL	chr8	8226802	G	A	82	44,00%	het	36			-	PRAGMIN	rs2979219			1,00E-012
reg_CTRL	chr8	8226809	A	G	81	44,00%	het	36			-	PRAGMIN	rs2976927			1,00E-012
reg_CTRL	chr8	8226848	T	C	84	100,00%	homvar	84			-	PRAGMIN	rs2980497			1,00E-012
reg_CTRL	chr8	8226912	G	A	86	44,00%	het	38			-	PRAGMIN	rs2945904			1,00E-012
reg_CTRL	chr8	8226996	T	G	71	48,00%	het	34			-	PRAGMIN	rs2945905			1,00E-012
reg_CTRL	chr8	8227475	T	C	43	51,00%	het	22			-	PRAGMIN	rs2945907			1,00E-012

reg_CTRL	chr8	8227809	T	G	90	34,00%	het	31	-	PRAGMIN	rs1518992		1,00E-012
reg_CTRL	chr8	8227825	T	C	87	34,00%	het	30	-	PRAGMIN	rs1850724		1,00E-012
reg_CTRL	chr8	8227900	C	G	98	40,00%	het	39	-	PRAGMIN	rs1850725		1,00E-012
reg_CTRL	chr8	8227975	A	G	99	41,00%	het	41	-	PRAGMIN	rs1850726		1,00E-012
reg_CTRL	chr8	8228199	T	C	88	36,00%	het	32	-	PRAGMIN	rs2980496		1,00E-012
reg_CTRL	chr8	8228481	T	G	122	50,00%	het	61	-	PRAGMIN	rs2979220		1,00E-012
reg_CTRL	chr8	8229144	C	T	30	60,00%	het	18	-	PRAGMIN	rs2980495		1,00E-012
reg_CTRL	chr8	8229293	T	C	22	45,00%	het	10	-	PRAGMIN	rs2979221		3,38E-011
reg_CTRL	chr8	8229301	G	A	21	43,00%	het	9	-	PRAGMIN	rs2976947		4,19E-010
reg_CTRL	chr8	8229490	C	T	54	52,00%	het	28	-	PRAGMIN			1,00E-012
reg_CTRL	chr8	8229639	A	C	54	48,00%	het	26	-	PRAGMIN	rs2945908		1,00E-012
reg_CTRL	chr8	8229935	T	C	32	47,00%	het	15	-	PRAGMIN	rs2976952		1,00E-012
reg_CTRL	chr8	8230241	T	C	48	50,00%	het	24	-	PRAGMIN	rs2976954		1,00E-012
reg_CTRL	chr8	8230250	A	C	47	51,00%	het	24	-	PRAGMIN	rs2979222		1,00E-012
reg_CTRL	chr8	8230631	T	C	21	33,00%	het	7	-	PRAGMIN	rs2980494		2,98E-007
reg_CTRL	chr8	8230864	G	A	39	100,00%	homvar	39	-	PRAGMIN	rs2979223		1,00E-012
reg_CTRL	chr8	8231138	C	T	86	52,00%	het	45	-	PRAGMIN	rs2979224		1,00E-012
reg_CTRL	chr8	8231195	T	G	83	47,00%	het	39	-	PRAGMIN	rs2976963		1,00E-012
reg_CTRL	chr8	8232030	A	C	28	50,00%	het	14	-	PRAGMIN	rs2979226		1,00E-012
reg_CTRL	chr8	8232156	C	G	50	50,00%	het	25	-	PRAGMIN	rs9329270		1,00E-012
reg_CTRL	chr8	8232408	G	A	85	58,00%	het	49	-	PRAGMIN	rs2976840		1,00E-012
reg_CTRL	chr8	8232580	A	C	72	47,00%	het	34	-	PRAGMIN	rs2945910		1,00E-012
reg_CTRL	chr8	8233348	C	T	102	49,00%	het	50	-	PRAGMIN	rs2976852		1,00E-012
reg_CTRL	chr8	8233599	T	C	128	48,00%	het	61	-	PRAGMIN	rs2980491		1,00E-012
reg_CTRL	chr8	8235132	A	G	111	40,00%	het	44	-	PRAGMIN	rs2945912		1,00E-012
reg_CTRL	chr8	8235635	C	T	80	44,00%	het	35	-	PRAGMIN	rs2945913		1,00E-012
reg_CTRL	chr8	8235716	C	G	84	58,00%	het	49	-	PRAGMIN	rs4840337		1,00E-012
reg_CTRL	chr8	8235760	T	G	84	44,00%	het	37	-	PRAGMIN	rs2980490		1,00E-012
reg_CTRL	chr8	8236281	C	T	72	42,00%	het	30	-	PRAGMIN	rs2976887		1,00E-012
reg_CTRL	chr8	8236848	A	C	9	100,00%	homvar	9	-	PRAGMIN	rs2945914		1,00E-012
reg_CTRL	chr8	8237374	G	A	25	52,00%	het	13	-	PRAGMIN		rs62496027	1,00E-012
reg_CTRL	chr8	8238677	A	G	37	100,00%	homvar	37	-	PRAGMIN	rs2176631		1,00E-012
reg_CTRL	chr8	8238782	A	G	35	60,00%	het	21	-	PRAGMIN	rs2945839		1,00E-012
reg_CTRL	chr8	8239704	A	G	54	37,00%	het	20	-	PRAGMIN	rs2980489		1,00E-012
reg_CTRL	chr8	8242420	C	T	87	99,00%	homvar	86	-	PRAGMIN	rs11785239		1,00E-012
reg_CTRL	chr8	8242644	A	T	65	52,00%	het	34	-	PRAGMIN	rs13273161		1,00E-012
reg_CTRL	chr8	8243226	C	T	95	100,00%	homvar	95	-	PRAGMIN	rs6990504		1,00E-012
reg_CTRL	chr8	8244163	A	G	17	100,00%	homvar	17	-	PRAGMIN	rs7833103		1,00E-012
reg_CTRL	chr8	8244749	A	G	58	43,00%	het	25	-	PRAGMIN	rs17150353		1,00E-012
reg_CTRL	chr8	8246169	G	C	44	50,00%	het	22	-	PRAGMIN	rs11786306		1,00E-012
reg_CTRL	chr8	8246601	G	T	96	100,00%	homvar	96	-	PRAGMIN	rs4840932		1,00E-012
reg_CTRL	chr8	8249567	T	C	49	65,00%	het	32	-	PRAGMIN	rs34796521		1,00E-012

reg_CTRL	chr8	8252759	T	C	74	45,00%	het	33			-	PRAGMIN	rs4840939		1,00E-012
reg_CTRL	chr8	8254396	A	G	66	100,00%	homvar	66			-	PRAGMIN	rs4840941		1,00E-012
reg_CTRL	chr8	8254926	C	T	48	50,00%	het	24			-	PRAGMIN	rs11778125		1,00E-012
reg_CTRL	chr8	8256007	T	C	61	100,00%	homvar	61			-	PRAGMIN	rs7005904		1,00E-012
reg_CTRL	chr8	8256108	G	C	50	44,00%	het	22			-	PRAGMIN	rs10099225		1,00E-012
reg_CTRL	chr8	8256279	T	C	36	58,00%	het	21			-	PRAGMIN	rs7006376		1,00E-012
reg_CTRL	chr8	8256377	A	G	43	98,00%	homvar	42			-	PRAGMIN	rs4840338		1,00E-012
reg_CTRL	chr8	8256592	G	A	74	100,00%	homvar	74			-	PRAGMIN	rs724265		1,00E-012
reg_CTRL	chr8	8256759	G	A	82	45,00%	het	37			-	PRAGMIN	rs724266		1,00E-012
reg_CTRL	chr8	8258014	C	T	7	43,00%	het	3			-	PRAGMIN	rs28578995		3,97E-004
reg_CTRL	chr8	8258498	C	T	20	45,00%	het	9			-	PRAGMIN	rs1914826		2,44E-010
reg_CTRL	chr8	8258721	C	G	18	50,00%	het	9			-	PRAGMIN	rs1914825		7,33E-011
reg_CTRL	chr8	8258769	G	A	26	46,00%	het	12			-	PRAGMIN	rs1914824		1,00E-012
reg_CTRL	chr8	8259890	T	C	72	100,00%	homvar	72			-	PRAGMIN	rs2030279		1,00E-012
reg_CTRL	chr8	8260359	A	G	25	12,00%	ambig	3			-	PRAGMIN			1,92E-002
reg_CTRL	chr8	8262672	A	G	8	100,00%	homvar	8			-	PRAGMIN	rs13282599		1,00E-012
reg_CTRL	chr8	8269101	A	G	121	99,00%	homvar	120			-	PRAGMIN	rs13280051		1,00E-012
reg_CTRL	chr8	8272061	A	G	18	17,00%	ambig	3	V	A	-1	PRAGMIN			7,66E-003
reg_CTRL	chr8	8273641	C	T	19	95,00%	homvar	18			-	PRAGMIN	rs34742161		1,00E-012
reg_CTRL	chr8	8274087	C	T	49	100,00%	homvar	49			-	PRAGMIN	rs2976958		1,00E-012
reg_CTRL	chr8	8274368	T	C	107	42,00%	het	45			-	PRAGMIN			1,00E-012
reg_CTRL	chr8	8679890	G	A	62	98,00%	homvar	61			-	MFHAS1	rs4841038		1,00E-012
reg_CTRL	chr8	8680797	G	A	23	100,00%	homvar	23			-	MFHAS1	rs10903311		1,00E-012
reg_CTRL	chr8	8680992	C	G	71	100,00%	homvar	71			-	MFHAS1	rs2271340		1,00E-012
reg_CTRL	chr8	8681135	T	A	119	100,00%	homvar	119			-	MFHAS1	rs2271341		1,00E-012
reg_CTRL	chr8	8681348	C	T	116	99,00%	homvar	115			-	MFHAS1	rs2271342		1,00E-012
reg_CTRL	chr8	8681684	G	C	39	95,00%	homvar	37			-	MFHAS1	rs12677543		1,00E-012
reg_CTRL	chr8	8681732	G	A	41	98,00%	homvar	40			-	MFHAS1	rs12677550		1,00E-012
reg_CTRL	chr8	8682101	T	C	39	97,00%	homvar	38			-	MFHAS1	rs7015606		1,00E-012
reg_CTRL	chr8	8682217	A	G	36	56,00%	het	20			-	MFHAS1	rs7010952		1,00E-012
reg_CTRL	chr8	8682221	T	C	38	53,00%	het	20			-	MFHAS1		rs61853364	1,00E-012
reg_CTRL	chr8	8683135	G	C	116	99,00%	homvar	115			-	MFHAS1	rs2409088		1,00E-012
reg_CTRL	chr8	8683656	T	C	52	100,00%	homvar	52			-	MFHAS1	rs12682352		1,00E-012
reg_CTRL	chr8	8686807	T	C	67	100,00%	homvar	67			-	MFHAS1	rs2409089		1,00E-012
reg_CTRL	chr8	8687291	C	T	35	97,00%	homvar	34			-	MFHAS1	rs11249891		1,00E-012
reg_CTRL	chr8	8688829	T	C	60	97,00%	homvar	58			-	MFHAS1	rs6601732		1,00E-012
reg_CTRL	chr8	8690299	G	A	68	99,00%	homvar	67			-	MFHAS1	rs7832968		1,00E-012
reg_CTRL	chr8	8691268	G	A	63	100,00%	homvar	63			-	MFHAS1	rs2409090		1,00E-012
reg_CTRL	chr8	8691467	G	A	11	100,00%	homvar	11			-	MFHAS1	rs2409091		1,00E-012
reg_CTRL	chr8	8691521	C	G	6	100,00%	homvar	6			-	MFHAS1	rs7460947		1,48E-010
reg_CTRL	chr8	8691687	G	A	5	80,00%	homvar	4			-	MFHAS1		rs71576013	1,37E-006
reg_CTRL	chr8	8691772	T	C	12	92,00%	homvar	11			-	MFHAS1			1,00E-012

reg_CTRL	chr8	8691951	C	G	25	96,00%	homvar	24	-	MFHAS1	rs4841041		1,00E-012
reg_CTRL	chr8	8692433	C	T	46	100,00%	homvar	46	-	MFHAS1	rs3748144		1,00E-012
reg_CTRL	chr8	8695950	A	G	41	95,00%	homvar	39	-	MFHAS1	rs2048419		1,00E-012
reg_CTRL	chr8	8697085	G	T	133	100,00%	homvar	133	-	MFHAS1	rs13282015		1,00E-012
reg_CTRL	chr8	8697948	C	A	76	97,00%	homvar	74	-	MFHAS1	rs6994038		1,00E-012
reg_CTRL	chr8	8698944	T	C	73	100,00%	homvar	73	-	MFHAS1	rs12547493		1,00E-012
reg_CTRL	chr8	8699091	C	G	99	100,00%	homvar	99	-	MFHAS1	rs12544992		1,00E-012
reg_CTRL	chr8	8700625	C	T	28	79,00%	homvar	22	-	MFHAS1	rs28399241		1,00E-012
reg_CTRL	chr8	8701507	C	T	42	100,00%	homvar	42	-	MFHAS1	rs9329167		1,00E-012
reg_CTRL	chr8	8701722	C	A	17	88,00%	homvar	15	-	MFHAS1	rs7015271		1,00E-012
reg_CTRL	chr8	8701932	C	A	35	11,00%	ambig	4	-	MFHAS1			8,30E-003
reg_CTRL	chr8	8702032	G	A	41	100,00%	homvar	41	-	MFHAS1	rs4841042		1,00E-012
reg_CTRL	chr8	8702089	G	A	59	90,00%	homvar	53	-	MFHAS1	rs4841043		1,00E-012
reg_CTRL	chr8	8702110	G	A	60	10,00%	ambig	6	-	MFHAS1			2,56E-003
reg_CTRL	chr8	8702350	G	A	83	98,00%	homvar	81	-	MFHAS1	rs4841044		1,00E-012
reg_CTRL	chr8	8702557	G	A	88	97,00%	homvar	85	-	MFHAS1	rs11783966		1,00E-012
reg_CTRL	chr8	8703143	T	A	43	95,00%	homvar	41	-	MFHAS1	rs9644775		1,00E-012
reg_CTRL	chr8	8703212	T	C	36	61,00%	het	22	-	MFHAS1	rs9644776		1,00E-012
reg_CTRL	chr8	8704326	C	T	75	99,00%	homvar	74	-	MFHAS1	rs6988939		1,00E-012
reg_CTRL	chr8	8704329	G	T	76	97,00%	homvar	74	-	MFHAS1	rs2175161		1,00E-012
reg_CTRL	chr8	8704854	C	T	70	100,00%	homvar	70	-	MFHAS1	rs6993494		1,00E-012
reg_CTRL	chr8	8705807	A	G	84	100,00%	homvar	84	-	MFHAS1	rs7006418		1,00E-012
reg_CTRL	chr8	8705896	A	G	84	100,00%	homvar	84	-	MFHAS1	rs7006589		1,00E-012
reg_CTRL	chr8	8706327	A	C	69	100,00%	homvar	69	-	MFHAS1	rs1473029		1,00E-012
reg_CTRL	chr8	8707492	G	C	85	100,00%	homvar	85	-	MFHAS1	rs4840362		1,00E-012
reg_CTRL	chr8	8707587	T	A	64	100,00%	homvar	64	-	MFHAS1	rs7823757		1,00E-012
reg_CTRL	chr8	8708009	A	G	21	100,00%	homvar	21	-	MFHAS1		rs60315134	1,00E-012
reg_CTRL	chr8	8708146	C	A	31	100,00%	homvar	31	-	MFHAS1		rs59046059	1,00E-012
reg_CTRL	chr8	8709372	C	T	65	100,00%	homvar	65	-	MFHAS1	rs11784052		1,00E-012
reg_CTRL	chr8	8709629	T	A	49	86,00%	homvar	42	-	MFHAS1	rs10088933		1,00E-012
reg_CTRL	chr8	8709839	G	C	15	93,00%	homvar	14	-	MFHAS1	rs11777085		1,00E-012
reg_CTRL	chr8	8709989	A	G	25	100,00%	homvar	25	-	MFHAS1	rs4841045		1,00E-012
reg_CTRL	chr8	8710211	C	T	11	91,00%	homvar	10	-	MFHAS1	rs4841046		1,00E-012
reg_CTRL	chr8	8710362	A	C	20	90,00%	homvar	18	-	MFHAS1	rs4841047		1,00E-012
reg_CTRL	chr8	8710730	T	C	109	99,00%	homvar	108	-	MFHAS1	rs13265731		1,00E-012
reg_CTRL	chr8	8711011	A	C	129	99,00%	homvar	128	-	MFHAS1	rs13259216		1,00E-012
reg_CTRL	chr8	8711146	T	C	107	98,00%	homvar	105	-	MFHAS1	rs35431455		1,00E-012
reg_CTRL	chr8	8712586	A	G	41	100,00%	homvar	41	-	MFHAS1	rs13260419		1,00E-012
reg_CTRL	chr8	8712735	A	T	58	100,00%	homvar	58	-	MFHAS1	rs35039922		1,00E-012
reg_CTRL	chr8	8713900	T	C	60	47,00%	het	28	-	MFHAS1	rs950721		1,00E-012
reg_CTRL	chr8	8714251	T	C	22	14,00%	ambig	3	-	MFHAS1			1,35E-002
reg_CTRL	chr8	8714960	C	T	36	36,00%	het	13	-	MFHAS1	rs13280206		1,00E-012

reg_CTRL	chr8	8715940	G	A	90	100,00%	homvar	90	-	MFHAS1	rs882462		1,00E-012
reg_CTRL	chr8	8716586	A	G	145	100,00%	homvar	145	-	MFHAS1	rs11775523		1,00E-012
reg_CTRL	chr8	8716735	G	C	110	97,00%	homvar	107	-	MFHAS1	rs28755903		1,00E-012
reg_CTRL	chr8	8716866	G	C	79	99,00%	homvar	78	-	MFHAS1	rs1039913		1,00E-012
reg_CTRL	chr8	8716959	C	T	73	99,00%	homvar	72	-	MFHAS1	rs1039914		1,00E-012
reg_CTRL	chr8	8717024	T	C	56	98,00%	homvar	55	-	MFHAS1	rs1039915		1,00E-012
reg_CTRL	chr8	8717493	G	A	10	90,00%	homvar	9	-	MFHAS1	rs11779585		1,00E-012
reg_CTRL	chr8	8717887	G	A	65	94,00%	homvar	61	-	MFHAS1		rs57312668	1,00E-012
reg_CTRL	chr8	8718276	C	G	71	100,00%	homvar	71	-	MFHAS1	rs4840364		1,00E-012
reg_CTRL	chr8	8718775	A	C	75	96,00%	homvar	72	-	MFHAS1	rs4841049		1,00E-012
reg_CTRL	chr8	8719000	T	C	82	100,00%	homvar	82	-	MFHAS1	rs4841050		1,00E-012
reg_CTRL	chr8	8719166	T	C	86	100,00%	homvar	86	-	MFHAS1	rs1876836		1,00E-012
reg_CTRL	chr8	8719602	A	T	81	99,00%	homvar	80	-	MFHAS1	rs2409092		1,00E-012
reg_CTRL	chr8	8720288	T	C	22	100,00%	homvar	22	-	MFHAS1	rs12545499		1,00E-012
reg_CTRL	chr8	8720310	G	C	24	100,00%	homvar	24	-	MFHAS1	rs2409094		1,00E-012
reg_CTRL	chr8	8720681	A	C	58	100,00%	homvar	58	-	MFHAS1	rs907179		1,00E-012
reg_CTRL	chr8	8720696	G	C	60	43,00%	het	26	-	MFHAS1	rs34731491		1,00E-012
reg_CTRL	chr8	8722363	G	A	86	100,00%	homvar	86	-	MFHAS1	rs1533059		1,00E-012
reg_CTRL	chr8	8722600	A	G	46	100,00%	homvar	46	-	MFHAS1	rs1533058		1,00E-012
reg_CTRL	chr8	8723056	T	C	90	100,00%	homvar	90	-	MFHAS1	rs4841051		1,00E-012
reg_CTRL	chr8	8723264	A	G	126	100,00%	homvar	126	-	MFHAS1	rs1039916		1,00E-012
reg_CTRL	chr8	8724090	T	A	88	98,00%	homvar	86	-	MFHAS1	rs2409095		1,00E-012
reg_CTRL	chr8	8724464	G	C	38	95,00%	homvar	36	-	MFHAS1	rs3789849		1,00E-012
reg_CTRL	chr8	8726598	A	G	29	10,00%	ambig	3	-	MFHAS1			2,85E-002
reg_CTRL	chr8	8726960	G	A	48	67,00%	het	32	-	MFHAS1			1,00E-012
reg_CTRL	chr8	8726976	G	A	20	95,00%	homvar	19	-	MFHAS1			1,00E-012
reg_CTRL	chr8	8726978	G	A	20	95,00%	homvar	19	-	MFHAS1			1,00E-012
reg_CTRL	chr8	8726980	G	A	20	95,00%	homvar	19	-	MFHAS1			1,00E-012
reg_CTRL	chr8	8726982	G	A	20	95,00%	homvar	19	-	MFHAS1	rs28821557		1,00E-012
reg_CTRL	chr8	8727010	G	A	21	33,00%	het	7	-	MFHAS1		rs71949256	2,98E-007
reg_CTRL	chr8	8727376	G	C	83	90,00%	homvar	75	-	MFHAS1	rs4840366		1,00E-012
reg_CTRL	chr8	8727803	G	T	48	96,00%	homvar	46	-	MFHAS1	rs11995244		1,00E-012
reg_CTRL	chr8	8727836	T	C	48	100,00%	homvar	48	-	MFHAS1	rs13259619		1,00E-012
reg_CTRL	chr8	8728197	C	T	56	98,00%	homvar	55	-	MFHAS1	rs13259070		1,00E-012
reg_CTRL	chr8	8728228	C	A	59	41,00%	het	24	-	MFHAS1			1,00E-012
reg_CTRL	chr8	8728794	C	G	42	98,00%	homvar	41	-	MFHAS1	rs9329169		1,00E-012
reg_CTRL	chr8	8729032	T	A	42	100,00%	homvar	42	-	MFHAS1	rs13270070		1,00E-012
reg_CTRL	chr8	8729476	G	C	93	100,00%	homvar	93	-	MFHAS1	rs1510932		1,00E-012
reg_CTRL	chr8	8729887	T	C	69	91,00%	homvar	63	-	MFHAS1	rs2409096		1,00E-012
reg_CTRL	chr8	8729950	C	G	63	98,00%	homvar	62	-	MFHAS1	rs1510933		1,00E-012
reg_CTRL	chr8	8731603	C	G	55	96,00%	homvar	53	-	MFHAS1	rs13254903		1,00E-012
reg_CTRL	chr8	8732984	C	T	98	100,00%	homvar	98	-	MFHAS1	rs6601265		1,00E-012

reg_CTRL	chr8	8733859	T	G	51	96,00%	homvar	49	-	MFHAS1	rs1510934		1,00E-012
reg_CTRL	chr8	8734939	C	A	99	42,00%	het	42	-	MFHAS1	rs36104437		1,00E-012
reg_CTRL	chr8	8735502	C	T	85	98,00%	homvar	83	-	MFHAS1	rs4841054		1,00E-012
reg_CTRL	chr8	8736571	A	G	78	95,00%	homvar	74	-	MFHAS1	rs4841055		1,00E-012
reg_CTRL	chr8	8737167	T	A	41	93,00%	homvar	38	-	MFHAS1	rs7820146		1,00E-012
reg_CTRL	chr8	8737171	C	T	41	100,00%	homvar	41	-	MFHAS1	rs7833171		1,00E-012
reg_CTRL	chr8	8738012	G	C	33	30,00%	het	10	-	MFHAS1	rs7017006		2,37E-009
reg_CTRL	chr8	8738114	C	G	36	44,00%	het	16	-	MFHAS1		rs4752439	1,00E-012
reg_CTRL	chr8	8738166	C	G	26	35,00%	het	9	-	MFHAS1		rs71163465	3,95E-009
reg_CTRL	chr8	8738261	C	T	12	100,00%	homvar	12	-	MFHAS1	rs11249893		1,00E-012
reg_CTRL	chr8	8740017	G	C	85	89,00%	homvar	76	-	MFHAS1	rs7820738		1,00E-012
reg_CTRL	chr8	8740237	A	G	65	97,00%	homvar	63	-	MFHAS1	rs907180		1,00E-012
reg_CTRL	chr8	8740285	T	C	63	97,00%	homvar	61	-	MFHAS1	rs907181		1,00E-012
reg_CTRL	chr8	8741091	C	T	13	100,00%	homvar	13	-	MFHAS1	rs6996376		1,00E-012
reg_CTRL	chr8	8741740	G	C	100	99,00%	homvar	99	-	MFHAS1	rs4481596		1,00E-012
reg_CTRL	chr8	8743619	A	C	81	98,00%	homvar	79	-	MFHAS1	rs11249896		1,00E-012
reg_CTRL	chr8	8743742	A	C	60	97,00%	homvar	58	-	MFHAS1	rs408459		1,00E-012
reg_CTRL	chr8	8744607	C	G	78	95,00%	homvar	74	-	MFHAS1	rs1877119		1,00E-012
reg_CTRL	chr8	8746124	A	C	88	100,00%	homvar	88	-	MFHAS1	rs440788		1,00E-012
reg_CTRL	chr8	8746384	C	G	101	95,00%	homvar	96	-	MFHAS1	rs3925830		1,00E-012
reg_CTRL	chr8	8747166	G	C	50	100,00%	homvar	50	-	MFHAS1	rs1964719		1,00E-012
reg_CTRL	chr8	8747381	C	T	48	98,00%	homvar	47	-	MFHAS1	rs3958877		1,00E-012
reg_CTRL	chr8	8748211	G	A	68	97,00%	homvar	66	-	MFHAS1	rs437895		1,00E-012
reg_CTRL	chr8	8750305	A	G	53	58,00%	het	31	-	MFHAS1	rs4348501		1,00E-012
reg_CTRL	chr8	8750413	A	G	45	64,00%	het	29	-	MFHAS1	rs13268671		1,00E-012
reg_CTRL	chr8	8750416	G	A	43	100,00%	homvar	43	-	MFHAS1	rs231188		1,00E-012
reg_CTRL	chr8	8750448	C	T	41	37,00%	het	15	-	MFHAS1	rs4523255		1,00E-012
reg_CTRL	chr8	8751363	T	C	72	56,00%	het	40	-	MFHAS1		rs56073940	1,00E-012
reg_CTRL	chr8	8752647	T	A	33	30,00%	het	10	-	MFHAS1			2,37E-009
reg_CTRL	chr8	8754381	C	T	59	34,00%	het	20	-	MFHAS1	rs5023278		1,00E-012
reg_CTRL	chr8	8756260	G	A	148	97,00%	homvar	144	-	MFHAS1	rs1039917		1,00E-012
reg_CTRL	chr8	8756923	G	A	57	95,00%	homvar	54	-	MFHAS1	rs35900578		1,00E-012
reg_CTRL	chr8	8758883	G	A	51	100,00%	homvar	51	-	MFHAS1	rs4382480		1,00E-012
reg_CTRL	chr8	8759109	G	A	46	50,00%	het	23	-	MFHAS1		rs71514515	1,00E-012
reg_CTRL	chr8	8759186	C	A	63	44,00%	het	28	-	MFHAS1			1,00E-012
reg_CTRL	chr8	8759937	G	A	35	49,00%	het	17	-	MFHAS1		rs56367294	1,00E-012
reg_CTRL	chr8	8760085	C	T	23	43,00%	het	10	-	MFHAS1	rs332037		3,67E-011
reg_CTRL	chr8	8761061	C	G	68	49,00%	het	33	-	MFHAS1	rs332039		1,00E-012
reg_CTRL	chr8	8761328	G	T	37	41,00%	het	15	-	MFHAS1	rs3789845		1,00E-012
reg_CTRL	chr8	8761667	C	T	74	51,00%	het	38	-	MFHAS1	rs3789843		1,00E-012
reg_CTRL	chr8	8761686	C	T	74	54,00%	het	40	-	MFHAS1	rs3827806		1,00E-012
reg_CTRL	chr8	8761825	C	T	67	43,00%	het	29	-	MFHAS1		rs60707155	1,00E-012

reg_CTRL	chr8	8762536	G	T	13	31,00%	het	4	-	MFHAS1	rs7017599		1,69E-004
reg_CTRL	chr8	8762639	G	A	18	33,00%	het	6	-	MFHAS1	rs1821007		2,17E-006
reg_CTRL	chr8	8762729	G	A	35	49,00%	het	17	-	MFHAS1	rs1821008		1,00E-012
reg_CTRL	chr8	8764214	G	T	132	55,00%	het	73	-	MFHAS1	rs1567398		1,00E-012
reg_CTRL	chr8	8766603	A	C	95	99,00%	homvar	94	-	MFHAS1	rs13274028		1,00E-012
reg_CTRL	chr8	8767171	G	C	115	99,00%	homvar	114	-	MFHAS1	rs907183		1,00E-012
reg_CTRL	chr8	8767898	G	A	68	100,00%	homvar	68	-	MFHAS1	rs332040		1,00E-012
reg_CTRL	chr8	8768326	G	C	65	97,00%	homvar	63	-	MFHAS1	rs4841058		1,00E-012
reg_CTRL	chr8	8768646	G	A	70	53,00%	het	37	-	MFHAS1		rs61591712	1,00E-012
reg_CTRL	chr8	8769293	C	T	65	95,00%	homvar	62	-	MFHAS1	rs9644694		1,00E-012
reg_CTRL	chr8	8770735	G	C	33	100,00%	homvar	33	-	MFHAS1	rs2009455		1,00E-012
reg_CTRL	chr8	8772507	A	C	86	100,00%	homvar	86	-	MFHAS1	rs10046783		1,00E-012
reg_CTRL	chr8	8772623	A	C	96	99,00%	homvar	95	-	MFHAS1	rs10046784		1,00E-012
reg_CTRL	chr8	8773796	A	G	59	100,00%	homvar	59	-	MFHAS1	rs12679021		1,00E-012
reg_CTRL	chr8	8774098	G	A	16	100,00%	homvar	16	-	MFHAS1	rs12681432		1,00E-012
reg_CTRL	chr8	8774113	A	T	17	76,00%	homvar	13	-	MFHAS1	rs7824578		1,00E-012
reg_CTRL	chr8	8774196	G	A	17	82,00%	homvar	14	-	MFHAS1	rs13261380		1,00E-012
reg_CTRL	chr8	8774295	G	T	35	57,00%	het	20	-	MFHAS1	rs34599909		1,00E-012
reg_CTRL	chr8	8774325	G	T	41	39,00%	het	16	-	MFHAS1		rs60965369	1,00E-012
reg_CTRL	chr8	8774535	T	C	99	49,00%	het	49	-	MFHAS1		rs73192206	1,00E-012
reg_CTRL	chr8	8774874	T	G	124	98,00%	homvar	122	-	MFHAS1	rs409997		1,00E-012
reg_CTRL	chr8	8775015	T	C	131	99,00%	homvar	130	-	MFHAS1	rs410487		1,00E-012
reg_CTRL	chr8	8775668	A	G	30	100,00%	homvar	30	-	MFHAS1	rs381800		1,00E-012
reg_CTRL	chr8	8776018	C	G	70	47,00%	het	33	-	MFHAS1		rs72626639	1,00E-012
reg_CTRL	chr8	8778570	A	G	23	13,00%	ambig	3	-	MFHAS1			1,53E-002
reg_CTRL	chr8	8778591	A	G	23	13,00%	ambig	3	-	MFHAS1			1,53E-002
reg_CTRL	chr8	8780758	G	T	38	100,00%	homvar	38	-	MFHAS1	rs435393		1,00E-012
reg_CTRL	chr8	8781650	G	A	33	42,00%	het	14	-	MFHAS1	rs7818276		1,00E-012
reg_CTRL	chr8	8784947	T	C	143	99,00%	homvar	142	-	MFHAS1	rs399123		1,00E-012
reg_CTRL	chr8	11738154	G	C	52	98,00%	homvar	51	-	CTSB	rs1736077		1,00E-012
reg_CTRL	chr8	11738505	A	C	67	37,00%	het	25	-	CTSB	rs8005		1,00E-012
reg_CTRL	chr8	11738607	G	A	60	95,00%	homvar	57	-	CTSB	rs12898		1,00E-012
reg_CTRL	chr8	11738662	A	G	46	54,00%	het	25	-	CTSB	rs2740592		1,00E-012
reg_CTRL	chr8	11738687	G	A	40	60,00%	het	24	-	CTSB	rs2645425		1,00E-012
reg_CTRL	chr8	11739251	A	C	105	100,00%	homvar	105	-	CTSB	rs1736078		1,00E-012
reg_CTRL	chr8	11739342	T	C	108	45,00%	het	49	-	CTSB	rs4839		1,00E-012
reg_CTRL	chr8	11739415	A	T	109	52,00%	het	57	-	CTSB	rs9009		1,00E-012
reg_CTRL	chr8	11739613	G	A	86	98,00%	homvar	84	-	CTSB	rs6730		1,00E-012
reg_CTRL	chr8	11739722	C	G	69	39,00%	het	27	-	CTSB	rs709822		1,00E-012
reg_CTRL	chr8	11739784	G	A	66	42,00%	het	28	-	CTSB	rs3947		1,00E-012
reg_CTRL	chr8	11740003	G	C	64	37,00%	het	24	-	CTSB	rs709821		1,00E-012
reg_CTRL	chr8	11740249	T	G	88	44,00%	het	39	-	CTSB	rs1736081		1,00E-012

reg_CTRL	chr8	11740457	C	G	69	41,00%	het	28			-	CTSB	rs1692811			1,00E-012
reg_CTRL	chr8	11740829	C	T	42	29,00%	het	12			-	CTSB	rs1736082			1,38E-010
reg_CTRL	chr8	11740906	G	T	48	40,00%	het	19			-	CTSB	rs1736083			1,00E-012
reg_CTRL	chr8	11740932	T	G	50	42,00%	het	21			-	CTSB	rs1692812			1,00E-012
reg_CTRL	chr8	11741061	A	G	58	43,00%	het	25			-	CTSB	rs6601616			1,00E-012
reg_CTRL	chr8	11741066	C	T	60	55,00%	het	33			-	CTSB	rs1736084			1,00E-012
reg_CTRL	chr8	11741068	T	C	59	54,00%	het	32			-	CTSB	rs1736085			1,00E-012
reg_CTRL	chr8	11741705	G	T	54	48,00%	het	26			-	CTSB	rs1692813			1,00E-012
reg_CTRL	chr8	11741707	T	C	54	48,00%	het	26			-	CTSB	rs1692814			1,00E-012
reg_CTRL	chr8	11741759	G	C	46	93,00%	homvar	43			-	CTSB	rs1692815			1,00E-012
reg_CTRL	chr8	11741866	A	C	35	97,00%	homvar	34			-	CTSB	rs1692816			1,00E-012
reg_CTRL	chr8	11742128	C	A	29	45,00%	het	13			-	CTSB	rs1692817			1,00E-012
reg_CTRL	chr8	11742287	C	G	34	94,00%	homvar	32			-	CTSB	rs1692818			1,00E-012
reg_CTRL	chr8	11742306	C	T	36	50,00%	het	18			-	CTSB	rs1736086			1,00E-012
reg_CTRL	chr8	11742548	C	T	22	59,00%	het	13			-	CTSB	rs2294138			1,00E-012
reg_CTRL	chr8	11742751	G	C	19	89,00%	homvar	17			-	CTSB	rs1736088			1,00E-012
reg_CTRL	chr8	11742851	G	T	26	46,00%	het	12			-	CTSB	rs2294139			1,00E-012
reg_CTRL	chr8	11742857	G	A	26	46,00%	het	12			-	CTSB	rs1692819			1,00E-012
reg_CTRL	chr8	11743086	C	G	69	61,00%	het	42			-	CTSB	rs2294140			1,00E-012
reg_CTRL	chr8	11743279	G	A	74	41,00%	het	30			-	CTSB	rs1736089			1,00E-012
reg_CTRL	chr8	11743638	T	C	63	92,00%	homvar	58			-	CTSB	rs1736090			1,00E-012
reg_CTRL	chr8	11743990	T	G	32	91,00%	homvar	29	T	T	-1	CTSB	rs13332			1,00E-012
reg_CTRL	chr8	11744243	T	C	35	49,00%	het	17			-	CTSB	rs35581201			1,00E-012
reg_CTRL	chr8	11744265	C	T	31	97,00%	homvar	30			-	CTSB	rs13280858			1,00E-012
reg_CTRL	chr8	11744315	T	C	27	96,00%	homvar	26			-	CTSB	rs13254438			1,00E-012
reg_CTRL	chr8	11744346	G	C	29	93,00%	homvar	27			-	CTSB	rs13278902			1,00E-012
reg_CTRL	chr8	11744382	T	C	32	100,00%	homvar	32			-	CTSB	rs4840586			1,00E-012
reg_CTRL	chr8	11744417	C	A	27	100,00%	homvar	27			-	CTSB	rs2645423			1,00E-012
reg_CTRL	chr8	11744436	A	G	24	96,00%	homvar	23			-	CTSB	rs2740593			1,00E-012
reg_CTRL	chr8	11744441	G	C	23	96,00%	homvar	22			-	CTSB	rs2645422			1,00E-012
reg_CTRL	chr8	11744583	A	G	7	71,00%	het	5			-	CTSB	rs2740594			1,30E-007
reg_CTRL	chr8	11744998	G	A	3	100,00%	homvar	3			-	CTSB	rs2645420			1,22E-005
reg_CTRL	chr8	11745209	T	C	6	100,00%	homvar	6			-	CTSB	rs2645419			1,48E-010
reg_CTRL	chr8	11745764	G	A	80	44,00%	het	35			-	CTSB	rs2272766			1,00E-012
reg_CTRL	chr8	11746124	A	T	34	44,00%	het	15			-	CTSB	rs28577034			1,00E-012
reg_CTRL	chr8	11746188	A	G	30	10,00%	ambig	3			-	CTSB		32,78		3,11E-002
reg_CTRL	chr8	11746209	T	G	29	48,00%	het	14			-	CTSB	rs2645417			1,00E-012
reg_CTRL	chr8	11746239	C	G	27	37,00%	het	10			-	CTSB	rs2740595			2,51E-010
reg_CTRL	chr8	11746416	G	A	53	40,00%	het	21			-	CTSB			rs62495697	1,00E-012
reg_CTRL	chr8	11746725	C	G	25	60,00%	het	15			-	CTSB	rs1961986			1,00E-012
reg_CTRL	chr8	11747366	T	C	30	40,00%	het	12			-	CTSB	rs1293290			2,51E-012
reg_CTRL	chr8	11747710	A	G	58	98,00%	homvar	57			-	CTSB	rs1293291			1,00E-012

reg_CTRL	chr8	11747778	G	A	67	45,00%	het	30			-	CTSB	rs1293292		1,00E-012
reg_CTRL	chr8	11748038	T	A	99	50,00%	het	50			-	CTSB	rs1122182		1,00E-012
reg_CTRL	chr8	11748297	G	C	80	51,00%	het	41	L	V	-1	CTSB	rs12338		1,00E-012
reg_CTRL	chr8	11748383	G	A	67	46,00%	het	31			-	CTSB	rs17154017		1,00E-012
reg_CTRL	chr8	11748468	C	T	63	43,00%	het	27			-	CTSB	rs2272767		1,00E-012
reg_CTRL	chr8	11748558	C	A	59	51,00%	het	30			-	CTSB	rs1293295		1,00E-012
reg_CTRL	chr8	11748566	A	C	60	48,00%	het	29			-	CTSB	rs1293296		1,00E-012
reg_CTRL	chr8	11748882	G	C	61	44,00%	het	27			-	CTSB	rs1293297		1,00E-012
reg_CTRL	chr8	11749852	A	C	43	42,00%	het	18			-	CTSB	rs1293298		1,00E-012
reg_CTRL	chr8	11750048	C	T	19	26,00%	het	5			-	CTSB	rs1736103		5,71E-005
reg_CTRL	chr8	11751261	A	G	40	50,00%	het	20			-	CTSB	rs17154027		1,00E-012
reg_CTRL	chr8	11751714	C	T	49	51,00%	het	25			-	CTSB	rs17814426		1,00E-012
reg_CTRL	chr8	11752509	A	T	50	50,00%	het	25			-	CTSB	rs9644756		1,00E-012
reg_CTRL	chr8	11752657	C	T	68	47,00%	het	32			-	CTSB			1,00E-012
reg_CTRL	chr8	11753882	G	C	106	43,00%	het	46			-	CTSB	rs6980952		1,00E-012
reg_CTRL	chr8	11755937	T	C	95	42,00%	het	40			-	CTSB	rs1293288		1,00E-012
reg_CTRL	chr8	11756896	T	C	42	62,00%	het	26			-	CTSB	rs2142470		1,00E-012
reg_CTRL	chr8	11757636	G	C	100	45,00%	het	45			-	CTSB	rs1293303		1,00E-012
reg_CTRL	chr8	11757954	A	G	68	40,00%	het	27			-	CTSB	rs1293304		1,00E-012
reg_CTRL	chr8	11758394	C	A	74	45,00%	het	33			-	CTSB	rs1293305		1,00E-012
reg_CTRL	chr8	11759455	A	G	39	51,00%	het	20			-	CTSB	rs2645415		1,00E-012
reg_CTRL	chr8	11759534	C	T	40	50,00%	het	20			-	CTSB	rs1299525		1,00E-012
reg_CTRL	chr8	11759764	G	A	66	41,00%	het	27			-	CTSB	rs1296022		1,00E-012
reg_CTRL	chr8	11760540	A	G	36	97,00%	homvar	35			-	CTSB	rs1293307		1,00E-012
reg_CTRL	chr8	11761184	G	A	43	51,00%	het	22			-	CTSB	rs1293309		1,00E-012
reg_CTRL	chr8	12624420	G	A	92	100,00%	homvar	92			-	LONRF1	rs7005881		1,00E-012
reg_CTRL	chr8	12625320	T	C	121	51,00%	het	62			-	LONRF1		rs73202627	1,00E-012
reg_CTRL	chr8	12626051	T	C	90	100,00%	homvar	90			-	LONRF1	rs4831767		1,00E-012
reg_CTRL	chr8	12626226	C	T	95	47,00%	het	45			-	LONRF1	rs4831768		1,00E-012
reg_CTRL	chr8	12626235	A	G	94	99,00%	homvar	93			-	LONRF1	rs4831769		1,00E-012
reg_CTRL	chr8	12627074	T	A	102	99,00%	homvar	101			-	LONRF1	rs10429335		1,00E-012
reg_CTRL	chr8	12627915	C	T	104	98,00%	homvar	102			-	LONRF1	rs11782145		1,00E-012
reg_CTRL	chr8	12628025	T	C	113	88,00%	homvar	99			-	LONRF1	rs10100866		1,00E-012
reg_CTRL	chr8	12628463	T	C	96	97,00%	homvar	93			-	LONRF1		rs72603953	1,00E-012
reg_CTRL	chr8	12628721	A	G	51	98,00%	homvar	50			-	LONRF1	rs9632851		1,00E-012
reg_CTRL	chr8	12629030	C	T	14	93,00%	homvar	13			-	LONRF1	rs10095845		1,00E-012
reg_CTRL	chr8	12629393	C	A	36	53,00%	het	19			-	LONRF1		rs73202633	1,00E-012
reg_CTRL	chr8	12629532	A	T	60	97,00%	homvar	58			-	LONRF1	rs6530953		1,00E-012
reg_CTRL	chr8	12629807	C	G	102	99,00%	homvar	101			-	LONRF1	rs7010337		1,00E-012
reg_CTRL	chr8	12630635	A	C	116	49,00%	het	57			-	LONRF1		rs73202635	1,00E-012
reg_CTRL	chr8	12631165	G	C	73	56,00%	het	41	T	S	-3	LONRF1			1,00E-012
reg_CTRL	chr8	12631166	T	A	72	58,00%	het	42	T	S	-3	LONRF1			1,00E-012

reg_CTRL	chr8	12631700	T	C	41	56,00%	het	23	-	LONRF1	rs17761564		1,00E-012
reg_CTRL	chr8	12631939	T	C	56	50,00%	het	28	-	LONRF1		rs56114121	1,00E-012
reg_CTRL	chr8	12632233	T	A	75	41,00%	het	31	-	LONRF1	rs17761606		1,00E-012
reg_CTRL	chr8	12632654	A	C	46	98,00%	homvar	45	-	LONRF1	rs6995647		1,00E-012
reg_CTRL	chr8	12633550	C	A	80	62,00%	het	50	-	LONRF1	rs3802269		1,00E-012
reg_CTRL	chr8	12634490	G	C	77	56,00%	het	43	-	LONRF1	rs4272378		1,00E-012
reg_CTRL	chr8	12634940	G	A	39	46,00%	het	18	-	LONRF1	rs7463601		1,00E-012
reg_CTRL	chr8	12634992	A	G	29	100,00%	homvar	29	-	LONRF1	rs7461006		1,00E-012
reg_CTRL	chr8	12635185	A	G	46	100,00%	homvar	46	-	LONRF1	rs6530956		1,00E-012
reg_CTRL	chr8	12635399	G	A	45	93,00%	homvar	42	-	LONRF1	rs6530958		1,00E-012
reg_CTRL	chr8	12635958	A	C	28	100,00%	homvar	28	-	LONRF1	rs4258004		1,00E-012
reg_CTRL	chr8	12636319	C	T	81	99,00%	homvar	80	-	LONRF1	rs6530959		1,00E-012
reg_CTRL	chr8	12637327	C	A	86	97,00%	homvar	83	-	LONRF1	rs13251315		1,00E-012
reg_CTRL	chr8	12637416	A	G	82	93,00%	homvar	76	-	LONRF1	rs13272425		1,00E-012
reg_CTRL	chr8	12637740	T	C	39	44,00%	het	17	-	LONRF1	rs11784110		1,00E-012
reg_CTRL	chr8	12637991	T	C	41	100,00%	homvar	41	-	LONRF1	rs9325786		1,00E-012
reg_CTRL	chr8	12640631	C	T	69	99,00%	homvar	68	-	LONRF1	rs7014187		1,00E-012
reg_CTRL	chr8	12641320	A	G	48	40,00%	het	19	-	LONRF1	rs4831777		1,00E-012
reg_CTRL	chr8	12641415	C	G	46	57,00%	het	26	-	LONRF1		rs73202639	1,00E-012
reg_CTRL	chr8	12642348	C	A	76	71,00%	het	54	-	LONRF1	rs6530962		1,00E-012
reg_CTRL	chr8	12642503	A	C	74	96,00%	homvar	71	-	LONRF1	rs4625037		1,00E-012
reg_CTRL	chr8	12642559	T	C	67	48,00%	het	32	-	LONRF1	rs11775169		1,00E-012
reg_CTRL	chr8	12642979	C	T	53	98,00%	homvar	52	-	LONRF1	rs4831354		1,00E-012
reg_CTRL	chr8	12643126	C	T	57	65,00%	het	37	-	LONRF1	rs7819033		1,00E-012
reg_CTRL	chr8	12643453	A	C	47	100,00%	homvar	47	-	LONRF1	rs4831780		1,00E-012
reg_CTRL	chr8	12643642	A	C	41	93,00%	homvar	38	-	LONRF1	rs10098734		1,00E-012
reg_CTRL	chr8	12643654	G	A	39	100,00%	homvar	39	-	LONRF1	rs10110145		1,00E-012
reg_CTRL	chr8	12643838	T	C	47	100,00%	homvar	47	-	LONRF1	rs7014429		1,00E-012
reg_CTRL	chr8	12644993	C	T	50	98,00%	homvar	49	-	LONRF1	rs7837242		1,00E-012
reg_CTRL	chr8	12645405	C	T	59	53,00%	het	31	-	LONRF1	rs6530964		1,00E-012
reg_CTRL	chr8	12645526	A	C	57	100,00%	homvar	57	-	LONRF1	rs6530965		1,00E-012
reg_CTRL	chr8	12646136	C	G	36	100,00%	homvar	36	-	LONRF1	rs7842201		1,00E-012
reg_CTRL	chr8	12646342	T	C	56	98,00%	homvar	55	-	LONRF1	rs7819248		1,00E-012
reg_CTRL	chr8	12646912	G	C	63	98,00%	homvar	62	-	LONRF1	rs4831784		1,00E-012
reg_CTRL	chr8	12647560	C	G	95	48,00%	het	46	-	LONRF1	rs6985289		1,00E-012
reg_CTRL	chr8	12647982	A	T	107	93,00%	homvar	100	-	LONRF1	rs6530966		1,00E-012
reg_CTRL	chr8	12648329	G	C	100	100,00%	homvar	100	-	LONRF1	rs7838660		1,00E-012
reg_CTRL	chr8	12648422	T	C	101	100,00%	homvar	101	-	LONRF1	rs7832448		1,00E-012
reg_CTRL	chr8	12648780	C	G	83	40,00%	het	33	-	LONRF1	rs9325792		1,00E-012
reg_CTRL	chr8	12648809	T	C	78	58,00%	het	45	-	LONRF1		rs73202647	1,00E-012
reg_CTRL	chr8	12648863	A	G	75	43,00%	het	32	-	LONRF1	rs9325793		1,00E-012
reg_CTRL	chr8	12649062	G	T	59	100,00%	homvar	59	-	LONRF1	rs7014516		1,00E-012

reg_CTRL	chr8	12649595	G	A	25	100,00%	homvar	25	-	LONRF1	rs10441667		1,00E-012
reg_CTRL	chr8	12650052	T	C	20	65,00%	het	13	-	LONRF1	rs17767600		1,00E-012
reg_CTRL	chr8	12650145	C	T	18	22,00%	ambig	4	-	LONRF1	rs7462166		6,61E-004
reg_CTRL	chr8	12651062	T	C	73	53,00%	het	39	-	LONRF1	rs4436128		1,00E-012
reg_CTRL	chr8	12651131	T	C	68	51,00%	het	35	-	LONRF1	rs4437649		1,00E-012
reg_CTRL	chr8	12651557	C	G	36	39,00%	het	14	-	LONRF1	rs10503427		1,00E-012
reg_CTRL	chr8	12651680	C	T	29	100,00%	homvar	29	-	LONRF1	rs7007056		1,00E-012
reg_CTRL	chr8	12652000	C	T	51	98,00%	homvar	50	-	LONRF1	rs7007550		1,00E-012
reg_CTRL	chr8	12652284	C	G	50	100,00%	homvar	50	-	LONRF1	rs6530968		1,00E-012
reg_CTRL	chr8	12652948	G	A	74	50,00%	het	37	-	LONRF1		rs73202653	1,00E-012
reg_CTRL	chr8	12653361	C	T	9	44,00%	het	4	-	LONRF1	rs6988764		3,21E-005
reg_CTRL	chr8	12653488	C	G	14	100,00%	homvar	14	-	LONRF1	rs4831795		1,00E-012
reg_CTRL	chr8	12654972	A	G	139	100,00%	homvar	139	-	LONRF1	rs6530969		1,00E-012
reg_CTRL	chr8	12655147	T	C	107	50,00%	het	54	-	LONRF1	rs6530970		1,00E-012
