# Supplementary material for: Comprehensive assessment of sequence variation within the copy number variable defensin cluster on 8p23 by target enriched in-depth 454 sequencing
Source: BMC Genomics. 2011 May 18;12:243. doi: 10.1186/1471-2164-12-243 (PMC3118217; doi:10.1186/1471-2164-12-243)
Supplement: Additional file 11 — HCDiffs identified from NA12760. HCDiffs identified from NA12760 (CTRL, DEFA, DEFB, after exclusion of indels and complex nucleotide exchanges) [file 1471-2164-12-243-S11.PDF]

additional file 11: HCDiffs identified from NA12760

dep_tot= sequence depth total / dep_var = sequence depth of variant / VAF = variant's allele frequency / aa = amino acid

region	segdup	hg18	pos	ref	var	dep_t	VAF	var_type	dep_var	aa_ref	aa_var	strand	gene	SNPhg18	SNPhg18	SNPblat	P
reg_CTRL		chr17	31286367	A	G	45	49,00%	het	22			-	LYZL6	rs17676662			1,00E-012
reg_CTRL		chr17	31288659	T	C	58	53,00%	het	31			-	LYZL6	rs2280784			1,00E-012
reg_CTRL		chr17	31288972	A	G	26	58,00%	het	15	D	D	-2	LYZL6	rs2280783			1,00E-012
reg_CTRL		chr17	31289472	C	T	15	27,00%	het	4			-	LYZL6	rs11654215			3,12E-004
reg_CTRL		chr17	31289599	G	A	10	50,00%	het	5			-	LYZL6				1,47E-006
reg_CTRL		chr17	31290141	T	C	45	49,00%	het	22			-	LYZL6	rs9901969			1,00E-012
reg_CTRL		chr19	59866310	T	C	9	33,00%	het	3	F	L	1	LILRB4	rs28366008			9,21E-004
reg_CTRL		chr19	59867552	C	T	9	44,00%	het	4	F	F	2	LILRB4	rs3745871			3,21E-005
reg_CTRL		chr19	59870501	T	G	7	43,00%	het	3			+	LILRB4				3,97E-004
reg_CTRL		chr19	59870545	A	G	7	43,00%	het	3			+	LILRB4	rs11574582			3,97E-004
reg_CTRL		chr19	59870587	G	A	11	36,00%	het	4			+	LILRB4				8,11E-005
reg_CTRL		chr19	59870609	A	G	10	30,00%	het	3			+	LILRB4				1,29E-003
reg_CTRL		chr19	59870677	A	G	11	27,00%	het	3			+	LILRB4				1,75E-003
reg_CTRL		chr19	59870732	G	A	10	30,00%	het	3			+	LILRB4	rs2569715			1,29E-003
reg_CTRL		chr19	59870794	G	A	9	78,00%	homvar	7			+	LILRB4	rs11574587			1,44E-010
reg_CTRL		chr19	59870823	T	C	9	33,00%	het	3			+	LILRB4			rs71365476	9,21E-004
reg_CTRL		chr19	59870834	G	A	10	50,00%	het	5			+	LILRB4				1,47E-006
reg_CTRL		chr19	59870860	A	G	10	40,00%	het	4			+	LILRB4				5,26E-005
reg_CTRL		chr19	59870864	T	C	10	50,00%	het	5			+	LILRB4				1,47E-006
reg_CTRL		chr19	60078214	G	A	16	19,00%	ambig	3			+	FCAR				5,44E-003
reg_CTRL		chr19	60079765	G	A	11	36,00%	het	4			+	FCAR	rs10407012			8,11E-005
reg_CTRL		chr19	60081277	A	G	17	53,00%	het	9			+	FCAR	rs4806604			5,40E-011
reg_CTRL		chr19	60082413	A	G	10	40,00%	het	4			+	FCAR	rs7257926			5,26E-005
reg_CTRL		chr19	60082645	G	A	10	40,00%	het	4			+	FCAR	rs10402725			5,26E-005
reg_CTRL		chr19	60084881	T	C	7	86,00%	homvar	6			+	FCAR	rs7248382	rs7247547		1,02E-009
reg_CTRL		chr19	60085431	A	G	13	100,00%	homvar	13			+	FCAR	rs4239591			1,00E-012
reg_CTRL		chr19	60085626	A	G	17	53,00%	het	9			+	FCAR	rs4806606			5,40E-011
reg_CTRL		chr19	60087161	C	G	9	56,00%	het	5			+	FCAR	rs28756208			7,51E-007
reg_CTRL		chr19	60088124	G	A	6	100,00%	homvar	6			+	FCAR	rs10401687			1,48E-010
reg_CTRL		chr19	60088425	G	A	20	100,00%	homvar	20			+	FCAR	rs10402324			1,00E-012
reg_CTRL		chr19	60088712	A	G	48	98,00%	homvar	47	R	R	2	FCAR	rs1865096			1,00E-012
reg_CTRL		chr19	60089029	A	G	32	100,00%	homvar	32			+	FCAR	rs1865097			1,00E-012
reg_CTRL		chr19	60089192	C	T	21	95,00%	homvar	20			+	FCAR	rs11666846			1,00E-012
reg_CTRL		chr19	60089677	T	C	7	100,00%	homvar	7			+	FCAR	rs12974020			3,40E-012
reg_CTRL		chr19	60089829	T	C	14	93,00%	homvar	13			+	FCAR	rs12974530			1,00E-012
reg_CTRL		chr19	60089913	A	G	18	100,00%	homvar	18			+	FCAR	rs12972637			1,00E-012
reg_CTRL		chr19	60090047	T	C	13	100,00%	homvar	13			+	FCAR	rs12975083			1,00E-012
reg_CTRL		chr19	60091123	G	T	10	40,00%	het	4			+	FCAR	rs7258679			5,26E-005
reg_CTRL		chr19	60092542	C	A	5	80,00%	homvar	4			+	FCAR	rs12976517			1,37E-006
reg_CTRL		chr19	60092982	A	G	37	46,00%	het	17	S	G	2	FCAR	rs16986050			1,00E-012
reg_CTRL		chr19	60093536	C	T	13	46,00%	het	6			+	FCAR	rs10413148			2,21E-007
reg_CTRL		chr19	60093544	T	G	13	46,00%	het	6			+	FCAR	rs10414707			2,21E-007
reg_CTRL		chr20	29530351	C	T	17	18,00%	ambig	3			+	REM1				6,50E-003
reg_CTRL		chr20	29530984	A	G	51	100,00%	homvar	51			+	REM1	rs215911			1,00E-012
reg_CTRL		chr20	29531283	T	G	20	20,00%	ambig	4			+	REM1				1,01E-003
reg_CTRL		chr20	29531301	C	T	19	16,00%	ambig	3			+	REM1				8,95E-003
reg_CTRL		chr20	29531308	T	C	19	21,00%	ambig	4			+	REM1				8,22E-004
reg_CTRL		chr20	29531364	A	G	18	17,00%	ambig	3			+	REM1				7,66E-003
reg_CTRL		chr20	29531374	A	T	17	18,00%	ambig	3			+	REM1				6,50E-003

reg_CTRL	chr20	29531866	T	C	24	21,00%	ambig	5	+	REM1	rs13037125		1,90E-004
reg_CTRL	chr20	29531869	C	T	24	12,00%	ambig	3	+	REM1			1,72E-002
reg_CTRL	chr20	29566293	C	T	11	73,00%	het	8	+	HM13			1,56E-011
reg_CTRL	chr20	29567135	C	G	39	44,00%	het	17	+	HM13	rs6088440		1,00E-012
reg_CTRL	chr20	29567312	C	T	40	42,00%	het	17	+	HM13	rs6088441		1,00E-012
reg_CTRL	chr20	29568468	A	G	41	44,00%	het	18	+	HM13	rs6059740		1,00E-012
reg_CTRL	chr20	29570332	G	T	16	94,00%	homvar	15	+	HM13		rs57705377	1,00E-012
reg_CTRL	chr20	29571828	T	C	27	11,00%	ambig	3	+	HM13			2,36E-002
reg_CTRL	chr20	29571836	T	C	27	11,00%	ambig	3	+	HM13			2,36E-002
reg_CTRL	chr20	29571869	A	G	26	15,00%	ambig	4	+	HM13			2,79E-003
reg_CTRL	chr20	29571903	A	G	27	15,00%	ambig	4	+	HM13			3,22E-003
reg_CTRL	chr20	29571922	T	C	29	17,00%	ambig	5	+	HM13		rs56917022	4,82E-004
reg_CTRL	chr20	29571924	C	T	30	13,00%	ambig	4	+	HM13			4,76E-003
reg_CTRL	chr20	29571940	A	G	32	16,00%	ambig	5	+	HM13		rs12245561	7,72E-004
reg_CTRL	chr20	29571953	T	A	32	16,00%	ambig	5	+	HM13			7,72E-004
reg_CTRL	chr20	29572029	T	A	39	13,00%	ambig	5	+	HM13			1,93E-003
reg_CTRL	chr20	29573801	C	T	29	10,00%	ambig	3	+	HM13			2,85E-002
reg_CTRL	chr20	29573808	T	C	25	12,00%	ambig	3	+	HM13			1,92E-002
reg_CTRL	chr20	29573824	T	C	25	12,00%	ambig	3	+	HM13			1,92E-002
reg_CTRL	chr20	29573856	A	C	19	63,00%	het	12	+	HM13			1,00E-012
reg_CTRL	chr20	29580150	T	C	14	21,00%	ambig	3	+	HM13			3,66E-003
reg_CTRL	chr20	29582163	G	A	32	59,00%	het	19	+	HM13	rs6088481		1,00E-012
reg_CTRL	chr20	29591769	T	G	36	100,00%	homvar	36	+	HM13	rs1555285		1,00E-012
reg_CTRL	chr20	29596516	T	A	33	48,00%	het	16	+	HM13			1,00E-012
reg_CTRL	chr20	29609235	A	G	14	21,00%	ambig	3	+	HM13			3,66E-003
reg_CTRL	chr20	29609258	A	G	18	17,00%	ambig	3	+	HM13			7,66E-003
reg_CTRL	chr20	29614169	C	G	34	44,00%	het	15	+	HM13			1,00E-012
reg_CTRL	chr8	6348637	A	C	41	51,00%	het	21	+	MCPH1	rs2979666		1,00E-012
reg_CTRL	chr8	6349014	G	A	10	60,00%	het	6	+	MCPH1			2,87E-008
reg_CTRL	chr8	6349033	A	G	10	50,00%	het	5	+	MCPH1	rs2515598		1,47E-006
reg_CTRL	chr8	6349115	C	T	13	23,00%	ambig	3	+	MCPH1			2,93E-003
reg_CTRL	chr8	6349127	G	T	14	43,00%	het	6	+	MCPH1	rs11779671		3,79E-007
reg_CTRL	chr8	6349196	G	A	18	17,00%	ambig	3	+	MCPH1			7,66E-003
reg_CTRL	chr8	6351021	T	A	18	61,00%	het	11	+	MCPH1	rs13249897		1,00E-012
reg_CTRL	chr8	6351031	G	C	22	36,00%	het	8	+	MCPH1	rs17077194		1,88E-008
reg_CTRL	chr8	6351326	A	G	47	53,00%	het	25	+	MCPH1		rs73199028	1,00E-012
reg_CTRL	chr8	6351358	C	G	47	57,00%	het	27	+	MCPH1	rs2442468		1,00E-012
reg_CTRL	chr8	6351983	C	T	30	60,00%	het	18	+	MCPH1	rs2442467		1,00E-012
reg_CTRL	chr8	6352330	T	C	27	44,00%	het	12	+	MCPH1	rs2515409		1,00E-012
reg_CTRL	chr8	6352755	G	A	16	50,00%	het	8	+	MCPH1	rs2515411		8,62E-010
reg_CTRL	chr8	6352770	C	T	16	50,00%	het	8	+	MCPH1	rs2515412		8,62E-010
reg_CTRL	chr8	6353028	T	C	29	41,00%	het	12	+	MCPH1	rs2515413		1,00E-012
reg_CTRL	chr8	6353146	C	A	23	48,00%	het	11	+	MCPH1	rs2515414		1,66E-012
reg_CTRL	chr8	6353242	C	G	23	43,00%	het	10	+	MCPH1	rs2442466		3,67E-011
reg_CTRL	chr8	6353746	G	A	64	56,00%	het	36	+	MCPH1	rs2515416		1,00E-012
reg_CTRL	chr8	6355043	C	T	37	68,00%	het	25	+	MCPH1	rs6559165		1,00E-012
reg_CTRL	chr8	6355246	C	G	38	53,00%	het	20	+	MCPH1	rs6559166		1,00E-012
reg_CTRL	chr8	6357304	G	A	54	56,00%	het	30	+	MCPH1	rs3780088		1,00E-012
reg_CTRL	chr8	6357957	C	G	89	45,00%	het	40	+	MCPH1	rs7013006		1,00E-012
reg_CTRL	chr8	6359192	G	C	22	18,00%	ambig	4	+	MCPH1			1,47E-003
reg_CTRL	chr8	6359199	C	A	22	14,00%	ambig	3	+	MCPH1			1,35E-002
reg_CTRL	chr8	6360058	T	A	31	45,00%	het	14	+	MCPH1	rs7838691		1,00E-012
reg_CTRL	chr8	6361113	T	G	66	58,00%	het	38	+	MCPH1	rs4276711		1,00E-012

reg_CTRL	chr8	6362181	T	C	57	40,00%	het	23			+	MCPH1	rs2515432		1,00E-012
reg_CTRL	chr8	6362697	G	A	43	51,00%	het	22			+	MCPH1		rs73199039	1,00E-012
reg_CTRL	chr8	6366192	C	A	57	47,00%	het	27	T	T	-3	ANGPT2		rs55633437	1,00E-012
reg_CTRL	chr8	6366992	A	T	29	38,00%	het	11			+	MCPH1		rs56405992	4,26E-011
reg_CTRL	chr8	6373087	T	A	26	42,00%	het	11			+	MCPH1	rs2442610		9,51E-012
reg_CTRL	chr8	6373270	G	C	41	59,00%	het	24			+	MCPH1	rs2515465		1,00E-012
reg_CTRL	chr8	6373518	C	T	60	53,00%	het	32			+	MCPH1			1,00E-012
reg_CTRL	chr8	6373535	G	C	61	41,00%	het	25			+	MCPH1	rs2922887		1,00E-012
reg_CTRL	chr8	6373658	T	C	57	46,00%	het	26			+	MCPH1	rs3824310		1,00E-012
reg_CTRL	chr8	6373694	A	G	56	43,00%	het	24			+	MCPH1	rs2515466		1,00E-012
reg_CTRL	chr8	6373849	G	A	59	58,00%	het	34			+	MCPH1	rs3824312		1,00E-012
reg_CTRL	chr8	6373992	G	C	76	49,00%	het	37			+	MCPH1	rs2922886		1,00E-012
reg_CTRL	chr8	6374001	T	C	79	48,00%	het	38			+	MCPH1	rs2442609		1,00E-012
reg_CTRL	chr8	6374007	T	G	77	35,00%	het	27			+	MCPH1	rs2515469		1,00E-012
reg_CTRL	chr8	6374028	T	C	77	44,00%	het	34			+	MCPH1	rs2442608		1,00E-012
reg_CTRL	chr8	6374155	T	A	74	96,00%	homvar	71			+	MCPH1	rs1868554		1,00E-012
reg_CTRL	chr8	6374404	A	G	29	10,00%	ambig	3			+	MCPH1			2,85E-002
reg_CTRL	chr8	6374450	T	C	20	55,00%	het	11			+	MCPH1	rs13268979		1,00E-012
reg_CTRL	chr8	6374526	T	C	15	100,00%	homvar	15			+	MCPH1	rs1868552		1,00E-012
reg_CTRL	chr8	6374645	C	T	20	50,00%	het	10			+	MCPH1	rs35735391		9,66E-012
reg_CTRL	chr8	6374772	G	A	36	44,00%	het	16			+	MCPH1			1,00E-012
reg_CTRL	chr8	6374964	T	C	37	41,00%	het	15			+	MCPH1	rs1807209		1,00E-012
reg_CTRL	chr8	6375253	G	A	15	100,00%	homvar	15			+	MCPH1	rs2256628		1,00E-012
reg_CTRL	chr8	6375399	C	T	6	100,00%	homvar	6			+	MCPH1	rs2515473		1,48E-010
reg_CTRL	chr8	6375563	A	G	21	95,00%	homvar	20			+	MCPH1	rs734703		1,00E-012
reg_CTRL	chr8	6375592	G	A	25	100,00%	homvar	25			+	MCPH1	rs746073		1,00E-012
reg_CTRL	chr8	6375621	T	C	28	100,00%	homvar	28			+	MCPH1	rs734702		1,00E-012
reg_CTRL	chr8	6375655	G	A	34	100,00%	homvar	34			+	MCPH1	rs734701		1,00E-012
reg_CTRL	chr8	6375714	A	G	39	100,00%	homvar	39			+	MCPH1	rs734704		1,00E-012
reg_CTRL	chr8	6375941	T	C	39	44,00%	het	17			+	MCPH1	rs2442604		1,00E-012
reg_CTRL	chr8	6376190	G	C	26	100,00%	homvar	26			+	MCPH1	rs10503371		1,00E-012
reg_CTRL	chr8	6376528	G	T	42	55,00%	het	23			+	MCPH1	rs2515479		1,00E-012
reg_CTRL	chr8	6376624	T	G	45	100,00%	homvar	45			+	MCPH1	rs12674822		1,00E-012
reg_CTRL	chr8	6376688	G	A	46	48,00%	het	22			+	MCPH1	rs2515480		1,00E-012
reg_CTRL	chr8	6376791	A	T	53	47,00%	het	25			+	MCPH1	rs1984860		1,00E-012
reg_CTRL	chr8	6376832	T	C	43	44,00%	het	19			+	MCPH1	rs1984859		1,00E-012
reg_CTRL	chr8	6377122	T	C	52	31,00%	het	16			+	MCPH1	rs1984857		1,00E-012
reg_CTRL	chr8	6377297	C	A	44	57,00%	het	25	A	A	-1	ANGPT2	rs6559167		1,00E-012
reg_CTRL	chr8	6377435	A	G	38	47,00%	het	18			+	MCPH1	rs2515481		1,00E-012
reg_CTRL	chr8	6377487	G	A	37	100,00%	homvar	37			+	MCPH1	rs2515482		1,00E-012
reg_CTRL	chr8	6377745	G	T	57	100,00%	homvar	57			+	MCPH1	rs1031303		1,00E-012
reg_CTRL	chr8	6377938	A	G	61	57,00%	het	35			+	MCPH1	rs17077419		1,00E-012
reg_CTRL	chr8	6378159	T	C	59	98,00%	homvar	58			+	MCPH1	rs2442602		1,00E-012
reg_CTRL	chr8	6378710	A	G	32	100,00%	homvar	32			+	MCPH1	rs2959812		1,00E-012
reg_CTRL	chr8	6378974	C	A	18	39,00%	het	7			+	MCPH1	rs2959811		8,67E-008
reg_CTRL	chr8	6379175	T	C	20	100,00%	homvar	20			+	MCPH1	rs2922881		1,00E-012
reg_CTRL	chr8	6379358	C	G	26	62,00%	het	16			+	MCPH1	rs12550255		1,00E-012
reg_CTRL	chr8	6379594	A	G	27	37,00%	het	10			+	MCPH1	rs12676103		2,51E-010
reg_CTRL	chr8	6379677	G	C	37	51,00%	het	19			+	MCPH1	rs2515483		1,00E-012
reg_CTRL	chr8	6379706	T	A	38	53,00%	het	20			+	MCPH1	rs17623064		1,00E-012
reg_CTRL	chr8	6379955	C	T	62	45,00%	het	28			+	MCPH1	rs4841224		1,00E-012
reg_CTRL	chr8	6381188	G	T	37	43,00%	het	16			+	MCPH1	rs2959809		1,00E-012
reg_CTRL	chr8	6381373	G	A	60	50,00%	het	30			+	MCPH1	rs2442600		1,00E-012

reg_CTRL	chr8	6381559	G	A	71	100,00%	homvar	71	+	MCPH1	rs2442599		1,00E-012
reg_CTRL	chr8	6381587	A	G	68	49,00%	het	33	+	MCPH1	rs17552444		1,00E-012
reg_CTRL	chr8	6382229	T	C	35	100,00%	homvar	35	+	MCPH1	rs2442598		1,00E-012
reg_CTRL	chr8	6382284	T	G	30	60,00%	het	18	+	MCPH1	rs12541780		1,00E-012
reg_CTRL	chr8	6382769	T	C	7	57,00%	het	4	+	MCPH1	rs17077452		9,26E-006
reg_CTRL	chr8	6382971	T	A	12	92,00%	homvar	11	+	MCPH1	rs2959808		1,00E-012
reg_CTRL	chr8	6383165	C	T	26	38,00%	het	10	+	MCPH1	rs12549309		1,61E-010
reg_CTRL	chr8	6383317	G	A	39	54,00%	het	21	+	MCPH1	rs11989215		1,00E-012
reg_CTRL	chr8	6383428	G	A	47	51,00%	het	24	+	MCPH1	rs11989242		1,00E-012
reg_CTRL	chr8	6383590	A	C	47	49,00%	het	23	+	MCPH1	rs11137037		1,00E-012
reg_CTRL	chr8	6383649	A	G	49	45,00%	het	22	+	MCPH1	rs10092206		1,00E-012
reg_CTRL	chr8	6383749	C	T	43	56,00%	het	24	+	MCPH1	rs17623313		1,00E-012
reg_CTRL	chr8	6384104	C	T	47	60,00%	het	28	+	MCPH1	rs2922876		1,00E-012
reg_CTRL	chr8	6384278	G	A	44	43,00%	het	19	+	MCPH1	rs1375668		1,00E-012
reg_CTRL	chr8	6384313	G	A	40	45,00%	het	18	+	MCPH1	rs1989321		1,00E-012
reg_CTRL	chr8	6384394	G	T	31	39,00%	het	12	+	MCPH1	rs2897911		4,09E-012
reg_CTRL	chr8	6384406	G	C	31	42,00%	het	13	+	MCPH1	rs1823375		1,00E-012
reg_CTRL	chr8	6384513	C	A	21	95,00%	homvar	20	+	MCPH1	rs1823376		1,00E-012
reg_CTRL	chr8	6384555	T	G	20	35,00%	het	7	+	MCPH1	rs2408341		2,03E-007
reg_CTRL	chr8	6384627	C	T	17	41,00%	het	7	+	MCPH1	rs4263789		5,41E-008
reg_CTRL	chr8	6384716	A	T	17	35,00%	het	6	+	MCPH1	rs4376511		1,47E-006
reg_CTRL	chr8	6384838	A	T	33	48,00%	het	16	+	MCPH1	rs4455855		1,00E-012
reg_CTRL	chr8	6384861	C	T	38	47,00%	het	18	+	MCPH1	rs4991608		1,00E-012
reg_CTRL	chr8	6385378	C	T	65	52,00%	het	34	+	MCPH1	rs2922875		1,00E-012
reg_CTRL	chr8	6385578	A	G	39	59,00%	het	23	+	MCPH1	rs2922874		1,00E-012
reg_CTRL	chr8	6385735	A	G	28	100,00%	homvar	28	+	MCPH1	rs2922873		1,00E-012
reg_CTRL	chr8	6385772	G	A	28	57,00%	het	16	+	MCPH1	rs2922872		1,00E-012
reg_CTRL	chr8	6385878	A	T	22	55,00%	het	12	+	MCPH1			1,00E-012
reg_CTRL	chr8	6385973	T	C	13	100,00%	homvar	13	+	MCPH1	rs2922871		1,00E-012
reg_CTRL	chr8	6386005	G	A	14	57,00%	het	8	+	MCPH1	rs2959819		2,09E-010
reg_CTRL	chr8	6386346	G	T	32	100,00%	homvar	32	+	MCPH1	rs2959820		1,00E-012
reg_CTRL	chr8	6387008	A	G	32	50,00%	het	16	+	MCPH1	rs17077465		1,00E-012
reg_CTRL	chr8	6387201	T	C	29	45,00%	het	13	+	MCPH1	rs2922869		1,00E-012
reg_CTRL	chr8	6387274	T	G	25	96,00%	homvar	24	+	MCPH1	rs35742902		1,00E-012
reg_CTRL	chr8	6387413	G	A	25	36,00%	het	9	+	MCPH1	rs1988762		2,64E-009
reg_CTRL	chr8	6388961	T	C	65	58,00%	het	38	+	MCPH1			1,00E-012
reg_CTRL	chr8	6389049	C	T	65	97,00%	homvar	63	+	MCPH1	rs2442597		1,00E-012
reg_CTRL	chr8	6389774	T	C	78	58,00%	het	45	+	MCPH1	rs4478599	rs13250248	1,00E-012
reg_CTRL	chr8	6390079	A	G	68	53,00%	het	36	+	MCPH1			1,00E-012
reg_CTRL	chr8	6390296	A	C	58	47,00%	het	27	+	MCPH1	rs7005658		1,00E-012
reg_CTRL	chr8	6390400	A	G	68	99,00%	homvar	67	+	MCPH1	rs2442596		1,00E-012
reg_CTRL	chr8	6390414	A	C	70	100,00%	homvar	70	+	MCPH1	rs2515488		1,00E-012
reg_CTRL	chr8	6391248	T	C	55	100,00%	homvar	55	+	MCPH1	rs2044744		1,00E-012
reg_CTRL	chr8	6391663	G	C	52	100,00%	homvar	52	+	MCPH1	rs2515489		1,00E-012
reg_CTRL	chr8	6391722	A	G	50	98,00%	homvar	49	+	MCPH1	rs2442595		1,00E-012
reg_CTRL	chr8	6392028	A	T	53	100,00%	homvar	53	+	MCPH1	rs2442594		1,00E-012
reg_CTRL	chr8	6392149	T	C	48	100,00%	homvar	48	+	MCPH1	rs2442593		1,00E-012
reg_CTRL	chr8	6392271	T	C	37	97,00%	homvar	36	+	MCPH1	rs2515490		1,00E-012
reg_CTRL	chr8	6393016	C	T	36	100,00%	homvar	36	+	MCPH1	rs2442592		1,00E-012
reg_CTRL	chr8	6393121	G	A	35	100,00%	homvar	35	+	MCPH1	rs2515492		1,00E-012
reg_CTRL	chr8	6393980	C	A	61	100,00%	homvar	61	+	MCPH1	rs2515493		1,00E-012
reg_CTRL	chr8	6394533	T	C	17	100,00%	homvar	17	+	MCPH1	rs2515494		1,00E-012
reg_CTRL	chr8	6397787	C	T	46	98,00%	homvar	45	+	MCPH1	rs2515497		1,00E-012

reg_CTRL	chr8	6399673	G	C	45	56,00%	het	25			+	MCPH1	rs2515499		1,00E-012
reg_CTRL	chr8	6399828	G	C	56	98,00%	homvar	55			+	MCPH1	rs2515500		1,00E-012
reg_CTRL	chr8	6400033	C	T	59	54,00%	het	32			+	MCPH1	rs2515501		1,00E-012
reg_CTRL	chr8	6400140	G	A	48	98,00%	homvar	47			+	MCPH1	rs2515502		1,00E-012
reg_CTRL	chr8	6400377	A	G	15	93,00%	homvar	14			+	MCPH1	rs2922883		1,00E-012
reg_CTRL	chr8	6400446	T	C	16	94,00%	homvar	15			+	MCPH1	rs2515503		1,00E-012
reg_CTRL	chr8	6401634	C	T	21	100,00%	homvar	21			+	MCPH1	rs2515504		1,00E-012
reg_CTRL	chr8	6404202	A	G	21	14,00%	ambig	3			+	MCPH1			1,19E-002
reg_CTRL	chr8	6404255	A	G	26	96,00%	homvar	25			+	MCPH1	rs2515505		1,00E-012
reg_CTRL	chr8	6406668	T	G	39	97,00%	homvar	38			+	MCPH1	rs2515506		1,00E-012
reg_CTRL	chr8	6407942	A	G	45	96,00%	homvar	43			+	MCPH1	rs3739391		1,00E-012
reg_CTRL	chr8	6653493	G	A	6	67,00%	het	4			-	XKR5	rs9693931		4,04E-006
reg_CTRL	chr8	6653898	G	A	48	46,00%	het	22			-	XKR5	rs17078203		1,00E-012
reg_CTRL	chr8	6653994	A	G	38	45,00%	het	17			-	XKR5	rs9314611		1,00E-012
reg_CTRL	chr8	6657359	T	C	62	50,00%	het	31			-	XKR5	rs2553728		1,00E-012
reg_CTRL	chr8	6661868	A	G	34	59,00%	het	20			-	XKR5	rs9773025		1,00E-012
reg_CTRL	chr8	6664775	C	T	22	27,00%	het	6			-	XKR5		rs72107959	8,04E-006
reg_CTRL	chr8	6664793	T	C	22	18,00%	ambig	4			-	XKR5			1,47E-003
reg_CTRL	chr8	6665852	T	A	40	42,00%	het	17			-	XKR5	rs13259050		1,00E-012
reg_CTRL	chr8	6666726	C	A	60	37,00%	het	22			-	XKR5		rs58387909	1,00E-012
reg_CTRL	chr8	6667128	A	T	31	52,00%	het	16			-	XKR5	rs28564455		1,00E-012
reg_CTRL	chr8	6667206	C	G	44	52,00%	het	23			-	XKR5	rs9774066		1,00E-012
reg_CTRL	chr8	6667776	A	T	71	51,00%	het	36			-	XKR5	rs11137070		1,00E-012
reg_CTRL	chr8	6668059	C	T	70	50,00%	het	35			-	XKR5	rs12678222		1,00E-012
reg_CTRL	chr8	6668168	C	A	60	57,00%	het	34			-	XKR5	rs11137071		1,00E-012
reg_CTRL	chr8	6668666	A	C	52	48,00%	het	25			-	XKR5	rs9772979		1,00E-012
reg_CTRL	chr8	6671038	G	A	56	39,00%	het	22			-	XKR5		rs57045395	1,00E-012
reg_CTRL	chr8	6671291	G	T	71	46,00%	het	33			-	XKR5	rs28578619		1,00E-012
reg_CTRL	chr8	6671411	A	G	66	36,00%	het	24			-	XKR5	rs4841770		1,00E-012
reg_CTRL	chr8	6672148	A	G	42	100,00%	homvar	42			-	XKR5	rs2980958		1,00E-012
reg_CTRL	chr8	6672229	T	G	48	100,00%	homvar	48			-	XKR5	rs2980957		1,00E-012
reg_CTRL	chr8	6672308	C	G	51	98,00%	homvar	50			-	XKR5	rs2980956		1,00E-012
reg_CTRL	chr8	6672920	A	G	28	11,00%	ambig	3			-	XKR5			2,60E-002
reg_CTRL	chr8	6674201	C	T	64	53,00%	het	34			-	XKR5	rs2741087		1,00E-012
reg_CTRL	chr8	6675647	T	C	48	50,00%	het	24			-	XKR5	rs2741089		1,00E-012
reg_CTRL	chr8	6675775	G	C	36	53,00%	het	19			-	XKR5	rs2978903		1,00E-012
reg_CTRL	chr8	6676673	T	C	43	58,00%	het	25			-	XKR5	rs2741091		1,00E-012
reg_CTRL	chr8	6677583	G	C	45	49,00%	het	22			-	XKR5	rs2978902		1,00E-012
reg_CTRL	chr8	6677625	G	C	46	48,00%	het	22			-	XKR5	rs2978901		1,00E-012
reg_CTRL	chr8	6677686	T	C	36	53,00%	het	19	M	V	-2	XKR5	rs2741098		1,00E-012
reg_CTRL	chr8	6678708	T	C	18	39,00%	het	7			-	XKR5	rs2978900		8,67E-008
reg_CTRL	chr8	6679008	C	G	18	44,00%	het	8			-	XKR5	rs2978899		2,79E-009
reg_DEFA	chr8	6717829	A	G	49	100,00%	homvar	49			-	DEFB1	rs2980928		1,00E-012
reg_DEFA	chr8	6717856	A	G	48	100,00%	homvar	48			-	DEFB1	rs2980927		1,00E-012
reg_DEFA	chr8	6717956	T	C	53	100,00%	homvar	53			-	DEFB1	rs2977779		1,00E-012
reg_DEFA	chr8	6717999	G	A	52	96,00%	homvar	50			-	DEFB1	rs2977778		1,00E-012
reg_DEFA	chr8	6718012	T	G	57	96,00%	homvar	55			-	DEFB1	rs2980926		1,00E-012
reg_DEFA	chr8	6718140	C	G	59	98,00%	homvar	58			-	DEFB1	rs2978872		1,00E-012
reg_DEFA	chr8	6718172	G	A	63	97,00%	homvar	61			-	DEFB1	rs2977777		1,00E-012
reg_DEFA	chr8	6718680	C	T	32	100,00%	homvar	32			-	DEFB1	rs2978870		1,00E-012
reg_DEFA	chr8	6718808	T	A	24	100,00%	homvar	24			-	DEFB1	rs2977776		1,00E-012
reg_DEFA	chr8	6718836	A	T	24	100,00%	homvar	24			-	DEFB1	rs2951854		1,00E-012
reg_DEFA	chr8	6718897	G	A	24	96,00%	homvar	23			-	DEFB1	rs2741127		1,00E-012

reg_DEFA	chr8	6718913	A	C	26	100,00%	homvar	26	-	DEFB1	rs2927345		1,00E-012
reg_DEFA	chr8	6719066	C	T	33	24,00%	ambig	8	-	DEFB1	rs10528208		6,50E-007
reg_DEFA	chr8	6719161	C	A	45	100,00%	homvar	45	-	DEFB1	rs2978869		1,00E-012
reg_DEFA	chr8	6719186	T	C	49	100,00%	homvar	49	-	DEFB1	rs2977774		1,00E-012
reg_DEFA	chr8	6719218	C	A	52	100,00%	homvar	52	-	DEFB1	rs2978868		1,00E-012
reg_DEFA	chr8	6719403	A	G	41	100,00%	homvar	41	-	DEFB1	rs2980924		1,00E-012
reg_DEFA	chr8	6719467	G	C	39	97,00%	homvar	38	-	DEFB1	rs5743465	rs34929240	1,00E-012
reg_DEFA	chr8	6719468	T	A	39	100,00%	homvar	39	-	DEFB1	rs34929240	rs5743464	1,00E-012
reg_DEFA	chr8	6719584	G	T	29	100,00%	homvar	29	-	DEFB1	rs5743463		1,00E-012
reg_DEFA	chr8	6719596	A	G	28	100,00%	homvar	28	-	DEFB1	rs5743462		1,00E-012
reg_DEFA	chr8	6719656	G	A	18	100,00%	homvar	18	-	DEFB1	rs2978867		1,00E-012
reg_DEFA	chr8	6719740	C	T	13	100,00%	homvar	13	-	DEFB1	rs2978866		1,00E-012
reg_DEFA	chr8	6720060	G	A	23	100,00%	homvar	23	-	DEFB1	rs2977773		1,00E-012
reg_DEFA	chr8	6720108	T	C	34	100,00%	homvar	34	-	DEFB1	rs2951855		1,00E-012
reg_DEFA	chr8	6720467	A	G	57	91,00%	homvar	52	-	DEFB1	rs2980923		1,00E-012
reg_DEFA	chr8	6720606	G	A	65	100,00%	homvar	65	-	DEFB1	rs2977772		1,00E-012
reg_DEFA	chr8	6720679	G	A	69	100,00%	homvar	69	-	DEFB1	rs2741129		1,00E-012
reg_DEFA	chr8	6721130	T	C	32	100,00%	homvar	32	-	DEFB1	rs2978864	rs5743441	1,00E-012
reg_DEFA	chr8	6721183	C	T	24	100,00%	homvar	24	-	DEFB1	rs5743440		1,00E-012
reg_DEFA	chr8	6721210	C	A	20	90,00%	homvar	18	-	DEFB1	rs5743439		1,00E-012
reg_DEFA	chr8	6721278	T	C	16	87,00%	homvar	14	-	DEFB1	rs5743437		1,00E-012
reg_DEFA	chr8	6721755	A	C	12	50,00%	het	6	-	DEFB1	rs2980921		1,21E-007
reg_DEFA	chr8	6722097	T	C	36	100,00%	homvar	36	-	DEFB1	rs2702945		1,00E-012
reg_DEFA	chr8	6722258	G	C	23	96,00%	homvar	22	-	DEFB1	rs2293960		1,00E-012
reg_DEFA	chr8	6722484	C	T	16	100,00%	homvar	16	-	DEFB1	rs2293959		1,00E-012
reg_DEFA	chr8	6722710	T	A	19	100,00%	homvar	19	-	DEFB1	rs2293958		1,00E-012
reg_DEFA	chr8	6722833	C	G	24	100,00%	homvar	24	-	DEFB1	rs1800972		1,00E-012
reg_DEFA	chr8	6722841	C	T	24	92,00%	homvar	22	-	DEFB1	rs1799946		1,00E-012
reg_DEFA	chr8	6723179	A	T	32	100,00%	homvar	32			rs2738182		1,00E-012
reg_DEFA	chr8	6723399	C	T	19	100,00%	homvar	19			rs2741132		1,00E-012
reg_DEFA	chr8	6723423	G	C	18	100,00%	homvar	18			rs2702876	rs7838626	1,00E-012
reg_DEFA	chr8	6723465	G	T	17	100,00%	homvar	17			rs2741133		1,00E-012
reg_DEFA	chr8	6723477	G	C	17	100,00%	homvar	17			rs2702877		1,00E-012
reg_DEFA	chr8	6723483	T	C	18	100,00%	homvar	18			rs2977829		1,00E-012
reg_DEFA	chr8	6723520	G	A	21	100,00%	homvar	21			rs2741134		1,00E-012
reg_DEFA	chr8	6723531	C	T	22	100,00%	homvar	22			rs2978863		1,00E-012
reg_DEFA	chr8	6723664	G	A	26	96,00%	homvar	25			rs2741135		1,00E-012
reg_DEFA	chr8	6723906	A	T	19	100,00%	homvar	19			rs2738181		1,00E-012
reg_DEFA	chr8	6723908	C	T	19	100,00%	homvar	19			rs2738180		1,00E-012
reg_DEFA	chr8	6723927	T	G	18	100,00%	homvar	18			rs2738179		1,00E-012
reg_DEFA	chr8	6723930	A	C	18	100,00%	homvar	18			rs2738178		1,00E-012
reg_DEFA	chr8	6725291	G	C	26	100,00%	homvar	26			rs2702881		1,00E-012
reg_DEFA	chr8	6725524	T	C	25	84,00%	homvar	21			rs2738177		1,00E-012
reg_DEFA	chr8	6725600	G	T	20	100,00%	homvar	20			rs2741138		1,00E-012
reg_DEFA	chr8	6725873	G	A	9	100,00%	homvar	9			rs11990152		1,00E-012
reg_DEFA	chr8	6726157	A	G	6	100,00%	homvar	6			rs2741139		1,48E-010
reg_DEFA	chr8	6726180	A	G	6	100,00%	homvar	6			rs2741140		1,48E-010
reg_DEFA	chr8	6726302	T	C	12	100,00%	homvar	12			rs2702884		1,00E-012
reg_DEFA	chr8	6726851	T	C	26	100,00%	homvar	26			rs2738175		1,00E-012
reg_DEFA	chr8	6727011	G	A	47	96,00%	homvar	45			rs2741141		1,00E-012
reg_DEFA	chr8	6727167	C	T	60	95,00%	homvar	57			rs2977827		1,00E-012
reg_DEFA	chr8	6727332	G	A	73	99,00%	homvar	72			rs2978861		1,00E-012
reg_DEFA	chr8	6727393	T	C	84	99,00%	homvar	83			rs2978860		1,00E-012

reg_DEFA	chr8	6727484	C	T	76	100,00%	homvar	76	rs2977826		1,00E-012
reg_DEFA	chr8	6727601	C	T	62	98,00%	homvar	61	rs2977825		1,00E-012
reg_DEFA	chr8	6727818	G	A	11	100,00%	homvar	11	rs9692818	rs9694351	1,00E-012
reg_DEFA	chr8	6727819	T	C	11	100,00%	homvar	11	rs9694351		1,00E-012
reg_DEFA	chr8	6727833	A	C	12	92,00%	homvar	11	rs9694106		1,00E-012
reg_DEFA	chr8	6727846	A	G	13	100,00%	homvar	13	rs9694107		1,00E-012
reg_DEFA	chr8	6727902	A	G	29	100,00%	homvar	29	rs9694118		1,00E-012
reg_DEFA	chr8	6728005	G	T	39	100,00%	homvar	39	rs2978859		1,00E-012
reg_DEFA	chr8	6728154	C	T	36	100,00%	homvar	36	rs2977824		1,00E-012
reg_DEFA	chr8	6728378	A	G	31	100,00%	homvar	31	rs2741143		1,00E-012
reg_DEFA	chr8	6728397	C	T	29	100,00%	homvar	29	rs2738172		1,00E-012
reg_DEFA	chr8	6728537	T	C	25	100,00%	homvar	25	rs2738171		1,00E-012
reg_DEFA	chr8	6728715	T	G	19	100,00%	homvar	19	rs2978857		1,00E-012
reg_DEFA	chr8	6728719	A	G	17	100,00%	homvar	17	rs2951840		1,00E-012
reg_DEFA	chr8	6728764	C	T	13	85,00%	homvar	11	rs2980919		1,00E-012
reg_DEFA	chr8	6728770	T	C	13	100,00%	homvar	13	rs2978856		1,00E-012
reg_DEFA	chr8	6728867	G	C	11	82,00%	homvar	9	rs2978855		1,00E-012
reg_DEFA	chr8	6729141	A	G	4	100,00%	homvar	4	rs2977823		2,80E-007
reg_DEFA	chr8	6729213	C	A	4	100,00%	homvar	4	rs2977822		2,80E-007
reg_DEFA	chr8	6729311	T	C	9	100,00%	homvar	9	rs2978853		1,00E-012
reg_DEFA	chr8	6729458	C	T	19	95,00%	homvar	18	rs2977821		1,00E-012
reg_DEFA	chr8	6729487	C	T	20	45,00%	het	9			2,44E-010
reg_DEFA	chr8	6729520	C	T	25	100,00%	homvar	25	rs2951850		1,00E-012
reg_DEFA	chr8	6729801	G	A	52	50,00%	het	26	rs2978852		1,00E-012
reg_DEFA	chr8	6730978	A	G	20	100,00%	homvar	20	rs2738165		1,00E-012
reg_DEFA	chr8	6731383	T	C	43	100,00%	homvar	43	rs2738164		1,00E-012
reg_DEFA	chr8	6732626	C	T	41	98,00%	homvar	40	rs2738163		1,00E-012
reg_DEFA	chr8	6734051	A	G	51	100,00%	homvar	51	rs2741718		1,00E-012
reg_DEFA	chr8	6734149	G	A	53	96,00%	homvar	51	rs2978851		1,00E-012
reg_DEFA	chr8	6735296	G	A	42	50,00%	het	21	rs2741717		1,00E-012
reg_DEFA	chr8	6736007	C	T	39	100,00%	homvar	39	rs2738161		1,00E-012
reg_DEFA	chr8	6736627	G	C	33	45,00%	het	15	rs2741149	rs10089905	1,00E-012
reg_DEFA	chr8	6736973	G	A	32	47,00%	het	15	rs4841787		1,00E-012
reg_DEFA	chr8	6737079	G	A	30	100,00%	homvar	30	rs2738158		1,00E-012
reg_DEFA	chr8	6737740	T	G	30	40,00%	het	12			2,51E-012
reg_DEFA	chr8	6737799	T	A	26	46,00%	het	12			1,00E-012
reg_DEFA	chr8	6738298	A	G	11	91,00%	homvar	10	rs2978964		1,00E-012
reg_DEFA	chr8	6738591	G	A	28	100,00%	homvar	28	rs2951870		1,00E-012
reg_DEFA	chr8	6738844	T	C	47	100,00%	homvar	47	rs2702936		1,00E-012
reg_DEFA	chr8	6739237	A	G	53	92,00%	homvar	49	rs2702935		1,00E-012
reg_DEFA	chr8	6739310	T	G	52	85,00%	homvar	44	rs2738153		1,00E-012
reg_DEFA	chr8	6739495	T	A	56	100,00%	homvar	56	rs2738152		1,00E-012
reg_DEFA	chr8	6741958	C	A	37	97,00%	homvar	36	rs751009		1,00E-012
reg_DEFA	chr8	6743931	T	C	8	100,00%	homvar	8	rs2980959		1,00E-012
reg_DEFA	chr8	6743987	A	G	8	100,00%	homvar	8	rs13275881		1,00E-012
reg_DEFA	chr8	6743992	G	C	8	100,00%	homvar	8	rs13272703		1,00E-012
reg_DEFA	chr8	6744124	T	C	11	91,00%	homvar	10	rs13255674		1,00E-012
reg_DEFA	chr8	6744173	A	C	15	100,00%	homvar	15	rs9693852		1,00E-012
reg_DEFA	chr8	6744180	T	C	15	100,00%	homvar	15	rs13255719		1,00E-012
reg_DEFA	chr8	6744212	G	A	17	100,00%	homvar	17	rs2741057		1,00E-012
reg_DEFA	chr8	6745807	G	A	44	100,00%	homvar	44	rs2702929		1,00E-012
reg_DEFA	chr8	6745889	A	C	42	100,00%	homvar	42	rs2702930		1,00E-012
reg_DEFA	chr8	6746226	G	A	37	100,00%	homvar	37	rs2738143		1,00E-012

reg_DEFA	chr8	6746266	A	T	31	100,00%	homvar	31			rs2741060		1,00E-012
reg_DEFA	chr8	6746389	A	C	24	100,00%	homvar	24			rs2702931		1,00E-012
reg_DEFA	chr8	6746793	T	C	9	100,00%	homvar	9			rs2702932		1,00E-012
reg_DEFA	chr8	6746980	C	G	9	100,00%	homvar	9			rs2741061		1,00E-012
reg_DEFA	chr8	6747103	C	G	11	100,00%	homvar	11			rs2741062		1,00E-012
reg_DEFA	chr8	6747377	C	A	22	91,00%	homvar	20			rs2741714		1,00E-012
reg_DEFA	chr8	6747535	A	C	27	100,00%	homvar	27			rs2951867		1,00E-012
reg_DEFA	chr8	6747552	T	C	28	96,00%	homvar	27			rs2702933		1,00E-012
reg_DEFA	chr8	6748150	G	C	47	17,00%	ambig	8					1,10E-005
reg_DEFA	chr8	6750581	T	C	32	100,00%	homvar	32			rs2981405		1,00E-012
reg_DEFA	chr8	6750651	A	G	40	100,00%	homvar	40			rs2741073		1,00E-012
reg_DEFA	chr8	6751207	C	T	49	100,00%	homvar	49			rs2741075		1,00E-012
reg_DEFA	chr8	6751396	T	C	43	100,00%	homvar	43			rs2741710		1,00E-012
reg_DEFA	chr8	6754935	T	C	53	51,00%	het	27				rs73194190	1,00E-012
reg_DEFA	chr8	6757796	A	G	33	97,00%	homvar	32			rs10216819		1,00E-012
reg_DEFA	chr8	6758791	T	C	23	100,00%	homvar	23			rs2981401		1,00E-012
reg_DEFA	chr8	6758869	C	T	22	45,00%	het	10					3,38E-011
reg_DEFA	chr8	6760323	T	G	38	97,00%	homvar	37			rs7461956		1,00E-012
reg_DEFA	chr8	6761967	G	A	71	100,00%	homvar	71			rs35200859		1,00E-012
reg_DEFA	chr8	6762964	G	A	46	98,00%	homvar	45			rs2741699		1,00E-012
reg_DEFA	chr8	6762978	C	A	44	82,00%	homvar	36			rs2741698		1,00E-012
reg_DEFA	chr8	6763722	G	A	49	96,00%	homvar	47			rs2741695		1,00E-012
reg_DEFA	chr8	6764461	A	G	35	97,00%	homvar	34			rs2741694		1,00E-012
reg_DEFA	chr8	6764692	T	C	40	10,00%	ambig	4					1,32E-002
reg_DEFA	chr8	6764855	C	T	44	52,00%	het	23					1,00E-012
reg_DEFA	chr8	6765363	G	A	22	100,00%	homvar	22			rs2702946		1,00E-012
reg_DEFA	chr8	6765676	A	G	22	91,00%	homvar	20			rs12545953		1,00E-012
reg_DEFA	chr8	6765994	T	C	30	97,00%	homvar	29			rs2738129		1,00E-012
reg_DEFA	chr8	6766076	A	C	30	100,00%	homvar	30			rs2738128		1,00E-012
reg_DEFA	chr8	6766524	T	C	31	100,00%	homvar	31			rs2978959		1,00E-012
reg_DEFA	chr8	6766756	T	A	24	96,00%	homvar	23			rs13265468		1,00E-012
reg_DEFA	chr8	6767209	C	T	31	100,00%	homvar	31			rs4294209		1,00E-012
reg_DEFA	chr8	6767654	T	C	32	97,00%	homvar	31			rs13275170		1,00E-012
reg_DEFA	chr8	6767778	G	T	20	100,00%	homvar	20			rs2738125		1,00E-012
reg_DEFA	chr8	6767996	T	C	4	100,00%	homvar	4			rs13276112		2,80E-007
reg_DEFA	chr8	6768647	T	A	19	95,00%	homvar	18			rs2702905		1,00E-012
reg_DEFA	chr8	6768913	G	A	21	100,00%	homvar	21			rs7826006		1,00E-012
reg_DEFA	chr8	6769030	T	C	17	100,00%	homvar	17			rs2738122		1,00E-012
reg_DEFA	chr8	6769096	T	C	15	100,00%	homvar	15			rs2738121		1,00E-012
reg_DEFA	chr8	6769372	G	A	39	90,00%	homvar	35			rs3888152		1,00E-012
reg_DEFA	chr8	6770046	G	C	77	96,00%	homvar	74	-	DEFA6	rs2738120		1,00E-012
reg_DEFA	chr8	6770127	G	C	57	96,00%	homvar	55	-	DEFA6	rs2738119		1,00E-012
reg_DEFA	chr8	6770376	G	T	27	100,00%	homvar	27	-	DEFA6	rs2741691		1,00E-012
reg_DEFA	chr8	6771046	G	T	40	100,00%	homvar	40			rs11784359		1,00E-012
reg_DEFA	chr8	6771370	C	G	40	100,00%	homvar	40			rs4458901		1,00E-012
reg_DEFA	chr8	6771627	C	G	30	100,00%	homvar	30			rs2741690		1,00E-012
reg_DEFA	chr8	6771666	C	T	27	100,00%	homvar	27			rs2741689		1,00E-012
reg_DEFA	chr8	6772381	G	T	26	100,00%	homvar	26			rs2738118		1,00E-012
reg_DEFA	chr8	6773483	G	C	59	100,00%	homvar	59			rs2741686		1,00E-012
reg_DEFA	chr8	6774191	T	C	14	93,00%	homvar	13			rs3918350		1,00E-012
reg_DEFA	chr8	6774412	C	G	11	100,00%	homvar	11			rs34502430		1,00E-012
reg_DEFA	chr8	6774485	T	C	9	100,00%	homvar	9			rs2702938		1,00E-012
reg_DEFA	chr8	6774810	T	C	13	92,00%	homvar	12			rs2702939		1,00E-012

reg_DEFA	chr8	6775470	G	A	20	95,00%	homvar	19					rs2012832	rs2738114		1,00E-012
reg_DEFA	chr8	6775828	C	G	28	96,00%	homvar	27					rs2741684			1,00E-012
reg_DEFA	chr8	6775890	T	C	30	100,00%	homvar	30					rs2741683			1,00E-012
reg_DEFA	chr8	6776054	A	G	24	100,00%	homvar	24					rs2738111			1,00E-012
reg_DEFA	chr8	6776610	T	C	35	97,00%	homvar	34					rs2702855			1,00E-012
reg_DEFA	chr8	6776698	C	G	40	52,00%	het	21					rs3887306			1,00E-012
reg_DEFA	chr8	6777064	A	T	30	100,00%	homvar	30					rs2738109			1,00E-012
reg_DEFA	chr8	6777196	T	C	42	100,00%	homvar	42					rs2702858			1,00E-012
reg_DEFA	chr8	6777240	G	A	42	69,00%	het	29					rs2977818			1,00E-012
reg_DEFA	chr8	6777294	G	A	45	58,00%	het	26					rs13274891			1,00E-012
reg_DEFA	chr8	6777394	T	C	47	57,00%	het	27					rs13250769			1,00E-012
reg_DEFA	chr8	6777652	C	T	49	33,00%	het	16					rs2702860			1,00E-012
reg_DEFA	chr8	6777734	T	C	55	91,00%	homvar	50					rs2702861			1,00E-012
reg_DEFA	chr8	6777846	G	A	64	41,00%	het	26								1,00E-012
reg_DEFA	chr8	6777962	C	T	57	56,00%	het	32							rs56248548	1,00E-012
reg_DEFA	chr8	6778120	C	A	27	52,00%	het	14					rs13251447			1,00E-012
reg_DEFA	chr8	6778219	T	C	6	100,00%	homvar	6					rs2741682			1,48E-010
reg_DEFA	chr8	6779011	A	G	22	36,00%	het	8					rs13262140			1,88E-008
reg_DEFA	chr8	6779125	A	G	24	100,00%	homvar	24					rs2738106			1,00E-012
reg_DEFA	chr8	6779205	C	T	25	48,00%	het	12								1,00E-012
reg_DEFA	chr8	6779250	C	T	30	43,00%	het	13					rs13261705			1,00E-012
reg_DEFA	chr8	6779667	G	A	32	100,00%	homvar	32					rs2738104			1,00E-012
reg_DEFA	chr8	6779811	C	T	32	53,00%	het	17					rs13263461			1,00E-012
reg_DEFA	chr8	6779861	C	G	29	52,00%	het	15					rs13263510			1,00E-012
reg_DEFA	chr8	6779930	G	A	33	91,00%	homvar	30					rs2738103			1,00E-012
reg_DEFA	chr8	6779979	C	T	36	33,00%	het	12					rs2702866			3,63E-011
reg_DEFA	chr8	6780050	A	G	45	64,00%	het	29					rs13254588			1,00E-012
reg_DEFA	chr8	6780152	G	T	57	54,00%	het	31					rs13251814			1,00E-012
reg_DEFA	chr8	6780481	G	A	71	100,00%	homvar	71					rs2738102			1,00E-012
reg_DEFA	chr8	6780732	T	C	67	94,00%	homvar	63					rs2702867			1,00E-012
reg_DEFA	chr8	6780950	C	T	68	96,00%	homvar	65			-	DEFA4	rs736227			1,00E-012
reg_DEFA	chr8	6780991	A	G	63	46,00%	het	29	G	G	-2	DEFA4	rs2738100			1,00E-012
reg_DEFA	chr8	6781429	G	A	48	100,00%	homvar	48			-	DEFA4	rs2239668			1,00E-012
reg_DEFA	chr8	6781617	G	A	41	63,00%	het	26			-	DEFA4	rs2239667			1,00E-012
reg_DEFA	chr8	6781843	C	T	21	57,00%	het	12			-	DEFA4			rs56007429	1,00E-012
reg_DEFA	chr8	6782277	T	A	47	100,00%	homvar	47			-	DEFA4	rs2741679			1,00E-012
reg_DEFA	chr8	6782499	G	A	67	51,00%	het	34			-	DEFA4	rs2741678			1,00E-012
reg_DEFA	chr8	6783452	A	G	34	44,00%	het	15					rs2738098			1,00E-012
reg_DEFA	chr8	6783611	C	T	34	56,00%	het	19					rs2741676			1,00E-012
reg_DEFA	chr8	6784021	C	T	26	35,00%	het	9					rs45482601			3,95E-009
reg_DEFA	chr8	6784715	G	A	40	50,00%	het	20								1,00E-012
reg_DEFA	chr8	6784942	C	A	51	98,00%	homvar	50					rs2741675			1,00E-012
reg_DEFA	chr8	6785122	C	T	43	42,00%	het	18					rs2741674			1,00E-012
reg_DEFA	chr8	6785597	G	A	68	50,00%	het	34					rs3890000			1,00E-012
reg_DEFA	chr8	6786255	G	A	61	51,00%	het	31								1,00E-012
reg_DEFA	chr8	6786308	C	G	53	49,00%	het	26					rs2615772			1,00E-012
reg_DEFA	chr8	6786381	C	A	44	48,00%	het	21					rs2741673			1,00E-012
reg_DEFA	chr8	6786918	G	T	35	51,00%	het	18					rs11991138			1,00E-012
reg_DEFA	chr8	6786956	G	C	35	54,00%	het	19								1,00E-012
reg_DEFA	chr8	6787913	C	T	14	57,00%	het	8					rs34431957			2,09E-010
reg_DEFA	chr8	6788129	C	G	17	53,00%	het	9					rs2951853			5,40E-011
reg_DEFA	chr8	6788410	C	T	25	60,00%	het	15					rs13263953			1,00E-012
reg_DEFA	chr8	6788432	C	T	25	56,00%	het	14					rs13263967			1,00E-012

reg_DEFA	chr8	6789122	A	G	28	54,00%	het	15			rs2981398		1,00E-012
reg_DEFA	chr8	6789483	T	C	21	43,00%	het	9			rs2741669		4,19E-010
reg_DEFA	chr8	6789749	G	T	42	45,00%	het	19			rs35900031		1,00E-012
reg_DEFA	chr8	6790937	T	C	52	58,00%	het	30			rs2741668		1,00E-012
reg_DEFA	chr8	6791083	C	A	43	33,00%	het	14					1,00E-012
reg_DEFA	chr8	6791260	C	T	49	57,00%	het	28			rs2981396		1,00E-012
reg_DEFA	chr8	6792879	A	G	54	100,00%	homvar	54			rs2951844		1,00E-012
reg_DEFA	chr8	6793624	A	G	49	100,00%	homvar	49			rs2702879		1,00E-012
reg_DEFA	chr8	6793699	C	T	42	38,00%	het	16			rs2741665		1,00E-012
reg_DEFA	chr8	6793802	C	T	34	50,00%	het	17					1,00E-012
reg_DEFA	chr8	6794668	A	G	35	51,00%	het	18			rs7016650		1,00E-012
reg_DEFA	chr8	6794720	T	G	35	54,00%	het	19			rs2978955		1,00E-012
reg_DEFA	chr8	6795249	C	T	56	41,00%	het	23			rs4840645		1,00E-012
reg_DEFA	chr8	6795351	A	G	46	37,00%	het	17			rs4840646		1,00E-012
reg_DEFA	chr8	6795430	A	T	43	51,00%	het	22			rs2741661		1,00E-012
reg_DEFA	chr8	6795791	T	A	45	20,00%	ambig	9			rs6998500		7,54E-007
reg_DEFA	chr8	6796132	T	G	54	56,00%	het	30			rs2075836		1,00E-012
reg_DEFA	chr8	6796298	C	T	55	42,00%	het	23			rs2075835		1,00E-012
reg_DEFA	chr8	6797176	C	G	67	52,00%	het	35					1,00E-012
reg_DEFA	chr8	6797225	T	C	69	49,00%	het	34			rs35237181		1,00E-012
reg_DEFA	chr8	6797578	T	C	43	49,00%	het	21			rs2741659		1,00E-012
reg_DEFA	chr8	6797707	C	T	37	46,00%	het	17			rs4841789		1,00E-012
reg_DEFA	chr8	6798559	T	G	35	57,00%	het	20			rs2615787		1,00E-012
reg_DEFA	chr8	6798969	C	T	53	51,00%	het	27			rs2741658		1,00E-012
reg_DEFA	chr8	6799608	A	G	31	100,00%	homvar	31			rs2741657		1,00E-012
reg_DEFA	chr8	6799659	G	T	30	100,00%	homvar	30			rs2472562		1,00E-012
reg_DEFA	chr8	6800003	T	C	17	18,00%	ambig	3					6,50E-003
reg_DEFA	chr8	6800015	T	C	18	17,00%	ambig	3					7,66E-003
reg_DEFA	chr8	6800090	G	T	28	46,00%	het	13				rs35000792	1,00E-012
reg_DEFA	chr8	6800169	A	G	40	50,00%	het	20			rs2615771		1,00E-012
reg_DEFA	chr8	6800911	T	G	41	34,00%	het	14			rs2702852		1,00E-012
reg_DEFA	chr8	6805553	T	C	43	56,00%	het	24			rs4322031		1,00E-012
reg_DEFA	chr8	6806978	A	G	11	36,00%	het	4				rs57688776	8,11E-005
reg_DEFA	chr8	6807665	T	C	24	29,00%	het	7					8,35E-007
reg_DEFA	chr8	6808829	T	C	69	100,00%	homvar	69			rs2977793		1,00E-012
reg_DEFA	chr8	6809027	T	C	76	97,00%	homvar	74			rs2738058		1,00E-012
reg_DEFA	chr8	6809450	G	A	69	99,00%	homvar	68			rs2951869		1,00E-012
reg_DEFA	chr8	6809904	T	C	27	93,00%	homvar	25			rs2977789		1,00E-012
reg_DEFA	chr8	6809966	A	C	27	100,00%	homvar	27			rs2977788		1,00E-012
reg_DEFA	chr8	6810016	C	T	27	26,00%	het	7			rs2615768		2,02E-006
reg_DEFA	chr8	6810195	A	G	25	28,00%	het	7			rs2738048		1,14E-006
reg_DEFA	chr8	6810705	A	G	54	96,00%	homvar	52			rs2978951		1,00E-012
reg_DEFA	chr8	6810952	C	A	62	45,00%	het	28			rs2738046		1,00E-012
reg_DEFA	chr8	6810975	C	G	63	44,00%	het	28			rs2702912		1,00E-012
reg_DEFA	chr8	6810978	T	C	64	42,00%	het	27			rs2738045		1,00E-012
reg_DEFA	chr8	6811301	G	A	63	43,00%	het	27			rs2702910		1,00E-012
reg_DEFA	chr8	6811635	T	C	45	47,00%	het	21			rs2702909		1,00E-012
reg_DEFA	chr8	6811987	G	T	25	48,00%	het	12			rs2738168		1,00E-012
reg_DEFA	chr8	6812024	G	A	23	48,00%	het	11			rs2702908		1,66E-012
reg_DEFA	chr8	6812694	G	C	67	54,00%	het	36			rs2978950		1,00E-012
reg_DEFA	chr8	6812705	G	A	66	44,00%	het	29			rs6996047		1,00E-012
reg_DEFA	chr8	6812906	G	C	59	51,00%	het	30			rs11996346		1,00E-012
reg_DEFA	chr8	6813499	A	G	76	97,00%	homvar	74	-	DEFA10P	rs2978947		1,00E-012

reg_DEFA	chr8	6813654	T	C	63	48,00%	het	30	-	DEFA10P	rs2702875			1,00E-012
reg_DEFA	chr8	6814126	A	G	40	40,00%	het	16			rs2978944			1,00E-012
reg_DEFA	chr8	6814709	T	A	60	43,00%	het	26			rs2738135			1,00E-012
reg_DEFA	chr8	6815234	G	A	40	37,00%	het	15			rs2738132			1,00E-012
reg_DEFA	chr8	6816191	T	C	13	62,00%	het	8			rs12682063			9,13E-011
reg_DEFA	chr8	6816201	T	C	14	57,00%	het	8			rs12682076			2,09E-010
reg_DEFA	chr8	6816223	G	A	15	60,00%	het	9			rs12675298			1,11E-011
reg_DEFA	chr8	6816300	T	C	13	54,00%	het	7			rs34914251			5,17E-009
reg_DEFA	chr8	6816495	G	T	13	38,00%	het	5			rs2738113			7,10E-006
reg_DEFA	chr8	6816971	G	A	44	27,00%	het	12						2,54E-010
reg_DEFA	chr8	6816974	C	T	45	27,00%	het	12			rs6989424			3,12E-010
reg_DEFA	chr8	6817024	G	A	61	33,00%	het	20			rs10095450			1,00E-012
reg_DEFA	chr8	6817036	T	C	61	98,00%	homvar	60			rs2738101			1,00E-012
reg_DEFA	chr8	6817114	C	T	95	44,00%	het	42			rs2615789			1,00E-012
reg_DEFA	chr8	6817172	G	A	109	25,00%	het	27			rs10095611			1,00E-012
reg_DEFA	chr8	6817394	C	G	80	30,00%	het	24			rs4841790			1,00E-012
reg_DEFA	chr8	6817403	A	C	80	10,00%	ambig	8					rs57466375	5,21E-004
reg_DEFA	chr8	6817418	C	T	76	43,00%	het	33			rs10096781			1,00E-012
reg_DEFA	chr8	6817429	A	G	75	27,00%	het	20			rs2738096			1,00E-012
reg_DEFA	chr8	6817438	G	A	75	27,00%	het	20			rs2978910			1,00E-012
reg_DEFA	chr8	6817815	C	T	9	89,00%	homvar	8			rs4841791			1,00E-012
reg_DEFA	chr8	6818268	C	T	23	87,00%	homvar	20						1,00E-012
reg_DEFA	chr8	6818340	C	A	37	76,00%	homvar	28						1,00E-012
reg_DEFA	chr8	6818375	G	A	49	98,00%	homvar	48			rs2738089			1,00E-012
reg_DEFA	chr8	6818397	C	T	46	30,00%	het	14			rs2927351			3,56E-012
reg_DEFA	chr8	6818577	T	C	61	34,00%	het	21			rs4082288			1,00E-012
reg_DEFA	chr8	6818799	G	T	46	33,00%	het	15			rs2738083			1,00E-012
reg_DEFA	chr8	6818898	A	G	35	11,00%	ambig	4			rs2927349			8,30E-003
reg_DEFA	chr8	6818923	C	T	33	42,00%	het	14						1,00E-012
reg_DEFA	chr8	6819028	A	C	24	100,00%	homvar	24			rs2978905			1,00E-012
reg_DEFA	chr8	6820385	T	G	43	23,00%	ambig	10						3,96E-008
reg_DEFA	chr8	6820395	G	A	46	20,00%	ambig	9						9,18E-007
reg_DEFA	chr8	6820410	C	T	51	25,00%	het	13						1,16E-010
reg_DEFA	chr8	6820640	C	T	99	15,00%	ambig	15					rs2702861	9,27E-009
reg_DEFA	chr8	6820642	T	G	100	13,00%	ambig	13						5,53E-007
reg_DEFA	chr8	6820920	T	C	64	12,00%	ambig	8						1,10E-004
reg_DEFA	chr8	6820983	G	A	43	100,00%	homvar	43			rs2615779			1,00E-012
reg_DEFA	chr8	6824570	C	A	6	100,00%	homvar	6	-	DEFA1B	rs2979395	rs3758132		1,48E-010
reg_DEFA	chr8	6824643	A	G	6	100,00%	homvar	6	-	DEFA1B	rs2472240	rs41421151		1,48E-010
reg_DEFA	chr8	6824741	G	A	6	100,00%	homvar	6	-	DEFA1B			rs58955451	1,48E-010
reg_DEFA	chr8	6825349	C	T	29	100,00%	homvar	29			rs2978854			1,00E-012
reg_DEFA	chr8	6825357	G	A	29	100,00%	homvar	29			rs2951835			1,00E-012
reg_DEFA	chr8	6825678	A	G	11	100,00%	homvar	11			rs6993352			1,00E-012
reg_DEFA	chr8	6825754	C	T	8	100,00%	homvar	8			rs6986023			1,00E-012
reg_DEFA	chr8	6825821	C	T	5	100,00%	homvar	5						6,44E-009
reg_DEFA	chr8	6843562	A	G	10	90,00%	homvar	9	-	DEFA1B	rs2739219			1,00E-012
reg_DEFA	chr8	6843896	G	A	38	95,00%	homvar	36	-	DEFA1B			rs71509218	1,00E-012
reg_DEFA	chr8	6844623	A	C	9	100,00%	homvar	9			rs28515027			1,00E-012
reg_DEFA	chr8	6846987	A	T	3	100,00%	homvar	3					rs6651509	1,22E-005
reg_DEFA	chr8	6859147	G	T	29	86,00%	homvar	25					rs2615778	1,00E-012
reg_DEFA	chr8	6859203	G	A	43	100,00%	homvar	43			rs28532282			1,00E-012
reg_DEFA	chr8	6859619	T	G	63	100,00%	homvar	63			rs2739221			1,00E-012
reg_DEFA	chr8	6859622	A	G	62	11,00%	ambig	7						5,53E-004

reg_DEFA	chr8	6859843	G	A	71	39,00%	het	28			rs2739220			1,00E-012
reg_DEFA	chr8	6859866	A	G	74	22,00%	ambig	16					rs4841796	1,35E-011
reg_DEFA	chr8	6860180	A	G	66	15,00%	ambig	10						2,69E-006
reg_DEFA	chr8	6860244	G	T	55	24,00%	ambig	13			rs10105163			2,98E-010
reg_DEFA	chr8	6860856	G	T	51	35,00%	het	18	-	DEFA1B	rs4841813			1,00E-012
reg_DEFA	chr8	6861144	T	G	61	49,00%	het	30	-	DEFA1B			rs4840655	1,00E-012
reg_DEFA	chr8	6861165	G	A	66	48,00%	het	32	-	DEFA1B			rs73195997	1,00E-012
reg_DEFA	chr8	6861269	A	T	51	92,00%	homvar	47	-	DEFA1B	rs2702913			1,00E-012
reg_DEFA	chr8	6862083	G	T	26	100,00%	homvar	26	-	DEFA1B				1,00E-012
reg_DEFA	chr8	6862522	A	G	18	94,00%	homvar	17	-	DEFA1B	rs4841798			1,00E-012
reg_DEFA	chr8	6862786	A	C	70	13,00%	ambig	9	-	DEFA1B	rs3758132			3,30E-005
reg_DEFA	chr8	6862950	G	T	79	23,00%	ambig	18	-	DEFA1B	rs34582127	rs2615798		1,00E-012
reg_DEFA	chr8	6864348	C	G	74	41,00%	het	30						1,00E-012
reg_DEFA	chr8	6864445	T	C	75	11,00%	ambig	8					rs62487509	3,35E-004
reg_DEFA	chr8	6864926	T	C	99	45,00%	het	45			rs2739218			1,00E-012
reg_DEFA	chr8	6864943	A	G	96	26,00%	het	25					rs59380237	1,00E-012
reg_DEFA	chr8	6865114	G	C	49	35,00%	het	17			rs4012963			1,00E-012
reg_DEFA	chr8	6865153	G	A	37	27,00%	het	10			rs4012962			8,17E-009
reg_DEFA	chr8	6865330	G	A	43	56,00%	het	24						1,00E-012
reg_DEFA	chr8	6865813	T	C	34	18,00%	ambig	6			rs11781199			1,14E-004
reg_DEFA	chr8	6865875	T	C	29	31,00%	het	9			rs11781205			1,19E-008
reg_DEFA	chr8	6865966	T	C	35	23,00%	ambig	8			rs11781229			1,06E-006
reg_DEFA	chr8	6866059	A	T	40	22,00%	ambig	9					rs56016462	2,58E-007
reg_DEFA	chr8	6866080	G	C	39	21,00%	ambig	8					rs55836016	2,55E-006
reg_DEFA	chr8	6866454	T	G	39	44,00%	het	17					rs59305955	1,00E-012
reg_DEFA	chr8	6866462	G	C	40	42,00%	het	17						1,00E-012
reg_DEFA	chr8	6866888	G	C	10	100,00%	homvar	10			rs35858635			1,00E-012
reg_DEFA	chr8	6866891	G	C	10	100,00%	homvar	10			rs34985860			1,00E-012
reg_DEFA	chr8	6866895	G	C	11	100,00%	homvar	11			rs35564068			1,00E-012
reg_DEFA	chr8	6866897	G	C	11	100,00%	homvar	11			rs35820601			1,00E-012
reg_DEFA	chr8	6866947	G	C	12	100,00%	homvar	12			rs4310228			1,00E-012
reg_DEFA	chr8	6867985	T	C	40	50,00%	het	20			rs4300027			1,00E-012
reg_DEFA	chr8	6868004	C	T	45	51,00%	het	23			rs4512398			1,00E-012
reg_DEFA	chr8	6868335	A	G	39	59,00%	het	23			rs7826487			1,00E-012
reg_DEFA	chr8	6868373	T	C	36	50,00%	het	18			rs6605578			1,00E-012
reg_DEFA	chr8	6868637	C	T	47	55,00%	het	26			rs17078510			1,00E-012
reg_DEFA	chr8	6868805	G	A	50	48,00%	het	24			rs7841223			1,00E-012
reg_DEFA	chr8	6869048	A	G	56	54,00%	het	30			rs4288398			1,00E-012
reg_DEFA	chr8	6869398	T	C	54	48,00%	het	26			rs4313182			1,00E-012
reg_DEFA	chr8	6869803	C	G	53	53,00%	het	28			rs883182			1,00E-012
reg_DEFA	chr8	6869887	A	T	56	98,00%	homvar	55			rs4481622			1,00E-012
reg_DEFA	chr8	6870176	T	G	89	99,00%	homvar	88			rs4314670			1,00E-012
reg_DEFA	chr8	6870220	G	T	95	99,00%	homvar	94			rs4332158			1,00E-012
reg_DEFA	chr8	6870259	G	A	85	53,00%	het	45			rs4332159			1,00E-012
reg_DEFA	chr8	6870678	C	T	53	38,00%	het	20			rs4448290			1,00E-012
reg_DEFA	chr8	6870686	G	C	51	37,00%	het	19			rs4469481			1,00E-012
reg_DEFA	chr8	6870701	A	G	49	35,00%	het	17			rs9774483			1,00E-012
reg_DEFA	chr8	6871262	C	T	32	47,00%	het	15						1,00E-012
reg_DEFA	chr8	6871265	A	G	32	100,00%	homvar	32			rs4840665			1,00E-012
reg_DEFA	chr8	6872534	A	C	50	48,00%	het	24			rs6605579			1,00E-012
reg_DEFA	chr8	6874265	A	T	38	100,00%	homvar	38			rs7821152			1,00E-012
reg_DEFA	chr8	6874382	G	A	28	46,00%	het	13			rs4403430			1,00E-012
reg_DEFA	chr8	6874473	T	G	29	41,00%	het	12			rs11137086			1,00E-012

reg_DEFA	chr8	6875301	C	T	42	55,00%	het	23		rs62487515	1,00E-012
reg_DEFA	chr8	6875366	G	A	46	48,00%	het	22	rs6982814		1,00E-012
reg_DEFA	chr8	6875538	C	A	44	32,00%	het	14		rs56230231	1,00E-012
reg_DEFA	chr8	6875544	T	C	43	44,00%	het	19		rs55851618	1,00E-012
reg_DEFA	chr8	6875673	C	G	39	44,00%	het	17		rs55660132	1,00E-012
reg_DEFA	chr8	6875975	A	G	40	47,00%	het	19		rs55740316	1,00E-012
reg_DEFA	chr8	6877006	A	T	41	44,00%	het	18	rs7011708		1,00E-012
reg_DEFA	chr8	6877045	G	A	46	48,00%	het	22	rs11776120		1,00E-012
reg_DEFA	chr8	6877291	A	G	47	36,00%	het	17	rs11786781		1,00E-012
reg_DEFA	chr8	6877358	G	C	47	40,00%	het	19	rs6993492		1,00E-012
reg_DEFA	chr8	6877487	T	C	50	36,00%	het	18	rs34825638		1,00E-012
reg_DEFA	chr8	6877608	G	A	54	37,00%	het	20	rs7824527		1,00E-012
reg_DEFA	chr8	6877988	G	T	61	51,00%	het	31	rs7825124		1,00E-012
reg_DEFA	chr8	6878106	C	G	55	44,00%	het	24			1,00E-012
reg_DEFA	chr8	6878312	T	C	34	32,00%	het	11	rs4490865		1,76E-010
reg_DEFA	chr8	6879022	T	G	49	94,00%	homvar	46	rs4841816		1,00E-012
reg_DEFA	chr8	6879381	C	G	56	41,00%	het	23	rs4433170		1,00E-012
reg_DEFA	chr8	6879828	A	G	34	53,00%	het	18	rs4602905		1,00E-012
reg_DEFA	chr8	6880925	A	G	17	41,00%	het	7			5,41E-008
reg_DEFA	chr8	6881221	C	T	10	60,00%	het	6		rs11774769	2,87E-008
reg_DEFA	chr8	6881365	G	T	25	48,00%	het	12	rs17078546		1,00E-012
reg_DEFA	chr8	6881460	G	A	40	45,00%	het	18	rs7015200		1,00E-012
reg_DEFA	chr8	6881573	G	A	48	40,00%	het	19	rs11775034		1,00E-012
reg_DEFA	chr8	6881816	T	C	55	49,00%	het	27	rs7004995		1,00E-012
reg_DEFA	chr8	6882227	G	T	27	56,00%	het	15	rs34333583		1,00E-012
reg_DEFA	chr8	6882354	T	A	8	50,00%	het	4	rs35708338		1,82E-005
reg_DEFA	chr8	6882359	G	T	8	50,00%	het	4	rs35866869		1,82E-005
reg_DEFA	chr8	6882374	T	C	7	43,00%	het	3	rs34268546		3,97E-004
reg_DEFA	chr8	6882583	C	G	13	46,00%	het	6		rs2515504	2,21E-007
reg_DEFA	chr8	6882620	T	C	13	46,00%	het	6	rs7009952		2,21E-007
reg_DEFA	chr8	6882937	G	C	39	46,00%	het	18	rs34219797		1,00E-012
reg_DEFA	chr8	6883103	C	T	26	50,00%	het	13	rs11994868		1,00E-012
reg_DEFA	chr8	6883131	T	C	21	52,00%	het	11	rs11985027		1,00E-012
reg_DEFA	chr8	6883151	T	G	16	50,00%	het	8	rs11985030		8,62E-010
reg_DEFA	chr8	6883232	T	C	18	44,00%	het	8	rs11985068		2,79E-009
reg_DEFA	chr8	6883286	T	C	23	52,00%	het	12	rs11985076		1,00E-012
reg_DEFA	chr8	6883469	A	C	35	40,00%	het	14	rs4841817		1,00E-012
reg_DEFA	chr8	6883493	G	T	38	37,00%	het	14	rs4840666		1,00E-012
reg_DEFA	chr8	6883556	G	C	48	42,00%	het	20	rs17078556		1,00E-012
reg_DEFA	chr8	6883571	T	G	50	36,00%	het	18	rs35196527		1,00E-012
reg_DEFA	chr8	6883577	A	G	50	36,00%	het	18	rs34725312		1,00E-012
reg_DEFA	chr8	6883643	C	T	66	42,00%	het	28	rs4621824		1,00E-012
reg_DEFA	chr8	6883724	A	G	79	38,00%	het	30	rs4345578		1,00E-012
reg_DEFA	chr8	6883754	T	C	80	39,00%	het	31	rs4342629		1,00E-012
reg_DEFA	chr8	6883823	G	C	84	99,00%	homvar	83	rs4342630		1,00E-012
reg_DEFA	chr8	6883831	G	C	85	100,00%	homvar	85	rs4342631		1,00E-012
reg_DEFA	chr8	6883942	C	T	82	41,00%	het	34	rs4549798		1,00E-012
reg_DEFA	chr8	6883982	A	C	75	100,00%	homvar	75	rs4342632		1,00E-012
reg_DEFA	chr8	6884242	G	T	42	93,00%	homvar	39	rs4601339		1,00E-012
reg_DEFA	chr8	6884304	G	T	30	100,00%	homvar	30	rs4601340		1,00E-012
reg_DEFA	chr8	6884370	C	A	32	94,00%	homvar	30	rs4642671		1,00E-012
reg_DEFA	chr8	6885310	C	T	32	41,00%	het	13	rs4546682		1,00E-012
reg_DEFA	chr8	6885553	C	T	24	67,00%	het	16	rs6996890		1,00E-012

reg_DEFA		chr8	6885796	T	G	39	54,00%	het	21			rs4841818			1,00E-012
reg_DEFA		chr8	6886141	A	G	44	50,00%	het	22			rs4358823			1,00E-012
reg_DEFA		chr8	6887746	G	A	51	49,00%	het	25			rs10086568			1,00E-012
reg_DEFA		chr8	6888052	G	A	31	42,00%	het	13			rs13257504			1,00E-012
reg_DEFA		chr8	6888529	G	C	17	65,00%	het	11						1,00E-012
reg_DEFA		chr8	6889029	C	G	31	65,00%	het	20			rs4304345			1,00E-012
reg_DEFA		chr8	6889086	C	T	34	35,00%	het	12						1,59E-011
reg_DEFA		chr8	6889453	G	A	25	56,00%	het	14					rs55690609	1,00E-012
reg_DEFA		chr8	6890057	T	A	50	62,00%	het	31			rs4841822			1,00E-012
reg_DEFA		chr8	6890276	C	T	40	42,00%	het	17			rs4260915			1,00E-012
reg_DEFA		chr8	6890313	C	G	40	50,00%	het	20			rs4260916			1,00E-012
reg_DEFA		chr8	6890558	G	A	32	56,00%	het	18			rs4379464			1,00E-012
reg_DEFA		chr8	6891017	C	A	40	55,00%	het	22			rs12682030			1,00E-012
reg_DEFA		chr8	6891739	T	C	71	59,00%	het	42					rs61187659	1,00E-012
reg_DEFA		chr8	6891919	A	G	64	59,00%	het	38			rs6999181			1,00E-012
reg_DEFA		chr8	6892711	A	G	35	43,00%	het	15			rs13249918			1,00E-012
reg_DEFA		chr8	6893086	T	C	19	37,00%	het	7			rs12544774			1,34E-007
reg_DEFA		chr8	6893641	G	A	25	52,00%	het	13						1,00E-012
reg_DEFA		chr8	6896339	C	G	11	55,00%	het	6			rs4446760			6,19E-008
reg_DEFA		chr8	6897064	A	C	53	51,00%	het	27			rs9644778			1,00E-012
reg_DEFA		chr8	6898012	G	C	38	47,00%	het	18			rs6605583			1,00E-012
reg_DEFA		chr8	6898076	G	A	41	44,00%	het	18			rs4841830			1,00E-012
reg_DEFA		chr8	6898613	T	G	61	36,00%	het	22			rs12680521			1,00E-012
reg_DEFA		chr8	6899982	G	A	50	60,00%	het	30			rs4645580			1,00E-012
reg_DEFA		chr8	6901260	A	G	42	57,00%	het	24	-	DEFA5	rs10095331			1,00E-012
reg_DEFA		chr8	6902056	T	C	61	31,00%	het	19			rs4395911			1,00E-012
reg_DEFA		chr8	6902827	G	C	33	100,00%	homvar	33			rs4415345			1,00E-012
reg_DEFA		chr8	6903072	A	G	52	98,00%	homvar	51			rs6995789			1,00E-012
reg_DEFA		chr8	6903083	C	G	52	100,00%	homvar	52			rs6988319			1,00E-012
reg_DEFA		chr8	6904688	A	G	24	37,00%	het	9			rs41471547	rs4841831		1,72E-009
reg_DEFA		chr8	6905827	C	G	38	53,00%	het	20					rs66643008	1,00E-012
reg_DEFA		chr8	6906215	T	G	13	23,00%	ambig	3						2,93E-003
reg_DEFA		chr8	6907158	G	T	25	48,00%	het	12			rs7834209			1,00E-012
reg_DEFA		chr8	6907549	C	G	72	29,00%	het	21					rs57992355	1,00E-012
reg_DEFA		chr8	6909077	T	G	33	67,00%	het	22			rs4394417			1,00E-012
reg_DEFB	YES	chr8	7157615	A	G	59	10,00%	1of6	6						2,35E-003
reg_DEFB	YES	chr8	7157850	C	G	46	15,00%	1of6	7	+	DEFB109P1B				8,29E-005
reg_DEFB	YES	chr8	7157878	T	G	42	17,00%	1of6	7	+	DEFB109P1B	rs35717569			4,53E-005
reg_DEFB	YES	chr8	7157902	A	T	42	14,00%	1of6	6	+	DEFB109P1B			rs4089933	3,81E-004
reg_DEFB	YES	chr8	7157915	C	A	41	12,00%	1of6	5	+	DEFB109P1B				2,42E-003
reg_DEFB	YES	chr8	7158094	A	G	16	19,00%	1of6	3	+	DEFB109P1B				5,44E-003
reg_DEFB	YES	chr8	7158098	G	A	16	19,00%	1of6	3	+	DEFB109P1B				5,44E-003
reg_DEFB	YES	chr8	7158368	A	G	36	11,00%	1of6	4	+	DEFB109P1B				9,17E-003
reg_DEFB	YES	chr8	7158412	G	A	40	10,00%	1of6	4	+	DEFB109P1B				1,32E-002
reg_DEFB	YES	chr8	7159448	T	A	100	12,00%	1of6	12	+	DEFB109P1B	rs7013998			3,52E-006
reg_DEFB	YES	chr8	7159450	C	G	103	11,00%	1of6	11	+	DEFB109P1B				2,72E-005
reg_DEFB	YES	chr8	7159461	C	T	100	13,00%	1of6	13	+	DEFB109P1B	rs7001891			5,53E-007
reg_DEFB	YES	chr8	7159601	C	T	105	13,00%	1of6	14	+	DEFB109P1B				1,49E-007
reg_DEFB	YES	chr8	7159605	T	G	104	25,00%	1of6	26	+	DEFB109P1B				1,00E-012
reg_DEFB	YES	chr8	7159615	G	A	103	26,00%	2of6	27	+	DEFB109P1B				1,00E-012
reg_DEFB	YES	chr8	7159663	C	T	94	29,00%	2of6	27	+	DEFB109P1B				1,00E-012
reg_DEFB	YES	chr8	7159700	C	G	84	29,00%	2of6	24	+	DEFB109P1B				1,00E-012
reg_DEFB	YES	chr8	7159772	C	T	72	28,00%	2of6	20	+	DEFB109P1B				1,00E-012

reg_DEFB	YES	chr8	7159834	T	A	56	18,00%	1of6	10	+	DEFB109P1B	rs28509470		5,60E-007
reg_DEFB	YES	chr8	7159850	A	T	50	12,00%	1of6	6	+	DEFB109P1B			9,88E-004
reg_DEFB	YES	chr8	7161651	T	C	13	23,00%	1of6	3	+	DEFB109P1B			2,93E-003
reg_DEFB	YES	chr8	7161701	A	G	14	36,00%	2of6	5	+	DEFB109P1B		rs4585725	1,08E-005
reg_DEFB	YES	chr8	7161705	T	C	14	29,00%	2of6	4	+	DEFB109P1B			2,33E-004
reg_DEFB	YES	chr8	7161716	C	G	13	38,00%	2of6	5	+	DEFB109P1B			7,10E-006
reg_DEFB	YES	chr8	7161736	T	C	12	42,00%	2of6	5	+	DEFB109P1B			4,45E-006
reg_DEFB	YES	chr8	7161809	G	A	30	37,00%	2of6	11	+	DEFB109P1B		rs6468887	3,60E-011
reg_DEFB	YES	chr8	7161813	T	C	31	39,00%	2of6	12	+	DEFB109P1B			4,09E-012
reg_DEFB	YES	chr8	7161816	A	G	31	35,00%	2of6	11	+	DEFB109P1B			5,47E-011
reg_DEFB	YES	chr8	7161822	C	T	30	43,00%	3of6	13	+	DEFB109P1B			1,00E-012
reg_DEFB	YES	chr8	7161838	G	A	32	41,00%	2of6	13	+	DEFB109P1B		rs6470342	1,00E-012
reg_DEFB	YES	chr8	7161843	A	G	33	48,00%	3of6	16	+	DEFB109P1B			1,00E-012
reg_DEFB	YES	chr8	7161862	C	G	37	32,00%	2of6	12	+	DEFB109P1B		rs71526779	2,51E-011
reg_DEFB	YES	chr8	7161864	T	C	38	39,00%	2of6	15	+	DEFB109P1B		rs35703273	1,00E-012
reg_DEFB	YES	chr8	7161903	A	T	46	33,00%	2of6	15	+	DEFB109P1B			1,00E-012
reg_DEFB	YES	chr8	7161905	C	T	47	32,00%	2of6	15	+	DEFB109P1B			1,00E-012
reg_DEFB	YES	chr8	7161942	T	G	48	31,00%	2of6	15	+	DEFB109P1B			1,00E-012
reg_DEFB	YES	chr8	7161986	C	T	56	11,00%	1of6	6	+	DEFB109P1B			1,80E-003
reg_DEFB	YES	chr8	7162017	G	A	60	15,00%	1of6	9	+	DEFB109P1B			9,22E-006
reg_DEFB	YES	chr8	7162060	T	G	64	20,00%	1of6	13	+	DEFB109P1B			2,21E-009
reg_DEFB	YES	chr8	7162065	T	G	65	22,00%	1of6	14	+	DEFB109P1B			2,37E-010
reg_DEFB	YES	chr8	7162126	A	G	71	18,00%	1of6	13	+	DEFB109P1B			8,42E-009
reg_DEFB	YES	chr8	7162150	T	C	73	18,00%	1of6	13	+	DEFB109P1B			1,20E-008
reg_DEFB	YES	chr8	7162179	C	G	75	16,00%	1of6	12	+	DEFB109P1B			1,49E-007
reg_DEFB	YES	chr8	7162263	T	C	88	19,00%	1of6	17	+	DEFB109P1B			2,22E-011
reg_DEFB	YES	chr8	7162291	A	C	86	15,00%	1of6	13	+	DEFB109P1B			9,13E-008
reg_DEFB	YES	chr8	7162308	A	C	88	12,00%	1of6	11	+	DEFB109P1B			5,97E-006
reg_DEFB	YES	chr8	7162321	A	G	90	10,00%	1of6	9	+	DEFB109P1B			2,37E-004
reg_DEFB	YES	chr8	7162338	T	C	88	14,00%	1of6	12	+	DEFB109P1B			8,89E-007
reg_DEFB	YES	chr8	7162365	G	A	83	17,00%	1of6	14	+	DEFB109P1B			6,90E-009
reg_DEFB	YES	chr8	7162410	A	G	85	15,00%	1of6	13	+	DEFB109P1B			7,92E-008
reg_DEFB	YES	chr8	7162442	G	C	86	17,00%	1of6	15	+	DEFB109P1B			1,24E-009
reg_DEFB	YES	chr8	7162550	A	C	104	12,00%	1of6	12	+	DEFB109P1B			5,33E-006
reg_DEFB	YES	chr8	7162860	G	T	87	16,00%	1of6	14	+	DEFB109P1B			1,29E-008
reg_DEFB	YES	chr8	7162861	A	T	86	16,00%	1of6	14	+	DEFB109P1B			1,11E-008
reg_DEFB	YES	chr8	7162865	C	G	85	20,00%	1of6	17	+	DEFB109P1B	rs34406851		1,22E-011
reg_DEFB	YES	chr8	7162927	C	T	91	11,00%	1of6	10	+	DEFB109P1B			4,87E-005
reg_DEFB	YES	chr8	7162966	C	G	101	33,00%	2of6	33	+	DEFB109P1B			1,00E-012
reg_DEFB	YES	chr8	7163030	A	T	120	12,00%	1of6	14	+	DEFB109P1B			7,88E-007
reg_DEFB	YES	chr8	7163038	C	T	121	11,00%	1of6	13	+	DEFB109P1B			4,88E-006
reg_DEFB	YES	chr8	7163075	G	A	128	41,00%	2of6	52	+	DEFB109P1B			1,00E-012
reg_DEFB	YES	chr8	7163106	C	T	127	32,00%	2of6	41	+	DEFB109P1B			1,00E-012
reg_DEFB	YES	chr8	7163115	G	C	126	36,00%	2of6	45	+	DEFB109P1B			1,00E-012
reg_DEFB	YES	chr8	7163135	A	T	130	14,00%	1of6	18	+	DEFB109P1B			1,43E-009
reg_DEFB	YES	chr8	7163183	G	A	123	15,00%	1of6	18	+	DEFB109P1B			5,71E-010
reg_DEFB	YES	chr8	7163229	A	C	121	13,00%	1of6	16	+	DEFB109P1B			2,23E-008
reg_DEFB	YES	chr8	7163236	C	T	120	32,00%	2of6	38	+	DEFB109P1B			1,00E-012
reg_DEFB	YES	chr8	7163242	C	T	120	47,00%	3of6	56	+	DEFB109P1B			1,00E-012
reg_DEFB	YES	chr8	7163246	C	A	118	47,00%	3of6	55	+	DEFB109P1B			1,00E-012
reg_DEFB	YES	chr8	7163250	A	G	120	12,00%	1of6	14	+	DEFB109P1B			7,88E-007
reg_DEFB	YES	chr8	7163295	G	A	112	45,00%	3of6	50	+	DEFB109P1B			1,00E-012
reg_DEFB	YES	chr8	7163341	G	C	113	40,00%	2of6	45	+	DEFB109P1B			1,00E-012

reg_DEFB	YES	chr8	7163423	C	T	94	20,00%	1of6	19	+	DEFB109P1B	rs3988781	1,00E-012
reg_DEFB	YES	chr8	7163430	T	C	98	29,00%	2of6	28	+	DEFB109P1B		1,00E-012
reg_DEFB	YES	chr8	7163923	A	G	64	11,00%	1of6	7	+	DEFB109P1B		6,71E-004
reg_DEFB	YES	chr8	7164584	C	T	58	12,00%	1of6	7	+	DEFB109P1B	rs2947426	3,66E-004
reg_DEFB	YES	chr8	7164586	T	C	58	17,00%	1of6	10	+	DEFB109P1B	rs2954046	7,87E-007
reg_DEFB	YES	chr8	7164674	T	G	52	12,00%	1of6	6	+	DEFB109P1B		1,22E-003
reg_DEFB	YES	chr8	7164874	C	G	61	21,00%	1of6	13	+	DEFB109P1B		1,19E-009
reg_DEFB	YES	chr8	7164890	A	G	65	23,00%	1of6	15				2,10E-011
reg_DEFB	YES	chr8	7164975	C	T	79	23,00%	1of6	18			rs11786798	1,00E-012
reg_DEFB	YES	chr8	7164976	G	A	80	11,00%	1of6	9			rs11783035	9,57E-005
reg_DEFB	YES	chr8	7165027	C	T	84	32,00%	2of6	27				1,00E-012
reg_DEFB	YES	chr8	7165051	A	G	83	19,00%	1of6	16				7,48E-011
reg_DEFB	YES	chr8	7165061	T	G	86	34,00%	2of6	29				1,00E-012
reg_DEFB	YES	chr8	7165115	G	A	83	25,00%	1of6	21				1,00E-012
reg_DEFB	YES	chr8	7165121	T	C	84	15,00%	1of6	13				6,86E-008
reg_DEFB	YES	chr8	7165164	T	A	89	34,00%	2of6	30				1,00E-012
reg_DEFB	YES	chr8	7165167	G	C	90	13,00%	1of6	12				1,14E-006
reg_DEFB	YES	chr8	7165176	G	A	86	20,00%	1of6	17				1,50E-011
reg_DEFB	YES	chr8	7165207	A	T	82	32,00%	2of6	26				1,00E-012
reg_DEFB	YES	chr8	7165621	T	A	26	12,00%	1of6	3				2,13E-002
reg_DEFB	YES	chr8	7165634	C	T	25	12,00%	1of6	3				1,92E-002
reg_DEFB	YES	chr8	7165741	G	A	25	12,00%	1of6	3				1,92E-002
reg_DEFB	YES	chr8	7165766	C	A	27	19,00%	1of6	5			rs3988809	3,40E-004
reg_DEFB	YES	chr8	7165798	C	T	27	19,00%	1of6	5				3,40E-004
reg_DEFB	YES	chr8	7165812	G	A	27	22,00%	1of6	6				2,89E-005
reg_DEFB	YES	chr8	7165831	T	G	27	15,00%	1of6	4				3,22E-003
reg_DEFB	YES	chr8	7167310	A	T	24	29,00%	2of6	7				8,35E-007
reg_DEFB	YES	chr8	7167370	T	G	28	36,00%	2of6	10			rs7835547	3,84E-010
reg_DEFB	YES	chr8	7167475	T	C	41	46,00%	3of6	19				1,00E-012
reg_DEFB	YES	chr8	7167493	A	G	43	42,00%	2of6	18				1,00E-012
reg_DEFB	YES	chr8	7167515	A	C	43	40,00%	2of6	17				1,00E-012
reg_DEFB	YES	chr8	7167603	C	A	40	10,00%	1of6	4				1,32E-002
reg_DEFB	YES	chr8	7167647	A	G	40	27,00%	2of6	11			rs55915041	1,19E-009
reg_DEFB	YES	chr8	7167691	G	A	44	18,00%	1of6	8			rs72680848	6,62E-006
reg_DEFB	YES	chr8	7167694	T	G	44	11,00%	1of6	5				3,31E-003
reg_DEFB	YES	chr8	7167705	C	T	46	22,00%	1of6	10			rs62492162	7,91E-008
reg_DEFB	YES	chr8	7167774	T	A	51	16,00%	1of6	8				2,06E-005
reg_DEFB	YES	chr8	7167808	G	T	57	14,00%	1of6	8				4,73E-005
reg_DEFB	YES	chr8	7167817	T	C	63	35,00%	2of6	22				1,00E-012
reg_DEFB	YES	chr8	7167844	A	T	74	16,00%	1of6	12				1,28E-007
reg_DEFB	YES	chr8	7167864	A	G	78	32,00%	2of6	25				1,00E-012
reg_DEFB	YES	chr8	7167885	C	A	79	15,00%	1of6	12				2,68E-007
reg_DEFB	YES	chr8	7167894	T	C	85	31,00%	2of6	26				1,00E-012
reg_DEFB	YES	chr8	7167922	A	C	82	24,00%	1of6	20				1,00E-012
reg_DEFB	YES	chr8	7167944	C	A	89	15,00%	1of6	13			rs71520419	1,38E-007
reg_DEFB	YES	chr8	7167957	G	C	92	29,00%	2of6	27				1,00E-012
reg_DEFB	YES	chr8	7167983	G	A	93	25,00%	1of6	23				1,00E-012
reg_DEFB	YES	chr8	7167987	T	C	94	24,00%	1of6	23				1,00E-012
reg_DEFB	YES	chr8	7168028	C	T	98	15,00%	1of6	15				8,04E-009
reg_DEFB	YES	chr8	7168044	C	G	99	20,00%	1of6	20				1,00E-012
reg_DEFB	YES	chr8	7168073	T	G	95	17,00%	1of6	16				6,05E-010
reg_DEFB	YES	chr8	7168178	T	C	73	25,00%	1of6	18				1,00E-012
reg_DEFB	YES	chr8	7168187	G	A	72	15,00%	1of6	11				7,90E-007

reg_DEFB	YES	chr8	7168243	T	G	59	20,00%	1of6	12				8,98E-009
reg_DEFB	YES	chr8	7168264	C	T	55	15,00%	1of6	8				3,63E-005
reg_DEFB	YES	chr8	7168306	G	T	46	11,00%	1of6	5	rs35981343			4,03E-003
reg_DEFB	YES	chr8	7168425	C	T	66	14,00%	1of6	9				2,04E-005
reg_DEFB	YES	chr8	7168451	G	C	69	20,00%	1of6	14			rs11777540	5,52E-010
reg_DEFB	YES	chr8	7168457	T	C	69	41,00%	2of6	28				1,00E-012
reg_DEFB	YES	chr8	7168499	G	A	73	47,00%	3of6	34				1,00E-012
reg_DEFB	YES	chr8	7168558	C	G	88	19,00%	1of6	17				2,22E-011
reg_DEFB	YES	chr8	7168569	C	T	89	20,00%	1of6	18				6,89E-012
reg_DEFB	YES	chr8	7168632	A	G	100	53,00%	3of6	53				1,00E-012
reg_DEFB	YES	chr8	7168694	T	C	114	23,00%	1of6	26				1,00E-012
reg_DEFB	YES	chr8	7168697	T	C	114	59,00%	4of6	67				1,00E-012
reg_DEFB	YES	chr8	7168738	C	T	110	59,00%	4of6	65				1,00E-012
reg_DEFB	YES	chr8	7168755	G	A	114	60,00%	4of6	68				1,00E-012
reg_DEFB	YES	chr8	7168766	C	A	118	61,00%	4of6	72			rs11786479	1,00E-012
reg_DEFB	YES	chr8	7168779	T	C	120	24,00%	1of6	29				1,00E-012
reg_DEFB	YES	chr8	7168781	G	A	117	15,00%	1of6	18				2,54E-010
reg_DEFB	YES	chr8	7168791	A	C	116	59,00%	4of6	68				1,00E-012
reg_DEFB	YES	chr8	7168807	T	C	114	35,00%	2of6	40			rs11776779	1,00E-012
reg_DEFB	YES	chr8	7168824	A	G	108	20,00%	1of6	22				1,00E-012
reg_DEFB	YES	chr8	7168834	C	T	108	33,00%	2of6	36			rs11774389	1,00E-012
reg_DEFB	YES	chr8	7168916	T	C	92	12,00%	1of6	11				9,22E-006
reg_DEFB	YES	chr8	7168926	T	C	88	57,00%	3of6	50	rs3178039	rs34791945		1,00E-012
reg_DEFB	YES	chr8	7169013	C	A	79	19,00%	1of6	15				3,64E-010
reg_DEFB	YES	chr8	7169050	T	G	74	36,00%	2of6	27			rs71509292	1,00E-012
reg_DEFB	YES	chr8	7169077	C	A	66	33,00%	2of6	22			rs10089913	1,00E-012
reg_DEFB	YES	chr8	7169103	G	C	57	14,00%	1of6	8				4,73E-005
reg_DEFB	YES	chr8	7169107	T	C	57	14,00%	1of6	8				4,73E-005
reg_DEFB	YES	chr8	7169595	A	G	44	43,00%	3of6	19	rs2078494			1,00E-012
reg_DEFB	YES	chr8	7169868	G	A	62	13,00%	1of6	8				8,75E-005
reg_DEFB	YES	chr8	7169921	T	A	82	26,00%	2of6	21	rs3907269			1,00E-012
reg_DEFB	YES	chr8	7169973	C	T	91	25,00%	1of6	23			rs4109371	1,00E-012
reg_DEFB	YES	chr8	7169980	C	T	91	13,00%	1of6	12				1,28E-006
reg_DEFB	YES	chr8	7169982	C	T	91	13,00%	1of6	12				1,28E-006
reg_DEFB	YES	chr8	7169999	C	T	94	16,00%	1of6	15				4,47E-009
reg_DEFB	YES	chr8	7170000	G	A	96	25,00%	1of6	24				1,00E-012
reg_DEFB	YES	chr8	7170022	T	C	98	45,00%	3of6	44			rs4096457	1,00E-012
reg_DEFB	YES	chr8	7170056	C	G	107	44,00%	3of6	47	rs34518748			1,00E-012
reg_DEFB	YES	chr8	7170064	C	T	111	28,00%	2of6	31			rs4096458	1,00E-012
reg_DEFB	YES	chr8	7170105	G	A	122	30,00%	2of6	37	rs3907268			1,00E-012
reg_DEFB	YES	chr8	7170112	C	A	120	18,00%	1of6	22			rs62488805	1,00E-012
reg_DEFB	YES	chr8	7170135	A	G	121	26,00%	2of6	31			rs2739990	1,00E-012
reg_DEFB	YES	chr8	7170213	T	C	130	35,00%	2of6	46				1,00E-012
reg_DEFB	YES	chr8	7170282	G	A	128	62,00%	4of6	79	rs34361581			1,00E-012
reg_DEFB	YES	chr8	7170290	C	T	129	56,00%	3of6	72				1,00E-012
reg_DEFB	YES	chr8	7170304	C	T	126	30,00%	2of6	38				1,00E-012
reg_DEFB	YES	chr8	7170312	C	T	124	29,00%	2of6	36				1,00E-012
reg_DEFB	YES	chr8	7170317	C	T	125	24,00%	1of6	30				1,00E-012
reg_DEFB	YES	chr8	7170331	G	T	123	55,00%	3of6	68				1,00E-012
reg_DEFB	YES	chr8	7170340	G	A	123	22,00%	1of6	27				1,00E-012
reg_DEFB	YES	chr8	7170351	G	T	122	58,00%	3of6	71				1,00E-012
reg_DEFB	YES	chr8	7170384	T	A	128	30,00%	2of6	38				1,00E-012
reg_DEFB	YES	chr8	7170412	G	A	135	23,00%	1of6	31				1,00E-012

reg_DEFB	YES	chr8	7170443	G	C	129	31,00%	2of6	40			1,00E-012
reg_DEFB	YES	chr8	7170453	T	C	126	30,00%	2of6	38			1,00E-012
reg_DEFB	YES	chr8	7170458	C	A	126	21,00%	1of6	26			1,00E-012
reg_DEFB	YES	chr8	7170468	T	A	127	51,00%	3of6	65			1,00E-012
reg_DEFB	YES	chr8	7170492	T	A	128	17,00%	1of6	22			1,00E-012
reg_DEFB	YES	chr8	7170518	A	T	112	15,00%	1of6	17			9,52E-010
reg_DEFB	YES	chr8	7170538	G	A	109	15,00%	1of6	16			4,82E-009
reg_DEFB	YES	chr8	7170546	T	A	107	12,00%	1of6	13			1,21E-006
reg_DEFB	YES	chr8	7170547	A	G	106	34,00%	2of6	36			1,00E-012
reg_DEFB	YES	chr8	7170569	T	A	99	32,00%	2of6	32			1,00E-012
reg_DEFB	YES	chr8	7170573	A	G	96	43,00%	3of6	41		rs71505181	1,00E-012
reg_DEFB	YES	chr8	7170645	T	C	80	36,00%	2of6	29			1,00E-012
reg_DEFB	YES	chr8	7170771	T	C	48	17,00%	1of6	8			1,30E-005
reg_DEFB	YES	chr8	7171489	G	A	19	16,00%	1of6	3			8,95E-003
reg_DEFB	YES	chr8	7171497	G	C	19	16,00%	1of6	3			8,95E-003
reg_DEFB	YES	chr8	7171581	G	A	20	15,00%	1of6	3			1,03E-002
reg_DEFB	YES	chr8	7171635	G	A	17	24,00%	1of6	4		rs59164418	5,24E-004
reg_DEFB	YES	chr8	7173464	A	G	76	12,00%	1of6	9			6,39E-005
reg_DEFB	YES	chr8	7173475	A	G	78	10,00%	1of6	8			4,39E-004
reg_DEFB	YES	chr8	7173529	T	G	82	16,00%	1of6	13		rs3988909	5,10E-008
reg_DEFB	YES	chr8	7173542	C	T	83	19,00%	1of6	16		rs3988908	7,48E-011
reg_DEFB	YES	chr8	7173577	A	C	90	21,00%	1of6	19			1,00E-012
reg_DEFB	YES	chr8	7173587	T	G	86	24,00%	1of6	21	rs34181137		1,00E-012
reg_DEFB	YES	chr8	7173717	C	T	92	25,00%	1of6	23			1,00E-012
reg_DEFB	YES	chr8	7173721	A	G	94	28,00%	2of6	26			1,00E-012
reg_DEFB	YES	chr8	7173725	C	T	93	26,00%	2of6	24			1,00E-012
reg_DEFB	YES	chr8	7173741	A	G	94	29,00%	2of6	27			1,00E-012
reg_DEFB	YES	chr8	7173771	T	G	97	29,00%	2of6	28			1,00E-012
reg_DEFB	YES	chr8	7173875	T	G	99	30,00%	2of6	30			1,00E-012
reg_DEFB	YES	chr8	7173894	T	C	98	27,00%	2of6	26		rs3976517	1,00E-012
reg_DEFB	YES	chr8	7173897	T	G	97	28,00%	2of6	27			1,00E-012
reg_DEFB	YES	chr8	7173926	A	G	91	29,00%	2of6	26		rs2864406	1,00E-012
reg_DEFB	YES	chr8	7173931	G	C	88	30,00%	2of6	26			1,00E-012
reg_DEFB	YES	chr8	7173970	G	T	87	24,00%	1of6	21			1,00E-012
reg_DEFB	YES	chr8	7173976	G	T	88	25,00%	1of6	22			1,00E-012
reg_DEFB	YES	chr8	7173991	A	G	86	20,00%	1of6	17			1,50E-011
reg_DEFB	YES	chr8	7174003	T	C	86	16,00%	1of6	14			1,11E-008
reg_DEFB	YES	chr8	7174006	T	C	86	19,00%	1of6	16		rs2864408	1,31E-010
reg_DEFB	YES	chr8	7174013	C	T	86	19,00%	1of6	16			1,31E-010
reg_DEFB	YES	chr8	7174047	C	T	89	99,00%	6of6	88		rs62488807	1,00E-012
reg_DEFB	YES	chr8	7174052	C	T	87	14,00%	1of6	12			7,84E-007
reg_DEFB	YES	chr8	7174097	C	T	94	13,00%	1of6	12			1,82E-006
reg_DEFB	YES	chr8	7174282	C	G	101	15,00%	1of6	15			1,23E-008
reg_DEFB	YES	chr8	7174316	G	T	103	17,00%	1of6	18			3,44E-011
reg_DEFB	YES	chr8	7174321	A	G	103	17,00%	1of6	18			3,44E-011
reg_DEFB	YES	chr8	7174355	T	A	98	22,00%	1of6	22		rs66483251	1,00E-012
reg_DEFB	YES	chr8	7174375	A	C	96	21,00%	1of6	20		rs13260764	1,00E-012
reg_DEFB	YES	chr8	7174387	T	A	97	23,00%	1of6	22			1,00E-012
reg_DEFB	YES	chr8	7174394	A	C	97	23,00%	1of6	22			1,00E-012
reg_DEFB	YES	chr8	7174405	A	T	97	15,00%	1of6	15			6,96E-009
reg_DEFB	YES	chr8	7174432	T	C	104	25,00%	1of6	26			1,00E-012
reg_DEFB	YES	chr8	7174433	A	C	105	17,00%	1of6	18			4,84E-011
reg_DEFB	YES	chr8	7174459	A	T	109	25,00%	1of6	27			1,00E-012

reg_DEFB	YES	chr8	7174469	T	C	108	24,00%	1of6	26			1,00E-012
reg_DEFB	YES	chr8	7174509	G	A	108	25,00%	1of6	27			1,00E-012
reg_DEFB	YES	chr8	7174523	A	T	108	24,00%	1of6	26			1,00E-012
reg_DEFB	YES	chr8	7174577	C	A	100	12,00%	1of6	12			3,52E-006
reg_DEFB	YES	chr8	7174608	T	C	97	27,00%	2of6	26			1,00E-012
reg_DEFB	YES	chr8	7174659	A	T	81	26,00%	2of6	21			1,00E-012
reg_DEFB	YES	chr8	7174725	A	G	72	18,00%	1of6	13			1,01E-008
reg_DEFB	YES	chr8	7174734	G	A	73	16,00%	1of6	12			1,09E-007
reg_DEFB	YES	chr8	7174748	C	A	70	17,00%	1of6	12			6,75E-008
reg_DEFB	YES	chr8	7174758	G	A	69	17,00%	1of6	12			5,71E-008
reg_DEFB	YES	chr8	7174795	G	A	63	14,00%	1of6	9			1,39E-005
reg_DEFB	YES	chr8	7174818	G	C	65	15,00%	1of6	10			2,33E-006
reg_DEFB	YES	chr8	7174819	A	C	65	15,00%	1of6	10			2,33E-006
reg_DEFB	YES	chr8	7174861	A	C	57	12,00%	1of6	7			3,29E-004
reg_DEFB	YES	chr8	7174867	G	T	57	11,00%	1of6	6			1,97E-003
reg_DEFB	YES	chr8	7175796	C	T	30	10,00%	1of6	3			3,11E-002
reg_DEFB	YES	chr8	7176191	A	C	31	13,00%	1of6	4			5,36E-003
reg_DEFB	YES	chr8	7176233	G	T	30	10,00%	1of6	3			3,11E-002
reg_DEFB	YES	chr8	7176255	A	C	27	15,00%	1of6	4			3,22E-003
reg_DEFB	YES	chr8	7176523	A	G	39	10,00%	1of6	4			1,21E-002
reg_DEFB	YES	chr8	7176768	C	T	28	39,00%	2of6	11	rs9692809		2,64E-011
reg_DEFB	YES	chr8	7177146	T	G	20	15,00%	1of6	3	rs35413820		1,03E-002
reg_DEFB	YES	chr8	7177201	C	A	20	20,00%	1of6	4			1,01E-003
reg_DEFB	YES	chr8	7177210	A	C	18	28,00%	2of6	5			4,29E-005
reg_DEFB	YES	chr8	7177261	G	A	15	20,00%	1of6	3			4,50E-003
reg_DEFB	YES	chr8	7177266	G	T	15	33,00%	2of6	5			1,59E-005
reg_DEFB	YES	chr8	7177287	A	C	14	29,00%	2of6	4			2,33E-004
reg_DEFB	YES	chr8	7177296	T	C	14	21,00%	1of6	3			3,66E-003
reg_DEFB	YES	chr8	7177310	G	C	14	29,00%	2of6	4			2,33E-004
reg_DEFB	YES	chr8	7177323	G	A	13	38,00%	2of6	5			7,10E-006
reg_DEFB	YES	chr8	7177430	C	T	7	57,00%	3of6	4			9,26E-006
reg_DEFB	YES	chr8	7177442	C	T	7	43,00%	3of6	3			3,97E-004
reg_DEFB	YES	chr8	7177462	T	C	7	71,00%	4of6	5			1,30E-007
reg_DEFB	YES	chr8	7178422	T	C	30	20,00%	1of6	6			5,46E-005
reg_DEFB	YES	chr8	7178500	C	T	27	30,00%	2of6	8			1,18E-007
reg_DEFB	YES	chr8	7178506	T	A	28	32,00%	2of6	9			8,38E-009
reg_DEFB	YES	chr8	7178529	A	C	30	33,00%	2of6	10		rs9694780	8,17E-010
reg_DEFB	YES	chr8	7178569	A	C	35	43,00%	3of6	15			1,00E-012
reg_DEFB	YES	chr8	7178638	G	T	41	56,00%	3of6	23	rs34108245		1,00E-012
reg_DEFB	YES	chr8	7178675	G	A	43	53,00%	3of6	23			1,00E-012
reg_DEFB	YES	chr8	7178702	A	G	44	57,00%	3of6	25			1,00E-012
reg_DEFB	YES	chr8	7178706	C	T	44	57,00%	3of6	25			1,00E-012
reg_DEFB	YES	chr8	7178746	A	G	48	56,00%	3of6	27			1,00E-012
reg_DEFB	YES	chr8	7178799	G	C	43	53,00%	3of6	23		rs9694759	1,00E-012
reg_DEFB	YES	chr8	7178804	G	C	42	64,00%	4of6	27			1,00E-012
reg_DEFB	YES	chr8	7178839	T	C	34	53,00%	3of6	18			1,00E-012
reg_DEFB	YES	chr8	7178844	A	G	34	53,00%	3of6	18			1,00E-012
reg_DEFB	YES	chr8	7178852	A	G	34	53,00%	3of6	18			1,00E-012
reg_DEFB	YES	chr8	7178861	G	T	34	53,00%	3of6	18			1,00E-012
reg_DEFB	YES	chr8	7178925	G	A	29	34,00%	2of6	10	rs34767109		5,76E-010
reg_DEFB	YES	chr8	7178949	G	A	28	25,00%	1of6	7			2,63E-006
reg_DEFB	YES	chr8	7178957	A	G	31	23,00%	1of6	7			5,51E-006
reg_DEFB	YES	chr8	7179103	G	A	20	15,00%	1of6	3	rs35175639		1,03E-002

reg_DEFB	YES	chr8	7179104	T	C	20	15,00%	1of6	3	rs35175639		1,03E-002
reg_DEFB	YES	chr8	7179121	A	G	19	16,00%	1of6	3			8,95E-003
reg_DEFB	YES	chr8	7179124	C	T	18	17,00%	1of6	3			7,66E-003
reg_DEFB	YES	chr8	7179183	T	A	27	26,00%	2of6	7			2,02E-006
reg_DEFB	YES	chr8	7179199	G	A	28	29,00%	2of6	8			1,61E-007
reg_DEFB	YES	chr8	7179213	C	G	29	34,00%	2of6	10			5,76E-010
reg_DEFB	YES	chr8	7179238	T	C	33	27,00%	2of6	9			4,21E-008
reg_DEFB	YES	chr8	7179263	T	A	32	31,00%	2of6	10			1,68E-009
reg_DEFB	YES	chr8	7179284	C	G	31	35,00%	2of6	11			5,47E-011
reg_DEFB	YES	chr8	7179324	G	T	32	34,00%	2of6	11			8,20E-011
reg_DEFB	YES	chr8	7179330	C	T	33	33,00%	2of6	11			1,21E-010
reg_DEFB	YES	chr8	7179332	G	A	33	33,00%	2of6	11			1,21E-010
reg_DEFB	YES	chr8	7179357	C	G	34	32,00%	2of6	11			1,76E-010
reg_DEFB	YES	chr8	7179410	A	G	33	30,00%	2of6	10			2,37E-009
reg_DEFB	YES	chr8	7179416	T	G	33	27,00%	2of6	9			4,21E-008
reg_DEFB	YES	chr8	7179418	G	A	34	26,00%	2of6	9			5,61E-008
reg_DEFB	YES	chr8	7179439	A	C	30	30,00%	2of6	9			1,66E-008
reg_DEFB	YES	chr8	7179443	T	G	30	27,00%	2of6	8			2,91E-007
reg_DEFB	YES	chr8	7179461	T	C	26	15,00%	1of6	4			2,79E-003
reg_DEFB	YES	chr8	7179467	T	C	26	15,00%	1of6	4			2,79E-003
reg_DEFB	YES	chr8	7179473	G	T	26	19,00%	1of6	5			2,83E-004
reg_DEFB	YES	chr8	7179531	A	G	27	15,00%	1of6	4			3,22E-003
reg_DEFB	YES	chr8	7179764	A	G	16	19,00%	1of6	3			5,44E-003
reg_DEFB	YES	chr8	7180330	C	G	18	28,00%	2of6	5			4,29E-005
reg_DEFB	YES	chr8	7180392	T	C	21	38,00%	2of6	8			1,22E-008
reg_DEFB	YES	chr8	7180417	G	T	23	35,00%	2of6	8	rs34030924		2,82E-008
reg_DEFB	YES	chr8	7180486	T	C	25	40,00%	2of6	10			1,01E-010
reg_DEFB	YES	chr8	7180501	T	C	26	35,00%	2of6	9			3,95E-009
reg_DEFB	YES	chr8	7180513	T	G	26	42,00%	2of6	11			9,51E-012
reg_DEFB	YES	chr8	7180548	T	C	26	35,00%	2of6	9			3,95E-009
reg_DEFB	YES	chr8	7180550	G	C	26	35,00%	2of6	9			3,95E-009
reg_DEFB	YES	chr8	7180560	G	C	27	37,00%	2of6	10			2,51E-010
reg_DEFB	YES	chr8	7180565	T	G	26	38,00%	2of6	10			1,61E-010
reg_DEFB	YES	chr8	7180582	G	T	29	34,00%	2of6	10			5,76E-010
reg_DEFB	YES	chr8	7180595	G	C	31	32,00%	2of6	10			1,18E-009
reg_DEFB	YES	chr8	7180619	T	G	36	28,00%	2of6	10		rs71509297	6,11E-009
reg_DEFB	YES	chr8	7180635	T	G	37	22,00%	1of6	8		rs60601573	1,66E-006
reg_DEFB	YES	chr8	7180647	G	A	41	12,00%	1of6	5			2,42E-003
reg_DEFB	YES	chr8	7180674	A	G	45	24,00%	1of6	11		rs2698948	4,70E-009
reg_DEFB	YES	chr8	7180691	G	C	49	22,00%	1of6	11			1,24E-008
reg_DEFB	YES	chr8	7180700	A	C	51	18,00%	1of6	9			2,29E-006
reg_DEFB	YES	chr8	7180705	A	C	52	23,00%	1of6	12			1,94E-009
reg_DEFB	YES	chr8	7180711	A	G	52	17,00%	1of6	9			2,71E-006
reg_DEFB	YES	chr8	7180719	C	A	51	14,00%	1of6	7		rs55731049	1,62E-004
reg_DEFB	YES	chr8	7180749	T	G	47	11,00%	1of6	5			4,42E-003
reg_DEFB	YES	chr8	7180777	C	T	46	11,00%	1of6	5			4,03E-003
reg_DEFB	YES	chr8	7180779	G	T	45	24,00%	1of6	11			4,70E-009
reg_DEFB	YES	chr8	7181103	C	T	33	61,00%	4of6	20			1,00E-012
reg_DEFB	YES	chr8	7181236	G	A	34	65,00%	4of6	22	rs35740946		1,00E-012
reg_DEFB	YES	chr8	7181244	T	A	34	62,00%	4of6	21		rs11250190	1,00E-012
reg_DEFB	YES	chr8	7181549	A	G	29	14,00%	1of6	4			4,20E-003
reg_DEFB	YES	chr8	7181652	A	G	23	17,00%	1of6	4			1,75E-003
reg_DEFB	YES	chr8	7181735	T	C	20	15,00%	1of6	3			1,03E-002

reg_DEFB	YES	chr8	7181741	C	T	18	17,00%	1of6	3		7,66E-003
reg_DEFB	YES	chr8	7181748	C	G	17	18,00%	1of6	3		6,50E-003
reg_DEFB	YES	chr8	7184304	G	A	35	11,00%	1of6	4		8,30E-003
reg_DEFB	YES	chr8	7184320	G	A	37	11,00%	1of6	4		1,01E-002
reg_DEFB	YES	chr8	7184344	T	G	36	11,00%	1of6	4		9,17E-003
reg_DEFB	YES	chr8	7184348	C	T	34	15,00%	1of6	5		1,03E-003
reg_DEFB	YES	chr8	7184353	A	C	34	12,00%	1of6	4		7,48E-003
reg_DEFB	YES	chr8	7184391	T	A	32	12,00%	1of6	4		6,02E-003
reg_DEFB	YES	chr8	7184454	A	G	28	14,00%	1of6	4		3,68E-003
reg_DEFB	YES	chr8	7184493	T	C	29	21,00%	1of6	6	rs55780142	4,46E-005
reg_DEFB	YES	chr8	7184501	A	G	29	21,00%	1of6	6		4,46E-005
reg_DEFB	YES	chr8	7184535	C	T	25	20,00%	1of6	5		2,33E-004
reg_DEFB	YES	chr8	7184621	T	C	19	42,00%	2of6	8		4,72E-009
reg_DEFB	YES	chr8	7184657	G	A	18	17,00%	1of6	3		7,66E-003
reg_DEFB	YES	chr8	7184672	T	G	18	17,00%	1of6	3		7,66E-003
reg_DEFB	YES	chr8	7184731	A	C	17	18,00%	1of6	3		6,50E-003
reg_DEFB	YES	chr8	7184756	G	C	21	29,00%	2of6	6		5,96E-006
reg_DEFB	YES	chr8	7184796	T	C	19	37,00%	2of6	7		1,34E-007
reg_DEFB	YES	chr8	7184831	T	C	18	39,00%	2of6	7		8,67E-008
reg_DEFB	YES	chr8	7184873	G	C	20	40,00%	2of6	8		7,71E-009
reg_DEFB	YES	chr8	7184892	A	G	23	35,00%	2of6	8		2,82E-008
reg_DEFB	YES	chr8	7184916	C	G	22	32,00%	2of6	7	rs28393679	4,28E-007
reg_DEFB	YES	chr8	7184967	C	T	22	27,00%	2of6	6		8,04E-006
reg_DEFB	YES	chr8	7184970	G	C	23	30,00%	2of6	7		6,03E-007
reg_DEFB	YES	chr8	7184985	C	T	24	25,00%	1of6	6		1,39E-005
reg_DEFB	YES	chr8	7185097	A	G	31	32,00%	2of6	10	rs72496220	1,18E-009
reg_DEFB	YES	chr8	7185117	C	T	31	32,00%	2of6	10	rs60248625	1,18E-009
reg_DEFB	YES	chr8	7185138	G	T	31	26,00%	2of6	8	rs61044488	3,85E-007
reg_DEFB	YES	chr8	7185210	C	T	31	23,00%	1of6	7	rs13256402	5,51E-006
reg_DEFB	YES	chr8	7185237	G	C	32	34,00%	2of6	11		8,20E-011
reg_DEFB	YES	chr8	7185241	A	G	32	19,00%	1of6	6	rs13252616	8,02E-005
reg_DEFB	YES	chr8	7185256	G	A	32	16,00%	1of6	5		7,72E-004
reg_DEFB	YES	chr8	7185288	G	A	31	16,00%	1of6	5		6,64E-004
reg_DEFB	YES	chr8	7186019	C	T	43	37,00%	2of6	16		1,00E-012
reg_DEFB	YES	chr8	7186692	A	G	42	100,00%	6of6	42	rs71507397	1,00E-012
reg_DEFB	YES	chr8	7186729	A	C	40	12,00%	1of6	5		2,17E-003
reg_DEFB	YES	chr8	7186745	T	C	43	14,00%	1of6	6		4,35E-004
reg_DEFB	YES	chr8	7186753	G	T	46	17,00%	1of6	8	rs62636811	9,36E-006
reg_DEFB	YES	chr8	7186832	A	C	46	13,00%	1of6	6	rs62636813	6,30E-004
reg_DEFB	YES	chr8	7187793	G	T	30	10,00%	1of6	3		3,11E-002
reg_DEFB	YES	chr8	7187829	T	C	32	12,00%	1of6	4		6,02E-003
reg_DEFB	YES	chr8	7187910	G	T	43	19,00%	1of6	8		5,53E-006
reg_DEFB	YES	chr8	7187917	G	A	45	20,00%	1of6	9		7,54E-007
reg_DEFB	YES	chr8	7187918	C	T	45	20,00%	1of6	9		7,54E-007
reg_DEFB	YES	chr8	7187931	A	G	44	18,00%	1of6	8		6,62E-006
reg_DEFB	YES	chr8	7187943	T	C	44	16,00%	1of6	7		6,18E-005
reg_DEFB	YES	chr8	7187951	A	G	46	20,00%	1of6	9		9,18E-007
reg_DEFB	YES	chr8	7187955	T	G	46	20,00%	1of6	9		9,18E-007
reg_DEFB	YES	chr8	7187975	T	A	45	20,00%	1of6	9		7,54E-007
reg_DEFB	YES	chr8	7187998	C	T	50	20,00%	1of6	10		1,83E-007
reg_DEFB	YES	chr8	7187999	A	G	49	20,00%	1of6	10		1,50E-007
reg_DEFB	YES	chr8	7188045	G	C	52	17,00%	1of6	9		2,71E-006
reg_DEFB	YES	chr8	7188087	T	C	50	18,00%	1of6	9	rs71507400	1,92E-006

reg_DEFB	YES	chr8	7188090	G	C	49	16,00%	1of6	8			1,52E-005
reg_DEFB	YES	chr8	7188096	C	T	49	14,00%	1of6	7			1,25E-004
reg_DEFB	YES	chr8	7188105	T	C	48	15,00%	1of6	7			1,10E-004
reg_DEFB	YES	chr8	7188112	A	C	48	15,00%	1of6	7			1,10E-004
reg_DEFB	YES	chr8	7188124	C	T	50	14,00%	1of6	7			1,43E-004
reg_DEFB	YES	chr8	7188129	T	C	50	16,00%	1of6	8			1,77E-005
reg_DEFB	YES	chr8	7188148	G	T	50	12,00%	1of6	6			9,88E-004
reg_DEFB	YES	chr8	7188157	A	C	51	14,00%	1of6	7		rs6990411	1,62E-004
reg_DEFB	YES	chr8	7188181	A	G	53	11,00%	1of6	6			1,35E-003
reg_DEFB	YES	chr8	7188183	A	T	53	13,00%	1of6	7			2,08E-004
reg_DEFB	YES	chr8	7188797	A	G	83	14,00%	1of6	12			4,66E-007
reg_DEFB	YES	chr8	7188823	T	C	77	10,00%	1of6	8			4,02E-004
reg_DEFB	YES	chr8	7188856	A	C	67	16,00%	1of6	11			3,73E-007
reg_DEFB	YES	chr8	7188887	G	A	61	18,00%	1of6	11			1,38E-007
reg_DEFB	YES	chr8	7188958	C	A	50	14,00%	1of6	7		rs34459218	1,43E-004
reg_DEFB	YES	chr8	7188981	C	G	48	21,00%	1of6	10		rs9987086	1,22E-007
reg_DEFB	YES	chr8	7189009	T	A	42	24,00%	1of6	10		rs62493294	3,11E-008
reg_DEFB	YES	chr8	7189037	A	T	33	15,00%	1of6	5		rs6986093	8,92E-004
reg_DEFB	YES	chr8	7189054	A	G	29	17,00%	1of6	5		rs4733735	4,82E-004
reg_DEFB	YES	chr8	7189072	C	A	25	12,00%	1of6	3			1,92E-002
reg_DEFB	YES	chr8	7189264	T	G	22	14,00%	1of6	3	rs41480145		1,35E-002
reg_DEFB	YES	chr8	7189338	G	A	29	17,00%	1of6	5		rs62507166	4,82E-004
reg_DEFB	YES	chr8	7189349	C	G	29	17,00%	1of6	5		rs813630	4,82E-004
reg_DEFB	YES	chr8	7189475	G	A	37	19,00%	1of6	7			1,91E-005
reg_DEFB	YES	chr8	7189479	C	A	37	22,00%	1of6	8		rs7830327	1,66E-006
reg_DEFB	YES	chr8	7189483	G	T	38	18,00%	1of6	7		rs2204808	2,30E-005
reg_DEFB	YES	chr8	7189491	T	G	40	20,00%	1of6	8		rs7833507	3,12E-006
reg_DEFB	YES	chr8	7189540	C	G	44	20,00%	1of6	9			6,16E-007
reg_DEFB	YES	chr8	7189575	A	G	48	19,00%	1of6	9			1,34E-006
reg_DEFB	YES	chr8	7189590	T	A	47	17,00%	1of6	8		rs10955320	1,10E-005
reg_DEFB	YES	chr8	7189611	C	G	46	15,00%	1of6	7		rs9642830	8,29E-005
reg_DEFB	YES	chr8	7189621	C	T	44	14,00%	1of6	6		rs6999521	4,93E-004
reg_DEFB	YES	chr8	7189661	A	G	33	15,00%	1of6	5		rs7008838	8,92E-004
reg_DEFB	YES	chr8	7189693	A	G	29	14,00%	1of6	4			4,20E-003
reg_DEFB	YES	chr8	7191312	G	C	9	56,00%	3of6	5			7,51E-007
reg_DEFB	YES	chr8	7191353	G	A	12	58,00%	3of6	7		rs28393830	2,45E-009
reg_DEFB	YES	chr8	7191358	A	C	12	58,00%	3of6	7		rs2054796	2,45E-009
reg_DEFB	YES	chr8	7191398	A	G	17	53,00%	3of6	9		rs73318390	5,40E-011
reg_DEFB	YES	chr8	7191449	C	T	31	16,00%	1of6	5			6,64E-004
reg_DEFB	YES	chr8	7191450	A	G	32	47,00%	3of6	15			1,00E-012
reg_DEFB	YES	chr8	7191452	A	G	33	36,00%	2of6	12			1,03E-011
reg_DEFB	YES	chr8	7191456	C	T	33	15,00%	1of6	5			8,92E-004
reg_DEFB	YES	chr8	7191460	A	G	34	21,00%	1of6	7			1,06E-005
reg_DEFB	YES	chr8	7191518	C	A	52	17,00%	1of6	9			2,71E-006
reg_DEFB	YES	chr8	7191635	T	C	86	10,00%	1of6	9			1,68E-004
reg_DEFB	YES	chr8	7191645	G	A	88	10,00%	1of6	9			2,00E-004
reg_DEFB	YES	chr8	7191671	C	T	92	40,00%	2of6	37			1,00E-012
reg_DEFB	YES	chr8	7191773	T	C	99	29,00%	2of6	29		rs55699527	1,00E-012
reg_DEFB	YES	chr8	7191776	C	A	105	28,00%	2of6	29			1,00E-012
reg_DEFB	YES	chr8	7191808	C	T	104	13,00%	1of6	14			1,32E-007
reg_DEFB	YES	chr8	7191810	G	A	104	22,00%	1of6	23			1,00E-012
reg_DEFB	YES	chr8	7191820	T	C	102	25,00%	1of6	26			1,00E-012
reg_DEFB	YES	chr8	7191873	T	C	89	13,00%	1of6	12			1,01E-006

reg_DEFB	YES	chr8	7191967	A	G	67	21,00%	1of6	14		rs62495666	3,64E-010
reg_DEFB	YES	chr8	7192002	T	A	54	15,00%	1of6	8			3,17E-005
reg_DEFB	YES	chr8	7192669	T	G	77	56,00%	3of6	43			1,00E-012
reg_DEFB	YES	chr8	7193462	T	C	25	12,00%	1of6	3			1,92E-002
reg_DEFB	YES	chr8	7193490	T	C	26	12,00%	1of6	3			2,13E-002
reg_DEFB	YES	chr8	7193832	G	A	29	10,00%	1of6	3			2,85E-002
reg_DEFB	YES	chr8	7193966	C	A	46	15,00%	1of6	7			8,29E-005
reg_DEFB	YES	chr8	7194072	A	G	65	49,00%	3of6	32		rs71511216	1,00E-012
reg_DEFB	YES	chr8	7194133	G	A	82	13,00%	1of6	11		rs71518600	2,96E-006
reg_DEFB	YES	chr8	7194174	G	A	88	11,00%	1of6	10	rs2719612		3,64E-005
reg_DEFB	YES	chr8	7194267	C	G	100	51,00%	3of6	51	rs28413957		1,00E-012
reg_DEFB	YES	chr8	7194497	T	A	103	17,00%	1of6	18		rs4109383	3,44E-011
reg_DEFB	YES	chr8	7194523	T	C	102	18,00%	1of6	18			2,90E-011
reg_DEFB	YES	chr8	7194566	G	A	123	15,00%	1of6	18			5,71E-010
reg_DEFB	YES	chr8	7194608	A	G	136	10,00%	1of6	14			3,55E-006
reg_DEFB	YES	chr8	7194652	A	T	166	10,00%	1of6	17			3,62E-007
reg_DEFB	YES	chr8	7194683	C	G	180	11,00%	1of6	20			8,83E-009
reg_DEFB	YES	chr8	7194714	T	C	194	21,00%	1of6	41		rs71511217	1,00E-012
reg_DEFB	YES	chr8	7194763	C	A	228	10,00%	1of6	23			4,64E-009
reg_DEFB	YES	chr8	7194778	G	A	233	12,00%	1of6	28			7,61E-012
reg_DEFB	YES	chr8	7194795	C	T	246	12,00%	1of6	30			1,00E-012
reg_DEFB	YES	chr8	7194912	C	T	252	22,00%	1of6	55			1,00E-012
reg_DEFB	YES	chr8	7194919	A	C	252	16,00%	1of6	40			1,00E-012
reg_DEFB	YES	chr8	7194944	A	G	251	18,00%	1of6	45			1,00E-012
reg_DEFB	YES	chr8	7194961	G	T	250	38,00%	2of6	95			1,00E-012
reg_DEFB	YES	chr8	7195039	T	C	269	28,00%	2of6	75			1,00E-012
reg_DEFB	YES	chr8	7195053	C	T	269	42,00%	2of6	113		rs4406416	1,00E-012
reg_DEFB	YES	chr8	7195058	T	A	278	24,00%	1of6	67			1,00E-012
reg_DEFB	YES	chr8	7195060	T	C	277	18,00%	1of6	50			1,00E-012
reg_DEFB	YES	chr8	7195091	T	G	292	12,00%	1of6	35			1,00E-012
reg_DEFB	YES	chr8	7195125	A	T	277	24,00%	1of6	66			1,00E-012
reg_DEFB	YES	chr8	7195130	A	T	273	19,00%	1of6	52			1,00E-012
reg_DEFB	YES	chr8	7195137	T	C	266	35,00%	2of6	93	rs2698983		1,00E-012
reg_DEFB	YES	chr8	7195192	G	A	248	22,00%	1of6	55			1,00E-012
reg_DEFB	YES	chr8	7195194	C	T	251	22,00%	1of6	55			1,00E-012
reg_DEFB	YES	chr8	7195197	T	G	251	11,00%	1of6	28			1,66E-011
reg_DEFB	YES	chr8	7195256	T	C	219	12,00%	1of6	26			2,08E-011
reg_DEFB	YES	chr8	7195278	G	C	205	12,00%	1of6	25			1,90E-011
reg_DEFB	YES	chr8	7195405	C	T	160	16,00%	1of6	26			1,00E-012
reg_DEFB	YES	chr8	7195438	C	T	154	20,00%	1of6	31	rs2684172		1,00E-012
reg_DEFB	YES	chr8	7195559	C	T	159	10,00%	1of6	16			9,99E-007
reg_DEFB	YES	chr8	7195583	G	T	152	12,00%	1of6	18			1,77E-008
reg_DEFB	YES	chr8	7195589	T	G	150	33,00%	2of6	50			1,00E-012
reg_DEFB	YES	chr8	7195614	C	A	134	15,00%	1of6	20			4,66E-011
reg_DEFB	YES	chr8	7195618	G	C	131	11,00%	1of6	14			2,28E-006
reg_DEFB	YES	chr8	7195621	C	A	129	12,00%	1of6	15		rs2739953	3,37E-007
reg_DEFB	YES	chr8	7195701	A	G	110	45,00%	3of6	50			1,00E-012
reg_DEFB	YES	chr8	7195707	C	A	111	22,00%	1of6	24			1,00E-012
reg_DEFB	YES	chr8	7195721	T	C	108	55,00%	3of6	59			1,00E-012
reg_DEFB	YES	chr8	7195766	A	T	116	18,00%	1of6	21			1,00E-012
reg_DEFB	YES	chr8	7195768	C	T	116	16,00%	1of6	19			3,30E-011
reg_DEFB	YES	chr8	7195788	T	C	114	19,00%	1of6	22			1,00E-012
reg_DEFB	YES	chr8	7195837	A	T	114	26,00%	2of6	30			1,00E-012

reg_DEFB	YES	chr8	7195838	G	C	114	19,00%	1of6	22			1,00E-012
reg_DEFB	YES	chr8	7195875	C	G	111	26,00%	2of6	29	rs9644813		1,00E-012
reg_DEFB	YES	chr8	7195903	C	T	102	49,00%	3of6	50			1,00E-012
reg_DEFB	YES	chr8	7195918	A	G	103	54,00%	3of6	56			1,00E-012
reg_DEFB	YES	chr8	7195928	C	G	101	20,00%	1of6	20			1,00E-012
reg_DEFB	YES	chr8	7196045	T	C	94	45,00%	3of6	42			1,00E-012
reg_DEFB	YES	chr8	7196080	G	T	99	32,00%	2of6	32			1,00E-012
reg_DEFB	YES	chr8	7196102	T	C	101	15,00%	1of6	15			1,23E-008
reg_DEFB	YES	chr8	7196131	C	G	102	19,00%	1of6	19	rs11783399		8,15E-012
reg_DEFB	YES	chr8	7196166	T	A	91	21,00%	1of6	19			1,00E-012
reg_DEFB	YES	chr8	7196370	A	G	100	41,00%	2of6	41	rs11786963		1,00E-012
reg_DEFB	YES	chr8	7196373	G	A	101	22,00%	1of6	22	rs11776329		1,00E-012
reg_DEFB	YES	chr8	7196542	A	G	139	43,00%	3of6	60	rs2719605		1,00E-012
reg_DEFB	YES	chr8	7196637	G	T	179	10,00%	1of6	18			2,19E-007
reg_DEFB	YES	chr8	7196648	G	A	180	13,00%	1of6	23			4,55E-011
reg_DEFB	YES	chr8	7196689	T	C	184	13,00%	1of6	24			1,56E-011
reg_DEFB	YES	chr8	7198085	C	G	60	28,00%	2of6	17	rs2719598		1,00E-012
reg_DEFB	YES	chr8	7198660	A	G	23	30,00%	2of6	7			6,03E-007
reg_DEFB	YES	chr8	7198678	G	A	23	17,00%	1of6	4			1,75E-003
reg_DEFB	YES	chr8	7198710	C	T	27	11,00%	1of6	3			2,36E-002
reg_DEFB	YES	chr8	7198835	T	C	45	13,00%	1of6	6			5,58E-004
reg_DEFB	YES	chr8	7198855	C	T	49	43,00%	3of6	21			1,00E-012
reg_DEFB	YES	chr8	7198887	G	A	56	16,00%	1of6	9			5,13E-006
reg_DEFB	YES	chr8	7198888	C	A	55	16,00%	1of6	9			4,40E-006
reg_DEFB	YES	chr8	7198932	G	T	56	21,00%	1of6	12			4,78E-009
reg_DEFB	YES	chr8	7198953	A	G	61	41,00%	2of6	25			1,00E-012
reg_DEFB	YES	chr8	7198957	G	A	62	13,00%	1of6	8			8,75E-005
reg_DEFB	YES	chr8	7198962	G	A	62	11,00%	1of6	7			5,53E-004
reg_DEFB	YES	chr8	7198985	C	T	66	14,00%	1of6	9			2,04E-005
reg_DEFB	YES	chr8	7198997	C	T	70	44,00%	3of6	31			1,00E-012
reg_DEFB	YES	chr8	7199006	A	T	71	42,00%	2of6	30			1,00E-012
reg_DEFB	YES	chr8	7199022	G	T	72	42,00%	2of6	30			1,00E-012
reg_DEFB	YES	chr8	7199050	C	A	79	14,00%	1of6	11			2,04E-006
reg_DEFB	YES	chr8	7199054	G	A	80	10,00%	1of6	8			5,21E-004
reg_DEFB	YES	chr8	7199055	A	T	80	16,00%	1of6	13			3,76E-008
reg_DEFB	YES	chr8	7199057	G	A	79	39,00%	2of6	31		rs2739951	1,00E-012
reg_DEFB	YES	chr8	7199070	T	G	74	15,00%	1of6	11			1,05E-006
reg_DEFB	YES	chr8	7199090	C	T	74	23,00%	1of6	17			2,76E-012
reg_DEFB	YES	chr8	7199114	G	A	72	12,00%	1of6	9			4,14E-005
reg_DEFB	YES	chr8	7199209	A	G	69	29,00%	2of6	20		rs2698933	1,00E-012
reg_DEFB	YES	chr8	7199227	G	A	69	10,00%	1of6	7			1,06E-003
reg_DEFB	YES	chr8	7199246	T	G	76	22,00%	1of6	17	rs2719594		4,48E-012
reg_DEFB	YES	chr8	7199260	T	G	80	24,00%	1of6	19			1,00E-012
reg_DEFB	YES	chr8	7199341	A	C	93	14,00%	1of6	13	rs2954093		2,35E-007
reg_DEFB	YES	chr8	7199423	C	T	106	16,00%	1of6	17			4,04E-010
reg_DEFB	YES	chr8	7199439	A	T	109	16,00%	1of6	17			6,17E-010
reg_DEFB	YES	chr8	7199492	A	C	107	10,00%	1of6	11	rs2719593		3,88E-005
reg_DEFB	YES	chr8	7199565	T	C	94	33,00%	2of6	31	rs41494048		1,00E-012
reg_DEFB	YES	chr8	7199580	G	A	91	32,00%	2of6	29	rs2719592		1,00E-012
reg_DEFB	YES	chr8	7199703	G	A	60	38,00%	2of6	23		rs4452804	1,00E-012
reg_DEFB	YES	chr8	7199777	C	T	28	36,00%	2of6	10			3,84E-010
reg_DEFB	YES	chr8	7199855	A	G	13	23,00%	1of6	3			2,93E-003
reg_DEFB	YES	chr8	7199887	A	C	12	42,00%	2of6	5		rs4471067	4,45E-006

reg_DEFB	YES	chr8	7199944	G	T	9	33,00%	2of6	3							9,21E-004
reg_DEFB	YES	chr8	7200009	G	A	9	44,00%	3of6	4					rs11783983		3,21E-005
reg_DEFB	YES	chr8	7200026	G	C	8	50,00%	3of6	4							1,82E-005
reg_DEFB	YES	chr8	7200043	G	A	7	43,00%	3of6	3							3,97E-004
reg_DEFB	YES	chr8	7200052	G	T	7	43,00%	3of6	3							3,97E-004
reg_DEFB	YES	chr8	7200141	C	G	13	23,00%	1of6	3							2,93E-003
reg_DEFB	YES	chr8	7200334	G	T	25	44,00%	3of6	11							5,49E-012
reg_DEFB	YES	chr8	7201007	C	G	92	25,00%	1of6	23						rs59917219	1,00E-012
reg_DEFB	YES	chr8	7201053	T	C	95	27,00%	2of6	26						rs71511221	1,00E-012
reg_DEFB	YES	chr8	7201379	T	G	114	14,00%	1of6	16							9,35E-009
reg_DEFB	YES	chr8	7201387	C	T	119	24,00%	1of6	29					rs9720953		1,00E-012
reg_DEFB	YES	chr8	7201478	A	G	141	15,00%	1of6	21							2,11E-011
reg_DEFB	YES	chr8	7201552	G	A	153	18,00%	1of6	28							1,00E-012
reg_DEFB	YES	chr8	7201604	G	A	166	11,00%	1of6	18							6,96E-008
reg_DEFB	YES	chr8	7201662	A	G	177	19,00%	1of6	34							1,00E-012
reg_DEFB	YES	chr8	7201669	G	A	176	35,00%	2of6	62							1,00E-012
reg_DEFB	YES	chr8	7201678	C	G	179	34,00%	2of6	61							1,00E-012
reg_DEFB	YES	chr8	7201691	C	T	173	34,00%	2of6	59							1,00E-012
reg_DEFB	YES	chr8	7201816	C	T	153	16,00%	1of6	24							1,00E-012
reg_DEFB	YES	chr8	7201817	G	T	153	16,00%	1of6	24							1,00E-012
reg_DEFB	YES	chr8	7202124	T	A	139	13,00%	1of6	18							4,24E-009
reg_DEFB	YES	chr8	7202227	T	C	115	15,00%	1of6	17							1,45E-009
reg_DEFB	YES	chr8	7202247	C	T	110	14,00%	1of6	15					rs2466110		3,99E-008
reg_DEFB	YES	chr8	7202290	G	A	107	11,00%	1of6	12							7,17E-006
reg_DEFB	YES	chr8	7202297	T	C	109	28,00%	2of6	31							1,00E-012
reg_DEFB	YES	chr8	7202739	T	C	99	42,00%	2of6	42					rs9720695		1,00E-012
reg_DEFB	YES	chr8	7202754	C	T	102	33,00%	2of6	34						rs55799624	1,00E-012
reg_DEFB	YES	chr8	7202856	T	C	109	30,00%	2of6	33					rs9693661		1,00E-012
reg_DEFB	YES	chr8	7202894	C	T	109	38,00%	2of6	41					rs11779457		1,00E-012
reg_DEFB	YES	chr8	7203104	A	G	105	30,00%	2of6	32	V	A	-1	ZNF705G	rs9693671		1,00E-012
reg_DEFB	YES	chr8	7203333	C	T	111	13,00%	1of6	14	E	K	-1	ZNF705G			3,00E-007
reg_DEFB	YES	chr8	7203336	T	G	110	12,00%	1of6	13	T	P	-1	ZNF705G			1,67E-006
reg_DEFB	YES	chr8	7203350	C	T	108	12,00%	1of6	13	R	H	-1	ZNF705G			1,35E-006
reg_DEFB	YES	chr8	7203356	G	C	108	12,00%	1of6	13	S	C	-1	ZNF705G			1,35E-006
reg_DEFB	YES	chr8	7203431	C	T	112	18,00%	1of6	20	R	H	-1	ZNF705G		rs59326007	5,18E-012
reg_DEFB	YES	chr8	7203440	T	C	113	14,00%	1of6	16	D	G	-1	ZNF705G			8,21E-009
reg_DEFB	YES	chr8	7203449	G	A	113	14,00%	1of6	16	S	L	-1	ZNF705G			8,21E-009
reg_DEFB	YES	chr8	7203472	C	A	106	14,00%	1of6	15	E	D	-1	ZNF705G			2,40E-008
reg_DEFB	YES	chr8	7203505	T	G	97	16,00%	1of6	16			-	ZNF705G			8,33E-010
reg_DEFB	YES	chr8	7203509	A	T	97	13,00%	1of6	13			-	ZNF705G			3,87E-007
reg_DEFB	YES	chr8	7203604	G	T	70	13,00%	1of6	9			-	ZNF705G			3,30E-005
reg_DEFB	YES	chr8	7203688	C	T	59	10,00%	1of6	6			-	ZNF705G			2,35E-003
reg_DEFB	YES	chr8	7203891	C	T	49	12,00%	1of6	6			-	ZNF705G			8,86E-004
reg_DEFB	YES	chr8	7204029	T	A	46	13,00%	1of6	6			-	ZNF705G			6,30E-004
reg_DEFB	YES	chr8	7204073	C	A	46	11,00%	1of6	5			-	ZNF705G			4,03E-003
reg_DEFB	YES	chr8	7204170	A	T	59	10,00%	1of6	6			-	ZNF705G			2,35E-003
reg_DEFB	YES	chr8	7204182	G	T	61	11,00%	1of6	7			-	ZNF705G			5,01E-004
reg_DEFB	YES	chr8	7204218	A	T	73	18,00%	1of6	13			-	ZNF705G			1,20E-008
reg_DEFB	YES	chr8	7204229	A	G	74	11,00%	1of6	8			-	ZNF705G			3,06E-004
reg_DEFB	YES	chr8	7204281	C	A	99	15,00%	1of6	15			-	ZNF705G			9,27E-009
reg_DEFB	YES	chr8	7204304	A	G	104	11,00%	1of6	11			-	ZNF705G			2,97E-005
reg_DEFB	YES	chr8	7204317	G	A	117	21,00%	1of6	25			-	ZNF705G			1,00E-012
reg_DEFB	YES	chr8	7204321	A	T	117	21,00%	1of6	25			-	ZNF705G			1,00E-012

reg_DEFB	YES	chr8	7204442	A	G	123	22,00%	1of6	27			-	ZNF705G			1,00E-012
reg_DEFB	YES	chr8	7204457	T	C	129	19,00%	1of6	25			-	ZNF705G			1,00E-012
reg_DEFB	YES	chr8	7204497	T	C	129	19,00%	1of6	25			-	ZNF705G		rs55661827	1,00E-012
reg_DEFB	YES	chr8	7204504	A	C	131	19,00%	1of6	25			-	ZNF705G			1,00E-012
reg_DEFB	YES	chr8	7204582	G	A	144	10,00%	1of6	14	T	I	-2	ZNF705G		rs56023905	6,93E-006
reg_DEFB	YES	chr8	7204731	G	T	124	12,00%	1of6	15			-	ZNF705G			2,00E-007
reg_DEFB	YES	chr8	7204796	A	G	138	11,00%	1of6	15			-	ZNF705G			8,08E-007
reg_DEFB	YES	chr8	7204809	T	G	143	26,00%	2of6	37			-	ZNF705G			1,00E-012
reg_DEFB	YES	chr8	7204869	A	T	153	37,00%	2of6	57			-	ZNF705G			1,00E-012
reg_DEFB	YES	chr8	7204899	A	G	153	20,00%	1of6	31			-	ZNF705G			1,00E-012
reg_DEFB	YES	chr8	7204956	C	T	156	23,00%	1of6	36			-	ZNF705G			1,00E-012
reg_DEFB	YES	chr8	7204963	G	A	152	24,00%	1of6	36			-	ZNF705G			1,00E-012
reg_DEFB	YES	chr8	7204973	A	T	148	23,00%	1of6	34			-	ZNF705G			1,00E-012
reg_DEFB	YES	chr8	7205020	T	C	143	22,00%	1of6	31			-	ZNF705G			1,00E-012
reg_DEFB	YES	chr8	7205055	A	G	132	20,00%	1of6	26			-	ZNF705G			1,00E-012
reg_DEFB	YES	chr8	7205169	T	C	100	40,00%	2of6	40	S	G	-2	ZNF705G			1,00E-012
reg_DEFB	YES	chr8	7205192	A	T	96	33,00%	2of6	32	V	E	-2	ZNF705G			1,00E-012
reg_DEFB	YES	chr8	7205205	T	A	94	15,00%	1of6	14	R	W	-2	ZNF705G	rs9721188	T	3,59E-008
reg_DEFB	YES	chr8	7205247	A	C	89	18,00%	1of6	16	S	A	-2	ZNF705G	rs3989701		2,25E-010
reg_DEFB	YES	chr8	7205333	T	G	75	15,00%	1of6	11			-	ZNF705G	rs3958834		1,20E-006
reg_DEFB	YES	chr8	7205528	G	A	121	26,00%	2of6	31			-	ZNF705G			1,00E-012
reg_DEFB	YES	chr8	7205569	T	A	147	27,00%	2of6	40			-	ZNF705G			1,00E-012
reg_DEFB	YES	chr8	7205590	C	T	161	29,00%	2of6	47			-	ZNF705G			1,00E-012
reg_DEFB	YES	chr8	7205625	A	T	165	12,00%	1of6	20			-	ZNF705G			1,94E-009
reg_DEFB	YES	chr8	7205630	C	G	165	13,00%	1of6	21			-	ZNF705G			3,15E-010
reg_DEFB	YES	chr8	7205634	A	T	166	13,00%	1of6	22			-	ZNF705G			5,73E-011
reg_DEFB	YES	chr8	7205677	C	A	180	41,00%	2of6	74			-	ZNF705G			1,00E-012
reg_DEFB	YES	chr8	7205696	A	G	186	16,00%	1of6	30			-	ZNF705G			1,00E-012
reg_DEFB	YES	chr8	7205712	G	A	191	15,00%	1of6	29			-	ZNF705G			1,00E-012
reg_DEFB	YES	chr8	7205734	T	G	195	12,00%	1of6	23			-	ZNF705G			2,18E-010
reg_DEFB	YES	chr8	7205738	T	G	193	17,00%	1of6	33			-	ZNF705G			1,00E-012
reg_DEFB	YES	chr8	7205746	C	T	193	46,00%	3of6	89			-	ZNF705G			1,00E-012
reg_DEFB	YES	chr8	7205753	C	T	196	79,00%	5of6	155			-	ZNF705G	rs3958831		1,00E-012
reg_DEFB	YES	chr8	7205758	A	G	195	14,00%	1of6	27			-	ZNF705G			1,00E-012
reg_DEFB	YES	chr8	7205760	T	C	195	30,00%	2of6	59			-	ZNF705G			1,00E-012
reg_DEFB	YES	chr8	7205778	A	G	195	13,00%	1of6	25			-	ZNF705G	rs3989700		8,77E-012
reg_DEFB	YES	chr8	7205901	G	A	139	14,00%	1of6	19			-	ZNF705G			6,33E-010
reg_DEFB	YES	chr8	7205911	C	T	132	23,00%	1of6	30			-	ZNF705G			1,00E-012
reg_DEFB	YES	chr8	7205938	T	G	132	23,00%	1of6	30			-	ZNF705G			1,00E-012
reg_DEFB	YES	chr8	7205976	T	C	125	16,00%	1of6	20			-	ZNF705G			1,60E-011
reg_DEFB	YES	chr8	7205999	T	C	127	13,00%	1of6	17			-	ZNF705G			6,79E-009
reg_DEFB	YES	chr8	7206008	C	G	123	11,00%	1of6	14			-	ZNF705G			1,07E-006
reg_DEFB	YES	chr8	7206031	C	G	130	38,00%	2of6	49			-	ZNF705G	rs3989699		1,00E-012
reg_DEFB	YES	chr8	7206042	C	T	130	25,00%	1of6	33	G	S	-1	ZNF705G	rs3989698		1,00E-012
reg_DEFB	YES	chr8	7206118	C	T	153	12,00%	1of6	18	W	*	-1	ZNF705G			1,96E-008
reg_DEFB	YES	chr8	7206162	G	C	156	42,00%	2of6	66	L	V	-1	ZNF705G	rs3989697		1,00E-012
reg_DEFB	YES	chr8	7206207	T	C	167	22,00%	1of6	37			-	ZNF705G			1,00E-012
reg_DEFB	YES	chr8	7206251	C	A	192	17,00%	1of6	33			-	ZNF705G			1,00E-012
reg_DEFB	YES	chr8	7206282	G	A	195	17,00%	1of6	33			-	ZNF705G		G	1,00E-012
reg_DEFB	YES	chr8	7206307	C	G	196	17,00%	1of6	33			-	ZNF705G			1,00E-012
reg_DEFB	YES	chr8	7206323	C	A	194	18,00%	1of6	35			-	ZNF705G			1,00E-012
reg_DEFB	YES	chr8	7206369	G	C	189	37,00%	2of6	70			-	ZNF705G			1,00E-012
reg_DEFB	YES	chr8	7206389	A	G	196	38,00%	2of6	74			-	ZNF705G			1,00E-012

reg_DEFB	YES	chr8	7206391	A	G	199	10,00%	1of6	20	-	ZNF705G			4,83E-008
reg_DEFB	YES	chr8	7206401	G	A	198	23,00%	1of6	46	-	ZNF705G	rs11986853		1,00E-012
reg_DEFB	YES	chr8	7206449	A	G	197	24,00%	1of6	47	-	ZNF705G			1,00E-012
reg_DEFB	YES	chr8	7206530	T	C	185	11,00%	1of6	20	-	ZNF705G	rs3935666		1,41E-008
reg_DEFB	YES	chr8	7206561	C	T	187	40,00%	2of6	75	-	ZNF705G			1,00E-012
reg_DEFB	YES	chr8	7206566	G	A	185	25,00%	1of6	46	-	ZNF705G	rs3989696		1,00E-012
reg_DEFB	YES	chr8	7206569	T	C	187	17,00%	1of6	32	-	ZNF705G			1,00E-012
reg_DEFB	YES	chr8	7206574	C	G	191	41,00%	2of6	78	-	ZNF705G			1,00E-012
reg_DEFB	YES	chr8	7206587	T	C	184	15,00%	1of6	28	-	ZNF705G			1,00E-012
reg_DEFB	YES	chr8	7206596	C	T	184	43,00%	3of6	79	-	ZNF705G			1,00E-012
reg_DEFB	YES	chr8	7206634	A	G	179	18,00%	1of6	32	-	ZNF705G			1,00E-012
reg_DEFB	YES	chr8	7206666	A	G	189	10,00%	1of6	19	-	ZNF705G			1,03E-007
reg_DEFB	YES	chr8	7206679	C	T	189	16,00%	1of6	30	-	ZNF705G			1,00E-012
reg_DEFB	YES	chr8	7206722	G	A	187	18,00%	1of6	34	-	ZNF705G			1,00E-012
reg_DEFB	YES	chr8	7206730	T	C	189	13,00%	1of6	25	-	ZNF705G			1,25E-011
reg_DEFB	YES	chr8	7206733	A	G	190	16,00%	1of6	30	-	ZNF705G		rs62636816	1,00E-012
reg_DEFB	YES	chr8	7206756	C	A	199	17,00%	1of6	34	-	ZNF705G			1,00E-012
reg_DEFB	YES	chr8	7206785	C	A	199	16,00%	1of6	32	-	ZNF705G			1,00E-012
reg_DEFB	YES	chr8	7206819	T	C	209	42,00%	2of6	88	-	ZNF705G			1,00E-012
reg_DEFB	YES	chr8	7206830	T	C	203	14,00%	1of6	28	-	ZNF705G			1,00E-012
reg_DEFB	YES	chr8	7206852	A	G	196	38,00%	2of6	74	-	ZNF705G			1,00E-012
reg_DEFB	YES	chr8	7206930	C	T	184	20,00%	1of6	37	-	ZNF705G			1,00E-012
reg_DEFB	YES	chr8	7206944	A	T	184	15,00%	1of6	28	-	ZNF705G			1,00E-012
reg_DEFB	YES	chr8	7206999	C	T	177	42,00%	2of6	74	-	ZNF705G			1,00E-012
reg_DEFB	YES	chr8	7207022	C	G	179	13,00%	1of6	23	-	ZNF705G			4,05E-011
reg_DEFB	YES	chr8	7207028	A	T	179	44,00%	3of6	79	-	ZNF705G			1,00E-012
reg_DEFB	YES	chr8	7207076	C	T	168	21,00%	1of6	35	-	ZNF705G			1,00E-012
reg_DEFB	YES	chr8	7207082	T	A	167	11,00%	1of6	18	-	ZNF705G			7,64E-008
reg_DEFB	YES	chr8	7207091	A	G	170	17,00%	1of6	29	-	ZNF705G			1,00E-012
reg_DEFB	YES	chr8	7207152	A	G	149	19,00%	1of6	28	-	ZNF705G			1,00E-012
reg_DEFB	YES	chr8	7207187	T	C	144	37,00%	2of6	53	-	ZNF705G			1,00E-012
reg_DEFB	YES	chr8	7207189	C	G	144	12,00%	1of6	17	-	ZNF705G			4,55E-008
reg_DEFB	YES	chr8	7207228	G	A	146	29,00%	2of6	42	-	ZNF705G			1,00E-012
reg_DEFB	YES	chr8	7207234	G	A	144	13,00%	1of6	19	-	ZNF705G			1,16E-009
reg_DEFB	YES	chr8	7207252	G	A	149	13,00%	1of6	19	-	ZNF705G			2,07E-009
reg_DEFB	YES	chr8	7207260	T	C	149	14,00%	1of6	21	-	ZNF705G			4,73E-011
reg_DEFB	YES	chr8	7207285	A	G	142	15,00%	1of6	21	-	ZNF705G			2,43E-011
reg_DEFB	YES	chr8	7207297	C	T	143	31,00%	2of6	44	-	ZNF705G			1,00E-012
reg_DEFB	YES	chr8	7207446	A	C	141	18,00%	1of6	25	-	ZNF705G			1,00E-012
reg_DEFB	YES	chr8	7207471	G	C	141	15,00%	1of6	21	-	ZNF705G			2,11E-011
reg_DEFB	YES	chr8	7207480	T	C	142	17,00%	1of6	24	-	ZNF705G			1,00E-012
reg_DEFB	YES	chr8	7208546	T	G	26	23,00%	1of6	6			rs12542313		2,29E-005
reg_DEFB	YES	chr8	7208551	G	A	28	25,00%	1of6	7					2,63E-006
reg_DEFB	YES	chr8	7208586	G	T	32	28,00%	2of6	9					3,13E-008
reg_DEFB	YES	chr8	7208588	A	G	31	16,00%	1of6	5				rs71511226	6,64E-004
reg_DEFB	YES	chr8	7208657	A	G	54	41,00%	2of6	22					1,00E-012
reg_DEFB	YES	chr8	7208678	A	G	59	20,00%	1of6	12					8,98E-009
reg_DEFB	YES	chr8	7208731	T	C	77	12,00%	1of6	9					7,08E-005
reg_DEFB	YES	chr8	7208794	G	A	99	12,00%	1of6	12					3,17E-006
reg_DEFB	YES	chr8	7208816	C	A	109	11,00%	1of6	12					8,69E-006
reg_DEFB	YES	chr8	7208837	A	C	129	44,00%	3of6	57					1,00E-012
reg_DEFB	YES	chr8	7208881	C	T	155	44,00%	3of6	68					1,00E-012
reg_DEFB	YES	chr8	7208887	A	T	160	11,00%	1of6	18					3,94E-008

reg_DEFB	YES	chr8	7208898	C	G	171	25,00%	1of6	43			1,00E-012
reg_DEFB	YES	chr8	7208906	T	C	174	26,00%	2of6	45			1,00E-012
reg_DEFB	YES	chr8	7208911	A	T	181	12,00%	1of6	22			2,97E-010
reg_DEFB	YES	chr8	7208920	C	T	186	11,00%	1of6	20			1,55E-008
reg_DEFB	YES	chr8	7208925	A	T	186	12,00%	1of6	22			5,04E-010
reg_DEFB	YES	chr8	7208940	T	C	188	23,00%	1of6	43			1,00E-012
reg_DEFB	YES	chr8	7208951	T	G	194	35,00%	2of6	68			1,00E-012
reg_DEFB	YES	chr8	7208956	A	G	195	39,00%	2of6	76			1,00E-012
reg_DEFB	YES	chr8	7208958	A	T	196	45,00%	3of6	88			1,00E-012
reg_DEFB	YES	chr8	7208996	C	T	219	15,00%	1of6	33			1,00E-012
reg_DEFB	YES	chr8	7209021	A	G	232	24,00%	1of6	56			1,00E-012
reg_DEFB	YES	chr8	7209090	C	T	225	34,00%	2of6	77			1,00E-012
reg_DEFB	YES	chr8	7209105	C	T	224	53,00%	3of6	119			1,00E-012
reg_DEFB	YES	chr8	7209110	G	A	221	34,00%	2of6	75			1,00E-012
reg_DEFB	YES	chr8	7209126	A	G	213	55,00%	3of6	117			1,00E-012
reg_DEFB	YES	chr8	7209200	T	C	170	21,00%	1of6	36			1,00E-012
reg_DEFB	YES	chr8	7209211	A	G	170	18,00%	1of6	31			1,00E-012
reg_DEFB	YES	chr8	7209224	T	C	163	12,00%	1of6	20			1,57E-009
reg_DEFB	YES	chr8	7209238	A	G	164	13,00%	1of6	21			2,81E-010
reg_DEFB	YES	chr8	7209244	A	G	161	19,00%	1of6	31			1,00E-012
reg_DEFB	YES	chr8	7209275	C	A	145	17,00%	1of6	25			1,00E-012
reg_DEFB	YES	chr8	7209286	T	C	144	21,00%	1of6	30			1,00E-012
reg_DEFB	YES	chr8	7209380	A	C	125	19,00%	1of6	24			1,00E-012
reg_DEFB	YES	chr8	7209405	C	A	124	15,00%	1of6	19			8,79E-011
reg_DEFB	YES	chr8	7209417	T	G	122	17,00%	1of6	21			3,23E-012
reg_DEFB	YES	chr8	7209477	C	T	112	12,00%	1of6	13			2,05E-006
reg_DEFB	YES	chr8	7209495	G	C	104	36,00%	2of6	37	rs10107229		1,00E-012
reg_DEFB	YES	chr8	7209500	G	A	106	18,00%	1of6	19			1,68E-011
reg_DEFB	YES	chr8	7209507	C	T	107	10,00%	1of6	11			3,88E-005
reg_DEFB	YES	chr8	7209543	C	G	101	34,00%	2of6	34			1,00E-012
reg_DEFB	YES	chr8	7209552	T	A	106	13,00%	1of6	14			1,68E-007
reg_DEFB	YES	chr8	7209570	T	A	107	11,00%	1of6	12			7,17E-006
reg_DEFB	YES	chr8	7209584	G	T	104	38,00%	2of6	40			1,00E-012
reg_DEFB	YES	chr8	7209625	T	C	134	11,00%	1of6	15			5,53E-007
reg_DEFB	YES	chr8	7209708	A	G	144	37,00%	2of6	53			1,00E-012
reg_DEFB	YES	chr8	7209711	T	C	148	37,00%	2of6	55			1,00E-012
reg_DEFB	YES	chr8	7209802	C	A	169	15,00%	1of6	25			1,00E-012
reg_DEFB	YES	chr8	7209809	T	C	163	15,00%	1of6	24			3,05E-012
reg_DEFB	YES	chr8	7209834	T	G	168	47,00%	3of6	79			1,00E-012
reg_DEFB	YES	chr8	7209849	A	G	170	31,00%	2of6	53			1,00E-012
reg_DEFB	YES	chr8	7209884	A	T	169	50,00%	3of6	85			1,00E-012
reg_DEFB	YES	chr8	7209889	A	G	168	38,00%	2of6	64			1,00E-012
reg_DEFB	YES	chr8	7209936	G	A	166	11,00%	1of6	18			6,96E-008
reg_DEFB	YES	chr8	7209960	A	G	172	13,00%	1of6	22			1,17E-010
reg_DEFB	YES	chr8	7210033	T	C	138	18,00%	1of6	25			1,00E-012
reg_DEFB	YES	chr8	7210112	A	T	98	49,00%	3of6	48	rs12681401		1,00E-012
reg_DEFB	YES	chr8	7210190	G	A	69	48,00%	3of6	33		rs71241132	1,00E-012
reg_DEFB	YES	chr8	7210207	G	A	61	15,00%	1of6	9			1,06E-005
reg_DEFB	YES	chr8	7210229	A	G	52	44,00%	3of6	23			1,00E-012
reg_DEFB	YES	chr8	7210238	C	A	48	17,00%	1of6	8			1,30E-005
reg_DEFB	YES	chr8	7210338	A	T	17	18,00%	1of6	3			6,50E-003
reg_DEFB	YES	chr8	7210441	G	T	25	36,00%	2of6	9			2,64E-009
reg_DEFB	YES	chr8	7210734	A	C	115	16,00%	1of6	18			1,89E-010

reg_DEFB	YES	chr8	7210769	T	C	132	17,00%	1of6	22		1,94E-012
reg_DEFB	YES	chr8	7210821	A	G	141	20,00%	1of6	28		1,00E-012
reg_DEFB	YES	chr8	7210836	G	C	148	22,00%	1of6	33		1,00E-012
reg_DEFB	YES	chr8	7210889	C	T	165	15,00%	1of6	25		1,00E-012
reg_DEFB	YES	chr8	7210899	G	C	166	28,00%	2of6	46	rs11137100	1,00E-012
reg_DEFB	YES	chr8	7210912	C	A	163	26,00%	2of6	42		1,00E-012
reg_DEFB	YES	chr8	7210988	G	C	171	11,00%	1of6	19	rs3989690	2,05E-008
reg_DEFB	YES	chr8	7211001	A	T	170	26,00%	2of6	44		1,00E-012
reg_DEFB	YES	chr8	7211163	G	C	137	13,00%	1of6	18		3,35E-009
reg_DEFB	YES	chr8	7211198	C	G	128	16,00%	1of6	20	rs3989724	2,54E-011
reg_DEFB	YES	chr8	7211270	A	G	105	12,00%	1of6	13		9,76E-007
reg_DEFB	YES	chr8	7211286	C	T	105	31,00%	2of6	33	rs3989723	1,00E-012
reg_DEFB	YES	chr8	7211302	G	T	108	15,00%	1of6	16	rs3989722	4,20E-009
reg_DEFB	YES	chr8	7211344	G	A	107	18,00%	1of6	19	rs3989721	7,31E-012
reg_DEFB	YES	chr8	7211388	G	C	112	25,00%	1of6	28	rs3989720	1,00E-012
reg_DEFB	YES	chr8	7211474	G	T	117	26,00%	2of6	30		1,00E-012
reg_DEFB	YES	chr8	7211500	A	G	118	19,00%	1of6	22	rs3989719	1,00E-012
reg_DEFB	YES	chr8	7211502	G	T	118	25,00%	1of6	30	rs3989718	1,00E-012
reg_DEFB	YES	chr8	7211511	T	C	120	20,00%	1of6	24		1,00E-012
reg_DEFB	YES	chr8	7211524	G	A	125	24,00%	1of6	30	rs3989717	1,00E-012
reg_DEFB	YES	chr8	7211575	C	A	127	18,00%	1of6	23		1,00E-012
reg_DEFB	YES	chr8	7211592	G	A	125	18,00%	1of6	23		1,00E-012
reg_DEFB	YES	chr8	7211652	C	A	113	12,00%	1of6	14		3,75E-007
reg_DEFB	YES	chr8	7211679	G	A	106	14,00%	1of6	15	rs9314626	2,40E-008
reg_DEFB	YES	chr8	7211695	C	A	109	12,00%	1of6	13		1,50E-006
reg_DEFB	YES	chr8	7212014	A	G	98	11,00%	1of6	11		1,69E-005
reg_DEFB	YES	chr8	7212099	C	T	96	30,00%	2of6	29		1,00E-012
reg_DEFB	YES	chr8	7212118	G	A	97	11,00%	1of6	11		1,54E-005
reg_DEFB	YES	chr8	7212160	C	T	93	13,00%	1of6	12		1,62E-006
reg_DEFB	YES	chr8	7212186	C	A	88	10,00%	1of6	9		2,00E-004
reg_DEFB	YES	chr8	7212211	G	T	90	11,00%	1of6	10		4,43E-005
reg_DEFB	YES	chr8	7212213	T	C	90	13,00%	1of6	12		1,14E-006
reg_DEFB	YES	chr8	7212223	A	G	91	27,00%	2of6	25		1,00E-012
reg_DEFB	YES	chr8	7212234	T	G	85	27,00%	2of6	23		1,00E-012
reg_DEFB	YES	chr8	7212276	G	A	89	13,00%	1of6	12		1,01E-006
reg_DEFB	YES	chr8	7212316	G	A	84	35,00%	2of6	29		1,00E-012
reg_DEFB	YES	chr8	7212317	T	C	83	22,00%	1of6	18		1,89E-012
reg_DEFB	YES	chr8	7212354	G	A	74	14,00%	1of6	10		7,76E-006
reg_DEFB	YES	chr8	7212402	G	A	68	35,00%	2of6	24		1,00E-012
reg_DEFB	YES	chr8	7212441	C	G	70	27,00%	2of6	19		1,00E-012
reg_DEFB	YES	chr8	7212461	A	C	76	37,00%	2of6	28		1,00E-012
reg_DEFB	YES	chr8	7212493	G	C	78	18,00%	1of6	14		2,98E-009
reg_DEFB	YES	chr8	7212526	C	A	75	45,00%	3of6	34	rs9720736	1,00E-012
reg_DEFB	YES	chr8	7212533	C	T	75	48,00%	3of6	36		1,00E-012
reg_DEFB	YES	chr8	7212572	G	C	79	30,00%	2of6	24		1,00E-012
reg_DEFB	YES	chr8	7212693	C	G	88	24,00%	1of6	21		1,00E-012
reg_DEFB	YES	chr8	7212712	A	G	91	36,00%	2of6	33		1,00E-012
reg_DEFB	YES	chr8	7212726	G	A	93	42,00%	2of6	39		1,00E-012
reg_DEFB	YES	chr8	7212732	A	G	92	24,00%	1of6	22		1,00E-012
reg_DEFB	YES	chr8	7212764	G	T	98	22,00%	1of6	22		1,00E-012
reg_DEFB	YES	chr8	7212782	C	A	100	45,00%	3of6	45		1,00E-012
reg_DEFB	YES	chr8	7212842	G	A	114	16,00%	1of6	18		1,63E-010
reg_DEFB	YES	chr8	7212873	A	G	121	18,00%	1of6	22		1,00E-012

reg_DEFB	YES	chr8	7212877	A	G	121	49,00%	3of6	59			1,00E-012
reg_DEFB	YES	chr8	7212929	C	T	129	19,00%	1of6	25			1,00E-012
reg_DEFB	YES	chr8	7212969	G	C	127	43,00%	3of6	55			1,00E-012
reg_DEFB	YES	chr8	7212983	C	T	126	24,00%	1of6	30			1,00E-012
reg_DEFB	YES	chr8	7213048	G	T	132	42,00%	2of6	55			1,00E-012
reg_DEFB	YES	chr8	7213076	C	T	139	21,00%	1of6	29			1,00E-012
reg_DEFB	YES	chr8	7213087	C	T	139	21,00%	1of6	29			1,00E-012
reg_DEFB	YES	chr8	7213098	T	C	137	23,00%	1of6	32	rs9720878		1,00E-012
reg_DEFB	YES	chr8	7213137	A	G	125	22,00%	1of6	28			1,00E-012
reg_DEFB	YES	chr8	7213139	C	T	126	45,00%	3of6	57			1,00E-012
reg_DEFB	YES	chr8	7213178	T	C	119	16,00%	1of6	19			4,19E-011
reg_DEFB	YES	chr8	7213182	G	T	115	17,00%	1of6	20			8,76E-012
reg_DEFB	YES	chr8	7213215	G	T	106	17,00%	1of6	18			5,72E-011
reg_DEFB	YES	chr8	7213222	A	G	112	31,00%	2of6	35			1,00E-012
reg_DEFB	YES	chr8	7213227	C	G	114	33,00%	2of6	38			1,00E-012
reg_DEFB	YES	chr8	7213245	A	T	112	42,00%	2of6	47			1,00E-012
reg_DEFB	YES	chr8	7213289	G	C	104	18,00%	1of6	19	rs41394946		1,18E-011
reg_DEFB	YES	chr8	7213305	A	G	101	45,00%	3of6	45			1,00E-012
reg_DEFB	YES	chr8	7213332	C	A	99	44,00%	3of6	44			1,00E-012
reg_DEFB	YES	chr8	7213344	T	C	96	15,00%	1of6	14			4,72E-008
reg_DEFB	YES	chr8	7213364	G	A	96	17,00%	1of6	16			7,11E-010
reg_DEFB	YES	chr8	7213393	C	T	87	49,00%	3of6	43			1,00E-012
reg_DEFB	YES	chr8	7213423	G	C	81	36,00%	2of6	29			1,00E-012
reg_DEFB	YES	chr8	7213445	C	T	73	49,00%	3of6	36	rs3958829		1,00E-012
reg_DEFB	YES	chr8	7213470	A	G	66	71,00%	4of6	47	rs9314629		1,00E-012
reg_DEFB	YES	chr8	7213488	T	C	60	18,00%	1of6	11			1,15E-007
reg_DEFB	YES	chr8	7213489	A	G	59	20,00%	1of6	12			8,98E-009
reg_DEFB	YES	chr8	7213541	G	A	48	15,00%	1of6	7			1,10E-004
reg_DEFB	YES	chr8	7213575	A	C	43	47,00%	3of6	20			1,00E-012
reg_DEFB	YES	chr8	7213584	A	C	40	22,00%	1of6	9			2,58E-007
reg_DEFB	YES	chr8	7213603	T	C	34	50,00%	3of6	17			1,00E-012
reg_DEFB	YES	chr8	7213616	T	G	35	20,00%	1of6	7			1,30E-005
reg_DEFB	YES	chr8	7213622	C	A	35	20,00%	1of6	7			1,30E-005
reg_DEFB	YES	chr8	7213631	T	C	35	40,00%	2of6	14			1,00E-012
reg_DEFB	YES	chr8	7213674	A	G	34	44,00%	3of6	15			1,00E-012
reg_DEFB	YES	chr8	7213693	A	G	34	44,00%	3of6	15			1,00E-012
reg_DEFB	YES	chr8	7213713	A	T	33	42,00%	2of6	14			1,00E-012
reg_DEFB	YES	chr8	7213766	T	A	32	50,00%	3of6	16			1,00E-012
reg_DEFB	YES	chr8	7213778	C	T	33	36,00%	2of6	12			1,03E-011
reg_DEFB	YES	chr8	7213785	G	A	33	21,00%	1of6	7			8,59E-006
reg_DEFB	YES	chr8	7213790	T	C	33	12,00%	1of6	4			6,72E-003
reg_DEFB	YES	chr8	7213799	C	T	32	16,00%	1of6	5			7,72E-004
reg_DEFB	YES	chr8	7213820	A	G	36	31,00%	2of6	11			3,56E-010
reg_DEFB	YES	chr8	7213824	A	C	36	22,00%	1of6	8			1,33E-006
reg_DEFB	YES	chr8	7213845	G	A	34	41,00%	2of6	14			1,00E-012
reg_DEFB	YES	chr8	7213856	A	G	35	14,00%	1of6	5			1,17E-003
reg_DEFB	YES	chr8	7213871	G	A	35	40,00%	2of6	14		rs1456304	1,00E-012
reg_DEFB	YES	chr8	7213935	C	T	34	18,00%	1of6	6			1,14E-004
reg_DEFB	YES	chr8	7214009	C	T	31	19,00%	1of6	6			6,64E-005
reg_DEFB	YES	chr8	7214022	A	T	31	19,00%	1of6	6			6,64E-005
reg_DEFB	YES	chr8	7214034	C	G	30	53,00%	3of6	16			1,00E-012
reg_DEFB	YES	chr8	7214038	T	G	31	16,00%	1of6	5			6,64E-004
reg_DEFB	YES	chr8	7214039	C	T	31	23,00%	1of6	7		rs6997873	5,51E-006

reg_DEFB	YES	chr8	7214040	G	A	31	19,00%	1of6	6			6,64E-005
reg_DEFB	YES	chr8	7214050	T	C	29	10,00%	1of6	3			2,85E-002
reg_DEFB	YES	chr8	7214061	G	A	28	18,00%	1of6	5			4,06E-004
reg_DEFB	YES	chr8	7214102	G	A	27	19,00%	1of6	5			3,40E-004
reg_DEFB	YES	chr8	7214129	G	A	28	21,00%	1of6	6		rs35534613	3,61E-005
reg_DEFB	YES	chr8	7214143	A	C	29	55,00%	3of6	16		rs62527852	1,00E-012
reg_DEFB	YES	chr8	7214164	T	C	31	58,00%	3of6	18	rs9693481		1,00E-012
reg_DEFB	YES	chr8	7214177	C	A	32	16,00%	1of6	5			7,72E-004
reg_DEFB	YES	chr8	7214198	T	C	34	15,00%	1of6	5			1,03E-003
reg_DEFB	YES	chr8	7214212	A	G	35	14,00%	1of6	5			1,17E-003
reg_DEFB	YES	chr8	7214223	G	A	32	41,00%	2of6	13			1,00E-012
reg_DEFB	YES	chr8	7214240	A	G	35	54,00%	3of6	19	rs3989715		1,00E-012
reg_DEFB	YES	chr8	7214272	A	G	36	53,00%	3of6	19			1,00E-012
reg_DEFB	YES	chr8	7214296	G	T	37	54,00%	3of6	20			1,00E-012
reg_DEFB	YES	chr8	7214308	C	G	34	44,00%	3of6	15		rs57788671	1,00E-012
reg_DEFB	YES	chr8	7214315	A	G	35	54,00%	3of6	19			1,00E-012
reg_DEFB	YES	chr8	7214316	C	T	35	29,00%	2of6	10			4,50E-009
reg_DEFB	YES	chr8	7214344	C	T	35	11,00%	1of6	4			8,30E-003
reg_DEFB	YES	chr8	7214351	G	C	35	31,00%	2of6	11			2,52E-010
reg_DEFB	YES	chr8	7214371	C	A	36	28,00%	2of6	10		rs60424581	6,11E-009
reg_DEFB	YES	chr8	7214386	G	T	33	42,00%	2of6	14	rs3958828		1,00E-012
reg_DEFB	YES	chr8	7214405	T	A	32	56,00%	3of6	18			1,00E-012
reg_DEFB	YES	chr8	7214458	A	T	28	11,00%	1of6	3			2,60E-002
reg_DEFB	YES	chr8	7214483	G	A	25	20,00%	1of6	5			2,33E-004
reg_DEFB	YES	chr8	7214498	T	C	25	20,00%	1of6	5		rs72669900	2,33E-004
reg_DEFB	YES	chr8	7214519	T	C	28	32,00%	2of6	9			8,38E-009
reg_DEFB	YES	chr8	7214555	A	G	25	24,00%	1of6	6			1,80E-005
reg_DEFB	YES	chr8	7214560	C	T	25	12,00%	1of6	3	rs3958827		1,92E-002
reg_DEFB	YES	chr8	7214565	A	C	25	12,00%	1of6	3			1,92E-002
reg_DEFB	YES	chr8	7214578	C	T	23	22,00%	1of6	5		rs12548551	1,53E-004
reg_DEFB	YES	chr8	7214598	C	G	26	35,00%	2of6	9			3,95E-009
reg_DEFB	YES	chr8	7214642	C	T	25	32,00%	2of6	8		rs72646358	5,97E-008
reg_DEFB	YES	chr8	7214643	A	G	26	31,00%	2of6	8			8,44E-008
reg_DEFB	YES	chr8	7214648	C	T	26	31,00%	2of6	8			8,44E-008
reg_DEFB	YES	chr8	7214649	A	G	26	27,00%	2of6	7			1,52E-006
reg_DEFB	YES	chr8	7214657	T	A	26	38,00%	2of6	10		rs59964474	1,61E-010
reg_DEFB	YES	chr8	7214679	G	A	29	41,00%	2of6	12	rs3989713		1,00E-012
reg_DEFB	YES	chr8	7214684	C	A	29	31,00%	2of6	9			1,19E-008
reg_DEFB	YES	chr8	7214746	G	A	32	31,00%	2of6	10			1,68E-009
reg_DEFB	YES	chr8	7214782	C	A	32	16,00%	1of6	5			7,72E-004
reg_DEFB	YES	chr8	7214792	A	G	32	37,00%	2of6	12	rs3989712		6,54E-012
reg_DEFB	YES	chr8	7214810	A	G	30	50,00%	3of6	15	rs9720498		1,00E-012
reg_DEFB	YES	chr8	7214844	G	A	30	13,00%	1of6	4			4,76E-003
reg_DEFB	YES	chr8	7214929	G	A	28	29,00%	2of6	8			1,61E-007
reg_DEFB	YES	chr8	7214947	A	G	24	21,00%	1of6	5	rs3989711		1,90E-004
reg_DEFB	YES	chr8	7215572	C	T	25	80,00%	5of6	20	rs9774404		1,00E-012
reg_DEFB	YES	chr8	7215725	G	C	21	14,00%	1of6	3			1,19E-002
reg_DEFB	YES	chr8	7215746	G	A	21	57,00%	3of6	12	rs4840274		1,00E-012
reg_DEFB	YES	chr8	7215787	T	C	26	27,00%	2of6	7		rs7839157	1,52E-006
reg_DEFB	YES	chr8	7215791	A	T	26	58,00%	3of6	15		rs71532134	1,00E-012
reg_DEFB	YES	chr8	7216063	G	C	34	32,00%	2of6	11		rs56093011	1,76E-010
reg_DEFB	YES	chr8	7216066	A	C	35	43,00%	3of6	15		rs9772145	1,00E-012
reg_DEFB	YES	chr8	7216075	C	T	38	21,00%	1of6	8			2,07E-006

reg_DEFB	YES	chr8	7216165	G	T	43	70,00%	4of6	30	rs2719560		1,00E-012
reg_DEFB	YES	chr8	7216177	A	G	42	74,00%	4of6	31	rs2698852		1,00E-012
reg_DEFB	YES	chr8	7216193	C	A	44	32,00%	2of6	14		rs2739920	1,00E-012
reg_DEFB	YES	chr8	7216236	G	T	50	22,00%	1of6	11		rs2740655	1,55E-008
reg_DEFB	YES	chr8	7216240	G	C	50	44,00%	3of6	22		rs2739919	1,00E-012
reg_DEFB	YES	chr8	7216357	C	A	83	39,00%	2of6	32			1,00E-012
reg_DEFB	YES	chr8	7216359	T	C	84	11,00%	1of6	9			1,40E-004
reg_DEFB	YES	chr8	7216399	C	T	91	11,00%	1of6	10			4,87E-005
reg_DEFB	YES	chr8	7216407	C	G	93	11,00%	1of6	10			5,88E-005
reg_DEFB	YES	chr8	7216462	T	A	115	18,00%	1of6	21		rs62636820	1,00E-012
reg_DEFB	YES	chr8	7216464	G	T	117	10,00%	1of6	12			1,79E-005
reg_DEFB	YES	chr8	7216565	A	C	150	52,00%	3of6	78	rs2739916		1,00E-012
reg_DEFB	YES	chr8	7216666	A	T	169	11,00%	1of6	19		rs2461004	1,69E-008
reg_DEFB	YES	chr8	7216730	C	A	180	10,00%	1of6	18			2,38E-007
reg_DEFB	YES	chr8	7216748	T	A	189	10,00%	1of6	19			1,03E-007
reg_DEFB	YES	chr8	7216859	T	C	222	11,00%	1of6	24			5,38E-010
reg_DEFB	YES	chr8	7217037	C	A	232	13,00%	1of6	30	rs3958825		1,00E-012
reg_DEFB	YES	chr8	7217057	T	C	219	12,00%	1of6	26			2,08E-011
reg_DEFB	YES	chr8	7217109	G	C	196	31,00%	2of6	61	rs2740653		1,00E-012
reg_DEFB	YES	chr8	7217116	G	A	196	13,00%	1of6	25			9,86E-012
reg_DEFB	YES	chr8	7217208	A	G	181	18,00%	1of6	33			1,00E-012
reg_DEFB	YES	chr8	7217227	T	C	173	16,00%	1of6	28			1,00E-012
reg_DEFB	YES	chr8	7217283	T	C	146	14,00%	1of6	20			2,18E-010
reg_DEFB	YES	chr8	7217293	C	T	141	11,00%	1of6	16			1,94E-007
reg_DEFB	YES	chr8	7217310	A	G	166	26,00%	2of6	43	rs2739913		1,00E-012
reg_DEFB	YES	chr8	7217386	C	T	199	11,00%	1of6	22			1,81E-009
reg_DEFB	YES	chr8	7217396	A	G	203	20,00%	1of6	41			1,00E-012
reg_DEFB	YES	chr8	7217411	C	T	213	20,00%	1of6	43	rs2739912		1,00E-012
reg_DEFB	YES	chr8	7217505	G	T	260	16,00%	1of6	42			1,00E-012
reg_DEFB	YES	chr8	7217544	T	A	268	19,00%	1of6	51	rs2740651		1,00E-012
reg_DEFB	YES	chr8	7217623	G	C	251	21,00%	1of6	53			1,00E-012
reg_DEFB	YES	chr8	7217638	C	A	247	11,00%	1of6	27			4,06E-011
reg_DEFB	YES	chr8	7217667	T	A	246	13,00%	1of6	32			1,00E-012
reg_DEFB	YES	chr8	7217748	T	C	232	13,00%	1of6	30	rs2739911		1,00E-012
reg_DEFB	YES	chr8	7217892	T	C	201	11,00%	1of6	22			2,18E-009
reg_DEFB	YES	chr8	7217922	G	A	209	11,00%	1of6	23			8,57E-010
reg_DEFB	YES	chr8	7217957	G	T	208	12,00%	1of6	25			2,62E-011
reg_DEFB	YES	chr8	7218003	C	G	200	10,00%	1of6	20			5,25E-008
reg_DEFB	YES	chr8	7218034	T	C	184	17,00%	1of6	31			1,00E-012
reg_DEFB	YES	chr8	7218052	C	A	175	11,00%	1of6	19			2,99E-008
reg_DEFB	YES	chr8	7218187	G	A	116	78,00%	5of6	90	rs3877968		1,00E-012
reg_DEFB	YES	chr8	7218454	G	A	90	27,00%	2of6	24			1,00E-012
reg_DEFB	YES	chr8	7218825	G	T	103	16,00%	1of6	16	rs2719558		2,07E-009
reg_DEFB	YES	chr8	7218845	T	C	104	73,00%	4of6	76			1,00E-012
reg_DEFB	YES	chr8	7218861	A	G	107	12,00%	1of6	13		rs2719557	1,21E-006
reg_DEFB	YES	chr8	7218900	C	T	108	12,00%	1of6	13		rs2739910	1,35E-006
reg_DEFB	YES	chr8	7218970	C	G	113	13,00%	1of6	15		rs2951096	5,76E-008
reg_DEFB	YES	chr8	7218973	T	C	111	13,00%	1of6	14		rs2954054	3,00E-007
reg_DEFB	YES	chr8	7219008	A	G	121	12,00%	1of6	15			1,44E-007
reg_DEFB	YES	chr8	7219078	T	G	141	14,00%	1of6	20			1,20E-010
reg_DEFB	YES	chr8	7219176	C	T	163	15,00%	1of6	24			3,05E-012
reg_DEFB	YES	chr8	7219186	T	G	166	14,00%	1of6	23			1,14E-011
reg_DEFB	YES	chr8	7219191	A	G	167	75,00%	4of6	125		rs71509104	1,00E-012

reg_DEFB	YES	chr8	7219218	T	C	170	77,00%	5of6	131			1,00E-012
reg_DEFB	YES	chr8	7219236	G	C	170	14,00%	1of6	24			7,96E-012
reg_DEFB	YES	chr8	7219251	T	C	167	10,00%	1of6	17			3,95E-007
reg_DEFB	YES	chr8	7219280	T	C	175	17,00%	1of6	30			1,00E-012
reg_DEFB	YES	chr8	7219346	C	A	163	14,00%	1of6	23			7,68E-012
reg_DEFB	YES	chr8	7219348	C	A	164	26,00%	2of6	43			1,00E-012
reg_DEFB	YES	chr8	7219375	T	C	171	26,00%	2of6	44			1,00E-012
reg_DEFB	YES	chr8	7219429	G	T	170	22,00%	1of6	37			1,00E-012
reg_DEFB	YES	chr8	7219459	C	A	156	24,00%	1of6	37			1,00E-012
reg_DEFB	YES	chr8	7219479	A	G	151	15,00%	1of6	23			4,21E-012
reg_DEFB	YES	chr8	7219497	C	G	141	21,00%	1of6	30			1,00E-012
reg_DEFB	YES	chr8	7219501	A	T	140	13,00%	1of6	18			4,76E-009
reg_DEFB	YES	chr8	7219548	C	A	128	12,00%	1of6	15			3,04E-007
reg_DEFB	YES	chr8	7219555	G	A	125	13,00%	1of6	16			3,56E-008
reg_DEFB	YES	chr8	7219581	G	A	119	18,00%	1of6	21			1,91E-012
reg_DEFB	YES	chr8	7219618	C	T	118	80,00%	5of6	94	rs34236860		1,00E-012
reg_DEFB	YES	chr8	7219641	G	A	118	13,00%	1of6	15			1,03E-007
reg_DEFB	YES	chr8	7219667	T	C	112	18,00%	1of6	20			5,18E-012
reg_DEFB	YES	chr8	7219722	T	C	106	11,00%	1of6	12			6,50E-006
reg_DEFB	YES	chr8	7219728	A	T	109	17,00%	1of6	19			1,04E-011
reg_DEFB	YES	chr8	7219739	G	A	108	17,00%	1of6	18			6,44E-011
reg_DEFB	YES	chr8	7219745	T	A	107	12,00%	1of6	13			1,21E-006
reg_DEFB	YES	chr8	7219750	A	G	107	12,00%	1of6	13			1,21E-006
reg_DEFB	YES	chr8	7219778	T	G	104	16,00%	1of6	17			2,97E-010
reg_DEFB	YES	chr8	7219802	C	T	102	21,00%	1of6	21			1,00E-012
reg_DEFB	YES	chr8	7219968	C	T	97	29,00%	2of6	28			1,00E-012
reg_DEFB	YES	chr8	7219996	T	C	106	14,00%	1of6	15			2,40E-008
reg_DEFB	YES	chr8	7220020	G	A	96	42,00%	2of6	40		rs2740649	1,00E-012
reg_DEFB	YES	chr8	7220066	G	A	89	37,00%	2of6	33	rs3959001		1,00E-012
reg_DEFB	YES	chr8	7220067	C	T	89	15,00%	1of6	13			1,38E-007
reg_DEFB	YES	chr8	7220069	G	A	88	16,00%	1of6	14			1,51E-008
reg_DEFB	YES	chr8	7220079	G	A	84	69,00%	4of6	58		rs34293419	1,00E-012
reg_DEFB	YES	chr8	7220082	C	T	86	15,00%	1of6	13			9,13E-008
reg_DEFB	YES	chr8	7220087	A	G	85	29,00%	2of6	25			1,00E-012
reg_DEFB	YES	chr8	7220132	A	T	91	20,00%	1of6	18		rs62493693	1,03E-011
reg_DEFB	YES	chr8	7220184	T	C	90	19,00%	1of6	17			3,25E-011
reg_DEFB	YES	chr8	7220216	T	G	93	23,00%	1of6	21		rs2139763	1,00E-012
reg_DEFB	YES	chr8	7220227	T	C	93	23,00%	1of6	21		rs2139762	1,00E-012
reg_DEFB	YES	chr8	7220232	C	A	92	16,00%	1of6	15			3,29E-009
reg_DEFB	YES	chr8	7220265	C	T	103	75,00%	4of6	77	rs34967312		1,00E-012
reg_DEFB	YES	chr8	7220273	G	A	105	18,00%	1of6	19			1,41E-011
reg_DEFB	YES	chr8	7220281	G	A	104	17,00%	1of6	18		rs62483102	4,09E-011
reg_DEFB	YES	chr8	7220288	A	G	111	17,00%	1of6	19			1,46E-011
reg_DEFB	YES	chr8	7220306	A	C	121	17,00%	1of6	21			2,71E-012
reg_DEFB	YES	chr8	7220320	G	A	129	18,00%	1of6	23			1,00E-012
reg_DEFB	YES	chr8	7220342	T	G	141	16,00%	1of6	23			1,00E-012
reg_DEFB	YES	chr8	7220424	G	A	175	13,00%	1of6	23			2,53E-011
reg_DEFB	YES	chr8	7220499	T	G	192	69,00%	4of6	132	rs34788125		1,00E-012
reg_DEFB	YES	chr8	7220520	C	T	192	13,00%	1of6	25			6,15E-012
reg_DEFB	YES	chr8	7220529	G	T	199	35,00%	2of6	70	rs35957899		1,00E-012
reg_DEFB	YES	chr8	7220593	C	G	231	14,00%	1of6	32			1,00E-012
reg_DEFB	YES	chr8	7220619	T	C	236	62,00%	4of6	146	rs4621826		1,00E-012
reg_DEFB	YES	chr8	7220646	T	C	235	14,00%	1of6	33			1,00E-012

reg_DEFB	YES	chr8	7220663	A	T	234	15,00%	1of6	35				1,00E-012
reg_DEFB	YES	chr8	7220811	A	G	234	16,00%	1of6	37				1,00E-012
reg_DEFB	YES	chr8	7220980	T	C	201	72,00%	4of6	145	rs4620307	rs36048429		1,00E-012
reg_DEFB	YES	chr8	7220984	G	A	197	41,00%	2of6	81	rs34205780			1,00E-012
reg_DEFB	YES	chr8	7221052	T	C	177	10,00%	1of6	18				1,85E-007
reg_DEFB	YES	chr8	7221074	A	C	186	10,00%	1of6	19				7,97E-008
reg_DEFB	YES	chr8	7221131	C	T	176	11,00%	1of6	19				3,28E-008
reg_DEFB	YES	chr8	7221184	G	A	153	25,00%	1of6	38				1,00E-012
reg_DEFB	YES	chr8	7221196	T	C	151	66,00%	4of6	100			rs2739907	1,00E-012
reg_DEFB	YES	chr8	7221235	G	T	141	33,00%	2of6	47				1,00E-012
reg_DEFB	YES	chr8	7221273	C	T	138	73,00%	4of6	101	rs35737531			1,00E-012
reg_DEFB	YES	chr8	7221351	C	T	159	17,00%	1of6	27			rs2463975	1,00E-012
reg_DEFB	YES	chr8	7221359	C	T	164	26,00%	2of6	43			rs2463974	1,00E-012
reg_DEFB	YES	chr8	7221372	A	G	167	21,00%	1of6	35	rs2466108			1,00E-012
reg_DEFB	YES	chr8	7221409	G	A	177	20,00%	1of6	35				1,00E-012
reg_DEFB	YES	chr8	7221417	T	A	176	19,00%	1of6	33			rs3867441	1,00E-012
reg_DEFB	YES	chr8	7221438	G	A	176	24,00%	1of6	42	rs10112635			1,00E-012
reg_DEFB	YES	chr8	7221460	T	A	180	32,00%	2of6	58	rs2698891			1,00E-012
reg_DEFB	YES	chr8	7221461	C	G	183	14,00%	1of6	26	rs2698891			1,00E-012
reg_DEFB	YES	chr8	7221470	G	A	184	68,00%	4of6	125	rs2739906	rs34051729		1,00E-012
reg_DEFB	YES	chr8	7221507	G	A	187	71,00%	4of6	133	rs4118276			1,00E-012
reg_DEFB	YES	chr8	7221611	G	T	201	13,00%	1of6	26			rs28409357	8,02E-012
reg_DEFB	YES	chr8	7221622	G	A	204	26,00%	2of6	53	rs2719550			1,00E-012
reg_DEFB	YES	chr8	7221627	G	A	207	11,00%	1of6	23				7,09E-010
reg_DEFB	YES	chr8	7221643	G	T	195	11,00%	1of6	21				6,67E-009
reg_DEFB	YES	chr8	7221650	C	A	193	10,00%	1of6	19				1,43E-007
reg_DEFB	YES	chr8	7221656	A	C	194	13,00%	1of6	25				7,80E-012
reg_DEFB	YES	chr8	7221664	G	A	187	27,00%	2of6	50				1,00E-012
reg_DEFB	YES	chr8	7221673	A	G	186	27,00%	2of6	50	rs2719549	rs2698890		1,00E-012
reg_DEFB	YES	chr8	7221674	T	C	185	28,00%	2of6	52	rs2698890	rs34808763		1,00E-012
reg_DEFB	YES	chr8	7221734	G	A	167	13,00%	1of6	22				6,47E-011
reg_DEFB	YES	chr8	7221771	C	T	151	26,00%	2of6	39	rs2739904			1,00E-012
reg_DEFB	YES	chr8	7221814	T	A	133	36,00%	2of6	48				1,00E-012
reg_DEFB	YES	chr8	7222272	A	G	89	11,00%	1of6	10				4,02E-005
reg_DEFB	YES	chr8	7222307	G	A	84	45,00%	3of6	38	rs2739903			1,00E-012
reg_DEFB	YES	chr8	7222348	A	T	78	21,00%	1of6	16	rs2719547			3,21E-011
reg_DEFB	YES	chr8	7222419	C	A	66	44,00%	3of6	29	rs2739902			1,00E-012
reg_DEFB	YES	chr8	7222502	G	T	47	81,00%	5of6	38	rs4118271			1,00E-012
reg_DEFB	YES	chr8	7222663	G	C	25	44,00%	3of6	11	rs2698889			5,49E-012
reg_DEFB	YES	chr8	7222754	C	T	22	14,00%	1of6	3				1,35E-002
reg_DEFB	YES	chr8	7222802	T	C	18	33,00%	2of6	6				2,17E-006
reg_DEFB	YES	chr8	7222843	A	G	15	80,00%	5of6	12			rs73356401	1,00E-012
reg_DEFB	YES	chr8	7222923	T	C	28	25,00%	1of6	7				2,63E-006
reg_DEFB	YES	chr8	7222958	C	T	38	18,00%	1of6	7				2,30E-005
reg_DEFB	YES	chr8	7222992	A	T	48	10,00%	1of6	5	rs41347745			4,85E-003
reg_DEFB	YES	chr8	7222997	A	C	55	18,00%	1of6	10				4,70E-007
reg_DEFB	YES	chr8	7223016	C	A	60	28,00%	2of6	17			rs2719544	1,00E-012
reg_DEFB	YES	chr8	7223022	A	C	61	13,00%	1of6	8				7,78E-005
reg_DEFB	YES	chr8	7223030	C	T	62	16,00%	1of6	10				1,49E-006
reg_DEFB	YES	chr8	7223044	A	C	65	11,00%	1of6	7				7,38E-004
reg_DEFB	YES	chr8	7223054	C	A	69	17,00%	1of6	12				5,71E-008
reg_DEFB	YES	chr8	7223063	C	T	73	15,00%	1of6	11	rs2719543			9,11E-007
reg_DEFB	YES	chr8	7223096	A	G	83	12,00%	1of6	10				2,18E-005

reg_DEFB	YES	chr8	7223105	C	T	87	18,00%	1of6	16			1,57E-010
reg_DEFB	YES	chr8	7223131	G	A	98	29,00%	2of6	28	rs2719542		1,00E-012
reg_DEFB	YES	chr8	7223250	T	C	122	29,00%	2of6	35	rs2719541		1,00E-012
reg_DEFB	YES	chr8	7223379	T	C	147	20,00%	1of6	29	rs2719540		1,00E-012
reg_DEFB	YES	chr8	7223579	G	A	148	12,00%	1of6	18	rs2698888		1,16E-008
reg_DEFB	YES	chr8	7223613	A	C	139	12,00%	1of6	17			2,68E-008
reg_DEFB	YES	chr8	7223755	G	T	116	15,00%	1of6	17		rs56354845	1,66E-009
reg_DEFB	YES	chr8	7223760	G	C	117	48,00%	3of6	56	rs2719539		1,00E-012
reg_DEFB	YES	chr8	7223811	C	A	116	45,00%	3of6	52	rs2719538		1,00E-012
reg_DEFB	YES	chr8	7224176	G	A	128	23,00%	1of6	29	rs2719537		1,00E-012
reg_DEFB	YES	chr8	7224181	T	C	129	23,00%	1of6	30		rs2739897	1,00E-012
reg_DEFB	YES	chr8	7224262	T	A	121	14,00%	1of6	17			3,21E-009
reg_DEFB	YES	chr8	7224337	C	T	112	61,00%	4of6	68	rs9720658		1,00E-012
reg_DEFB	YES	chr8	7224692	G	A	66	12,00%	1of6	8		rs2740646	1,37E-004
reg_DEFB	YES	chr8	7224717	G	T	66	12,00%	1of6	8	rs2719533		1,37E-004
reg_DEFB	YES	chr8	7224859	C	A	38	11,00%	1of6	4	rs2719532		1,11E-002
reg_DEFB	YES	chr8	7224910	T	G	32	59,00%	4of6	19	rs10087379		1,00E-012
reg_DEFB	YES	chr8	7225003	A	T	36	69,00%	4of6	25	rs28624344		1,00E-012
reg_DEFB	YES	chr8	7225089	C	G	33	36,00%	2of6	12			1,03E-011
reg_DEFB	YES	chr8	7225112	G	A	30	60,00%	4of6	18	rs4840741		1,00E-012
reg_DEFB	YES	chr8	7225257	G	C	53	13,00%	1of6	7	rs2719530		2,08E-004
reg_DEFB	YES	chr8	7225316	A	C	68	28,00%	2of6	19	rs9720218		1,00E-012
reg_DEFB	YES	chr8	7225374	A	G	76	82,00%	5of6	62	rs4840742		1,00E-012
reg_DEFB	YES	chr8	7225426	T	C	78	41,00%	2of6	32	rs9720354		1,00E-012
reg_DEFB	YES	chr8	7225445	G	T	83	22,00%	1of6	18	rs9720806		1,89E-012
reg_DEFB	YES	chr8	7225457	A	G	84	23,00%	1of6	19			1,00E-012
reg_DEFB	YES	chr8	7225462	T	C	85	22,00%	1of6	19			1,00E-012
reg_DEFB	YES	chr8	7225473	C	A	89	11,00%	1of6	10			4,02E-005
reg_DEFB	YES	chr8	7225488	G	A	87	11,00%	1of6	10			3,30E-005
reg_DEFB	YES	chr8	7225515	A	G	90	11,00%	1of6	10			4,43E-005
reg_DEFB	YES	chr8	7225527	T	C	91	11,00%	1of6	10			4,87E-005
reg_DEFB	YES	chr8	7225533	T	C	91	11,00%	1of6	10			4,87E-005
reg_DEFB	YES	chr8	7225561	A	C	93	11,00%	1of6	10			5,88E-005
reg_DEFB	YES	chr8	7225563	G	A	93	24,00%	1of6	22			1,00E-012
reg_DEFB	YES	chr8	7225570	G	C	95	23,00%	1of6	22			1,00E-012
reg_DEFB	YES	chr8	7225582	C	A	94	14,00%	1of6	13			2,66E-007
reg_DEFB	YES	chr8	7225613	C	T	83	14,00%	1of6	12			4,66E-007
reg_DEFB	YES	chr8	7225617	G	C	82	15,00%	1of6	12			4,07E-007
reg_DEFB	YES	chr8	7225642	G	T	76	24,00%	1of6	18			1,00E-012
reg_DEFB	YES	chr8	7225690	G	A	59	46,00%	3of6	27	rs2740645		1,00E-012
reg_DEFB	YES	chr8	7225701	T	C	52	23,00%	1of6	12			1,94E-009
reg_DEFB	YES	chr8	7225735	C	T	39	18,00%	1of6	7			2,74E-005
reg_DEFB	YES	chr8	7225750	A	G	34	12,00%	1of6	4			7,48E-003
reg_DEFB		chr8	7225885	T	C	45	64,00%	4of6	29			1,00E-012
reg_DEFB		chr8	7226233	T	C	117	19,00%	1of6	22	rs2719528		1,00E-012
reg_DEFB		chr8	7226320	A	G	116	37,00%	2of6	43	rs2740644		1,00E-012
reg_DEFB		chr8	7226330	C	T	117	38,00%	2of6	44	rs4592060		1,00E-012
reg_DEFB		chr8	7226617	C	G	75	45,00%	3of6	34	rs2719527		1,00E-012
reg_DEFB		chr8	7226633	C	G	70	36,00%	2of6	25		rs4610778	1,00E-012
reg_DEFB		chr8	7226645	T	C	71	51,00%	3of6	36		rs2698882	1,00E-012
reg_DEFB		chr8	7226650	T	G	71	99,00%	6of6	70		rs62636823	1,00E-012
reg_DEFB		chr8	7226864	C	A	78	44,00%	3of6	34	rs2740643		1,00E-012
reg_DEFB		chr8	7227149	T	G	131	50,00%	3of6	66		rs2739892	1,00E-012

reg_DEFB	chr8	7227202	C	G	117	79,00%	5of6	92	rs4110303			1,00E-012
reg_DEFB	chr8	7227284	A	C	120	29,00%	2of6	35			rs71511231	1,00E-012
reg_DEFB	chr8	7227393	T	C	115	47,00%	3of6	54			rs58068279	1,00E-012
reg_DEFB	chr8	7227554	G	A	114	44,00%	3of6	50			rs60497429	1,00E-012
reg_DEFB	chr8	7227642	C	T	95	84,00%	5of6	80	rs3915363			1,00E-012
reg_DEFB	chr8	7227652	C	G	95	45,00%	3of6	43	rs2719524			1,00E-012
reg_DEFB	chr8	7227677	C	A	90	44,00%	3of6	40	rs2719523			1,00E-012
reg_DEFB	chr8	7227756	G	C	72	47,00%	3of6	34			rs5004606	1,00E-012
reg_DEFB	chr8	7227784	C	T	73	45,00%	3of6	33			rs4096974	1,00E-012
reg_DEFB	chr8	7227789	T	C	69	43,00%	3of6	30			rs3915362	1,00E-012
reg_DEFB	chr8	7227922	A	T	53	70,00%	4of6	37	rs28413583	rs5004605		1,00E-012
reg_DEFB	chr8	7227939	C	T	48	23,00%	1of6	11	rs2698880			9,81E-009
reg_DEFB	chr8	7228194	T	C	64	52,00%	3of6	33	rs2719522			1,00E-012
reg_DEFB	chr8	7228313	T	C	85	40,00%	2of6	34	rs2719521			1,00E-012
reg_DEFB	chr8	7228345	G	A	85	44,00%	3of6	37	rs2719520			1,00E-012
reg_DEFB	chr8	7228356	A	G	83	47,00%	3of6	39			rs2739890	1,00E-012
reg_DEFB	chr8	7228410	G	A	82	15,00%	1of6	12			rs5000920	4,07E-007
reg_DEFB	chr8	7228430	A	C	82	44,00%	3of6	36	rs2719519			1,00E-012
reg_DEFB	chr8	7228471	A	G	88	40,00%	2of6	35				1,00E-012
reg_DEFB	chr8	7228505	T	C	90	39,00%	2of6	35				1,00E-012
reg_DEFB	chr8	7228522	G	C	87	39,00%	2of6	34	rs2466107			1,00E-012
reg_DEFB	chr8	7228560	A	G	88	41,00%	2of6	36			rs2463972	1,00E-012
reg_DEFB	chr8	7228802	T	G	83	11,00%	1of6	9			rs2719518	1,28E-004
reg_DEFB	chr8	7229225	A	G	101	18,00%	1of6	18			rs2739886	2,43E-011
reg_DEFB	chr8	7229247	G	A	104	44,00%	3of6	46			rs2719517	1,00E-012
reg_DEFB	chr8	7229277	A	G	107	42,00%	2of6	45			rs2739885	1,00E-012
reg_DEFB	chr8	7229410	C	T	100	33,00%	2of6	33			rs71236244	1,00E-012
reg_DEFB	chr8	7229511	A	G	91	30,00%	2of6	27			rs2739883	1,00E-012
reg_DEFB	chr8	7229783	G	A	116	10,00%	1of6	12			rs2698877	1,64E-005
reg_DEFB	chr8	7229789	T	C	116	41,00%	2of6	48	rs2719516			1,00E-012
reg_DEFB	chr8	7230035	C	T	85	44,00%	3of6	37	rs2739882			1,00E-012
reg_DEFB	chr8	7230147	C	T	61	43,00%	3of6	26	rs2739881			1,00E-012
reg_DEFB	chr8	7230182	T	C	65	18,00%	1of6	12			rs3898684	2,84E-008
reg_DEFB	chr8	7230346	C	A	56	14,00%	1of6	8				4,15E-005
reg_DEFB	chr8	7230397	A	C	49	33,00%	2of6	16	rs2698876			1,00E-012
reg_DEFB	chr8	7230572	G	A	51	12,00%	1of6	6				1,10E-003
reg_DEFB	chr8	7230646	C	G	50	80,00%	5of6	40			rs4295681	1,00E-012
reg_DEFB	chr8	7230690	T	G	50	14,00%	1of6	7			rs62636824	1,43E-004
reg_DEFB	chr8	7230699	T	C	50	56,00%	3of6	28			rs2719514	1,00E-012
reg_DEFB	chr8	7230752	G	A	48	27,00%	2of6	13			rs71511233	4,99E-011
reg_DEFB	chr8	7231074	G	A	52	38,00%	2of6	20	rs2740639			1,00E-012
reg_DEFB	chr8	7231137	C	A	53	40,00%	2of6	21	rs2740638			1,00E-012
reg_DEFB	chr8	7231158	G	A	53	17,00%	1of6	9	rs2698873			3,19E-006
reg_DEFB	chr8	7231435	G	A	97	52,00%	3of6	50			rs71511234	1,00E-012
reg_DEFB	chr8	7231456	G	A	103	28,00%	2of6	29	rs2739878			1,00E-012
reg_DEFB	chr8	7231537	C	T	108	36,00%	2of6	39	rs2698872			1,00E-012
reg_DEFB	chr8	7231549	C	G	110	35,00%	2of6	39	rs2719513			1,00E-012
reg_DEFB	chr8	7231781	A	G	107	40,00%	2of6	43	rs2739877			1,00E-012
reg_DEFB	chr8	7232001	C	T	116	46,00%	3of6	53	rs2698870			1,00E-012
reg_DEFB	chr8	7232162	G	A	102	48,00%	3of6	49				1,00E-012
reg_DEFB	chr8	7232277	G	T	92	14,00%	1of6	13				2,06E-007
reg_DEFB	chr8	7232299	C	T	98	48,00%	3of6	47	rs2719511			1,00E-012
reg_DEFB	chr8	7232309	G	T	99	47,00%	3of6	47				1,00E-012

reg_DEFB	chr8	7232402	T	G	114	32,00%	2of6	36		rs2739876	1,00E-012
reg_DEFB	chr8	7232411	T	G	119	49,00%	3of6	58	rs2466106		1,00E-012
reg_DEFB	chr8	7232542	A	G	113	20,00%	1of6	23		rs2739875	1,00E-012
reg_DEFB	chr8	7232595	C	T	111	50,00%	3of6	56	rs2739874		1,00E-012
reg_DEFB	chr8	7232643	A	T	110	80,00%	5of6	88	rs3915354		1,00E-012
reg_DEFB	chr8	7232788	A	G	90	33,00%	2of6	30	rs2698867		1,00E-012
reg_DEFB	chr8	7232919	G	A	91	16,00%	1of6	15			2,81E-009
reg_DEFB	chr8	7232985	C	G	98	51,00%	3of6	50		rs2739873	1,00E-012
reg_DEFB	chr8	7233075	T	C	108	51,00%	3of6	55		rs2740636	1,00E-012
reg_DEFB	chr8	7233215	G	A	127	15,00%	1of6	19		rs2740635	1,35E-010
reg_DEFB	chr8	7233270	T	C	133	14,00%	1of6	19		rs2698866	2,93E-010
reg_DEFB	chr8	7233352	T	C	120	48,00%	3of6	58		rs2740634	1,00E-012
reg_DEFB	chr8	7233515	G	A	71	45,00%	3of6	32	rs2740633		1,00E-012
reg_DEFB	chr8	7233678	A	T	51	14,00%	1of6	7		rs2739872	1,62E-004
reg_DEFB	chr8	7233706	A	G	53	43,00%	3of6	23	rs2739871		1,00E-012
reg_DEFB	chr8	7233725	C	T	58	33,00%	2of6	19	rs2740632		1,00E-012
reg_DEFB	chr8	7233768	G	C	57	40,00%	2of6	23	rs2740631		1,00E-012
reg_DEFB	chr8	7233949	C	G	85	13,00%	1of6	11		rs2739870	4,24E-006
reg_DEFB	chr8	7234062	A	C	93	45,00%	3of6	42	rs2719510		1,00E-012
reg_DEFB	chr8	7234084	G	T	97	43,00%	3of6	42	rs28544205		1,00E-012
reg_DEFB	chr8	7234115	A	G	107	40,00%	2of6	43		rs2739868	1,00E-012
reg_DEFB	chr8	7234191	C	T	123	42,00%	2of6	52		rs2739867	1,00E-012
reg_DEFB	chr8	7234287	C	T	134	43,00%	3of6	58		rs2739866	1,00E-012
reg_DEFB	chr8	7234433	A	C	131	46,00%	3of6	60		rs2698865	1,00E-012
reg_DEFB	chr8	7234521	A	T	119	48,00%	3of6	57		rs2739865	1,00E-012
reg_DEFB	chr8	7234552	T	C	110	53,00%	3of6	58		rs2740630	1,00E-012
reg_DEFB	chr8	7234557	T	C	110	54,00%	3of6	59		rs2740629	1,00E-012
reg_DEFB	chr8	7234590	G	A	100	53,00%	3of6	53		rs2740628	1,00E-012
reg_DEFB	chr8	7234600	G	A	98	12,00%	1of6	12		rs2740627	2,84E-006
reg_DEFB	chr8	7234601	C	T	96	24,00%	1of6	23			1,00E-012
reg_DEFB	chr8	7234948	A	T	89	16,00%	1of6	14			1,75E-008
reg_DEFB	chr8	7234989	C	T	84	52,00%	3of6	44		rs2698864	1,00E-012
reg_DEFB	chr8	7235018	G	A	87	54,00%	3of6	47		rs2740626	1,00E-012
reg_DEFB	chr8	7235095	G	A	98	44,00%	3of6	43		rs4437677	1,00E-012
reg_DEFB	chr8	7235113	T	A	103	49,00%	3of6	50		rs4439153	1,00E-012
reg_DEFB	chr8	7235127	A	G	106	48,00%	3of6	51		rs4633080	1,00E-012
reg_DEFB	chr8	7235144	G	A	114	47,00%	3of6	54		rs4545129	1,00E-012
reg_DEFB	chr8	7235204	C	T	127	47,00%	3of6	60		rs4623450	1,00E-012
reg_DEFB	chr8	7235255	G	A	135	47,00%	3of6	63		rs4545128	1,00E-012
reg_DEFB	chr8	7235443	G	C	144	98,00%	6of6	141	rs9720375		1,00E-012
reg_DEFB	chr8	7235591	C	G	136	44,00%	3of6	60	rs2740625		1,00E-012
reg_DEFB	chr8	7235889	C	G	83	13,00%	1of6	11	rs2698951		3,35E-006
reg_DEFB	chr8	7235967	G	T	62	34,00%	2of6	21	rs2719506		1,00E-012
reg_DEFB	chr8	7236007	T	C	48	10,00%	1of6	5			4,85E-003
reg_DEFB	chr8	7236029	G	C	45	36,00%	2of6	16		rs2740624	1,00E-012
reg_DEFB	chr8	7236128	G	T	34	32,00%	2of6	11	rs2719502		1,76E-010
reg_DEFB	chr8	7236404	C	G	46	37,00%	2of6	17		rs3958986	1,00E-012
reg_DEFB	chr8	7236418	C	T	47	36,00%	2of6	17		rs3988877	1,00E-012
reg_DEFB	chr8	7236506	A	G	61	38,00%	2of6	23		rs2719494	1,00E-012
reg_DEFB	chr8	7236551	T	C	61	38,00%	2of6	23	rs28459499		1,00E-012
reg_DEFB	chr8	7236608	C	T	67	34,00%	2of6	23		rs2740165	1,00E-012
reg_DEFB	chr8	7236670	C	A	62	31,00%	2of6	19		rs62636825	1,00E-012
reg_DEFB	chr8	7236701	C	T	69	39,00%	2of6	27	rs2740164		1,00E-012

reg_DEFB	chr8	7236753	G	A	78	44,00%	3of6	34	rs2740623		1,00E-012
reg_DEFB	chr8	7236764	T	C	77	30,00%	2of6	23		rs2740163	1,00E-012
reg_DEFB	chr8	7236897	G	C	72	25,00%	1of6	18		rs72494251	1,00E-012
reg_DEFB	chr8	7237003	A	G	67	43,00%	3of6	29		rs2740622	1,00E-012
reg_DEFB	chr8	7237030	G	A	70	11,00%	1of6	8			2,08E-004
reg_DEFB	chr8	7237110	G	A	71	46,00%	3of6	33		rs2740621	1,00E-012
reg_DEFB	chr8	7237169	C	T	79	46,00%	3of6	36		rs2740162	1,00E-012
reg_DEFB	chr8	7237229	G	A	73	42,00%	2of6	31		rs2740620	1,00E-012
reg_DEFB	chr8	7237268	C	T	71	41,00%	2of6	29		rs2740161	1,00E-012
reg_DEFB	chr8	7237506	C	T	53	41,00%	2of6	22	rs2740160		1,00E-012
reg_DEFB	chr8	7237549	T	C	47	36,00%	2of6	17		rs72626631	1,00E-012
reg_DEFB	chr8	7237560	A	G	45	44,00%	3of6	20	rs2740618		1,00E-012
reg_DEFB	chr8	7237571	A	T	44	23,00%	1of6	10		rs55939595	5,02E-008
reg_DEFB	chr8	7237869	T	C	48	48,00%	3of6	23		rs2740616	1,00E-012
reg_DEFB	chr8	7237961	T	C	56	37,00%	2of6	21		rs2740615	1,00E-012
reg_DEFB	chr8	7238229	G	C	45	18,00%	1of6	8	rs2719466		7,89E-006
reg_DEFB	chr8	7238361	A	G	31	42,00%	2of6	13	rs2719465		1,00E-012
reg_DEFB	chr8	7238365	A	G	31	45,00%	3of6	14	rs9720329		1,00E-012
reg_DEFB	chr8	7238371	A	T	32	44,00%	3of6	14		rs2698958	1,00E-012
reg_DEFB	chr8	7238984	C	T	34	15,00%	1of6	5			1,03E-003
reg_DEFB	chr8	7239140	C	T	35	51,00%	3of6	18		rs2740159	1,00E-012
reg_DEFB	chr8	7239175	C	T	36	19,00%	1of6	7		rs2698959	1,58E-005
reg_DEFB	chr8	7239239	T	C	40	37,00%	2of6	15		rs2698960	1,00E-012
reg_DEFB	chr8	7239336	T	C	46	24,00%	1of6	11			6,04E-009
reg_DEFB	chr8	7239401	C	G	53	58,00%	3of6	31	rs2740613		1,00E-012
reg_DEFB	chr8	7239431	A	G	63	56,00%	3of6	35	rs2719454		1,00E-012
reg_DEFB	chr8	7239620	T	C	102	13,00%	1of6	13	rs2740612		6,97E-007
reg_DEFB	chr8	7239710	T	G	128	49,00%	3of6	63	rs2740611		1,00E-012
reg_DEFB	chr8	7240036	G	A	93	29,00%	2of6	27		rs2740610	1,00E-012
reg_DEFB	chr8	7240395	C	A	51	96,00%	6of6	49	rs13260072		1,00E-012
reg_DEFB	chr8	7240612	C	A	70	47,00%	3of6	33	rs2740609		1,00E-012
reg_DEFB	chr8	7240639	C	G	67	12,00%	1of6	8		rs2698962	1,52E-004
reg_DEFB	chr8	7240641	G	C	67	12,00%	1of6	8		rs2719435	1,52E-004
reg_DEFB	chr8	7240705	T	C	74	14,00%	1of6	10	rs7005466		7,76E-006
reg_DEFB	chr8	7240763	G	C	70	13,00%	1of6	9		rs2719434	3,30E-005
reg_DEFB	chr8	7240831	C	T	56	70,00%	4of6	39		rs71250725	1,00E-012
reg_DEFB	chr8	7240856	C	A	54	46,00%	3of6	25	rs2740608		1,00E-012
reg_DEFB	chr8	7240870	C	T	53	15,00%	1of6	8		rs2719433	2,76E-005
reg_DEFB	chr8	7241049	T	C	28	32,00%	2of6	9		rs2698963	8,38E-009
reg_DEFB	chr8	7241078	T	C	30	53,00%	3of6	16		rs71250726	1,00E-012
reg_DEFB	chr8	7241147	C	A	39	49,00%	3of6	19		rs2698964	1,00E-012
reg_DEFB	chr8	7241218	T	C	41	22,00%	1of6	9	rs7009957		3,24E-007
reg_DEFB	chr8	7241264	C	T	43	56,00%	3of6	24	rs4625055		1,00E-012
reg_DEFB	chr8	7241267	C	T	44	11,00%	1of6	5			3,31E-003
reg_DEFB	chr8	7241282	A	G	46	26,00%	2of6	12		rs2740607	4,14E-010
reg_DEFB	chr8	7241285	A	G	45	51,00%	3of6	23		rs3958824	1,00E-012
reg_DEFB	chr8	7241358	T	C	43	40,00%	2of6	17		rs2740157	1,00E-012
reg_DEFB	chr8	7241366	G	T	42	38,00%	2of6	16		rs2740606	1,00E-012
reg_DEFB	chr8	7241581	G	C	50	14,00%	1of6	7		rs4540430	1,43E-004
reg_DEFB	chr8	7241686	A	G	59	76,00%	5of6	45	rs4571754		1,00E-012
reg_DEFB	chr8	7241779	A	G	56	32,00%	2of6	18		rs2719626	1,00E-012
reg_DEFB	chr8	7241782	T	G	59	59,00%	4of6	35			1,00E-012
reg_DEFB	chr8	7241787	C	T	65	45,00%	3of6	29		rs2740605	1,00E-012

reg_DEFB	chr8	7241835	T	C	71	18,00%	1of6	13	rs2740604			8,42E-009
reg_DEFB	chr8	7241903	G	A	95	19,00%	1of6	18				8,12E-012
reg_DEFB	chr8	7242034	T	A	107	63,00%	4of6	67			rs71247376	1,00E-012
reg_DEFB	chr8	7242037	G	C	106	63,00%	4of6	67			rs71247376	1,00E-012
reg_DEFB	chr8	7242252	G	C	109	74,00%	4of6	81			rs3988892	1,00E-012
reg_DEFB	chr8	7242301	C	G	107	10,00%	1of6	11			rs2740750	3,88E-005
reg_DEFB	chr8	7242513	T	C	78	74,00%	4of6	58			rs3988890	1,00E-012
reg_DEFB	chr8	7242527	T	C	76	11,00%	1of6	8				3,67E-004
reg_DEFB	chr8	7242637	T	G	62	68,00%	4of6	42			rs3927359	1,00E-012
reg_DEFB	chr8	7242769	A	T	60	13,00%	1of6	8			rs72494256	6,90E-005
reg_DEFB	chr8	7242795	G	C	68	85,00%	5of6	58			rs3928107	1,00E-012
reg_DEFB	chr8	7242850	G	A	76	29,00%	2of6	22			rs2740749	1,00E-012
reg_DEFB	chr8	7242925	C	T	83	57,00%	3of6	47	rs2698913			1,00E-012
reg_DEFB	chr8	7242996	A	C	98	16,00%	1of6	16				9,75E-010
reg_DEFB	chr8	7243080	G	A	122	67,00%	4of6	82				1,00E-012
reg_DEFB	chr8	7243097	A	G	125	66,00%	4of6	83				1,00E-012
reg_DEFB	chr8	7243226	G	C	145	66,00%	4of6	96			rs56017112	1,00E-012
reg_DEFB	chr8	7243230	C	T	144	48,00%	3of6	69			rs2719607	1,00E-012
reg_DEFB	chr8	7243275	T	A	142	46,00%	3of6	65			rs2719604	1,00E-012
reg_DEFB	chr8	7243307	T	C	140	44,00%	3of6	62			rs2719603	1,00E-012
reg_DEFB	chr8	7243444	T	G	131	17,00%	1of6	22			rs55647651	1,00E-012
reg_DEFB	chr8	7243453	C	T	131	60,00%	4of6	79	rs4840743			1,00E-012
reg_DEFB	chr8	7243457	A	T	126	77,00%	5of6	97	rs4840275			1,00E-012
reg_DEFB	chr8	7243460	T	C	126	40,00%	2of6	50				1,00E-012
reg_DEFB	chr8	7243500	A	G	117	16,00%	1of6	19	rs2698912			3,87E-011
reg_DEFB	chr8	7243502	T	C	120	16,00%	1of6	19	rs2740153			4,87E-011
reg_DEFB	chr8	7243506	A	T	119	16,00%	1of6	19				4,19E-011
reg_DEFB	chr8	7243517	G	A	116	28,00%	2of6	32			rs2463985	1,00E-012
reg_DEFB	chr8	7243526	C	T	113	29,00%	2of6	33			rs2466114	1,00E-012
reg_DEFB	chr8	7243649	A	G	124	31,00%	2of6	38	rs2463984			1,00E-012
reg_DEFB	chr8	7243720	C	A	145	17,00%	1of6	25	rs2698911			1,00E-012
reg_DEFB	chr8	7243739	T	C	145	32,00%	2of6	46				1,00E-012
reg_DEFB	chr8	7243778	T	C	137	53,00%	3of6	73	rs2737539			1,00E-012
reg_DEFB	chr8	7243788	G	C	134	38,00%	2of6	51	rs2740152			1,00E-012
reg_DEFB	chr8	7243809	T	C	130	31,00%	2of6	40	rs2977410			1,00E-012
reg_DEFB	chr8	7243942	C	A	112	16,00%	1of6	18	rs2463983			1,20E-010
reg_DEFB	chr8	7243954	C	A	105	18,00%	1of6	19	rs2740150	rs34384791		1,41E-011
reg_DEFB	chr8	7243999	A	G	91	19,00%	1of6	17	rs2740149	rs35454996		3,92E-011
reg_DEFB	chr8	7244056	A	T	75	32,00%	2of6	24	rs6983203			1,00E-012
reg_DEFB	chr8	7244068	T	C	72	21,00%	1of6	15				1,03E-010
reg_DEFB	chr8	7244089	C	T	62	29,00%	2of6	18	rs7013742			1,00E-012
reg_DEFB	chr8	7244144	T	A	43	33,00%	2of6	14	rs6988073			1,00E-012
reg_DEFB	chr8	7244187	T	C	43	30,00%	2of6	13	rs9693476			2,49E-011
reg_DEFB	chr8	7244256	G	C	45	47,00%	3of6	21	rs2954087			1,00E-012
reg_DEFB	chr8	7244281	G	A	48	21,00%	1of6	10	rs9694285			1,22E-007
reg_DEFB	chr8	7244286	C	A	47	21,00%	1of6	10	rs9694349			9,84E-008
reg_DEFB	chr8	7244326	C	T	52	19,00%	1of6	10	rs9694359			2,71E-007
reg_DEFB	chr8	7244340	C	T	52	15,00%	1of6	8	rs2977415			2,39E-005
reg_DEFB	chr8	7244470	T	G	68	18,00%	1of6	12				4,82E-008
reg_DEFB	chr8	7244486	G	A	68	19,00%	1of6	13				4,85E-009
reg_DEFB	chr8	7244542	C	A	66	17,00%	1of6	11				3,19E-007
reg_DEFB	chr8	7244846	T	C	96	31,00%	2of6	30	rs34757760			1,00E-012
reg_DEFB	chr8	7244847	G	A	95	32,00%	2of6	30	rs34757760			1,00E-012

reg_DEFB	chr8	7244856	T	G	94	34,00%	2of6	32	rs35642932			1,00E-012
reg_DEFB	chr8	7244884	C	T	97	22,00%	1of6	21	rs35338468			1,00E-012
reg_DEFB	chr8	7244931	A	T	102	24,00%	1of6	24	rs35669108			1,00E-012
reg_DEFB	chr8	7244935	T	A	102	24,00%	1of6	24	rs34965334			1,00E-012
reg_DEFB	chr8	7245066	G	A	109	15,00%	1of6	16	rs2977405			4,82E-009
reg_DEFB	chr8	7245081	C	G	116	27,00%	2of6	31	rs2980526			1,00E-012
reg_DEFB	chr8	7245136	C	T	123	28,00%	2of6	34	rs2977406			1,00E-012
reg_DEFB	chr8	7245160	T	C	126	26,00%	2of6	33	rs2977407			1,00E-012
reg_DEFB	chr8	7245210	G	A	124	40,00%	2of6	50	rs2977408	rs2977409		1,00E-012
reg_DEFB	chr8	7245211	T	C	124	27,00%	2of6	33	rs2977409			1,00E-012
reg_DEFB	chr8	7245266	C	T	118	26,00%	2of6	31	rs2698902			1,00E-012
reg_DEFB	chr8	7245290	C	T	118	14,00%	1of6	17	rs35021319			2,17E-009
reg_DEFB	chr8	7245325	G	A	117	29,00%	2of6	34	rs2472432			1,00E-012
reg_DEFB	chr8	7245375	C	T	103	31,00%	2of6	32	rs2463977			1,00E-012
reg_DEFB	chr8	7245556	A	G	78	49,00%	3of6	38	rs2740748			1,00E-012
reg_DEFB	chr8	7245577	T	C	70	17,00%	1of6	12	rs2740148	rs4840745		6,75E-008
reg_DEFB	chr8	7245592	T	G	66	27,00%	2of6	18	rs2740747			1,00E-012
reg_DEFB	chr8	7245649	G	A	57	14,00%	1of6	8	rs2740146	rs4840746		4,73E-005
reg_DEFB	chr8	7245666	G	A	57	26,00%	2of6	15	rs2740145			6,21E-012
reg_DEFB	chr8	7245748	C	G	54	13,00%	1of6	7				2,34E-004
reg_DEFB	chr8	7245898	G	C	72	51,00%	3of6	37	rs2719573			1,00E-012
reg_DEFB	chr8	7246008	A	G	75	23,00%	1of6	17	rs2740746			3,52E-012
reg_DEFB	chr8	7246164	C	T	72	24,00%	1of6	17	rs2719567			1,00E-012
reg_DEFB	chr8	7246177	T	C	69	13,00%	1of6	9	rs2698901			2,93E-005
reg_DEFB	chr8	7246366	C	G	55	22,00%	1of6	12	rs2740144			3,83E-009
reg_DEFB	chr8	7246487	C	T	46	37,00%	2of6	17	rs2719563			1,00E-012
reg_DEFB	chr8	7246593	C	G	37	35,00%	2of6	13	rs2247710			2,43E-012
reg_DEFB	chr8	7246760	A	G	44	32,00%	2of6	14	rs2737552			1,00E-012
reg_DEFB	chr8	7246774	G	A	43	12,00%	1of6	5	rs2977411			2,99E-003
reg_DEFB	chr8	7246845	T	G	50	26,00%	2of6	13	rs2737553			8,84E-011
reg_DEFB	chr8	7246914	C	T	51	27,00%	2of6	14	rs2951098			1,73E-011
reg_DEFB	chr8	7246948	G	A	54	28,00%	2of6	15	rs34687479			2,57E-012
reg_DEFB	chr8	7246955	C	G	53	28,00%	2of6	15	rs35525365			1,89E-012
reg_DEFB	chr8	7246956	A	G	54	28,00%	2of6	15	rs35525365			2,57E-012
reg_DEFB	chr8	7247000	G	C	54	26,00%	2of6	14	rs35271627			1,74E-011
reg_DEFB	chr8	7247013	C	G	53	25,00%	1of6	13	rs34849967			1,80E-010
reg_DEFB	chr8	7247046	T	C	56	12,00%	1of6	7	rs2951099			2,94E-004
reg_DEFB	chr8	7247056	G	A	56	34,00%	2of6	19	rs2954062			1,00E-012
reg_DEFB	chr8	7247090	T	C	59	24,00%	1of6	14	rs34343796			6,50E-011
reg_DEFB	chr8	7247188	G	T	57	23,00%	1of6	13	rs2977412	rs35145385		4,82E-010
reg_DEFB	chr8	7247198	C	A	57	35,00%	2of6	20	rs2954061			1,00E-012
reg_DEFB	chr8	7247272	T	C	60	20,00%	1of6	12	rs35437838	rs2719562		1,10E-008
reg_DEFB	chr8	7247289	C	T	55	20,00%	1of6	11	rs2719561	rs35165471		4,48E-008
reg_DEFB	chr8	7247379	C	T	35	14,00%	1of6	5	rs2740143	rs34835930		1,17E-003
reg_DEFB	chr8	7249100	T	C	3	100,00%	6of6	3	rs2740138			1,22E-005
reg_DEFB	chr8	7249157	C	G	3	100,00%	6of6	3				1,22E-005
reg_DEFB	chr8	7249212	C	T	3	100,00%	6of6	3			rs4324940	1,22E-005
reg_DEFB	chr8	7249224	T	C	3	100,00%	6of6	3				1,22E-005
reg_DEFB	chr8	7249269	T	C	3	100,00%	6of6	3			rs62494626	1,22E-005
reg_DEFB	chr8	7249320	A	G	3	100,00%	6of6	3			rs62530484	1,22E-005
reg_DEFB	chr8	7249925	G	A	5	60,00%	4of6	3			rs62510592	1,18E-004
reg_DEFB	chr8	7250219	T	C	4	75,00%	4of6	3				4,78E-005
reg_DEFB	chr8	7250371	T	C	5	60,00%	4of6	3			rs71521097	1,18E-004

reg_DEFB	chr8	7250404	T	C	5	80,00%	5of6	4				1,37E-006
reg_DEFB	chr8	7250428	T	G	4	75,00%	4of6	3			rs28883954	4,78E-005
reg_DEFB	chr8	7250939	A	C	6	67,00%	4of6	4			rs13275935	4,04E-006
reg_DEFB	chr8	7250960	G	C	7	71,00%	4of6	5			rs2471963	1,30E-007
reg_DEFB	chr8	7251011	T	C	7	43,00%	3of6	3			rs62518636	3,97E-004
reg_DEFB	chr8	7251070	C	A	5	60,00%	4of6	3				1,18E-004
reg_DEFB	chr8	7251080	G	T	4	100,00%	6of6	4			rs2471964	2,80E-007
reg_DEFB	chr8	7251118	T	C	4	100,00%	6of6	4	rs2740126			2,80E-007
reg_DEFB	chr8	7252395	G	C	5	100,00%	6of6	5				6,44E-009
reg_DEFB	chr8	7252426	T	C	5	80,00%	5of6	4			rs71512308	1,37E-006
reg_DEFB	chr8	7252477	T	C	5	100,00%	6of6	5	rs2740113			6,44E-009
reg_DEFB	chr8	7252507	A	G	6	100,00%	6of6	6				1,48E-010
reg_DEFB	chr8	7252518	A	G	6	100,00%	6of6	6				1,48E-010
reg_DEFB	chr8	7252592	A	G	5	100,00%	6of6	5			rs7009930	6,44E-009
reg_DEFB	chr8	7252655	A	G	3	100,00%	6of6	3				1,22E-005
reg_DEFB	chr8	7252666	T	G	3	100,00%	6of6	3			rs59392872	1,22E-005
reg_DEFB	chr8	7253210	C	T	21	19,00%	1of6	4	rs3988850			1,22E-003
reg_DEFB	chr8	7253242	G	A	23	17,00%	1of6	4	rs3988851			1,75E-003
reg_DEFB	chr8	7253389	T	C	28	61,00%	4of6	17	rs2719572			1,00E-012
reg_DEFB	chr8	7253589	G	T	34	41,00%	2of6	14	rs2698825			1,00E-012
reg_DEFB	chr8	7253615	G	A	38	42,00%	2of6	16	rs3988853	rs2740112		1,00E-012
reg_DEFB	chr8	7253933	A	G	49	24,00%	1of6	12	rs2698823			9,21E-010
reg_DEFB	chr8	7253959	G	A	53	26,00%	2of6	14	rs2740111			1,31E-011
reg_DEFB	chr8	7254208	T	C	72	22,00%	1of6	16				2,16E-011
reg_DEFB	chr8	7254247	A	G	76	97,00%	6of6	74	rs3915368			1,00E-012
reg_DEFB	chr8	7254287	G	A	82	23,00%	1of6	19	rs2740109			1,00E-012
reg_DEFB	chr8	7254402	G	A	90	11,00%	1of6	10	rs2740108			4,43E-005
reg_DEFB	chr8	7254420	T	C	92	22,00%	1of6	20	rs2740107			1,00E-012
reg_DEFB	chr8	7254732	T	C	137	28,00%	2of6	38	rs2740742			1,00E-012
reg_DEFB	chr8	7254738	A	G	138	28,00%	2of6	39	rs2740105			1,00E-012
reg_DEFB	chr8	7254807	C	G	126	21,00%	1of6	26	rs2740741			1,00E-012
reg_DEFB	chr8	7254922	G	A	126	30,00%	2of6	38	rs2740104			1,00E-012
reg_DEFB	chr8	7255000	T	C	125	15,00%	1of6	19	rs2257803		T	1,02E-010
reg_DEFB	chr8	7255018	G	A	132	11,00%	1of6	15	rs2257802			4,55E-007
reg_DEFB	chr8	7255075	A	G	132	19,00%	1of6	25				1,00E-012
reg_DEFB	chr8	7255159	G	A	133	32,00%	2of6	43	rs2740103			1,00E-012
reg_DEFB	chr8	7255375	G	A	102	35,00%	2of6	36	rs2719462			1,00E-012
reg_DEFB	chr8	7255556	T	G	97	26,00%	2of6	25			rs2698822	1,00E-012
reg_DEFB	chr8	7255599	G	C	101	20,00%	1of6	20				1,00E-012
reg_DEFB	chr8	7255820	G	T	119	18,00%	1of6	21			rs2737494	1,91E-012
reg_DEFB	chr8	7255839	C	T	126	83,00%	5of6	105	rs3877964			1,00E-012
reg_DEFB	chr8	7255879	G	A	131	18,00%	1of6	24			rs2719586	1,00E-012
reg_DEFB	chr8	7255946	A	G	137	19,00%	1of6	26			rs2698821	1,00E-012
reg_DEFB	chr8	7255958	T	C	137	14,00%	1of6	19	rs3877963			4,92E-010
reg_DEFB	chr8	7255995	A	G	137	16,00%	1of6	22	rs2698820			4,22E-012
reg_DEFB	chr8	7256236	A	G	125	14,00%	1of6	18			rs2257751	7,47E-010
reg_DEFB	chr8	7256256	C	T	123	15,00%	1of6	18			rs2737497	5,71E-010
reg_DEFB	chr8	7256331	C	G	121	32,00%	2of6	39	rs2719545			1,00E-012
reg_DEFB	chr8	7256384	C	G	117	15,00%	1of6	18	rs6983800			2,54E-010
reg_DEFB	chr8	7256473	T	C	114	16,00%	1of6	18	rs2257742			1,63E-010
reg_DEFB	chr8	7256550	G	A	106	11,00%	1of6	12			rs3915496	6,50E-006
reg_DEFB	chr8	7256564	A	G	102	15,00%	1of6	15	rs2740099			1,41E-008
reg_DEFB	chr8	7256584	G	A	98	16,00%	1of6	16	rs2740098			9,75E-010

reg_DEFB	chr8	7256617	G	A	93	99,00%	6of6	92	rs28576922	rs3866483		1,00E-012
reg_DEFB	chr8	7256695	C	A	70	19,00%	1of6	13	rs2737917			7,03E-009
reg_DEFB	chr8	7256705	A	C	64	17,00%	1of6	11				2,30E-007
reg_DEFB	chr8	7256739	C	T	59	20,00%	1of6	12			rs7813724	8,98E-009
reg_DEFB	chr8	7256789	A	G	42	17,00%	1of6	7	rs2740097			4,53E-005
reg_DEFB	chr8	7256872	G	A	33	27,00%	2of6	9			rs71513067	4,21E-008
reg_DEFB	chr8	7256897	T	C	35	17,00%	1of6	6	rs2737916			1,35E-004
reg_DEFB	chr8	7257104	T	C	65	15,00%	1of6	10	rs2740095			2,33E-006
reg_DEFB	chr8	7257126	G	A	69	85,00%	5of6	59	rs4840277			1,00E-012
reg_DEFB	chr8	7257169	C	T	71	13,00%	1of6	9			rs55862841	3,70E-005
reg_DEFB	chr8	7257175	G	A	69	14,00%	1of6	10			rs55897833	4,08E-006
reg_DEFB	chr8	7257221	G	A	64	11,00%	1of6	7			rs73701026	6,71E-004
reg_DEFB	chr8	7257305	C	A	79	15,00%	1of6	12			rs71247231	2,68E-007
reg_DEFB	chr8	7257338	T	G	90	14,00%	1of6	13			rs56007428	1,58E-007
reg_DEFB	chr8	7257342	C	A	92	15,00%	1of6	14			rs56035080	2,71E-008
reg_DEFB	chr8	7257449	A	G	118	18,00%	1of6	21				1,00E-012
reg_DEFB	chr8	7257475	T	A	130	18,00%	1of6	23				1,00E-012
reg_DEFB	chr8	7257517	A	C	133	19,00%	1of6	25	rs2463965			1,00E-012
reg_DEFB	chr8	7257543	C	T	140	14,00%	1of6	20				1,05E-010
reg_DEFB	chr8	7257703	A	G	155	16,00%	1of6	25	rs2698818			1,00E-012
reg_DEFB	chr8	7257705	T	A	153	16,00%	1of6	24	rs2737915			1,00E-012
reg_DEFB	chr8	7257754	G	A	149	17,00%	1of6	25	rs2740093			1,00E-012
reg_DEFB	chr8	7257896	G	C	162	11,00%	1of6	18	rs2740739			4,78E-008
reg_DEFB	chr8	7258017	A	C	147	12,00%	1of6	18	rs2698817			1,04E-008
reg_DEFB	chr8	7258022	A	G	149	13,00%	1of6	19				2,07E-009
reg_DEFB	chr8	7258165	G	A	144	19,00%	1of6	27	rs2740092			1,00E-012
reg_DEFB	chr8	7258243	C	G	146	12,00%	1of6	18	rs2737527			9,32E-009
reg_DEFB	chr8	7258281	C	T	151	14,00%	1of6	21	rs2737528			6,12E-011
reg_DEFB	chr8	7258328	T	C	143	14,00%	1of6	20	rs2740738			1,56E-010
reg_DEFB	chr8	7258383	T	C	133	17,00%	1of6	23	rs3915367			1,00E-012
reg_DEFB	chr8	7258399	G	A	137	14,00%	1of6	19			rs71251805	4,92E-010
reg_DEFB	chr8	7258464	G	A	124	12,00%	1of6	15			rs72626630	2,00E-007
reg_DEFB	chr8	7258546	C	T	124	12,00%	1of6	15	rs2737529			2,00E-007
reg_DEFB	chr8	7258552	T	C	123	11,00%	1of6	14	rs2740737			1,07E-006
reg_DEFB	chr8	7258587	T	C	123	44,00%	3of6	54	rs2737530			1,00E-012
reg_DEFB	chr8	7259118	A	C	138	20,00%	1of6	28	rs2740736			1,00E-012
reg_DEFB	chr8	7259185	G	T	123	18,00%	1of6	22	rs3901234			1,00E-012
reg_DEFB	chr8	7259453	G	A	101	15,00%	1of6	15				1,23E-008
reg_DEFB	chr8	7259539	G	A	77	16,00%	1of6	12	rs41390446			2,01E-007
reg_DEFB	chr8	7259563	G	T	80	14,00%	1of6	11	rs41507446			2,31E-006
reg_DEFB	chr8	7259739	C	T	81	48,00%	3of6	39	rs2737531			1,00E-012
reg_DEFB	chr8	7259789	G	C	87	17,00%	1of6	15	rs2737912			1,47E-009
reg_DEFB	chr8	7259849	G	A	83	22,00%	1of6	18	rs2740091			1,89E-012
reg_DEFB	chr8	7260004	A	G	85	35,00%	2of6	30	rs2740090			1,00E-012
reg_DEFB	chr8	7260322	T	C	58	24,00%	1of6	14			rs71251804	5,05E-011
reg_DEFB	chr8	7260460	T	C	35	14,00%	1of6	5	rs2737532			1,17E-003
reg_DEFB	chr8	7261003	T	A	62	18,00%	1of6	11	rs2698815			1,64E-007
reg_DEFB	chr8	7261175	C	T	102	16,00%	1of6	16				1,79E-009
reg_DEFB	chr8	7261306	G	T	104	15,00%	1of6	16				2,40E-009
reg_DEFB	chr8	7261330	G	A	104	13,00%	1of6	14				1,32E-007
reg_DEFB	chr8	7261371	G	T	94	15,00%	1of6	14				3,59E-008
reg_DEFB	chr8	7261420	G	T	79	23,00%	1of6	18	rs2740089			1,00E-012
reg_DEFB	chr8	7261718	A	G	62	97,00%	6of6	60			rs71509106	1,00E-012

reg_DEFB	chr8	7261867	A	G	62	23,00%	1of6	14	rs2740086		1,34E-010
reg_DEFB	chr8	7262028	C	G	52	40,00%	2of6	21	rs2698827		1,00E-012
reg_DEFB	chr8	7262234	G	T	53	26,00%	2of6	14	rs2698828		1,31E-011
reg_DEFB	chr8	7262270	C	T	50	22,00%	1of6	11			1,55E-008
reg_DEFB	chr8	7262634	C	T	47	30,00%	2of6	14	rs2737535		4,96E-012
reg_DEFB	chr8	7262672	G	A	43	16,00%	1of6	7		rs71513122	5,30E-005
reg_DEFB	chr8	7262683	G	A	43	21,00%	1of6	9	rs3762041		5,00E-007
reg_DEFB	chr8	7262687	C	T	44	23,00%	1of6	10			5,02E-008
reg_DEFB	chr8	7262787	C	T	57	19,00%	1of6	11	rs3762040		6,62E-008
reg_DEFB	chr8	7262795	C	A	59	19,00%	1of6	11	rs3762039		9,63E-008
reg_DEFB	chr8	7262812	C	T	58	12,00%	1of6	7			3,66E-004
reg_DEFB	chr8	7263102	T	C	51	80,00%	5of6	41	rs4840278		1,00E-012
reg_DEFB	chr8	7263181	C	A	49	10,00%	1of6	5			5,30E-003
reg_DEFB	chr8	7263191	G	C	46	26,00%	2of6	12	rs3762052		4,14E-010
reg_DEFB	chr8	7263345	G	A	36	69,00%	4of6	25	rs4840751		1,00E-012
reg_DEFB	chr8	7263349	G	A	38	16,00%	1of6	6	rs3762051		2,17E-004
reg_DEFB	chr8	7263407	A	G	40	17,00%	1of6	7		rs71244071	3,26E-005
reg_DEFB	chr8	7263412	G	A	41	22,00%	1of6	9	rs2698830		3,24E-007
reg_DEFB	chr8	7263419	A	G	45	27,00%	2of6	12	rs2737910		3,12E-010
reg_DEFB	chr8	7263436	G	A	48	62,00%	4of6	30	rs4840752		1,00E-012
reg_DEFB	chr8	7263531	T	C	57	23,00%	1of6	13			4,82E-010
reg_DEFB	chr8	7263647	A	G	59	22,00%	1of6	13			7,64E-010
reg_DEFB	chr8	7263683	T	C	55	18,00%	1of6	10			4,70E-007
reg_DEFB	chr8	7263744	G	A	64	27,00%	2of6	17	rs2740083		1,00E-012
reg_DEFB	chr8	7263764	G	C	68	16,00%	1of6	11	rs2409862		4,36E-007
reg_DEFB	chr8	7263877	A	C	54	100,00%	6of6	54	rs6651513		1,00E-012
reg_DEFB	chr8	7263885	C	T	53	13,00%	1of6	7	rs3762038		2,08E-004
reg_DEFB	chr8	7263922	G	T	56	11,00%	1of6	6	rs3988843		1,80E-003
reg_DEFB	chr8	7264091	G	A	76	20,00%	1of6	15	rs3762037		2,04E-010
reg_DEFB	chr8	7264095	A	G	77	22,00%	1of6	17	rs3988844		5,67E-012
reg_DEFB	chr8	7264156	G	C	79	53,00%	3of6	42	rs2737536		1,00E-012
reg_DEFB	chr8	7264462	A	C	96	12,00%	1of6	12		rs71254898	2,28E-006
reg_DEFB	chr8	7264470	A	T	95	17,00%	1of6	16	rs2737909		6,05E-010
reg_DEFB	chr8	7264552	A	C	97	20,00%	1of6	19	rs2737538		3,14E-012
reg_DEFB	chr8	7264604	G	A	100	17,00%	1of6	17		rs71513120	1,56E-010
reg_DEFB	chr8	7264610	T	C	98	15,00%	1of6	15			8,04E-009
reg_DEFB	chr8	7264753	G	C	83	22,00%	1of6	18		rs71537819	1,89E-012
reg_DEFB	chr8	7264799	C	T	84	19,00%	1of6	16	rs3866482		9,04E-011
reg_DEFB	chr8	7264836	T	G	76	20,00%	1of6	15	rs4118281		2,04E-010
reg_DEFB	chr8	7264944	G	C	69	72,00%	4of6	50	rs4840753		1,00E-012
reg_DEFB	chr8	7264948	G	A	69	17,00%	1of6	12	rs3866481		5,71E-008
reg_DEFB	chr8	7265065	G	A	59	10,00%	1of6	6	rs3927369		2,35E-003
reg_DEFB	chr8	7265160	T	C	53	13,00%	1of6	7		rs71513119	2,08E-004
reg_DEFB	chr8	7265254	T	C	59	15,00%	1of6	9		rs71513117	8,00E-006
reg_DEFB	chr8	7265340	T	C	60	17,00%	1of6	10	rs2698832		1,09E-006
reg_DEFB	chr8	7265354	C	T	61	15,00%	1of6	9			1,06E-005
reg_DEFB	chr8	7265410	G	A	74	14,00%	1of6	10		rs71513116	7,76E-006
reg_DEFB	chr8	7265947	G	T	116	14,00%	1of6	16	rs3988845		1,21E-008
reg_DEFB	chr8	7265983	G	A	123	17,00%	1of6	21	rs3866480		3,83E-012
reg_DEFB	chr8	7266035	T	C	128	14,00%	1of6	18	rs3988846		1,11E-009
reg_DEFB	chr8	7266053	C	T	134	31,00%	2of6	42	rs2740731		1,00E-012
reg_DEFB	chr8	7266297	C	A	116	20,00%	1of6	23	rs3988829		1,00E-012
reg_DEFB	chr8	7266317	C	T	112	20,00%	1of6	22	rs3988830		1,00E-012

reg_DEFB	chr8	7266358	C	T	99	20,00%	1of6	20	rs3988831			1,00E-012
reg_DEFB	chr8	7266438	A	C	90	20,00%	1of6	18	rs3988832			8,42E-012
reg_DEFB	chr8	7266500	G	A	93	14,00%	1of6	13	rs746659			2,35E-007
reg_DEFB	chr8	7266687	C	T	80	31,00%	2of6	25	rs2740730			1,00E-012
reg_DEFB	chr8	7266786	T	C	79	11,00%	1of6	9			rs71513115	8,67E-005
reg_DEFB	chr8	7266858	C	T	93	13,00%	1of6	12			rs71513114	1,62E-006
reg_DEFB	chr8	7266868	G	T	99	17,00%	1of6	17				1,33E-010
reg_DEFB	chr8	7267031	T	G	92	100,00%	6of6	92	rs7815816			1,00E-012
reg_DEFB	chr8	7267055	A	C	86	21,00%	1of6	18			rs71513113	3,72E-012
reg_DEFB	chr8	7267305	C	G	70	20,00%	1of6	14	rs2737543			6,76E-010
reg_DEFB	chr8	7267395	G	T	60	12,00%	1of6	7			rs71513110	4,52E-004
reg_DEFB	chr8	7267436	A	C	65	15,00%	1of6	10			rs71513109	2,33E-006
reg_DEFB	chr8	7267461	T	G	60	15,00%	1of6	9			rs71513108	9,22E-006
reg_DEFB	chr8	7267609	T	G	53	15,00%	1of6	8			rs71513107	2,76E-005
reg_DEFB	chr8	7267636	C	G	53	19,00%	1of6	10			rs71513106	3,27E-007
reg_DEFB	chr8	7267855	A	G	68	78,00%	5of6	53	rs3958991			1,00E-012
reg_DEFB	chr8	7267995	T	C	78	17,00%	1of6	13	rs2740729			2,75E-008
reg_DEFB	chr8	7268025	T	A	89	20,00%	1of6	18	rs28610011	rs3866479		6,89E-012
reg_DEFB	chr8	7268520	A	T	116	21,00%	1of6	24			rs71513105	1,00E-012
reg_DEFB	chr8	7268623	G	T	113	63,00%	4of6	71	rs4840754			1,00E-012
reg_DEFB	chr8	7268975	C	T	105	14,00%	1of6	15	rs3958992			2,10E-008
reg_DEFB	chr8	7269055	T	C	101	69,00%	4of6	70				1,00E-012
reg_DEFB	chr8	7269068	G	C	103	17,00%	1of6	18	rs2737546			3,44E-011
reg_DEFB	chr8	7269085	G	T	101	65,00%	4of6	66				1,00E-012
reg_DEFB	chr8	7269368	T	C	70	61,00%	4of6	43			rs71511302	1,00E-012
reg_DEFB	chr8	7269455	T	C	68	24,00%	1of6	16			rs2698835	8,17E-012
reg_DEFB	chr8	7269776	G	A	113	46,00%	3of6	52			rs2737547	1,00E-012
reg_DEFB	chr8	7269817	C	A	116	97,00%	6of6	113			rs62636842	1,00E-012
reg_DEFB	chr8	7269942	G	T	125	100,00%	6of6	125				1,00E-012
reg_DEFB	chr8	7269967	T	C	124	98,00%	6of6	122				1,00E-012
reg_DEFB	chr8	7269989	G	T	124	20,00%	1of6	25			rs9720715	1,00E-012
reg_DEFB	chr8	7270164	G	A	128	15,00%	1of6	19				1,55E-010
reg_DEFB	chr8	7270168	G	T	127	65,00%	4of6	83				1,00E-012
reg_DEFB	chr8	7270508	C	T	149	17,00%	1of6	25			rs71511299	1,00E-012
reg_DEFB	chr8	7270703	T	C	121	42,00%	2of6	51			rs71511298	1,00E-012
reg_DEFB	chr8	7270806	C	T	91	20,00%	1of6	18	rs2740728			1,03E-011
reg_DEFB	chr8	7270961	C	T	63	25,00%	1of6	16	rs2740727			2,20E-012
reg_DEFB	chr8	7270994	G	A	61	26,00%	2of6	16	rs2737548			1,00E-012
reg_DEFB	chr8	7271125	G	A	59	19,00%	1of6	11	rs2737907			9,63E-008
reg_DEFB	chr8	7271226	C	A	59	42,00%	2of6	25				1,00E-012
reg_DEFB	chr8	7271434	T	C	79	82,00%	5of6	65			rs4543549	1,00E-012
reg_DEFB	chr8	7271577	A	G	92	11,00%	1of6	10			rs11986964	5,35E-005
reg_DEFB	chr8	7271654	T	C	119	19,00%	1of6	23				1,00E-012
reg_DEFB	chr8	7271670	G	C	121	16,00%	1of6	19			rs62636843	5,66E-011
reg_DEFB	chr8	7271706	G	A	133	17,00%	1of6	23			rs62636844	1,00E-012
reg_DEFB	chr8	7271837	C	T	144	56,00%	3of6	81			rs62636845	1,00E-012
reg_DEFB	chr8	7271938	C	T	145	81,00%	5of6	117			rs62636846	1,00E-012
reg_DEFB	chr8	7272539	G	T	100	13,00%	1of6	13	rs2737904			5,53E-007
reg_DEFB	chr8	7272799	T	G	137	26,00%	2of6	36	rs2737549			1,00E-012
reg_DEFB	chr8	7272812	G	T	138	51,00%	3of6	70	rs3988903			1,00E-012
reg_DEFB	chr8	7272893	G	A	150	83,00%	5of6	125	rs3988902			1,00E-012
reg_DEFB	chr8	7272905	G	C	147	99,00%	6of6	146			rs71509107	1,00E-012
reg_DEFB	chr8	7272963	A	G	142	23,00%	1of6	33			rs71511249	1,00E-012

reg_DEFB	chr8	7273000	T	A	142	23,00%	1of6	33			rs2737550		1,00E-012
reg_DEFB	chr8	7273160	G	A	96	31,00%	2of6	30			rs3988900		1,00E-012
reg_DEFB	chr8	7273179	A	G	89	12,00%	1of6	11			rs2740725		6,67E-006
reg_DEFB	chr8	7273532	G	C	75	55,00%	3of6	41			rs3866478		1,00E-012
reg_DEFB	chr8	7274151	C	T	106	66,00%	4of6	70	-	DEFB103B		rs62636848	1,00E-012
reg_DEFB	chr8	7274285	G	T	104	17,00%	1of6	18	-	DEFB103B			4,09E-011
reg_DEFB	chr8	7274449	C	A	78	12,00%	1of6	9	-	DEFB103B		rs62636849	7,85E-005
reg_DEFB	chr8	7274576	T	C	40	50,00%	3of6	20	-	DEFB103B	rs3789865		1,00E-012
reg_DEFB	chr8	7274772	T	C	46	11,00%	1of6	5	-	DEFB103B			4,03E-003
reg_DEFB	chr8	7274787	G	C	49	29,00%	2of6	14	-	DEFB103B	rs3789862		9,40E-012
reg_DEFB	chr8	7274794	G	A	51	20,00%	1of6	10	-	DEFB103B		rs73356491	2,23E-007
reg_DEFB	chr8	7274798	A	G	51	14,00%	1of6	7	-	DEFB103B		rs72709834	1,62E-004
reg_DEFB	chr8	7274817	T	C	52	15,00%	1of6	8	-	DEFB103B		rs71553678	2,39E-005
reg_DEFB	chr8	7274831	A	G	55	13,00%	1of6	7	-	DEFB103B		rs13256156	2,62E-004
reg_DEFB	chr8	7275507	G	T	72	17,00%	1of6	12				rs71511250	9,34E-008
reg_DEFB	chr8	7275590	A	G	56	21,00%	1of6	12			rs4461923		4,78E-009
reg_DEFB	chr8	7275599	C	G	53	11,00%	1of6	6			rs3958981		1,35E-003
reg_DEFB	chr8	7275809	A	G	31	84,00%	5of6	26				rs71511253	1,00E-012
reg_DEFB	chr8	7275837	T	A	28	18,00%	1of6	5			rs13439779		4,06E-004
reg_DEFB	chr8	7275956	G	A	48	21,00%	1of6	10					1,22E-007
reg_DEFB	chr8	7276019	A	G	62	23,00%	1of6	14			rs4347011		1,34E-010
reg_DEFB	chr8	7276276	A	T	117	27,00%	2of6	32			rs3762049		1,00E-012
reg_DEFB	chr8	7276298	A	G	116	16,00%	1of6	19			rs2737902		3,30E-011
reg_DEFB	chr8	7276309	G	T	118	12,00%	1of6	14			rs2737901		6,41E-007
reg_DEFB	chr8	7276674	A	C	115	70,00%	4of6	81			rs2737554		1,00E-012
reg_DEFB	chr8	7277001	G	A	75	19,00%	1of6	14			rs3762046		1,74E-009
reg_DEFB	chr8	7277359	T	C	95	69,00%	4of6	66				rs71509108	1,00E-012
reg_DEFB	chr8	7277387	T	C	92	36,00%	2of6	33				rs71511255	1,00E-012
reg_DEFB	chr8	7277422	T	A	95	74,00%	4of6	70				rs71509109	1,00E-012
reg_DEFB	chr8	7277424	C	A	94	74,00%	4of6	70				rs71509110	1,00E-012
reg_DEFB	chr8	7277451	C	G	100	24,00%	1of6	24				rs71511256	1,00E-012
reg_DEFB	chr8	7277460	G	A	103	25,00%	1of6	26				rs71511257	1,00E-012
reg_DEFB	chr8	7277507	T	C	102	48,00%	3of6	49					1,00E-012
reg_DEFB	chr8	7277521	C	G	105	30,00%	2of6	32				rs71511259	1,00E-012
reg_DEFB	chr8	7277545	C	T	107	25,00%	1of6	27				rs71511260	1,00E-012
reg_DEFB	chr8	7277566	G	A	110	25,00%	1of6	28				rs71509111	1,00E-012
reg_DEFB	chr8	7277662	A	G	113	27,00%	2of6	31				rs4999975	1,00E-012
reg_DEFB	chr8	7277710	T	C	106	29,00%	2of6	31				rs4999974	1,00E-012
reg_DEFB	chr8	7277745	A	T	99	22,00%	1of6	22				rs62636851	1,00E-012
reg_DEFB	chr8	7277755	T	C	103	32,00%	2of6	33			rs4999973		1,00E-012
reg_DEFB	chr8	7277810	A	G	102	30,00%	2of6	31					1,00E-012
reg_DEFB	chr8	7277830	C	G	104	30,00%	2of6	31					1,00E-012
reg_DEFB	chr8	7278010	C	T	112	81,00%	5of6	91					1,00E-012
reg_DEFB	chr8	7278055	C	T	120	16,00%	1of6	19			rs2698834		4,87E-011
reg_DEFB	chr8	7278116	C	T	128	32,00%	2of6	41				rs4840303	1,00E-012
reg_DEFB	chr8	7278208	T	C	138	18,00%	1of6	25					1,00E-012
reg_DEFB	chr8	7278542	T	A	144	88,00%	5of6	127			rs41380147		1,00E-012
reg_DEFB	chr8	7278548	A	C	144	33,00%	2of6	48				rs4840304	1,00E-012
reg_DEFB	chr8	7278563	G	T	144	32,00%	2of6	46				rs9693075	1,00E-012
reg_DEFB	chr8	7278594	T	C	147	84,00%	5of6	123					1,00E-012
reg_DEFB	chr8	7278660	C	A	165	30,00%	2of6	50				rs71276790	1,00E-012
reg_DEFB	chr8	7278727	C	T	168	30,00%	2of6	50				rs71276791	1,00E-012
reg_DEFB	chr8	7278763	C	T	175	27,00%	2of6	47				rs71276792	1,00E-012

reg_DEFB	chr8	7278790	G	T	172	24,00%	1of6	41			1,00E-012
reg_DEFB	chr8	7278926	A	G	176	29,00%	2of6	51		rs71276794	1,00E-012
reg_DEFB	chr8	7279069	A	C	160	21,00%	1of6	34		rs71276795	1,00E-012
reg_DEFB	chr8	7279284	C	A	100	25,00%	1of6	25	rs2463968		1,00E-012
reg_DEFB	chr8	7279289	T	C	97	80,00%	5of6	78			1,00E-012
reg_DEFB	chr8	7279315	T	C	85	81,00%	5of6	69			1,00E-012
reg_DEFB	chr8	7279414	G	A	76	39,00%	2of6	30		rs4840836	1,00E-012
reg_DEFB	chr8	7279450	C	T	77	32,00%	2of6	25	rs4840306		1,00E-012
reg_DEFB	chr8	7279477	A	G	76	37,00%	2of6	28		rs4840307	1,00E-012
reg_DEFB	chr8	7279596	C	G	83	31,00%	2of6	26		rs4840308	1,00E-012
reg_DEFB	chr8	7279621	C	G	92	29,00%	2of6	27		rs4840837	1,00E-012
reg_DEFB	chr8	7279696	A	T	110	44,00%	3of6	48	rs2737899		1,00E-012
reg_DEFB	chr8	7279780	C	G	107	61,00%	4of6	65		rs71276797	1,00E-012
reg_DEFB	chr8	7279877	C	T	105	55,00%	3of6	58		rs71276798	1,00E-012
reg_DEFB	chr8	7279897	C	G	106	15,00%	1of6	16			3,19E-009
reg_DEFB	chr8	7280090	T	C	83	83,00%	5of6	69			1,00E-012
reg_DEFB	chr8	7280221	C	T	61	52,00%	3of6	32		rs71299143	1,00E-012
reg_DEFB	chr8	7280227	A	T	63	81,00%	5of6	51			1,00E-012
reg_DEFB	chr8	7280259	T	G	59	27,00%	2of6	16	rs2698836		1,00E-012
reg_DEFB	chr8	7280364	G	C	65	68,00%	4of6	44		rs2737897	1,00E-012
reg_DEFB	chr8	7280790	G	A	87	25,00%	1of6	22	rs2737896		1,00E-012
reg_DEFB	chr8	7281118	T	C	69	16,00%	1of6	11	rs2737895		5,08E-007
reg_DEFB	chr8	7281292	C	T	61	80,00%	5of6	49	rs4840279		1,00E-012
reg_DEFB	chr8	7281324	C	A	55	15,00%	1of6	8	rs2740075		3,63E-005
reg_DEFB	chr8	7281372	T	G	58	53,00%	3of6	31	rs4247403		1,00E-012
reg_DEFB	chr8	7281483	A	T	50	18,00%	1of6	9	rs2740074		1,92E-006
reg_DEFB	chr8	7281562	C	T	70	40,00%	2of6	28		rs2737894	1,00E-012
reg_DEFB	chr8	7281568	A	T	71	18,00%	1of6	13	rs2740073		8,42E-009
reg_DEFB	chr8	7281649	T	A	66	23,00%	1of6	15		rs71509112	2,67E-011
reg_DEFB	chr8	7281710	G	T	61	57,00%	3of6	35	rs2698838		1,00E-012
reg_DEFB	chr8	7282390	A	C	86	19,00%	1of6	16			1,31E-010
reg_DEFB	chr8	7282443	T	C	96	80,00%	5of6	77			1,00E-012
reg_DEFB	chr8	7282482	G	A	95	20,00%	1of6	19		rs2698840	2,11E-012
reg_DEFB	chr8	7282560	C	T	101	29,00%	2of6	29		rs71267735	1,00E-012
reg_DEFB	chr8	7282592	G	A	100	31,00%	2of6	31		rs71267736	1,00E-012
reg_DEFB	chr8	7282670	T	C	109	86,00%	5of6	94			1,00E-012
reg_DEFB	chr8	7282719	A	G	114	12,00%	1of6	14		rs2737556	4,19E-007
reg_DEFB	chr8	7282985	T	A	115	23,00%	1of6	26		rs2737892	1,00E-012
reg_DEFB	chr8	7283081	C	T	101	29,00%	2of6	29	rs3915374		1,00E-012
reg_DEFB	chr8	7283244	C	A	66	21,00%	1of6	14		rs2740070	2,94E-010
reg_DEFB	chr8	7283283	C	T	64	42,00%	2of6	27		rs71267737	1,00E-012
reg_DEFB	chr8	7283305	A	G	68	84,00%	5of6	57			1,00E-012
reg_DEFB	chr8	7283750	C	T	87	24,00%	1of6	21		rs62636852	1,00E-012
reg_DEFB	chr8	7283805	C	T	120	17,00%	1of6	20			7,22E-012
reg_DEFB	chr8	7283828	C	G	127	32,00%	2of6	41	rs3915371		1,00E-012
reg_DEFB	chr8	7283981	G	A	127	33,00%	2of6	42		rs3915370	1,00E-012
reg_DEFB	chr8	7284058	A	G	126	16,00%	1of6	20			1,87E-011
reg_DEFB	chr8	7284122	A	G	119	16,00%	1of6	19	rs2698841		4,19E-011
reg_DEFB	chr8	7284285	G	A	75	29,00%	2of6	22	rs2698842		1,00E-012
reg_DEFB	chr8	7284343	T	G	66	21,00%	1of6	14			2,94E-010
reg_DEFB	chr8	7284450	G	A	54	17,00%	1of6	9			3,75E-006
reg_DEFB	chr8	7285027	C	T	119	34,00%	2of6	40	rs2740069		1,00E-012
reg_DEFB	chr8	7285028	A	G	118	54,00%	3of6	64	rs2740069		1,00E-012

reg_DEFB	chr8	7285076	C	T	104	20,00%	1of6	21			rs2698844			1,00E-012
reg_DEFB	chr8	7285531	G	C	76	43,00%	3of6	33			rs2740068	rs2737887		1,00E-012
reg_DEFB	chr8	7285565	G	T	72	18,00%	1of6	13			rs2737886			1,01E-008
reg_DEFB	chr8	7285576	C	G	72	22,00%	1of6	16			rs2740067			2,16E-011
reg_DEFB	chr8	7285682	T	C	74	18,00%	1of6	13					rs2698845	1,42E-008
reg_DEFB	chr8	7285744	C	T	74	78,00%	5of6	58			rs3988838			1,00E-012
reg_DEFB	chr8	7285834	T	A	66	62,00%	4of6	41			rs4501606			1,00E-012
reg_DEFB	chr8	7285981	C	G	58	72,00%	4of6	42					rs3988839	1,00E-012
reg_DEFB	chr8	7286036	C	T	60	33,00%	2of6	20					rs71267752	1,00E-012
reg_DEFB	chr8	7286545	G	A	89	84,00%	5of6	75			rs2280948			1,00E-012
reg_DEFB	chr8	7286699	A	T	98	33,00%	2of6	32			rs2280947			1,00E-012
reg_DEFB	chr8	7286812	G	A	92	30,00%	2of6	28			rs2280946			1,00E-012
reg_DEFB	chr8	7286995	G	C	74	57,00%	3of6	42			rs2280945			1,00E-012
reg_DEFB	chr8	7287010	G	A	74	20,00%	1of6	15			rs2280944			1,37E-010
reg_DEFB	chr8	7287258	C	T	45	11,00%	1of6	5						3,66E-003
reg_DEFB	chr8	7287421	C	G	40	20,00%	1of6	8					rs2740065	3,12E-006
reg_DEFB	chr8	7287438	G	A	38	13,00%	1of6	5						1,72E-003
reg_DEFB	chr8	7287713	T	C	36	58,00%	3of6	21						1,00E-012
reg_DEFB	chr8	7287838	G	T	31	26,00%	2of6	8					rs71267755	3,85E-007
reg_DEFB	chr8	7288049	G	A	36	14,00%	1of6	5			rs2740063			1,34E-003
reg_DEFB	chr8	7288153	G	A	36	19,00%	1of6	7			rs2740062			1,58E-005
reg_DEFB	chr8	7288169	G	A	38	13,00%	1of6	5						1,72E-003
reg_DEFB	chr8	7288182	G	A	39	28,00%	2of6	11			rs2698848			8,84E-010
reg_DEFB	chr8	7288411	T	A	57	19,00%	1of6	11			rs2740061			6,62E-008
reg_DEFB	chr8	7288723	A	G	73	16,00%	1of6	12			rs2740060			1,09E-007
reg_DEFB	chr8	7288985	T	C	82	61,00%	4of6	50			rs4466423			1,00E-012
reg_DEFB	chr8	7289081	C	G	89	18,00%	1of6	16						2,25E-010
reg_DEFB	chr8	7289545	G	A	83	65,00%	4of6	54			rs2740059			1,00E-012
reg_DEFB	chr8	7289788	C	T	71	17,00%	1of6	12			rs2698849			7,95E-008
reg_DEFB	chr8	7290162	T	G	39	64,00%	4of6	25			rs2698850			1,00E-012
reg_DEFB	chr8	7290257	G	A	48	12,00%	1of6	6			rs2740057			7,93E-004
reg_DEFB	chr8	7290567	C	T	72	21,00%	1of6	15			rs2740056			1,03E-010
reg_DEFB	chr8	7290634	G	A	64	14,00%	1of6	9			rs2740055			1,58E-005
reg_DEFB	chr8	7290637	G	C	64	14,00%	1of6	9			rs2740054			1,58E-005
reg_DEFB	chr8	7290646	G	A	63	59,00%	4of6	37			rs2740053			1,00E-012
reg_DEFB	chr8	7290832	C	T	44	16,00%	1of6	7					rs3110053	6,18E-005
reg_DEFB	chr8	7290907	A	G	52	15,00%	1of6	8			rs2740052			2,39E-005
reg_DEFB	chr8	7291101	G	A	102	25,00%	1of6	26					rs71267758	1,00E-012
reg_DEFB	chr8	7291778	C	T	53	17,00%	1of6	9			rs2740050			3,19E-006
reg_DEFB	chr8	7291811	C	T	50	38,00%	2of6	19					rs71267759	1,00E-012
reg_DEFB	chr8	7291975	G	T	67	31,00%	2of6	21					rs71267760	1,00E-012
reg_DEFB	chr8	7292033	G	C	64	31,00%	2of6	20					rs71267761	1,00E-012
reg_DEFB	chr8	7292224	G	A	60	12,00%	1of6	7						4,52E-004
reg_DEFB	chr8	7292386	T	C	48	33,00%	2of6	16					rs71267762	1,00E-012
reg_DEFB	chr8	7292398	G	C	48	15,00%	1of6	7			rs2698915			1,10E-004
reg_DEFB	chr8	7292605	G	C	30	27,00%	2of6	8					rs71267763	2,91E-007
reg_DEFB	chr8	7292760	G	A	35	26,00%	2of6	9	-	SPAG11B	rs2740048			7,40E-008
reg_DEFB	chr8	7292773	T	C	37	100,00%	6of6	37	-	SPAG11B			rs4626629	1,00E-012
reg_DEFB	chr8	7292820	C	G	39	77,00%	5of6	30	-	SPAG11B			rs2740047	1,00E-012
reg_DEFB	chr8	7292896	A	C	36	100,00%	6of6	36	-	SPAG11B	rs3901154			1,00E-012
reg_DEFB	chr8	7293009	A	G	37	11,00%	1of6	4	-	SPAG11B				1,01E-002
reg_DEFB	chr8	7293040	A	C	30	20,00%	1of6	6	-	SPAG11B	rs2740046			5,46E-005
reg_DEFB	chr8	7293741	A	G	7	100,00%	6of6	7	-	SPAG11B			rs2740041	3,40E-012

reg_DEFB	chr8	7293835	G	A	13	62,00%	4of6	8			-	SPAG11B	rs2740040		9,13E-011
reg_DEFB	chr8	7293918	T	A	19	42,00%	2of6	8			-	SPAG11B		rs71511262	4,72E-009
reg_DEFB	chr8	7294380	G	C	43	28,00%	2of6	12			-	SPAG11B	rs2737557		1,88E-010
reg_DEFB	chr8	7294480	A	C	53	32,00%	2of6	17			-	SPAG11B		rs71511263	1,00E-012
reg_DEFB	chr8	7294502	C	G	62	45,00%	3of6	28			-	SPAG11B	rs2853664		1,00E-012
reg_DEFB	chr8	7294800	C	T	107	48,00%	3of6	51			-	SPAG11B	rs2853663		1,00E-012
reg_DEFB	chr8	7295003	A	G	141	78,00%	5of6	110			-	SPAG11B	rs2853661		1,00E-012
reg_DEFB	chr8	7295159	G	C	123	100,00%	6of6	123			-	SPAG11B		rs3915372	1,00E-012
reg_DEFB	chr8	7295311	T	A	123	46,00%	3of6	57			-	SPAG11B	rs2853660		1,00E-012
reg_DEFB	chr8	7295321	A	T	123	50,00%	3of6	62			-	SPAG11B	rs2737558		1,00E-012
reg_DEFB	chr8	7295346	G	A	115	15,00%	1of6	17			-	SPAG11B			1,45E-009
reg_DEFB	chr8	7295375	G	A	114	17,00%	1of6	19			-	SPAG11B	rs4089926		2,39E-011
reg_DEFB	chr8	7295796	T	C	99	85,00%	5of6	84	H	R	-2	SPAG11B	rs1042797		1,00E-012
reg_DEFB	chr8	7295813	A	C	97	24,00%	1of6	23	I	M	-2	SPAG11B	rs12063		1,00E-012
reg_DEFB	chr8	7295820	G	A	97	43,00%	3of6	42	P	L	-2	SPAG11B	rs2256100		1,00E-012
reg_DEFB	chr8	7295894	C	A	90	46,00%	3of6	41			-	SPAG11B	rs2853659		1,00E-012
reg_DEFB	chr8	7295903	G	A	87	45,00%	3of6	39			-	SPAG11B	rs2738036		1,00E-012
reg_DEFB	chr8	7295939	C	T	83	40,00%	2of6	33			-	SPAG11B		rs2737559	1,00E-012
reg_DEFB	chr8	7296080	T	C	80	44,00%	3of6	35	D	G	-2	SPAG11B	rs2738035		1,00E-012
reg_DEFB	chr8	7296085	A	G	82	44,00%	3of6	36	C	C	-2	SPAG11B		rs61749560	1,00E-012
reg_DEFB	chr8	7296092	C	T	83	45,00%	3of6	37	R	K	-2	SPAG11B		rs61749561	1,00E-012
reg_DEFB	chr8	7296116	C	T	86	43,00%	3of6	37	R	Q	-2	SPAG11B	rs2853658		1,00E-012
reg_DEFB	chr8	7296139	G	C	90	33,00%	2of6	30			-	SPAG11B	rs2294141		1,00E-012
reg_DEFB	chr8	7296208	A	G	92	96,00%	6of6	88			-	SPAG11B	rs4840280		1,00E-012
reg_DEFB	chr8	7296408	T	C	97	40,00%	2of6	39			-	SPAG11B	rs12682529		1,00E-012
reg_DEFB	chr8	7297111	T	C	106	23,00%	1of6	24			-	SPAG11B	rs2853656		1,00E-012
reg_DEFB	chr8	7297409	G	T	91	18,00%	1of6	16			-	SPAG11B	rs2738034		3,18E-010
reg_DEFB	chr8	7297513	G	A	109	19,00%	1of6	21			-	SPAG11B	rs2738033		1,00E-012
reg_DEFB	chr8	7297645	A	T	105	29,00%	2of6	30			-	SPAG11B		rs71511266	1,00E-012
reg_DEFB	chr8	7297906	C	T	103	14,00%	1of6	14			-	SPAG11B	rs2738032		1,17E-007
reg_DEFB	chr8	7297910	G	A	103	16,00%	1of6	16			-	SPAG11B	rs2853654		2,07E-009
reg_DEFB	chr8	7297951	A	G	96	18,00%	1of6	17			-	SPAG11B	rs2740034		7,97E-011
reg_DEFB	chr8	7298154	C	T	65	11,00%	1of6	7			-	SPAG11B		rs4840309	7,38E-004
reg_DEFB	chr8	7298212	T	G	73	48,00%	3of6	35			-	SPAG11B	rs2738031		1,00E-012
reg_DEFB	chr8	7298298	T	A	86	15,00%	1of6	13			-	SPAG11B	rs2738030		9,13E-008
reg_DEFB	chr8	7298320	A	G	97	14,00%	1of6	14			-	SPAG11B	rs2740033		5,40E-008
reg_DEFB	chr8	7298410	C	A	119	13,00%	1of6	15			-	SPAG11B	rs2738029		1,16E-007
reg_DEFB	chr8	7298525	T	G	110	98,00%	6of6	108			-	SPAG11B		rs62636856	1,00E-012
reg_DEFB	chr8	7298554	T	C	112	16,00%	1of6	18			-	SPAG11B			1,20E-010
reg_DEFB	chr8	7298614	T	C	107	12,00%	1of6	13			-	SPAG11B	rs2853653		1,21E-006
reg_DEFB	chr8	7298676	G	T	91	38,00%	2of6	35			-	SPAG11B		rs71511269	1,00E-012
reg_DEFB	chr8	7298691	T	G	86	41,00%	2of6	35			-	SPAG11B		rs71511270	1,00E-012
reg_DEFB	chr8	7298786	T	C	70	23,00%	1of6	16			-	SPAG11B	rs2738027		1,34E-011
reg_DEFB	chr8	7298840	C	T	60	22,00%	1of6	13			-	SPAG11B		rs72056310	9,56E-010
reg_DEFB	chr8	7299088	C	A	75	36,00%	2of6	27			-	SPAG11B		rs71235968	1,00E-012
reg_DEFB	chr8	7299344	T	G	117	15,00%	1of6	18			-	SPAG11B	rs2737562		2,54E-010
reg_DEFB	chr8	7299529	T	C	123	85,00%	5of6	105			-	SPAG11B	rs2738025		1,00E-012
reg_DEFB	chr8	7299717	G	C	121	18,00%	1of6	22			-	SPAG11B	rs2738024		1,00E-012
reg_DEFB	chr8	7299852	A	T	102	18,00%	1of6	18			-	SPAG11B		rs71235967	2,90E-011
reg_DEFB	chr8	7300154	T	C	27	11,00%	1of6	3			-	SPAG11B			2,36E-002
reg_DEFB	chr8	7300286	A	G	40	10,00%	1of6	4			-	SPAG11B			1,32E-002
reg_DEFB	chr8	7300402	A	G	68	16,00%	1of6	11			-	SPAG11B	rs2266517		4,36E-007
reg_DEFB	chr8	7300520	T	C	93	20,00%	1of6	19			-	SPAG11B			1,00E-012

reg_DEFB	chr8	7300697	C	T	116	13,00%	1of6	15	-	SPAG11B	rs2738021		8,21E-008
reg_DEFB	chr8	7300781	A	G	108	11,00%	1of6	12	-	SPAG11B	rs2740032		7,90E-006
reg_DEFB	chr8	7301157	C	G	78	36,00%	2of6	28	-	SPAG11B		rs71235965	1,00E-012
reg_DEFB	chr8	7301232	G	A	77	16,00%	1of6	12	-	SPAG11B		rs71235964	2,01E-007
reg_DEFB	chr8	7301270	A	G	75	25,00%	1of6	19	-	SPAG11B		rs71235963	1,00E-012
reg_DEFB	chr8	7301410	G	T	59	44,00%	3of6	26	-	SPAG11B		rs71235962	1,00E-012
reg_DEFB	chr8	7301857	A	G	41	83,00%	5of6	34	-	SPAG11B	rs2740031		1,00E-012
reg_DEFB	chr8	7301960	T	G	48	12,00%	1of6	6	-	SPAG11B		rs55978454	7,93E-004
reg_DEFB	chr8	7301986	G	A	49	96,00%	6of6	47	-	SPAG11B	rs2738017		1,00E-012
reg_DEFB	chr8	7302094	G	A	57	28,00%	2of6	16	-	SPAG11B		rs71235961	1,00E-012
reg_DEFB	chr8	7302098	C	T	57	23,00%	1of6	13	-	SPAG11B		rs2740718	4,82E-010
reg_DEFB	chr8	7302236	T	C	62	34,00%	2of6	21	-	SPAG11B		rs71235960	1,00E-012
reg_DEFB	chr8	7302765	C	T	73	62,00%	4of6	45	-	SPAG11B	rs4840756		1,00E-012
reg_DEFB	chr8	7302817	C	T	68	13,00%	1of6	9	-	SPAG11B	rs2740716		2,61E-005
reg_DEFB	chr8	7303071	A	G	75	13,00%	1of6	10	-	SPAG11B			8,77E-006
reg_DEFB	chr8	7303100	G	T	83	16,00%	1of6	13	-	SPAG11B			5,92E-008
reg_DEFB	chr8	7303169	G	A	92	14,00%	1of6	13	-	SPAG11B			2,06E-007
reg_DEFB	chr8	7303532	G	A	80	19,00%	1of6	15	-	SPAG11B			4,38E-010
reg_DEFB	chr8	7303590	C	G	76	26,00%	2of6	20	-	SPAG11B	rs2740030		1,00E-012
reg_DEFB	chr8	7303683	A	T	75	27,00%	2of6	20	-	SPAG11B	rs4532613		1,00E-012
reg_DEFB	chr8	7303698	G	A	71	21,00%	1of6	15	-	SPAG11B		rs71235959	8,33E-011
reg_DEFB	chr8	7303880	C	T	49	63,00%	4of6	31	-	SPAG11B		rs62641376	1,00E-012
reg_DEFB	chr8	7303899	C	G	48	83,00%	5of6	40	-	SPAG11B		rs71526141	1,00E-012
reg_DEFB	chr8	7303958	T	C	50	76,00%	5of6	38	-	SPAG11B		rs2740712	1,00E-012
reg_DEFB	chr8	7303987	G	A	52	81,00%	5of6	42	-	SPAG11B		rs34315736	1,00E-012
reg_DEFB	chr8	7303999	A	G	51	82,00%	5of6	42	-	SPAG11B		rs7464358	1,00E-012
reg_DEFB	chr8	7304135	G	A	51	22,00%	1of6	11	-	SPAG11B			1,94E-008
reg_DEFB	chr8	7304356	C	G	95	15,00%	1of6	14	-	SPAG11B		rs71242685	4,12E-008
reg_DEFB	chr8	7304384	C	G	98	10,00%	1of6	10	-	SPAG11B	rs2738015		9,18E-005
reg_DEFB	chr8	7304399	A	C	98	20,00%	1of6	20	-	SPAG11B		rs2853665	1,00E-012
reg_DEFB	chr8	7304490	A	G	87	16,00%	1of6	14	-	SPAG11B		rs2737566	1,29E-008
reg_DEFB	chr8	7304912	A	G	47	100,00%	6of6	47	-	SPAG11B	rs4840757		1,00E-012
reg_DEFB	chr8	7305009	G	A	57	21,00%	1of6	12	-	SPAG11B			5,92E-009
reg_DEFB	chr8	7305131	G	T	81	16,00%	1of6	13	-	SPAG11B			4,39E-008
reg_DEFB	chr8	7305501	C	T	112	14,00%	1of6	16	-	SPAG11B			7,20E-009
reg_DEFB	chr8	7306469	G	T	124	82,00%	5of6	102	-	SPAG11B	rs4840282		1,00E-012
reg_DEFB	chr8	7306543	G	A	123	15,00%	1of6	18	-	SPAG11B			5,71E-010
reg_DEFB	chr8	7306637	G	C	122	23,00%	1of6	28	-	SPAG11B			1,00E-012
reg_DEFB	chr8	7307198	T	C	60	100,00%	6of6	60	-	SPAG11B		rs62636859	1,00E-012
reg_DEFB	chr8	7307400	C	G	45	11,00%	1of6	5	-	SPAG11B	rs2740708		3,66E-003
reg_DEFB	chr8	7307943	G	A	63	90,00%	5of6	57	-	SPAG11B	rs2251705		1,00E-012
reg_DEFB	chr8	7308164	G	A	66	23,00%	1of6	15	-	SPAG11B	rs3817721		2,67E-011
reg_DEFB	chr8	7308325	C	A	63	22,00%	1of6	14	-	SPAG11B			1,51E-010
reg_DEFB	chr8	7308331	A	G	64	37,00%	2of6	24	-	SPAG11B	rs2272769		1,00E-012
reg_DEFB	chr8	7308457	C	T	64	19,00%	1of6	12	-	SPAG11B		rs71242684	2,37E-008
reg_DEFB	chr8	7308498	T	A	60	20,00%	1of6	12	-	SPAG11B	rs2272768		1,10E-008
reg_DEFB	chr8	7308534	G	A	64	20,00%	1of6	13	-	SPAG11B			2,21E-009
reg_DEFB	chr8	7308849	C	G	131	13,00%	1of6	17			rs4521786		1,09E-008
reg_DEFB	chr8	7309001	C	T	130	17,00%	1of6	22			rs3762045		1,00E-012
reg_DEFB	chr8	7309292	G	A	120	22,00%	1of6	26			rs17149290		1,00E-012
reg_DEFB	chr8	7309563	T	C	94	13,00%	1of6	12			rs2740704		1,82E-006
reg_DEFB	chr8	7309624	T	G	85	45,00%	3of6	38			rs2738013		1,00E-012
reg_DEFB	chr8	7309975	G	A	100	99,00%	6of6	99					1,00E-012

reg_DEFB	chr8	7310156	A	C	122	57,00%	3of6	70					rs2740702		1,00E-012
reg_DEFB	chr8	7310241	G	T	126	69,00%	4of6	87					rs4840283		1,00E-012
reg_DEFB	chr8	7310265	T	A	124	50,00%	3of6	62					rs2740701		1,00E-012
reg_DEFB	chr8	7310309	C	T	130	14,00%	1of6	18					rs2740700		1,43E-009
reg_DEFB	chr8	7310735	G	A	124	19,00%	1of6	24							1,00E-012
reg_DEFB	chr8	7310943	T	G	141	19,00%	1of6	27							1,00E-012
reg_DEFB	chr8	7311305	A	T	114	15,00%	1of6	17							1,26E-009
reg_DEFB	chr8	7311383	G	A	101	14,00%	1of6	14							9,08E-008
reg_DEFB	chr8	7311411	C	T	97	13,00%	1of6	13							3,87E-007
reg_DEFB	chr8	7311441	C	T	81	100,00%	6of6	81							1,00E-012
reg_DEFB	chr8	7311522	A	T	60	97,00%	6of6	58							1,00E-012
reg_DEFB	chr8	7311592	C	T	58	10,00%	1of6	6							2,15E-003
reg_DEFB	chr8	7311642	G	A	64	11,00%	1of6	7						rs3132871	6,71E-004
reg_DEFB	chr8	7311904	A	G	117	16,00%	1of6	19							3,87E-011
reg_DEFB	chr8	7312136	G	T	124	18,00%	1of6	22							1,00E-012
reg_DEFB	chr8	7312244	T	C	126	19,00%	1of6	24							1,00E-012
reg_DEFB	chr8	7312534	T	C	128	13,00%	1of6	17						rs71242683	7,66E-009
reg_DEFB	chr8	7312649	A	G	137	66,00%	4of6	90					rs2737570		1,00E-012
reg_DEFB	chr8	7312682	G	T	153	38,00%	2of6	58					rs28374698		1,00E-012
reg_DEFB	chr8	7312925	A	C	156	16,00%	1of6	25					rs2737572		1,00E-012
reg_DEFB	chr8	7313500	C	G	111	35,00%	2of6	39					rs2737573		1,00E-012
reg_DEFB	chr8	7313562	C	T	123	11,00%	1of6	14							1,07E-006
reg_DEFB	chr8	7313761	A	G	108	20,00%	1of6	22					rs2737574		1,00E-012
reg_DEFB	chr8	7314038	C	T	134	45,00%	3of6	60					rs2740026		1,00E-012
reg_DEFB	chr8	7314172	A	G	129	17,00%	1of6	22					rs2740698		1,00E-012
reg_DEFB	chr8	7314381	G	C	112	17,00%	1of6	19					rs2737575		1,72E-011
reg_DEFB	chr8	7314399	C	A	112	47,00%	3of6	53					rs2740025		1,00E-012
reg_DEFB	chr8	7314602	G	C	119	17,00%	1of6	20						rs71242681	6,12E-012
reg_DEFB	chr8	7314802	A	G	170	18,00%	1of6	31							1,00E-012
reg_DEFB	chr8	7315137	A	G	130	18,00%	1of6	23					rs2740697		1,00E-012
reg_DEFB	chr8	7315210	C	T	136	16,00%	1of6	22					rs2740024		3,62E-012
reg_DEFB	chr8	7315223	G	A	134	14,00%	1of6	19						rs71242680	3,34E-010
reg_DEFB	chr8	7315394	C	T	125	16,00%	1of6	20	R	Q	-2	DEFB104A		rs71242679	1,60E-011
reg_DEFB	chr8	7315477	A	C	103	47,00%	3of6	48			-	DEFB104A		rs2740023	1,00E-012
reg_DEFB	chr8	7315525	G	A	97	19,00%	1of6	18			-	DEFB104A			1,18E-011
reg_DEFB	chr8	7315533	G	A	95	95,00%	6of6	90			-	DEFB104A			1,00E-012
reg_DEFB	chr8	7315562	G	T	104	17,00%	1of6	18			-	DEFB104A	rs2740696		4,09E-011
reg_DEFB	chr8	7315606	C	T	116	11,00%	1of6	13			-	DEFB104A	rs2740022		3,04E-006
reg_DEFB	chr8	7315640	A	G	112	46,00%	3of6	52			-	DEFB104A		rs71242678	1,00E-012
reg_DEFB	chr8	7315688	A	G	118	19,00%	1of6	22			-	DEFB104A		rs71242677	1,00E-012
reg_DEFB	chr8	7315783	T	C	121	15,00%	1of6	18			-	DEFB104A		rs71242676	4,34E-010
reg_DEFB	chr8	7315804	C	A	120	20,00%	1of6	24			-	DEFB104A			1,00E-012
reg_DEFB	chr8	7315842	G	C	120	12,00%	1of6	14			-	DEFB104A			7,88E-007
reg_DEFB	chr8	7316076	T	C	84	15,00%	1of6	13			-	DEFB104A			6,86E-008
reg_DEFB	chr8	7316113	C	T	85	86,00%	5of6	73			-	DEFB104A		rs62639764	1,00E-012
reg_DEFB	chr8	7316116	A	C	83	49,00%	3of6	41			-	DEFB104A	rs2740021		1,00E-012
reg_DEFB	chr8	7316428	C	T	97	96,00%	6of6	93			-	DEFB104A			1,00E-012
reg_DEFB	chr8	7316918	C	A	67	15,00%	1of6	10			-	DEFB104A	rs2740020		3,10E-006
reg_DEFB	chr8	7316983	C	T	63	51,00%	3of6	32			-	DEFB104A	rs2740019		1,00E-012
reg_DEFB	chr8	7317035	C	T	71	100,00%	6of6	71			-	DEFB104A		rs62639766	1,00E-012
reg_DEFB	chr8	7317106	G	T	83	23,00%	1of6	19			-	DEFB104A	rs2740018		1,00E-012
reg_DEFB	chr8	7317157	G	A	84	48,00%	3of6	40			-	DEFB104A		rs62641371	1,00E-012
reg_DEFB	chr8	7317224	G	A	89	12,00%	1of6	11			-	DEFB104A			6,67E-006

reg_DEFB	chr8	7317239	G	A	95	33,00%	2of6	31			-	DEFB104A	rs2737576			1,00E-012
reg_DEFB	chr8	7317261	C	A	97	97,00%	6of6	94			-	DEFB104A				1,00E-012
reg_DEFB	chr8	7317363	C	A	98	22,00%	1of6	22			-	DEFB104A	rs2740011			1,00E-012
reg_DEFB	chr8	7317411	T	C	81	22,00%	1of6	18			-	DEFB104A				1,00E-012
reg_DEFB	chr8	7317588	G	A	59	19,00%	1of6	11			-	DEFB104A	rs6985641			9,63E-008
reg_DEFB	chr8	7317699	A	G	36	11,00%	1of6	4			-	DEFB104A	rs28681639			9,17E-003
reg_DEFB	chr8	7317749	T	G	26	15,00%	1of6	4			-	DEFB104A	rs2740007			2,79E-003
reg_DEFB	chr8	7317813	C	G	17	94,00%	6of6	16			-	DEFB104A	rs7001088			1,00E-012
reg_DEFB	chr8	7318122	A	T	5	100,00%	6of6	5			-	DEFB104A	rs7012750			6,44E-009
reg_DEFB	chr8	7318293	T	C	9	33,00%	2of6	3			-	DEFB104A			rs73357863	9,21E-004
reg_DEFB	chr8	7318523	G	A	29	10,00%	1of6	3			-	DEFB104A				2,85E-002
reg_DEFB	chr8	7318552	G	T	30	10,00%	1of6	3			-	DEFB104A				3,11E-002
reg_DEFB	chr8	7318591	C	T	31	29,00%	2of6	9			-	DEFB104A	rs2680505			2,29E-008
reg_DEFB	chr8	7318603	G	C	33	12,00%	1of6	4			-	DEFB104A	rs6990950	rs28609051		6,72E-003
reg_DEFB	chr8	7318648	C	T	37	11,00%	1of6	4			-	DEFB104A				1,01E-002
reg_DEFB	chr8	7318756	A	T	35	54,00%	3of6	19			-	DEFB104A	rs2739991			1,00E-012
reg_DEFB	chr8	7318925	T	C	52	62,00%	4of6	32			-	DEFB104A	rs2739988			1,00E-012
reg_DEFB	chr8	7319055	T	C	81	62,00%	4of6	50			-	DEFB104A	rs2739981			1,00E-012
reg_DEFB	chr8	7319063	T	C	80	64,00%	4of6	51			-	DEFB104A	rs2680506			1,00E-012
reg_DEFB	chr8	7319275	A	T	97	44,00%	3of6	43			-	DEFB104A	rs2739976			1,00E-012
reg_DEFB	chr8	7319333	T	C	98	18,00%	1of6	18			-	DEFB104A				1,42E-011
reg_DEFB	chr8	7319456	T	C	92	54,00%	3of6	50			-	DEFB104A	rs2739969			1,00E-012
reg_DEFB	chr8	7319556	A	G	84	52,00%	3of6	44			-	DEFB104A			rs2740692	1,00E-012
reg_DEFB	chr8	7319599	C	T	84	44,00%	3of6	37			-	DEFB104A			rs2739962	1,00E-012
reg_DEFB	chr8	7319638	C	T	81	41,00%	2of6	33			-	DEFB104A			rs2739960	1,00E-012
reg_DEFB	chr8	7319747	A	G	83	86,00%	5of6	71			-	DEFB104A	rs4259430			1,00E-012
reg_DEFB	chr8	7319771	T	C	85	87,00%	5of6	74			-	DEFB104A			rs17843872	1,00E-012
reg_DEFB	chr8	7319973	T	C	83	18,00%	1of6	15	I	V	-1	DEFB104B	rs2680507			7,41E-010
reg_DEFB	chr8	7320136	G	C	55	71,00%	4of6	39					rs28590291			1,00E-012
reg_DEFB	chr8	7320212	C	G	53	68,00%	4of6	36								1,00E-012
reg_DEFB	chr8	7320256	C	T	56	64,00%	4of6	36								1,00E-012
reg_DEFB	chr8	7320389	C	T	64	70,00%	4of6	45								1,00E-012
reg_DEFB	chr8	7320465	C	T	61	77,00%	5of6	47								1,00E-012
reg_DEFB	chr8	7320516	A	G	56	75,00%	4of6	42							rs71308312	1,00E-012
reg_DEFB	chr8	7320524	C	G	57	77,00%	5of6	44							rs71308312	1,00E-012
reg_DEFB	chr8	7320543	A	G	57	72,00%	4of6	41								1,00E-012
reg_DEFB	chr8	7320547	A	G	59	75,00%	4of6	44								1,00E-012
reg_DEFB	chr8	7320699	G	A	58	88,00%	5of6	51							rs71272074	1,00E-012
reg_DEFB	chr8	7320772	A	C	50	72,00%	4of6	36					rs2680508			1,00E-012
reg_DEFB	chr8	7320916	A	G	66	95,00%	6of6	63							rs61413127	1,00E-012
reg_DEFB	chr8	7320949	G	A	76	68,00%	4of6	52					rs2740690			1,00E-012
reg_DEFB	chr8	7320963	C	T	79	19,00%	1of6	15								3,64E-010
reg_DEFB	chr8	7320971	C	G	81	21,00%	1of6	17					rs2680509			1,41E-011
reg_DEFB	chr8	7321565	A	G	47	13,00%	1of6	6					rs2739944			7,08E-004
reg_DEFB	chr8	7321575	T	A	46	57,00%	3of6	26					rs2739943			1,00E-012
reg_DEFB	chr8	7321621	C	T	46	57,00%	3of6	26							rs2680510	1,00E-012
reg_DEFB	chr8	7321847	G	T	76	13,00%	1of6	10								9,89E-006
reg_DEFB	chr8	7322011	C	T	69	10,00%	1of6	7								1,06E-003
reg_DEFB	chr8	7322171	G	A	72	57,00%	3of6	41							rs2680512	1,00E-012
reg_DEFB	chr8	7322389	A	G	54	74,00%	4of6	40							rs2680515	1,00E-012
reg_DEFB	chr8	7322414	C	T	49	67,00%	4of6	33							rs2680516	1,00E-012
reg_DEFB	chr8	7322468	T	C	39	31,00%	2of6	12								5,13E-011
reg_DEFB	chr8	7322839	C	A	31	65,00%	4of6	20					rs28689192			1,00E-012

reg_DEFB	chr8	7322882	A	C	32	97,00%	6of6	31							rs6472800	1,00E-012
reg_DEFB	chr8	7322889	C	T	32	100,00%	6of6	32							rs4602913	1,00E-012
reg_DEFB	chr8	7322954	C	G	49	100,00%	6of6	49					rs28695206			1,00E-012
reg_DEFB	chr8	7323020	A	T	68	74,00%	4of6	50							rs2739908	1,00E-012
reg_DEFB	chr8	7323118	A	G	99	89,00%	5of6	88					rs2737579			1,00E-012
reg_DEFB	chr8	7323294	A	G	114	31,00%	2of6	35					rs2739898			1,00E-012
reg_DEFB	chr8	7323491	T	G	98	15,00%	1of6	15					rs2739894			8,04E-009
reg_DEFB	chr8	7323584	G	A	107	20,00%	1of6	21							rs71247893	1,00E-012
reg_DEFB	chr8	7323601	G	A	102	13,00%	1of6	13							rs2680519	6,97E-007
reg_DEFB	chr8	7323645	G	C	100	34,00%	2of6	34							rs71247894	1,00E-012
reg_DEFB	chr8	7323679	C	T	105	93,00%	6of6	98					rs28557092	rs41405850		1,00E-012
reg_DEFB	chr8	7323747	T	G	107	14,00%	1of6	15					rs2739889			2,73E-008
reg_DEFB	chr8	7323827	T	C	102	16,00%	1of6	16							rs2737581	1,79E-009
reg_DEFB	chr8	7323880	G	A	100	15,00%	1of6	15							rs2680521	1,07E-008
reg_DEFB	chr8	7323982	C	T	105	16,00%	1of6	17								3,47E-010
reg_DEFB	chr8	7323985	G	A	107	15,00%	1of6	16					rs2680522			3,66E-009
reg_DEFB	chr8	7324031	C	A	116	32,00%	2of6	37					rs4840760			1,00E-012
reg_DEFB	chr8	7324145	G	A	101	15,00%	1of6	15							rs2680523	1,23E-008
reg_DEFB	chr8	7324233	A	T	108	44,00%	3of6	48					rs4467955			1,00E-012
reg_DEFB	chr8	7324614	A	G	80	16,00%	1of6	13					rs2680525			3,76E-008
reg_DEFB	chr8	7324635	T	A	81	11,00%	1of6	9					rs2740689			1,06E-004
reg_DEFB	chr8	7324672	C	T	77	10,00%	1of6	8					rs2680526			4,02E-004
reg_DEFB	chr8	7324797	T	G	58	14,00%	1of6	8							rs56122448	5,38E-005
reg_DEFB	chr8	7324845	T	G	64	14,00%	1of6	9							rs55754272	1,58E-005
reg_DEFB	chr8	7324880	C	T	65	11,00%	1of6	7					rs2954313			7,38E-004
reg_DEFB	chr8	7324941	G	C	69	12,00%	1of6	8					rs2977416			1,88E-004
reg_DEFB	chr8	7324973	G	A	67	10,00%	1of6	7							rs2740158	8,86E-004
reg_DEFB	chr8	7325028	A	G	81	75,00%	4of6	61					rs6651517			1,00E-012
reg_DEFB	chr8	7325133	A	G	109	20,00%	1of6	22					rs2680528			1,00E-012
reg_DEFB	chr8	7325219	A	G	114	18,00%	1of6	21							rs2740154	1,00E-012
reg_DEFB	chr8	7325548	T	C	81	14,00%	1of6	11					rs2737584			2,62E-006
reg_DEFB	chr8	7325767	T	C	106	38,00%	2of6	40							rs62639773	1,00E-012
reg_DEFB	chr8	7325835	A	T	103	15,00%	1of6	15								1,61E-008
reg_DEFB	chr8	7325839	C	T	105	13,00%	1of6	14								1,49E-007
reg_DEFB	chr8	7325875	A	G	102	31,00%	2of6	32					rs2740137			1,00E-012
reg_DEFB	chr8	7325879	A	C	100	16,00%	1of6	16					rs2680531			1,33E-009
reg_DEFB	chr8	7326033	G	C	93	12,00%	1of6	11					rs2737585			1,02E-005
reg_DEFB	chr8	7326060	C	T	89	55,00%	3of6	49					rs2740125			1,00E-012
reg_DEFB	chr8	7326067	C	G	86	13,00%	1of6	11					rs2740124			4,76E-006
reg_DEFB	chr8	7326472	G	A	115	19,00%	1of6	22					rs2680532			1,00E-012
reg_DEFB	chr8	7326494	A	G	119	20,00%	1of6	24					rs2740102			1,00E-012
reg_DEFB	chr8	7326592	T	C	104	22,00%	1of6	23							rs2737586	1,00E-012
reg_DEFB	chr8	7326628	C	T	92	99,00%	6of6	91							rs62639774	1,00E-012
reg_DEFB	chr8	7326698	C	T	60	18,00%	1of6	11					rs2737587			1,15E-007
reg_DEFB	chr8	7326715	A	G	46	24,00%	1of6	11					rs2979555			6,04E-009
reg_DEFB	chr8	7327125	A	G	37	22,00%	1of6	8					rs2980523			1,66E-006
reg_DEFB	chr8	7327254	C	T	61	26,00%	2of6	16					rs2740084			1,00E-012
reg_DEFB	chr8	7327310	T	A	68	22,00%	1of6	15					rs2680545			4,26E-011
reg_DEFB	chr8	7327328	G	A	72	100,00%	6of6	72					rs2293956			1,00E-012
reg_DEFB	chr8	7327333	G	A	71	99,00%	6of6	70								1,00E-012
reg_DEFB	chr8	7327614	A	G	108	12,00%	1of6	13	N	N	-2	DEFB106A	rs2293957			1,35E-006
reg_DEFB	chr8	7327688	C	T	103	12,00%	1of6	12			-	DEFB106B	rs2740082			4,81E-006
reg_DEFB	chr8	7327723	T	A	103	16,00%	1of6	16			-	DEFB106B				2,07E-009

reg_DEFB	chr8	7327733	A	G	107	34,00%	2of6	36			-	DEFB106B	rs2244098			1,00E-012
reg_DEFB	chr8	7327748	G	A	107	64,00%	4of6	68			-	DEFB106B	rs2244096			1,00E-012
reg_DEFB	chr8	7327863	C	T	92	15,00%	1of6	14			-	DEFB106B				2,71E-008
reg_DEFB	chr8	7328121	A	G	76	36,00%	2of6	27			-	DEFB106B	rs2243995			1,00E-012
reg_DEFB	chr8	7328248	C	T	95	21,00%	1of6	20			-	DEFB106B	rs2738002			1,00E-012
reg_DEFB	chr8	7328290	T	C	96	61,00%	4of6	59			-	DEFB106B	rs2740081			1,00E-012
reg_DEFB	chr8	7328305	C	A	95	34,00%	2of6	32			-	DEFB106B			rs62641365	1,00E-012
reg_DEFB	chr8	7328421	T	C	112	13,00%	1of6	15			-	DEFB106B	rs6984237			5,10E-008
reg_DEFB	chr8	7328517	A	G	107	15,00%	1of6	16			-	DEFB106B	rs2740078			3,66E-009
reg_DEFB	chr8	7328700	C	T	46	100,00%	6of6	46			-	DEFB106B	rs28548808			1,00E-012
reg_DEFB	chr8	7328712	T	C	46	37,00%	2of6	17			-	DEFB106B	rs2680547	rs28377333		1,00E-012
reg_DEFB	chr8	7328762	A	C	34	32,00%	2of6	11			-	DEFB106B	rs2680548			1,76E-010
reg_DEFB	chr8	7328769	T	G	31	16,00%	1of6	5			-	DEFB106B			rs72626625	6,64E-004
reg_DEFB	chr8	7329799	A	C	7	43,00%	3of6	3			-	DEFB106B	rs2737589			3,97E-004
reg_DEFB	chr8	7329944	A	T	13	85,00%	5of6	11			-	DEFB106B	rs2740058			1,00E-012
reg_DEFB	chr8	7330145	A	T	15	100,00%	6of6	15			-	DEFB106B				1,00E-012
reg_DEFB	chr8	7330373	G	C	33	21,00%	1of6	7			-	DEFB106B	rs2740039			8,59E-006
reg_DEFB	chr8	7330474	G	A	51	25,00%	1of6	13			-	DEFB106B	rs2740038			1,16E-010
reg_DEFB	chr8	7330551	T	C	58	45,00%	3of6	26			-	DEFB106B	rs6605634			1,00E-012
reg_DEFB	chr8	7330574	C	G	71	25,00%	1of6	18			-	DEFB106B			rs62639775	1,00E-012
reg_DEFB	chr8	7330881	T	A	102	28,00%	2of6	29			-	DEFB106B			rs62639776	1,00E-012
reg_DEFB	chr8	7331105	C	T	64	11,00%	1of6	7			-	DEFB106B	rs3748149			6,71E-004
reg_DEFB	chr8	7331216	C	G	61	69,00%	4of6	42			-	DEFB106B	rs2738000			1,00E-012
reg_DEFB	chr8	7331608	C	G	83	100,00%	6of6	83								1,00E-012
reg_DEFB	chr8	7331822	C	T	91	21,00%	1of6	19					rs6999662			1,00E-012
reg_DEFB	chr8	7331835	G	A	91	18,00%	1of6	16					rs2740027			3,18E-010
reg_DEFB	chr8	7332215	T	A	99	17,00%	1of6	17					rs6982591			1,33E-010
reg_DEFB	chr8	7332633	C	T	104	17,00%	1of6	18					rs2737997			4,09E-011
reg_DEFB	chr8	7332663	T	A	111	33,00%	2of6	37	I	N	1	DEFB105B			rs62639778	1,00E-012
reg_DEFB	chr8	7332749	T	C	119	20,00%	1of6	24			+	DEFB105A	rs2737996			1,00E-012
reg_DEFB	chr8	7333168	G	C	115	100,00%	6of6	115			+	DEFB105A				1,00E-012
reg_DEFB	chr8	7333507	T	C	125	36,00%	2of6	45			+	DEFB105A	rs2737994			1,00E-012
reg_DEFB	chr8	7333953	C	T	144	35,00%	2of6	50			+	DEFB105A	rs6422765	rs2737593		1,00E-012
reg_DEFB	chr8	7334564	C	G	105	36,00%	2of6	38							rs62639779	1,00E-012
reg_DEFB	chr8	7334688	T	C	62	19,00%	1of6	12							rs2737992	1,63E-008
reg_DEFB	chr8	7334804	C	T	56	20,00%	1of6	11					rs2737594			5,46E-008
reg_DEFB	chr8	7334844	G	C	60	35,00%	2of6	21							rs62639780	1,00E-012
reg_DEFB	chr8	7334996	A	G	112	83,00%	5of6	93					rs2737595			1,00E-012
reg_DEFB	chr8	7335115	C	T	138	18,00%	1of6	25					rs2737596			1,00E-012
reg_DEFB	chr8	7335121	T	C	137	100,00%	6of6	137								1,00E-012
reg_DEFB	chr8	7335165	C	G	145	17,00%	1of6	25					rs2737597			1,00E-012
reg_DEFB	chr8	7335196	A	G	147	16,00%	1of6	24					rs2737598			1,00E-012
reg_DEFB	chr8	7335401	A	G	134	99,00%	6of6	133					rs2977418			1,00E-012
reg_DEFB	chr8	7335410	T	C	136	18,00%	1of6	24					rs7462905			1,00E-012
reg_DEFB	chr8	7335573	T	C	123	33,00%	2of6	41					rs4481626			1,00E-012
reg_DEFB	chr8	7336339	A	T	108	13,00%	1of6	14					rs2737991			2,13E-007
reg_DEFB	chr8	7336570	C	T	61	18,00%	1of6	11							rs62639783	1,38E-007
reg_DEFB	chr8	7336640	T	G	44	30,00%	2of6	13					rs2737599			3,42E-011
reg_DEFB	chr8	7336834	A	G	19	21,00%	1of6	4					rs2737600			8,22E-004
reg_DEFB	chr8	7336931	A	C	26	58,00%	3of6	15					rs2737601			1,00E-012
reg_DEFB	chr8	7337241	C	T	74	19,00%	1of6	14					rs2737602			1,45E-009
reg_DEFB	chr8	7337388	G	A	95	18,00%	1of6	17								6,70E-011
reg_DEFB	chr8	7337863	A	C	51	86,00%	5of6	44								1,00E-012

reg_DEFB	chr8	7338251	A	C	86	26,00%	2of6	22					rs2737989		1,00E-012
reg_DEFB	chr8	7338279	T	C	88	99,00%	6of6	87					rs2946448		1,00E-012
reg_DEFB	chr8	7338381	C	T	94	100,00%	6of6	94					rs2977421		1,00E-012
reg_DEFB	chr8	7338534	G	T	72	68,00%	4of6	49					rs2737604		1,00E-012
reg_DEFB	chr8	7338703	C	T	72	24,00%	1of6	17					rs2737605		1,00E-012
reg_DEFB	chr8	7338734	C	T	77	61,00%	4of6	47					rs2680559		1,00E-012
reg_DEFB	chr8	7338884	A	G	98	89,00%	5of6	87						rs62639786	1,00E-012
reg_DEFB	chr8	7338975	G	A	105	17,00%	1of6	18							4,84E-011
reg_DEFB	chr8	7339053	T	G	108	18,00%	1of6	19						rs71236463	8,71E-012
reg_DEFB	chr8	7339159	G	T	117	12,00%	1of6	14					rs2680560		5,77E-007
reg_DEFB	chr8	7339447	C	T	123	98,00%	6of6	121						rs62639787	1,00E-012
reg_DEFB	chr8	7339485	C	T	124	60,00%	4of6	74					rs2680561		1,00E-012
reg_DEFB	chr8	7339502	A	G	130	15,00%	1of6	20							3,44E-011
reg_DEFB	chr8	7339538	C	G	137	100,00%	6of6	137					rs2737606		1,00E-012
reg_DEFB	chr8	7339593	A	G	142	20,00%	1of6	28						rs71236462	1,00E-012
reg_DEFB	chr8	7339872	A	G	137	69,00%	4of6	95					rs2737608		1,00E-012
reg_DEFB	chr8	7339927	A	C	130	70,00%	4of6	91					rs2737609		1,00E-012
reg_DEFB	chr8	7340022	T	C	108	33,00%	2of6	36					rs2737610		1,00E-012
reg_DEFB	chr8	7340195	C	A	68	65,00%	4of6	44					rs2737988		1,00E-012
reg_DEFB	chr8	7340261	C	G	54	19,00%	1of6	10						rs62639790	3,93E-007
reg_DEFB	chr8	7340654	G	A	68	15,00%	1of6	10					rs2737611		3,56E-006
reg_DEFB	chr8	7340708	T	C	82	16,00%	1of6	13						rs2680425	5,10E-008
reg_DEFB	chr8	7340710	G	A	82	100,00%	6of6	82							1,00E-012
reg_DEFB	chr8	7340890	G	T	95	26,00%	2of6	25	V	F	1	DEFB107A	rs2246582		1,00E-012
reg_DEFB	chr8	7341300	T	C	40	97,00%	6of6	39			+	DEFB107A	rs12682203		1,00E-012
reg_DEFB	chr8	7341374	C	G	39	41,00%	2of6	16			+	DEFB107A		rs62639791	1,00E-012
reg_DEFB	chr8	7341377	A	G	39	38,00%	2of6	15			+	DEFB107A		rs73366541	1,00E-012
reg_DEFB	chr8	7341389	G	T	36	17,00%	1of6	6			+	DEFB107A	rs12675434		1,59E-004
reg_DEFB	chr8	7341391	C	G	36	25,00%	1of6	9			+	DEFB107A	rs2737612		9,66E-008
reg_DEFB	chr8	7341618	G	T	106	20,00%	1of6	21			+	DEFB107A		rs62641362	1,00E-012
reg_DEFB	chr8	7341639	C	T	117	22,00%	1of6	26			+	DEFB107A		rs62641361	1,00E-012
reg_DEFB	chr8	7341834	G	C	196	15,00%	1of6	29			+	DEFB107A		rs71251575	1,00E-012
reg_DEFB	chr8	7342189	A	G	194	17,00%	1of6	33			+	DEFB107A	rs2737614		1,00E-012
reg_DEFB	chr8	7342213	G	C	197	17,00%	1of6	33			+	DEFB107A		rs73199782	1,00E-012
reg_DEFB	chr8	7342346	G	A	182	32,00%	2of6	58			+	DEFB107A		rs66911494	1,00E-012
reg_DEFB	chr8	7343575	C	T	3	100,00%	6of6	3			+	DEFB107A		rs4538904	1,22E-005
reg_DEFB	chr8	7343596	C	T	3	100,00%	6of6	3			+	DEFB107A		rs9774358	1,22E-005
reg_DEFB	chr8	7343651	C	T	3	100,00%	6of6	3			+	DEFB107A		rs35904342	1,22E-005
reg_DEFB	chr8	7343692	C	T	3	100,00%	6of6	3			+	DEFB107A		rs62515824	1,22E-005
reg_DEFB	chr8	7343714	C	T	3	100,00%	6of6	3			+	DEFB107A	rs9720857		1,22E-005
reg_DEFB	chr8	7346227	C	T	3	100,00%	6of6	3			+	DEFB107A			1,22E-005
reg_DEFB	chr8	7347990	G	A	7	71,00%	4of6	5			+	DEFB107A			1,30E-007
reg_DEFB	chr8	7348074	T	C	7	86,00%	5of6	6			+	DEFB107A			1,02E-009
reg_DEFB	chr8	7348206	C	T	11	27,00%	2of6	3			+	DEFB107A			1,75E-003
reg_DEFB	chr8	7348303	A	G	12	100,00%	6of6	12			+	DEFB107A			1,00E-012
reg_DEFB	chr8	7348338	C	A	11	100,00%	6of6	11			+	DEFB107A			1,00E-012
reg_DEFB	chr8	7348349	T	C	10	50,00%	3of6	5			+	DEFB107A			1,47E-006
reg_DEFB	chr8	7348357	A	G	11	73,00%	4of6	8			+	DEFB107A			1,56E-011
reg_DEFB	chr8	7348411	A	C	9	44,00%	3of6	4			+	DEFB107A			3,21E-005
reg_DEFB	chr8	7348467	C	A	6	67,00%	4of6	4			+	DEFB107A			4,04E-006
reg_DEFB	chr8	7352319	C	G	40	32,00%	2of6	13			+	DEFB107A		rs72494253	8,21E-012
reg_DEFB	chr8	7352334	T	C	51	33,00%	2of6	17			+	DEFB107A		rs73199783	1,00E-012
reg_DEFB	chr8	7352362	G	T	59	54,00%	3of6	32			+	DEFB107A		rs2737986	1,00E-012

reg_DEFB	chr8	7352774	T	A	112	14,00%	1of6	16	+	DEFB107A	rs2737616		7,20E-009
reg_DEFB	chr8	7352969	T	C	91	98,00%	6of6	89	+	DEFB107A	rs12155828		1,00E-012
reg_DEFB	chr8	7353013	A	G	108	45,00%	3of6	49	+	DEFB107A		rs2737984	1,00E-012
reg_DEFB	chr8	7353015	A	G	109	92,00%	5of6	100	+	DEFB107A		rs72329447	1,00E-012
reg_DEFB	chr8	7353162	C	T	127	26,00%	2of6	33	+	DEFB107A		rs2737474	1,00E-012
reg_DEFB	chr8	7353297	A	C	149	13,00%	1of6	19	+	DEFB107A		rs71511272	2,07E-009
reg_DEFB	chr8	7353385	A	G	154	42,00%	2of6	65	+	DEFB107A	rs2737983		1,00E-012
reg_DEFB	chr8	7353437	A	G	145	19,00%	1of6	28	+	DEFB107A	rs2737475		1,00E-012
reg_DEFB	chr8	7353557	C	T	135	33,00%	2of6	45	+	DEFB107A	rs11775409		1,00E-012
reg_DEFB	chr8	7353946	C	T	108	39,00%	2of6	42	+	DEFB107A		rs62639796	1,00E-012
reg_DEFB	chr8	7353996	T	C	110	46,00%	3of6	51	+	DEFB107A	rs2737476		1,00E-012
reg_DEFB	chr8	7354227	T	C	140	77,00%	5of6	108	+	DEFB107A	rs2737477		1,00E-012
reg_DEFB	chr8	7354364	A	G	153	33,00%	2of6	50			rs4143089		1,00E-012
reg_DEFB	chr8	7354376	T	C	154	32,00%	2of6	49			rs4143090		1,00E-012
reg_DEFB	chr8	7354417	T	A	152	18,00%	1of6	27			rs2680430		1,00E-012
reg_DEFB	chr8	7354477	G	A	150	27,00%	2of6	41			rs4143091		1,00E-012
reg_DEFB	chr8	7354592	A	T	151	15,00%	1of6	23				rs2737478	4,21E-012
reg_DEFB	chr8	7354673	C	G	132	80,00%	5of6	106				rs62639797	1,00E-012
reg_DEFB	chr8	7354872	T	G	121	13,00%	1of6	16			rs2737479		2,23E-008
reg_DEFB	chr8	7355739	G	A	75	100,00%	6of6	75				rs71511273	1,00E-012
reg_DEFB	chr8	7356482	G	C	88	15,00%	1of6	13				rs71511274	1,21E-007
reg_DEFB	chr8	7357088	T	C	147	46,00%	3of6	68			rs2737481		1,00E-012
reg_DEFB	chr8	7357411	A	C	147	95,00%	6of6	140			rs4840763		1,00E-012
reg_DEFB	chr8	7357553	G	A	173	51,00%	3of6	88			rs2737979		1,00E-012
reg_DEFB	chr8	7357665	T	G	175	16,00%	1of6	28			rs2737482		1,00E-012
reg_DEFB	chr8	7357798	C	T	175	34,00%	2of6	60				rs2737483	1,00E-012
reg_DEFB	chr8	7357880	T	C	164	96,00%	6of6	157				rs71509114	1,00E-012
reg_DEFB	chr8	7357983	G	A	173	32,00%	2of6	55				rs2737978	1,00E-012
reg_DEFB	chr8	7358487	A	G	164	32,00%	2of6	52			rs2737977		1,00E-012
reg_DEFB	chr8	7358618	C	T	181	98,00%	6of6	177					1,00E-012
reg_DEFB	chr8	7358904	G	A	148	100,00%	6of6	148				rs62639798	1,00E-012
reg_DEFB	chr8	7358942	C	T	131	38,00%	2of6	50			rs2680433		1,00E-012
reg_DEFB	chr8	7359000	G	A	111	100,00%	6of6	111			rs11984588		1,00E-012
reg_DEFB	chr8	7359515	C	A	66	100,00%	6of6	66				rs71239493	1,00E-012
reg_DEFB	chr8	7359657	T	G	70	100,00%	6of6	70				rs62639800	1,00E-012
reg_DEFB	chr8	7359721	C	T	61	16,00%	1of6	10				rs62640758	1,28E-006
reg_DEFB	chr8	7360225	G	A	51	45,00%	3of6	23				rs2680434	1,00E-012
reg_DEFB	chr8	7360474	C	T	43	14,00%	1of6	6			rs2737487		4,35E-004
reg_DEFB	chr8	7360635	T	G	82	16,00%	1of6	13				rs71259275	5,10E-008
reg_DEFB	chr8	7361108	A	G	72	31,00%	2of6	22				rs62639801	1,00E-012
reg_DEFB	chr8	7361125	T	C	76	32,00%	2of6	24				rs62639802	1,00E-012
reg_DEFB	chr8	7361697	C	T	80	17,00%	1of6	14				rs67213127	4,20E-009
reg_DEFB	chr8	7361701	G	T	82	49,00%	3of6	40				rs62639803	1,00E-012
reg_DEFB	chr8	7361824	G	T	60	18,00%	1of6	11				rs71268643	1,15E-007
reg_DEFB	chr8	7361829	G	T	60	17,00%	1of6	10				rs71268643	1,09E-006
reg_DEFB	chr8	7361937	C	T	61	49,00%	3of6	30			rs4840765		1,00E-012
reg_DEFB	chr8	7361941	A	G	61	15,00%	1of6	9					1,06E-005
reg_DEFB	chr8	7362037	C	G	50	14,00%	1of6	7				rs66478539	1,43E-004
reg_DEFB	chr8	7362076	A	G	47	30,00%	2of6	14				rs71230560	4,96E-012
reg_DEFB	chr8	7362278	G	A	31	16,00%	1of6	5				rs71230561	6,64E-004
reg_DEFB	chr8	7362617	A	G	30	80,00%	5of6	24				rs71213914	1,00E-012
reg_DEFB	chr8	7362978	C	T	36	36,00%	2of6	13				rs62639805	1,00E-012
reg_DEFB	chr8	7362993	C	T	37	30,00%	2of6	11			rs2680436		4,70E-010

reg_DEFB	chr8	7363131	C	T	25	24,00%	1of6	6		rs66602902	1,80E-005
reg_DEFB	chr8	7363844	A	T	77	38,00%	2of6	29		rs2737488	1,00E-012
reg_DEFB	chr8	7363941	A	G	87	37,00%	2of6	32		rs2680438	1,00E-012
reg_DEFB	chr8	7364133	G	A	111	14,00%	1of6	16	rs2680439		6,31E-009
reg_DEFB	chr8	7364280	A	G	107	14,00%	1of6	15		rs71228231	2,73E-008
reg_DEFB	chr8	7364478	C	G	154	71,00%	4of6	109	rs2680440		1,00E-012
reg_DEFB	chr8	7364562	G	T	149	20,00%	1of6	30		rs4590459	1,00E-012
reg_DEFB	chr8	7364725	T	A	139	17,00%	1of6	24	rs4556104		1,00E-012
reg_DEFB	chr8	7364726	T	C	140	21,00%	1of6	29			1,00E-012
reg_DEFB	chr8	7365153	T	C	139	17,00%	1of6	24		rs71228230	1,00E-012
reg_DEFB	chr8	7365196	A	G	138	18,00%	1of6	25		rs67575026	1,00E-012
reg_DEFB	chr8	7365483	G	C	119	10,00%	1of6	12			2,13E-005
reg_DEFB	chr8	7365664	C	A	125	25,00%	1of6	31		rs71511277	1,00E-012
reg_DEFB	chr8	7365938	G	A	129	15,00%	1of6	19		rs71511278	1,78E-010
reg_DEFB	chr8	7366130	G	T	137	15,00%	1of6	21		rs71511279	1,19E-011
reg_DEFB	chr8	7366324	G	A	115	17,00%	1of6	20	rs4584163		8,76E-012
reg_DEFB	chr8	7366488	A	G	74	12,00%	1of6	9	rs4263787		5,16E-005
reg_DEFB	chr8	7366494	A	C	73	49,00%	3of6	36	rs4270988		1,00E-012
reg_DEFB	chr8	7366516	C	G	71	13,00%	1of6	9	rs4446761		3,70E-005
reg_DEFB	chr8	7366531	T	G	66	12,00%	1of6	8	rs4335141		1,37E-004
reg_DEFB	chr8	7367473	C	G	103	20,00%	1of6	21	rs4440657		1,00E-012
reg_DEFB	chr8	7367494	C	G	101	29,00%	2of6	29			1,00E-012
reg_DEFB	chr8	7367539	G	C	95	29,00%	2of6	28			1,00E-012
reg_DEFB	chr8	7367542	G	T	94	26,00%	2of6	24			1,00E-012
reg_DEFB	chr8	7367818	T	C	21	90,00%	5of6	19		rs7842766	1,00E-012
reg_DEFB	chr8	7367967	G	A	18	22,00%	1of6	4			6,61E-004
reg_DEFB	chr8	7368321	C	T	111	12,00%	1of6	13	rs2737964		1,85E-006
reg_DEFB	chr8	7368407	T	G	130	19,00%	1of6	25		rs4599836	1,00E-012
reg_DEFB	chr8	7368542	T	A	149	63,00%	4of6	94		rs71511292	1,00E-012
reg_DEFB	chr8	7368771	A	T	102	30,00%	2of6	31	rs2737491		1,00E-012
reg_DEFB	chr8	7369708	C	T	148	71,00%	4of6	105	rs725058		1,00E-012
reg_DEFB	chr8	7369907	A	G	149	16,00%	1of6	24	rs2680485		1,00E-012
reg_DEFB	chr8	7369959	G	A	138	28,00%	2of6	39	rs725057		1,00E-012
reg_DEFB	chr8	7370028	T	G	122	32,00%	2of6	39	rs2680484		1,00E-012
reg_DEFB	chr8	7370074	C	A	113	29,00%	2of6	33		rs62639806	1,00E-012
reg_DEFB	chr8	7370374	G	C	89	28,00%	2of6	25	rs2737961		1,00E-012
reg_DEFB	chr8	7370610	A	G	131	64,00%	4of6	84		rs2680482	1,00E-012
reg_DEFB	chr8	7371168	C	T	83	19,00%	1of6	16	rs1002943		7,48E-011
reg_DEFB	chr8	7371271	C	A	84	31,00%	2of6	26	rs2737492		1,00E-012
reg_DEFB	chr8	7371475	C	T	78	71,00%	4of6	55	rs4840766		1,00E-012
reg_DEFB	chr8	7371487	C	A	73	30,00%	2of6	22	rs2737493		1,00E-012
reg_DEFB	chr8	7372029	C	T	67	13,00%	1of6	9		rs71264712	2,31E-005
reg_DEFB	chr8	7372124	C	T	79	28,00%	2of6	22	rs2977691		1,00E-012
reg_DEFB	chr8	7372488	C	G	155	15,00%	1of6	23		rs71264713	7,44E-012
reg_DEFB	chr8	7373102	G	A	73	22,00%	1of6	16	rs2977399		1,07E-011
reg_DEFB	chr8	7373254	C	T	47	55,00%	3of6	26	rs1807385		1,00E-012
reg_DEFB	chr8	7373360	A	T	29	21,00%	1of6	6		rs1985804	4,46E-005
reg_DEFB	chr8	7374510	C	T	79	18,00%	1of6	14		rs71249119	3,54E-009
reg_DEFB	chr8	7374904	G	A	68	22,00%	1of6	15	rs2977690		4,26E-011
reg_DEFB	chr8	7375439	G	A	90	59,00%	4of6	53		rs71249120	1,00E-012
reg_DEFB	chr8	7375518	G	A	70	43,00%	3of6	30	rs4840769		1,00E-012
reg_DEFB	chr8	7375596	C	A	81	38,00%	2of6	31	rs11786478		1,00E-012
reg_DEFB	chr8	7375735	G	T	82	65,00%	4of6	53		rs71249122	1,00E-012

reg_DEFB	chr8	7375812	G	A	82	50,00%	3of6	41			rs71264915	1,00E-012
reg_DEFB	chr8	7375818	C	T	81	36,00%	2of6	29			rs71264915	1,00E-012
reg_DEFB	chr8	7375977	G	T	79	59,00%	4of6	47	rs2737951			1,00E-012
reg_DEFB	chr8	7375987	A	G	78	58,00%	3of6	45	rs2737950			1,00E-012
reg_DEFB	chr8	7376096	T	A	71	48,00%	3of6	34	rs2680503			1,00E-012
reg_DEFB	chr8	7376598	C	T	119	27,00%	2of6	32	rs2737949			1,00E-012
reg_DEFB	chr8	7377165	A	G	56	70,00%	4of6	39			rs71249124	1,00E-012
reg_DEFB	chr8	7377439	G	A	101	35,00%	2of6	35	rs2680501			1,00E-012
reg_DEFB	chr8	7377464	C	A	101	99,00%	6of6	100				1,00E-012
reg_DEFB	chr8	7377536	G	C	95	33,00%	2of6	31	rs2737947			1,00E-012
reg_DEFB	chr8	7378654	T	C	81	96,00%	6of6	78			rs71249125	1,00E-012
reg_DEFB	chr8	7379026	G	A	66	98,00%	6of6	65			rs67847292	1,00E-012
reg_DEFB	chr8	7379048	T	C	61	98,00%	6of6	60			rs71249126	1,00E-012
reg_DEFB	chr8	7380428	G	A	79	99,00%	6of6	78				1,00E-012
reg_DEFB	chr8	7381443	A	G	128	44,00%	3of6	56	rs2737940			1,00E-012
reg_DEFB	chr8	7381476	G	A	124	60,00%	4of6	74	rs2737939			1,00E-012
reg_DEFB	chr8	7381694	T	C	84	25,00%	1of6	21	rs2737498			1,00E-012
reg_DEFB	chr8	7381963	G	A	52	19,00%	1of6	10	rs4311672			2,71E-007
reg_DEFB	chr8	7382091	A	C	55	49,00%	3of6	27	rs2737936			1,00E-012
reg_DEFB	chr8	7382305	C	G	82	65,00%	4of6	53	rs2737499			1,00E-012
reg_DEFB	chr8	7382355	G	A	89	16,00%	1of6	14	rs2737935			1,75E-008
reg_DEFB	chr8	7382367	G	C	91	15,00%	1of6	14				2,35E-008
reg_DEFB	chr8	7382406	C	A	102	34,00%	2of6	35	rs4461922			1,00E-012
reg_DEFB	chr8	7382462	C	T	105	33,00%	2of6	35	rs4633079			1,00E-012
reg_DEFB	chr8	7382473	T	G	106	32,00%	2of6	34			rs62639815	1,00E-012
reg_DEFB	chr8	7382556	T	C	118	31,00%	2of6	37			rs62639816	1,00E-012
reg_DEFB	chr8	7382833	G	A	154	99,00%	6of6	152				1,00E-012
reg_DEFB	chr8	7383272	A	C	134	20,00%	1of6	27			rs67373661	1,00E-012
reg_DEFB	chr8	7383406	C	T	128	34,00%	2of6	44	rs4504661			1,00E-012
reg_DEFB	chr8	7383498	T	C	124	21,00%	1of6	26	rs4392927			1,00E-012
reg_DEFB	chr8	7383669	A	G	125	15,00%	1of6	19			rs66747636	1,02E-010
reg_DEFB	chr8	7383694	G	A	126	19,00%	1of6	24				1,00E-012
reg_DEFB	chr8	7384348	A	C	184	49,00%	3of6	90	rs2737932			1,00E-012
reg_DEFB	chr8	7384398	C	A	178	29,00%	2of6	52				1,00E-012
reg_DEFB	chr8	7384978	T	C	101	54,00%	3of6	55	rs2680541			1,00E-012
reg_DEFB	chr8	7385749	G	A	56	39,00%	2of6	22	rs2737931			1,00E-012
reg_DEFB	chr8	7386060	T	A	54	35,00%	2of6	19			rs62639820	1,00E-012
reg_DEFB	chr8	7386182	T	G	54	52,00%	3of6	28	rs2737502			1,00E-012
reg_DEFB	chr8	7386319	T	A	46	37,00%	2of6	17			rs62639821	1,00E-012
reg_DEFB	chr8	7386388	T	C	42	21,00%	1of6	9	rs2017768			4,04E-007
reg_DEFB	chr8	7386396	G	A	42	45,00%	3of6	19			rs62639822	1,00E-012
reg_DEFB	chr8	7386641	T	C	35	57,00%	3of6	20	rs2737503			1,00E-012
reg_DEFB	chr8	7386669	A	G	42	36,00%	2of6	15			rs62639824	1,00E-012
reg_DEFB	chr8	7386904	T	C	98	11,00%	1of6	11				1,69E-005
reg_DEFB	chr8	7386935	A	G	103	28,00%	2of6	29			rs62639825	1,00E-012
reg_DEFB	chr8	7387038	C	A	124	28,00%	2of6	35	rs3175182			1,00E-012
reg_DEFB	chr8	7387256	A	G	142	13,00%	1of6	18	rs1054672			5,98E-009
reg_DEFB	chr8	7387328	T	G	133	29,00%	2of6	39	rs4840284			1,00E-012
reg_DEFB	chr8	7387420	C	G	131	51,00%	3of6	67	rs2258428			1,00E-012
reg_DEFB	chr8	7387897	G	A	32	19,00%	1of6	6	rs2737506			8,02E-005
reg_DEFB	chr8	7387908	G	A	32	53,00%	3of6	17	rs2737507			1,00E-012
reg_DEFB	chr8	7388003	A	G	26	31,00%	2of6	8	rs2737508			8,44E-008
reg_DEFB	chr8	7388670	T	C	51	22,00%	1of6	11	rs2737511	rs2680539		1,94E-008

reg_DEFB	chr8	7388966	G	C	40	22,00%	1of6	9								2,58E-007
reg_DEFB	chr8	7389499	A	G	80	59,00%	4of6	47					rs2075896			1,00E-012
reg_DEFB	chr8	7389589	G	A	82	43,00%	3of6	35					rs2737512			1,00E-012
reg_DEFB	chr8	7389644	G	T	98	32,00%	2of6	31								1,00E-012
reg_DEFB	chr8	7390205	A	G	123	19,00%	1of6	23					rs2737513			1,00E-012
reg_DEFB	chr8	7390502	G	A	136	34,00%	2of6	46					rs2737514	rs4840775		1,00E-012
reg_DEFB	chr8	7390503	C	G	137	28,00%	2of6	38					rs4840775			1,00E-012
reg_DEFB	chr8	7390657	G	A	125	30,00%	2of6	38							rs2737889	1,00E-012
reg_DEFB	chr8	7390776	G	A	123	80,00%	5of6	98					rs2737515			1,00E-012
reg_DEFB	chr8	7390893	A	T	112	37,00%	2of6	41							rs62639831	1,00E-012
reg_DEFB	chr8	7391064	T	G	73	100,00%	6of6	73					rs4840776			1,00E-012
reg_DEFB	chr8	7391191	T	C	25	68,00%	4of6	17							rs71511284	1,00E-012
reg_DEFB	chr8	7391199	G	C	22	14,00%	1of6	3					rs2737885			1,35E-002
reg_CTRL	chr8	8214235	A	G	23	57,00%	het	13			-	PRAGMIN	rs6601691			1,00E-012
reg_CTRL	chr8	8214527	A	G	48	52,00%	het	25			-	PRAGMIN	rs6996347			1,00E-012
reg_CTRL	chr8	8214897	A	C	43	60,00%	het	26			-	PRAGMIN	rs4840922			1,00E-012
reg_CTRL	chr8	8215271	G	A	24	62,00%	het	15			-	PRAGMIN	rs2980503			1,00E-012
reg_CTRL	chr8	8215339	T	C	21	14,00%	ambig	3			-	PRAGMIN				1,19E-002
reg_CTRL	chr8	8215510	T	C	33	55,00%	het	18			-	PRAGMIN	rs13276576			1,00E-012
reg_CTRL	chr8	8215633	T	C	37	100,00%	homvar	37			-	PRAGMIN	rs1548198			1,00E-012
reg_CTRL	chr8	8217059	C	G	30	67,00%	het	20			-	PRAGMIN	rs4840923			1,00E-012
reg_CTRL	chr8	8218156	G	A	28	61,00%	het	17			-	PRAGMIN	rs12674854			1,00E-012
reg_CTRL	chr8	8218440	T	C	14	21,00%	ambig	3			-	PRAGMIN				3,66E-003
reg_CTRL	chr8	8218471	T	C	17	18,00%	ambig	3			-	PRAGMIN				6,50E-003
reg_CTRL	chr8	8218520	A	G	19	16,00%	ambig	3			-	PRAGMIN				8,95E-003
reg_CTRL	chr8	8218928	G	C	43	51,00%	het	22			-	PRAGMIN	rs867757			1,00E-012
reg_CTRL	chr8	8218968	A	T	41	49,00%	het	20			-	PRAGMIN	rs2945898			1,00E-012
reg_CTRL	chr8	8219680	G	C	19	63,00%	het	12			-	PRAGMIN	rs939069			1,00E-012
reg_CTRL	chr8	8223156	T	C	12	50,00%	het	6	H	R	-2	PRAGMIN			rs56215812	1,21E-007
reg_CTRL	chr8	8223577	T	C	62	35,00%	het	22			-	PRAGMIN	rs2979208			1,00E-012
reg_CTRL	chr8	8223747	A	T	52	40,00%	het	21			-	PRAGMIN	rs2976910			1,00E-012
reg_CTRL	chr8	8223842	C	T	39	54,00%	het	21			-	PRAGMIN	rs10099374			1,00E-012
reg_CTRL	chr8	8224229	G	T	13	31,00%	het	4			-	PRAGMIN	rs2945900			1,69E-004
reg_CTRL	chr8	8224240	G	A	15	20,00%	ambig	3			-	PRAGMIN				4,50E-003
reg_CTRL	chr8	8224259	T	C	15	27,00%	het	4			-	PRAGMIN				3,12E-004
reg_CTRL	chr8	8224912	A	G	40	30,00%	het	12			-	PRAGMIN	rs2979210			7,20E-011
reg_CTRL	chr8	8224960	T	C	34	35,00%	het	12			-	PRAGMIN	rs2945901			1,59E-011
reg_CTRL	chr8	8225163	A	G	26	42,00%	het	11			-	PRAGMIN	rs2980498	rs2945902		9,51E-012
reg_CTRL	chr8	8225164	T	C	24	42,00%	het	10			-	PRAGMIN	rs2945902			6,17E-011
reg_CTRL	chr8	8225230	T	C	21	52,00%	het	11			-	PRAGMIN	rs2945903			1,00E-012
reg_CTRL	chr8	8225641	G	A	29	48,00%	het	14			-	PRAGMIN	rs2979211			1,00E-012
reg_CTRL	chr8	8225874	G	A	31	39,00%	het	12			-	PRAGMIN	rs2979212			4,09E-012
reg_CTRL	chr8	8225954	G	A	44	45,00%	het	20			-	PRAGMIN	rs2979213			1,00E-012
reg_CTRL	chr8	8225986	A	G	45	51,00%	het	23			-	PRAGMIN	rs2976917			1,00E-012
reg_CTRL	chr8	8226005	A	G	45	42,00%	het	19			-	PRAGMIN	rs2976918			1,00E-012
reg_CTRL	chr8	8226193	A	G	54	50,00%	het	27			-	PRAGMIN	rs2979215			1,00E-012
reg_CTRL	chr8	8226315	T	C	66	47,00%	het	31			-	PRAGMIN	rs2979216			1,00E-012
reg_CTRL	chr8	8226494	G	A	71	55,00%	het	39			-	PRAGMIN	rs2979217			1,00E-012
reg_CTRL	chr8	8226774	G	C	55	53,00%	het	29			-	PRAGMIN	rs2979218			1,00E-012
reg_CTRL	chr8	8226802	G	A	55	56,00%	het	31			-	PRAGMIN	rs2979219			1,00E-012
reg_CTRL	chr8	8226809	A	G	56	57,00%	het	32			-	PRAGMIN	rs2976927			1,00E-012
reg_CTRL	chr8	8226848	T	C	58	100,00%	homvar	58			-	PRAGMIN	rs2980497			1,00E-012
reg_CTRL	chr8	8226912	G	A	48	60,00%	het	29			-	PRAGMIN	rs2945904			1,00E-012

reg_CTRL	chr8	8226996	T	G	44	61,00%	het	27	-	PRAGMIN	rs2945905			1,00E-012
reg_CTRL	chr8	8227475	T	C	35	43,00%	het	15	-	PRAGMIN	rs2945907			1,00E-012
reg_CTRL	chr8	8227809	T	G	62	48,00%	het	30	-	PRAGMIN	rs1518992			1,00E-012
reg_CTRL	chr8	8227825	T	C	63	48,00%	het	30	-	PRAGMIN	rs1850724			1,00E-012
reg_CTRL	chr8	8227900	C	G	70	39,00%	het	27	-	PRAGMIN	rs1850725			1,00E-012
reg_CTRL	chr8	8227975	A	G	67	43,00%	het	29	-	PRAGMIN	rs1850726			1,00E-012
reg_CTRL	chr8	8228199	T	C	48	54,00%	het	26	-	PRAGMIN	rs2980496			1,00E-012
reg_CTRL	chr8	8228481	T	G	64	50,00%	het	32	-	PRAGMIN	rs2979220			1,00E-012
reg_CTRL	chr8	8229144	C	T	25	32,00%	het	8	-	PRAGMIN	rs2980495			5,97E-008
reg_CTRL	chr8	8229293	T	C	19	37,00%	het	7	-	PRAGMIN	rs2979221			1,34E-007
reg_CTRL	chr8	8229301	G	A	18	39,00%	het	7	-	PRAGMIN	rs2976947			8,67E-008
reg_CTRL	chr8	8229639	A	C	38	45,00%	het	17	-	PRAGMIN	rs2945908			1,00E-012
reg_CTRL	chr8	8229935	T	C	32	53,00%	het	17	-	PRAGMIN	rs2976952			1,00E-012
reg_CTRL	chr8	8230241	T	C	35	40,00%	het	14	-	PRAGMIN	rs2976954			1,00E-012
reg_CTRL	chr8	8230250	A	C	35	40,00%	het	14	-	PRAGMIN	rs2979222			1,00E-012
reg_CTRL	chr8	8230631	T	C	17	41,00%	het	7	-	PRAGMIN	rs2980494			5,41E-008
reg_CTRL	chr8	8230864	G	A	28	100,00%	homvar	28	-	PRAGMIN	rs2979223			1,00E-012
reg_CTRL	chr8	8231138	C	T	47	45,00%	het	21	-	PRAGMIN	rs2979224			1,00E-012
reg_CTRL	chr8	8231195	T	G	50	42,00%	het	21	-	PRAGMIN	rs2976963			1,00E-012
reg_CTRL	chr8	8232030	A	C	14	57,00%	het	8	-	PRAGMIN	rs2979226			2,09E-010
reg_CTRL	chr8	8232142	T	G	21	62,00%	het	13	-	PRAGMIN	rs9329268	rs9329269		1,00E-012
reg_CTRL	chr8	8232143	T	C	23	65,00%	het	15	-	PRAGMIN	rs9329269			1,00E-012
reg_CTRL	chr8	8232156	C	G	25	60,00%	het	15	-	PRAGMIN	rs9329270			1,00E-012
reg_CTRL	chr8	8232408	G	A	52	54,00%	het	28	-	PRAGMIN	rs2976840			1,00E-012
reg_CTRL	chr8	8232580	A	C	36	50,00%	het	18	-	PRAGMIN	rs2945910			1,00E-012
reg_CTRL	chr8	8233348	C	T	66	41,00%	het	27	-	PRAGMIN	rs2976852			1,00E-012
reg_CTRL	chr8	8233599	T	C	83	35,00%	het	29	-	PRAGMIN	rs2980491			1,00E-012
reg_CTRL	chr8	8235132	A	G	89	51,00%	het	45	-	PRAGMIN	rs2945912			1,00E-012
reg_CTRL	chr8	8235635	C	T	63	51,00%	het	32	-	PRAGMIN	rs2945913			1,00E-012
reg_CTRL	chr8	8235716	C	G	55	55,00%	het	30	-	PRAGMIN	rs4840337			1,00E-012
reg_CTRL	chr8	8235760	T	G	55	44,00%	het	24	-	PRAGMIN	rs2980490			1,00E-012
reg_CTRL	chr8	8236281	C	T	63	38,00%	het	24	-	PRAGMIN	rs2976887			1,00E-012
reg_CTRL	chr8	8236848	A	C	8	100,00%	homvar	8	-	PRAGMIN	rs2945914			1,00E-012
reg_CTRL	chr8	8237074	T	C	5	60,00%	het	3	-	PRAGMIN			rs67646927	1,18E-004
reg_CTRL	chr8	8237374	G	A	37	46,00%	het	17	-	PRAGMIN			rs62496027	1,00E-012
reg_CTRL	chr8	8237387	A	G	38	11,00%	ambig	4	-	PRAGMIN				1,11E-002
reg_CTRL	chr8	8238677	A	G	24	100,00%	homvar	24	-	PRAGMIN	rs2176631			1,00E-012
reg_CTRL	chr8	8238782	A	G	28	61,00%	het	17	-	PRAGMIN	rs2945839			1,00E-012
reg_CTRL	chr8	8239704	A	G	21	33,00%	het	7	-	PRAGMIN	rs2980489			2,98E-007
reg_CTRL	chr8	8240257	T	C	21	52,00%	het	11	-	PRAGMIN				1,00E-012
reg_CTRL	chr8	8240551	C	A	11	27,00%	het	3	-	PRAGMIN				1,75E-003
reg_CTRL	chr8	8240669	T	C	23	17,00%	ambig	4	-	PRAGMIN				1,75E-003
reg_CTRL	chr8	8242420	C	T	56	98,00%	homvar	55	-	PRAGMIN	rs11785239			1,00E-012
reg_CTRL	chr8	8242644	A	T	42	62,00%	het	26	-	PRAGMIN	rs13273161			1,00E-012
reg_CTRL	chr8	8243226	C	T	67	100,00%	homvar	67	-	PRAGMIN	rs6990504			1,00E-012
reg_CTRL	chr8	8244163	A	G	21	76,00%	homvar	16	-	PRAGMIN	rs7833103			1,00E-012
reg_CTRL	chr8	8244364	G	A	11	64,00%	het	7	-	PRAGMIN				1,04E-009
reg_CTRL	chr8	8244749	A	G	42	52,00%	het	22	-	PRAGMIN	rs17150353			1,00E-012
reg_CTRL	chr8	8246169	G	C	37	57,00%	het	21	-	PRAGMIN	rs11786306			1,00E-012
reg_CTRL	chr8	8246601	G	T	92	99,00%	homvar	91	-	PRAGMIN	rs4840932			1,00E-012
reg_CTRL	chr8	8249567	T	C	29	55,00%	het	16	-	PRAGMIN	rs34796521			1,00E-012
reg_CTRL	chr8	8252759	T	C	42	43,00%	het	18	-	PRAGMIN	rs4840939			1,00E-012
reg_CTRL	chr8	8254396	A	G	51	100,00%	homvar	51	-	PRAGMIN	rs4840941			1,00E-012

reg_CTRL	chr8	8254926	C	T	36	53,00%	het	19			-	PRAGMIN	rs11778125			1,00E-012
reg_CTRL	chr8	8256007	T	C	36	97,00%	homvar	35			-	PRAGMIN	rs7005904			1,00E-012
reg_CTRL	chr8	8256108	G	C	29	52,00%	het	15			-	PRAGMIN	rs10099225			1,00E-012
reg_CTRL	chr8	8256279	T	C	19	26,00%	het	5			-	PRAGMIN	rs7006376			5,71E-005
reg_CTRL	chr8	8256377	A	G	21	95,00%	homvar	20			-	PRAGMIN	rs4840338			1,00E-012
reg_CTRL	chr8	8256592	G	A	60	100,00%	homvar	60			-	PRAGMIN	rs724265			1,00E-012
reg_CTRL	chr8	8256759	G	A	80	46,00%	het	37			-	PRAGMIN	rs724266			1,00E-012
reg_CTRL	chr8	8257851	T	C	16	62,00%	het	10			-	PRAGMIN			rs71513189	1,00E-012
reg_CTRL	chr8	8258498	C	T	17	59,00%	het	10			-	PRAGMIN	rs1914826			1,00E-012
reg_CTRL	chr8	8258721	C	G	29	48,00%	het	14			-	PRAGMIN	rs1914825			1,00E-012
reg_CTRL	chr8	8258769	G	A	33	45,00%	het	15			-	PRAGMIN	rs1914824			1,00E-012
reg_CTRL	chr8	8259602	T	G	86	45,00%	het	39			-	PRAGMIN	rs11996133			1,00E-012
reg_CTRL	chr8	8259890	T	C	65	100,00%	homvar	65			-	PRAGMIN	rs2030279			1,00E-012
reg_CTRL	chr8	8261308	T	C	23	57,00%	het	13			-	PRAGMIN	rs2979237			1,00E-012
reg_CTRL	chr8	8261611	A	C	21	62,00%	het	13			-	PRAGMIN			rs36109453	1,00E-012
reg_CTRL	chr8	8261787	T	A	24	58,00%	het	14			-	PRAGMIN	rs34628823			1,00E-012
reg_CTRL	chr8	8261903	C	T	32	56,00%	het	18			-	PRAGMIN	rs35038563			1,00E-012
reg_CTRL	chr8	8264586	C	A	58	57,00%	het	33			-	PRAGMIN	rs7841735			1,00E-012
reg_CTRL	chr8	8265440	C	T	69	45,00%	het	31			-	PRAGMIN	rs2976972			1,00E-012
reg_CTRL	chr8	8265655	C	A	55	58,00%	het	32			-	PRAGMIN	rs2979166			1,00E-012
reg_CTRL	chr8	8265814	C	T	27	26,00%	het	7			-	PRAGMIN	rs10481460			2,02E-006
reg_CTRL	chr8	8267228	C	G	49	61,00%	het	30			-	PRAGMIN	rs2979134			1,00E-012
reg_CTRL	chr8	8268322	G	A	41	59,00%	het	24			-	PRAGMIN	rs2921009			1,00E-012
reg_CTRL	chr8	8269101	A	G	74	55,00%	het	41			-	PRAGMIN	rs13280051			1,00E-012
reg_CTRL	chr8	8269203	A	C	71	45,00%	het	32			-	PRAGMIN	rs4840946			1,00E-012
reg_CTRL	chr8	8269553	T	C	65	57,00%	het	37			-	PRAGMIN	rs4840947			1,00E-012
reg_CTRL	chr8	8269943	C	T	43	37,00%	het	16			-	PRAGMIN	rs4840948			1,00E-012
reg_CTRL	chr8	8270279	G	A	18	50,00%	het	9			-	PRAGMIN	rs12546045			7,33E-011
reg_CTRL	chr8	8270377	C	A	19	53,00%	het	10			-	PRAGMIN	rs12547958			4,83E-012
reg_CTRL	chr8	8270536	G	C	26	46,00%	het	12			-	PRAGMIN	rs4840950			1,00E-012
reg_CTRL	chr8	8270542	G	A	26	50,00%	het	13			-	PRAGMIN	rs4840951			1,00E-012
reg_CTRL	chr8	8271487	G	C	17	59,00%	het	10	A	A	-1	PRAGMIN	rs4840952			1,00E-012
reg_CTRL	chr8	8271602	G	C	15	60,00%	het	9	S	C	-1	PRAGMIN	rs4840953			1,11E-011
reg_CTRL	chr8	8271628	C	T	16	56,00%	het	9	P	P	-1	PRAGMIN	rs41314930	rs4840954		2,54E-011
reg_CTRL	chr8	8271629	G	A	16	56,00%	het	9	P	L	-1	PRAGMIN	rs4840954	rs4840955		2,54E-011
reg_CTRL	chr8	8274087	C	T	34	100,00%	homvar	34			-	PRAGMIN	rs2976958			1,00E-012
reg_CTRL	chr8	8274496	T	G	75	41,00%	het	31			-	PRAGMIN	rs3932318			1,00E-012
reg_CTRL	chr8	8679890	G	A	36	100,00%	homvar	36			-	MFHAS1	rs4841038			1,00E-012
reg_CTRL	chr8	8680797	G	A	17	94,00%	homvar	16			-	MFHAS1	rs10903311			1,00E-012
reg_CTRL	chr8	8680992	C	G	57	100,00%	homvar	57			-	MFHAS1	rs2271340			1,00E-012
reg_CTRL	chr8	8681135	T	A	71	97,00%	homvar	69			-	MFHAS1	rs2271341			1,00E-012
reg_CTRL	chr8	8681348	C	T	66	100,00%	homvar	66			-	MFHAS1	rs2271342			1,00E-012
reg_CTRL	chr8	8681684	G	C	34	97,00%	homvar	33			-	MFHAS1	rs12677543			1,00E-012
reg_CTRL	chr8	8681732	G	A	35	100,00%	homvar	35			-	MFHAS1	rs12677550			1,00E-012
reg_CTRL	chr8	8682101	T	C	30	93,00%	homvar	28			-	MFHAS1	rs7015606			1,00E-012
reg_CTRL	chr8	8683135	G	C	66	98,00%	homvar	65			-	MFHAS1	rs2409088			1,00E-012
reg_CTRL	chr8	8683656	T	C	21	100,00%	homvar	21			-	MFHAS1	rs12682352			1,00E-012
reg_CTRL	chr8	8684040	C	T	43	65,00%	het	28			-	MFHAS1				1,00E-012
reg_CTRL	chr8	8686807	T	C	30	100,00%	homvar	30			-	MFHAS1	rs2409089			1,00E-012
reg_CTRL	chr8	8687291	C	T	34	91,00%	homvar	31			-	MFHAS1	rs11249891			1,00E-012
reg_CTRL	chr8	8688829	T	C	33	100,00%	homvar	33			-	MFHAS1	rs6601732			1,00E-012
reg_CTRL	chr8	8690299	G	A	57	96,00%	homvar	55			-	MFHAS1	rs7832968			1,00E-012
reg_CTRL	chr8	8691268	G	A	47	98,00%	homvar	46			-	MFHAS1	rs2409090			1,00E-012

reg_CTRL	chr8	8691467	G	A	6	83,00%	homvar	5	-	MFHAS1	rs2409091		3,79E-008
reg_CTRL	chr8	8691521	C	G	6	100,00%	homvar	6	-	MFHAS1	rs7460947		1,48E-010
reg_CTRL	chr8	8691937	T	C	13	100,00%	homvar	13	-	MFHAS1	rs4841040		1,00E-012
reg_CTRL	chr8	8691951	C	G	15	100,00%	homvar	15	-	MFHAS1	rs4841041		1,00E-012
reg_CTRL	chr8	8692433	C	T	35	94,00%	homvar	33	-	MFHAS1	rs3748144		1,00E-012
reg_CTRL	chr8	8694647	A	G	27	11,00%	ambig	3	-	MFHAS1			2,36E-002
reg_CTRL	chr8	8694650	C	T	28	11,00%	ambig	3	-	MFHAS1	rs11249892		2,60E-002
reg_CTRL	chr8	8695314	T	A	66	44,00%	het	29	-	MFHAS1			1,00E-012
reg_CTRL	chr8	8695950	A	G	19	95,00%	homvar	18	-	MFHAS1	rs2048419		1,00E-012
reg_CTRL	chr8	8696081	C	G	38	11,00%	ambig	4	-	MFHAS1			1,11E-002
reg_CTRL	chr8	8696864	C	T	76	50,00%	het	38	-	MFHAS1		rs62495537	1,00E-012
reg_CTRL	chr8	8697085	G	T	74	100,00%	homvar	74	-	MFHAS1	rs13282015		1,00E-012
reg_CTRL	chr8	8697948	C	A	27	100,00%	homvar	27	-	MFHAS1	rs6994038		1,00E-012
reg_CTRL	chr8	8698944	T	C	55	100,00%	homvar	55	-	MFHAS1	rs12547493		1,00E-012
reg_CTRL	chr8	8699091	C	G	79	100,00%	homvar	79	-	MFHAS1	rs12544992		1,00E-012
reg_CTRL	chr8	8700625	C	T	15	73,00%	het	11	-	MFHAS1	rs28399241		1,00E-012
reg_CTRL	chr8	8701507	C	T	33	97,00%	homvar	32	-	MFHAS1	rs9329167		1,00E-012
reg_CTRL	chr8	8701755	T	C	19	16,00%	ambig	3	-	MFHAS1			8,95E-003
reg_CTRL	chr8	8701932	C	A	27	11,00%	ambig	3	-	MFHAS1			2,36E-002
reg_CTRL	chr8	8702026	C	T	26	23,00%	ambig	6	-	MFHAS1			2,29E-005
reg_CTRL	chr8	8702028	T	A	27	22,00%	ambig	6	-	MFHAS1			2,89E-005
reg_CTRL	chr8	8702032	G	A	26	96,00%	homvar	25	-	MFHAS1	rs4841042		1,00E-012
reg_CTRL	chr8	8702089	G	A	35	74,00%	het	26	-	MFHAS1	rs4841043		1,00E-012
reg_CTRL	chr8	8702110	G	A	35	26,00%	het	9	-	MFHAS1			7,40E-008
reg_CTRL	chr8	8702129	C	A	35	26,00%	het	9	-	MFHAS1			7,40E-008
reg_CTRL	chr8	8702133	C	T	34	26,00%	het	9	-	MFHAS1		rs6982682	5,61E-008
reg_CTRL	chr8	8702350	G	A	47	96,00%	homvar	45	-	MFHAS1	rs4841044		1,00E-012
reg_CTRL	chr8	8702557	G	A	51	94,00%	homvar	48	-	MFHAS1	rs11783966		1,00E-012
reg_CTRL	chr8	8703143	T	A	9	67,00%	het	6	-	MFHAS1	rs9644775		1,17E-008
reg_CTRL	chr8	8703212	T	C	12	67,00%	het	8	-	MFHAS1	rs9644776		4,67E-011
reg_CTRL	chr8	8704326	C	T	30	100,00%	homvar	30	-	MFHAS1	rs6988939		1,00E-012
reg_CTRL	chr8	8704329	G	T	30	97,00%	homvar	29	-	MFHAS1	rs2175161		1,00E-012
reg_CTRL	chr8	8704854	C	T	56	100,00%	homvar	56	-	MFHAS1	rs6993494		1,00E-012
reg_CTRL	chr8	8705169	T	C	48	10,00%	ambig	5	-	MFHAS1			4,85E-003
reg_CTRL	chr8	8705807	A	G	37	97,00%	homvar	36	-	MFHAS1	rs7006418		1,00E-012
reg_CTRL	chr8	8705896	A	G	38	100,00%	homvar	38	-	MFHAS1	rs7006589		1,00E-012
reg_CTRL	chr8	8706327	A	C	36	100,00%	homvar	36	-	MFHAS1	rs1473029		1,00E-012
reg_CTRL	chr8	8707492	G	C	31	100,00%	homvar	31	-	MFHAS1	rs4840362		1,00E-012
reg_CTRL	chr8	8707587	T	A	30	100,00%	homvar	30	-	MFHAS1	rs7823757		1,00E-012
reg_CTRL	chr8	8708009	A	G	16	100,00%	homvar	16	-	MFHAS1		rs60315134	1,00E-012
reg_CTRL	chr8	8708146	C	A	22	100,00%	homvar	22	-	MFHAS1		rs59046059	1,00E-012
reg_CTRL	chr8	8709372	C	T	46	100,00%	homvar	46	-	MFHAS1	rs11784052		1,00E-012
reg_CTRL	chr8	8709629	T	A	33	88,00%	homvar	29	-	MFHAS1	rs10088933		1,00E-012
reg_CTRL	chr8	8709839	G	C	16	94,00%	homvar	15	-	MFHAS1	rs11777085		1,00E-012
reg_CTRL	chr8	8709989	A	G	12	100,00%	homvar	12	-	MFHAS1	rs4841045		1,00E-012
reg_CTRL	chr8	8710211	C	T	6	100,00%	homvar	6	-	MFHAS1	rs4841046		1,48E-010
reg_CTRL	chr8	8710362	A	C	9	89,00%	homvar	8	-	MFHAS1	rs4841047		1,00E-012
reg_CTRL	chr8	8710730	T	C	52	98,00%	homvar	51	-	MFHAS1	rs13265731		1,00E-012
reg_CTRL	chr8	8711011	A	C	72	99,00%	homvar	71	-	MFHAS1	rs13259216		1,00E-012
reg_CTRL	chr8	8711146	T	C	76	99,00%	homvar	75	-	MFHAS1	rs35431455		1,00E-012
reg_CTRL	chr8	8712586	A	G	17	100,00%	homvar	17	-	MFHAS1	rs13260419		1,00E-012
reg_CTRL	chr8	8712735	A	T	21	100,00%	homvar	21	-	MFHAS1	rs35039922		1,00E-012
reg_CTRL	chr8	8713900	T	C	59	53,00%	het	31	-	MFHAS1	rs950721		1,00E-012

reg_CTRL	chr8	8715940	G	A	37	97,00%	homvar	36	-	MFHAS1	rs882462		1,00E-012
reg_CTRL	chr8	8716307	G	C	50	100,00%	homvar	50	-	MFHAS1	rs3827809		1,00E-012
reg_CTRL	chr8	8716586	A	G	65	98,00%	homvar	64	-	MFHAS1	rs11775523		1,00E-012
reg_CTRL	chr8	8716735	G	C	51	98,00%	homvar	50	-	MFHAS1	rs28755903		1,00E-012
reg_CTRL	chr8	8716866	G	C	30	100,00%	homvar	30	-	MFHAS1	rs1039913		1,00E-012
reg_CTRL	chr8	8716959	C	T	18	100,00%	homvar	18	-	MFHAS1	rs1039914		1,00E-012
reg_CTRL	chr8	8717024	T	C	13	100,00%	homvar	13	-	MFHAS1	rs1039915		1,00E-012
reg_CTRL	chr8	8717493	G	A	3	100,00%	homvar	3	-	MFHAS1	rs11779585		1,22E-005
reg_CTRL	chr8	8717887	G	A	35	100,00%	homvar	35	-	MFHAS1		rs57312668	1,00E-012
reg_CTRL	chr8	8718276	C	G	48	100,00%	homvar	48	-	MFHAS1	rs4840364		1,00E-012
reg_CTRL	chr8	8718775	A	C	41	98,00%	homvar	40	-	MFHAS1	rs4841049		1,00E-012
reg_CTRL	chr8	8719000	T	C	44	100,00%	homvar	44	-	MFHAS1	rs4841050		1,00E-012
reg_CTRL	chr8	8719166	T	C	49	100,00%	homvar	49	-	MFHAS1	rs1876836		1,00E-012
reg_CTRL	chr8	8719602	A	T	36	100,00%	homvar	36	-	MFHAS1	rs2409092		1,00E-012
reg_CTRL	chr8	8720288	T	C	11	91,00%	homvar	10	-	MFHAS1	rs12545499		1,00E-012
reg_CTRL	chr8	8720310	G	C	12	100,00%	homvar	12	-	MFHAS1	rs2409094		1,00E-012
reg_CTRL	chr8	8720681	A	C	36	97,00%	homvar	35	-	MFHAS1	rs907179		1,00E-012
reg_CTRL	chr8	8722363	G	A	87	100,00%	homvar	87	-	MFHAS1	rs1533059		1,00E-012
reg_CTRL	chr8	8722600	A	G	50	98,00%	homvar	49	-	MFHAS1	rs1533058		1,00E-012
reg_CTRL	chr8	8723056	T	C	59	100,00%	homvar	59	-	MFHAS1	rs4841051		1,00E-012
reg_CTRL	chr8	8723264	A	G	65	100,00%	homvar	65	-	MFHAS1	rs1039916		1,00E-012
reg_CTRL	chr8	8724090	T	A	37	95,00%	homvar	35	-	MFHAS1	rs2409095		1,00E-012
reg_CTRL	chr8	8724255	C	A	24	46,00%	het	11	-	MFHAS1	rs3789850		3,07E-012
reg_CTRL	chr8	8724430	A	G	27	11,00%	ambig	3	-	MFHAS1			2,36E-002
reg_CTRL	chr8	8724464	G	C	28	61,00%	het	17	-	MFHAS1	rs3789849		1,00E-012
reg_CTRL	chr8	8724497	A	G	32	22,00%	ambig	7	-	MFHAS1	rs3789848		6,91E-006
reg_CTRL	chr8	8724735	A	G	32	59,00%	het	19	-	MFHAS1	rs7013471		1,00E-012
reg_CTRL	chr8	8725871	T	C	23	48,00%	het	11	-	MFHAS1	rs13275083		1,66E-012
reg_CTRL	chr8	8726186	G	A	48	42,00%	het	20	-	MFHAS1			1,00E-012
reg_CTRL	chr8	8726960	G	A	38	26,00%	het	10	-	MFHAS1			1,09E-008
reg_CTRL	chr8	8726963	C	T	32	66,00%	het	21	-	MFHAS1			1,00E-012
reg_CTRL	chr8	8726976	G	A	28	89,00%	homvar	25	-	MFHAS1			1,00E-012
reg_CTRL	chr8	8726978	G	A	28	100,00%	homvar	28	-	MFHAS1			1,00E-012
reg_CTRL	chr8	8726980	G	A	30	93,00%	homvar	28	-	MFHAS1			1,00E-012
reg_CTRL	chr8	8726982	G	A	29	86,00%	homvar	25	-	MFHAS1	rs28821557		1,00E-012
reg_CTRL	chr8	8727030	A	G	14	50,00%	het	7	-	MFHAS1			1,01E-008
reg_CTRL	chr8	8727280	T	C	32	62,00%	het	20	-	MFHAS1		rs57784779	1,00E-012
reg_CTRL	chr8	8727376	G	C	53	70,00%	het	37	-	MFHAS1	rs4840366		1,00E-012
reg_CTRL	chr8	8727494	A	C	45	62,00%	het	28	-	MFHAS1		rs73504221	1,00E-012
reg_CTRL	chr8	8727500	G	A	45	60,00%	het	27	-	MFHAS1		rs73504222	1,00E-012
reg_CTRL	chr8	8727803	G	T	35	100,00%	homvar	35	-	MFHAS1	rs11995244		1,00E-012
reg_CTRL	chr8	8727836	T	C	38	100,00%	homvar	38	-	MFHAS1	rs13259619		1,00E-012
reg_CTRL	chr8	8728197	C	T	45	49,00%	het	22	-	MFHAS1	rs13259070		1,00E-012
reg_CTRL	chr8	8728321	G	A	57	47,00%	het	27	-	MFHAS1	rs4840367		1,00E-012
reg_CTRL	chr8	8728410	G	A	53	62,00%	het	33	-	MFHAS1	rs4840368		1,00E-012
reg_CTRL	chr8	8728607	T	A	60	47,00%	het	28	-	MFHAS1	rs4840369		1,00E-012
reg_CTRL	chr8	8728688	T	G	52	46,00%	het	24	-	MFHAS1	rs4840370		1,00E-012
reg_CTRL	chr8	8728794	C	G	37	100,00%	homvar	37	-	MFHAS1	rs9329169		1,00E-012
reg_CTRL	chr8	8729032	T	A	26	73,00%	het	19	-	MFHAS1	rs13270070		1,00E-012
reg_CTRL	chr8	8729476	G	C	47	100,00%	homvar	47	-	MFHAS1	rs1510932		1,00E-012
reg_CTRL	chr8	8729887	T	C	40	32,00%	het	13	-	MFHAS1	rs2409096		8,21E-012
reg_CTRL	chr8	8729950	C	G	41	100,00%	homvar	41	-	MFHAS1	rs1510933		1,00E-012
reg_CTRL	chr8	8730145	T	C	48	46,00%	het	22	-	MFHAS1	rs7820478		1,00E-012

reg_CTRL	chr8	8731603	C	G	41	98,00%	homvar	40	-	MFHAS1	rs13254903		1,00E-012
reg_CTRL	chr8	8732984	C	T	60	100,00%	homvar	60	-	MFHAS1	rs6601265		1,00E-012
reg_CTRL	chr8	8733538	C	G	28	11,00%	ambig	3	-	MFHAS1			2,60E-002
reg_CTRL	chr8	8733859	T	G	58	48,00%	het	28	-	MFHAS1	rs1510934		1,00E-012
reg_CTRL	chr8	8735238	A	G	25	12,00%	ambig	3	-	MFHAS1			1,92E-002
reg_CTRL	chr8	8735502	C	T	41	41,00%	het	17	-	MFHAS1	rs4841054		1,00E-012
reg_CTRL	chr8	8736213	A	G	23	13,00%	ambig	3	-	MFHAS1			1,53E-002
reg_CTRL	chr8	8736247	G	T	24	17,00%	ambig	4	-	MFHAS1			2,06E-003
reg_CTRL	chr8	8736571	A	G	38	97,00%	homvar	37	-	MFHAS1	rs4841055		1,00E-012
reg_CTRL	chr8	8737167	T	A	28	57,00%	het	16	-	MFHAS1	rs7820146		1,00E-012
reg_CTRL	chr8	8737171	C	T	28	61,00%	het	17	-	MFHAS1	rs7833171		1,00E-012
reg_CTRL	chr8	8737668	G	A	58	36,00%	het	21	-	MFHAS1		rs73190070	1,00E-012
reg_CTRL	chr8	8738012	G	C	21	62,00%	het	13	-	MFHAS1	rs7017006		1,00E-012
reg_CTRL	chr8	8738114	C	G	17	41,00%	het	7	-	MFHAS1		rs12265954	5,41E-008
reg_CTRL	chr8	8738151	T	C	14	21,00%	ambig	3	-	MFHAS1			3,66E-003
reg_CTRL	chr8	8738166	C	G	12	33,00%	het	4	-	MFHAS1		rs11995330	1,19E-004
reg_CTRL	chr8	8738261	C	T	7	100,00%	homvar	7	-	MFHAS1	rs11249893		3,40E-012
reg_CTRL	chr8	8738469	C	T	23	48,00%	het	11	-	MFHAS1		rs73190071	1,66E-012
reg_CTRL	chr8	8739698	G	C	72	47,00%	het	34	-	MFHAS1			1,00E-012
reg_CTRL	chr8	8740017	G	C	67	55,00%	het	37	-	MFHAS1	rs7820738		1,00E-012
reg_CTRL	chr8	8740237	A	G	55	56,00%	het	31	-	MFHAS1	rs907180		1,00E-012
reg_CTRL	chr8	8740285	T	C	53	58,00%	het	31	-	MFHAS1	rs907181		1,00E-012
reg_CTRL	chr8	8740955	T	C	26	12,00%	ambig	3	-	MFHAS1			2,13E-002
reg_CTRL	chr8	8741091	C	T	8	100,00%	homvar	8	-	MFHAS1	rs6996376		1,00E-012
reg_CTRL	chr8	8741740	G	C	72	100,00%	homvar	72	-	MFHAS1	rs4481596		1,00E-012
reg_CTRL	chr8	8742375	G	T	39	62,00%	het	24	-	MFHAS1	rs11249894		1,00E-012
reg_CTRL	chr8	8742590	C	T	82	48,00%	het	39	-	MFHAS1	rs11249895		1,00E-012
reg_CTRL	chr8	8743742	A	C	37	41,00%	het	15	-	MFHAS1	rs408459		1,00E-012
reg_CTRL	chr8	8743837	A	T	24	67,00%	het	16	-	MFHAS1			1,00E-012
reg_CTRL	chr8	8744430	G	A	34	32,00%	het	11	-	MFHAS1			1,76E-010
reg_CTRL	chr8	8744607	C	G	51	43,00%	het	22	-	MFHAS1	rs1877119		1,00E-012
reg_CTRL	chr8	8745380	C	A	62	40,00%	het	25	-	MFHAS1	rs387706		1,00E-012
reg_CTRL	chr8	8746124	A	C	81	100,00%	homvar	81	-	MFHAS1	rs440788		1,00E-012
reg_CTRL	chr8	8746384	C	G	89	47,00%	het	42	-	MFHAS1	rs3925830		1,00E-012
reg_CTRL	chr8	8746560	T	G	68	47,00%	het	32	-	MFHAS1	rs451082		1,00E-012
reg_CTRL	chr8	8747166	G	C	21	67,00%	het	14	-	MFHAS1	rs1964719		1,00E-012
reg_CTRL	chr8	8747381	C	T	26	46,00%	het	12	-	MFHAS1	rs3958877		1,00E-012
reg_CTRL	chr8	8748211	G	A	27	100,00%	homvar	27	-	MFHAS1	rs437895		1,00E-012
reg_CTRL	chr8	8749016	T	A	14	29,00%	het	4	-	MFHAS1	rs1251003		2,33E-004
reg_CTRL	chr8	8749357	A	C	7	100,00%	homvar	7	-	MFHAS1	rs435953		3,40E-012
reg_CTRL	chr8	8750151	G	A	18	61,00%	het	11	-	MFHAS1			1,00E-012
reg_CTRL	chr8	8750305	A	G	32	56,00%	het	18	-	MFHAS1	rs4348501		1,00E-012
reg_CTRL	chr8	8750416	G	A	32	97,00%	homvar	31	-	MFHAS1	rs231188		1,00E-012
reg_CTRL	chr8	8750448	C	T	25	96,00%	homvar	24	-	MFHAS1	rs4523255		1,00E-012
reg_CTRL	chr8	8751363	T	C	47	45,00%	het	21	-	MFHAS1		rs56073940	1,00E-012
reg_CTRL	chr8	8752492	T	C	18	17,00%	ambig	3	-	MFHAS1			7,66E-003
reg_CTRL	chr8	8752514	C	T	18	17,00%	ambig	3	-	MFHAS1			7,66E-003
reg_CTRL	chr8	8752581	A	G	26	15,00%	ambig	4	-	MFHAS1			2,79E-003
reg_CTRL	chr8	8753576	G	T	54	30,00%	het	16	-	MFHAS1	rs332029		1,00E-012
reg_CTRL	chr8	8755553	C	T	72	50,00%	het	36	-	MFHAS1			1,00E-012
reg_CTRL	chr8	8756260	G	A	93	57,00%	het	53	-	MFHAS1	rs1039917		1,00E-012
reg_CTRL	chr8	8756622	A	C	68	53,00%	het	36	-	MFHAS1			1,00E-012
reg_CTRL	chr8	8756923	G	A	54	46,00%	het	25	-	MFHAS1	rs35900578		1,00E-012

reg_CTRL	chr8	8758831	C	T	31	48,00%	het	15			-	MFHAS1			1,00E-012
reg_CTRL	chr8	8758883	G	A	30	100,00%	homvar	30			-	MFHAS1	rs4382480		1,00E-012
reg_CTRL	chr8	8759937	G	A	32	47,00%	het	15			-	MFHAS1		rs56367294	1,00E-012
reg_CTRL	chr8	8760085	C	T	17	100,00%	homvar	17			-	MFHAS1	rs332037		1,00E-012
reg_CTRL	chr8	8760419	G	A	54	43,00%	het	23			-	MFHAS1			1,00E-012
reg_CTRL	chr8	8761061	C	G	37	100,00%	homvar	37			-	MFHAS1	rs332039		1,00E-012
reg_CTRL	chr8	8761328	G	T	21	43,00%	het	9			-	MFHAS1	rs3789845		4,19E-010
reg_CTRL	chr8	8761521	A	G	50	50,00%	het	25			-	MFHAS1	rs3789844		1,00E-012
reg_CTRL	chr8	8761667	C	T	65	100,00%	homvar	65			-	MFHAS1	rs3789843		1,00E-012
reg_CTRL	chr8	8761686	C	T	64	100,00%	homvar	64			-	MFHAS1	rs3827806		1,00E-012
reg_CTRL	chr8	8761706	A	C	62	53,00%	het	33			-	MFHAS1			1,00E-012
reg_CTRL	chr8	8761825	C	T	57	98,00%	homvar	56			-	MFHAS1		rs60707155	1,00E-012
reg_CTRL	chr8	8762536	G	T	19	89,00%	homvar	17			-	MFHAS1	rs7017599		1,00E-012
reg_CTRL	chr8	8762639	G	A	15	93,00%	homvar	14			-	MFHAS1	rs1821007		1,00E-012
reg_CTRL	chr8	8762729	G	A	24	92,00%	homvar	22			-	MFHAS1	rs1821008		1,00E-012
reg_CTRL	chr8	8764214	G	T	92	95,00%	homvar	87			-	MFHAS1	rs1567398		1,00E-012
reg_CTRL	chr8	8766603	A	C	52	46,00%	het	24			-	MFHAS1	rs13274028		1,00E-012
reg_CTRL	chr8	8766641	C	G	60	52,00%	het	31			-	MFHAS1		rs73524014	1,00E-012
reg_CTRL	chr8	8766707	G	A	68	50,00%	het	34			-	MFHAS1		rs73524015	1,00E-012
reg_CTRL	chr8	8767171	G	C	57	100,00%	homvar	57			-	MFHAS1	rs907183		1,00E-012
reg_CTRL	chr8	8767310	G	A	49	51,00%	het	25			-	MFHAS1	rs10098667		1,00E-012
reg_CTRL	chr8	8767552	A	G	55	42,00%	het	23			-	MFHAS1	rs11991673		1,00E-012
reg_CTRL	chr8	8767898	G	A	42	100,00%	homvar	42			-	MFHAS1	rs332040		1,00E-012
reg_CTRL	chr8	8768326	G	C	56	95,00%	homvar	53			-	MFHAS1	rs4841058		1,00E-012
reg_CTRL	chr8	8768646	G	A	56	41,00%	het	23			-	MFHAS1		rs61591712	1,00E-012
reg_CTRL	chr8	8769293	C	T	47	81,00%	homvar	38			-	MFHAS1	rs9644694		1,00E-012
reg_CTRL	chr8	8770589	T	C	32	41,00%	het	13			-	MFHAS1	rs4840372		1,00E-012
reg_CTRL	chr8	8770670	T	C	38	11,00%	ambig	4			-	MFHAS1			1,11E-002
reg_CTRL	chr8	8770694	C	G	38	11,00%	ambig	4			-	MFHAS1			1,11E-002
reg_CTRL	chr8	8770735	G	C	42	100,00%	homvar	42			-	MFHAS1	rs2009455		1,00E-012
reg_CTRL	chr8	8771021	G	C	85	49,00%	het	42			-	MFHAS1	rs9644671		1,00E-012
reg_CTRL	chr8	8772507	A	C	59	100,00%	homvar	59			-	MFHAS1	rs10046783		1,00E-012
reg_CTRL	chr8	8772623	A	C	70	97,00%	homvar	68			-	MFHAS1	rs10046784		1,00E-012
reg_CTRL	chr8	8773796	A	G	57	100,00%	homvar	57			-	MFHAS1	rs12679021		1,00E-012
reg_CTRL	chr8	8774098	G	A	24	29,00%	het	7			-	MFHAS1	rs12681432		8,35E-007
reg_CTRL	chr8	8774113	A	T	23	74,00%	het	17			-	MFHAS1	rs7824578		1,00E-012
reg_CTRL	chr8	8774196	G	A	27	33,00%	het	9			-	MFHAS1	rs13261380		5,80E-009
reg_CTRL	chr8	8774295	G	T	37	43,00%	het	16			-	MFHAS1	rs34599909		1,00E-012
reg_CTRL	chr8	8774325	G	T	43	53,00%	het	23			-	MFHAS1		rs60965369	1,00E-012
reg_CTRL	chr8	8774535	T	C	70	56,00%	het	39			-	MFHAS1		rs73192206	1,00E-012
reg_CTRL	chr8	8774874	T	G	68	96,00%	homvar	65			-	MFHAS1	rs409997		1,00E-012
reg_CTRL	chr8	8775015	T	C	60	98,00%	homvar	59			-	MFHAS1	rs410487		1,00E-012
reg_CTRL	chr8	8775059	C	T	58	57,00%	het	33			-	MFHAS1	rs1533100		1,00E-012
reg_CTRL	chr8	8775749	C	T	30	10,00%	ambig	3			-	MFHAS1			3,11E-002
reg_CTRL	chr8	8775750	A	G	30	10,00%	ambig	3			-	MFHAS1			3,11E-002
reg_CTRL	chr8	8775793	T	C	30	10,00%	ambig	3			-	MFHAS1			3,11E-002
reg_CTRL	chr8	8776018	C	G	38	42,00%	het	16			-	MFHAS1		rs72626639	1,00E-012
reg_CTRL	chr8	8776268	A	G	55	49,00%	het	27			-	MFHAS1			1,00E-012
reg_CTRL	chr8	8776551	T	C	27	11,00%	ambig	3			-	MFHAS1			2,36E-002
reg_CTRL	chr8	8780758	G	T	22	95,00%	homvar	21			-	MFHAS1	rs435393		1,00E-012
reg_CTRL	chr8	8781650	G	A	37	59,00%	het	22			-	MFHAS1	rs7818276		1,00E-012
reg_CTRL	chr8	8784947	T	C	82	98,00%	homvar	80			-	MFHAS1	rs399123		1,00E-012
reg_CTRL	chr8	8785304	A	G	46	98,00%	homvar	45	L	P	-1	MFHAS1	rs429433		1,00E-012

reg_CTRL	chr8	11738136	A	C	30	100,00%	homvar	30			-	CTSB	rs1142957	rs1142956		1,00E-012
reg_CTRL	chr8	11738154	G	C	35	100,00%	homvar	35			-	CTSB	rs1736077			1,00E-012
reg_CTRL	chr8	11738505	A	C	37	100,00%	homvar	37			-	CTSB	rs8005			1,00E-012
reg_CTRL	chr8	11738739	G	A	10	100,00%	homvar	10			-	CTSB	rs11786618			1,00E-012
reg_CTRL	chr8	11739251	A	C	61	97,00%	homvar	59			-	CTSB	rs1736078			1,00E-012
reg_CTRL	chr8	11739415	A	T	50	100,00%	homvar	50			-	CTSB	rs9009			1,00E-012
reg_CTRL	chr8	11739613	G	A	26	100,00%	homvar	26			-	CTSB	rs6730			1,00E-012
reg_CTRL	chr8	11739951	T	C	33	100,00%	homvar	33			-	CTSB	rs8898			1,00E-012
reg_CTRL	chr8	11743086	C	G	29	100,00%	homvar	29			-	CTSB	rs2294140			1,00E-012
reg_CTRL	chr8	11744382	T	C	20	100,00%	homvar	20			-	CTSB	rs4840586			1,00E-012
reg_CTRL	chr8	11744417	C	A	21	95,00%	homvar	20			-	CTSB	rs2645423			1,00E-012
reg_CTRL	chr8	11746049	T	C	24	12,00%	ambig	3			-	CTSB				1,72E-002
reg_CTRL	chr8	11746749	A	T	17	18,00%	ambig	3			-	CTSB				6,50E-003
reg_CTRL	chr8	11749604	A	G	18	17,00%	ambig	3			-	CTSB				7,66E-003
reg_CTRL	chr8	11752051	T	C	27	11,00%	ambig	3			-	CTSB				2,36E-002
reg_CTRL	chr8	11752052	G	C	27	11,00%	ambig	3			-	CTSB				2,36E-002
reg_CTRL	chr8	11752204	A	G	13	23,00%	ambig	3			-	CTSB				2,93E-003
reg_CTRL	chr8	11755710	T	C	32	47,00%	het	15			-	CTSB				1,00E-012
reg_CTRL	chr8	11758292	A	G	39	13,00%	ambig	5			-	CTSB				1,93E-003
reg_CTRL	chr8	11760540	A	G	32	100,00%	homvar	32			-	CTSB	rs1293307			1,00E-012
reg_CTRL	chr8	11761184	G	A	20	35,00%	het	7			-	CTSB	rs1293309			2,03E-007
reg_CTRL	chr8	12624420	G	A	70	100,00%	homvar	70			-	LONRF1	rs7005881			1,00E-012
reg_CTRL	chr8	12625320	T	C	58	52,00%	het	30			-	LONRF1			rs73202627	1,00E-012
reg_CTRL	chr8	12626051	T	C	64	100,00%	homvar	64			-	LONRF1	rs4831767			1,00E-012
reg_CTRL	chr8	12626226	C	T	66	53,00%	het	35			-	LONRF1	rs4831768			1,00E-012
reg_CTRL	chr8	12626235	A	G	66	98,00%	homvar	65			-	LONRF1	rs4831769			1,00E-012
reg_CTRL	chr8	12627074	T	A	57	100,00%	homvar	57			-	LONRF1	rs10429335			1,00E-012
reg_CTRL	chr8	12627236	A	G	49	59,00%	het	29			-	LONRF1			rs55752837	1,00E-012
reg_CTRL	chr8	12627915	C	T	75	49,00%	het	37			-	LONRF1	rs11782145			1,00E-012
reg_CTRL	chr8	12628025	T	C	78	47,00%	het	37			-	LONRF1	rs10100866			1,00E-012
reg_CTRL	chr8	12628174	G	C	57	44,00%	het	25			-	LONRF1			rs73202629	1,00E-012
reg_CTRL	chr8	12628721	A	G	34	35,00%	het	12			-	LONRF1	rs9632851			1,59E-011
reg_CTRL	chr8	12629393	C	A	30	50,00%	het	15			-	LONRF1			rs73202633	1,00E-012
reg_CTRL	chr8	12629532	A	T	52	96,00%	homvar	50			-	LONRF1	rs6530953			1,00E-012
reg_CTRL	chr8	12629807	C	G	93	56,00%	het	52			-	LONRF1	rs7010337			1,00E-012
reg_CTRL	chr8	12630196	A	T	87	45,00%	het	39			-	LONRF1	rs4831770			1,00E-012
reg_CTRL	chr8	12630632	A	G	54	50,00%	het	27			-	LONRF1	rs3802268			1,00E-012
reg_CTRL	chr8	12630635	A	C	54	50,00%	het	27			-	LONRF1			rs73202635	1,00E-012
reg_CTRL	chr8	12631165	G	C	49	14,00%	ambig	7	T	S	-3	LONRF1				1,25E-004
reg_CTRL	chr8	12631166	T	A	49	14,00%	ambig	7	T	S	-3	LONRF1				1,25E-004
reg_CTRL	chr8	12631700	T	C	44	48,00%	het	21			-	LONRF1	rs17761564			1,00E-012
reg_CTRL	chr8	12631939	T	C	37	59,00%	het	22			-	LONRF1			rs56114121	1,00E-012
reg_CTRL	chr8	12632233	T	A	66	61,00%	het	40			-	LONRF1	rs17761606			1,00E-012
reg_CTRL	chr8	12632654	A	C	48	98,00%	homvar	47			-	LONRF1	rs6995647			1,00E-012
reg_CTRL	chr8	12633550	C	A	51	100,00%	homvar	51			-	LONRF1	rs3802269			1,00E-012
reg_CTRL	chr8	12634490	G	C	62	35,00%	het	22			-	LONRF1	rs4272378			1,00E-012
reg_CTRL	chr8	12634940	G	A	27	100,00%	homvar	27			-	LONRF1	rs7463601			1,00E-012
reg_CTRL	chr8	12634992	A	G	31	97,00%	homvar	30			-	LONRF1	rs7461006			1,00E-012
reg_CTRL	chr8	12635185	A	G	34	100,00%	homvar	34			-	LONRF1	rs6530956			1,00E-012
reg_CTRL	chr8	12635399	G	A	37	95,00%	homvar	35			-	LONRF1	rs6530958			1,00E-012
reg_CTRL	chr8	12635958	A	C	38	95,00%	homvar	36			-	LONRF1	rs4258004			1,00E-012
reg_CTRL	chr8	12636319	C	T	66	100,00%	homvar	66			-	LONRF1	rs6530959			1,00E-012
reg_CTRL	chr8	12637327	C	A	57	100,00%	homvar	57			-	LONRF1	rs13251315			1,00E-012

reg_CTRL	chr8	12637416	A	G	54	94,00%	homvar	51			-	LONRF1	rs13272425		1,00E-012
reg_CTRL	chr8	12637991	T	C	45	100,00%	homvar	45			-	LONRF1	rs9325786		1,00E-012
reg_CTRL	chr8	12640631	C	T	56	100,00%	homvar	56			-	LONRF1	rs7014187		1,00E-012
reg_CTRL	chr8	12641320	A	G	38	42,00%	het	16			-	LONRF1	rs4831777		1,00E-012
reg_CTRL	chr8	12641415	C	G	46	61,00%	het	28			-	LONRF1		rs73202639	1,00E-012
reg_CTRL	chr8	12641627	G	A	71	56,00%	het	40			-	LONRF1			1,00E-012
reg_CTRL	chr8	12642348	C	A	56	66,00%	het	37			-	LONRF1	rs6530962		1,00E-012
reg_CTRL	chr8	12642503	A	C	55	51,00%	het	28			-	LONRF1	rs4625037		1,00E-012
reg_CTRL	chr8	12642559	T	C	57	51,00%	het	29			-	LONRF1	rs11775169		1,00E-012
reg_CTRL	chr8	12642979	C	T	38	100,00%	homvar	38			-	LONRF1	rs4831354		1,00E-012
reg_CTRL	chr8	12643126	C	T	31	48,00%	het	15			-	LONRF1	rs7819033		1,00E-012
reg_CTRL	chr8	12643453	A	C	48	98,00%	homvar	47			-	LONRF1	rs4831780		1,00E-012
reg_CTRL	chr8	12643642	A	C	47	94,00%	homvar	44			-	LONRF1	rs10098734		1,00E-012
reg_CTRL	chr8	12643654	G	A	48	96,00%	homvar	46			-	LONRF1	rs10110145		1,00E-012
reg_CTRL	chr8	12643838	T	C	48	52,00%	het	25			-	LONRF1	rs7014429		1,00E-012
reg_CTRL	chr8	12644977	T	A	35	46,00%	het	16			-	LONRF1		rs73202645	1,00E-012
reg_CTRL	chr8	12644993	C	T	37	62,00%	het	23			-	LONRF1	rs7837242		1,00E-012
reg_CTRL	chr8	12645091	T	A	48	42,00%	het	20	I	L	-2	LONRF1	rs1139354		1,00E-012
reg_CTRL	chr8	12645405	C	T	52	98,00%	homvar	51			-	LONRF1	rs6530964		1,00E-012
reg_CTRL	chr8	12645526	A	C	44	52,00%	het	23			-	LONRF1	rs6530965		1,00E-012
reg_CTRL	chr8	12646136	C	G	39	97,00%	homvar	38			-	LONRF1	rs7842201		1,00E-012
reg_CTRL	chr8	12646342	T	C	38	39,00%	het	15			-	LONRF1	rs7819248		1,00E-012
reg_CTRL	chr8	12646448	A	G	47	34,00%	het	16			-	LONRF1	rs4831360		1,00E-012
reg_CTRL	chr8	12646912	G	C	44	73,00%	het	32			-	LONRF1	rs4831784		1,00E-012
reg_CTRL	chr8	12647447	T	C	77	55,00%	het	42			-	LONRF1	rs17829381		1,00E-012
reg_CTRL	chr8	12647560	C	G	82	45,00%	het	37			-	LONRF1	rs6985289		1,00E-012
reg_CTRL	chr8	12647843	T	C	85	48,00%	het	41			-	LONRF1	rs17829441		1,00E-012
reg_CTRL	chr8	12647982	A	T	93	49,00%	het	46			-	LONRF1	rs6530966		1,00E-012
reg_CTRL	chr8	12648329	G	C	102	98,00%	homvar	100			-	LONRF1	rs7838660		1,00E-012
reg_CTRL	chr8	12648422	T	C	89	49,00%	het	44			-	LONRF1	rs7832448		1,00E-012
reg_CTRL	chr8	12648780	C	G	72	54,00%	het	39			-	LONRF1	rs9325792		1,00E-012
reg_CTRL	chr8	12648809	T	C	70	47,00%	het	33			-	LONRF1		rs73202647	1,00E-012
reg_CTRL	chr8	12648863	A	G	61	52,00%	het	32			-	LONRF1	rs9325793		1,00E-012
reg_CTRL	chr8	12649062	G	T	56	100,00%	homvar	56			-	LONRF1	rs7014516		1,00E-012
reg_CTRL	chr8	12649595	G	A	19	100,00%	homvar	19			-	LONRF1	rs10441667		1,00E-012
reg_CTRL	chr8	12650052	T	C	29	45,00%	het	13			-	LONRF1	rs17767600		1,00E-012
reg_CTRL	chr8	12650145	C	T	31	45,00%	het	14			-	LONRF1	rs7462166		1,00E-012
reg_CTRL	chr8	12650206	G	C	35	49,00%	het	17			-	LONRF1	rs6530967		1,00E-012
reg_CTRL	chr8	12651062	T	C	45	51,00%	het	23			-	LONRF1	rs4436128		1,00E-012
reg_CTRL	chr8	12651131	T	C	51	45,00%	het	23			-	LONRF1	rs4437649		1,00E-012
reg_CTRL	chr8	12651557	C	G	46	39,00%	het	18			-	LONRF1	rs10503427		1,00E-012
reg_CTRL	chr8	12651605	G	A	34	59,00%	het	20			-	LONRF1	rs6991754		1,00E-012
reg_CTRL	chr8	12651680	C	T	46	100,00%	homvar	46			-	LONRF1	rs7007056		1,00E-012
reg_CTRL	chr8	12652000	C	T	56	98,00%	homvar	55			-	LONRF1	rs7007550		1,00E-012
reg_CTRL	chr8	12652284	C	G	63	98,00%	homvar	62			-	LONRF1	rs6530968		1,00E-012
reg_CTRL	chr8	12652948	G	A	62	50,00%	het	31			-	LONRF1		rs73202653	1,00E-012
reg_CTRL	chr8	12653488	C	G	27	96,00%	homvar	26			-	LONRF1	rs4831795		1,00E-012
reg_CTRL	chr8	12654972	A	G	85	99,00%	homvar	84			-	LONRF1	rs6530969		1,00E-012
reg_CTRL	chr8	12655147	T	C	88	52,00%	het	46			-	LONRF1	rs6530970		1,00E-012
