# Supplementary material for: Comprehensive assessment of sequence variation within the copy number variable defensin cluster on 8p23 by target enriched in-depth 454 sequencing
Source: BMC Genomics. 2011 May 18;12:243. doi: 10.1186/1471-2164-12-243 (PMC3118217; doi:10.1186/1471-2164-12-243)
Supplement: Additional file 12 — SNVs identified from NA12716 and NA12760. SNVs identified from NA12716 and NA12760 (CTRL, DEFA, DEFB), after filtering of HCDiffs following workflow in Figure 1 [file 1471-2164-12-243-S12.PDF]

additional file 12: SNVs identified from NA12716 and NA12760

DNA	region	hg18	pos	ref	var	dep_tot	VAF	dep_var	aa_ref	aa_var	strand	gene	SNP_ID	SNP_alias	SNPblat_db130	P
NA12716	reg_CTRL	chr17	31285944	A	G	75	49,00%	37	F	S	-3	LYZL6	rs9754			1,00E-012
NA12716	reg_CTRL	chr17	31287000	G	C	8	37,00%	3			-	LYZL6	rs11654713			6,25E-004
NA12716	reg_CTRL	chr19	59865309	A	G	36	47,00%	17					rs1749311			1,00E-012
NA12716	reg_CTRL	chr19	59865333	A	G	39	100,00%	39					rs430978			1,00E-012
NA12716	reg_CTRL	chr19	59865618	C	G	68	100,00%	68					rs413679			1,00E-012
NA12716	reg_CTRL	chr19	59865626	G	C	67	97,00%	65					rs12609815			1,00E-012
NA12716	reg_CTRL	chr19	59866025	C	T	41	44,00%	18			+	LILRB4	rs1654668			1,00E-012
NA12716	reg_CTRL	chr19	59866821	G	T	3	100,00%	3	R	S	2	LILRB4	rs11574570			1,22E-005
NA12716	reg_CTRL	chr19	59867552	C	T	23	30,00%	7	F	F	2	LILRB4	rs3745871			6,03E-007
NA12716	reg_CTRL	chr19	59868074	A	G	24	100,00%	24	D	G	2	LILRB4	rs731170			1,00E-012
NA12716	reg_CTRL	chr19	59868347	C	T	18	33,00%	6			+	LILRB4	rs1925241			2,17E-006
NA12716	reg_CTRL	chr19	59868514	A	C	11	45,00%	5			+	LILRB4	rs1631746			2,65E-006
NA12716	reg_CTRL	chr19	59868598	C	T	11	36,00%	4			+	LILRB4	rs1749316			8,11E-005
NA12716	reg_CTRL	chr19	59870069	T	A	19	21,00%	4			+	LILRB4			rs393665	8,22E-004
NA12716	reg_CTRL	chr19	59870148	T	C	21	38,00%	8			+	LILRB4				1,22E-008
NA12716	reg_CTRL	chr19	59870157	G	T	21	24,00%	5			+	LILRB4				9,62E-005
NA12716	reg_CTRL	chr19	59870171	C	T	20	30,00%	6			+	LILRB4				4,35E-006
NA12716	reg_CTRL	chr19	59870190	A	G	20	50,00%	10			+	LILRB4				9,66E-012
NA12716	reg_CTRL	chr19	59870239	A	G	19	47,00%	9			+	LILRB4				1,37E-010
NA12716	reg_CTRL	chr19	59870279	C	G	21	24,00%	5			+	LILRB4				9,62E-005
NA12716	reg_CTRL	chr19	59870298	A	G	22	45,00%	10			+	LILRB4				3,38E-011
NA12716	reg_CTRL	chr19	59870327	T	A	24	29,00%	7			+	LILRB4			rs3865478	8,35E-007
NA12716	reg_CTRL	chr19	59870329	T	C	24	29,00%	7			+	LILRB4				8,35E-007
NA12716	reg_CTRL	chr19	59870332	T	C	24	33,00%	8			+	LILRB4				4,14E-008
NA12716	reg_CTRL	chr19	59870428	G	A	28	39,00%	11			+	LILRB4			rs443874	2,64E-011
NA12716	reg_CTRL	chr19	59870494	A	C	26	50,00%	13			+	LILRB4			rs3893859	1,00E-012
NA12716	reg_CTRL	chr19	59870501	T	G	25	56,00%	14			+	LILRB4				1,00E-012
NA12716	reg_CTRL	chr19	59870545	A	G	31	55,00%	17			+	LILRB4	rs11574582			1,00E-012
NA12716	reg_CTRL	chr19	59870587	G	A	29	48,00%	14			+	LILRB4				1,00E-012
NA12716	reg_CTRL	chr19	59870591	A	T	28	46,00%	13			+	LILRB4			rs62133430	1,00E-012
NA12716	reg_CTRL	chr19	59870609	A	G	31	45,00%	14			+	LILRB4			rs73933940	1,00E-012
NA12716	reg_CTRL	chr19	59870612	G	A	30	17,00%	5			+	LILRB4				5,67E-004
NA12716	reg_CTRL	chr19	59870677	A	G	34	21,00%	7			+	LILRB4			rs685381	1,06E-005
NA12716	reg_CTRL	chr19	59870794	G	A	36	61,00%	22			+	LILRB4	rs11574587			1,00E-012
NA12716	reg_CTRL	chr19	59870797	A	G	35	29,00%	10			+	LILRB4			rs41308138	4,50E-009
NA12716	reg_CTRL	chr19	59870823	T	C	35	43,00%	15			+	LILRB4			rs71365476	1,00E-012
NA12716	reg_CTRL	chr19	59870834	G	A	35	66,00%	23			+	LILRB4				1,00E-012
NA12716	reg_CTRL	chr19	59870860	A	G	41	34,00%	14			+	LILRB4				1,00E-012
NA12716	reg_CTRL	chr19	59870864	T	C	40	45,00%	18			+	LILRB4				1,00E-012
NA12716	reg_CTRL	chr19	59870940	A	G	37	19,00%	7	K	E	1	LILRB4	rs2764337	rs11574589		1,91E-005
NA12716	reg_CTRL	chr19	59870957	A	T	35	34,00%	12	R	S	1	LILRB4			rs61738946	2,42E-011
NA12716	reg_CTRL	chr19	59870988	C	T	37	30,00%	11	P	S	1	LILRB4			rs61743356	4,70E-010
NA12716	reg_CTRL	chr19	59871028	C	T	36	25,00%	9	A	V	1	LILRB4	rs11574591			9,66E-008
NA12716	reg_CTRL	chr19	59871029	A	G	36	39,00%	14	A	A	1	LILRB4	rs11574591			1,00E-012
NA12716	reg_CTRL	chr19	59871041	A	G	36	47,00%	17	R	R	1	LILRB4	rs11574592			1,00E-012
NA12716	reg_CTRL	chr19	59871057	G	A	36	17,00%	6			+	LILRB4				1,59E-004
NA12716	reg_CTRL	chr19	59871112	C	A	28	25,00%	7			+	LILRB4			rs57804895	2,63E-006
NA12716	reg_CTRL	chr19	59871189	T	G	35	17,00%	6	F	L	1	LILRB4			rs634222	1,35E-004
NA12716	reg_CTRL	chr19	60076609	C	A	17	41,00%	7					rs11084374			5,41E-008
NA12716	reg_CTRL	chr19	60077218	G	A	49	55,00%	27					rs17772004			1,00E-012

NA12716	reg_CTRL	chr19	60077247	T	C	45	49,00%	22					rs12462181			1,00E-012
NA12716	reg_CTRL	chr19	60077416	T	C	71	46,00%	33			+	FCAR	rs3816051			1,00E-012
NA12716	reg_CTRL	chr19	60078436	C	G	28	39,00%	11			+	FCAR	rs11084376			2,64E-011
NA12716	reg_CTRL	chr19	60078702	A	G	90	42,00%	38			+	FCAR	rs11084377			1,00E-012
NA12716	reg_CTRL	chr19	60078732	A	G	88	42,00%	37			+	FCAR	rs8112766			1,00E-012
NA12716	reg_CTRL	chr19	60081277	A	G	10	70,00%	7			+	FCAR	rs4806604			3,85E-010
NA12716	reg_CTRL	chr19	60081476	T	C	16	50,00%	8			+	FCAR	rs4806605			8,62E-010
NA12716	reg_CTRL	chr19	60082413	A	G	9	89,00%	8			+	FCAR	rs7257926			1,00E-012
NA12716	reg_CTRL	chr19	60083518	A	T	18	50,00%	9			+	FCAR	rs6509905			7,33E-011
NA12716	reg_CTRL	chr19	60084458	A	G	21	95,00%	20			+	FCAR	rs7259090			1,00E-012
NA12716	reg_CTRL	chr19	60084567	C	T	16	100,00%	16			+	FCAR	rs7259347			1,00E-012
NA12716	reg_CTRL	chr19	60084881	T	C	14	100,00%	14			+	FCAR	rs7248382	rs7247547		1,00E-012
NA12716	reg_CTRL	chr19	60084977	A	G	12	100,00%	12			+	FCAR	rs12975418			1,00E-012
NA12716	reg_CTRL	chr19	60085090	A	G	11	45,00%	5			+	FCAR	rs34764559			2,65E-006
NA12716	reg_CTRL	chr19	60085431	A	G	41	100,00%	41			+	FCAR	rs4239591			1,00E-012
NA12716	reg_CTRL	chr19	60085626	A	G	33	24,00%	8			+	FCAR	rs4806606			6,50E-007
NA12716	reg_CTRL	chr19	60085864	G	A	20	30,00%	6			+	FCAR	rs10412499			4,35E-006
NA12716	reg_CTRL	chr19	60086546	G	A	26	50,00%	13			+	FCAR	rs11671686			1,00E-012
NA12716	reg_CTRL	chr19	60087161	C	G	33	55,00%	18			+	FCAR	rs28756208			1,00E-012
NA12716	reg_CTRL	chr19	60087357	C	T	26	31,00%	8			+	FCAR	rs4806607			8,44E-008
NA12716	reg_CTRL	chr19	60087394	A	G	26	27,00%	7			+	FCAR	rs4806608			1,52E-006
NA12716	reg_CTRL	chr19	60087684	G	A	20	50,00%	10			+	FCAR	rs7260414			9,66E-012
NA12716	reg_CTRL	chr19	60088124	G	A	8	100,00%	8			+	FCAR	rs10401687			1,00E-012
NA12716	reg_CTRL	chr19	60088425	G	A	38	100,00%	38			+	FCAR	rs10402324			1,00E-012
NA12716	reg_CTRL	chr19	60088712	A	G	71	99,00%	70	R	R	2	FCAR	rs1865096			1,00E-012
NA12716	reg_CTRL	chr19	60088725	G	A	73	53,00%	39	D	N	2	FCAR	rs11666735			1,00E-012
NA12716	reg_CTRL	chr19	60089029	A	G	37	100,00%	37			+	FCAR	rs1865097			1,00E-012
NA12716	reg_CTRL	chr19	60089192	C	T	13	100,00%	13			+	FCAR	rs11666846			1,00E-012
NA12716	reg_CTRL	chr19	60089829	T	C	14	71,00%	10			+	FCAR	rs12974530			1,00E-012
NA12716	reg_CTRL	chr19	60089913	A	G	17	100,00%	17			+	FCAR	rs12972637			1,00E-012
NA12716	reg_CTRL	chr19	60090047	T	C	27	100,00%	27			+	FCAR	rs12975083			1,00E-012
NA12716	reg_CTRL	chr19	60090359	T	C	9	100,00%	9			+	FCAR			rs59223694	1,00E-012
NA12716	reg_CTRL	chr19	60090376	C	T	9	100,00%	9			+	FCAR			rs59401716	1,00E-012
NA12716	reg_CTRL	chr19	60090674	T	C	3	100,00%	3			+	FCAR	rs7258735			1,22E-005
NA12716	reg_CTRL	chr19	60092982	A	G	72	58,00%	42	S	G	2	FCAR	rs16986050			1,00E-012
NA12716	reg_CTRL	chr19	60093536	C	T	20	35,00%	7			+	FCAR	rs10413148			2,03E-007
NA12716	reg_CTRL	chr19	60093544	T	G	20	35,00%	7			+	FCAR	rs10414707			2,03E-007
NA12716	reg_CTRL	chr20	29527992	A	G	40	47,00%	19	H	R	1	REM1	rs1006459			1,00E-012
NA12716	reg_CTRL	chr20	29530017	G	T	45	53,00%	24			+	REM1	rs717064			1,00E-012
NA12716	reg_CTRL	chr20	29530975	C	T	86	42,00%	36			+	REM1	rs752841			1,00E-012
NA12716	reg_CTRL	chr20	29530984	A	G	86	100,00%	86			+	REM1	rs215911			1,00E-012
NA12716	reg_CTRL	chr20	29532051	T	C	49	47,00%	23			+	REM1	rs8120526			1,00E-012
NA12716	reg_CTRL	chr20	29535505	C	T	62	52,00%	32			+	REM1	rs2233834			1,00E-012
NA12716	reg_CTRL	chr20	29591769	T	G	75	97,00%	73			+	HM13	rs1555285			1,00E-012
NA12716	reg_CTRL	chr20	29596516	T	A	58	71,00%	41			+	HM13				1,00E-012
NA12716	reg_CTRL	chr20	29597668	T	C	8	37,00%	3			+	HM13				6,25E-004
NA12716	reg_CTRL	chr20	29620213	C	T	85	15,00%	13			+	HM13				7,92E-008
NA12716	reg_CTRL	chr8	6346527	C	T	9	78,00%	7			+	MCPH1	rs2916714			1,44E-010
NA12716	reg_CTRL	chr8	6349118	A	G	29	90,00%	26			+	MCPH1	rs7841673			1,00E-012
NA12716	reg_CTRL	chr8	6354204	C	T	85	98,00%	83			+	MCPH1				1,00E-012
NA12716	reg_CTRL	chr8	6358884	C	T	77	95,00%	73			+	MCPH1	rs1982386			1,00E-012
NA12716	reg_CTRL	chr8	6360207	G	A	70	90,00%	63			+	MCPH1	rs7816398			1,00E-012
NA12716	reg_CTRL	chr8	6360907	T	C	72	100,00%	72			+	MCPH1	rs2009235			1,00E-012

NA12716	reg_CTRL	chr8	6365605	G	A	65	98,00%	64			+	MCPH1	rs1960240	1,00E-012
NA12716	reg_CTRL	chr8	6367073	G	A	30	97,00%	29			+	MCPH1	rs2515446	1,00E-012
NA12716	reg_CTRL	chr8	6368638	C	G	11	100,00%	11			+	MCPH1	rs1989105	1,00E-012
NA12716	reg_CTRL	chr8	6370028	C	T	67	97,00%	65			+	MCPH1	rs2959817	1,00E-012
NA12716	reg_CTRL	chr8	6370280	A	G	23	91,00%	21			+	MCPH1	rs3020225	1,00E-012
NA12716	reg_CTRL	chr8	6370714	A	T	61	89,00%	54			+	MCPH1	rs2922889	1,00E-012
NA12716	reg_CTRL	chr8	6371007	A	G	64	92,00%	59			+	MCPH1	rs2515462	1,00E-012
NA12716	reg_CTRL	chr8	6372214	C	T	57	96,00%	55			+	MCPH1	rs2959814	1,00E-012
NA12716	reg_CTRL	chr8	6373087	T	A	13	100,00%	13			+	MCPH1	rs2442610	1,00E-012
NA12716	reg_CTRL	chr8	6373247	T	G	42	98,00%	41			+	MCPH1	rs2515464	1,00E-012
NA12716	reg_CTRL	chr8	6373270	G	C	44	100,00%	44			+	MCPH1	rs2515465	1,00E-012
NA12716	reg_CTRL	chr8	6373612	C	T	97	97,00%	94			+	MCPH1	rs6990020	1,00E-012
NA12716	reg_CTRL	chr8	6373694	A	G	90	97,00%	87			+	MCPH1	rs2515466	1,00E-012
NA12716	reg_CTRL	chr8	6374188	C	G	89	98,00%	87			+	MCPH1	rs7825407	1,00E-012
NA12716	reg_CTRL	chr8	6376528	G	T	33	100,00%	33			+	MCPH1	rs2515479	1,00E-012
NA12716	reg_CTRL	chr8	6377297	C	G	69	99,00%	68	A	A	-1	ANGPT2	rs6559167	1,00E-012
NA12716	reg_CTRL	chr8	6382432	G	A	43	58,00%	25			+	MCPH1		1,00E-012
NA12716	reg_CTRL	chr8	6382971	T	A	14	43,00%	6			+	MCPH1	rs2959808	3,79E-007
NA12716	reg_CTRL	chr8	6383317	G	A	75	45,00%	34			+	MCPH1	rs11989215	1,00E-012
NA12716	reg_CTRL	chr8	6383428	G	A	78	50,00%	39			+	MCPH1	rs11989242	1,00E-012
NA12716	reg_CTRL	chr8	6383749	C	T	67	49,00%	33			+	MCPH1	rs17623313	1,00E-012
NA12716	reg_CTRL	chr8	6384278	G	A	53	41,00%	22			+	MCPH1	rs1375668	1,00E-012
NA12716	reg_CTRL	chr8	6384313	G	A	49	39,00%	19			+	MCPH1	rs1989321	1,00E-012
NA12716	reg_CTRL	chr8	6384394	G	T	49	43,00%	21			+	MCPH1	rs2897911	1,00E-012
NA12716	reg_CTRL	chr8	6384406	G	C	48	37,00%	18			+	MCPH1	rs1823375	1,00E-012
NA12716	reg_CTRL	chr8	6384513	C	A	24	25,00%	6			+	MCPH1	rs1823376	1,39E-005
NA12716	reg_CTRL	chr8	6384555	T	G	20	25,00%	5			+	MCPH1	rs2408341	7,47E-005
NA12716	reg_CTRL	chr8	6384627	C	T	19	53,00%	10			+	MCPH1	rs4263789	4,83E-012
NA12716	reg_CTRL	chr8	6384716	A	T	15	40,00%	6			+	MCPH1	rs4376511	6,20E-007
NA12716	reg_CTRL	chr8	6384838	A	T	44	41,00%	18			+	MCPH1	rs4455855	1,00E-012
NA12716	reg_CTRL	chr8	6384861	C	T	48	44,00%	21			+	MCPH1	rs4991608	1,00E-012
NA12716	reg_CTRL	chr8	6385735	A	G	76	54,00%	41			+	MCPH1	rs2922873	1,00E-012
NA12716	reg_CTRL	chr8	6385973	T	C	46	52,00%	24			+	MCPH1	rs2922871	1,00E-012
NA12716	reg_CTRL	chr8	6386346	G	T	43	49,00%	21			+	MCPH1	rs2959820	1,00E-012
NA12716	reg_CTRL	chr8	6387110	A	G	17	29,00%	5			+	MCPH1		3,16E-005
NA12716	reg_CTRL	chr8	6387274	T	G	28	43,00%	12			+	MCPH1	rs35742902	1,00E-012
NA12716	reg_CTRL	chr8	6387413	G	A	46	46,00%	21			+	MCPH1	rs1988762	1,00E-012
NA12716	reg_CTRL	chr8	6387489	C	A	48	48,00%	23			+	MCPH1	rs2515486	1,00E-012
NA12716	reg_CTRL	chr8	6387856	C	A	52	63,00%	33			+	MCPH1	rs2515487	1,00E-012
NA12716	reg_CTRL	chr8	6389049	C	T	87	99,00%	86			+	MCPH1	rs2442597	1,00E-012
NA12716	reg_CTRL	chr8	6389775	G	A	115	50,00%	58			+	MCPH1	rs13250248	1,00E-012
NA12716	reg_CTRL	chr8	6390400	A	G	61	100,00%	61			+	MCPH1	rs2442596	1,00E-012
NA12716	reg_CTRL	chr8	6391248	T	C	67	100,00%	67			+	MCPH1	rs2044744	1,00E-012
NA12716	reg_CTRL	chr8	6391663	G	C	81	99,00%	80			+	MCPH1	rs2515489	1,00E-012
NA12716	reg_CTRL	chr8	6391722	A	G	81	99,00%	80			+	MCPH1	rs2442595	1,00E-012
NA12716	reg_CTRL	chr8	6392028	A	T	67	100,00%	67			+	MCPH1	rs2442594	1,00E-012
NA12716	reg_CTRL	chr8	6392149	T	C	57	98,00%	56			+	MCPH1	rs2442593	1,00E-012
NA12716	reg_CTRL	chr8	6392271	T	C	56	82,00%	46			+	MCPH1	rs2515490	1,00E-012
NA12716	reg_CTRL	chr8	6393016	C	T	71	100,00%	71			+	MCPH1	rs2442592	1,00E-012
NA12716	reg_CTRL	chr8	6393121	G	A	83	100,00%	83			+	MCPH1	rs2515492	1,00E-012
NA12716	reg_CTRL	chr8	6393980	C	A	120	98,00%	118			+	MCPH1	rs2515493	1,00E-012
NA12716	reg_CTRL	chr8	6394533	T	C	53	100,00%	53			+	MCPH1	rs2515494	1,00E-012
NA12716	reg_CTRL	chr8	6395624	A	T	58	84,00%	49			+	MCPH1	rs2442591	1,00E-012

NA12716	reg_CTRL	chr8	6397787	C	T	61	52,00%	32			+	MCPH1	rs2515497			1,00E-012
NA12716	reg_CTRL	chr8	6399828	G	C	49	100,00%	49			+	MCPH1	rs2515500			1,00E-012
NA12716	reg_CTRL	chr8	6399852	G	C	50	70,00%	35			+	MCPH1	rs13262156			1,00E-012
NA12716	reg_CTRL	chr8	6400140	G	A	67	100,00%	67			+	MCPH1	rs2515502			1,00E-012
NA12716	reg_CTRL	chr8	6400377	A	G	24	96,00%	23			+	MCPH1	rs2922883			1,00E-012
NA12716	reg_CTRL	chr8	6400446	T	C	25	96,00%	24			+	MCPH1	rs2515503			1,00E-012
NA12716	reg_CTRL	chr8	6401094	A	T	50	12,00%	6			+	MCPH1				9,88E-004
NA12716	reg_CTRL	chr8	6401634	C	T	40	90,00%	36			+	MCPH1	rs2515504			1,00E-012
NA12716	reg_CTRL	chr8	6404255	A	G	38	100,00%	38			+	MCPH1	rs2515505			1,00E-012
NA12716	reg_CTRL	chr8	6406668	T	G	52	92,00%	48			+	MCPH1	rs2515506			1,00E-012
NA12716	reg_CTRL	chr8	6407942	A	G	86	99,00%	85			+	MCPH1	rs3739391			1,00E-012
NA12716	reg_CTRL	chr8	6657680	A	G	110	49,00%	54			-	XKR5	rs12544341			1,00E-012
NA12716	reg_CTRL	chr8	6657763	G	C	114	97,00%	111			-	XKR5	rs2615788			1,00E-012
NA12716	reg_CTRL	chr8	6659973	G	T	52	62,00%	32			-	XKR5				1,00E-012
NA12716	reg_CTRL	chr8	6660158	G	C	35	46,00%	16			-	XKR5				1,00E-012
NA12716	reg_CTRL	chr8	6660662	A	G	33	58,00%	19			-	XKR5			rs58112583	1,00E-012
NA12716	reg_CTRL	chr8	6660984	G	C	51	45,00%	23			-	XKR5	rs2738077			1,00E-012
NA12716	reg_CTRL	chr8	6661868	A	G	48	54,00%	26			-	XKR5	rs9773025			1,00E-012
NA12716	reg_CTRL	chr8	6669959	T	C	62	47,00%	29			-	XKR5				1,00E-012
NA12716	reg_CTRL	chr8	6671547	C	T	97	54,00%	52			-	XKR5	rs2741083			1,00E-012
NA12716	reg_CTRL	chr8	6672148	A	G	101	100,00%	101			-	XKR5	rs2980958			1,00E-012
NA12716	reg_CTRL	chr8	6672229	T	G	106	100,00%	106			-	XKR5	rs2980957			1,00E-012
NA12716	reg_CTRL	chr8	6672308	C	G	113	100,00%	113			-	XKR5	rs2980956			1,00E-012
NA12716	reg_CTRL	chr8	6673095	C	A	55	49,00%	27			-	XKR5	rs2702924			1,00E-012
NA12716	reg_CTRL	chr8	6673198	G	C	86	45,00%	39			-	XKR5	rs2741086			1,00E-012
NA12716	reg_CTRL	chr8	6674201	C	T	55	35,00%	19			-	XKR5	rs2741087			1,00E-012
NA12716	reg_CTRL	chr8	6675647	T	C	39	49,00%	19			-	XKR5	rs2741089			1,00E-012
NA12716	reg_CTRL	chr8	6675775	G	C	43	51,00%	22			-	XKR5	rs2978903			1,00E-012
NA12716	reg_CTRL	chr8	6676149	C	T	62	53,00%	33			-	XKR5				1,00E-012
NA12716	reg_CTRL	chr8	6676673	T	C	69	96,00%	66			-	XKR5	rs2741091			1,00E-012
NA12716	reg_CTRL	chr8	6676904	A	G	31	16,00%	5			-	XKR5				6,64E-004
NA12716	reg_CTRL	chr8	6677583	G	C	90	99,00%	89			-	XKR5	rs2978902			1,00E-012
NA12716	reg_CTRL	chr8	6677625	G	C	83	99,00%	82			-	XKR5	rs2978901			1,00E-012
NA12716	reg_CTRL	chr8	6677686	T	C	80	99,00%	79	M	V	-2	XKR5	rs2741098			1,00E-012
NA12716	reg_CTRL	chr8	6678708	T	C	57	93,00%	53			-	XKR5	rs2978900			1,00E-012
NA12716	reg_CTRL	chr8	6679008	C	G	64	98,00%	63			-	XKR5	rs2978899			1,00E-012
NA12716	reg_CTRL	chr8	6679788	T	C	3	100,00%	3			-	XKR5	rs2978898			1,22E-005
NA12716	reg_CTRL	chr8	6680072	G	C	3	100,00%	3			-	XKR5	rs2978897			1,22E-005
NA12716	reg_DEFA	chr8	6714615	G	A	48	48,00%	23					rs2702829			1,00E-012
NA12716	reg_DEFA	chr8	6715398	A	G	24	54,00%	13					rs2741123			1,00E-012
NA12716	reg_DEFA	chr8	6715928	C	G	94	40,00%	38			-	DEFB1	rs2702885			1,00E-012
NA12716	reg_DEFA	chr8	6715984	G	A	86	44,00%	38			-	DEFB1	rs2741124			1,00E-012
NA12716	reg_DEFA	chr8	6716532	A	G	76	51,00%	39			-	DEFB1	rs2741125			1,00E-012
NA12716	reg_DEFA	chr8	6716724	G	A	75	40,00%	30			-	DEFB1				1,00E-012
NA12716	reg_DEFA	chr8	6717327	T	C	68	54,00%	37			-	DEFB1	rs2978874	rs5743482		1,00E-012
NA12716	reg_DEFA	chr8	6717328	G	A	69	55,00%	38			-	DEFB1	rs5743482			1,00E-012
NA12716	reg_DEFA	chr8	6717829	A	G	95	99,00%	94			-	DEFB1	rs2980928			1,00E-012
NA12716	reg_DEFA	chr8	6717856	A	G	89	100,00%	89			-	DEFB1	rs2980927			1,00E-012
NA12716	reg_DEFA	chr8	6717956	T	C	102	43,00%	44			-	DEFB1	rs2977779			1,00E-012
NA12716	reg_DEFA	chr8	6717999	G	A	108	99,00%	107			-	DEFB1	rs2977778			1,00E-012
NA12716	reg_DEFA	chr8	6718012	T	G	107	100,00%	107			-	DEFB1	rs2980926			1,00E-012
NA12716	reg_DEFA	chr8	6718140	C	G	113	100,00%	113			-	DEFB1	rs2978872			1,00E-012
NA12716	reg_DEFA	chr8	6718172	G	A	103	100,00%	103			-	DEFB1	rs2977777			1,00E-012

NA12716	reg_DEFA	chr8	6718680	C	T	64	98,00%	63	-	DEFB1	rs2978870			1,00E-012
NA12716	reg_DEFA	chr8	6718690	C	T	63	38,00%	24	-	DEFB1	rs2741126			1,00E-012
NA12716	reg_DEFA	chr8	6718808	T	A	54	98,00%	53	-	DEFB1	rs2977776			1,00E-012
NA12716	reg_DEFA	chr8	6718836	A	T	55	98,00%	54	-	DEFB1	rs2951854			1,00E-012
NA12716	reg_DEFA	chr8	6718897	G	A	51	53,00%	27	-	DEFB1	rs2741127			1,00E-012
NA12716	reg_DEFA	chr8	6718913	A	C	50	98,00%	49	-	DEFB1	rs2927345			1,00E-012
NA12716	reg_DEFA	chr8	6718939	C	G	54	46,00%	25	-	DEFB1	rs5743467			1,00E-012
NA12716	reg_DEFA	chr8	6719079	G	A	48	75,00%	36	-	DEFB1	rs11269568	rs9694202		1,00E-012
NA12716	reg_DEFA	chr8	6719161	C	A	49	100,00%	49	-	DEFB1	rs2978869			1,00E-012
NA12716	reg_DEFA	chr8	6719186	T	C	53	100,00%	53	-	DEFB1	rs2977774			1,00E-012
NA12716	reg_DEFA	chr8	6719218	C	A	53	100,00%	53	-	DEFB1	rs2978868			1,00E-012
NA12716	reg_DEFA	chr8	6719403	A	G	48	100,00%	48	-	DEFB1	rs2980924			1,00E-012
NA12716	reg_DEFA	chr8	6719467	G	C	48	98,00%	47	-	DEFB1	rs5743465	rs34929240		1,00E-012
NA12716	reg_DEFA	chr8	6719468	T	A	48	96,00%	46	-	DEFB1	rs34929240	rs5743464		1,00E-012
NA12716	reg_DEFA	chr8	6719584	G	T	42	100,00%	42	-	DEFB1	rs5743463			1,00E-012
NA12716	reg_DEFA	chr8	6719596	A	G	37	100,00%	37	-	DEFB1	rs5743462			1,00E-012
NA12716	reg_DEFA	chr8	6719656	G	A	30	100,00%	30	-	DEFB1	rs2978867			1,00E-012
NA12716	reg_DEFA	chr8	6719740	C	T	24	96,00%	23	-	DEFB1	rs2978866			1,00E-012
NA12716	reg_DEFA	chr8	6720060	G	A	36	100,00%	36	-	DEFB1	rs2977773			1,00E-012
NA12716	reg_DEFA	chr8	6720108	T	C	43	98,00%	42	-	DEFB1	rs2951855			1,00E-012
NA12716	reg_DEFA	chr8	6720467	A	G	82	94,00%	77	-	DEFB1	rs2980923			1,00E-012
NA12716	reg_DEFA	chr8	6720606	G	A	80	97,00%	78	-	DEFB1	rs2977772			1,00E-012
NA12716	reg_DEFA	chr8	6720679	G	A	91	49,00%	45	-	DEFB1	rs2741129			1,00E-012
NA12716	reg_DEFA	chr8	6721130	T	C	45	93,00%	42	-	DEFB1	rs2978864	rs5743441		1,00E-012
NA12716	reg_DEFA	chr8	6721183	C	T	36	97,00%	35	-	DEFB1	rs5743440			1,00E-012
NA12716	reg_DEFA	chr8	6721210	C	A	32	94,00%	30	-	DEFB1	rs5743439			1,00E-012
NA12716	reg_DEFA	chr8	6721278	T	C	17	100,00%	17	-	DEFB1	rs5743437			1,00E-012
NA12716	reg_DEFA	chr8	6721281	A	G	17	47,00%	8	-	DEFB1	rs2741130			1,60E-009
NA12716	reg_DEFA	chr8	6721663	A	G	22	41,00%	9	-	DEFB1	rs2980922			6,96E-010
NA12716	reg_DEFA	chr8	6721755	A	C	33	82,00%	27	-	DEFB1	rs2980921			1,00E-012
NA12716	reg_DEFA	chr8	6722097	T	C	93	98,00%	91	-	DEFB1	rs2702945			1,00E-012
NA12716	reg_DEFA	chr8	6722258	G	C	48	100,00%	48	-	DEFB1	rs2293960			1,00E-012
NA12716	reg_DEFA	chr8	6722484	C	T	38	100,00%	38	-	DEFB1	rs2293959			1,00E-012
NA12716	reg_DEFA	chr8	6722710	T	A	65	98,00%	64	-	DEFB1	rs2293958			1,00E-012
NA12716	reg_DEFA	chr8	6722809	C	T	72	49,00%	35	-	DEFB1	rs11362			1,00E-012
NA12716	reg_DEFA	chr8	6722833	C	G	74	100,00%	74	-	DEFB1	rs1800972			1,00E-012
NA12716	reg_DEFA	chr8	6722841	C	T	71	49,00%	35	-	DEFB1	rs1799946			1,00E-012
NA12716	reg_DEFA	chr8	6723179	A	T	65	48,00%	31			rs2738182			1,00E-012
NA12716	reg_DEFA	chr8	6723399	C	T	52	44,00%	23			rs2741132			1,00E-012
NA12716	reg_DEFA	chr8	6723423	G	C	46	46,00%	21			rs2702876	rs7838626		1,00E-012
NA12716	reg_DEFA	chr8	6723465	G	T	42	48,00%	20			rs2741133			1,00E-012
NA12716	reg_DEFA	chr8	6723477	G	C	43	98,00%	42			rs2702877			1,00E-012
NA12716	reg_DEFA	chr8	6723483	T	C	46	100,00%	46			rs2977829			1,00E-012
NA12716	reg_DEFA	chr8	6723520	G	A	49	43,00%	21			rs2741134			1,00E-012
NA12716	reg_DEFA	chr8	6723531	C	T	50	44,00%	22			rs2978863			1,00E-012
NA12716	reg_DEFA	chr8	6723664	G	A	57	46,00%	26			rs2741135			1,00E-012
NA12716	reg_DEFA	chr8	6723906	A	T	43	42,00%	18			rs2738181			1,00E-012
NA12716	reg_DEFA	chr8	6723908	C	T	42	40,00%	17			rs2738180			1,00E-012
NA12716	reg_DEFA	chr8	6723927	T	G	39	36,00%	14			rs2738179			1,00E-012
NA12716	reg_DEFA	chr8	6723930	A	C	39	36,00%	14			rs2738178			1,00E-012
NA12716	reg_DEFA	chr8	6724030	C	A	25	48,00%	12					rs5743409	1,00E-012
NA12716	reg_DEFA	chr8	6724531	G	A	48	52,00%	25			rs5743404			1,00E-012
NA12716	reg_DEFA	chr8	6724606	A	G	48	48,00%	23			rs2741136			1,00E-012

NA12716	reg_DEFA	chr8	6724645	A	G	46	48,00%	22	rs2741137			1,00E-012
NA12716	reg_DEFA	chr8	6724992	A	G	15	40,00%	6	rs5743401			6,20E-007
NA12716	reg_DEFA	chr8	6725055	G	A	12	33,00%	4	rs5743399			1,19E-004
NA12716	reg_DEFA	chr8	6725193	C	T	14	36,00%	5	rs2472143			1,08E-005
NA12716	reg_DEFA	chr8	6725224	G	A	15	33,00%	5	rs2977828			1,59E-005
NA12716	reg_DEFA	chr8	6725291	G	C	31	48,00%	15	rs2702881			1,00E-012
NA12716	reg_DEFA	chr8	6725524	T	C	27	33,00%	9	rs2738177			5,80E-009
NA12716	reg_DEFA	chr8	6725600	G	T	25	60,00%	15	rs2741138			1,00E-012
NA12716	reg_DEFA	chr8	6726157	A	G	7	43,00%	3	rs2741139			3,97E-004
NA12716	reg_DEFA	chr8	6726180	A	G	7	43,00%	3	rs2741140			3,97E-004
NA12716	reg_DEFA	chr8	6726302	T	C	14	29,00%	4	rs2702884			2,33E-004
NA12716	reg_DEFA	chr8	6726851	T	C	89	43,00%	38	rs2738175			1,00E-012
NA12716	reg_DEFA	chr8	6727011	G	A	71	52,00%	37	rs2741141			1,00E-012
NA12716	reg_DEFA	chr8	6727167	C	T	61	52,00%	32	rs2977827			1,00E-012
NA12716	reg_DEFA	chr8	6727332	G	A	97	49,00%	48	rs2978861			1,00E-012
NA12716	reg_DEFA	chr8	6727393	T	C	100	53,00%	53	rs2978860			1,00E-012
NA12716	reg_DEFA	chr8	6727484	C	T	98	53,00%	52	rs2977826			1,00E-012
NA12716	reg_DEFA	chr8	6727601	C	T	94	50,00%	47	rs2977825			1,00E-012
NA12716	reg_DEFA	chr8	6727790	G	A	38	42,00%	16			rs71214968	1,00E-012
NA12716	reg_DEFA	chr8	6727818	G	A	33	55,00%	18	rs9692818	rs9694351		1,00E-012
NA12716	reg_DEFA	chr8	6727819	T	C	34	71,00%	24	rs9694351			1,00E-012
NA12716	reg_DEFA	chr8	6727833	A	C	36	50,00%	18	rs9694106			1,00E-012
NA12716	reg_DEFA	chr8	6727846	A	G	39	74,00%	29	rs9694107			1,00E-012
NA12716	reg_DEFA	chr8	6727902	A	G	59	66,00%	39	rs9694118			1,00E-012
NA12716	reg_DEFA	chr8	6728005	G	T	69	65,00%	45	rs2978859			1,00E-012
NA12716	reg_DEFA	chr8	6728154	C	T	66	61,00%	40	rs2977824			1,00E-012
NA12716	reg_DEFA	chr8	6728378	A	G	54	48,00%	26	rs2741143			1,00E-012
NA12716	reg_DEFA	chr8	6728397	C	T	57	49,00%	28	rs2738172			1,00E-012
NA12716	reg_DEFA	chr8	6728537	T	C	50	44,00%	22	rs2738171			1,00E-012
NA12716	reg_DEFA	chr8	6728715	T	G	38	39,00%	15	rs2978857			1,00E-012
NA12716	reg_DEFA	chr8	6728719	A	G	38	39,00%	15	rs2951840			1,00E-012
NA12716	reg_DEFA	chr8	6728764	C	T	32	37,00%	12	rs2980919			6,54E-012
NA12716	reg_DEFA	chr8	6728770	T	C	31	42,00%	13	rs2978856			1,00E-012
NA12716	reg_DEFA	chr8	6728867	G	C	17	35,00%	6	rs2978855			1,47E-006
NA12716	reg_DEFA	chr8	6729141	A	G	14	50,00%	7	rs2977823			1,01E-008
NA12716	reg_DEFA	chr8	6729311	T	C	37	49,00%	18	rs2978853			1,00E-012
NA12716	reg_DEFA	chr8	6729458	C	T	51	45,00%	23	rs2977821			1,00E-012
NA12716	reg_DEFA	chr8	6729520	C	T	57	47,00%	27	rs2951850			1,00E-012
NA12716	reg_DEFA	chr8	6729801	G	A	100	49,00%	49	rs2978852			1,00E-012
NA12716	reg_DEFA	chr8	6730195	A	T	51	51,00%	26				1,00E-012
NA12716	reg_DEFA	chr8	6730978	A	G	36	100,00%	36	rs2738165			1,00E-012
NA12716	reg_DEFA	chr8	6731383	T	C	40	42,00%	17	rs2738164			1,00E-012
NA12716	reg_DEFA	chr8	6732145	G	T	34	50,00%	17			rs73517795	1,00E-012
NA12716	reg_DEFA	chr8	6732419	C	T	69	42,00%	29				1,00E-012
NA12716	reg_DEFA	chr8	6732626	C	T	96	56,00%	54	rs2738163			1,00E-012
NA12716	reg_DEFA	chr8	6734051	A	G	117	48,00%	56	rs2741718			1,00E-012
NA12716	reg_DEFA	chr8	6734149	G	A	108	100,00%	108	rs2978851			1,00E-012
NA12716	reg_DEFA	chr8	6735296	G	A	73	56,00%	41	rs2741717			1,00E-012
NA12716	reg_DEFA	chr8	6735747	A	C	64	55,00%	35	rs2738162			1,00E-012
NA12716	reg_DEFA	chr8	6736007	C	T	57	39,00%	22	rs2738161			1,00E-012
NA12716	reg_DEFA	chr8	6736585	C	T	80	54,00%	43	rs2738159			1,00E-012
NA12716	reg_DEFA	chr8	6737079	G	A	62	100,00%	62	rs2738158			1,00E-012
NA12716	reg_DEFA	chr8	6737661	T	C	68	56,00%	38	rs2738157			1,00E-012

NA12716	reg_DEFA	chr8	6738298	A	G	29	100,00%	29	rs2978964		1,00E-012
NA12716	reg_DEFA	chr8	6738591	G	A	51	100,00%	51	rs2951870		1,00E-012
NA12716	reg_DEFA	chr8	6738844	T	C	75	55,00%	41	rs2702936		1,00E-012
NA12716	reg_DEFA	chr8	6738879	C	T	77	44,00%	34	rs2738155		1,00E-012
NA12716	reg_DEFA	chr8	6739237	A	G	105	50,00%	53	rs2702935		1,00E-012
NA12716	reg_DEFA	chr8	6739310	T	G	96	36,00%	35	rs2738153		1,00E-012
NA12716	reg_DEFA	chr8	6739495	T	A	111	46,00%	51	rs2738152		1,00E-012
NA12716	reg_DEFA	chr8	6739788	T	C	78	55,00%	43	rs2741715		1,00E-012
NA12716	reg_DEFA	chr8	6739842	C	T	79	57,00%	45	rs2738149		1,00E-012
NA12716	reg_DEFA	chr8	6741958	C	A	79	99,00%	78	rs751009		1,00E-012
NA12716	reg_DEFA	chr8	6742329	C	G	82	38,00%	31	rs2738148		1,00E-012
NA12716	reg_DEFA	chr8	6742481	A	C	89	38,00%	34	rs2702911		1,00E-012
NA12716	reg_DEFA	chr8	6743172	C	G	55	40,00%	22	rs2738145		1,00E-012
NA12716	reg_DEFA	chr8	6743230	C	A	45	44,00%	20	rs2741056		1,00E-012
NA12716	reg_DEFA	chr8	6743367	A	G	22	45,00%	10	rs2978963		3,38E-011
NA12716	reg_DEFA	chr8	6743931	T	C	11	55,00%	6	rs2980959		6,19E-008
NA12716	reg_DEFA	chr8	6744173	A	C	13	62,00%	8	rs9693852		9,13E-011
NA12716	reg_DEFA	chr8	6744180	T	C	13	62,00%	8	rs13255719		9,13E-011
NA12716	reg_DEFA	chr8	6744212	G	A	13	62,00%	8	rs2741057		9,13E-011
NA12716	reg_DEFA	chr8	6745807	G	A	114	37,00%	42	rs2702929		1,00E-012
NA12716	reg_DEFA	chr8	6745889	A	C	125	37,00%	46	rs2702930		1,00E-012
NA12716	reg_DEFA	chr8	6746226	G	A	74	42,00%	31	rs2738143		1,00E-012
NA12716	reg_DEFA	chr8	6746266	A	T	69	42,00%	29	rs2741060		1,00E-012
NA12716	reg_DEFA	chr8	6746389	A	C	40	42,00%	17	rs2702931		1,00E-012
NA12716	reg_DEFA	chr8	6746793	T	C	13	38,00%	5	rs2702932		7,10E-006
NA12716	reg_DEFA	chr8	6746980	C	G	13	92,00%	12	rs2741061		1,00E-012
NA12716	reg_DEFA	chr8	6747103	C	G	13	85,00%	11	rs2741062		1,00E-012
NA12716	reg_DEFA	chr8	6747377	C	A	31	61,00%	19	rs2741714		1,00E-012
NA12716	reg_DEFA	chr8	6747535	A	C	40	100,00%	40	rs2951867		1,00E-012
NA12716	reg_DEFA	chr8	6747552	T	C	42	62,00%	26	rs2702933		1,00E-012
NA12716	reg_DEFA	chr8	6747562	C	T	45	40,00%	18	rs2741064		1,00E-012
NA12716	reg_DEFA	chr8	6747770	C	T	46	37,00%	17	rs2741065		1,00E-012
NA12716	reg_DEFA	chr8	6747968	A	G	36	39,00%	14	rs2738142		1,00E-012
NA12716	reg_DEFA	chr8	6748106	A	C	61	44,00%	27	rs2741713		1,00E-012
NA12716	reg_DEFA	chr8	6748195	A	G	70	40,00%	28	rs2738141		1,00E-012
NA12716	reg_DEFA	chr8	6748407	A	G	88	51,00%	45	rs2741068		1,00E-012
NA12716	reg_DEFA	chr8	6748447	C	G	86	44,00%	38	rs2738140		1,00E-012
NA12716	reg_DEFA	chr8	6748699	C	T	72	50,00%	36		rs55848359	1,00E-012
NA12716	reg_DEFA	chr8	6748878	T	C	100	43,00%	43	rs20530		1,00E-012
NA12716	reg_DEFA	chr8	6748942	A	G	122	46,00%	56	rs20528		1,00E-012
NA12716	reg_DEFA	chr8	6748981	A	G	119	48,00%	57	rs20527		1,00E-012
NA12716	reg_DEFA	chr8	6749191	G	A	102	49,00%	50	rs2741070		1,00E-012
NA12716	reg_DEFA	chr8	6749195	A	C	101	51,00%	52	rs2702886		1,00E-012
NA12716	reg_DEFA	chr8	6749262	T	G	88	41,00%	36	rs2738139		1,00E-012
NA12716	reg_DEFA	chr8	6749839	A	C	29	48,00%	14	rs2741712		1,00E-012
NA12716	reg_DEFA	chr8	6750400	G	C	42	50,00%	21	rs2741072		1,00E-012
NA12716	reg_DEFA	chr8	6750581	T	C	78	99,00%	77	rs2981405		1,00E-012
NA12716	reg_DEFA	chr8	6750651	A	G	92	53,00%	49	rs2741073		1,00E-012
NA12716	reg_DEFA	chr8	6750694	A	G	94	46,00%	43	rs2738137		1,00E-012
NA12716	reg_DEFA	chr8	6751071	C	T	69	45,00%	31	rs2741711		1,00E-012
NA12716	reg_DEFA	chr8	6751139	A	T	62	53,00%	33	rs2702887		1,00E-012
NA12716	reg_DEFA	chr8	6751207	C	T	58	98,00%	57	rs2741075		1,00E-012
NA12716	reg_DEFA	chr8	6751396	T	C	52	100,00%	52	rs2741710		1,00E-012

NA12716	reg_DEFA	chr8	6751654	C	T	22	55,00%	12	rs11782467		1,00E-012
NA12716	reg_DEFA	chr8	6751657	C	G	20	55,00%	11	rs35621199		1,00E-012
NA12716	reg_DEFA	chr8	6751684	T	C	19	63,00%	12	rs17078355		1,00E-012
NA12716	reg_DEFA	chr8	6751724	G	A	12	67,00%	8	rs35809894		4,67E-011
NA12716	reg_DEFA	chr8	6751748	T	A	12	67,00%	8			4,67E-011
NA12716	reg_DEFA	chr8	6751750	G	A	11	64,00%	7			1,04E-009
NA12716	reg_DEFA	chr8	6751808	T	C	11	55,00%	6	rs2980953		6,19E-008
NA12716	reg_DEFA	chr8	6752098	G	A	11	45,00%	5	rs2741079		2,65E-006
NA12716	reg_DEFA	chr8	6752323	C	T	25	48,00%	12	rs34412952		1,00E-012
NA12716	reg_DEFA	chr8	6752361	T	C	36	44,00%	16	rs10085957		1,00E-012
NA12716	reg_DEFA	chr8	6752442	G	C	51	45,00%	23	rs2702888		1,00E-012
NA12716	reg_DEFA	chr8	6752546	A	G	83	42,00%	35	rs2702889		1,00E-012
NA12716	reg_DEFA	chr8	6752550	A	G	84	43,00%	36	rs2741708		1,00E-012
NA12716	reg_DEFA	chr8	6752578	C	T	91	43,00%	39	rs2702890		1,00E-012
NA12716	reg_DEFA	chr8	6752677	C	T	109	43,00%	47		rs73194180	1,00E-012
NA12716	reg_DEFA	chr8	6752728	A	G	107	40,00%	43	rs2702891		1,00E-012
NA12716	reg_DEFA	chr8	6752872	G	A	108	45,00%	49	rs2702892		1,00E-012
NA12716	reg_DEFA	chr8	6752904	A	G	109	47,00%	51	rs2702893		1,00E-012
NA12716	reg_DEFA	chr8	6753127	A	G	69	38,00%	26	rs3888148		1,00E-012
NA12716	reg_DEFA	chr8	6753189	A	C	52	48,00%	25	rs6605602		1,00E-012
NA12716	reg_DEFA	chr8	6753230	G	T	46	39,00%	18	rs3888149		1,00E-012
NA12716	reg_DEFA	chr8	6753242	C	G	44	41,00%	18	rs3888150		1,00E-012
NA12716	reg_DEFA	chr8	6753261	C	G	46	39,00%	18	rs35516150		1,00E-012
NA12716	reg_DEFA	chr8	6753275	T	C	49	39,00%	19	rs34494557		1,00E-012
NA12716	reg_DEFA	chr8	6753286	C	T	49	59,00%	29			1,00E-012
NA12716	reg_DEFA	chr8	6753328	C	G	50	32,00%	16	rs36092179		1,00E-012
NA12716	reg_DEFA	chr8	6753376	A	G	54	35,00%	19	rs34166140		1,00E-012
NA12716	reg_DEFA	chr8	6753385	C	G	55	33,00%	18	rs36021248		1,00E-012
NA12716	reg_DEFA	chr8	6753589	G	C	64	44,00%	28	rs2951848		1,00E-012
NA12716	reg_DEFA	chr8	6753656	C	T	63	38,00%	24	rs2981404		1,00E-012
NA12716	reg_DEFA	chr8	6753661	C	G	67	42,00%	28	rs35504411		1,00E-012
NA12716	reg_DEFA	chr8	6753664	C	G	73	42,00%	31	rs2981403		1,00E-012
NA12716	reg_DEFA	chr8	6753731	G	A	82	44,00%	36	rs2951849		1,00E-012
NA12716	reg_DEFA	chr8	6753760	C	T	79	47,00%	37	rs2981402		1,00E-012
NA12716	reg_DEFA	chr8	6753858	A	G	61	36,00%	22	rs28533565		1,00E-012
NA12716	reg_DEFA	chr8	6753882	T	C	57	33,00%	19	rs34987687		1,00E-012
NA12716	reg_DEFA	chr8	6753915	G	A	47	26,00%	12	rs34087549		5,44E-010
NA12716	reg_DEFA	chr8	6753932	C	T	41	24,00%	10	rs34661040		2,42E-008
NA12716	reg_DEFA	chr8	6754046	A	C	25	24,00%	6		rs56174646	1,80E-005
NA12716	reg_DEFA	chr8	6754602	C	T	85	48,00%	41	rs2702898		1,00E-012
NA12716	reg_DEFA	chr8	6754803	G	A	89	53,00%	47	rs2702899		1,00E-012
NA12716	reg_DEFA	chr8	6755659	A	G	81	48,00%	39	rs2702900		1,00E-012
NA12716	reg_DEFA	chr8	6757796	A	G	55	53,00%	29	rs10216819		1,00E-012
NA12716	reg_DEFA	chr8	6758791	T	C	53	100,00%	53	rs2981401		1,00E-012
NA12716	reg_DEFA	chr8	6760323	T	G	62	53,00%	33	rs7461956		1,00E-012
NA12716	reg_DEFA	chr8	6761824	G	A	102	45,00%	46	rs2738133		1,00E-012
NA12716	reg_DEFA	chr8	6762080	C	T	61	52,00%	32	rs2741703		1,00E-012
NA12716	reg_DEFA	chr8	6762169	C	T	45	64,00%	29	rs2741701		1,00E-012
NA12716	reg_DEFA	chr8	6762964	G	A	65	100,00%	65	rs2741699		1,00E-012
NA12716	reg_DEFA	chr8	6762978	C	A	64	94,00%	60	rs2741698		1,00E-012
NA12716	reg_DEFA	chr8	6763134	A	G	43	56,00%	24	rs2741697		1,00E-012
NA12716	reg_DEFA	chr8	6763299	T	A	58	45,00%	26	rs2741696		1,00E-012
NA12716	reg_DEFA	chr8	6763722	G	A	59	100,00%	59	rs2741695		1,00E-012

NA12716	reg_DEFA	chr8	6764461	A	G	29	100,00%	29			rs2741694			1,00E-012
NA12716	reg_DEFA	chr8	6765176	G	A	63	48,00%	30			rs2738131			1,00E-012
NA12716	reg_DEFA	chr8	6765363	G	A	58	98,00%	57			rs2702946			1,00E-012
NA12716	reg_DEFA	chr8	6765676	A	G	53	57,00%	30			rs12545953			1,00E-012
NA12716	reg_DEFA	chr8	6765994	T	C	30	100,00%	30			rs2738129			1,00E-012
NA12716	reg_DEFA	chr8	6766076	A	C	43	98,00%	42			rs2738128			1,00E-012
NA12716	reg_DEFA	chr8	6766524	T	C	66	100,00%	66			rs2978959			1,00E-012
NA12716	reg_DEFA	chr8	6767164	C	T	58	48,00%	28			rs2738127			1,00E-012
NA12716	reg_DEFA	chr8	6767542	G	A	91	54,00%	49			rs2738126			1,00E-012
NA12716	reg_DEFA	chr8	6767654	T	C	69	48,00%	33			rs13275170			1,00E-012
NA12716	reg_DEFA	chr8	6767778	G	T	39	100,00%	39			rs2738125			1,00E-012
NA12716	reg_DEFA	chr8	6767823	A	C	31	29,00%	9			rs2738124			2,29E-008
NA12716	reg_DEFA	chr8	6767996	T	C	14	50,00%	7			rs13276112			1,01E-008
NA12716	reg_DEFA	chr8	6768647	T	A	46	50,00%	23			rs2702905			1,00E-012
NA12716	reg_DEFA	chr8	6769030	T	C	51	41,00%	21			rs2738122			1,00E-012
NA12716	reg_DEFA	chr8	6769096	T	C	40	25,00%	10			rs2738121			1,87E-008
NA12716	reg_DEFA	chr8	6769681	A	G	97	49,00%	48	-	DEFA6	rs712276			1,00E-012
NA12716	reg_DEFA	chr8	6770046	G	C	101	96,00%	97	-	DEFA6	rs2738120			1,00E-012
NA12716	reg_DEFA	chr8	6770127	G	C	82	100,00%	82	-	DEFA6	rs2738119			1,00E-012
NA12716	reg_DEFA	chr8	6770376	G	T	51	49,00%	25	-	DEFA6	rs2741691			1,00E-012
NA12716	reg_DEFA	chr8	6771046	G	T	80	50,00%	40			rs11784359			1,00E-012
NA12716	reg_DEFA	chr8	6771370	C	G	72	43,00%	31			rs4458901			1,00E-012
NA12716	reg_DEFA	chr8	6771627	C	G	64	42,00%	27			rs2741690			1,00E-012
NA12716	reg_DEFA	chr8	6771666	C	T	58	45,00%	26			rs2741689			1,00E-012
NA12716	reg_DEFA	chr8	6772381	G	T	14	57,00%	8			rs2738118			2,09E-010
NA12716	reg_DEFA	chr8	6773483	G	C	123	51,00%	63			rs2741686			1,00E-012
NA12716	reg_DEFA	chr8	6774191	T	C	36	53,00%	19			rs3918350			1,00E-012
NA12716	reg_DEFA	chr8	6774412	C	G	26	58,00%	15			rs34502430			1,00E-012
NA12716	reg_DEFA	chr8	6774485	T	C	24	54,00%	13			rs2702938			1,00E-012
NA12716	reg_DEFA	chr8	6774796	C	G	48	42,00%	20						1,00E-012
NA12716	reg_DEFA	chr8	6774810	T	C	50	54,00%	27			rs2702939			1,00E-012
NA12716	reg_DEFA	chr8	6775470	G	A	54	54,00%	29			rs2012832	rs2738114		1,00E-012
NA12716	reg_DEFA	chr8	6775499	C	G	57	40,00%	23			rs2077406			1,00E-012
NA12716	reg_DEFA	chr8	6775828	C	G	70	47,00%	33			rs2741684			1,00E-012
NA12716	reg_DEFA	chr8	6775890	T	C	75	49,00%	37			rs2741683			1,00E-012
NA12716	reg_DEFA	chr8	6776054	A	G	73	51,00%	37			rs2738111			1,00E-012
NA12716	reg_DEFA	chr8	6776610	T	C	82	48,00%	39			rs2702855			1,00E-012
NA12716	reg_DEFA	chr8	6776698	C	G	88	48,00%	42			rs3887306			1,00E-012
NA12716	reg_DEFA	chr8	6777064	A	T	70	46,00%	32			rs2738109			1,00E-012
NA12716	reg_DEFA	chr8	6777196	T	C	63	49,00%	31			rs2702858			1,00E-012
NA12716	reg_DEFA	chr8	6777240	G	A	58	98,00%	57			rs2977818			1,00E-012
NA12716	reg_DEFA	chr8	6777294	G	A	62	34,00%	21			rs13274891			1,00E-012
NA12716	reg_DEFA	chr8	6777394	T	C	51	41,00%	21			rs13250769			1,00E-012
NA12716	reg_DEFA	chr8	6777734	T	C	83	45,00%	37			rs2702861			1,00E-012
NA12716	reg_DEFA	chr8	6777962	C	T	82	44,00%	36					rs56248548	1,00E-012
NA12716	reg_DEFA	chr8	6778120	C	A	64	27,00%	17			rs13251447			1,00E-012
NA12716	reg_DEFA	chr8	6778433	A	G	39	100,00%	39					rs59334047	1,00E-012
NA12716	reg_DEFA	chr8	6779011	A	G	66	36,00%	24			rs13262140			1,00E-012
NA12716	reg_DEFA	chr8	6779125	A	G	60	42,00%	25			rs2738106			1,00E-012
NA12716	reg_DEFA	chr8	6779250	C	T	65	35,00%	23			rs13261705			1,00E-012
NA12716	reg_DEFA	chr8	6779667	G	A	73	45,00%	33			rs2738104			1,00E-012
NA12716	reg_DEFA	chr8	6779811	C	T	74	45,00%	33			rs13263461			1,00E-012
NA12716	reg_DEFA	chr8	6779861	C	G	71	48,00%	34			rs13263510			1,00E-012

NA12716	reg_DEFA	chr8	6779930	G	A	71	48,00%	34					rs2738103		1,00E-012
NA12716	reg_DEFA	chr8	6780050	A	G	75	48,00%	36					rs13254588		1,00E-012
NA12716	reg_DEFA	chr8	6780152	G	T	78	45,00%	35					rs13251814		1,00E-012
NA12716	reg_DEFA	chr8	6780481	G	A	105	48,00%	50					rs2738102		1,00E-012
NA12716	reg_DEFA	chr8	6780732	T	C	110	58,00%	64					rs2702867		1,00E-012
NA12716	reg_DEFA	chr8	6780950	C	T	109	50,00%	55			-	DEFA4	rs736227		1,00E-012
NA12716	reg_DEFA	chr8	6780991	A	G	105	46,00%	48	G	G	-2	DEFA4	rs2738100		1,00E-012
NA12716	reg_DEFA	chr8	6781429	G	A	91	51,00%	46			-	DEFA4	rs2239668		1,00E-012
NA12716	reg_DEFA	chr8	6781617	G	A	91	44,00%	40			-	DEFA4	rs2239667		1,00E-012
NA12716	reg_DEFA	chr8	6782247	C	T	88	40,00%	35			-	DEFA4	rs4273874		1,00E-012
NA12716	reg_DEFA	chr8	6782277	T	A	93	54,00%	50			-	DEFA4	rs2741679		1,00E-012
NA12716	reg_DEFA	chr8	6783611	C	T	89	51,00%	45					rs2741676		1,00E-012
NA12716	reg_DEFA	chr8	6784942	C	A	89	51,00%	45					rs2741675		1,00E-012
NA12716	reg_DEFA	chr8	6785122	C	T	90	91,00%	82					rs2741674		1,00E-012
NA12716	reg_DEFA	chr8	6786308	C	G	84	48,00%	40					rs2615772		1,00E-012
NA12716	reg_DEFA	chr8	6786381	C	A	72	50,00%	36					rs2741673		1,00E-012
NA12716	reg_DEFA	chr8	6786975	A	C	101	50,00%	51					rs3888293		1,00E-012
NA12716	reg_DEFA	chr8	6787981	A	G	64	55,00%	35					rs2741672		1,00E-012
NA12716	reg_DEFA	chr8	6788129	C	G	52	100,00%	52					rs2951853		1,00E-012
NA12716	reg_DEFA	chr8	6788271	C	T	57	44,00%	25						rs56136595	1,00E-012
NA12716	reg_DEFA	chr8	6789186	T	C	61	44,00%	27					rs41384045		1,00E-012
NA12716	reg_DEFA	chr8	6789260	A	C	56	39,00%	22						rs61038944	1,00E-012
NA12716	reg_DEFA	chr8	6789483	T	C	43	47,00%	20					rs2741669		1,00E-012
NA12716	reg_DEFA	chr8	6790039	A	G	61	49,00%	30					rs12056400		1,00E-012
NA12716	reg_DEFA	chr8	6790121	G	T	59	54,00%	32					rs12056552		1,00E-012
NA12716	reg_DEFA	chr8	6790937	T	C	87	51,00%	44					rs2741668		1,00E-012
NA12716	reg_DEFA	chr8	6791260	C	T	90	99,00%	89					rs2981396		1,00E-012
NA12716	reg_DEFA	chr8	6792714	T	C	78	42,00%	33					rs2702878		1,00E-012
NA12716	reg_DEFA	chr8	6792879	A	G	86	100,00%	86					rs2951844		1,00E-012
NA12716	reg_DEFA	chr8	6793624	A	G	92	48,00%	44					rs2702879		1,00E-012
NA12716	reg_DEFA	chr8	6793699	C	T	86	45,00%	39					rs2741665		1,00E-012
NA12716	reg_DEFA	chr8	6793824	A	T	68	49,00%	33					rs2741663		1,00E-012
NA12716	reg_DEFA	chr8	6794417	G	T	55	51,00%	28					rs2738088		1,00E-012
NA12716	reg_DEFA	chr8	6794720	T	G	59	47,00%	28					rs2978955		1,00E-012
NA12716	reg_DEFA	chr8	6795292	G	C	122	45,00%	55					rs2738086		1,00E-012
NA12716	reg_DEFA	chr8	6795430	A	T	94	48,00%	45					rs2741661		1,00E-012
NA12716	reg_DEFA	chr8	6796132	T	G	78	60,00%	47					rs2075836		1,00E-012
NA12716	reg_DEFA	chr8	6797186	T	C	76	45,00%	34					rs2741660		1,00E-012
NA12716	reg_DEFA	chr8	6797410	T	G	84	51,00%	43					rs2702907		1,00E-012
NA12716	reg_DEFA	chr8	6797578	T	C	81	48,00%	39					rs2741659		1,00E-012
NA12716	reg_DEFA	chr8	6798559	T	G	102	57,00%	58					rs2615787		1,00E-012
NA12716	reg_DEFA	chr8	6798969	C	T	88	45,00%	40					rs2741658		1,00E-012
NA12716	reg_DEFA	chr8	6799532	G	T	63	35,00%	22					rs2702850		1,00E-012
NA12716	reg_DEFA	chr8	6799608	A	G	54	44,00%	24					rs2741657		1,00E-012
NA12716	reg_DEFA	chr8	6799659	G	T	51	41,00%	21					rs2472562		1,00E-012
NA12716	reg_DEFA	chr8	6799831	T	C	27	41,00%	11					rs2741653		1,60E-011
NA12716	reg_DEFA	chr8	6800036	T	G	34	53,00%	18					rs2702851		1,00E-012
NA12716	reg_DEFA	chr8	6800169	A	G	65	46,00%	30					rs2615771		1,00E-012
NA12716	reg_DEFA	chr8	6800833	G	A	85	40,00%	34							1,00E-012
NA12716	reg_DEFA	chr8	6800911	T	G	76	54,00%	41					rs2702852		1,00E-012
NA12716	reg_DEFA	chr8	6802066	T	C	74	50,00%	37					rs2977812		1,00E-012
NA12716	reg_DEFA	chr8	6802738	A	C	62	48,00%	30					rs2738082		1,00E-012
NA12716	reg_DEFA	chr8	6802928	G	T	58	48,00%	28					rs2738081		1,00E-012

NA12716	reg_DEFA	chr8	6803862	C	G	107	46,00%	49			rs10503361		1,00E-012
NA12716	reg_DEFA	chr8	6804222	C	A	116	43,00%	50			rs2738079		1,00E-012
NA12716	reg_DEFA	chr8	6804434	A	G	148	44,00%	65			rs2738078		1,00E-012
NA12716	reg_DEFA	chr8	6805574	A	G	79	40,00%	32			rs2738071		1,00E-012
NA12716	reg_DEFA	chr8	6805746	T	C	86	36,00%	31			rs2738069		1,00E-012
NA12716	reg_DEFA	chr8	6805887	G	A	81	36,00%	29			rs2738068		1,00E-012
NA12716	reg_DEFA	chr8	6806185	A	G	68	53,00%	36			rs2738067		1,00E-012
NA12716	reg_DEFA	chr8	6806801	T	A	34	50,00%	17			rs2702853		1,00E-012
NA12716	reg_DEFA	chr8	6807411	C	T	29	48,00%	14			rs2738066		1,00E-012
NA12716	reg_DEFA	chr8	6807433	C	G	28	50,00%	14			rs6983753		1,00E-012
NA12716	reg_DEFA	chr8	6807488	T	C	32	50,00%	16			rs6995959		1,00E-012
NA12716	reg_DEFA	chr8	6807874	T	G	60	57,00%	34			rs12680018		1,00E-012
NA12716	reg_DEFA	chr8	6808715	A	G	106	46,00%	49			rs4840647		1,00E-012
NA12716	reg_DEFA	chr8	6808829	T	C	129	99,00%	128			rs2977793		1,00E-012
NA12716	reg_DEFA	chr8	6809027	T	C	118	54,00%	64			rs2738058		1,00E-012
NA12716	reg_DEFA	chr8	6809450	G	A	72	100,00%	72			rs2951869		1,00E-012
NA12716	reg_DEFA	chr8	6809904	T	C	35	94,00%	33			rs2977789		1,00E-012
NA12716	reg_DEFA	chr8	6809966	A	C	40	100,00%	40			rs2977788		1,00E-012
NA12716	reg_DEFA	chr8	6810705	A	G	86	94,00%	81			rs2978951		1,00E-012
NA12716	reg_DEFA	chr8	6810952	C	A	71	48,00%	34			rs2738046		1,00E-012
NA12716	reg_DEFA	chr8	6810975	C	G	78	49,00%	38			rs2702912		1,00E-012
NA12716	reg_DEFA	chr8	6810978	T	C	77	49,00%	38			rs2738045		1,00E-012
NA12716	reg_DEFA	chr8	6811987	G	T	63	52,00%	33			rs2738168		1,00E-012
NA12716	reg_DEFA	chr8	6812041	G	T	56	55,00%	31			rs13257112		1,00E-012
NA12716	reg_DEFA	chr8	6812694	G	C	94	40,00%	38			rs2978950		1,00E-012
NA12716	reg_DEFA	chr8	6812705	G	A	102	55,00%	56			rs6996047		1,00E-012
NA12716	reg_DEFA	chr8	6812906	G	C	90	52,00%	47			rs11996346		1,00E-012
NA12716	reg_DEFA	chr8	6812946	C	G	92	40,00%	37			rs4543566		1,00E-012
NA12716	reg_DEFA	chr8	6813287	T	G	97	43,00%	42	-	DEFA10P	rs13278390		1,00E-012
NA12716	reg_DEFA	chr8	6813295	A	G	93	41,00%	38	-	DEFA10P	rs13270884		1,00E-012
NA12716	reg_DEFA	chr8	6813301	G	A	92	42,00%	39	-	DEFA10P	rs13267882		1,00E-012
NA12716	reg_DEFA	chr8	6813385	T	G	74	38,00%	28	-	DEFA10P	rs13278672		1,00E-012
NA12716	reg_DEFA	chr8	6813408	A	G	72	33,00%	24	-	DEFA10P	rs13271399		1,00E-012
NA12716	reg_DEFA	chr8	6813420	C	G	72	29,00%	21	-	DEFA10P	rs13270339		1,00E-012
NA12716	reg_DEFA	chr8	6813435	A	G	69	26,00%	18	-	DEFA10P	rs13271426		1,00E-012
NA12716	reg_DEFA	chr8	6813460	C	T	65	20,00%	13	-	DEFA10P	rs13270374		2,71E-009
NA12716	reg_DEFA	chr8	6813467	T	C	63	21,00%	13	-	DEFA10P	rs13278935		1,80E-009
NA12716	reg_DEFA	chr8	6813491	A	G	54	17,00%	9	-	DEFA10P		rs71525777	3,75E-006
NA12716	reg_DEFA	chr8	6813499	A	G	53	100,00%	53	-	DEFA10P	rs2978947		1,00E-012
NA12716	reg_DEFA	chr8	6815365	C	G	40	17,00%	7					3,26E-005
NA12716	reg_DEFA	chr8	6815387	C	T	44	18,00%	8					6,62E-006
NA12716	reg_DEFA	chr8	6815411	G	A	43	16,00%	7					5,30E-005
NA12716	reg_DEFA	chr8	6815430	A	C	46	22,00%	10					7,91E-008
NA12716	reg_DEFA	chr8	6815437	C	A	47	23,00%	11					7,73E-009
NA12716	reg_DEFA	chr8	6815442	G	C	48	23,00%	11					9,81E-009
NA12716	reg_DEFA	chr8	6815446	T	G	48	23,00%	11					9,81E-009
NA12716	reg_DEFA	chr8	6815845	G	A	72	35,00%	25			rs10088393		1,00E-012
NA12716	reg_DEFA	chr8	6816495	G	T	35	57,00%	20			rs2738113		1,00E-012
NA12716	reg_DEFA	chr8	6816665	C	A	36	47,00%	17			rs2738107		1,00E-012
NA12716	reg_DEFA	chr8	6817036	T	C	106	17,00%	18			rs2738101		5,72E-011
NA12716	reg_DEFA	chr8	6817114	C	T	136	15,00%	20			rs2615789		6,14E-011
NA12716	reg_DEFA	chr8	6817149	T	A	135	13,00%	18			rs2738099		2,64E-009
NA12716	reg_DEFA	chr8	6817203	C	T	118	11,00%	13			rs2702917		3,69E-006

NA12716	reg_DEFA	chr8	6817334	A	G	106	16,00%	17			rs2738097			4,04E-010
NA12716	reg_DEFA	chr8	6817394	C	G	101	16,00%	16			rs4841790			1,54E-009
NA12716	reg_DEFA	chr8	6817412	C	T	95	15,00%	14			rs2978911			4,12E-008
NA12716	reg_DEFA	chr8	6817429	A	G	86	13,00%	11			rs2738096			4,76E-006
NA12716	reg_DEFA	chr8	6817438	G	A	82	13,00%	11			rs2978910			2,96E-006
NA12716	reg_DEFA	chr8	6817564	T	C	57	23,00%	13			rs2738094			4,82E-010
NA12716	reg_DEFA	chr8	6818357	G	A	43	44,00%	19			rs2738091			1,00E-012
NA12716	reg_DEFA	chr8	6818361	C	T	43	44,00%	19			rs2738090	rs2447474		1,00E-012
NA12716	reg_DEFA	chr8	6818374	C	T	36	50,00%	18			rs2702915	rs2738089		1,00E-012
NA12716	reg_DEFA	chr8	6818375	G	A	36	47,00%	17			rs2738089			1,00E-012
NA12716	reg_DEFA	chr8	6818397	C	T	25	48,00%	12			rs2927351			1,00E-012
NA12716	reg_DEFA	chr8	6818535	T	A	69	38,00%	26			rs2615784	rs28647412		1,00E-012
NA12716	reg_DEFA	chr8	6818566	A	C	87	53,00%	46					rs62488547	1,00E-012
NA12716	reg_DEFA	chr8	6818614	A	G	103	38,00%	39			rs11305893			1,00E-012
NA12716	reg_DEFA	chr8	6818706	G	C	125	50,00%	63			rs2702914			1,00E-012
NA12716	reg_DEFA	chr8	6818828	C	A	112	25,00%	28						1,00E-012
NA12716	reg_DEFA	chr8	6818877	C	A	101	43,00%	43			rs28694551	rs2927350		1,00E-012
NA12716	reg_DEFA	chr8	6818898	A	G	97	44,00%	43			rs2927349			1,00E-012
NA12716	reg_DEFA	chr8	6818919	C	T	92	42,00%	39			rs28693063			1,00E-012
NA12716	reg_DEFA	chr8	6818941	A	G	89	43,00%	38			rs7460147			1,00E-012
NA12716	reg_DEFA	chr8	6818949	G	A	90	42,00%	38						1,00E-012
NA12716	reg_DEFA	chr8	6819003	C	T	79	58,00%	46			rs2977813			1,00E-012
NA12716	reg_DEFA	chr8	6819005	A	G	78	44,00%	34			rs2978906			1,00E-012
NA12716	reg_DEFA	chr8	6820626	C	A	180	50,00%	90					rs59243516	1,00E-012
NA12716	reg_DEFA	chr8	6820983	G	A	96	93,00%	89			rs2615779			1,00E-012
NA12716	reg_DEFA	chr8	6821065	G	A	50	22,00%	11						1,55E-008
NA12716	reg_DEFA	chr8	6821169	T	C	9	89,00%	8			rs10108690			1,00E-012
NA12716	reg_DEFA	chr8	6821178	A	G	9	89,00%	8			rs12381526			1,00E-012
NA12716	reg_DEFA	chr8	6824570	C	A	22	95,00%	21	-	DEFA1B	rs2979395	rs3758132		1,00E-012
NA12716	reg_DEFA	chr8	6824741	G	A	24	96,00%	23	-	DEFA1B			rs58955451	1,00E-012
NA12716	reg_DEFA	chr8	6825349	C	T	108	32,00%	35			rs2978854			1,00E-012
NA12716	reg_DEFA	chr8	6825357	G	A	107	33,00%	35			rs2951835			1,00E-012
NA12716	reg_DEFA	chr8	6825678	A	G	32	97,00%	31			rs6993352			1,00E-012
NA12716	reg_DEFA	chr8	6825754	C	T	17	88,00%	15			rs6986023			1,00E-012
NA12716	reg_DEFA	chr8	6825821	C	T	7	71,00%	5						1,30E-007
NA12716	reg_DEFA	chr8	6843886	T	A	206	11,00%	23	-	DEFA1B				6,44E-010
NA12716	reg_DEFA	chr8	6844196	G	T	102	22,00%	22						1,00E-012
NA12716	reg_DEFA	chr8	6844623	A	C	179	29,00%	52			rs28515027			1,00E-012
NA12716	reg_DEFA	chr8	6844915	C	T	23	30,00%	7					rs71509223	6,03E-007
NA12716	reg_DEFA	chr8	6846612	G	A	87	24,00%	21			rs4484718			1,00E-012
NA12716	reg_DEFA	chr8	6846709	T	C	131	59,00%	77			rs3758131			1,00E-012
NA12716	reg_DEFA	chr8	6859021	A	C	5	60,00%	3					rs71525791	1,18E-004
NA12716	reg_DEFA	chr8	6859147	G	T	52	92,00%	48					rs2615778	1,00E-012
NA12716	reg_DEFA	chr8	6859203	G	A	86	94,00%	81			rs28532282			1,00E-012
NA12716	reg_DEFA	chr8	6859285	G	A	146	22,00%	32						1,00E-012
NA12716	reg_DEFA	chr8	6859444	T	C	190	31,00%	59			rs2951846			1,00E-012
NA12716	reg_DEFA	chr8	6859619	T	G	146	46,00%	67			rs2739221			1,00E-012
NA12716	reg_DEFA	chr8	6859843	G	A	154	31,00%	48			rs2739220			1,00E-012
NA12716	reg_DEFA	chr8	6859866	A	G	159	16,00%	25					rs4841796	1,00E-012
NA12716	reg_DEFA	chr8	6860098	C	T	121	21,00%	25			rs10105775			1,00E-012
NA12716	reg_DEFA	chr8	6860180	A	G	103	11,00%	11						2,72E-005
NA12716	reg_DEFA	chr8	6860244	G	T	74	36,00%	27			rs10105163			1,00E-012
NA12716	reg_DEFA	chr8	6860274	C	T	73	60,00%	44			rs9694309			1,00E-012

NA12716	reg_DEFA	chr8	6861013	T	G	136	19,00%	26	D	A	-2	DEFA1	rs2230231		1,00E-012
NA12716	reg_DEFA	chr8	6861144	T	G	180	16,00%	29			-	DEFA1B		rs4840655	1,00E-012
NA12716	reg_DEFA	chr8	6861269	A	T	205	39,00%	80			-	DEFA1B	rs2702913		1,00E-012
NA12716	reg_DEFA	chr8	6862522	A	G	15	100,00%	15			-	DEFA1B	rs4841798		1,00E-012
NA12716	reg_DEFA	chr8	6863605	C	T	122	44,00%	54							1,00E-012
NA12716	reg_DEFA	chr8	6864445	T	C	137	18,00%	25						rs62487509	1,00E-012
NA12716	reg_DEFA	chr8	6864484	A	G	142	15,00%	21					rs4102677		2,43E-011
NA12716	reg_DEFA	chr8	6864926	T	C	149	52,00%	77					rs2739218		1,00E-012
NA12716	reg_DEFA	chr8	6864955	A	T	141	21,00%	30					rs9650669		1,00E-012
NA12716	reg_DEFA	chr8	6864962	G	A	141	45,00%	63					rs2615770		1,00E-012
NA12716	reg_DEFA	chr8	6865114	G	C	80	27,00%	22					rs4012963		1,00E-012
NA12716	reg_DEFA	chr8	6865153	G	A	67	25,00%	17					rs4012962		1,00E-012
NA12716	reg_DEFA	chr8	6865359	A	T	126	11,00%	14						rs7839709	1,43E-006
NA12716	reg_DEFA	chr8	6865813	T	C	92	20,00%	18					rs11781199		1,25E-011
NA12716	reg_DEFA	chr8	6865875	T	C	100	22,00%	22					rs11781205		1,00E-012
NA12716	reg_DEFA	chr8	6865966	T	C	99	29,00%	29					rs11781229		1,00E-012
NA12716	reg_DEFA	chr8	6866059	A	T	91	31,00%	28						rs56016462	1,00E-012
NA12716	reg_DEFA	chr8	6866080	G	C	94	29,00%	27						rs55836016	1,00E-012
NA12716	reg_DEFA	chr8	6866206	A	C	96	28,00%	27						rs55842276	1,00E-012
NA12716	reg_DEFA	chr8	6866221	C	T	95	28,00%	27							1,00E-012
NA12716	reg_DEFA	chr8	6866222	G	A	93	29,00%	27							1,00E-012
NA12716	reg_DEFA	chr8	6866255	C	T	98	31,00%	30						rs73661330	1,00E-012
NA12716	reg_DEFA	chr8	6866345	A	G	100	34,00%	34						rs59539636	1,00E-012
NA12716	reg_DEFA	chr8	6866462	G	C	84	19,00%	16							9,04E-011
NA12716	reg_DEFA	chr8	6866888	G	C	18	100,00%	18					rs35858635		1,00E-012
NA12716	reg_DEFA	chr8	6866891	G	C	19	100,00%	19					rs34985860		1,00E-012
NA12716	reg_DEFA	chr8	6866895	G	C	20	100,00%	20					rs35564068		1,00E-012
NA12716	reg_DEFA	chr8	6866897	G	C	20	95,00%	19					rs35820601		1,00E-012
NA12716	reg_DEFA	chr8	6866947	G	C	21	95,00%	20					rs4310228		1,00E-012
NA12716	reg_DEFA	chr8	6869398	T	C	74	93,00%	69					rs4313182		1,00E-012
NA12716	reg_DEFA	chr8	6869803	C	G	81	49,00%	40					rs883182		1,00E-012
NA12716	reg_DEFA	chr8	6869887	A	T	98	99,00%	97					rs4481622		1,00E-012
NA12716	reg_DEFA	chr8	6870176	T	G	97	99,00%	96					rs4314670		1,00E-012
NA12716	reg_DEFA	chr8	6870220	G	T	97	99,00%	96					rs4332158		1,00E-012
NA12716	reg_DEFA	chr8	6870259	G	A	89	97,00%	86					rs4332159		1,00E-012
NA12716	reg_DEFA	chr8	6870678	C	A	90	43,00%	39					rs4448290		1,00E-012
NA12716	reg_DEFA	chr8	6870686	G	C	90	97,00%	87					rs4469481		1,00E-012
NA12716	reg_DEFA	chr8	6870701	A	G	89	99,00%	88					rs9774483		1,00E-012
NA12716	reg_DEFA	chr8	6871265	A	G	65	100,00%	65					rs4840665		1,00E-012
NA12716	reg_DEFA	chr8	6872288	T	C	83	40,00%	33					rs6984215		1,00E-012
NA12716	reg_DEFA	chr8	6872534	A	C	66	100,00%	66					rs6605579		1,00E-012
NA12716	reg_DEFA	chr8	6873321	T	A	112	46,00%	52					rs7820625		1,00E-012
NA12716	reg_DEFA	chr8	6873505	A	G	133	50,00%	67					rs7816622		1,00E-012
NA12716	reg_DEFA	chr8	6874265	A	T	65	100,00%	65					rs7821152		1,00E-012
NA12716	reg_DEFA	chr8	6874382	G	A	52	44,00%	23					rs4403430		1,00E-012
NA12716	reg_DEFA	chr8	6874473	T	G	64	100,00%	64					rs11137086		1,00E-012
NA12716	reg_DEFA	chr8	6875301	C	T	42	52,00%	22						rs62487515	1,00E-012
NA12716	reg_DEFA	chr8	6875366	G	A	40	95,00%	38					rs6982814		1,00E-012
NA12716	reg_DEFA	chr8	6875538	C	A	68	46,00%	31						rs56230231	1,00E-012
NA12716	reg_DEFA	chr8	6875544	T	C	69	51,00%	35						rs55851618	1,00E-012
NA12716	reg_DEFA	chr8	6875673	C	G	73	52,00%	38						rs55660132	1,00E-012
NA12716	reg_DEFA	chr8	6875975	A	G	60	55,00%	33						rs55740316	1,00E-012
NA12716	reg_DEFA	chr8	6877006	A	T	42	95,00%	40					rs7011708		1,00E-012

NA12716	reg_DEFA	chr8	6877045	G	A	44	57,00%	25	rs11776120		1,00E-012
NA12716	reg_DEFA	chr8	6877291	A	G	43	56,00%	24	rs11786781		1,00E-012
NA12716	reg_DEFA	chr8	6877358	G	C	41	100,00%	41	rs6993492		1,00E-012
NA12716	reg_DEFA	chr8	6877487	T	C	42	64,00%	27	rs34825638		1,00E-012
NA12716	reg_DEFA	chr8	6877608	G	A	48	100,00%	48	rs7824527		1,00E-012
NA12716	reg_DEFA	chr8	6877988	G	T	46	98,00%	45	rs7825124		1,00E-012
NA12716	reg_DEFA	chr8	6878312	T	C	22	100,00%	22	rs4490865		1,00E-012
NA12716	reg_DEFA	chr8	6879022	T	G	78	96,00%	75	rs4841816		1,00E-012
NA12716	reg_DEFA	chr8	6879381	C	G	109	50,00%	55	rs4433170		1,00E-012
NA12716	reg_DEFA	chr8	6879503	C	G	73	49,00%	36	rs4300028		1,00E-012
NA12716	reg_DEFA	chr8	6879828	A	G	47	98,00%	46	rs4602905		1,00E-012
NA12716	reg_DEFA	chr8	6881033	C	T	19	100,00%	19	rs6605580		1,00E-012
NA12716	reg_DEFA	chr8	6881147	G	A	16	75,00%	12	rs6605581		1,00E-012
NA12716	reg_DEFA	chr8	6881163	G	A	17	94,00%	16	rs7014712		1,00E-012
NA12716	reg_DEFA	chr8	6881365	G	T	49	98,00%	48	rs17078546		1,00E-012
NA12716	reg_DEFA	chr8	6881460	G	A	55	96,00%	53	rs7015200		1,00E-012
NA12716	reg_DEFA	chr8	6881573	G	A	77	56,00%	43	rs11775034		1,00E-012
NA12716	reg_DEFA	chr8	6881816	T	C	98	99,00%	97	rs7004995		1,00E-012
NA12716	reg_DEFA	chr8	6882227	G	T	36	92,00%	33	rs34333583		1,00E-012
NA12716	reg_DEFA	chr8	6882354	T	A	20	90,00%	18	rs35708338		1,00E-012
NA12716	reg_DEFA	chr8	6882359	G	T	18	100,00%	18	rs35866869		1,00E-012
NA12716	reg_DEFA	chr8	6882374	T	C	14	100,00%	14	rs34268546		1,00E-012
NA12716	reg_DEFA	chr8	6882401	G	C	13	100,00%	13	rs34599455		1,00E-012
NA12716	reg_DEFA	chr8	6882406	T	A	13	38,00%	5		rs62488970	7,10E-006
NA12716	reg_DEFA	chr8	6882583	C	G	17	47,00%	8		rs2515504	1,60E-009
NA12716	reg_DEFA	chr8	6882620	T	C	18	56,00%	10	rs7009952		2,29E-012
NA12716	reg_DEFA	chr8	6882669	G	T	16	69,00%	11	rs6982904		1,00E-012
NA12716	reg_DEFA	chr8	6882937	G	C	43	98,00%	42	rs34219797		1,00E-012
NA12716	reg_DEFA	chr8	6883103	C	T	32	94,00%	30	rs11994868		1,00E-012
NA12716	reg_DEFA	chr8	6883131	T	C	32	97,00%	31	rs11985027		1,00E-012
NA12716	reg_DEFA	chr8	6883151	T	G	29	97,00%	28	rs11985030		1,00E-012
NA12716	reg_DEFA	chr8	6883232	T	C	24	100,00%	24	rs11985068		1,00E-012
NA12716	reg_DEFA	chr8	6883286	T	C	26	100,00%	26	rs11985076		1,00E-012
NA12716	reg_DEFA	chr8	6883469	A	C	52	94,00%	49	rs4841817		1,00E-012
NA12716	reg_DEFA	chr8	6883493	G	T	56	96,00%	54	rs4840666		1,00E-012
NA12716	reg_DEFA	chr8	6883556	G	C	73	96,00%	70	rs17078556		1,00E-012
NA12716	reg_DEFA	chr8	6883571	T	G	77	97,00%	75	rs35196527		1,00E-012
NA12716	reg_DEFA	chr8	6883577	A	G	79	97,00%	77	rs34725312		1,00E-012
NA12716	reg_DEFA	chr8	6883643	C	T	82	98,00%	80	rs4621824		1,00E-012
NA12716	reg_DEFA	chr8	6883724	A	G	93	98,00%	91	rs4345578		1,00E-012
NA12716	reg_DEFA	chr8	6883754	T	C	95	98,00%	93	rs4342629		1,00E-012
NA12716	reg_DEFA	chr8	6883823	G	C	98	100,00%	98	rs4342630		1,00E-012
NA12716	reg_DEFA	chr8	6883831	G	C	99	100,00%	99	rs4342631		1,00E-012
NA12716	reg_DEFA	chr8	6883942	C	T	86	98,00%	84	rs4549798		1,00E-012
NA12716	reg_DEFA	chr8	6883982	A	C	84	98,00%	82	rs4342632		1,00E-012
NA12716	reg_DEFA	chr8	6884098	A	T	91	52,00%	47	rs13256091		1,00E-012
NA12716	reg_DEFA	chr8	6884242	G	T	75	97,00%	73	rs4601339		1,00E-012
NA12716	reg_DEFA	chr8	6884293	G	C	72	53,00%	38	rs4367573		1,00E-012
NA12716	reg_DEFA	chr8	6884304	G	T	69	99,00%	68	rs4601340		1,00E-012
NA12716	reg_DEFA	chr8	6884370	C	A	62	100,00%	62	rs4642671		1,00E-012
NA12716	reg_DEFA	chr8	6884762	G	C	66	45,00%	30	rs4840668		1,00E-012
NA12716	reg_DEFA	chr8	6884922	G	A	69	45,00%	31	rs4240690		1,00E-012
NA12716	reg_DEFA	chr8	6885295	C	T	48	40,00%	19	rs4418364		1,00E-012

NA12716	reg_DEFA	chr8	6885310	C	T	47	96,00%	45	rs4546682		1,00E-012
NA12716	reg_DEFA	chr8	6885444	G	A	44	59,00%	26	rs6981771		1,00E-012
NA12716	reg_DEFA	chr8	6885525	C	T	65	52,00%	34	rs6996729		1,00E-012
NA12716	reg_DEFA	chr8	6885553	C	T	66	98,00%	65	rs6996890		1,00E-012
NA12716	reg_DEFA	chr8	6885601	C	A	65	48,00%	31	rs6996918		1,00E-012
NA12716	reg_DEFA	chr8	6885642	A	G	70	46,00%	32	rs7004474		1,00E-012
NA12716	reg_DEFA	chr8	6885716	T	A	77	45,00%	35	rs7009276		1,00E-012
NA12716	reg_DEFA	chr8	6885754	C	T	77	47,00%	36	rs6997211		1,00E-012
NA12716	reg_DEFA	chr8	6885796	T	G	72	96,00%	69	rs4841818		1,00E-012
NA12716	reg_DEFA	chr8	6886141	A	G	95	96,00%	91	rs4358823		1,00E-012
NA12716	reg_DEFA	chr8	6886408	T	C	85	47,00%	40	rs12716641		1,00E-012
NA12716	reg_DEFA	chr8	6886563	G	A	70	50,00%	35	rs12716642		1,00E-012
NA12716	reg_DEFA	chr8	6886619	G	A	58	52,00%	30	rs13274544		1,00E-012
NA12716	reg_DEFA	chr8	6886896	C	A	43	40,00%	17	rs12716643		1,00E-012
NA12716	reg_DEFA	chr8	6886899	C	T	42	38,00%	16	rs12716644		1,00E-012
NA12716	reg_DEFA	chr8	6887255	A	C	54	94,00%	51	rs7843319		1,00E-012
NA12716	reg_DEFA	chr8	6887285	C	G	57	96,00%	55	rs7836636		1,00E-012
NA12716	reg_DEFA	chr8	6887373	C	T	64	92,00%	59	rs7836778		1,00E-012
NA12716	reg_DEFA	chr8	6887405	A	C	70	93,00%	65	rs41514044	rs7843493	1,00E-012
NA12716	reg_DEFA	chr8	6887848	C	A	86	93,00%	80	rs12674716		1,00E-012
NA12716	reg_DEFA	chr8	6887922	G	T	75	87,00%	65	rs13250252		1,00E-012
NA12716	reg_DEFA	chr8	6887979	C	G	71	96,00%	68	rs13252474		1,00E-012
NA12716	reg_DEFA	chr8	6888029	T	A	65	94,00%	61	rs12716645		1,00E-012
NA12716	reg_DEFA	chr8	6888052	G	A	62	95,00%	59	rs13257504		1,00E-012
NA12716	reg_DEFA	chr8	6888345	T	G	28	96,00%	27	rs10108420		1,00E-012
NA12716	reg_DEFA	chr8	6888430	G	A	24	96,00%	23	rs4841819		1,00E-012
NA12716	reg_DEFA	chr8	6888510	C	T	24	92,00%	22	rs4841820		1,00E-012
NA12716	reg_DEFA	chr8	6888714	G	C	45	93,00%	42	rs12716647		1,00E-012
NA12716	reg_DEFA	chr8	6888737	C	G	46	96,00%	44	rs13261710		1,00E-012
NA12716	reg_DEFA	chr8	6888783	C	A	60	97,00%	58	rs13261750		1,00E-012
NA12716	reg_DEFA	chr8	6888786	A	G	61	97,00%	59	rs13262801		1,00E-012
NA12716	reg_DEFA	chr8	6888796	G	A	60	90,00%	54	rs13259722		1,00E-012
NA12716	reg_DEFA	chr8	6888800	T	G	63	90,00%	57	rs13270539		1,00E-012
NA12716	reg_DEFA	chr8	6889029	C	G	78	99,00%	77	rs4304345		1,00E-012
NA12716	reg_DEFA	chr8	6889141	C	T	71	97,00%	69	rs7017585		1,00E-012
NA12716	reg_DEFA	chr8	6889250	C	G	69	94,00%	65	rs7017866		1,00E-012
NA12716	reg_DEFA	chr8	6889297	T	C	63	97,00%	61	rs6992098		1,00E-012
NA12716	reg_DEFA	chr8	6889488	T	G	70	94,00%	66	rs13279261		1,00E-012
NA12716	reg_DEFA	chr8	6889853	A	T	61	93,00%	57	rs6988346		1,00E-012
NA12716	reg_DEFA	chr8	6890032	C	G	63	90,00%	57	rs6981058		1,00E-012
NA12716	reg_DEFA	chr8	6890057	T	A	63	95,00%	60	rs4841822		1,00E-012
NA12716	reg_DEFA	chr8	6890122	T	C	65	94,00%	61	rs4841823		1,00E-012
NA12716	reg_DEFA	chr8	6891017	C	A	60	98,00%	59	rs12682030		1,00E-012
NA12716	reg_DEFA	chr8	6891039	G	T	66	91,00%	60	rs13269815		1,00E-012
NA12716	reg_DEFA	chr8	6891182	A	G	69	91,00%	63	rs6998006		1,00E-012
NA12716	reg_DEFA	chr8	6891217	C	G	63	92,00%	58	rs6990416		1,00E-012
NA12716	reg_DEFA	chr8	6891225	T	G	61	93,00%	57	rs7002813		1,00E-012
NA12716	reg_DEFA	chr8	6891463	G	T	78	96,00%	75	rs28480342		1,00E-012
NA12716	reg_DEFA	chr8	6891605	A	C	89	97,00%	86	rs6998687		1,00E-012
NA12716	reg_DEFA	chr8	6891649	G	A	80	96,00%	77	rs7014280		1,00E-012
NA12716	reg_DEFA	chr8	6891711	C	A	78	94,00%	73	rs6991235		1,00E-012
NA12716	reg_DEFA	chr8	6891919	A	G	60	97,00%	58	rs6999181		1,00E-012
NA12716	reg_DEFA	chr8	6892865	T	C	34	88,00%	30	rs13257750		1,00E-012

NA12716	reg_DEFA	chr8	6892895	C	G	34	88,00%	30					rs13249237			1,00E-012
NA12716	reg_DEFA	chr8	6893048	T	C	22	95,00%	21					rs13265227			1,00E-012
NA12716	reg_DEFA	chr8	6893329	G	A	16	94,00%	15					rs13255432			1,00E-012
NA12716	reg_DEFA	chr8	6893366	A	G	20	100,00%	20					rs13258671			1,00E-012
NA12716	reg_DEFA	chr8	6893449	C	T	16	100,00%	16					rs12675019	rs11137087		1,00E-012
NA12716	reg_DEFA	chr8	6893450	A	G	16	100,00%	16					rs11137087			1,00E-012
NA12716	reg_DEFA	chr8	6893571	A	T	17	94,00%	16					rs12677144			1,00E-012
NA12716	reg_DEFA	chr8	6894199	A	G	13	92,00%	12					rs4841824			1,00E-012
NA12716	reg_DEFA	chr8	6894251	G	A	17	100,00%	17					rs4841825			1,00E-012
NA12716	reg_DEFA	chr8	6894335	C	T	23	96,00%	22					rs13267464			1,00E-012
NA12716	reg_DEFA	chr8	6894359	A	G	27	100,00%	27					rs4841827			1,00E-012
NA12716	reg_DEFA	chr8	6894414	T	C	28	93,00%	26					rs4841829			1,00E-012
NA12716	reg_DEFA	chr8	6894855	C	A	21	100,00%	21					rs10867024			1,00E-012
NA12716	reg_DEFA	chr8	6895055	T	G	30	100,00%	30					rs10780177			1,00E-012
NA12716	reg_DEFA	chr8	6895465	T	G	52	94,00%	49					rs10503360			1,00E-012
NA12716	reg_DEFA	chr8	6895593	G	A	48	96,00%	46					rs7007253			1,00E-012
NA12716	reg_DEFA	chr8	6896339	C	G	16	100,00%	16					rs4446760			1,00E-012
NA12716	reg_DEFA	chr8	6896851	C	T	69	93,00%	64					rs10867025			1,00E-012
NA12716	reg_DEFA	chr8	6897044	G	C	74	93,00%	69					rs11137088			1,00E-012
NA12716	reg_DEFA	chr8	6897177	C	G	76	95,00%	72					rs4451329			1,00E-012
NA12716	reg_DEFA	chr8	6897235	C	T	72	94,00%	68					rs4344104			1,00E-012
NA12716	reg_DEFA	chr8	6897291	T	C	75	95,00%	71					rs4392921			1,00E-012
NA12716	reg_DEFA	chr8	6897555	T	G	76	96,00%	73					rs13271389			1,00E-012
NA12716	reg_DEFA	chr8	6898076	G	A	84	94,00%	79					rs4841830			1,00E-012
NA12716	reg_DEFA	chr8	6898613	T	G	72	99,00%	71					rs12680521			1,00E-012
NA12716	reg_DEFA	chr8	6898728	C	T	56	98,00%	55					rs13273327			1,00E-012
NA12716	reg_DEFA	chr8	6898818	A	G	34	100,00%	34					rs12680095			1,00E-012
NA12716	reg_DEFA	chr8	6898847	C	T	30	97,00%	29					rs12678005			1,00E-012
NA12716	reg_DEFA	chr8	6899381	G	A	53	98,00%	52					rs13279849			1,00E-012
NA12716	reg_DEFA	chr8	6899982	G	A	54	96,00%	52					rs4645580			1,00E-012
NA12716	reg_DEFA	chr8	6900441	G	A	61	93,00%	57	T	T	-2	DEFA5	rs2272719			1,00E-012
NA12716	reg_DEFA	chr8	6901260	A	G	61	98,00%	60			-	DEFA5	rs10095331			1,00E-012
NA12716	reg_DEFA	chr8	6902056	T	C	52	92,00%	48					rs4395911			1,00E-012
NA12716	reg_DEFB	chr8	7226320	A	G	57	91,00%	52					rs2740644			1,00E-012
NA12716	reg_DEFB	chr8	7226650	T	G	47	98,00%	46							rs62636823	1,00E-012
NA12716	reg_DEFB	chr8	7227202	C	G	60	98,00%	59					rs4110303			1,00E-012
NA12716	reg_DEFB	chr8	7227284	A	C	46	98,00%	45							rs71511231	1,00E-012
NA12716	reg_DEFB	chr8	7227642	C	T	29	100,00%	29					rs3915363			1,00E-012
NA12716	reg_DEFB	chr8	7227922	A	T	19	95,00%	18					rs28413583	rs5004605		1,00E-012
NA12716	reg_DEFB	chr8	7230646	C	G	33	94,00%	31							rs4295681	1,00E-012
NA12716	reg_DEFB	chr8	7230752	G	A	30	83,00%	25							rs71511233	1,00E-012
NA12716	reg_DEFB	chr8	7230809	G	T	29	52,00%	15								1,00E-012
NA12716	reg_DEFB	chr8	7231435	G	A	30	100,00%	30							rs71511234	1,00E-012
NA12716	reg_DEFB	chr8	7231456	G	A	33	100,00%	33					rs2739878			1,00E-012
NA12716	reg_DEFB	chr8	7231549	C	G	36	97,00%	35					rs2719513			1,00E-012
NA12716	reg_DEFB	chr8	7232643	A	T	42	93,00%	39					rs3915354			1,00E-012
NA12716	reg_DEFB	chr8	7232788	A	G	38	92,00%	35					rs2698867			1,00E-012
NA12716	reg_DEFB	chr8	7233725	C	T	24	96,00%	23					rs2740632			1,00E-012
NA12716	reg_DEFB	chr8	7235443	G	C	88	95,00%	84					rs9720375			1,00E-012
NA12716	reg_DEFB	chr8	7235975	G	A	33	36,00%	12							rs71511240	1,03E-011
NA12716	reg_DEFB	chr8	7236869	C	T	35	43,00%	15								1,00E-012
NA12716	reg_DEFB	chr8	7238365	A	G	29	97,00%	28					rs9720329			1,00E-012
NA12716	reg_DEFB	chr8	7240395	C	A	56	96,00%	54					rs13260072			1,00E-012

NA12716	reg_DEFB	chr8	7240612	C	A	49	39,00%	19	rs2740609			1,00E-012
NA12716	reg_DEFB	chr8	7240705	T	C	45	44,00%	20	rs7005466			1,00E-012
NA12716	reg_DEFB	chr8	7240831	C	T	38	24,00%	9			rs71250725	1,60E-007
NA12716	reg_DEFB	chr8	7240856	C	A	39	28,00%	11	rs2740608			8,84E-010
NA12716	reg_DEFB	chr8	7241049	T	C	24	25,00%	6			rs2698963	1,39E-005
NA12716	reg_DEFB	chr8	7241078	T	C	23	30,00%	7			rs71250726	6,03E-007
NA12716	reg_DEFB	chr8	7241147	C	A	27	41,00%	11			rs2698964	1,60E-011
NA12716	reg_DEFB	chr8	7241218	T	C	30	50,00%	15	rs7009957			1,00E-012
NA12716	reg_DEFB	chr8	7241259	A	T	30	47,00%	14				1,00E-012
NA12716	reg_DEFB	chr8	7241264	C	T	31	52,00%	16	rs4625055			1,00E-012
NA12716	reg_DEFB	chr8	7241285	A	G	32	50,00%	16			rs3958824	1,00E-012
NA12716	reg_DEFB	chr8	7241358	T	C	31	45,00%	14			rs2740157	1,00E-012
NA12716	reg_DEFB	chr8	7241366	G	T	32	41,00%	13			rs2740606	1,00E-012
NA12716	reg_DEFB	chr8	7241686	A	G	34	97,00%	33	rs4571754			1,00E-012
NA12716	reg_DEFB	chr8	7241782	T	G	20	90,00%	18				1,00E-012
NA12716	reg_DEFB	chr8	7241787	C	T	21	38,00%	8			rs2740605	1,22E-008
NA12716	reg_DEFB	chr8	7242034	T	A	42	57,00%	24			rs71247376	1,00E-012
NA12716	reg_DEFB	chr8	7242037	G	C	42	57,00%	24			rs71247376	1,00E-012
NA12716	reg_DEFB	chr8	7242252	G	C	35	49,00%	17			rs3988892	1,00E-012
NA12716	reg_DEFB	chr8	7242513	T	C	41	46,00%	19			rs3988890	1,00E-012
NA12716	reg_DEFB	chr8	7242527	T	C	42	48,00%	20				1,00E-012
NA12716	reg_DEFB	chr8	7242637	T	G	42	55,00%	23			rs3927359	1,00E-012
NA12716	reg_DEFB	chr8	7242769	A	T	34	50,00%	17			rs72494256	1,00E-012
NA12716	reg_DEFB	chr8	7242795	G	C	35	100,00%	35			rs3928107	1,00E-012
NA12716	reg_DEFB	chr8	7242850	G	A	38	47,00%	18			rs2740749	1,00E-012
NA12716	reg_DEFB	chr8	7242925	C	T	37	54,00%	20	rs2698913			1,00E-012
NA12716	reg_DEFB	chr8	7243080	G	A	35	37,00%	13				1,00E-012
NA12716	reg_DEFB	chr8	7243097	A	G	39	36,00%	14				1,00E-012
NA12716	reg_DEFB	chr8	7243226	G	C	42	36,00%	15			rs56017112	1,00E-012
NA12716	reg_DEFB	chr8	7243230	C	T	41	37,00%	15			rs2719607	1,00E-012
NA12716	reg_DEFB	chr8	7243275	T	A	43	35,00%	15			rs2719604	1,00E-012
NA12716	reg_DEFB	chr8	7243307	T	C	40	40,00%	16			rs2719603	1,00E-012
NA12716	reg_DEFB	chr8	7243453	C	T	40	45,00%	18	rs4840743			1,00E-012
NA12716	reg_DEFB	chr8	7243457	A	T	40	92,00%	37	rs4840275			1,00E-012
NA12716	reg_DEFB	chr8	7243460	T	C	38	42,00%	16				1,00E-012
NA12716	reg_DEFB	chr8	7243517	G	A	33	45,00%	15			rs2463985	1,00E-012
NA12716	reg_DEFB	chr8	7243526	C	T	30	50,00%	15			rs2466114	1,00E-012
NA12716	reg_DEFB	chr8	7243649	A	G	30	37,00%	11	rs2463984			3,60E-011
NA12716	reg_DEFB	chr8	7243739	T	C	33	42,00%	14				1,00E-012
NA12716	reg_DEFB	chr8	7243778	T	C	35	54,00%	19	rs2737539			1,00E-012
NA12716	reg_DEFB	chr8	7243809	T	C	37	43,00%	16	rs2977410			1,00E-012
NA12716	reg_DEFB	chr8	7243954	C	A	37	41,00%	15	rs2740150	rs34384791		1,00E-012
NA12716	reg_DEFB	chr8	7243999	A	G	36	36,00%	13	rs2740149	rs35454996		1,00E-012
NA12716	reg_DEFB	chr8	7244256	G	C	16	44,00%	7	rs2954087			3,25E-008
NA12716	reg_DEFB	chr8	7244340	C	T	17	47,00%	8	rs2977415			1,60E-009
NA12716	reg_DEFB	chr8	7244846	T	C	30	100,00%	30	rs34757760			1,00E-012
NA12716	reg_DEFB	chr8	7244847	G	A	31	100,00%	31	rs34757760			1,00E-012
NA12716	reg_DEFB	chr8	7244856	T	G	32	97,00%	31	rs35642932			1,00E-012
NA12716	reg_DEFB	chr8	7245066	G	A	29	41,00%	12	rs2977405			1,00E-012
NA12716	reg_DEFB	chr8	7245210	G	A	39	38,00%	15	rs2977408	rs2977409		1,00E-012
NA12716	reg_DEFB	chr8	7245290	C	T	38	42,00%	16	rs35021319			1,00E-012
NA12716	reg_DEFB	chr8	7245577	T	C	17	47,00%	8	rs2740148	rs4840745		1,60E-009
NA12716	reg_DEFB	chr8	7245649	G	A	16	56,00%	9	rs2740146	rs4840746		2,54E-011

NA12716	reg_DEFB	chr8	7245898	G	C	24	92,00%	22	rs2719573			1,00E-012
NA12716	reg_DEFB	chr8	7246164	C	T	46	46,00%	21	rs2719567			1,00E-012
NA12716	reg_DEFB	chr8	7246774	G	A	27	52,00%	14	rs2977411			1,00E-012
NA12716	reg_DEFB	chr8	7247056	G	A	44	48,00%	21	rs2954062			1,00E-012
NA12716	reg_DEFB	chr8	7247198	C	A	36	39,00%	14	rs2954061			1,00E-012
NA12716	reg_DEFB	chr8	7247379	C	T	17	71,00%	12	rs2740143	rs34835930		1,00E-012
NA12716	reg_DEFB	chr8	7247690	T	C	16	31,00%	5				2,27E-005
NA12716	reg_DEFB	chr8	7247728	A	C	20	25,00%	5				7,47E-005
NA12716	reg_DEFB	chr8	7247737	T	C	19	42,00%	8				4,72E-009
NA12716	reg_DEFB	chr8	7247775	A	C	16	50,00%	8				8,62E-010
NA12716	reg_DEFB	chr8	7247847	G	C	14	64,00%	9				4,45E-012
NA12716	reg_DEFB	chr8	7247936	A	G	9	56,00%	5				7,51E-007
NA12716	reg_DEFB	chr8	7247959	T	A	8	62,00%	5	rs2266498			3,40E-007
NA12716	reg_DEFB	chr8	7247963	A	G	8	37,00%	3				6,25E-004
NA12716	reg_DEFB	chr8	7248073	T	A	6	50,00%	3				2,31E-004
NA12716	reg_DEFB	chr8	7249027	A	C	3	100,00%	3				1,22E-005
NA12716	reg_DEFB	chr8	7249069	C	G	3	100,00%	3				1,22E-005
NA12716	reg_DEFB	chr8	7249100	T	C	3	100,00%	3	rs2740138			1,22E-005
NA12716	reg_DEFB	chr8	7249269	T	C	4	75,00%	3			rs62494626	4,78E-005
NA12716	reg_DEFB	chr8	7249925	G	A	5	60,00%	3			rs62510592	1,18E-004
NA12716	reg_DEFB	chr8	7250102	G	A	7	86,00%	6			rs62636833	1,02E-009
NA12716	reg_DEFB	chr8	7250132	C	T	8	62,00%	5			rs4108895	3,40E-007
NA12716	reg_DEFB	chr8	7250139	A	C	8	87,00%	7				3,21E-011
NA12716	reg_DEFB	chr8	7250185	A	G	9	33,00%	3				9,21E-004
NA12716	reg_DEFB	chr8	7250219	T	C	10	90,00%	9				1,00E-012
NA12716	reg_DEFB	chr8	7250289	T	C	14	57,00%	8			rs35064420	2,09E-010
NA12716	reg_DEFB	chr8	7250365	G	A	12	42,00%	5			rs9802024	4,45E-006
NA12716	reg_DEFB	chr8	7250371	T	C	12	58,00%	7			rs71521097	2,45E-009
NA12716	reg_DEFB	chr8	7250404	T	C	10	90,00%	9				1,00E-012
NA12716	reg_DEFB	chr8	7250428	T	G	10	80,00%	8			rs28883954	4,25E-012
NA12716	reg_DEFB	chr8	7250500	T	G	4	100,00%	4				2,80E-007
NA12716	reg_DEFB	chr8	7252395	G	C	4	75,00%	3				4,78E-005
NA12716	reg_DEFB	chr8	7252477	T	C	5	80,00%	4	rs2740113			1,37E-006
NA12716	reg_DEFB	chr8	7252507	A	G	6	83,00%	5				3,79E-008
NA12716	reg_DEFB	chr8	7252518	A	G	6	83,00%	5				3,79E-008
NA12716	reg_DEFB	chr8	7252595	A	G	6	67,00%	4			rs4876565	4,04E-006
NA12716	reg_DEFB	chr8	7252655	A	G	3	100,00%	3				1,22E-005
NA12716	reg_DEFB	chr8	7253101	G	A	16	44,00%	7	rs2698826	rs3988849		3,25E-008
NA12716	reg_DEFB	chr8	7253210	C	T	18	39,00%	7	rs3988850			8,67E-008
NA12716	reg_DEFB	chr8	7253275	G	A	20	35,00%	7			rs2719571	2,03E-007
NA12716	reg_DEFB	chr8	7253389	T	C	27	52,00%	14	rs2719572			1,00E-012
NA12716	reg_DEFB	chr8	7254208	T	C	38	39,00%	15				1,00E-012
NA12716	reg_DEFB	chr8	7254247	A	G	35	80,00%	28	rs3915368			1,00E-012
NA12716	reg_DEFB	chr8	7254807	C	G	71	52,00%	37	rs2740741			1,00E-012
NA12716	reg_DEFB	chr8	7255839	C	T	64	94,00%	60	rs3877964			1,00E-012
NA12716	reg_DEFB	chr8	7256331	C	G	49	51,00%	25	rs2719545			1,00E-012
NA12716	reg_DEFB	chr8	7256550	G	A	44	45,00%	20			rs3915496	1,00E-012
NA12716	reg_DEFB	chr8	7256617	G	A	44	98,00%	43	rs28576922	rs3866483		1,00E-012
NA12716	reg_DEFB	chr8	7256739	C	T	30	33,00%	10			rs7813724	8,17E-010
NA12716	reg_DEFB	chr8	7256872	G	A	8	37,00%	3			rs71513067	6,25E-004
NA12716	reg_DEFB	chr8	7257104	T	C	31	45,00%	14	rs2740095			1,00E-012
NA12716	reg_DEFB	chr8	7257126	G	A	34	100,00%	34	rs4840277			1,00E-012
NA12716	reg_DEFB	chr8	7257138	G	A	34	50,00%	17	rs7386020			1,00E-012

NA12716	reg_DEFB	chr8	7257305	C	A	49	51,00%	25		rs71247231	1,00E-012
NA12716	reg_DEFB	chr8	7258456	G	T	78	22,00%	17	rs2737914		7,16E-012
NA12716	reg_DEFB	chr8	7258464	G	A	76	32,00%	24		rs72626630	1,00E-012
NA12716	reg_DEFB	chr8	7259539	G	A	67	40,00%	27	rs41390446		1,00E-012
NA12716	reg_DEFB	chr8	7259563	G	T	65	38,00%	25	rs41507446		1,00E-012
NA12716	reg_DEFB	chr8	7259739	C	T	80	49,00%	39	rs2737531		1,00E-012
NA12716	reg_DEFB	chr8	7260004	A	G	72	42,00%	30	rs2740090		1,00E-012
NA12716	reg_DEFB	chr8	7260322	T	C	35	57,00%	20		rs71251804	1,00E-012
NA12716	reg_DEFB	chr8	7260751	C	T	6	67,00%	4			4,04E-006
NA12716	reg_DEFB	chr8	7261175	C	T	54	44,00%	24			1,00E-012
NA12716	reg_DEFB	chr8	7261330	G	A	48	37,00%	18			1,00E-012
NA12716	reg_DEFB	chr8	7261371	G	T	46	39,00%	18			1,00E-012
NA12716	reg_DEFB	chr8	7261718	A	G	50	100,00%	50		rs71509106	1,00E-012
NA12716	reg_DEFB	chr8	7261867	A	G	65	43,00%	28	rs2740086		1,00E-012
NA12716	reg_DEFB	chr8	7262672	G	A	42	48,00%	20		rs71513122	1,00E-012
NA12716	reg_DEFB	chr8	7262787	C	T	38	47,00%	18	rs3762040		1,00E-012
NA12716	reg_DEFB	chr8	7263102	T	C	46	98,00%	45	rs4840278		1,00E-012
NA12716	reg_DEFB	chr8	7263191	G	C	33	52,00%	17	rs3762052		1,00E-012
NA12716	reg_DEFB	chr8	7263349	G	A	24	58,00%	14	rs3762051		1,00E-012
NA12716	reg_DEFB	chr8	7263407	A	G	30	50,00%	15		rs71244071	1,00E-012
NA12716	reg_DEFB	chr8	7263531	T	C	38	53,00%	20			1,00E-012
NA12716	reg_DEFB	chr8	7263605	A	G	34	41,00%	14		rs56020554	1,00E-012
NA12716	reg_DEFB	chr8	7263647	A	G	30	37,00%	11			3,60E-011
NA12716	reg_DEFB	chr8	7263683	T	C	32	37,00%	12			6,54E-012
NA12716	reg_DEFB	chr8	7263744	G	A	35	37,00%	13	rs2740083		1,00E-012
NA12716	reg_DEFB	chr8	7263764	G	C	32	34,00%	11	rs2409862		8,20E-011
NA12716	reg_DEFB	chr8	7263877	A	C	27	100,00%	27	rs6651513		1,00E-012
NA12716	reg_DEFB	chr8	7263885	C	T	29	31,00%	9	rs3762038		1,19E-008
NA12716	reg_DEFB	chr8	7263922	G	T	24	21,00%	5	rs3988843		1,90E-004
NA12716	reg_DEFB	chr8	7264091	G	A	30	43,00%	13	rs3762037		1,00E-012
NA12716	reg_DEFB	chr8	7264095	A	G	30	40,00%	12	rs3988844		2,51E-012
NA12716	reg_DEFB	chr8	7264156	G	C	36	81,00%	29	rs2737536		1,00E-012
NA12716	reg_DEFB	chr8	7264462	A	C	52	38,00%	20		rs71254898	1,00E-012
NA12716	reg_DEFB	chr8	7264552	A	C	46	39,00%	18	rs2737538		1,00E-012
NA12716	reg_DEFB	chr8	7264604	G	A	45	42,00%	19		rs71513120	1,00E-012
NA12716	reg_DEFB	chr8	7264753	G	C	35	40,00%	14		rs71537819	1,00E-012
NA12716	reg_DEFB	chr8	7264799	C	T	37	43,00%	16	rs3866482		1,00E-012
NA12716	reg_DEFB	chr8	7264836	T	G	36	39,00%	14	rs4118281		1,00E-012
NA12716	reg_DEFB	chr8	7264944	G	C	35	94,00%	33	rs4840753		1,00E-012
NA12716	reg_DEFB	chr8	7264948	G	A	35	37,00%	13	rs3866481		1,00E-012
NA12716	reg_DEFB	chr8	7265160	T	C	29	45,00%	13		rs71513119	1,00E-012
NA12716	reg_DEFB	chr8	7265202	A	T	35	43,00%	15		rs71513118	1,00E-012
NA12716	reg_DEFB	chr8	7265254	T	C	33	39,00%	13		rs71513117	1,00E-012
NA12716	reg_DEFB	chr8	7265340	T	C	36	56,00%	20	rs2698832		1,00E-012
NA12716	reg_DEFB	chr8	7265354	C	T	37	30,00%	11			4,70E-010
NA12716	reg_DEFB	chr8	7265410	G	A	41	22,00%	9		rs71513116	3,24E-007
NA12716	reg_DEFB	chr8	7265947	G	T	42	33,00%	14	rs3988845		1,00E-012
NA12716	reg_DEFB	chr8	7265983	G	A	53	55,00%	29	rs3866480		1,00E-012
NA12716	reg_DEFB	chr8	7266035	T	C	54	43,00%	23	rs3988846		1,00E-012
NA12716	reg_DEFB	chr8	7266053	C	T	55	49,00%	27	rs2740731		1,00E-012
NA12716	reg_DEFB	chr8	7266297	C	A	55	45,00%	25	rs3988829		1,00E-012
NA12716	reg_DEFB	chr8	7266317	C	T	55	47,00%	26	rs3988830		1,00E-012
NA12716	reg_DEFB	chr8	7266358	C	T	45	44,00%	20	rs3988831		1,00E-012

NA12716	reg_DEFB	chr8	7266438	A	C	40	42,00%	17			rs3988832			1,00E-012
NA12716	reg_DEFB	chr8	7266786	T	C	49	41,00%	20					rs71513115	1,00E-012
NA12716	reg_DEFB	chr8	7266858	C	T	51	47,00%	24					rs71513114	1,00E-012
NA12716	reg_DEFB	chr8	7267031	T	G	65	100,00%	65			rs7815816			1,00E-012
NA12716	reg_DEFB	chr8	7267055	A	C	63	38,00%	24					rs71513113	1,00E-012
NA12716	reg_DEFB	chr8	7267305	C	G	46	39,00%	18			rs2737543			1,00E-012
NA12716	reg_DEFB	chr8	7267395	G	T	33	27,00%	9					rs71513110	4,21E-008
NA12716	reg_DEFB	chr8	7267436	A	C	28	43,00%	12					rs71513109	1,00E-012
NA12716	reg_DEFB	chr8	7267461	T	G	28	46,00%	13					rs71513108	1,00E-012
NA12716	reg_DEFB	chr8	7267609	T	G	52	50,00%	26					rs71513107	1,00E-012
NA12716	reg_DEFB	chr8	7267636	C	G	55	49,00%	27					rs71513106	1,00E-012
NA12716	reg_DEFB	chr8	7267855	A	G	59	98,00%	58			rs3958991			1,00E-012
NA12716	reg_DEFB	chr8	7268025	T	A	59	46,00%	27			rs28610011	rs3866479		1,00E-012
NA12716	reg_DEFB	chr8	7268334	A	G	53	38,00%	20						1,00E-012
NA12716	reg_DEFB	chr8	7268520	A	T	48	50,00%	24					rs71513105	1,00E-012
NA12716	reg_DEFB	chr8	7268623	G	T	49	98,00%	48			rs4840754			1,00E-012
NA12716	reg_DEFB	chr8	7268975	C	T	40	50,00%	20			rs3958992			1,00E-012
NA12716	reg_DEFB	chr8	7269055	T	C	33	100,00%	33						1,00E-012
NA12716	reg_DEFB	chr8	7269085	G	T	37	97,00%	36						1,00E-012
NA12716	reg_DEFB	chr8	7269368	T	C	35	97,00%	34					rs71511302	1,00E-012
NA12716	reg_DEFB	chr8	7269455	T	C	31	42,00%	13					rs2698835	1,00E-012
NA12716	reg_DEFB	chr8	7269776	G	A	34	94,00%	32					rs2737547	1,00E-012
NA12716	reg_DEFB	chr8	7269817	C	A	36	100,00%	36					rs62636842	1,00E-012
NA12716	reg_DEFB	chr8	7269942	G	T	32	97,00%	31						1,00E-012
NA12716	reg_DEFB	chr8	7269967	T	C	34	94,00%	32						1,00E-012
NA12716	reg_DEFB	chr8	7270168	G	T	32	94,00%	30						1,00E-012
NA12716	reg_DEFB	chr8	7270508	C	T	38	63,00%	24					rs71511299	1,00E-012
NA12716	reg_DEFB	chr8	7270703	T	C	33	45,00%	15					rs71511298	1,00E-012
NA12716	reg_DEFB	chr8	7270806	C	T	23	52,00%	12			rs2740728			1,00E-012
NA12716	reg_DEFB	chr8	7270994	G	A	16	50,00%	8			rs2737548			8,62E-010
NA12716	reg_DEFB	chr8	7271226	C	A	18	22,00%	4						6,61E-004
NA12716	reg_DEFB	chr8	7271232	T	C	18	22,00%	4						6,61E-004
NA12716	reg_DEFB	chr8	7271434	T	C	18	100,00%	18					rs4543549	1,00E-012
NA12716	reg_DEFB	chr8	7271837	C	T	45	58,00%	26					rs62636845	1,00E-012
NA12716	reg_DEFB	chr8	7271938	C	T	62	55,00%	34					rs62636846	1,00E-012
NA12716	reg_DEFB	chr8	7272893	G	A	80	47,00%	38			rs3988902			1,00E-012
NA12716	reg_DEFB	chr8	7272905	G	C	80	99,00%	79					rs71509107	1,00E-012
NA12716	reg_DEFB	chr8	7272963	A	G	76	41,00%	31					rs71511249	1,00E-012
NA12716	reg_DEFB	chr8	7273532	G	C	53	58,00%	31			rs3866478			1,00E-012
NA12716	reg_DEFB	chr8	7273819	A	G	34	41,00%	14						1,00E-012
NA12716	reg_DEFB	chr8	7274151	C	T	66	64,00%	42	-	DEFB103B			rs62636848	1,00E-012
NA12716	reg_DEFB	chr8	7274576	T	C	26	42,00%	11	-	DEFB103B	rs3789865			9,51E-012
NA12716	reg_DEFB	chr8	7274717	T	C	18	50,00%	9	-	DEFB103B	rs3789864			7,33E-011
NA12716	reg_DEFB	chr8	7274787	G	C	20	40,00%	8	-	DEFB103B	rs3789862			7,71E-009
NA12716	reg_DEFB	chr8	7275507	G	T	38	39,00%	15					rs71511250	1,00E-012
NA12716	reg_DEFB	chr8	7275590	A	G	25	48,00%	12			rs4461923			1,00E-012
NA12716	reg_DEFB	chr8	7275765	T	C	15	33,00%	5						1,59E-005
NA12716	reg_DEFB	chr8	7275809	A	G	21	62,00%	13					rs71511253	1,00E-012
NA12716	reg_DEFB	chr8	7275956	G	A	33	55,00%	18						1,00E-012
NA12716	reg_DEFB	chr8	7276674	A	C	77	93,00%	72			rs2737554			1,00E-012
NA12716	reg_DEFB	chr8	7277359	T	C	58	100,00%	58					rs71509108	1,00E-012
NA12716	reg_DEFB	chr8	7277387	T	C	60	45,00%	27					rs71511255	1,00E-012
NA12716	reg_DEFB	chr8	7277422	T	A	62	100,00%	62					rs71509109	1,00E-012

NA12716	reg_DEFB	chr8	7277424	C	A	62	94,00%	58		rs71509110	1,00E-012
NA12716	reg_DEFB	chr8	7277451	C	G	64	44,00%	28		rs71511256	1,00E-012
NA12716	reg_DEFB	chr8	7277460	G	A	63	43,00%	27		rs71511257	1,00E-012
NA12716	reg_DEFB	chr8	7277507	T	C	68	59,00%	40			1,00E-012
NA12716	reg_DEFB	chr8	7277521	C	G	68	37,00%	25		rs71511259	1,00E-012
NA12716	reg_DEFB	chr8	7277545	C	T	68	34,00%	23		rs71511260	1,00E-012
NA12716	reg_DEFB	chr8	7277566	G	A	62	35,00%	22		rs71509111	1,00E-012
NA12716	reg_DEFB	chr8	7277662	A	G	60	32,00%	19		rs4999975	1,00E-012
NA12716	reg_DEFB	chr8	7277710	T	C	55	27,00%	15		rs4999974	3,47E-012
NA12716	reg_DEFB	chr8	7277744	A	C	57	30,00%	17		rs71252681	1,00E-012
NA12716	reg_DEFB	chr8	7277755	T	C	56	34,00%	19	rs4999973		1,00E-012
NA12716	reg_DEFB	chr8	7277810	A	G	59	34,00%	20			1,00E-012
NA12716	reg_DEFB	chr8	7277830	C	G	54	26,00%	14			1,74E-011
NA12716	reg_DEFB	chr8	7278010	C	T	84	98,00%	82			1,00E-012
NA12716	reg_DEFB	chr8	7278116	C	T	97	51,00%	49		rs4840303	1,00E-012
NA12716	reg_DEFB	chr8	7278542	T	A	81	100,00%	81	rs41380147		1,00E-012
NA12716	reg_DEFB	chr8	7278548	A	C	81	48,00%	39		rs4840304	1,00E-012
NA12716	reg_DEFB	chr8	7278563	G	T	85	46,00%	39		rs9693075	1,00E-012
NA12716	reg_DEFB	chr8	7278594	T	C	87	97,00%	84			1,00E-012
NA12716	reg_DEFB	chr8	7278727	C	T	91	42,00%	38		rs71276791	1,00E-012
NA12716	reg_DEFB	chr8	7279289	T	C	56	43,00%	24			1,00E-012
NA12716	reg_DEFB	chr8	7279315	T	C	44	98,00%	43			1,00E-012
NA12716	reg_DEFB	chr8	7279450	C	T	37	41,00%	15	rs4840306		1,00E-012
NA12716	reg_DEFB	chr8	7279696	A	T	63	49,00%	31	rs2737899		1,00E-012
NA12716	reg_DEFB	chr8	7279780	C	G	64	53,00%	34		rs71276797	1,00E-012
NA12716	reg_DEFB	chr8	7279877	C	T	61	57,00%	35		rs71276798	1,00E-012
NA12716	reg_DEFB	chr8	7280090	T	C	48	94,00%	45			1,00E-012
NA12716	reg_DEFB	chr8	7280221	C	T	64	48,00%	31		rs71299143	1,00E-012
NA12716	reg_DEFB	chr8	7280224	G	C	64	44,00%	28			1,00E-012
NA12716	reg_DEFB	chr8	7280227	A	T	64	95,00%	61			1,00E-012
NA12716	reg_DEFB	chr8	7280259	T	G	60	47,00%	28	rs2698836		1,00E-012
NA12716	reg_DEFB	chr8	7280364	G	C	63	98,00%	62		rs2737897	1,00E-012
NA12716	reg_DEFB	chr8	7280790	G	A	51	43,00%	22	rs2737896		1,00E-012
NA12716	reg_DEFB	chr8	7281118	T	C	49	57,00%	28	rs2737895		1,00E-012
NA12716	reg_DEFB	chr8	7281292	C	T	37	100,00%	37	rs4840279		1,00E-012
NA12716	reg_DEFB	chr8	7281372	T	G	35	37,00%	13	rs4247403		1,00E-012
NA12716	reg_DEFB	chr8	7281483	A	T	32	28,00%	9	rs2740074		3,13E-008
NA12716	reg_DEFB	chr8	7281562	C	T	29	52,00%	15		rs2737894	1,00E-012
NA12716	reg_DEFB	chr8	7281568	A	T	29	41,00%	12	rs2740073		1,00E-012
NA12716	reg_DEFB	chr8	7281649	T	A	26	46,00%	12		rs71509112	1,00E-012
NA12716	reg_DEFB	chr8	7281710	G	T	28	46,00%	13	rs2698838		1,00E-012
NA12716	reg_DEFB	chr8	7282103	G	T	37	38,00%	14		rs71267734	1,00E-012
NA12716	reg_DEFB	chr8	7282443	T	C	48	96,00%	46			1,00E-012
NA12716	reg_DEFB	chr8	7282560	C	T	60	35,00%	21		rs71267735	1,00E-012
NA12716	reg_DEFB	chr8	7282592	G	A	63	40,00%	25		rs71267736	1,00E-012
NA12716	reg_DEFB	chr8	7282670	T	C	73	97,00%	71			1,00E-012
NA12716	reg_DEFB	chr8	7282719	A	G	81	51,00%	41		rs2737556	1,00E-012
NA12716	reg_DEFB	chr8	7282985	T	A	81	48,00%	39		rs2737892	1,00E-012
NA12716	reg_DEFB	chr8	7283081	C	T	61	51,00%	31	rs3915374		1,00E-012
NA12716	reg_DEFB	chr8	7283283	C	T	45	51,00%	23		rs71267737	1,00E-012
NA12716	reg_DEFB	chr8	7283305	A	G	38	97,00%	37			1,00E-012
NA12716	reg_DEFB	chr8	7283441	C	G	31	48,00%	15		rs71267738	1,00E-012
NA12716	reg_DEFB	chr8	7283465	C	T	30	47,00%	14		rs71267739	1,00E-012

NA12716	reg_DEFB	chr8	7283568	A	G	25	36,00%	9					rs7836098	2,64E-009
NA12716	reg_DEFB	chr8	7283609	G	C	31	32,00%	10					rs71267741	1,18E-009
NA12716	reg_DEFB	chr8	7283828	C	G	72	58,00%	42			rs3915371			1,00E-012
NA12716	reg_DEFB	chr8	7283981	G	A	81	93,00%	75					rs3915370	1,00E-012
NA12716	reg_DEFB	chr8	7284078	A	G	84	45,00%	38						1,00E-012
NA12716	reg_DEFB	chr8	7284088	T	C	83	45,00%	37						1,00E-012
NA12716	reg_DEFB	chr8	7284285	G	A	38	45,00%	17			rs2698842			1,00E-012
NA12716	reg_DEFB	chr8	7284296	G	T	35	51,00%	18						1,00E-012
NA12716	reg_DEFB	chr8	7284304	G	C	33	52,00%	17						1,00E-012
NA12716	reg_DEFB	chr8	7284416	A	G	22	32,00%	7					rs71267742	4,28E-007
NA12716	reg_DEFB	chr8	7284587	C	T	16	44,00%	7					rs71267744	3,25E-008
NA12716	reg_DEFB	chr8	7284697	G	C	21	52,00%	11					rs71267745	1,00E-012
NA12716	reg_DEFB	chr8	7284872	T	A	34	53,00%	18					rs71267746	1,00E-012
NA12716	reg_DEFB	chr8	7284977	A	G	37	54,00%	20					rs71267747	1,00E-012
NA12716	reg_DEFB	chr8	7285008	T	A	35	49,00%	17					rs71267748	1,00E-012
NA12716	reg_DEFB	chr8	7285027	C	T	35	34,00%	12			rs2740069			2,42E-011
NA12716	reg_DEFB	chr8	7285028	A	G	35	40,00%	14			rs2740069			1,00E-012
NA12716	reg_DEFB	chr8	7285109	C	T	34	47,00%	16					rs71267749	1,00E-012
NA12716	reg_DEFB	chr8	7285531	G	C	58	43,00%	25			rs2740068	rs2737887		1,00E-012
NA12716	reg_DEFB	chr8	7285682	T	C	52	42,00%	22					rs2698845	1,00E-012
NA12716	reg_DEFB	chr8	7285744	C	T	47	94,00%	44			rs3988838			1,00E-012
NA12716	reg_DEFB	chr8	7285834	T	A	43	86,00%	37			rs4501606			1,00E-012
NA12716	reg_DEFB	chr8	7285981	C	G	42	93,00%	39					rs3988839	1,00E-012
NA12716	reg_DEFB	chr8	7286036	C	T	47	45,00%	21					rs71267752	1,00E-012
NA12716	reg_DEFB	chr8	7286545	G	A	80	95,00%	76			rs2280948			1,00E-012
NA12716	reg_DEFB	chr8	7286699	A	T	88	50,00%	44			rs2280947			1,00E-012
NA12716	reg_DEFB	chr8	7286812	G	A	98	47,00%	46			rs2280946			1,00E-012
NA12716	reg_DEFB	chr8	7286995	G	C	66	94,00%	62			rs2280945			1,00E-012
NA12716	reg_DEFB	chr8	7287010	G	A	65	58,00%	38			rs2280944			1,00E-012
NA12716	reg_DEFB	chr8	7287700	A	G	37	51,00%	19			rs2740064			1,00E-012
NA12716	reg_DEFB	chr8	7287713	T	C	38	97,00%	37						1,00E-012
NA12716	reg_DEFB	chr8	7287772	G	A	34	59,00%	20					rs71267754	1,00E-012
NA12716	reg_DEFB	chr8	7287838	G	T	38	29,00%	11					rs71267755	6,48E-010
NA12716	reg_DEFB	chr8	7288049	G	A	27	59,00%	16			rs2740063			1,00E-012
NA12716	reg_DEFB	chr8	7288723	A	G	62	37,00%	23			rs2740060			1,00E-012
NA12716	reg_DEFB	chr8	7288985	T	C	53	94,00%	50			rs4466423			1,00E-012
NA12716	reg_DEFB	chr8	7289545	G	A	75	47,00%	35			rs2740059			1,00E-012
NA12716	reg_DEFB	chr8	7289788	C	T	49	45,00%	22			rs2698849			1,00E-012
NA12716	reg_DEFB	chr8	7289903	T	G	47	45,00%	21					rs71267756	1,00E-012
NA12716	reg_DEFB	chr8	7290162	T	G	23	70,00%	16			rs2698850			1,00E-012
NA12716	reg_DEFB	chr8	7290359	A	G	35	34,00%	12					rs71267757	2,42E-011
NA12716	reg_DEFB	chr8	7290646	G	A	38	55,00%	21			rs2740053			1,00E-012
NA12716	reg_DEFB	chr8	7291101	G	A	57	53,00%	30					rs71267758	1,00E-012
NA12716	reg_DEFB	chr8	7291811	C	T	55	51,00%	28					rs71267759	1,00E-012
NA12716	reg_DEFB	chr8	7292605	G	C	36	47,00%	17					rs71267763	1,00E-012
NA12716	reg_DEFB	chr8	7292773	T	C	43	56,00%	24	-	SPAG11B			rs4626629	1,00E-012
NA12716	reg_DEFB	chr8	7292820	C	G	40	60,00%	24	-	SPAG11B			rs2740047	1,00E-012
NA12716	reg_DEFB	chr8	7292896	A	C	36	58,00%	21	-	SPAG11B	rs3901154			1,00E-012
NA12716	reg_DEFB	chr8	7293741	A	G	9	67,00%	6	-	SPAG11B			rs2740041	1,17E-008
NA12716	reg_DEFB	chr8	7293835	G	A	13	54,00%	7	-	SPAG11B	rs2740040			5,17E-009
NA12716	reg_DEFB	chr8	7294502	C	G	43	56,00%	24	-	SPAG11B	rs2853664			1,00E-012
NA12716	reg_DEFB	chr8	7294800	C	T	69	59,00%	41	-	SPAG11B	rs2853663			1,00E-012
NA12716	reg_DEFB	chr8	7295003	A	G	66	50,00%	33	-	SPAG11B	rs2853661			1,00E-012

NA12716	reg_DEFB	chr8	7295159	G	C	58	45,00%	26			-	SPAG11B		rs3915372	1,00E-012
NA12716	reg_DEFB	chr8	7295311	T	A	57	42,00%	24			-	SPAG11B	rs2853660		1,00E-012
NA12716	reg_DEFB	chr8	7295321	A	T	61	48,00%	29			-	SPAG11B	rs2737558		1,00E-012
NA12716	reg_DEFB	chr8	7295796	T	C	74	97,00%	72	H	R	-2	SPAG11B	rs1042797		1,00E-012
NA12716	reg_DEFB	chr8	7295813	A	C	68	49,00%	33	I	M	-2	SPAG11B	rs12063		1,00E-012
NA12716	reg_DEFB	chr8	7295820	G	A	68	49,00%	33	P	L	-2	SPAG11B	rs2256100		1,00E-012
NA12716	reg_DEFB	chr8	7295830	C	T	69	43,00%	30	D	N	-2	SPAG11B			1,00E-012
NA12716	reg_DEFB	chr8	7295894	C	A	72	40,00%	29			-	SPAG11B	rs2853659		1,00E-012
NA12716	reg_DEFB	chr8	7295903	G	A	72	40,00%	29			-	SPAG11B	rs2738036		1,00E-012
NA12716	reg_DEFB	chr8	7295939	C	T	67	30,00%	20			-	SPAG11B		rs2737559	1,00E-012
NA12716	reg_DEFB	chr8	7296080	T	C	62	35,00%	22	D	G	-2	SPAG11B	rs2738035		1,00E-012
NA12716	reg_DEFB	chr8	7296085	A	G	61	38,00%	23	C	C	-2	SPAG11B		rs61749560	1,00E-012
NA12716	reg_DEFB	chr8	7296092	C	T	61	38,00%	23	R	K	-2	SPAG11B		rs61749561	1,00E-012
NA12716	reg_DEFB	chr8	7296116	C	T	61	36,00%	22	R	Q	-2	SPAG11B	rs2853658		1,00E-012
NA12716	reg_DEFB	chr8	7296208	A	G	59	100,00%	59			-	SPAG11B	rs4840280		1,00E-012
NA12716	reg_DEFB	chr8	7297645	A	T	80	51,00%	41			-	SPAG11B		rs71511266	1,00E-012
NA12716	reg_DEFB	chr8	7298128	T	C	51	39,00%	20			-	SPAG11B		rs71511267	1,00E-012
NA12716	reg_DEFB	chr8	7298525	T	G	54	96,00%	52			-	SPAG11B		rs62636856	1,00E-012
NA12716	reg_DEFB	chr8	7298616	A	G	44	50,00%	22			-	SPAG11B	rs2738028		1,00E-012
NA12716	reg_DEFB	chr8	7299529	T	C	64	59,00%	38			-	SPAG11B	rs2738025		1,00E-012
NA12716	reg_DEFB	chr8	7299852	A	T	48	46,00%	22			-	SPAG11B		rs71235967	1,00E-012
NA12716	reg_DEFB	chr8	7301157	C	G	49	29,00%	14			-	SPAG11B		rs71235965	9,40E-012
NA12716	reg_DEFB	chr8	7301270	A	G	48	44,00%	21			-	SPAG11B		rs71235963	1,00E-012
NA12716	reg_DEFB	chr8	7301857	A	G	16	25,00%	4			-	SPAG11B	rs2740031		4,08E-004
NA12716	reg_DEFB	chr8	7301986	G	A	16	50,00%	8			-	SPAG11B	rs2738017		8,62E-010
NA12716	reg_DEFB	chr8	7302658	T	C	21	57,00%	12			-	SPAG11B	rs2740717		1,00E-012
NA12716	reg_DEFB	chr8	7302765	C	T	26	92,00%	24			-	SPAG11B	rs4840756		1,00E-012
NA12716	reg_DEFB	chr8	7303683	A	T	39	46,00%	18			-	SPAG11B	rs4532613		1,00E-012
NA12716	reg_DEFB	chr8	7303698	G	A	38	55,00%	21			-	SPAG11B		rs71235959	1,00E-012
NA12716	reg_DEFB	chr8	7303880	C	T	21	100,00%	21			-	SPAG11B		rs62641376	1,00E-012
NA12716	reg_DEFB	chr8	7303899	C	G	17	100,00%	17			-	SPAG11B		rs71526141	1,00E-012
NA12716	reg_DEFB	chr8	7303958	T	C	16	100,00%	16			-	SPAG11B		rs2740712	1,00E-012
NA12716	reg_DEFB	chr8	7303987	G	A	21	100,00%	21			-	SPAG11B		rs34315736	1,00E-012
NA12716	reg_DEFB	chr8	7303999	A	G	23	100,00%	23			-	SPAG11B		rs7464358	1,00E-012
NA12716	reg_DEFB	chr8	7304356	C	G	68	44,00%	30			-	SPAG11B		rs71242685	1,00E-012
NA12716	reg_DEFB	chr8	7304399	A	C	66	55,00%	36			-	SPAG11B		rs2853665	1,00E-012
NA12716	reg_DEFB	chr8	7304490	A	G	63	59,00%	37			-	SPAG11B		rs2737566	1,00E-012
NA12716	reg_DEFB	chr8	7304912	A	G	44	98,00%	43			-	SPAG11B	rs4840757		1,00E-012
NA12716	reg_DEFB	chr8	7306469	G	T	68	96,00%	65			-	SPAG11B	rs4840282		1,00E-012
NA12716	reg_DEFB	chr8	7306933	C	A	62	45,00%	28			-	SPAG11B			1,00E-012
NA12716	reg_DEFB	chr8	7307198	T	C	38	100,00%	38			-	SPAG11B		rs62636859	1,00E-012
NA12716	reg_DEFB	chr8	7307400	C	G	38	47,00%	18			-	SPAG11B	rs2740708		1,00E-012
NA12716	reg_DEFB	chr8	7307943	G	A	97	97,00%	94			-	SPAG11B	rs2251705		1,00E-012
NA12716	reg_DEFB	chr8	7308164	G	A	60	45,00%	27			-	SPAG11B	rs3817721		1,00E-012
NA12716	reg_DEFB	chr8	7308331	A	G	50	54,00%	27			-	SPAG11B	rs2272769		1,00E-012
NA12716	reg_DEFB	chr8	7308457	C	T	51	39,00%	20			-	SPAG11B		rs71242684	1,00E-012
NA12716	reg_DEFB	chr8	7309001	C	T	50	44,00%	22					rs3762045		1,00E-012
NA12716	reg_DEFB	chr8	7309292	G	A	63	49,00%	31					rs17149290		1,00E-012
NA12716	reg_DEFB	chr8	7309624	T	G	76	50,00%	38					rs2738013		1,00E-012
NA12716	reg_DEFB	chr8	7309975	G	A	57	100,00%	57							1,00E-012
NA12716	reg_DEFB	chr8	7310156	A	C	54	57,00%	31					rs2740702		1,00E-012
NA12716	reg_DEFB	chr8	7310241	G	T	50	90,00%	45					rs4840283		1,00E-012
NA12716	reg_DEFB	chr8	7310265	T	A	47	55,00%	26					rs2740701		1,00E-012

NA12716	reg_DEFB	chr8	7311441	C	T	46	96,00%	44							1,00E-012
NA12716	reg_DEFB	chr8	7311522	A	T	38	100,00%	38							1,00E-012
NA12716	reg_DEFB	chr8	7312534	T	C	57	54,00%	31						rs71242683	1,00E-012
NA12716	reg_DEFB	chr8	7312649	A	G	69	97,00%	67					rs2737570		1,00E-012
NA12716	reg_DEFB	chr8	7313500	C	G	77	53,00%	41					rs2737573		1,00E-012
NA12716	reg_DEFB	chr8	7313761	A	G	72	53,00%	38					rs2737574		1,00E-012
NA12716	reg_DEFB	chr8	7314038	C	T	66	52,00%	34					rs2740026		1,00E-012
NA12716	reg_DEFB	chr8	7314399	C	A	74	43,00%	32					rs2740025		1,00E-012
NA12716	reg_DEFB	chr8	7314602	G	C	94	51,00%	48						rs71242681	1,00E-012
NA12716	reg_DEFB	chr8	7315223	G	A	84	42,00%	35						rs71242680	1,00E-012
NA12716	reg_DEFB	chr8	7315394	C	T	101	38,00%	38	R	Q	-2	DEFB104A		rs71242679	1,00E-012
NA12716	reg_DEFB	chr8	7315477	A	C	97	41,00%	40			-	DEFB104A		rs2740023	1,00E-012
NA12716	reg_DEFB	chr8	7315533	G	A	83	96,00%	80			-	DEFB104A			1,00E-012
NA12716	reg_DEFB	chr8	7315562	G	T	81	37,00%	30			-	DEFB104A	rs2740696		1,00E-012
NA12716	reg_DEFB	chr8	7315640	A	G	70	49,00%	34			-	DEFB104A		rs71242678	1,00E-012
NA12716	reg_DEFB	chr8	7315688	A	G	62	47,00%	29			-	DEFB104A		rs71242677	1,00E-012
NA12716	reg_DEFB	chr8	7315783	T	C	49	43,00%	21			-	DEFB104A		rs71242676	1,00E-012
NA12716	reg_DEFB	chr8	7316113	C	T	40	85,00%	34			-	DEFB104A		rs62639764	1,00E-012
NA12716	reg_DEFB	chr8	7316116	A	C	38	45,00%	17			-	DEFB104A	rs2740021		1,00E-012
NA12716	reg_DEFB	chr8	7316428	C	T	59	97,00%	57			-	DEFB104A			1,00E-012
NA12716	reg_DEFB	chr8	7316983	C	T	59	37,00%	22			-	DEFB104A	rs2740019		1,00E-012
NA12716	reg_DEFB	chr8	7317035	C	T	57	96,00%	55			-	DEFB104A		rs62639766	1,00E-012
NA12716	reg_DEFB	chr8	7317157	G	A	56	36,00%	20			-	DEFB104A		rs62641371	1,00E-012
NA12716	reg_DEFB	chr8	7317239	G	A	50	34,00%	17			-	DEFB104A	rs2737576		1,00E-012
NA12716	reg_DEFB	chr8	7317261	C	A	49	98,00%	48			-	DEFB104A			1,00E-012
NA12716	reg_DEFB	chr8	7317363	C	A	44	55,00%	24			-	DEFB104A	rs2740011		1,00E-012
NA12716	reg_DEFB	chr8	7317396	C	G	42	31,00%	13			-	DEFB104A	rs2740010		1,74E-011
NA12716	reg_DEFB	chr8	7317398	G	A	42	31,00%	13			-	DEFB104A	rs2740695		1,74E-011
NA12716	reg_DEFB	chr8	7317588	G	A	27	56,00%	15			-	DEFB104A	rs6985641		1,00E-012
NA12716	reg_DEFB	chr8	7317699	A	G	16	37,00%	6			-	DEFB104A	rs28681639		9,72E-007
NA12716	reg_DEFB	chr8	7317749	T	G	11	55,00%	6			-	DEFB104A	rs2740007		6,19E-008
NA12716	reg_DEFB	chr8	7317813	C	G	11	100,00%	11			-	DEFB104A	rs7001088		1,00E-012
NA12716	reg_DEFB	chr8	7318591	C	T	24	29,00%	7			-	DEFB104A	rs2680505		8,35E-007
NA12716	reg_DEFB	chr8	7318756	A	T	33	33,00%	11			-	DEFB104A	rs2739991		1,21E-010
NA12716	reg_DEFB	chr8	7318925	T	C	46	59,00%	27			-	DEFB104A	rs2739988		1,00E-012
NA12716	reg_DEFB	chr8	7319055	T	C	51	55,00%	28			-	DEFB104A	rs2739981		1,00E-012
NA12716	reg_DEFB	chr8	7319063	T	C	53	55,00%	29			-	DEFB104A	rs2680506		1,00E-012
NA12716	reg_DEFB	chr8	7319275	A	T	65	52,00%	34			-	DEFB104A	rs2739976		1,00E-012
NA12716	reg_DEFB	chr8	7319456	T	C	64	42,00%	27			-	DEFB104A	rs2739969		1,00E-012
NA12716	reg_DEFB	chr8	7319532	G	C	69	54,00%	37			-	DEFB104A		rs62639768	1,00E-012
NA12716	reg_DEFB	chr8	7319556	A	G	67	34,00%	23			-	DEFB104A		rs2740692	1,00E-012
NA12716	reg_DEFB	chr8	7319599	C	T	71	51,00%	36			-	DEFB104A		rs2739962	1,00E-012
NA12716	reg_DEFB	chr8	7319638	C	T	63	51,00%	32			-	DEFB104A		rs2739960	1,00E-012
NA12716	reg_DEFB	chr8	7319747	A	G	58	98,00%	57			-	DEFB104A	rs4259430		1,00E-012
NA12716	reg_DEFB	chr8	7319771	T	C	64	98,00%	63			-	DEFB104A		rs17843872	1,00E-012
NA12716	reg_DEFB	chr8	7319973	T	C	53	45,00%	24	I	V	-1	DEFB104B	rs2680507		1,00E-012
NA12716	reg_DEFB	chr8	7320136	G	C	36	97,00%	35					rs28590291		1,00E-012
NA12716	reg_DEFB	chr8	7320212	C	G	27	96,00%	26							1,00E-012
NA12716	reg_DEFB	chr8	7320256	C	T	29	97,00%	28							1,00E-012
NA12716	reg_DEFB	chr8	7320337	G	A	36	44,00%	16						rs2740691	1,00E-012
NA12716	reg_DEFB	chr8	7320389	C	T	39	97,00%	38							1,00E-012
NA12716	reg_DEFB	chr8	7320465	C	T	43	100,00%	43							1,00E-012
NA12716	reg_DEFB	chr8	7320516	A	G	42	100,00%	42						rs71308312	1,00E-012

NA12716	reg_DEFB	chr8	7320524	C	G	41	100,00%	41							rs71308312	1,00E-012
NA12716	reg_DEFB	chr8	7320543	A	G	42	98,00%	41								1,00E-012
NA12716	reg_DEFB	chr8	7320547	A	G	41	100,00%	41								1,00E-012
NA12716	reg_DEFB	chr8	7320699	G	A	38	97,00%	37							rs71272074	1,00E-012
NA12716	reg_DEFB	chr8	7320772	A	C	28	96,00%	27					rs2680508			1,00E-012
NA12716	reg_DEFB	chr8	7320916	A	G	35	100,00%	35							rs61413127	1,00E-012
NA12716	reg_DEFB	chr8	7320949	G	A	41	85,00%	35					rs2740690			1,00E-012
NA12716	reg_DEFB	chr8	7320971	C	G	44	39,00%	17					rs2680509			1,00E-012
NA12716	reg_DEFB	chr8	7321565	A	G	33	45,00%	15					rs2739944			1,00E-012
NA12716	reg_DEFB	chr8	7321575	T	A	33	94,00%	31					rs2739943			1,00E-012
NA12716	reg_DEFB	chr8	7321621	C	T	42	93,00%	39							rs2680510	1,00E-012
NA12716	reg_DEFB	chr8	7322171	G	A	45	98,00%	44							rs2680512	1,00E-012
NA12716	reg_DEFB	chr8	7322389	A	G	43	98,00%	42							rs2680515	1,00E-012
NA12716	reg_DEFB	chr8	7322414	C	T	41	95,00%	39							rs2680516	1,00E-012
NA12716	reg_DEFB	chr8	7322882	A	C	31	48,00%	15							rs6472800	1,00E-012
NA12716	reg_DEFB	chr8	7322889	C	T	32	50,00%	16							rs4602913	1,00E-012
NA12716	reg_DEFB	chr8	7322954	C	G	38	97,00%	37					rs28695206			1,00E-012
NA12716	reg_DEFB	chr8	7323016	A	G	48	50,00%	24							rs2680518	1,00E-012
NA12716	reg_DEFB	chr8	7323020	A	T	49	92,00%	45							rs2739908	1,00E-012
NA12716	reg_DEFB	chr8	7323062	T	A	68	51,00%	35							rs2739905	1,00E-012
NA12716	reg_DEFB	chr8	7323118	A	G	82	49,00%	40					rs2737579			1,00E-012
NA12716	reg_DEFB	chr8	7323491	T	G	79	39,00%	31					rs2739894			1,00E-012
NA12716	reg_DEFB	chr8	7323679	C	T	61	100,00%	61					rs28557092	rs41405850		1,00E-012
NA12716	reg_DEFB	chr8	7324031	C	A	56	46,00%	26					rs4840760			1,00E-012
NA12716	reg_DEFB	chr8	7325028	A	G	47	51,00%	24					rs6651517			1,00E-012
NA12716	reg_DEFB	chr8	7325195	C	G	57	39,00%	22							rs71247896	1,00E-012
NA12716	reg_DEFB	chr8	7325767	T	C	42	48,00%	20							rs62639773	1,00E-012
NA12716	reg_DEFB	chr8	7326060	C	T	51	41,00%	21					rs2740125			1,00E-012
NA12716	reg_DEFB	chr8	7326628	C	T	56	100,00%	56							rs62639774	1,00E-012
NA12716	reg_DEFB	chr8	7327328	G	A	36	100,00%	36					rs2293956			1,00E-012
NA12716	reg_DEFB	chr8	7327333	G	A	37	100,00%	37								1,00E-012
NA12716	reg_DEFB	chr8	7327748	G	A	74	54,00%	40			-	DEFB106B	rs2244096			1,00E-012
NA12716	reg_DEFB	chr8	7328290	T	C	50	50,00%	25			-	DEFB106B	rs2740081			1,00E-012
NA12716	reg_DEFB	chr8	7328305	C	A	54	46,00%	25			-	DEFB106B			rs62641365	1,00E-012
NA12716	reg_DEFB	chr8	7328700	C	T	22	100,00%	22			-	DEFB106B	rs28548808			1,00E-012
NA12716	reg_DEFB	chr8	7329944	A	T	4	75,00%	3			-	DEFB106B	rs2740058			4,78E-005
NA12716	reg_DEFB	chr8	7330145	A	T	4	100,00%	4			-	DEFB106B				2,80E-007
NA12716	reg_DEFB	chr8	7330551	T	C	55	40,00%	22			-	DEFB106B	rs6605634			1,00E-012
NA12716	reg_DEFB	chr8	7330574	C	G	56	37,00%	21			-	DEFB106B			rs62639775	1,00E-012
NA12716	reg_DEFB	chr8	7330881	T	A	78	46,00%	36			-	DEFB106B			rs62639776	1,00E-012
NA12716	reg_DEFB	chr8	7331216	C	G	41	46,00%	19			-	DEFB106B	rs2738000			1,00E-012
NA12716	reg_DEFB	chr8	7331608	C	G	59	100,00%	59								1,00E-012
NA12716	reg_DEFB	chr8	7332663	T	A	52	54,00%	28	I	N	1	DEFB105B			rs62639778	1,00E-012
NA12716	reg_DEFB	chr8	7333168	G	C	42	100,00%	42			+	DEFB105A				1,00E-012
NA12716	reg_DEFB	chr8	7334564	C	G	46	61,00%	28							rs62639779	1,00E-012
NA12716	reg_DEFB	chr8	7334844	G	C	38	47,00%	18							rs62639780	1,00E-012
NA12716	reg_DEFB	chr8	7334996	A	G	52	48,00%	25					rs2737595			1,00E-012
NA12716	reg_DEFB	chr8	7335121	T	C	59	95,00%	56								1,00E-012
NA12716	reg_DEFB	chr8	7335401	A	G	57	100,00%	57					rs2977418			1,00E-012
NA12716	reg_DEFB	chr8	7335573	T	C	51	94,00%	48					rs4481626			1,00E-012
NA12716	reg_DEFB	chr8	7336570	C	T	33	42,00%	14							rs62639783	1,00E-012
NA12716	reg_DEFB	chr8	7336931	A	C	11	36,00%	4					rs2737601			8,11E-005
NA12716	reg_DEFB	chr8	7337863	A	C	25	100,00%	25								1,00E-012

NA12716	reg_DEFB	chr8	7338251	A	C	26	38,00%	10			rs2737989		1,61E-010
NA12716	reg_DEFB	chr8	7338279	T	C	28	100,00%	28			rs2946448		1,00E-012
NA12716	reg_DEFB	chr8	7338381	C	T	30	60,00%	18			rs2977421		1,00E-012
NA12716	reg_DEFB	chr8	7338534	G	T	26	50,00%	13			rs2737604		1,00E-012
NA12716	reg_DEFB	chr8	7338734	C	T	31	35,00%	11			rs2680559		5,47E-011
NA12716	reg_DEFB	chr8	7338884	A	G	42	100,00%	42				rs62639786	1,00E-012
NA12716	reg_DEFB	chr8	7339447	C	T	39	95,00%	37				rs62639787	1,00E-012
NA12716	reg_DEFB	chr8	7339485	C	T	42	93,00%	39			rs2680561		1,00E-012
NA12716	reg_DEFB	chr8	7339538	C	G	40	100,00%	40			rs2737606		1,00E-012
NA12716	reg_DEFB	chr8	7339872	A	G	40	100,00%	40			rs2737608		1,00E-012
NA12716	reg_DEFB	chr8	7339927	A	C	40	100,00%	40			rs2737609		1,00E-012
NA12716	reg_DEFB	chr8	7340195	C	A	31	94,00%	29			rs2737988		1,00E-012
NA12716	reg_DEFB	chr8	7340261	C	G	24	46,00%	11				rs62639790	3,07E-012
NA12716	reg_DEFB	chr8	7340710	G	A	48	100,00%	48					1,00E-012
NA12716	reg_DEFB	chr8	7341096	G	C	35	54,00%	19	+	DEFB107A	rs4355796		1,00E-012
NA12716	reg_DEFB	chr8	7341300	T	C	19	89,00%	17	+	DEFB107A	rs12682203		1,00E-012
NA12716	reg_DEFB	chr8	7341317	T	C	18	39,00%	7	+	DEFB107A	rs12682205		8,67E-008
NA12716	reg_DEFB	chr8	7341325	A	G	18	39,00%	7	+	DEFB107A	rs12681807		8,67E-008
NA12716	reg_DEFB	chr8	7341374	C	G	11	45,00%	5	+	DEFB107A		rs62639791	2,65E-006
NA12716	reg_DEFB	chr8	7341377	A	G	11	45,00%	5	+	DEFB107A		rs73366541	2,65E-006
NA12716	reg_DEFB	chr8	7341389	G	T	11	45,00%	5	+	DEFB107A	rs12675434		2,65E-006
NA12716	reg_DEFB	chr8	7341479	T	G	10	50,00%	5	+	DEFB107A		rs6471470	1,47E-006
NA12716	reg_DEFB	chr8	7341618	G	T	32	31,00%	10	+	DEFB107A		rs62641362	1,68E-009
NA12716	reg_DEFB	chr8	7341639	C	T	35	37,00%	13	+	DEFB107A		rs62641361	1,00E-012
NA12716	reg_DEFB	chr8	7342213	G	C	69	52,00%	36	+	DEFB107A		rs73199782	1,00E-012
NA12716	reg_DEFB	chr8	7342346	G	A	67	40,00%	27	+	DEFB107A		rs66911494	1,00E-012
NA12716	reg_DEFB	chr8	7343575	C	T	4	75,00%	3	+	DEFB107A		rs4538904	4,78E-005
NA12716	reg_DEFB	chr8	7343596	C	T	4	100,00%	4	+	DEFB107A		rs9774358	2,80E-007
NA12716	reg_DEFB	chr8	7343619	C	T	4	100,00%	4	+	DEFB107A	rs693031		2,80E-007
NA12716	reg_DEFB	chr8	7343692	C	T	4	100,00%	4	+	DEFB107A		rs62515824	2,80E-007
NA12716	reg_DEFB	chr8	7343696	T	G	4	75,00%	3	+	DEFB107A		rs10102892	4,78E-005
NA12716	reg_DEFB	chr8	7343714	C	T	3	100,00%	3	+	DEFB107A	rs9720857		1,22E-005
NA12716	reg_DEFB	chr8	7348303	A	G	11	91,00%	10	+	DEFB107A			1,00E-012
NA12716	reg_DEFB	chr8	7348338	C	A	10	100,00%	10	+	DEFB107A			1,00E-012
NA12716	reg_DEFB	chr8	7348349	T	C	10	50,00%	5	+	DEFB107A			1,47E-006
NA12716	reg_DEFB	chr8	7348357	A	G	10	80,00%	8	+	DEFB107A			4,25E-012
NA12716	reg_DEFB	chr8	7348411	A	C	9	56,00%	5	+	DEFB107A			7,51E-007
NA12716	reg_DEFB	chr8	7348467	C	A	9	89,00%	8	+	DEFB107A			1,00E-012
NA12716	reg_DEFB	chr8	7348683	A	G	4	100,00%	4	+	DEFB107A			2,80E-007
NA12716	reg_DEFB	chr8	7348763	T	C	4	100,00%	4	+	DEFB107A			2,80E-007
NA12716	reg_DEFB	chr8	7348942	T	C	5	80,00%	4	+	DEFB107A			1,37E-006
NA12716	reg_DEFB	chr8	7349108	A	G	3	100,00%	3	+	DEFB107A			1,22E-005
NA12716	reg_DEFB	chr8	7351384	G	T	3	100,00%	3	+	DEFB107A			1,22E-005
NA12716	reg_DEFB	chr8	7351413	T	G	3	100,00%	3	+	DEFB107A			1,22E-005
NA12716	reg_DEFB	chr8	7352362	G	T	20	95,00%	19	+	DEFB107A		rs2737986	1,00E-012
NA12716	reg_DEFB	chr8	7352697	A	G	58	52,00%	30	+	DEFB107A	rs12155781		1,00E-012
NA12716	reg_DEFB	chr8	7352969	T	C	34	94,00%	32	+	DEFB107A	rs12155828		1,00E-012
NA12716	reg_DEFB	chr8	7353182	T	C	48	44,00%	21	+	DEFB107A	rs12155887		1,00E-012
NA12716	reg_DEFB	chr8	7353385	A	G	73	48,00%	35	+	DEFB107A	rs2737983		1,00E-012
NA12716	reg_DEFB	chr8	7353557	C	T	59	54,00%	32	+	DEFB107A	rs11775409		1,00E-012
NA12716	reg_DEFB	chr8	7353946	C	T	57	47,00%	27	+	DEFB107A		rs62639796	1,00E-012
NA12716	reg_DEFB	chr8	7353996	T	C	63	54,00%	34	+	DEFB107A	rs2737476		1,00E-012
NA12716	reg_DEFB	chr8	7354227	T	C	85	99,00%	84	+	DEFB107A	rs2737477		1,00E-012

NA12716	reg_DEFB	chr8	7354364	A	G	106	44,00%	47	rs4143089		1,00E-012
NA12716	reg_DEFB	chr8	7354376	T	C	108	44,00%	48	rs4143090		1,00E-012
NA12716	reg_DEFB	chr8	7354477	G	A	114	45,00%	51	rs4143091		1,00E-012
NA12716	reg_DEFB	chr8	7354673	C	G	106	98,00%	104		rs62639797	1,00E-012
NA12716	reg_DEFB	chr8	7355739	G	A	17	100,00%	17		rs71511273	1,00E-012
NA12716	reg_DEFB	chr8	7356015	G	A	8	75,00%	6		rs66488835	3,99E-009
NA12716	reg_DEFB	chr8	7357088	T	C	88	49,00%	43	rs2737481		1,00E-012
NA12716	reg_DEFB	chr8	7357411	A	C	89	97,00%	86	rs4840763		1,00E-012
NA12716	reg_DEFB	chr8	7357553	G	A	70	41,00%	29	rs2737979		1,00E-012
NA12716	reg_DEFB	chr8	7357798	C	T	67	52,00%	35		rs2737483	1,00E-012
NA12716	reg_DEFB	chr8	7357880	T	C	68	99,00%	67		rs71509114	1,00E-012
NA12716	reg_DEFB	chr8	7357983	G	A	81	54,00%	44		rs2737978	1,00E-012
NA12716	reg_DEFB	chr8	7358321	C	T	127	51,00%	65		rs71511276	1,00E-012
NA12716	reg_DEFB	chr8	7358618	C	T	109	97,00%	106			1,00E-012
NA12716	reg_DEFB	chr8	7358904	G	A	77	99,00%	76		rs62639798	1,00E-012
NA12716	reg_DEFB	chr8	7358942	C	T	67	42,00%	28	rs2680433		1,00E-012
NA12716	reg_DEFB	chr8	7359000	G	A	57	100,00%	57	rs11984588		1,00E-012
NA12716	reg_DEFB	chr8	7359515	C	A	31	100,00%	31		rs71239493	1,00E-012
NA12716	reg_DEFB	chr8	7359657	T	G	32	100,00%	32		rs62639800	1,00E-012
NA12716	reg_DEFB	chr8	7360635	T	G	36	42,00%	15		rs71259275	1,00E-012
NA12716	reg_DEFB	chr8	7361108	A	G	35	49,00%	17		rs62639801	1,00E-012
NA12716	reg_DEFB	chr8	7361125	T	C	31	58,00%	18		rs62639802	1,00E-012
NA12716	reg_DEFB	chr8	7361343	A	C	24	37,00%	9			1,72E-009
NA12716	reg_DEFB	chr8	7361697	C	T	41	46,00%	19		rs67213127	1,00E-012
NA12716	reg_DEFB	chr8	7361701	G	T	42	90,00%	38		rs62639803	1,00E-012
NA12716	reg_DEFB	chr8	7361941	A	G	24	42,00%	10			6,17E-011
NA12716	reg_DEFB	chr8	7362037	C	G	24	50,00%	12		rs66478539	1,00E-012
NA12716	reg_DEFB	chr8	7362076	A	G	22	45,00%	10		rs71230560	3,38E-011
NA12716	reg_DEFB	chr8	7362278	G	A	17	47,00%	8		rs71230561	1,60E-009
NA12716	reg_DEFB	chr8	7362561	G	C	22	41,00%	9		rs71230562	6,96E-010
NA12716	reg_DEFB	chr8	7362617	A	G	28	89,00%	25		rs71213914	1,00E-012
NA12716	reg_DEFB	chr8	7362619	G	A	29	41,00%	12			1,00E-012
NA12716	reg_DEFB	chr8	7362633	A	G	31	45,00%	14		rs71213915	1,00E-012
NA12716	reg_DEFB	chr8	7362640	A	G	31	45,00%	14		rs71213915	1,00E-012
NA12716	reg_DEFB	chr8	7362978	C	T	36	61,00%	22		rs62639805	1,00E-012
NA12716	reg_DEFB	chr8	7363131	C	T	36	50,00%	18		rs66602902	1,00E-012
NA12716	reg_DEFB	chr8	7363844	A	T	39	59,00%	23		rs2737488	1,00E-012
NA12716	reg_DEFB	chr8	7363941	A	G	36	64,00%	23		rs2680438	1,00E-012
NA12716	reg_DEFB	chr8	7364280	A	G	64	45,00%	29		rs71228231	1,00E-012
NA12716	reg_DEFB	chr8	7364478	C	G	72	99,00%	71	rs2680440		1,00E-012
NA12716	reg_DEFB	chr8	7364562	G	T	74	50,00%	37		rs4590459	1,00E-012
NA12716	reg_DEFB	chr8	7364725	T	A	64	50,00%	32	rs4556104		1,00E-012
NA12716	reg_DEFB	chr8	7365153	T	C	53	28,00%	15		rs71228230	1,89E-012
NA12716	reg_DEFB	chr8	7365196	A	G	57	32,00%	18		rs67575026	1,00E-012
NA12716	reg_DEFB	chr8	7365938	G	A	86	47,00%	40		rs71511278	1,00E-012
NA12716	reg_DEFB	chr8	7366130	G	T	72	46,00%	33		rs71511279	1,00E-012
NA12716	reg_DEFB	chr8	7366324	G	A	61	49,00%	30	rs4584163		1,00E-012
NA12716	reg_DEFB	chr8	7366488	A	G	38	42,00%	16	rs4263787		1,00E-012
NA12716	reg_DEFB	chr8	7366494	A	C	38	97,00%	37	rs4270988		1,00E-012
NA12716	reg_DEFB	chr8	7366516	C	G	38	45,00%	17	rs4446761		1,00E-012
NA12716	reg_DEFB	chr8	7366531	T	G	38	47,00%	18	rs4335141		1,00E-012
NA12716	reg_DEFB	chr8	7367473	C	A	55	35,00%	19	rs4440657		1,00E-012
NA12716	reg_DEFB	chr8	7367818	T	C	12	100,00%	12		rs7842766	1,00E-012

NA12716	reg_DEFB	chr8	7367967	G	A	10	40,00%	4			5,26E-005
NA12716	reg_DEFB	chr8	7368330	G	T	58	50,00%	29			1,00E-012
NA12716	reg_DEFB	chr8	7368407	T	G	71	48,00%	34		rs4599836	1,00E-012
NA12716	reg_DEFB	chr8	7368542	T	A	73	55,00%	40		rs71511292	1,00E-012
NA12716	reg_DEFB	chr8	7369708	C	T	84	46,00%	39	rs725058		1,00E-012
NA12716	reg_DEFB	chr8	7370010	A	G	67	40,00%	27			1,00E-012
NA12716	reg_DEFB	chr8	7370028	T	G	60	45,00%	27	rs2680484		1,00E-012
NA12716	reg_DEFB	chr8	7370060	T	C	61	39,00%	24			1,00E-012
NA12716	reg_DEFB	chr8	7370074	C	A	57	51,00%	29		rs62639806	1,00E-012
NA12716	reg_DEFB	chr8	7370610	A	G	82	40,00%	33		rs2680482	1,00E-012
NA12716	reg_DEFB	chr8	7371475	C	T	59	41,00%	24	rs4840766		1,00E-012
NA12716	reg_DEFB	chr8	7372793	T	C	88	43,00%	38			1,00E-012
NA12716	reg_DEFB	chr8	7373254	C	T	27	59,00%	16	rs1807385		1,00E-012
NA12716	reg_DEFB	chr8	7374467	T	C	54	44,00%	24			1,00E-012
NA12716	reg_DEFB	chr8	7375518	G	A	49	45,00%	22	rs4840769		1,00E-012
NA12716	reg_DEFB	chr8	7375596	C	A	45	49,00%	22	rs11786478		1,00E-012
NA12716	reg_DEFB	chr8	7375818	C	T	30	30,00%	9		rs71264915	1,66E-008
NA12716	reg_DEFB	chr8	7376276	A	C	43	42,00%	18			1,00E-012
NA12716	reg_DEFB	chr8	7376945	T	G	29	45,00%	13		rs71249123	1,00E-012
NA12716	reg_DEFB	chr8	7377165	A	G	25	48,00%	12		rs71249124	1,00E-012
NA12716	reg_DEFB	chr8	7377464	C	A	28	100,00%	28			1,00E-012
NA12716	reg_DEFB	chr8	7378147	C	T	23	48,00%	11	rs2977689		1,66E-012
NA12716	reg_DEFB	chr8	7378654	T	C	13	100,00%	13		rs71249125	1,00E-012
NA12716	reg_DEFB	chr8	7379026	G	A	11	91,00%	10		rs67847292	1,00E-012
NA12716	reg_DEFB	chr8	7379048	T	C	11	100,00%	11		rs71249126	1,00E-012
NA12716	reg_DEFB	chr8	7379519	C	T	15	27,00%	4		rs62639811	3,12E-004
NA12716	reg_DEFB	chr8	7380428	G	A	41	100,00%	41			1,00E-012
NA12716	reg_DEFB	chr8	7380733	A	G	68	53,00%	36	rs2954331		1,00E-012
NA12716	reg_DEFB	chr8	7380984	G	C	89	46,00%	41	rs2977404		1,00E-012
NA12716	reg_DEFB	chr8	7381451	A	G	65	48,00%	31	rs2977687		1,00E-012
NA12716	reg_DEFB	chr8	7381476	G	A	60	97,00%	58	rs2737939		1,00E-012
NA12716	reg_DEFB	chr8	7381963	G	A	36	31,00%	11	rs4311672		3,56E-010
NA12716	reg_DEFB	chr8	7382091	A	C	42	90,00%	38	rs2737936		1,00E-012
NA12716	reg_DEFB	chr8	7382305	C	G	45	38,00%	17	rs2737499		1,00E-012
NA12716	reg_DEFB	chr8	7382406	C	A	36	50,00%	18	rs4461922		1,00E-012
NA12716	reg_DEFB	chr8	7382462	C	T	46	52,00%	24	rs4633079		1,00E-012
NA12716	reg_DEFB	chr8	7382473	T	G	45	51,00%	23		rs62639815	1,00E-012
NA12716	reg_DEFB	chr8	7382556	T	C	42	55,00%	23		rs62639816	1,00E-012
NA12716	reg_DEFB	chr8	7382833	G	A	77	100,00%	77			1,00E-012
NA12716	reg_DEFB	chr8	7383272	A	C	65	54,00%	35		rs67373661	1,00E-012
NA12716	reg_DEFB	chr8	7383406	C	T	58	55,00%	32	rs4504661		1,00E-012
NA12716	reg_DEFB	chr8	7383498	T	C	52	42,00%	22	rs4392927		1,00E-012
NA12716	reg_DEFB	chr8	7383738	A	G	50	52,00%	26	rs2977686		1,00E-012
NA12716	reg_DEFB	chr8	7383983	A	G	43	51,00%	22	rs2977685		1,00E-012
NA12716	reg_DEFB	chr8	7384348	A	C	89	60,00%	53	rs2737932		1,00E-012
NA12716	reg_DEFB	chr8	7385749	G	A	49	55,00%	27	rs2737931		1,00E-012
NA12716	reg_DEFB	chr8	7385987	T	C	54	44,00%	24	rs2946446		1,00E-012
NA12716	reg_DEFB	chr8	7386060	T	A	47	47,00%	22		rs62639820	1,00E-012
NA12716	reg_DEFB	chr8	7386182	T	G	54	54,00%	29	rs2737502		1,00E-012
NA12716	reg_DEFB	chr8	7386319	T	A	55	64,00%	35		rs62639821	1,00E-012
NA12716	reg_DEFB	chr8	7386396	G	A	56	66,00%	37		rs62639822	1,00E-012
NA12716	reg_DEFB	chr8	7386594	C	T	58	24,00%	14	rs2017780		5,05E-011
NA12716	reg_DEFB	chr8	7386641	T	C	50	72,00%	36	rs2737503		1,00E-012

NA12716	reg_DEFB	chr8	7386669	A	G	46	65,00%	30							rs62639824	1,00E-012
NA12716	reg_DEFB	chr8	7386935	A	G	76	50,00%	38							rs62639825	1,00E-012
NA12716	reg_DEFB	chr8	7387038	C	A	90	44,00%	40					rs3175182			1,00E-012
NA12716	reg_DEFB	chr8	7387328	T	G	83	46,00%	38					rs4840284			1,00E-012
NA12716	reg_DEFB	chr8	7387420	C	G	68	44,00%	30					rs2258428			1,00E-012
NA12716	reg_DEFB	chr8	7388003	A	G	26	46,00%	12					rs2737508			1,00E-012
NA12716	reg_DEFB	chr8	7388132	C	G	27	48,00%	13					rs2946444			1,00E-012
NA12716	reg_DEFB	chr8	7389499	A	G	72	50,00%	36					rs2075896			1,00E-012
NA12716	reg_DEFB	chr8	7390072	C	G	87	51,00%	44					rs2946443			1,00E-012
NA12716	reg_DEFB	chr8	7390501	T	A	100	47,00%	47					rs4840774	rs2737514		1,00E-012
NA12716	reg_DEFB	chr8	7390503	C	G	101	50,00%	51					rs4840775			1,00E-012
NA12716	reg_DEFB	chr8	7390657	G	A	83	59,00%	49							rs2737889	1,00E-012
NA12716	reg_DEFB	chr8	7390776	G	A	79	96,00%	76					rs2737515			1,00E-012
NA12716	reg_DEFB	chr8	7390893	A	T	63	43,00%	27							rs62639831	1,00E-012
NA12716	reg_DEFB	chr8	7391064	T	G	39	100,00%	39					rs4840776			1,00E-012
NA12716	reg_DEFB	chr8	7391199	G	C	14	29,00%	4					rs2737885			2,33E-004
NA12716	reg_DEFB	chr8	7391201	A	T	14	64,00%	9							rs2954332	4,45E-012
NA12716	reg_CTRL	chr8	8213964	C	T	18	44,00%	8	A	T	-1	PRAGMIN	rs12549973			2,79E-009
NA12716	reg_CTRL	chr8	8214235	A	G	58	31,00%	18			-	PRAGMIN	rs6601691			1,00E-012
NA12716	reg_CTRL	chr8	8214527	A	G	68	49,00%	33			-	PRAGMIN	rs6996347			1,00E-012
NA12716	reg_CTRL	chr8	8214897	A	C	74	54,00%	40			-	PRAGMIN	rs4840922			1,00E-012
NA12716	reg_CTRL	chr8	8215271	G	A	68	44,00%	30			-	PRAGMIN	rs2980503			1,00E-012
NA12716	reg_CTRL	chr8	8215510	T	C	78	54,00%	42			-	PRAGMIN	rs13276576			1,00E-012
NA12716	reg_CTRL	chr8	8215633	T	C	87	100,00%	87			-	PRAGMIN	rs1548198			1,00E-012
NA12716	reg_CTRL	chr8	8217059	C	G	31	55,00%	17			-	PRAGMIN	rs4840923			1,00E-012
NA12716	reg_CTRL	chr8	8217969	G	C	61	48,00%	29			-	PRAGMIN	rs2102866			1,00E-012
NA12716	reg_CTRL	chr8	8218156	G	A	32	56,00%	18			-	PRAGMIN	rs12674854			1,00E-012
NA12716	reg_CTRL	chr8	8218928	G	C	86	44,00%	38			-	PRAGMIN	rs867757			1,00E-012
NA12716	reg_CTRL	chr8	8218968	A	T	89	56,00%	50			-	PRAGMIN	rs2945898			1,00E-012
NA12716	reg_CTRL	chr8	8219680	G	C	54	48,00%	26			-	PRAGMIN	rs939069			1,00E-012
NA12716	reg_CTRL	chr8	8221093	T	C	8	50,00%	4			-	PRAGMIN	rs2945899			1,82E-005
NA12716	reg_CTRL	chr8	8223577	T	C	55	45,00%	25			-	PRAGMIN	rs2979208			1,00E-012
NA12716	reg_CTRL	chr8	8223747	A	T	58	57,00%	33			-	PRAGMIN	rs2976910			1,00E-012
NA12716	reg_CTRL	chr8	8223842	C	T	53	41,00%	22			-	PRAGMIN	rs10099374			1,00E-012
NA12716	reg_CTRL	chr8	8224229	G	T	38	29,00%	11			-	PRAGMIN	rs2945900			6,48E-010
NA12716	reg_CTRL	chr8	8224912	A	G	82	56,00%	46			-	PRAGMIN	rs2979210			1,00E-012
NA12716	reg_CTRL	chr8	8224960	T	C	76	57,00%	43			-	PRAGMIN	rs2945901			1,00E-012
NA12716	reg_CTRL	chr8	8225163	A	G	41	41,00%	17			-	PRAGMIN	rs2980498	rs2945902		1,00E-012
NA12716	reg_CTRL	chr8	8225164	T	C	41	41,00%	17			-	PRAGMIN	rs2945902			1,00E-012
NA12716	reg_CTRL	chr8	8225230	T	C	45	49,00%	22			-	PRAGMIN	rs2945903			1,00E-012
NA12716	reg_CTRL	chr8	8225641	G	A	78	50,00%	39			-	PRAGMIN	rs2979211			1,00E-012
NA12716	reg_CTRL	chr8	8225874	G	A	87	55,00%	48			-	PRAGMIN	rs2979212			1,00E-012
NA12716	reg_CTRL	chr8	8225954	G	A	93	56,00%	52			-	PRAGMIN	rs2979213			1,00E-012
NA12716	reg_CTRL	chr8	8225986	A	G	98	57,00%	56			-	PRAGMIN	rs2976917			1,00E-012
NA12716	reg_CTRL	chr8	8226005	A	G	97	53,00%	51			-	PRAGMIN	rs2976918			1,00E-012
NA12716	reg_CTRL	chr8	8226193	A	G	100	55,00%	55			-	PRAGMIN	rs2979215			1,00E-012
NA12716	reg_CTRL	chr8	8226315	T	C	101	48,00%	48			-	PRAGMIN	rs2979216			1,00E-012
NA12716	reg_CTRL	chr8	8226494	G	A	100	26,00%	26			-	PRAGMIN	rs2979217			1,00E-012
NA12716	reg_CTRL	chr8	8226774	G	C	87	41,00%	36			-	PRAGMIN	rs2979218			1,00E-012
NA12716	reg_CTRL	chr8	8226802	G	A	82	44,00%	36			-	PRAGMIN	rs2979219			1,00E-012
NA12716	reg_CTRL	chr8	8226809	A	G	81	44,00%	36			-	PRAGMIN	rs2976927			1,00E-012
NA12716	reg_CTRL	chr8	8226848	T	C	84	100,00%	84			-	PRAGMIN	rs2980497			1,00E-012
NA12716	reg_CTRL	chr8	8226912	G	A	86	44,00%	38			-	PRAGMIN	rs2945904			1,00E-012

NA12716	reg_CTRL	chr8	8226996	T	G	71	48,00%	34	-	PRAGMIN	rs2945905		1,00E-012
NA12716	reg_CTRL	chr8	8227475	T	C	43	51,00%	22	-	PRAGMIN	rs2945907		1,00E-012
NA12716	reg_CTRL	chr8	8227809	T	G	90	34,00%	31	-	PRAGMIN	rs1518992		1,00E-012
NA12716	reg_CTRL	chr8	8227825	T	C	87	34,00%	30	-	PRAGMIN	rs1850724		1,00E-012
NA12716	reg_CTRL	chr8	8227900	C	G	98	40,00%	39	-	PRAGMIN	rs1850725		1,00E-012
NA12716	reg_CTRL	chr8	8227975	A	G	99	41,00%	41	-	PRAGMIN	rs1850726		1,00E-012
NA12716	reg_CTRL	chr8	8228199	T	C	88	36,00%	32	-	PRAGMIN	rs2980496		1,00E-012
NA12716	reg_CTRL	chr8	8228481	T	G	122	50,00%	61	-	PRAGMIN	rs2979220		1,00E-012
NA12716	reg_CTRL	chr8	8229144	C	T	30	60,00%	18	-	PRAGMIN	rs2980495		1,00E-012
NA12716	reg_CTRL	chr8	8229293	T	C	22	45,00%	10	-	PRAGMIN	rs2979221		3,38E-011
NA12716	reg_CTRL	chr8	8229301	G	A	21	43,00%	9	-	PRAGMIN	rs2976947		4,19E-010
NA12716	reg_CTRL	chr8	8229490	C	T	54	52,00%	28	-	PRAGMIN			1,00E-012
NA12716	reg_CTRL	chr8	8229639	A	C	54	48,00%	26	-	PRAGMIN	rs2945908		1,00E-012
NA12716	reg_CTRL	chr8	8229935	T	C	32	47,00%	15	-	PRAGMIN	rs2976952		1,00E-012
NA12716	reg_CTRL	chr8	8230241	T	C	48	50,00%	24	-	PRAGMIN	rs2976954		1,00E-012
NA12716	reg_CTRL	chr8	8230250	A	C	47	51,00%	24	-	PRAGMIN	rs2979222		1,00E-012
NA12716	reg_CTRL	chr8	8230631	T	C	21	33,00%	7	-	PRAGMIN	rs2980494		2,98E-007
NA12716	reg_CTRL	chr8	8230864	G	A	39	100,00%	39	-	PRAGMIN	rs2979223		1,00E-012
NA12716	reg_CTRL	chr8	8231138	C	T	86	52,00%	45	-	PRAGMIN	rs2979224		1,00E-012
NA12716	reg_CTRL	chr8	8231195	T	G	83	47,00%	39	-	PRAGMIN	rs2976963		1,00E-012
NA12716	reg_CTRL	chr8	8232030	A	C	28	50,00%	14	-	PRAGMIN	rs2979226		1,00E-012
NA12716	reg_CTRL	chr8	8232156	C	G	50	50,00%	25	-	PRAGMIN	rs9329270		1,00E-012
NA12716	reg_CTRL	chr8	8232408	G	A	85	58,00%	49	-	PRAGMIN	rs2976840		1,00E-012
NA12716	reg_CTRL	chr8	8232580	A	C	72	47,00%	34	-	PRAGMIN	rs2945910		1,00E-012
NA12716	reg_CTRL	chr8	8233348	C	T	102	49,00%	50	-	PRAGMIN	rs2976852		1,00E-012
NA12716	reg_CTRL	chr8	8233599	T	C	128	48,00%	61	-	PRAGMIN	rs2980491		1,00E-012
NA12716	reg_CTRL	chr8	8235132	A	G	111	40,00%	44	-	PRAGMIN	rs2945912		1,00E-012
NA12716	reg_CTRL	chr8	8235635	C	T	80	44,00%	35	-	PRAGMIN	rs2945913		1,00E-012
NA12716	reg_CTRL	chr8	8235716	C	G	84	58,00%	49	-	PRAGMIN	rs4840337		1,00E-012
NA12716	reg_CTRL	chr8	8235760	T	G	84	44,00%	37	-	PRAGMIN	rs2980490		1,00E-012
NA12716	reg_CTRL	chr8	8236281	C	T	72	42,00%	30	-	PRAGMIN	rs2976887		1,00E-012
NA12716	reg_CTRL	chr8	8236848	A	C	9	100,00%	9	-	PRAGMIN	rs2945914		1,00E-012
NA12716	reg_CTRL	chr8	8237374	G	A	25	52,00%	13	-	PRAGMIN		rs62496027	1,00E-012
NA12716	reg_CTRL	chr8	8238677	A	G	37	100,00%	37	-	PRAGMIN	rs2176631		1,00E-012
NA12716	reg_CTRL	chr8	8238782	A	G	35	60,00%	21	-	PRAGMIN	rs2945839		1,00E-012
NA12716	reg_CTRL	chr8	8239704	A	G	54	37,00%	20	-	PRAGMIN	rs2980489		1,00E-012
NA12716	reg_CTRL	chr8	8242420	C	T	87	99,00%	86	-	PRAGMIN	rs11785239		1,00E-012
NA12716	reg_CTRL	chr8	8242644	A	T	65	52,00%	34	-	PRAGMIN	rs13273161		1,00E-012
NA12716	reg_CTRL	chr8	8243226	C	T	95	100,00%	95	-	PRAGMIN	rs6990504		1,00E-012
NA12716	reg_CTRL	chr8	8244163	A	G	17	100,00%	17	-	PRAGMIN	rs7833103		1,00E-012
NA12716	reg_CTRL	chr8	8244749	A	G	58	43,00%	25	-	PRAGMIN	rs17150353		1,00E-012
NA12716	reg_CTRL	chr8	8246169	G	C	44	50,00%	22	-	PRAGMIN	rs11786306		1,00E-012
NA12716	reg_CTRL	chr8	8246601	G	T	96	100,00%	96	-	PRAGMIN	rs4840932		1,00E-012
NA12716	reg_CTRL	chr8	8249567	T	C	49	65,00%	32	-	PRAGMIN	rs34796521		1,00E-012
NA12716	reg_CTRL	chr8	8252759	T	C	74	45,00%	33	-	PRAGMIN	rs4840939		1,00E-012
NA12716	reg_CTRL	chr8	8254396	A	G	66	100,00%	66	-	PRAGMIN	rs4840941		1,00E-012
NA12716	reg_CTRL	chr8	8254926	C	T	48	50,00%	24	-	PRAGMIN	rs11778125		1,00E-012
NA12716	reg_CTRL	chr8	8256007	T	C	61	100,00%	61	-	PRAGMIN	rs7005904		1,00E-012
NA12716	reg_CTRL	chr8	8256108	G	C	50	44,00%	22	-	PRAGMIN	rs10099225		1,00E-012
NA12716	reg_CTRL	chr8	8256279	T	C	36	58,00%	21	-	PRAGMIN	rs7006376		1,00E-012
NA12716	reg_CTRL	chr8	8256377	A	G	43	98,00%	42	-	PRAGMIN	rs4840338		1,00E-012
NA12716	reg_CTRL	chr8	8256592	G	A	74	100,00%	74	-	PRAGMIN	rs724265		1,00E-012
NA12716	reg_CTRL	chr8	8256759	G	A	82	45,00%	37	-	PRAGMIN	rs724266		1,00E-012

NA12716	reg_CTRL	chr8	8258014	C	T	7	43,00%	3	-	PRAGMIN	rs28578995		3,97E-004
NA12716	reg_CTRL	chr8	8258498	C	T	20	45,00%	9	-	PRAGMIN	rs1914826		2,44E-010
NA12716	reg_CTRL	chr8	8258721	C	G	18	50,00%	9	-	PRAGMIN	rs1914825		7,33E-011
NA12716	reg_CTRL	chr8	8258769	G	A	26	46,00%	12	-	PRAGMIN	rs1914824		1,00E-012
NA12716	reg_CTRL	chr8	8259890	T	C	72	100,00%	72	-	PRAGMIN	rs2030279		1,00E-012
NA12716	reg_CTRL	chr8	8262672	A	G	8	100,00%	8	-	PRAGMIN	rs13282599		1,00E-012
NA12716	reg_CTRL	chr8	8269101	A	G	121	99,00%	120	-	PRAGMIN	rs13280051		1,00E-012
NA12716	reg_CTRL	chr8	8273641	C	T	19	95,00%	18	-	PRAGMIN	rs34742161		1,00E-012
NA12716	reg_CTRL	chr8	8274087	C	T	49	100,00%	49	-	PRAGMIN	rs2976958		1,00E-012
NA12716	reg_CTRL	chr8	8274368	T	C	107	42,00%	45	-	PRAGMIN			1,00E-012
NA12716	reg_CTRL	chr8	8679890	G	A	62	98,00%	61	-	MFHAS1	rs4841038		1,00E-012
NA12716	reg_CTRL	chr8	8680797	G	A	23	100,00%	23	-	MFHAS1	rs10903311		1,00E-012
NA12716	reg_CTRL	chr8	8680992	C	G	71	100,00%	71	-	MFHAS1	rs2271340		1,00E-012
NA12716	reg_CTRL	chr8	8681135	T	A	119	100,00%	119	-	MFHAS1	rs2271341		1,00E-012
NA12716	reg_CTRL	chr8	8681348	C	T	116	99,00%	115	-	MFHAS1	rs2271342		1,00E-012
NA12716	reg_CTRL	chr8	8681684	G	C	39	95,00%	37	-	MFHAS1	rs12677543		1,00E-012
NA12716	reg_CTRL	chr8	8681732	G	A	41	98,00%	40	-	MFHAS1	rs12677550		1,00E-012
NA12716	reg_CTRL	chr8	8682101	T	C	39	97,00%	38	-	MFHAS1	rs7015606		1,00E-012
NA12716	reg_CTRL	chr8	8682217	A	G	36	56,00%	20	-	MFHAS1	rs7010952		1,00E-012
NA12716	reg_CTRL	chr8	8682221	T	C	38	53,00%	20	-	MFHAS1		rs61853364	1,00E-012
NA12716	reg_CTRL	chr8	8683135	G	C	116	99,00%	115	-	MFHAS1	rs2409088		1,00E-012
NA12716	reg_CTRL	chr8	8683656	T	C	52	100,00%	52	-	MFHAS1	rs12682352		1,00E-012
NA12716	reg_CTRL	chr8	8686807	T	C	67	100,00%	67	-	MFHAS1	rs2409089		1,00E-012
NA12716	reg_CTRL	chr8	8687291	C	T	35	97,00%	34	-	MFHAS1	rs11249891		1,00E-012
NA12716	reg_CTRL	chr8	8688829	T	C	60	97,00%	58	-	MFHAS1	rs6601732		1,00E-012
NA12716	reg_CTRL	chr8	8690299	G	A	68	99,00%	67	-	MFHAS1	rs7832968		1,00E-012
NA12716	reg_CTRL	chr8	8691268	G	A	63	100,00%	63	-	MFHAS1	rs2409090		1,00E-012
NA12716	reg_CTRL	chr8	8691467	G	A	11	100,00%	11	-	MFHAS1	rs2409091		1,00E-012
NA12716	reg_CTRL	chr8	8691521	C	G	6	100,00%	6	-	MFHAS1	rs7460947		1,48E-010
NA12716	reg_CTRL	chr8	8691687	G	A	5	80,00%	4	-	MFHAS1		rs71576013	1,37E-006
NA12716	reg_CTRL	chr8	8691772	T	C	12	92,00%	11	-	MFHAS1			1,00E-012
NA12716	reg_CTRL	chr8	8691951	C	G	25	96,00%	24	-	MFHAS1	rs4841041		1,00E-012
NA12716	reg_CTRL	chr8	8692433	C	T	46	100,00%	46	-	MFHAS1	rs3748144		1,00E-012
NA12716	reg_CTRL	chr8	8695950	A	G	41	95,00%	39	-	MFHAS1	rs2048419		1,00E-012
NA12716	reg_CTRL	chr8	8697085	G	T	133	100,00%	133	-	MFHAS1	rs13282015		1,00E-012
NA12716	reg_CTRL	chr8	8697948	C	A	76	97,00%	74	-	MFHAS1	rs6994038		1,00E-012
NA12716	reg_CTRL	chr8	8698944	T	C	73	100,00%	73	-	MFHAS1	rs12547493		1,00E-012
NA12716	reg_CTRL	chr8	8699091	C	G	99	100,00%	99	-	MFHAS1	rs12544992		1,00E-012
NA12716	reg_CTRL	chr8	8700625	C	T	28	79,00%	22	-	MFHAS1	rs28399241		1,00E-012
NA12716	reg_CTRL	chr8	8701507	C	T	42	100,00%	42	-	MFHAS1	rs9329167		1,00E-012
NA12716	reg_CTRL	chr8	8701722	C	A	17	88,00%	15	-	MFHAS1	rs7015271		1,00E-012
NA12716	reg_CTRL	chr8	8702032	G	A	41	100,00%	41	-	MFHAS1	rs4841042		1,00E-012
NA12716	reg_CTRL	chr8	8702089	G	A	59	90,00%	53	-	MFHAS1	rs4841043		1,00E-012
NA12716	reg_CTRL	chr8	8702350	G	A	83	98,00%	81	-	MFHAS1	rs4841044		1,00E-012
NA12716	reg_CTRL	chr8	8702557	G	A	88	97,00%	85	-	MFHAS1	rs11783966		1,00E-012
NA12716	reg_CTRL	chr8	8703143	T	A	43	95,00%	41	-	MFHAS1	rs9644775		1,00E-012
NA12716	reg_CTRL	chr8	8703212	T	C	36	61,00%	22	-	MFHAS1	rs9644776		1,00E-012
NA12716	reg_CTRL	chr8	8704326	C	T	75	99,00%	74	-	MFHAS1	rs6988939		1,00E-012
NA12716	reg_CTRL	chr8	8704329	G	T	76	97,00%	74	-	MFHAS1	rs2175161		1,00E-012
NA12716	reg_CTRL	chr8	8704854	C	T	70	100,00%	70	-	MFHAS1	rs6993494		1,00E-012
NA12716	reg_CTRL	chr8	8705807	A	G	84	100,00%	84	-	MFHAS1	rs7006418		1,00E-012
NA12716	reg_CTRL	chr8	8705896	A	G	84	100,00%	84	-	MFHAS1	rs7006589		1,00E-012
NA12716	reg_CTRL	chr8	8706327	A	C	69	100,00%	69	-	MFHAS1	rs1473029		1,00E-012

NA12716	reg_CTRL	chr8	8707492	G	C	85	100,00%	85	-	MFHAS1	rs4840362		1,00E-012
NA12716	reg_CTRL	chr8	8707587	T	A	64	100,00%	64	-	MFHAS1	rs7823757		1,00E-012
NA12716	reg_CTRL	chr8	8708009	A	G	21	100,00%	21	-	MFHAS1		rs60315134	1,00E-012
NA12716	reg_CTRL	chr8	8708146	C	A	31	100,00%	31	-	MFHAS1		rs59046059	1,00E-012
NA12716	reg_CTRL	chr8	8709372	C	T	65	100,00%	65	-	MFHAS1	rs11784052		1,00E-012
NA12716	reg_CTRL	chr8	8709629	T	A	49	86,00%	42	-	MFHAS1	rs10088933		1,00E-012
NA12716	reg_CTRL	chr8	8709839	G	C	15	93,00%	14	-	MFHAS1	rs11777085		1,00E-012
NA12716	reg_CTRL	chr8	8709989	A	G	25	100,00%	25	-	MFHAS1	rs4841045		1,00E-012
NA12716	reg_CTRL	chr8	8710211	C	T	11	91,00%	10	-	MFHAS1	rs4841046		1,00E-012
NA12716	reg_CTRL	chr8	8710362	A	C	20	90,00%	18	-	MFHAS1	rs4841047		1,00E-012
NA12716	reg_CTRL	chr8	8710730	T	C	109	99,00%	108	-	MFHAS1	rs13265731		1,00E-012
NA12716	reg_CTRL	chr8	8711011	A	C	129	99,00%	128	-	MFHAS1	rs13259216		1,00E-012
NA12716	reg_CTRL	chr8	8711146	T	C	107	98,00%	105	-	MFHAS1	rs35431455		1,00E-012
NA12716	reg_CTRL	chr8	8712586	A	G	41	100,00%	41	-	MFHAS1	rs13260419		1,00E-012
NA12716	reg_CTRL	chr8	8712735	A	T	58	100,00%	58	-	MFHAS1	rs35039922		1,00E-012
NA12716	reg_CTRL	chr8	8713900	T	C	60	47,00%	28	-	MFHAS1	rs950721		1,00E-012
NA12716	reg_CTRL	chr8	8714960	C	T	36	36,00%	13	-	MFHAS1	rs13280206		1,00E-012
NA12716	reg_CTRL	chr8	8715940	G	A	90	100,00%	90	-	MFHAS1	rs882462		1,00E-012
NA12716	reg_CTRL	chr8	8716586	A	G	145	100,00%	145	-	MFHAS1	rs11775523		1,00E-012
NA12716	reg_CTRL	chr8	8716735	G	C	110	97,00%	107	-	MFHAS1	rs28755903		1,00E-012
NA12716	reg_CTRL	chr8	8716866	G	C	79	99,00%	78	-	MFHAS1	rs1039913		1,00E-012
NA12716	reg_CTRL	chr8	8716959	C	T	73	99,00%	72	-	MFHAS1	rs1039914		1,00E-012
NA12716	reg_CTRL	chr8	8717024	T	C	56	98,00%	55	-	MFHAS1	rs1039915		1,00E-012
NA12716	reg_CTRL	chr8	8717493	G	A	10	90,00%	9	-	MFHAS1	rs11779585		1,00E-012
NA12716	reg_CTRL	chr8	8717887	G	A	65	94,00%	61	-	MFHAS1		rs57312668	1,00E-012
NA12716	reg_CTRL	chr8	8718276	C	G	71	100,00%	71	-	MFHAS1	rs4840364		1,00E-012
NA12716	reg_CTRL	chr8	8718775	A	C	75	96,00%	72	-	MFHAS1	rs4841049		1,00E-012
NA12716	reg_CTRL	chr8	8719000	T	C	82	100,00%	82	-	MFHAS1	rs4841050		1,00E-012
NA12716	reg_CTRL	chr8	8719166	T	C	86	100,00%	86	-	MFHAS1	rs1876836		1,00E-012
NA12716	reg_CTRL	chr8	8719602	A	T	81	99,00%	80	-	MFHAS1	rs2409092		1,00E-012
NA12716	reg_CTRL	chr8	8720288	T	C	22	100,00%	22	-	MFHAS1	rs12545499		1,00E-012
NA12716	reg_CTRL	chr8	8720310	G	C	24	100,00%	24	-	MFHAS1	rs2409094		1,00E-012
NA12716	reg_CTRL	chr8	8720681	A	C	58	100,00%	58	-	MFHAS1	rs907179		1,00E-012
NA12716	reg_CTRL	chr8	8720696	G	C	60	43,00%	26	-	MFHAS1	rs34731491		1,00E-012
NA12716	reg_CTRL	chr8	8722363	G	A	86	100,00%	86	-	MFHAS1	rs1533059		1,00E-012
NA12716	reg_CTRL	chr8	8722600	A	G	46	100,00%	46	-	MFHAS1	rs1533058		1,00E-012
NA12716	reg_CTRL	chr8	8723056	T	C	90	100,00%	90	-	MFHAS1	rs4841051		1,00E-012
NA12716	reg_CTRL	chr8	8723264	A	G	126	100,00%	126	-	MFHAS1	rs1039916		1,00E-012
NA12716	reg_CTRL	chr8	8724090	T	A	88	98,00%	86	-	MFHAS1	rs2409095		1,00E-012
NA12716	reg_CTRL	chr8	8724464	G	C	38	95,00%	36	-	MFHAS1	rs3789849		1,00E-012
NA12716	reg_CTRL	chr8	8726960	G	A	48	67,00%	32	-	MFHAS1			1,00E-012
NA12716	reg_CTRL	chr8	8726976	G	A	20	95,00%	19	-	MFHAS1			1,00E-012
NA12716	reg_CTRL	chr8	8726978	G	A	20	95,00%	19	-	MFHAS1			1,00E-012
NA12716	reg_CTRL	chr8	8726980	G	A	20	95,00%	19	-	MFHAS1			1,00E-012
NA12716	reg_CTRL	chr8	8726982	G	A	20	95,00%	19	-	MFHAS1	rs28821557		1,00E-012
NA12716	reg_CTRL	chr8	8727010	G	A	21	33,00%	7	-	MFHAS1		rs71949256	2,98E-007
NA12716	reg_CTRL	chr8	8727376	G	C	83	90,00%	75	-	MFHAS1	rs4840366		1,00E-012
NA12716	reg_CTRL	chr8	8727803	G	T	48	96,00%	46	-	MFHAS1	rs11995244		1,00E-012
NA12716	reg_CTRL	chr8	8727836	T	C	48	100,00%	48	-	MFHAS1	rs13259619		1,00E-012
NA12716	reg_CTRL	chr8	8728197	C	T	56	98,00%	55	-	MFHAS1	rs13259070		1,00E-012
NA12716	reg_CTRL	chr8	8728228	C	A	59	41,00%	24	-	MFHAS1			1,00E-012
NA12716	reg_CTRL	chr8	8728794	C	G	42	98,00%	41	-	MFHAS1	rs9329169		1,00E-012
NA12716	reg_CTRL	chr8	8729032	T	A	42	100,00%	42	-	MFHAS1	rs13270070		1,00E-012

NA12716	reg_CTRL	chr8	8729476	G	C	93	100,00%	93	-	MFHAS1	rs1510932		1,00E-012
NA12716	reg_CTRL	chr8	8729887	T	C	69	91,00%	63	-	MFHAS1	rs2409096		1,00E-012
NA12716	reg_CTRL	chr8	8729950	C	G	63	98,00%	62	-	MFHAS1	rs1510933		1,00E-012
NA12716	reg_CTRL	chr8	8731603	C	G	55	96,00%	53	-	MFHAS1	rs13254903		1,00E-012
NA12716	reg_CTRL	chr8	8732984	C	T	98	100,00%	98	-	MFHAS1	rs6601265		1,00E-012
NA12716	reg_CTRL	chr8	8733859	T	G	51	96,00%	49	-	MFHAS1	rs1510934		1,00E-012
NA12716	reg_CTRL	chr8	8734939	C	A	99	42,00%	42	-	MFHAS1	rs36104437		1,00E-012
NA12716	reg_CTRL	chr8	8735502	C	T	85	98,00%	83	-	MFHAS1	rs4841054		1,00E-012
NA12716	reg_CTRL	chr8	8736571	A	G	78	95,00%	74	-	MFHAS1	rs4841055		1,00E-012
NA12716	reg_CTRL	chr8	8737167	T	A	41	93,00%	38	-	MFHAS1	rs7820146		1,00E-012
NA12716	reg_CTRL	chr8	8737171	C	T	41	100,00%	41	-	MFHAS1	rs7833171		1,00E-012
NA12716	reg_CTRL	chr8	8738012	G	C	33	30,00%	10	-	MFHAS1	rs7017006		2,37E-009
NA12716	reg_CTRL	chr8	8738114	C	G	36	44,00%	16	-	MFHAS1		rs4752439	1,00E-012
NA12716	reg_CTRL	chr8	8738166	C	G	26	35,00%	9	-	MFHAS1		rs71163465	3,95E-009
NA12716	reg_CTRL	chr8	8738261	C	T	12	100,00%	12	-	MFHAS1	rs11249893		1,00E-012
NA12716	reg_CTRL	chr8	8740017	G	C	85	89,00%	76	-	MFHAS1	rs7820738		1,00E-012
NA12716	reg_CTRL	chr8	8740237	A	G	65	97,00%	63	-	MFHAS1	rs907180		1,00E-012
NA12716	reg_CTRL	chr8	8740285	T	C	63	97,00%	61	-	MFHAS1	rs907181		1,00E-012
NA12716	reg_CTRL	chr8	8741091	C	T	13	100,00%	13	-	MFHAS1	rs6996376		1,00E-012
NA12716	reg_CTRL	chr8	8741740	G	C	100	99,00%	99	-	MFHAS1	rs4481596		1,00E-012
NA12716	reg_CTRL	chr8	8743619	A	C	81	98,00%	79	-	MFHAS1	rs11249896		1,00E-012
NA12716	reg_CTRL	chr8	8743742	A	C	60	97,00%	58	-	MFHAS1	rs408459		1,00E-012
NA12716	reg_CTRL	chr8	8744607	C	G	78	95,00%	74	-	MFHAS1	rs1877119		1,00E-012
NA12716	reg_CTRL	chr8	8746124	A	C	88	100,00%	88	-	MFHAS1	rs440788		1,00E-012
NA12716	reg_CTRL	chr8	8746384	C	G	101	95,00%	96	-	MFHAS1	rs3925830		1,00E-012
NA12716	reg_CTRL	chr8	8747166	G	C	50	100,00%	50	-	MFHAS1	rs1964719		1,00E-012
NA12716	reg_CTRL	chr8	8747381	C	T	48	98,00%	47	-	MFHAS1	rs3958877		1,00E-012
NA12716	reg_CTRL	chr8	8748211	G	A	68	97,00%	66	-	MFHAS1	rs437895		1,00E-012
NA12716	reg_CTRL	chr8	8750305	A	G	53	58,00%	31	-	MFHAS1	rs4348501		1,00E-012
NA12716	reg_CTRL	chr8	8750413	A	G	45	64,00%	29	-	MFHAS1	rs13268671		1,00E-012
NA12716	reg_CTRL	chr8	8750416	G	A	43	100,00%	43	-	MFHAS1	rs231188		1,00E-012
NA12716	reg_CTRL	chr8	8750448	C	T	41	37,00%	15	-	MFHAS1	rs4523255		1,00E-012
NA12716	reg_CTRL	chr8	8751363	T	C	72	56,00%	40	-	MFHAS1		rs56073940	1,00E-012
NA12716	reg_CTRL	chr8	8752647	T	A	33	30,00%	10	-	MFHAS1			2,37E-009
NA12716	reg_CTRL	chr8	8754381	C	T	59	34,00%	20	-	MFHAS1	rs5023278		1,00E-012
NA12716	reg_CTRL	chr8	8756260	G	A	148	97,00%	144	-	MFHAS1	rs1039917		1,00E-012
NA12716	reg_CTRL	chr8	8756923	G	A	57	95,00%	54	-	MFHAS1	rs35900578		1,00E-012
NA12716	reg_CTRL	chr8	8758883	G	A	51	100,00%	51	-	MFHAS1	rs4382480		1,00E-012
NA12716	reg_CTRL	chr8	8759109	G	A	46	50,00%	23	-	MFHAS1		rs71514515	1,00E-012
NA12716	reg_CTRL	chr8	8759186	C	A	63	44,00%	28	-	MFHAS1			1,00E-012
NA12716	reg_CTRL	chr8	8759937	G	A	35	49,00%	17	-	MFHAS1		rs56367294	1,00E-012
NA12716	reg_CTRL	chr8	8760085	C	T	23	43,00%	10	-	MFHAS1	rs332037		3,67E-011
NA12716	reg_CTRL	chr8	8761061	C	G	68	49,00%	33	-	MFHAS1	rs332039		1,00E-012
NA12716	reg_CTRL	chr8	8761328	G	T	37	41,00%	15	-	MFHAS1	rs3789845		1,00E-012
NA12716	reg_CTRL	chr8	8761667	C	T	74	51,00%	38	-	MFHAS1	rs3789843		1,00E-012
NA12716	reg_CTRL	chr8	8761686	C	T	74	54,00%	40	-	MFHAS1	rs3827806		1,00E-012
NA12716	reg_CTRL	chr8	8761825	C	T	67	43,00%	29	-	MFHAS1		rs60707155	1,00E-012
NA12716	reg_CTRL	chr8	8762536	G	T	13	31,00%	4	-	MFHAS1	rs7017599		1,69E-004
NA12716	reg_CTRL	chr8	8762639	G	A	18	33,00%	6	-	MFHAS1	rs1821007		2,17E-006
NA12716	reg_CTRL	chr8	8762729	G	A	35	49,00%	17	-	MFHAS1	rs1821008		1,00E-012
NA12716	reg_CTRL	chr8	8764214	G	T	132	55,00%	73	-	MFHAS1	rs1567398		1,00E-012
NA12716	reg_CTRL	chr8	8766603	A	C	95	99,00%	94	-	MFHAS1	rs13274028		1,00E-012
NA12716	reg_CTRL	chr8	8767171	G	C	115	99,00%	114	-	MFHAS1	rs907183		1,00E-012

NA12716	reg_CTRL	chr8	8767898	G	A	68	100,00%	68	-	MFHAS1	rs332040		1,00E-012
NA12716	reg_CTRL	chr8	8768326	G	C	65	97,00%	63	-	MFHAS1	rs4841058		1,00E-012
NA12716	reg_CTRL	chr8	8768646	G	A	70	53,00%	37	-	MFHAS1		rs61591712	1,00E-012
NA12716	reg_CTRL	chr8	8769293	C	T	65	95,00%	62	-	MFHAS1	rs9644694		1,00E-012
NA12716	reg_CTRL	chr8	8770735	G	C	33	100,00%	33	-	MFHAS1	rs2009455		1,00E-012
NA12716	reg_CTRL	chr8	8772507	A	C	86	100,00%	86	-	MFHAS1	rs10046783		1,00E-012
NA12716	reg_CTRL	chr8	8772623	A	C	96	99,00%	95	-	MFHAS1	rs10046784		1,00E-012
NA12716	reg_CTRL	chr8	8773796	A	G	59	100,00%	59	-	MFHAS1	rs12679021		1,00E-012
NA12716	reg_CTRL	chr8	8774098	G	A	16	100,00%	16	-	MFHAS1	rs12681432		1,00E-012
NA12716	reg_CTRL	chr8	8774113	A	T	17	76,00%	13	-	MFHAS1	rs7824578		1,00E-012
NA12716	reg_CTRL	chr8	8774196	G	A	17	82,00%	14	-	MFHAS1	rs13261380		1,00E-012
NA12716	reg_CTRL	chr8	8774295	G	T	35	57,00%	20	-	MFHAS1	rs34599909		1,00E-012
NA12716	reg_CTRL	chr8	8774325	G	T	41	39,00%	16	-	MFHAS1		rs60965369	1,00E-012
NA12716	reg_CTRL	chr8	8774535	T	C	99	49,00%	49	-	MFHAS1		rs73192206	1,00E-012
NA12716	reg_CTRL	chr8	8774874	T	G	124	98,00%	122	-	MFHAS1	rs409997		1,00E-012
NA12716	reg_CTRL	chr8	8775015	T	C	131	99,00%	130	-	MFHAS1	rs410487		1,00E-012
NA12716	reg_CTRL	chr8	8775668	A	G	30	100,00%	30	-	MFHAS1	rs381800		1,00E-012
NA12716	reg_CTRL	chr8	8776018	C	G	70	47,00%	33	-	MFHAS1		rs72626639	1,00E-012
NA12716	reg_CTRL	chr8	8780758	G	T	38	100,00%	38	-	MFHAS1	rs435393		1,00E-012
NA12716	reg_CTRL	chr8	8781650	G	A	33	42,00%	14	-	MFHAS1	rs7818276		1,00E-012
NA12716	reg_CTRL	chr8	8784947	T	C	143	99,00%	142	-	MFHAS1	rs399123		1,00E-012
NA12716	reg_CTRL	chr8	11738154	G	C	52	98,00%	51	-	CTSB	rs1736077		1,00E-012
NA12716	reg_CTRL	chr8	11738505	A	C	67	37,00%	25	-	CTSB	rs8005		1,00E-012
NA12716	reg_CTRL	chr8	11738607	G	A	60	95,00%	57	-	CTSB	rs12898		1,00E-012
NA12716	reg_CTRL	chr8	11738662	A	G	46	54,00%	25	-	CTSB	rs2740592		1,00E-012
NA12716	reg_CTRL	chr8	11738687	G	A	40	60,00%	24	-	CTSB	rs2645425		1,00E-012
NA12716	reg_CTRL	chr8	11739251	A	C	105	100,00%	105	-	CTSB	rs1736078		1,00E-012
NA12716	reg_CTRL	chr8	11739342	T	C	108	45,00%	49	-	CTSB	rs4839		1,00E-012
NA12716	reg_CTRL	chr8	11739415	A	T	109	52,00%	57	-	CTSB	rs9009		1,00E-012
NA12716	reg_CTRL	chr8	11739613	G	A	86	98,00%	84	-	CTSB	rs6730		1,00E-012
NA12716	reg_CTRL	chr8	11739722	C	G	69	39,00%	27	-	CTSB	rs709822		1,00E-012
NA12716	reg_CTRL	chr8	11739784	G	A	66	42,00%	28	-	CTSB	rs3947		1,00E-012
NA12716	reg_CTRL	chr8	11740003	G	C	64	37,00%	24	-	CTSB	rs709821		1,00E-012
NA12716	reg_CTRL	chr8	11740249	T	G	88	44,00%	39	-	CTSB	rs1736081		1,00E-012
NA12716	reg_CTRL	chr8	11740457	C	G	69	41,00%	28	-	CTSB	rs1692811		1,00E-012
NA12716	reg_CTRL	chr8	11740829	C	T	42	29,00%	12	-	CTSB	rs1736082		1,38E-010
NA12716	reg_CTRL	chr8	11740906	G	T	48	40,00%	19	-	CTSB	rs1736083		1,00E-012
NA12716	reg_CTRL	chr8	11740932	T	G	50	42,00%	21	-	CTSB	rs1692812		1,00E-012
NA12716	reg_CTRL	chr8	11741061	A	G	58	43,00%	25	-	CTSB	rs6601616		1,00E-012
NA12716	reg_CTRL	chr8	11741066	C	T	60	55,00%	33	-	CTSB	rs1736084		1,00E-012
NA12716	reg_CTRL	chr8	11741068	T	C	59	54,00%	32	-	CTSB	rs1736085		1,00E-012
NA12716	reg_CTRL	chr8	11741705	G	T	54	48,00%	26	-	CTSB	rs1692813		1,00E-012
NA12716	reg_CTRL	chr8	11741707	T	C	54	48,00%	26	-	CTSB	rs1692814		1,00E-012
NA12716	reg_CTRL	chr8	11741759	G	C	46	93,00%	43	-	CTSB	rs1692815		1,00E-012
NA12716	reg_CTRL	chr8	11741866	A	C	35	97,00%	34	-	CTSB	rs1692816		1,00E-012
NA12716	reg_CTRL	chr8	11742128	C	A	29	45,00%	13	-	CTSB	rs1692817		1,00E-012
NA12716	reg_CTRL	chr8	11742287	C	G	34	94,00%	32	-	CTSB	rs1692818		1,00E-012
NA12716	reg_CTRL	chr8	11742306	C	T	36	50,00%	18	-	CTSB	rs1736086		1,00E-012
NA12716	reg_CTRL	chr8	11742548	C	T	22	59,00%	13	-	CTSB	rs2294138		1,00E-012
NA12716	reg_CTRL	chr8	11742751	G	C	19	89,00%	17	-	CTSB	rs1736088		1,00E-012
NA12716	reg_CTRL	chr8	11742851	G	T	26	46,00%	12	-	CTSB	rs2294139		1,00E-012
NA12716	reg_CTRL	chr8	11742857	G	A	26	46,00%	12	-	CTSB	rs1692819		1,00E-012
NA12716	reg_CTRL	chr8	11743086	C	G	69	61,00%	42	-	CTSB	rs2294140		1,00E-012

NA12716	reg_CTRL	chr8	11743279	G	A	74	41,00%	30			-	CTSB	rs1736089		1,00E-012
NA12716	reg_CTRL	chr8	11743638	T	C	63	92,00%	58			-	CTSB	rs1736090		1,00E-012
NA12716	reg_CTRL	chr8	11743990	T	G	32	91,00%	29	T	T	-1	CTSB	rs13332		1,00E-012
NA12716	reg_CTRL	chr8	11744243	T	C	35	49,00%	17			-	CTSB	rs35581201		1,00E-012
NA12716	reg_CTRL	chr8	11744265	C	T	31	97,00%	30			-	CTSB	rs13280858		1,00E-012
NA12716	reg_CTRL	chr8	11744315	T	C	27	96,00%	26			-	CTSB	rs13254438		1,00E-012
NA12716	reg_CTRL	chr8	11744346	G	C	29	93,00%	27			-	CTSB	rs13278902		1,00E-012
NA12716	reg_CTRL	chr8	11744382	T	C	32	100,00%	32			-	CTSB	rs4840586		1,00E-012
NA12716	reg_CTRL	chr8	11744417	C	A	27	100,00%	27			-	CTSB	rs2645423		1,00E-012
NA12716	reg_CTRL	chr8	11744436	A	G	24	96,00%	23			-	CTSB	rs2740593		1,00E-012
NA12716	reg_CTRL	chr8	11744441	G	C	23	96,00%	22			-	CTSB	rs2645422		1,00E-012
NA12716	reg_CTRL	chr8	11744583	A	G	7	71,00%	5			-	CTSB	rs2740594		1,30E-007
NA12716	reg_CTRL	chr8	11744998	G	A	3	100,00%	3			-	CTSB	rs2645420		1,22E-005
NA12716	reg_CTRL	chr8	11745209	T	C	6	100,00%	6			-	CTSB	rs2645419		1,48E-010
NA12716	reg_CTRL	chr8	11745764	G	A	80	44,00%	35			-	CTSB	rs2272766		1,00E-012
NA12716	reg_CTRL	chr8	11746124	A	T	34	44,00%	15			-	CTSB	rs28577034		1,00E-012
NA12716	reg_CTRL	chr8	11746209	T	G	29	48,00%	14			-	CTSB	rs2645417		1,00E-012
NA12716	reg_CTRL	chr8	11746239	C	G	27	37,00%	10			-	CTSB	rs2740595		2,51E-010
NA12716	reg_CTRL	chr8	11746416	G	A	53	40,00%	21			-	CTSB		rs62495697	1,00E-012
NA12716	reg_CTRL	chr8	11746725	C	G	25	60,00%	15			-	CTSB	rs1961986		1,00E-012
NA12716	reg_CTRL	chr8	11747366	T	C	30	40,00%	12			-	CTSB	rs1293290		2,51E-012
NA12716	reg_CTRL	chr8	11747710	A	G	58	98,00%	57			-	CTSB	rs1293291		1,00E-012
NA12716	reg_CTRL	chr8	11747778	G	A	67	45,00%	30			-	CTSB	rs1293292		1,00E-012
NA12716	reg_CTRL	chr8	11748038	T	A	99	50,00%	50			-	CTSB	rs1122182		1,00E-012
NA12716	reg_CTRL	chr8	11748297	G	C	80	51,00%	41	L	V	-1	CTSB	rs12338		1,00E-012
NA12716	reg_CTRL	chr8	11748383	G	A	67	46,00%	31			-	CTSB	rs17154017		1,00E-012
NA12716	reg_CTRL	chr8	11748468	C	T	63	43,00%	27			-	CTSB	rs2272767		1,00E-012
NA12716	reg_CTRL	chr8	11748558	C	A	59	51,00%	30			-	CTSB	rs1293295		1,00E-012
NA12716	reg_CTRL	chr8	11748566	A	C	60	48,00%	29			-	CTSB	rs1293296		1,00E-012
NA12716	reg_CTRL	chr8	11748882	G	C	61	44,00%	27			-	CTSB	rs1293297		1,00E-012
NA12716	reg_CTRL	chr8	11749852	A	C	43	42,00%	18			-	CTSB	rs1293298		1,00E-012
NA12716	reg_CTRL	chr8	11750048	C	T	19	26,00%	5			-	CTSB	rs1736103		5,71E-005
NA12716	reg_CTRL	chr8	11751261	A	G	40	50,00%	20			-	CTSB	rs17154027		1,00E-012
NA12716	reg_CTRL	chr8	11751714	C	T	49	51,00%	25			-	CTSB	rs17814426		1,00E-012
NA12716	reg_CTRL	chr8	11752509	A	T	50	50,00%	25			-	CTSB	rs9644756		1,00E-012
NA12716	reg_CTRL	chr8	11752657	C	T	68	47,00%	32			-	CTSB			1,00E-012
NA12716	reg_CTRL	chr8	11753882	G	C	106	43,00%	46			-	CTSB	rs6980952		1,00E-012
NA12716	reg_CTRL	chr8	11755937	T	C	95	42,00%	40			-	CTSB	rs1293288		1,00E-012
NA12716	reg_CTRL	chr8	11756896	T	C	42	62,00%	26			-	CTSB	rs2142470		1,00E-012
NA12716	reg_CTRL	chr8	11757636	G	C	100	45,00%	45			-	CTSB	rs1293303		1,00E-012
NA12716	reg_CTRL	chr8	11757954	A	G	68	40,00%	27			-	CTSB	rs1293304		1,00E-012
NA12716	reg_CTRL	chr8	11758394	C	A	74	45,00%	33			-	CTSB	rs1293305		1,00E-012
NA12716	reg_CTRL	chr8	11759455	A	G	39	51,00%	20			-	CTSB	rs2645415		1,00E-012
NA12716	reg_CTRL	chr8	11759534	C	T	40	50,00%	20			-	CTSB	rs1299525		1,00E-012
NA12716	reg_CTRL	chr8	11759764	G	A	66	41,00%	27			-	CTSB	rs1296022		1,00E-012
NA12716	reg_CTRL	chr8	11760540	A	G	36	97,00%	35			-	CTSB	rs1293307		1,00E-012
NA12716	reg_CTRL	chr8	11761184	G	A	43	51,00%	22			-	CTSB	rs1293309		1,00E-012
NA12716	reg_CTRL	chr8	12624420	G	A	92	100,00%	92			-	LONRF1	rs7005881		1,00E-012
NA12716	reg_CTRL	chr8	12625320	T	C	121	51,00%	62			-	LONRF1		rs73202627	1,00E-012
NA12716	reg_CTRL	chr8	12626051	T	C	90	100,00%	90			-	LONRF1	rs4831767		1,00E-012
NA12716	reg_CTRL	chr8	12626226	C	T	95	47,00%	45			-	LONRF1	rs4831768		1,00E-012
NA12716	reg_CTRL	chr8	12626235	A	G	94	99,00%	93			-	LONRF1	rs4831769		1,00E-012
NA12716	reg_CTRL	chr8	12627074	T	A	102	99,00%	101			-	LONRF1	rs10429335		1,00E-012

NA12716	reg_CTRL	chr8	12627915	C	T	104	98,00%	102			-	LONRF1	rs11782145		1,00E-012
NA12716	reg_CTRL	chr8	12628025	T	C	113	88,00%	99			-	LONRF1	rs10100866		1,00E-012
NA12716	reg_CTRL	chr8	12628463	T	C	96	97,00%	93			-	LONRF1		rs72603953	1,00E-012
NA12716	reg_CTRL	chr8	12628721	A	G	51	98,00%	50			-	LONRF1	rs9632851		1,00E-012
NA12716	reg_CTRL	chr8	12629030	C	T	14	93,00%	13			-	LONRF1	rs10095845		1,00E-012
NA12716	reg_CTRL	chr8	12629393	C	A	36	53,00%	19			-	LONRF1		rs73202633	1,00E-012
NA12716	reg_CTRL	chr8	12629532	A	T	60	97,00%	58			-	LONRF1	rs6530953		1,00E-012
NA12716	reg_CTRL	chr8	12629807	C	G	102	99,00%	101			-	LONRF1	rs7010337		1,00E-012
NA12716	reg_CTRL	chr8	12630635	A	C	116	49,00%	57			-	LONRF1		rs73202635	1,00E-012
NA12716	reg_CTRL	chr8	12631165	G	C	73	56,00%	41	T	S	-3	LONRF1			1,00E-012
NA12716	reg_CTRL	chr8	12631166	T	A	72	58,00%	42	T	S	-3	LONRF1			1,00E-012
NA12716	reg_CTRL	chr8	12631700	T	C	41	56,00%	23			-	LONRF1	rs17761564		1,00E-012
NA12716	reg_CTRL	chr8	12631939	T	C	56	50,00%	28			-	LONRF1		rs56114121	1,00E-012
NA12716	reg_CTRL	chr8	12632233	T	A	75	41,00%	31			-	LONRF1	rs17761606		1,00E-012
NA12716	reg_CTRL	chr8	12632654	A	C	46	98,00%	45			-	LONRF1	rs6995647		1,00E-012
NA12716	reg_CTRL	chr8	12633550	C	A	80	62,00%	50			-	LONRF1	rs3802269		1,00E-012
NA12716	reg_CTRL	chr8	12634490	G	C	77	56,00%	43			-	LONRF1	rs4272378		1,00E-012
NA12716	reg_CTRL	chr8	12634940	G	A	39	46,00%	18			-	LONRF1	rs7463601		1,00E-012
NA12716	reg_CTRL	chr8	12634992	A	G	29	100,00%	29			-	LONRF1	rs7461006		1,00E-012
NA12716	reg_CTRL	chr8	12635185	A	G	46	100,00%	46			-	LONRF1	rs6530956		1,00E-012
NA12716	reg_CTRL	chr8	12635399	G	A	45	93,00%	42			-	LONRF1	rs6530958		1,00E-012
NA12716	reg_CTRL	chr8	12635958	A	C	28	100,00%	28			-	LONRF1	rs4258004		1,00E-012
NA12716	reg_CTRL	chr8	12636319	C	T	81	99,00%	80			-	LONRF1	rs6530959		1,00E-012
NA12716	reg_CTRL	chr8	12637327	C	A	86	97,00%	83			-	LONRF1	rs13251315		1,00E-012
NA12716	reg_CTRL	chr8	12637416	A	G	82	93,00%	76			-	LONRF1	rs13272425		1,00E-012
NA12716	reg_CTRL	chr8	12637740	T	C	39	44,00%	17			-	LONRF1	rs11784110		1,00E-012
NA12716	reg_CTRL	chr8	12637991	T	C	41	100,00%	41			-	LONRF1	rs9325786		1,00E-012
NA12716	reg_CTRL	chr8	12640631	C	T	69	99,00%	68			-	LONRF1	rs7014187		1,00E-012
NA12716	reg_CTRL	chr8	12641320	A	G	48	40,00%	19			-	LONRF1	rs4831777		1,00E-012
NA12716	reg_CTRL	chr8	12641415	C	G	46	57,00%	26			-	LONRF1		rs73202639	1,00E-012
NA12716	reg_CTRL	chr8	12642348	C	A	76	71,00%	54			-	LONRF1	rs6530962		1,00E-012
NA12716	reg_CTRL	chr8	12642503	A	C	74	96,00%	71			-	LONRF1	rs4625037		1,00E-012
NA12716	reg_CTRL	chr8	12642559	T	C	67	48,00%	32			-	LONRF1	rs11775169		1,00E-012
NA12716	reg_CTRL	chr8	12642979	C	T	53	98,00%	52			-	LONRF1	rs4831354		1,00E-012
NA12716	reg_CTRL	chr8	12643126	C	T	57	65,00%	37			-	LONRF1	rs7819033		1,00E-012
NA12716	reg_CTRL	chr8	12643453	A	C	47	100,00%	47			-	LONRF1	rs4831780		1,00E-012
NA12716	reg_CTRL	chr8	12643642	A	C	41	93,00%	38			-	LONRF1	rs10098734		1,00E-012
NA12716	reg_CTRL	chr8	12643654	G	A	39	100,00%	39			-	LONRF1	rs10110145		1,00E-012
NA12716	reg_CTRL	chr8	12643838	T	C	47	100,00%	47			-	LONRF1	rs7014429		1,00E-012
NA12716	reg_CTRL	chr8	12644993	C	T	50	98,00%	49			-	LONRF1	rs7837242		1,00E-012
NA12716	reg_CTRL	chr8	12645405	C	T	59	53,00%	31			-	LONRF1	rs6530964		1,00E-012
NA12716	reg_CTRL	chr8	12645526	A	C	57	100,00%	57			-	LONRF1	rs6530965		1,00E-012
NA12716	reg_CTRL	chr8	12646136	C	G	36	100,00%	36			-	LONRF1	rs7842201		1,00E-012
NA12716	reg_CTRL	chr8	12646342	T	C	56	98,00%	55			-	LONRF1	rs7819248		1,00E-012
NA12716	reg_CTRL	chr8	12646912	G	C	63	98,00%	62			-	LONRF1	rs4831784		1,00E-012
NA12716	reg_CTRL	chr8	12647560	C	G	95	48,00%	46			-	LONRF1	rs6985289		1,00E-012
NA12716	reg_CTRL	chr8	12647982	A	T	107	93,00%	100			-	LONRF1	rs6530966		1,00E-012
NA12716	reg_CTRL	chr8	12648329	G	C	100	100,00%	100			-	LONRF1	rs7838660		1,00E-012
NA12716	reg_CTRL	chr8	12648422	T	C	101	100,00%	101			-	LONRF1	rs7832448		1,00E-012
NA12716	reg_CTRL	chr8	12648780	C	G	83	40,00%	33			-	LONRF1	rs9325792		1,00E-012
NA12716	reg_CTRL	chr8	12648809	T	C	78	58,00%	45			-	LONRF1		rs73202647	1,00E-012
NA12716	reg_CTRL	chr8	12648863	A	G	75	43,00%	32			-	LONRF1	rs9325793		1,00E-012
NA12716	reg_CTRL	chr8	12649062	G	T	59	100,00%	59			-	LONRF1	rs7014516		1,00E-012

NA12716	reg_CTRL	chr8	12649595	G	A	25	100,00%	25			-	LONRF1	rs10441667			1,00E-012
NA12716	reg_CTRL	chr8	12650052	T	C	20	65,00%	13			-	LONRF1	rs17767600			1,00E-012
NA12716	reg_CTRL	chr8	12650145	C	T	18	22,00%	4			-	LONRF1	rs7462166			6,61E-004
NA12716	reg_CTRL	chr8	12651062	T	C	73	53,00%	39			-	LONRF1	rs4436128			1,00E-012
NA12716	reg_CTRL	chr8	12651131	T	C	68	51,00%	35			-	LONRF1	rs4437649			1,00E-012
NA12716	reg_CTRL	chr8	12651557	C	G	36	39,00%	14			-	LONRF1	rs10503427			1,00E-012
NA12716	reg_CTRL	chr8	12651680	C	T	29	100,00%	29			-	LONRF1	rs7007056			1,00E-012
NA12716	reg_CTRL	chr8	12652000	C	T	51	98,00%	50			-	LONRF1	rs7007550			1,00E-012
NA12716	reg_CTRL	chr8	12652284	C	G	50	100,00%	50			-	LONRF1	rs6530968			1,00E-012
NA12716	reg_CTRL	chr8	12652948	G	A	74	50,00%	37			-	LONRF1			rs73202653	1,00E-012
NA12716	reg_CTRL	chr8	12653361	C	T	9	44,00%	4			-	LONRF1	rs6988764			3,21E-005
NA12716	reg_CTRL	chr8	12653488	C	G	14	100,00%	14			-	LONRF1	rs4831795			1,00E-012
NA12716	reg_CTRL	chr8	12654972	A	G	139	100,00%	139			-	LONRF1	rs6530969			1,00E-012
NA12716	reg_CTRL	chr8	12655147	T	C	107	50,00%	54			-	LONRF1	rs6530970			1,00E-012
NA12760	reg_CTRL	chr17	31286367	A	G	45	49,00%	22			-	LYZL6	rs17676662			1,00E-012
NA12760	reg_CTRL	chr17	31288659	T	C	58	53,00%	31			-	LYZL6	rs2280784			1,00E-012
NA12760	reg_CTRL	chr17	31288972	A	G	26	58,00%	15	D	D	-2	LYZL6	rs2280783			1,00E-012
NA12760	reg_CTRL	chr17	31289472	C	T	15	27,00%	4			-	LYZL6	rs11654215			3,12E-004
NA12760	reg_CTRL	chr17	31289599	G	A	10	50,00%	5			-	LYZL6				1,47E-006
NA12760	reg_CTRL	chr17	31290141	T	C	45	49,00%	22			-	LYZL6	rs9901969			1,00E-012
NA12760	reg_CTRL	chr19	59866310	T	C	9	33,00%	3	F	L	1	LILRB4	rs28366008			9,21E-004
NA12760	reg_CTRL	chr19	59867552	C	T	9	44,00%	4	F	F	2	LILRB4	rs3745871			3,21E-005
NA12760	reg_CTRL	chr19	59870501	T	G	7	43,00%	3			+	LILRB4				3,97E-004
NA12760	reg_CTRL	chr19	59870545	A	G	7	43,00%	3			+	LILRB4	rs11574582			3,97E-004
NA12760	reg_CTRL	chr19	59870587	G	A	11	36,00%	4			+	LILRB4				8,11E-005
NA12760	reg_CTRL	chr19	59870794	G	A	9	78,00%	7			+	LILRB4	rs11574587			1,44E-010
NA12760	reg_CTRL	chr19	59870823	T	C	9	33,00%	3			+	LILRB4			rs71365476	9,21E-004
NA12760	reg_CTRL	chr19	59870834	G	A	10	50,00%	5			+	LILRB4				1,47E-006
NA12760	reg_CTRL	chr19	59870860	A	G	10	40,00%	4			+	LILRB4				5,26E-005
NA12760	reg_CTRL	chr19	59870864	T	C	10	50,00%	5			+	LILRB4				1,47E-006
NA12760	reg_CTRL	chr19	60079765	G	A	11	36,00%	4			+	FCAR	rs10407012			8,11E-005
NA12760	reg_CTRL	chr19	60081277	A	G	17	53,00%	9			+	FCAR	rs4806604			5,40E-011
NA12760	reg_CTRL	chr19	60082413	A	G	10	40,00%	4			+	FCAR	rs7257926			5,26E-005
NA12760	reg_CTRL	chr19	60082645	G	A	10	40,00%	4			+	FCAR	rs10402725			5,26E-005
NA12760	reg_CTRL	chr19	60084881	T	C	7	86,00%	6			+	FCAR	rs7248382	rs7247547		1,02E-009
NA12760	reg_CTRL	chr19	60085431	A	G	13	100,00%	13			+	FCAR	rs4239591			1,00E-012
NA12760	reg_CTRL	chr19	60085626	A	G	17	53,00%	9			+	FCAR	rs4806606			5,40E-011
NA12760	reg_CTRL	chr19	60087161	C	G	9	56,00%	5			+	FCAR	rs28756208			7,51E-007
NA12760	reg_CTRL	chr19	60088124	G	A	6	100,00%	6			+	FCAR	rs10401687			1,48E-010
NA12760	reg_CTRL	chr19	60088425	G	A	20	100,00%	20			+	FCAR	rs10402324			1,00E-012
NA12760	reg_CTRL	chr19	60088712	A	G	48	98,00%	47	R	R	2	FCAR	rs1865096			1,00E-012
NA12760	reg_CTRL	chr19	60089029	A	G	32	100,00%	32			+	FCAR	rs1865097			1,00E-012
NA12760	reg_CTRL	chr19	60089192	C	T	21	95,00%	20			+	FCAR	rs11666846			1,00E-012
NA12760	reg_CTRL	chr19	60089677	T	C	7	100,00%	7			+	FCAR	rs12974020			3,40E-012
NA12760	reg_CTRL	chr19	60089829	T	C	14	93,00%	13			+	FCAR	rs12974530			1,00E-012
NA12760	reg_CTRL	chr19	60089913	A	G	18	100,00%	18			+	FCAR	rs12972637			1,00E-012
NA12760	reg_CTRL	chr19	60090047	T	C	13	100,00%	13			+	FCAR	rs12975083			1,00E-012
NA12760	reg_CTRL	chr19	60091123	G	T	10	40,00%	4			+	FCAR	rs7258679			5,26E-005
NA12760	reg_CTRL	chr19	60092542	C	A	5	80,00%	4			+	FCAR	rs12976517			1,37E-006
NA12760	reg_CTRL	chr19	60092982	A	G	37	46,00%	17	S	G	2	FCAR	rs16986050			1,00E-012
NA12760	reg_CTRL	chr19	60093536	C	T	13	46,00%	6			+	FCAR	rs10413148			2,21E-007
NA12760	reg_CTRL	chr19	60093544	T	G	13	46,00%	6			+	FCAR	rs10414707			2,21E-007
NA12760	reg_CTRL	chr20	29530984	A	G	51	100,00%	51			+	REM1	rs215911			1,00E-012

NA12760	reg_CTRL	chr20	29531308	T	C	19	21,00%	4			+	REM1			8,22E-004
NA12760	reg_CTRL	chr20	29531866	T	C	24	21,00%	5			+	REM1	rs13037125		1,90E-004
NA12760	reg_CTRL	chr20	29566293	C	T	11	73,00%	8			+	HM13			1,56E-011
NA12760	reg_CTRL	chr20	29567135	C	G	39	44,00%	17			+	HM13	rs6088440		1,00E-012
NA12760	reg_CTRL	chr20	29567312	C	T	40	42,00%	17			+	HM13	rs6088441		1,00E-012
NA12760	reg_CTRL	chr20	29568468	A	G	41	44,00%	18			+	HM13	rs6059740		1,00E-012
NA12760	reg_CTRL	chr20	29570332	G	T	16	94,00%	15			+	HM13		rs57705377	1,00E-012
NA12760	reg_CTRL	chr20	29571922	T	C	29	17,00%	5			+	HM13		rs56917022	4,82E-004
NA12760	reg_CTRL	chr20	29571940	A	G	32	16,00%	5			+	HM13		rs12245561	7,72E-004
NA12760	reg_CTRL	chr20	29571953	T	A	32	16,00%	5			+	HM13			7,72E-004
NA12760	reg_CTRL	chr20	29573856	A	C	19	63,00%	12			+	HM13			1,00E-012
NA12760	reg_CTRL	chr20	29582163	G	A	32	59,00%	19			+	HM13	rs6088481		1,00E-012
NA12760	reg_CTRL	chr20	29591769	T	G	36	100,00%	36			+	HM13	rs1555285		1,00E-012
NA12760	reg_CTRL	chr20	29596516	T	A	33	48,00%	16			+	HM13			1,00E-012
NA12760	reg_CTRL	chr20	29614169	C	G	34	44,00%	15			+	HM13			1,00E-012
NA12760	reg_CTRL	chr8	6348637	A	C	41	51,00%	21			+	MCPH1	rs2979666		1,00E-012
NA12760	reg_CTRL	chr8	6349014	G	A	10	60,00%	6			+	MCPH1			2,87E-008
NA12760	reg_CTRL	chr8	6349033	A	G	10	50,00%	5			+	MCPH1	rs2515598		1,47E-006
NA12760	reg_CTRL	chr8	6349127	G	T	14	43,00%	6			+	MCPH1	rs11779671		3,79E-007
NA12760	reg_CTRL	chr8	6351021	T	A	18	61,00%	11			+	MCPH1	rs13249897		1,00E-012
NA12760	reg_CTRL	chr8	6351031	G	C	22	36,00%	8			+	MCPH1	rs17077194		1,88E-008
NA12760	reg_CTRL	chr8	6351326	A	G	47	53,00%	25			+	MCPH1		rs73199028	1,00E-012
NA12760	reg_CTRL	chr8	6351358	C	G	47	57,00%	27			+	MCPH1	rs2442468		1,00E-012
NA12760	reg_CTRL	chr8	6351983	C	T	30	60,00%	18			+	MCPH1	rs2442467		1,00E-012
NA12760	reg_CTRL	chr8	6352330	T	C	27	44,00%	12			+	MCPH1	rs2515409		1,00E-012
NA12760	reg_CTRL	chr8	6352755	G	A	16	50,00%	8			+	MCPH1	rs2515411		8,62E-010
NA12760	reg_CTRL	chr8	6352770	C	T	16	50,00%	8			+	MCPH1	rs2515412		8,62E-010
NA12760	reg_CTRL	chr8	6353028	T	C	29	41,00%	12			+	MCPH1	rs2515413		1,00E-012
NA12760	reg_CTRL	chr8	6353146	C	A	23	48,00%	11			+	MCPH1	rs2515414		1,66E-012
NA12760	reg_CTRL	chr8	6353242	C	G	23	43,00%	10			+	MCPH1	rs2442466		3,67E-011
NA12760	reg_CTRL	chr8	6353746	G	A	64	56,00%	36			+	MCPH1	rs2515416		1,00E-012
NA12760	reg_CTRL	chr8	6355043	C	T	37	68,00%	25			+	MCPH1	rs6559165		1,00E-012
NA12760	reg_CTRL	chr8	6355246	C	G	38	53,00%	20			+	MCPH1	rs6559166		1,00E-012
NA12760	reg_CTRL	chr8	6357304	G	A	54	56,00%	30			+	MCPH1	rs3780088		1,00E-012
NA12760	reg_CTRL	chr8	6357957	C	G	89	45,00%	40			+	MCPH1	rs7013006		1,00E-012
NA12760	reg_CTRL	chr8	6360058	T	A	31	45,00%	14			+	MCPH1	rs7838691		1,00E-012
NA12760	reg_CTRL	chr8	6361113	T	G	66	58,00%	38			+	MCPH1	rs4276711		1,00E-012
NA12760	reg_CTRL	chr8	6362181	T	C	57	40,00%	23			+	MCPH1	rs2515432		1,00E-012
NA12760	reg_CTRL	chr8	6362697	G	A	43	51,00%	22			+	MCPH1		rs73199039	1,00E-012
NA12760	reg_CTRL	chr8	6366192	C	A	57	47,00%	27	T	T	-3	ANGPT2		rs55633437	1,00E-012
NA12760	reg_CTRL	chr8	6366992	A	T	29	38,00%	11			+	MCPH1		rs56405992	4,26E-011
NA12760	reg_CTRL	chr8	6373087	T	A	26	42,00%	11			+	MCPH1	rs2442610		9,51E-012
NA12760	reg_CTRL	chr8	6373270	G	C	41	59,00%	24			+	MCPH1	rs2515465		1,00E-012
NA12760	reg_CTRL	chr8	6373518	C	T	60	53,00%	32			+	MCPH1			1,00E-012
NA12760	reg_CTRL	chr8	6373535	G	C	61	41,00%	25			+	MCPH1	rs2922887		1,00E-012
NA12760	reg_CTRL	chr8	6373658	T	C	57	46,00%	26			+	MCPH1	rs3824310		1,00E-012
NA12760	reg_CTRL	chr8	6373694	A	G	56	43,00%	24			+	MCPH1	rs2515466		1,00E-012
NA12760	reg_CTRL	chr8	6373849	G	A	59	58,00%	34			+	MCPH1	rs3824312		1,00E-012
NA12760	reg_CTRL	chr8	6373992	G	C	76	49,00%	37			+	MCPH1	rs2922886		1,00E-012
NA12760	reg_CTRL	chr8	6374001	T	C	79	48,00%	38			+	MCPH1	rs2442609		1,00E-012
NA12760	reg_CTRL	chr8	6374007	T	G	77	35,00%	27			+	MCPH1	rs2515469		1,00E-012
NA12760	reg_CTRL	chr8	6374028	T	C	77	44,00%	34			+	MCPH1	rs2442608		1,00E-012
NA12760	reg_CTRL	chr8	6374155	T	A	74	96,00%	71			+	MCPH1	rs1868554		1,00E-012

NA12760	reg_CTRL	chr8	6374450	T	C	20	55,00%	11			+	MCPH1	rs13268979	1,00E-012
NA12760	reg_CTRL	chr8	6374526	T	C	15	100,00%	15			+	MCPH1	rs1868552	1,00E-012
NA12760	reg_CTRL	chr8	6374645	C	T	20	50,00%	10			+	MCPH1	rs35735391	9,66E-012
NA12760	reg_CTRL	chr8	6374772	G	A	36	44,00%	16			+	MCPH1		1,00E-012
NA12760	reg_CTRL	chr8	6374964	T	C	37	41,00%	15			+	MCPH1	rs1807209	1,00E-012
NA12760	reg_CTRL	chr8	6375253	G	A	15	100,00%	15			+	MCPH1	rs2256628	1,00E-012
NA12760	reg_CTRL	chr8	6375399	C	T	6	100,00%	6			+	MCPH1	rs2515473	1,48E-010
NA12760	reg_CTRL	chr8	6375563	A	G	21	95,00%	20			+	MCPH1	rs734703	1,00E-012
NA12760	reg_CTRL	chr8	6375592	G	A	25	100,00%	25			+	MCPH1	rs746073	1,00E-012
NA12760	reg_CTRL	chr8	6375621	T	C	28	100,00%	28			+	MCPH1	rs734702	1,00E-012
NA12760	reg_CTRL	chr8	6375655	G	A	34	100,00%	34			+	MCPH1	rs734701	1,00E-012
NA12760	reg_CTRL	chr8	6375714	A	G	39	100,00%	39			+	MCPH1	rs734704	1,00E-012
NA12760	reg_CTRL	chr8	6375941	T	C	39	44,00%	17			+	MCPH1	rs2442604	1,00E-012
NA12760	reg_CTRL	chr8	6376190	G	C	26	100,00%	26			+	MCPH1	rs10503371	1,00E-012
NA12760	reg_CTRL	chr8	6376528	G	T	42	55,00%	23			+	MCPH1	rs2515479	1,00E-012
NA12760	reg_CTRL	chr8	6376624	T	G	45	100,00%	45			+	MCPH1	rs12674822	1,00E-012
NA12760	reg_CTRL	chr8	6376688	G	A	46	48,00%	22			+	MCPH1	rs2515480	1,00E-012
NA12760	reg_CTRL	chr8	6376791	A	T	53	47,00%	25			+	MCPH1	rs1984860	1,00E-012
NA12760	reg_CTRL	chr8	6376832	T	C	43	44,00%	19			+	MCPH1	rs1984859	1,00E-012
NA12760	reg_CTRL	chr8	6377122	T	C	52	31,00%	16			+	MCPH1	rs1984857	1,00E-012
NA12760	reg_CTRL	chr8	6377297	C	A	44	57,00%	25	A	A	-1	ANGPT2	rs6559167	1,00E-012
NA12760	reg_CTRL	chr8	6377435	A	G	38	47,00%	18			+	MCPH1	rs2515481	1,00E-012
NA12760	reg_CTRL	chr8	6377487	G	A	37	100,00%	37			+	MCPH1	rs2515482	1,00E-012
NA12760	reg_CTRL	chr8	6377745	G	T	57	100,00%	57			+	MCPH1	rs1031303	1,00E-012
NA12760	reg_CTRL	chr8	6377938	A	G	61	57,00%	35			+	MCPH1	rs17077419	1,00E-012
NA12760	reg_CTRL	chr8	6378159	T	C	59	98,00%	58			+	MCPH1	rs2442602	1,00E-012
NA12760	reg_CTRL	chr8	6378710	A	G	32	100,00%	32			+	MCPH1	rs2959812	1,00E-012
NA12760	reg_CTRL	chr8	6378974	C	A	18	39,00%	7			+	MCPH1	rs2959811	8,67E-008
NA12760	reg_CTRL	chr8	6379175	T	C	20	100,00%	20			+	MCPH1	rs2922881	1,00E-012
NA12760	reg_CTRL	chr8	6379358	C	G	26	62,00%	16			+	MCPH1	rs12550255	1,00E-012
NA12760	reg_CTRL	chr8	6379594	A	G	27	37,00%	10			+	MCPH1	rs12676103	2,51E-010
NA12760	reg_CTRL	chr8	6379677	G	C	37	51,00%	19			+	MCPH1	rs2515483	1,00E-012
NA12760	reg_CTRL	chr8	6379706	T	A	38	53,00%	20			+	MCPH1	rs17623064	1,00E-012
NA12760	reg_CTRL	chr8	6379955	C	T	62	45,00%	28			+	MCPH1	rs4841224	1,00E-012
NA12760	reg_CTRL	chr8	6381188	G	T	37	43,00%	16			+	MCPH1	rs2959809	1,00E-012
NA12760	reg_CTRL	chr8	6381373	G	A	60	50,00%	30			+	MCPH1	rs2442600	1,00E-012
NA12760	reg_CTRL	chr8	6381559	G	A	71	100,00%	71			+	MCPH1	rs2442599	1,00E-012
NA12760	reg_CTRL	chr8	6381587	A	G	68	49,00%	33			+	MCPH1	rs17552444	1,00E-012
NA12760	reg_CTRL	chr8	6382229	T	C	35	100,00%	35			+	MCPH1	rs2442598	1,00E-012
NA12760	reg_CTRL	chr8	6382284	T	G	30	60,00%	18			+	MCPH1	rs12541780	1,00E-012
NA12760	reg_CTRL	chr8	6382769	T	C	7	57,00%	4			+	MCPH1	rs17077452	9,26E-006
NA12760	reg_CTRL	chr8	6382971	T	A	12	92,00%	11			+	MCPH1	rs2959808	1,00E-012
NA12760	reg_CTRL	chr8	6383165	C	T	26	38,00%	10			+	MCPH1	rs12549309	1,61E-010
NA12760	reg_CTRL	chr8	6383317	G	A	39	54,00%	21			+	MCPH1	rs11989215	1,00E-012
NA12760	reg_CTRL	chr8	6383428	G	A	47	51,00%	24			+	MCPH1	rs11989242	1,00E-012
NA12760	reg_CTRL	chr8	6383590	A	C	47	49,00%	23			+	MCPH1	rs11137037	1,00E-012
NA12760	reg_CTRL	chr8	6383649	A	G	49	45,00%	22			+	MCPH1	rs10092206	1,00E-012
NA12760	reg_CTRL	chr8	6383749	C	T	43	56,00%	24			+	MCPH1	rs17623313	1,00E-012
NA12760	reg_CTRL	chr8	6384104	C	T	47	60,00%	28			+	MCPH1	rs2922876	1,00E-012
NA12760	reg_CTRL	chr8	6384278	G	A	44	43,00%	19			+	MCPH1	rs1375668	1,00E-012
NA12760	reg_CTRL	chr8	6384313	G	A	40	45,00%	18			+	MCPH1	rs1989321	1,00E-012
NA12760	reg_CTRL	chr8	6384394	G	T	31	39,00%	12			+	MCPH1	rs2897911	4,09E-012
NA12760	reg_CTRL	chr8	6384406	G	C	31	42,00%	13			+	MCPH1	rs1823375	1,00E-012

NA12760	reg_CTRL	chr8	6384513	C	A	21	95,00%	20	+	MCPH1	rs1823376			1,00E-012
NA12760	reg_CTRL	chr8	6384555	T	G	20	35,00%	7	+	MCPH1	rs2408341			2,03E-007
NA12760	reg_CTRL	chr8	6384627	C	T	17	41,00%	7	+	MCPH1	rs4263789			5,41E-008
NA12760	reg_CTRL	chr8	6384716	A	T	17	35,00%	6	+	MCPH1	rs4376511			1,47E-006
NA12760	reg_CTRL	chr8	6384838	A	T	33	48,00%	16	+	MCPH1	rs4455855			1,00E-012
NA12760	reg_CTRL	chr8	6384861	C	T	38	47,00%	18	+	MCPH1	rs4991608			1,00E-012
NA12760	reg_CTRL	chr8	6385378	C	T	65	52,00%	34	+	MCPH1	rs2922875			1,00E-012
NA12760	reg_CTRL	chr8	6385578	A	G	39	59,00%	23	+	MCPH1	rs2922874			1,00E-012
NA12760	reg_CTRL	chr8	6385735	A	G	28	100,00%	28	+	MCPH1	rs2922873			1,00E-012
NA12760	reg_CTRL	chr8	6385772	G	A	28	57,00%	16	+	MCPH1	rs2922872			1,00E-012
NA12760	reg_CTRL	chr8	6385878	A	T	22	55,00%	12	+	MCPH1				1,00E-012
NA12760	reg_CTRL	chr8	6385973	T	C	13	100,00%	13	+	MCPH1	rs2922871			1,00E-012
NA12760	reg_CTRL	chr8	6386005	G	A	14	57,00%	8	+	MCPH1	rs2959819			2,09E-010
NA12760	reg_CTRL	chr8	6386346	G	T	32	100,00%	32	+	MCPH1	rs2959820			1,00E-012
NA12760	reg_CTRL	chr8	6387008	A	G	32	50,00%	16	+	MCPH1	rs17077465			1,00E-012
NA12760	reg_CTRL	chr8	6387201	T	C	29	45,00%	13	+	MCPH1	rs2922869			1,00E-012
NA12760	reg_CTRL	chr8	6387274	T	G	25	96,00%	24	+	MCPH1	rs35742902			1,00E-012
NA12760	reg_CTRL	chr8	6387413	G	A	25	36,00%	9	+	MCPH1	rs1988762			2,64E-009
NA12760	reg_CTRL	chr8	6388961	T	C	65	58,00%	38	+	MCPH1				1,00E-012
NA12760	reg_CTRL	chr8	6389049	C	T	65	97,00%	63	+	MCPH1	rs2442597			1,00E-012
NA12760	reg_CTRL	chr8	6389774	T	C	78	58,00%	45	+	MCPH1	rs4478599	rs13250248		1,00E-012
NA12760	reg_CTRL	chr8	6390079	A	G	68	53,00%	36	+	MCPH1				1,00E-012
NA12760	reg_CTRL	chr8	6390296	A	C	58	47,00%	27	+	MCPH1	rs7005658			1,00E-012
NA12760	reg_CTRL	chr8	6390400	A	G	68	99,00%	67	+	MCPH1	rs2442596			1,00E-012
NA12760	reg_CTRL	chr8	6390414	A	C	70	100,00%	70	+	MCPH1	rs2515488			1,00E-012
NA12760	reg_CTRL	chr8	6391248	T	C	55	100,00%	55	+	MCPH1	rs2044744			1,00E-012
NA12760	reg_CTRL	chr8	6391663	G	C	52	100,00%	52	+	MCPH1	rs2515489			1,00E-012
NA12760	reg_CTRL	chr8	6391722	A	G	50	98,00%	49	+	MCPH1	rs2442595			1,00E-012
NA12760	reg_CTRL	chr8	6392028	A	T	53	100,00%	53	+	MCPH1	rs2442594			1,00E-012
NA12760	reg_CTRL	chr8	6392149	T	C	48	100,00%	48	+	MCPH1	rs2442593			1,00E-012
NA12760	reg_CTRL	chr8	6392271	T	C	37	97,00%	36	+	MCPH1	rs2515490			1,00E-012
NA12760	reg_CTRL	chr8	6393016	C	T	36	100,00%	36	+	MCPH1	rs2442592			1,00E-012
NA12760	reg_CTRL	chr8	6393121	G	A	35	100,00%	35	+	MCPH1	rs2515492			1,00E-012
NA12760	reg_CTRL	chr8	6393980	C	A	61	100,00%	61	+	MCPH1	rs2515493			1,00E-012
NA12760	reg_CTRL	chr8	6394533	T	C	17	100,00%	17	+	MCPH1	rs2515494			1,00E-012
NA12760	reg_CTRL	chr8	6397787	C	T	46	98,00%	45	+	MCPH1	rs2515497			1,00E-012
NA12760	reg_CTRL	chr8	6399673	G	C	45	56,00%	25	+	MCPH1	rs2515499			1,00E-012
NA12760	reg_CTRL	chr8	6399828	G	C	56	98,00%	55	+	MCPH1	rs2515500			1,00E-012
NA12760	reg_CTRL	chr8	6400033	C	T	59	54,00%	32	+	MCPH1	rs2515501			1,00E-012
NA12760	reg_CTRL	chr8	6400140	G	A	48	98,00%	47	+	MCPH1	rs2515502			1,00E-012
NA12760	reg_CTRL	chr8	6400377	A	G	15	93,00%	14	+	MCPH1	rs2922883			1,00E-012
NA12760	reg_CTRL	chr8	6400446	T	C	16	94,00%	15	+	MCPH1	rs2515503			1,00E-012
NA12760	reg_CTRL	chr8	6401634	C	T	21	100,00%	21	+	MCPH1	rs2515504			1,00E-012
NA12760	reg_CTRL	chr8	6404255	A	G	26	96,00%	25	+	MCPH1	rs2515505			1,00E-012
NA12760	reg_CTRL	chr8	6406668	T	G	39	97,00%	38	+	MCPH1	rs2515506			1,00E-012
NA12760	reg_CTRL	chr8	6407942	A	G	45	96,00%	43	+	MCPH1	rs3739391			1,00E-012
NA12760	reg_CTRL	chr8	6653493	G	A	6	67,00%	4	-	XKR5	rs9693931			4,04E-006
NA12760	reg_CTRL	chr8	6653898	G	A	48	46,00%	22	-	XKR5	rs17078203			1,00E-012
NA12760	reg_CTRL	chr8	6653994	A	G	38	45,00%	17	-	XKR5	rs9314611			1,00E-012
NA12760	reg_CTRL	chr8	6657359	T	C	62	50,00%	31	-	XKR5	rs2553728			1,00E-012
NA12760	reg_CTRL	chr8	6661868	A	G	34	59,00%	20	-	XKR5	rs9773025			1,00E-012
NA12760	reg_CTRL	chr8	6664775	C	T	22	27,00%	6	-	XKR5			rs72107959	8,04E-006
NA12760	reg_CTRL	chr8	6665852	T	A	40	42,00%	17	-	XKR5	rs13259050			1,00E-012

NA12760	reg_CTRL	chr8	6666726	C	A	60	37,00%	22			-	XKR5			rs58387909	1,00E-012
NA12760	reg_CTRL	chr8	6667128	A	T	31	52,00%	16			-	XKR5	rs28564455			1,00E-012
NA12760	reg_CTRL	chr8	6667206	C	G	44	52,00%	23			-	XKR5	rs9774066			1,00E-012
NA12760	reg_CTRL	chr8	6667776	A	T	71	51,00%	36			-	XKR5	rs11137070			1,00E-012
NA12760	reg_CTRL	chr8	6668059	C	T	70	50,00%	35			-	XKR5	rs12678222			1,00E-012
NA12760	reg_CTRL	chr8	6668168	C	A	60	57,00%	34			-	XKR5	rs11137071			1,00E-012
NA12760	reg_CTRL	chr8	6668666	A	C	52	48,00%	25			-	XKR5	rs9772979			1,00E-012
NA12760	reg_CTRL	chr8	6671038	G	A	56	39,00%	22			-	XKR5			rs57045395	1,00E-012
NA12760	reg_CTRL	chr8	6671291	G	T	71	46,00%	33			-	XKR5	rs28578619			1,00E-012
NA12760	reg_CTRL	chr8	6671411	A	G	66	36,00%	24			-	XKR5	rs4841770			1,00E-012
NA12760	reg_CTRL	chr8	6672148	A	G	42	100,00%	42			-	XKR5	rs2980958			1,00E-012
NA12760	reg_CTRL	chr8	6672229	T	G	48	100,00%	48			-	XKR5	rs2980957			1,00E-012
NA12760	reg_CTRL	chr8	6672308	C	G	51	98,00%	50			-	XKR5	rs2980956			1,00E-012
NA12760	reg_CTRL	chr8	6674201	C	T	64	53,00%	34			-	XKR5	rs2741087			1,00E-012
NA12760	reg_CTRL	chr8	6675647	T	C	48	50,00%	24			-	XKR5	rs2741089			1,00E-012
NA12760	reg_CTRL	chr8	6675775	G	C	36	53,00%	19			-	XKR5	rs2978903			1,00E-012
NA12760	reg_CTRL	chr8	6676673	T	C	43	58,00%	25			-	XKR5	rs2741091			1,00E-012
NA12760	reg_CTRL	chr8	6677583	G	C	45	49,00%	22			-	XKR5	rs2978902			1,00E-012
NA12760	reg_CTRL	chr8	6677625	G	C	46	48,00%	22			-	XKR5	rs2978901			1,00E-012
NA12760	reg_CTRL	chr8	6677686	T	C	36	53,00%	19	M	V	-2	XKR5	rs2741098			1,00E-012
NA12760	reg_CTRL	chr8	6678708	T	C	18	39,00%	7			-	XKR5	rs2978900			8,67E-008
NA12760	reg_CTRL	chr8	6679008	C	G	18	44,00%	8			-	XKR5	rs2978899			2,79E-009
NA12760	reg_DEFA	chr8	6717829	A	G	49	100,00%	49			-	DEFB1	rs2980928			1,00E-012
NA12760	reg_DEFA	chr8	6717856	A	G	48	100,00%	48			-	DEFB1	rs2980927			1,00E-012
NA12760	reg_DEFA	chr8	6717956	T	C	53	100,00%	53			-	DEFB1	rs2977779			1,00E-012
NA12760	reg_DEFA	chr8	6717999	G	A	52	96,00%	50			-	DEFB1	rs2977778			1,00E-012
NA12760	reg_DEFA	chr8	6718012	T	G	57	96,00%	55			-	DEFB1	rs2980926			1,00E-012
NA12760	reg_DEFA	chr8	6718140	C	G	59	98,00%	58			-	DEFB1	rs2978872			1,00E-012
NA12760	reg_DEFA	chr8	6718172	G	A	63	97,00%	61			-	DEFB1	rs2977777			1,00E-012
NA12760	reg_DEFA	chr8	6718680	C	T	32	100,00%	32			-	DEFB1	rs2978870			1,00E-012
NA12760	reg_DEFA	chr8	6718808	T	A	24	100,00%	24			-	DEFB1	rs2977776			1,00E-012
NA12760	reg_DEFA	chr8	6718836	A	T	24	100,00%	24			-	DEFB1	rs2951854			1,00E-012
NA12760	reg_DEFA	chr8	6718897	G	A	24	96,00%	23			-	DEFB1	rs2741127			1,00E-012
NA12760	reg_DEFA	chr8	6718913	A	C	26	100,00%	26			-	DEFB1	rs2927345			1,00E-012
NA12760	reg_DEFA	chr8	6719066	C	T	33	24,00%	8			-	DEFB1	rs10528208			6,50E-007
NA12760	reg_DEFA	chr8	6719161	C	A	45	100,00%	45			-	DEFB1	rs2978869			1,00E-012
NA12760	reg_DEFA	chr8	6719186	T	C	49	100,00%	49			-	DEFB1	rs2977774			1,00E-012
NA12760	reg_DEFA	chr8	6719218	C	A	52	100,00%	52			-	DEFB1	rs2978868			1,00E-012
NA12760	reg_DEFA	chr8	6719403	A	G	41	100,00%	41			-	DEFB1	rs2980924			1,00E-012
NA12760	reg_DEFA	chr8	6719467	G	C	39	97,00%	38			-	DEFB1	rs5743465	rs34929240		1,00E-012
NA12760	reg_DEFA	chr8	6719468	T	A	39	100,00%	39			-	DEFB1	rs34929240	rs5743464		1,00E-012
NA12760	reg_DEFA	chr8	6719584	G	T	29	100,00%	29			-	DEFB1	rs5743463			1,00E-012
NA12760	reg_DEFA	chr8	6719596	A	G	28	100,00%	28			-	DEFB1	rs5743462			1,00E-012
NA12760	reg_DEFA	chr8	6719656	G	A	18	100,00%	18			-	DEFB1	rs2978867			1,00E-012
NA12760	reg_DEFA	chr8	6719740	C	T	13	100,00%	13			-	DEFB1	rs2978866			1,00E-012
NA12760	reg_DEFA	chr8	6720060	G	A	23	100,00%	23			-	DEFB1	rs2977773			1,00E-012
NA12760	reg_DEFA	chr8	6720108	T	C	34	100,00%	34			-	DEFB1	rs2951855			1,00E-012
NA12760	reg_DEFA	chr8	6720467	A	G	57	91,00%	52			-	DEFB1	rs2980923			1,00E-012
NA12760	reg_DEFA	chr8	6720606	G	A	65	100,00%	65			-	DEFB1	rs2977772			1,00E-012
NA12760	reg_DEFA	chr8	6720679	G	A	69	100,00%	69			-	DEFB1	rs2741129			1,00E-012
NA12760	reg_DEFA	chr8	6721130	T	C	32	100,00%	32			-	DEFB1	rs2978864	rs5743441		1,00E-012
NA12760	reg_DEFA	chr8	6721183	C	T	24	100,00%	24			-	DEFB1	rs5743440			1,00E-012
NA12760	reg_DEFA	chr8	6721210	C	A	20	90,00%	18			-	DEFB1	rs5743439			1,00E-012

NA12760	reg_DEFA	chr8	6721278	T	C	16	87,00%	14	-	DEFB1	rs5743437		1,00E-012
NA12760	reg_DEFA	chr8	6721755	A	C	12	50,00%	6	-	DEFB1	rs2980921		1,21E-007
NA12760	reg_DEFA	chr8	6722097	T	C	36	100,00%	36	-	DEFB1	rs2702945		1,00E-012
NA12760	reg_DEFA	chr8	6722258	G	C	23	96,00%	22	-	DEFB1	rs2293960		1,00E-012
NA12760	reg_DEFA	chr8	6722484	C	T	16	100,00%	16	-	DEFB1	rs2293959		1,00E-012
NA12760	reg_DEFA	chr8	6722710	T	A	19	100,00%	19	-	DEFB1	rs2293958		1,00E-012
NA12760	reg_DEFA	chr8	6722833	C	G	24	100,00%	24	-	DEFB1	rs1800972		1,00E-012
NA12760	reg_DEFA	chr8	6722841	C	T	24	92,00%	22	-	DEFB1	rs1799946		1,00E-012
NA12760	reg_DEFA	chr8	6723179	A	T	32	100,00%	32			rs2738182		1,00E-012
NA12760	reg_DEFA	chr8	6723399	C	T	19	100,00%	19			rs2741132		1,00E-012
NA12760	reg_DEFA	chr8	6723423	G	C	18	100,00%	18			rs2702876	rs7838626	1,00E-012
NA12760	reg_DEFA	chr8	6723465	G	T	17	100,00%	17			rs2741133		1,00E-012
NA12760	reg_DEFA	chr8	6723477	G	C	17	100,00%	17			rs2702877		1,00E-012
NA12760	reg_DEFA	chr8	6723483	T	C	18	100,00%	18			rs2977829		1,00E-012
NA12760	reg_DEFA	chr8	6723520	G	A	21	100,00%	21			rs2741134		1,00E-012
NA12760	reg_DEFA	chr8	6723531	C	T	22	100,00%	22			rs2978863		1,00E-012
NA12760	reg_DEFA	chr8	6723664	G	A	26	96,00%	25			rs2741135		1,00E-012
NA12760	reg_DEFA	chr8	6723906	A	T	19	100,00%	19			rs2738181		1,00E-012
NA12760	reg_DEFA	chr8	6723908	C	T	19	100,00%	19			rs2738180		1,00E-012
NA12760	reg_DEFA	chr8	6723927	T	G	18	100,00%	18			rs2738179		1,00E-012
NA12760	reg_DEFA	chr8	6723930	A	C	18	100,00%	18			rs2738178		1,00E-012
NA12760	reg_DEFA	chr8	6725291	G	C	26	100,00%	26			rs2702881		1,00E-012
NA12760	reg_DEFA	chr8	6725524	T	C	25	84,00%	21			rs2738177		1,00E-012
NA12760	reg_DEFA	chr8	6725600	G	T	20	100,00%	20			rs2741138		1,00E-012
NA12760	reg_DEFA	chr8	6725873	G	A	9	100,00%	9			rs11990152		1,00E-012
NA12760	reg_DEFA	chr8	6726157	A	G	6	100,00%	6			rs2741139		1,48E-010
NA12760	reg_DEFA	chr8	6726180	A	G	6	100,00%	6			rs2741140		1,48E-010
NA12760	reg_DEFA	chr8	6726302	T	C	12	100,00%	12			rs2702884		1,00E-012
NA12760	reg_DEFA	chr8	6726851	T	C	26	100,00%	26			rs2738175		1,00E-012
NA12760	reg_DEFA	chr8	6727011	G	A	47	96,00%	45			rs2741141		1,00E-012
NA12760	reg_DEFA	chr8	6727167	C	T	60	95,00%	57			rs2977827		1,00E-012
NA12760	reg_DEFA	chr8	6727332	G	A	73	99,00%	72			rs2978861		1,00E-012
NA12760	reg_DEFA	chr8	6727393	T	C	84	99,00%	83			rs2978860		1,00E-012
NA12760	reg_DEFA	chr8	6727484	C	T	76	100,00%	76			rs2977826		1,00E-012
NA12760	reg_DEFA	chr8	6727601	C	T	62	98,00%	61			rs2977825		1,00E-012
NA12760	reg_DEFA	chr8	6727818	G	A	11	100,00%	11			rs9692818	rs9694351	1,00E-012
NA12760	reg_DEFA	chr8	6727819	T	C	11	100,00%	11			rs9694351		1,00E-012
NA12760	reg_DEFA	chr8	6727833	A	C	12	92,00%	11			rs9694106		1,00E-012
NA12760	reg_DEFA	chr8	6727846	A	G	13	100,00%	13			rs9694107		1,00E-012
NA12760	reg_DEFA	chr8	6727902	A	G	29	100,00%	29			rs9694118		1,00E-012
NA12760	reg_DEFA	chr8	6728005	G	T	39	100,00%	39			rs2978859		1,00E-012
NA12760	reg_DEFA	chr8	6728154	C	T	36	100,00%	36			rs2977824		1,00E-012
NA12760	reg_DEFA	chr8	6728378	A	G	31	100,00%	31			rs2741143		1,00E-012
NA12760	reg_DEFA	chr8	6728397	C	T	29	100,00%	29			rs2738172		1,00E-012
NA12760	reg_DEFA	chr8	6728537	T	C	25	100,00%	25			rs2738171		1,00E-012
NA12760	reg_DEFA	chr8	6728715	T	G	19	100,00%	19			rs2978857		1,00E-012
NA12760	reg_DEFA	chr8	6728719	A	G	17	100,00%	17			rs2951840		1,00E-012
NA12760	reg_DEFA	chr8	6728764	C	T	13	85,00%	11			rs2980919		1,00E-012
NA12760	reg_DEFA	chr8	6728770	T	C	13	100,00%	13			rs2978856		1,00E-012
NA12760	reg_DEFA	chr8	6728867	G	C	11	82,00%	9			rs2978855		1,00E-012
NA12760	reg_DEFA	chr8	6729141	A	G	4	100,00%	4			rs2977823		2,80E-007
NA12760	reg_DEFA	chr8	6729213	C	A	4	100,00%	4			rs2977822		2,80E-007
NA12760	reg_DEFA	chr8	6729311	T	C	9	100,00%	9			rs2978853		1,00E-012

NA12760	reg_DEFA	chr8	6729458	C	T	19	95,00%	18	rs2977821			1,00E-012
NA12760	reg_DEFA	chr8	6729487	C	T	20	45,00%	9				2,44E-010
NA12760	reg_DEFA	chr8	6729520	C	T	25	100,00%	25	rs2951850			1,00E-012
NA12760	reg_DEFA	chr8	6729801	G	A	52	50,00%	26	rs2978852			1,00E-012
NA12760	reg_DEFA	chr8	6730978	A	G	20	100,00%	20	rs2738165			1,00E-012
NA12760	reg_DEFA	chr8	6731383	T	C	43	100,00%	43	rs2738164			1,00E-012
NA12760	reg_DEFA	chr8	6732626	C	T	41	98,00%	40	rs2738163			1,00E-012
NA12760	reg_DEFA	chr8	6734051	A	G	51	100,00%	51	rs2741718			1,00E-012
NA12760	reg_DEFA	chr8	6734149	G	A	53	96,00%	51	rs2978851			1,00E-012
NA12760	reg_DEFA	chr8	6735296	G	A	42	50,00%	21	rs2741717			1,00E-012
NA12760	reg_DEFA	chr8	6736007	C	T	39	100,00%	39	rs2738161			1,00E-012
NA12760	reg_DEFA	chr8	6736627	G	C	33	45,00%	15	rs2741149	rs10089905		1,00E-012
NA12760	reg_DEFA	chr8	6736973	G	A	32	47,00%	15	rs4841787			1,00E-012
NA12760	reg_DEFA	chr8	6737079	G	A	30	100,00%	30	rs2738158			1,00E-012
NA12760	reg_DEFA	chr8	6737740	T	G	30	40,00%	12				2,51E-012
NA12760	reg_DEFA	chr8	6737799	T	A	26	46,00%	12				1,00E-012
NA12760	reg_DEFA	chr8	6738298	A	G	11	91,00%	10	rs2978964			1,00E-012
NA12760	reg_DEFA	chr8	6738591	G	A	28	100,00%	28	rs2951870			1,00E-012
NA12760	reg_DEFA	chr8	6738844	T	C	47	100,00%	47	rs2702936			1,00E-012
NA12760	reg_DEFA	chr8	6739237	A	G	53	92,00%	49	rs2702935			1,00E-012
NA12760	reg_DEFA	chr8	6739310	T	G	52	85,00%	44	rs2738153			1,00E-012
NA12760	reg_DEFA	chr8	6739495	T	A	56	100,00%	56	rs2738152			1,00E-012
NA12760	reg_DEFA	chr8	6741958	C	A	37	97,00%	36	rs751009			1,00E-012
NA12760	reg_DEFA	chr8	6743931	T	C	8	100,00%	8	rs2980959			1,00E-012
NA12760	reg_DEFA	chr8	6743987	A	G	8	100,00%	8	rs13275881			1,00E-012
NA12760	reg_DEFA	chr8	6743992	G	C	8	100,00%	8	rs13272703			1,00E-012
NA12760	reg_DEFA	chr8	6744124	T	C	11	91,00%	10	rs13255674			1,00E-012
NA12760	reg_DEFA	chr8	6744173	A	C	15	100,00%	15	rs9693852			1,00E-012
NA12760	reg_DEFA	chr8	6744180	T	C	15	100,00%	15	rs13255719			1,00E-012
NA12760	reg_DEFA	chr8	6744212	G	A	17	100,00%	17	rs2741057			1,00E-012
NA12760	reg_DEFA	chr8	6745807	G	A	44	100,00%	44	rs2702929			1,00E-012
NA12760	reg_DEFA	chr8	6745889	A	C	42	100,00%	42	rs2702930			1,00E-012
NA12760	reg_DEFA	chr8	6746226	G	A	37	100,00%	37	rs2738143			1,00E-012
NA12760	reg_DEFA	chr8	6746266	A	T	31	100,00%	31	rs2741060			1,00E-012
NA12760	reg_DEFA	chr8	6746389	A	C	24	100,00%	24	rs2702931			1,00E-012
NA12760	reg_DEFA	chr8	6746793	T	C	9	100,00%	9	rs2702932			1,00E-012
NA12760	reg_DEFA	chr8	6746980	C	G	9	100,00%	9	rs2741061			1,00E-012
NA12760	reg_DEFA	chr8	6747103	C	G	11	100,00%	11	rs2741062			1,00E-012
NA12760	reg_DEFA	chr8	6747377	C	A	22	91,00%	20	rs2741714			1,00E-012
NA12760	reg_DEFA	chr8	6747535	A	C	27	100,00%	27	rs2951867			1,00E-012
NA12760	reg_DEFA	chr8	6747552	T	C	28	96,00%	27	rs2702933			1,00E-012
NA12760	reg_DEFA	chr8	6748150	G	C	47	17,00%	8				1,10E-005
NA12760	reg_DEFA	chr8	6750581	T	C	32	100,00%	32	rs2981405			1,00E-012
NA12760	reg_DEFA	chr8	6750651	A	G	40	100,00%	40	rs2741073			1,00E-012
NA12760	reg_DEFA	chr8	6751207	C	T	49	100,00%	49	rs2741075			1,00E-012
NA12760	reg_DEFA	chr8	6751396	T	C	43	100,00%	43	rs2741710			1,00E-012
NA12760	reg_DEFA	chr8	6754935	T	C	53	51,00%	27			rs73194190	1,00E-012
NA12760	reg_DEFA	chr8	6757796	A	G	33	97,00%	32	rs10216819			1,00E-012
NA12760	reg_DEFA	chr8	6758791	T	C	23	100,00%	23	rs2981401			1,00E-012
NA12760	reg_DEFA	chr8	6758869	C	T	22	45,00%	10				3,38E-011
NA12760	reg_DEFA	chr8	6760323	T	G	38	97,00%	37	rs7461956			1,00E-012
NA12760	reg_DEFA	chr8	6761967	G	A	71	100,00%	71	rs35200859			1,00E-012
NA12760	reg_DEFA	chr8	6762964	G	A	46	98,00%	45	rs2741699			1,00E-012

NA12760	reg_DEFA	chr8	6762978	C	A	44	82,00%	36			rs2741698			1,00E-012
NA12760	reg_DEFA	chr8	6763722	G	A	49	96,00%	47			rs2741695			1,00E-012
NA12760	reg_DEFA	chr8	6764461	A	G	35	97,00%	34			rs2741694			1,00E-012
NA12760	reg_DEFA	chr8	6764855	C	T	44	52,00%	23						1,00E-012
NA12760	reg_DEFA	chr8	6765363	G	A	22	100,00%	22			rs2702946			1,00E-012
NA12760	reg_DEFA	chr8	6765676	A	G	22	91,00%	20			rs12545953			1,00E-012
NA12760	reg_DEFA	chr8	6765994	T	C	30	97,00%	29			rs2738129			1,00E-012
NA12760	reg_DEFA	chr8	6766076	A	C	30	100,00%	30			rs2738128			1,00E-012
NA12760	reg_DEFA	chr8	6766524	T	C	31	100,00%	31			rs2978959			1,00E-012
NA12760	reg_DEFA	chr8	6766756	T	A	24	96,00%	23			rs13265468			1,00E-012
NA12760	reg_DEFA	chr8	6767209	C	T	31	100,00%	31			rs4294209			1,00E-012
NA12760	reg_DEFA	chr8	6767654	T	C	32	97,00%	31			rs13275170			1,00E-012
NA12760	reg_DEFA	chr8	6767778	G	T	20	100,00%	20			rs2738125			1,00E-012
NA12760	reg_DEFA	chr8	6767996	T	C	4	100,00%	4			rs13276112			2,80E-007
NA12760	reg_DEFA	chr8	6768647	T	A	19	95,00%	18			rs2702905			1,00E-012
NA12760	reg_DEFA	chr8	6768913	G	A	21	100,00%	21			rs7826006			1,00E-012
NA12760	reg_DEFA	chr8	6769030	T	C	17	100,00%	17			rs2738122			1,00E-012
NA12760	reg_DEFA	chr8	6769096	T	C	15	100,00%	15			rs2738121			1,00E-012
NA12760	reg_DEFA	chr8	6769372	G	A	39	90,00%	35			rs3888152			1,00E-012
NA12760	reg_DEFA	chr8	6770046	G	C	77	96,00%	74	-	DEFA6	rs2738120			1,00E-012
NA12760	reg_DEFA	chr8	6770127	G	C	57	96,00%	55	-	DEFA6	rs2738119			1,00E-012
NA12760	reg_DEFA	chr8	6770376	G	T	27	100,00%	27	-	DEFA6	rs2741691			1,00E-012
NA12760	reg_DEFA	chr8	6771046	G	T	40	100,00%	40			rs11784359			1,00E-012
NA12760	reg_DEFA	chr8	6771370	C	G	40	100,00%	40			rs4458901			1,00E-012
NA12760	reg_DEFA	chr8	6771627	C	G	30	100,00%	30			rs2741690			1,00E-012
NA12760	reg_DEFA	chr8	6771666	C	T	27	100,00%	27			rs2741689			1,00E-012
NA12760	reg_DEFA	chr8	6772381	G	T	26	100,00%	26			rs2738118			1,00E-012
NA12760	reg_DEFA	chr8	6773483	G	C	59	100,00%	59			rs2741686			1,00E-012
NA12760	reg_DEFA	chr8	6774191	T	C	14	93,00%	13			rs3918350			1,00E-012
NA12760	reg_DEFA	chr8	6774412	C	G	11	100,00%	11			rs34502430			1,00E-012
NA12760	reg_DEFA	chr8	6774485	T	C	9	100,00%	9			rs2702938			1,00E-012
NA12760	reg_DEFA	chr8	6774810	T	C	13	92,00%	12			rs2702939			1,00E-012
NA12760	reg_DEFA	chr8	6775470	G	A	20	95,00%	19			rs2012832	rs2738114		1,00E-012
NA12760	reg_DEFA	chr8	6775828	C	G	28	96,00%	27			rs2741684			1,00E-012
NA12760	reg_DEFA	chr8	6775890	T	C	30	100,00%	30			rs2741683			1,00E-012
NA12760	reg_DEFA	chr8	6776054	A	G	24	100,00%	24			rs2738111			1,00E-012
NA12760	reg_DEFA	chr8	6776610	T	C	35	97,00%	34			rs2702855			1,00E-012
NA12760	reg_DEFA	chr8	6776698	C	G	40	52,00%	21			rs3887306			1,00E-012
NA12760	reg_DEFA	chr8	6777064	A	T	30	100,00%	30			rs2738109			1,00E-012
NA12760	reg_DEFA	chr8	6777196	T	C	42	100,00%	42			rs2702858			1,00E-012
NA12760	reg_DEFA	chr8	6777240	G	A	42	69,00%	29			rs2977818			1,00E-012
NA12760	reg_DEFA	chr8	6777294	G	A	45	58,00%	26			rs13274891			1,00E-012
NA12760	reg_DEFA	chr8	6777394	T	C	47	57,00%	27			rs13250769			1,00E-012
NA12760	reg_DEFA	chr8	6777652	C	T	49	33,00%	16			rs2702860			1,00E-012
NA12760	reg_DEFA	chr8	6777734	T	C	55	91,00%	50			rs2702861			1,00E-012
NA12760	reg_DEFA	chr8	6777846	G	A	64	41,00%	26						1,00E-012
NA12760	reg_DEFA	chr8	6777962	C	T	57	56,00%	32					rs56248548	1,00E-012
NA12760	reg_DEFA	chr8	6778120	C	A	27	52,00%	14			rs13251447			1,00E-012
NA12760	reg_DEFA	chr8	6778219	T	C	6	100,00%	6			rs2741682			1,48E-010
NA12760	reg_DEFA	chr8	6779011	A	G	22	36,00%	8			rs13262140			1,88E-008
NA12760	reg_DEFA	chr8	6779125	A	G	24	100,00%	24			rs2738106			1,00E-012
NA12760	reg_DEFA	chr8	6779205	C	T	25	48,00%	12						1,00E-012
NA12760	reg_DEFA	chr8	6779250	C	T	30	43,00%	13			rs13261705			1,00E-012

NA12760	reg_DEFA	chr8	6779667	G	A	32	100,00%	32					rs2738104		1,00E-012
NA12760	reg_DEFA	chr8	6779811	C	T	32	53,00%	17					rs13263461		1,00E-012
NA12760	reg_DEFA	chr8	6779861	C	G	29	52,00%	15					rs13263510		1,00E-012
NA12760	reg_DEFA	chr8	6779930	G	A	33	91,00%	30					rs2738103		1,00E-012
NA12760	reg_DEFA	chr8	6779979	C	T	36	33,00%	12					rs2702866		3,63E-011
NA12760	reg_DEFA	chr8	6780050	A	G	45	64,00%	29					rs13254588		1,00E-012
NA12760	reg_DEFA	chr8	6780152	G	T	57	54,00%	31					rs13251814		1,00E-012
NA12760	reg_DEFA	chr8	6780481	G	A	71	100,00%	71					rs2738102		1,00E-012
NA12760	reg_DEFA	chr8	6780732	T	C	67	94,00%	63					rs2702867		1,00E-012
NA12760	reg_DEFA	chr8	6780950	C	T	68	96,00%	65			-	DEFA4	rs736227		1,00E-012
NA12760	reg_DEFA	chr8	6780991	A	G	63	46,00%	29	G	G	-2	DEFA4	rs2738100		1,00E-012
NA12760	reg_DEFA	chr8	6781429	G	A	48	100,00%	48			-	DEFA4	rs2239668		1,00E-012
NA12760	reg_DEFA	chr8	6781617	G	A	41	63,00%	26			-	DEFA4	rs2239667		1,00E-012
NA12760	reg_DEFA	chr8	6781843	C	T	21	57,00%	12			-	DEFA4		rs56007429	1,00E-012
NA12760	reg_DEFA	chr8	6782277	T	A	47	100,00%	47			-	DEFA4	rs2741679		1,00E-012
NA12760	reg_DEFA	chr8	6782499	G	A	67	51,00%	34			-	DEFA4	rs2741678		1,00E-012
NA12760	reg_DEFA	chr8	6783452	A	G	34	44,00%	15					rs2738098		1,00E-012
NA12760	reg_DEFA	chr8	6783611	C	T	34	56,00%	19					rs2741676		1,00E-012
NA12760	reg_DEFA	chr8	6784021	C	T	26	35,00%	9					rs45482601		3,95E-009
NA12760	reg_DEFA	chr8	6784715	G	A	40	50,00%	20							1,00E-012
NA12760	reg_DEFA	chr8	6784942	C	A	51	98,00%	50					rs2741675		1,00E-012
NA12760	reg_DEFA	chr8	6785122	C	T	43	42,00%	18					rs2741674		1,00E-012
NA12760	reg_DEFA	chr8	6785597	G	A	68	50,00%	34					rs3890000		1,00E-012
NA12760	reg_DEFA	chr8	6786255	G	A	61	51,00%	31							1,00E-012
NA12760	reg_DEFA	chr8	6786308	C	G	53	49,00%	26					rs2615772		1,00E-012
NA12760	reg_DEFA	chr8	6786381	C	A	44	48,00%	21					rs2741673		1,00E-012
NA12760	reg_DEFA	chr8	6786918	G	T	35	51,00%	18					rs11991138		1,00E-012
NA12760	reg_DEFA	chr8	6786956	G	C	35	54,00%	19							1,00E-012
NA12760	reg_DEFA	chr8	6787913	C	T	14	57,00%	8					rs34431957		2,09E-010
NA12760	reg_DEFA	chr8	6788129	C	G	17	53,00%	9					rs2951853		5,40E-011
NA12760	reg_DEFA	chr8	6788410	C	T	25	60,00%	15					rs13263953		1,00E-012
NA12760	reg_DEFA	chr8	6788432	C	T	25	56,00%	14					rs13263967		1,00E-012
NA12760	reg_DEFA	chr8	6789122	A	G	28	54,00%	15					rs2981398		1,00E-012
NA12760	reg_DEFA	chr8	6789483	T	C	21	43,00%	9					rs2741669		4,19E-010
NA12760	reg_DEFA	chr8	6789749	G	T	42	45,00%	19					rs35900031		1,00E-012
NA12760	reg_DEFA	chr8	6790937	T	C	52	58,00%	30					rs2741668		1,00E-012
NA12760	reg_DEFA	chr8	6791083	C	A	43	33,00%	14							1,00E-012
NA12760	reg_DEFA	chr8	6791260	C	T	49	57,00%	28					rs2981396		1,00E-012
NA12760	reg_DEFA	chr8	6792879	A	G	54	100,00%	54					rs2951844		1,00E-012
NA12760	reg_DEFA	chr8	6793624	A	G	49	100,00%	49					rs2702879		1,00E-012
NA12760	reg_DEFA	chr8	6793699	C	T	42	38,00%	16					rs2741665		1,00E-012
NA12760	reg_DEFA	chr8	6793802	C	T	34	50,00%	17							1,00E-012
NA12760	reg_DEFA	chr8	6794668	A	G	35	51,00%	18					rs7016650		1,00E-012
NA12760	reg_DEFA	chr8	6794720	T	G	35	54,00%	19					rs2978955		1,00E-012
NA12760	reg_DEFA	chr8	6795249	C	T	56	41,00%	23					rs4840645		1,00E-012
NA12760	reg_DEFA	chr8	6795351	A	G	46	37,00%	17					rs4840646		1,00E-012
NA12760	reg_DEFA	chr8	6795430	A	T	43	51,00%	22					rs2741661		1,00E-012
NA12760	reg_DEFA	chr8	6795791	T	A	45	20,00%	9					rs6998500		7,54E-007
NA12760	reg_DEFA	chr8	6796132	T	G	54	56,00%	30					rs2075836		1,00E-012
NA12760	reg_DEFA	chr8	6796298	C	T	55	42,00%	23					rs2075835		1,00E-012
NA12760	reg_DEFA	chr8	6797176	C	G	67	52,00%	35							1,00E-012
NA12760	reg_DEFA	chr8	6797225	T	C	69	49,00%	34					rs35237181		1,00E-012
NA12760	reg_DEFA	chr8	6797578	T	C	43	49,00%	21					rs2741659		1,00E-012

NA12760	reg_DEFA	chr8	6797707	C	T	37	46,00%	17			rs4841789		1,00E-012
NA12760	reg_DEFA	chr8	6798559	T	G	35	57,00%	20			rs2615787		1,00E-012
NA12760	reg_DEFA	chr8	6798969	C	T	53	51,00%	27			rs2741658		1,00E-012
NA12760	reg_DEFA	chr8	6799608	A	G	31	100,00%	31			rs2741657		1,00E-012
NA12760	reg_DEFA	chr8	6799659	G	T	30	100,00%	30			rs2472562		1,00E-012
NA12760	reg_DEFA	chr8	6800090	G	T	28	46,00%	13				rs35000792	1,00E-012
NA12760	reg_DEFA	chr8	6800169	A	G	40	50,00%	20			rs2615771		1,00E-012
NA12760	reg_DEFA	chr8	6800911	T	G	41	34,00%	14			rs2702852		1,00E-012
NA12760	reg_DEFA	chr8	6805553	T	C	43	56,00%	24			rs4322031		1,00E-012
NA12760	reg_DEFA	chr8	6806978	A	G	11	36,00%	4				rs57688776	8,11E-005
NA12760	reg_DEFA	chr8	6807665	T	C	24	29,00%	7					8,35E-007
NA12760	reg_DEFA	chr8	6808829	T	C	69	100,00%	69			rs2977793		1,00E-012
NA12760	reg_DEFA	chr8	6809027	T	C	76	97,00%	74			rs2738058		1,00E-012
NA12760	reg_DEFA	chr8	6809450	G	A	69	99,00%	68			rs2951869		1,00E-012
NA12760	reg_DEFA	chr8	6809904	T	C	27	93,00%	25			rs2977789		1,00E-012
NA12760	reg_DEFA	chr8	6809966	A	C	27	100,00%	27			rs2977788		1,00E-012
NA12760	reg_DEFA	chr8	6810016	C	T	27	26,00%	7			rs2615768		2,02E-006
NA12760	reg_DEFA	chr8	6810195	A	G	25	28,00%	7			rs2738048		1,14E-006
NA12760	reg_DEFA	chr8	6810705	A	G	54	96,00%	52			rs2978951		1,00E-012
NA12760	reg_DEFA	chr8	6810952	C	A	62	45,00%	28			rs2738046		1,00E-012
NA12760	reg_DEFA	chr8	6810975	C	G	63	44,00%	28			rs2702912		1,00E-012
NA12760	reg_DEFA	chr8	6810978	T	C	64	42,00%	27			rs2738045		1,00E-012
NA12760	reg_DEFA	chr8	6811301	G	A	63	43,00%	27			rs2702910		1,00E-012
NA12760	reg_DEFA	chr8	6811635	T	C	45	47,00%	21			rs2702909		1,00E-012
NA12760	reg_DEFA	chr8	6811987	G	T	25	48,00%	12			rs2738168		1,00E-012
NA12760	reg_DEFA	chr8	6812024	G	A	23	48,00%	11			rs2702908		1,66E-012
NA12760	reg_DEFA	chr8	6812694	G	C	67	54,00%	36			rs2978950		1,00E-012
NA12760	reg_DEFA	chr8	6812705	G	A	66	44,00%	29			rs6996047		1,00E-012
NA12760	reg_DEFA	chr8	6812906	G	C	59	51,00%	30			rs11996346		1,00E-012
NA12760	reg_DEFA	chr8	6813499	A	G	76	97,00%	74	-	DEFA10P	rs2978947		1,00E-012
NA12760	reg_DEFA	chr8	6813654	T	C	63	48,00%	30	-	DEFA10P	rs2702875		1,00E-012
NA12760	reg_DEFA	chr8	6814126	A	G	40	40,00%	16			rs2978944		1,00E-012
NA12760	reg_DEFA	chr8	6814709	T	A	60	43,00%	26			rs2738135		1,00E-012
NA12760	reg_DEFA	chr8	6815234	G	A	40	37,00%	15			rs2738132		1,00E-012
NA12760	reg_DEFA	chr8	6816191	T	C	13	62,00%	8			rs12682063		9,13E-011
NA12760	reg_DEFA	chr8	6816201	T	C	14	57,00%	8			rs12682076		2,09E-010
NA12760	reg_DEFA	chr8	6816223	G	A	15	60,00%	9			rs12675298		1,11E-011
NA12760	reg_DEFA	chr8	6816300	T	C	13	54,00%	7			rs34914251		5,17E-009
NA12760	reg_DEFA	chr8	6816495	G	T	13	38,00%	5			rs2738113		7,10E-006
NA12760	reg_DEFA	chr8	6816971	G	A	44	27,00%	12					2,54E-010
NA12760	reg_DEFA	chr8	6816974	C	T	45	27,00%	12			rs6989424		3,12E-010
NA12760	reg_DEFA	chr8	6817024	G	A	61	33,00%	20			rs10095450		1,00E-012
NA12760	reg_DEFA	chr8	6817036	T	C	61	98,00%	60			rs2738101		1,00E-012
NA12760	reg_DEFA	chr8	6817114	C	T	95	44,00%	42			rs2615789		1,00E-012
NA12760	reg_DEFA	chr8	6817172	G	A	109	25,00%	27			rs10095611		1,00E-012
NA12760	reg_DEFA	chr8	6817394	C	G	80	30,00%	24			rs4841790		1,00E-012
NA12760	reg_DEFA	chr8	6817403	A	C	80	10,00%	8				rs57466375	5,21E-004
NA12760	reg_DEFA	chr8	6817418	C	T	76	43,00%	33			rs10096781		1,00E-012
NA12760	reg_DEFA	chr8	6817429	A	G	75	27,00%	20			rs2738096		1,00E-012
NA12760	reg_DEFA	chr8	6817438	G	A	75	27,00%	20			rs2978910		1,00E-012
NA12760	reg_DEFA	chr8	6817815	C	T	9	89,00%	8			rs4841791		1,00E-012
NA12760	reg_DEFA	chr8	6818268	C	T	23	87,00%	20					1,00E-012
NA12760	reg_DEFA	chr8	6818340	C	A	37	76,00%	28					1,00E-012

NA12760	reg_DEFA	chr8	6818375	G	A	49	98,00%	48			rs2738089			1,00E-012
NA12760	reg_DEFA	chr8	6818397	C	T	46	30,00%	14			rs2927351			3,56E-012
NA12760	reg_DEFA	chr8	6818577	T	C	61	34,00%	21			rs4082288			1,00E-012
NA12760	reg_DEFA	chr8	6818799	G	T	46	33,00%	15			rs2738083			1,00E-012
NA12760	reg_DEFA	chr8	6818923	C	T	33	42,00%	14						1,00E-012
NA12760	reg_DEFA	chr8	6819028	A	C	24	100,00%	24			rs2978905			1,00E-012
NA12760	reg_DEFA	chr8	6820385	T	G	43	23,00%	10						3,96E-008
NA12760	reg_DEFA	chr8	6820395	G	A	46	20,00%	9						9,18E-007
NA12760	reg_DEFA	chr8	6820410	C	T	51	25,00%	13						1,16E-010
NA12760	reg_DEFA	chr8	6820640	C	T	99	15,00%	15					rs2702861	9,27E-009
NA12760	reg_DEFA	chr8	6820642	T	G	100	13,00%	13						5,53E-007
NA12760	reg_DEFA	chr8	6820920	T	C	64	12,00%	8						1,10E-004
NA12760	reg_DEFA	chr8	6820983	G	A	43	100,00%	43			rs2615779			1,00E-012
NA12760	reg_DEFA	chr8	6824570	C	A	6	100,00%	6	-	DEFA1B	rs2979395	rs3758132		1,48E-010
NA12760	reg_DEFA	chr8	6824643	A	G	6	100,00%	6	-	DEFA1B	rs2472240	rs41421151		1,48E-010
NA12760	reg_DEFA	chr8	6824741	G	A	6	100,00%	6	-	DEFA1B			rs58955451	1,48E-010
NA12760	reg_DEFA	chr8	6825349	C	T	29	100,00%	29			rs2978854			1,00E-012
NA12760	reg_DEFA	chr8	6825357	G	A	29	100,00%	29			rs2951835			1,00E-012
NA12760	reg_DEFA	chr8	6825678	A	G	11	100,00%	11			rs6993352			1,00E-012
NA12760	reg_DEFA	chr8	6825754	C	T	8	100,00%	8			rs6986023			1,00E-012
NA12760	reg_DEFA	chr8	6825821	C	T	5	100,00%	5						6,44E-009
NA12760	reg_DEFA	chr8	6843562	A	G	10	90,00%	9	-	DEFA1B	rs2739219			1,00E-012
NA12760	reg_DEFA	chr8	6843896	G	A	38	95,00%	36	-	DEFA1B			rs71509218	1,00E-012
NA12760	reg_DEFA	chr8	6844623	A	C	9	100,00%	9			rs28515027			1,00E-012
NA12760	reg_DEFA	chr8	6846987	A	T	3	100,00%	3					rs6651509	1,22E-005
NA12760	reg_DEFA	chr8	6859147	G	T	29	86,00%	25					rs2615778	1,00E-012
NA12760	reg_DEFA	chr8	6859203	G	A	43	100,00%	43			rs28532282			1,00E-012
NA12760	reg_DEFA	chr8	6859619	T	G	63	100,00%	63			rs2739221			1,00E-012
NA12760	reg_DEFA	chr8	6859622	A	G	62	11,00%	7						5,53E-004
NA12760	reg_DEFA	chr8	6859843	G	A	71	39,00%	28			rs2739220			1,00E-012
NA12760	reg_DEFA	chr8	6859866	A	G	74	22,00%	16					rs4841796	1,35E-011
NA12760	reg_DEFA	chr8	6860180	A	G	66	15,00%	10						2,69E-006
NA12760	reg_DEFA	chr8	6860244	G	T	55	24,00%	13			rs10105163			2,98E-010
NA12760	reg_DEFA	chr8	6860856	G	T	51	35,00%	18	-	DEFA1B	rs4841813			1,00E-012
NA12760	reg_DEFA	chr8	6861144	T	G	61	49,00%	30	-	DEFA1B			rs4840655	1,00E-012
NA12760	reg_DEFA	chr8	6861165	G	A	66	48,00%	32	-	DEFA1B			rs73195997	1,00E-012
NA12760	reg_DEFA	chr8	6861269	A	T	51	92,00%	47	-	DEFA1B	rs2702913			1,00E-012
NA12760	reg_DEFA	chr8	6862083	G	T	26	100,00%	26	-	DEFA1B				1,00E-012
NA12760	reg_DEFA	chr8	6862522	A	G	18	94,00%	17	-	DEFA1B	rs4841798			1,00E-012
NA12760	reg_DEFA	chr8	6862786	A	C	70	13,00%	9	-	DEFA1B	rs3758132			3,30E-005
NA12760	reg_DEFA	chr8	6862950	G	T	79	23,00%	18	-	DEFA1B	rs34582127	rs2615798		1,00E-012
NA12760	reg_DEFA	chr8	6864348	C	G	74	41,00%	30						1,00E-012
NA12760	reg_DEFA	chr8	6864445	T	C	75	11,00%	8					rs62487509	3,35E-004
NA12760	reg_DEFA	chr8	6864926	T	C	99	45,00%	45			rs2739218			1,00E-012
NA12760	reg_DEFA	chr8	6864943	A	G	96	26,00%	25					rs59380237	1,00E-012
NA12760	reg_DEFA	chr8	6865114	G	C	49	35,00%	17			rs4012963			1,00E-012
NA12760	reg_DEFA	chr8	6865153	G	A	37	27,00%	10			rs4012962			8,17E-009
NA12760	reg_DEFA	chr8	6865330	G	A	43	56,00%	24						1,00E-012
NA12760	reg_DEFA	chr8	6865813	T	C	34	18,00%	6			rs11781199			1,14E-004
NA12760	reg_DEFA	chr8	6865875	T	C	29	31,00%	9			rs11781205			1,19E-008
NA12760	reg_DEFA	chr8	6865966	T	C	35	23,00%	8			rs11781229			1,06E-006
NA12760	reg_DEFA	chr8	6866059	A	T	40	22,00%	9					rs56016462	2,58E-007
NA12760	reg_DEFA	chr8	6866080	G	C	39	21,00%	8					rs55836016	2,55E-006

NA12760	reg_DEFA	chr8	6866454	T	G	39	44,00%	17		rs59305955	1,00E-012
NA12760	reg_DEFA	chr8	6866462	G	C	40	42,00%	17			1,00E-012
NA12760	reg_DEFA	chr8	6866888	G	C	10	100,00%	10	rs35858635		1,00E-012
NA12760	reg_DEFA	chr8	6866891	G	C	10	100,00%	10	rs34985860		1,00E-012
NA12760	reg_DEFA	chr8	6866895	G	C	11	100,00%	11	rs35564068		1,00E-012
NA12760	reg_DEFA	chr8	6866897	G	C	11	100,00%	11	rs35820601		1,00E-012
NA12760	reg_DEFA	chr8	6866947	G	C	12	100,00%	12	rs4310228		1,00E-012
NA12760	reg_DEFA	chr8	6867985	T	C	40	50,00%	20	rs4300027		1,00E-012
NA12760	reg_DEFA	chr8	6868004	C	T	45	51,00%	23	rs4512398		1,00E-012
NA12760	reg_DEFA	chr8	6868335	A	G	39	59,00%	23	rs7826487		1,00E-012
NA12760	reg_DEFA	chr8	6868373	T	C	36	50,00%	18	rs6605578		1,00E-012
NA12760	reg_DEFA	chr8	6868637	C	T	47	55,00%	26	rs17078510		1,00E-012
NA12760	reg_DEFA	chr8	6868805	G	A	50	48,00%	24	rs7841223		1,00E-012
NA12760	reg_DEFA	chr8	6869048	A	G	56	54,00%	30	rs4288398		1,00E-012
NA12760	reg_DEFA	chr8	6869398	T	C	54	48,00%	26	rs4313182		1,00E-012
NA12760	reg_DEFA	chr8	6869803	C	G	53	53,00%	28	rs883182		1,00E-012
NA12760	reg_DEFA	chr8	6869887	A	T	56	98,00%	55	rs4481622		1,00E-012
NA12760	reg_DEFA	chr8	6870176	T	G	89	99,00%	88	rs4314670		1,00E-012
NA12760	reg_DEFA	chr8	6870220	G	T	95	99,00%	94	rs4332158		1,00E-012
NA12760	reg_DEFA	chr8	6870259	G	A	85	53,00%	45	rs4332159		1,00E-012
NA12760	reg_DEFA	chr8	6870678	C	T	53	38,00%	20	rs4448290		1,00E-012
NA12760	reg_DEFA	chr8	6870686	G	C	51	37,00%	19	rs4469481		1,00E-012
NA12760	reg_DEFA	chr8	6870701	A	G	49	35,00%	17	rs9774483		1,00E-012
NA12760	reg_DEFA	chr8	6871262	C	T	32	47,00%	15			1,00E-012
NA12760	reg_DEFA	chr8	6871265	A	G	32	100,00%	32	rs4840665		1,00E-012
NA12760	reg_DEFA	chr8	6872534	A	C	50	48,00%	24	rs6605579		1,00E-012
NA12760	reg_DEFA	chr8	6874265	A	T	38	100,00%	38	rs7821152		1,00E-012
NA12760	reg_DEFA	chr8	6874382	G	A	28	46,00%	13	rs4403430		1,00E-012
NA12760	reg_DEFA	chr8	6874473	T	G	29	41,00%	12	rs11137086		1,00E-012
NA12760	reg_DEFA	chr8	6875301	C	T	42	55,00%	23		rs62487515	1,00E-012
NA12760	reg_DEFA	chr8	6875366	G	A	46	48,00%	22	rs6982814		1,00E-012
NA12760	reg_DEFA	chr8	6875538	C	A	44	32,00%	14		rs56230231	1,00E-012
NA12760	reg_DEFA	chr8	6875544	T	C	43	44,00%	19		rs55851618	1,00E-012
NA12760	reg_DEFA	chr8	6875673	C	G	39	44,00%	17		rs55660132	1,00E-012
NA12760	reg_DEFA	chr8	6875975	A	G	40	47,00%	19		rs55740316	1,00E-012
NA12760	reg_DEFA	chr8	6877006	A	T	41	44,00%	18	rs7011708		1,00E-012
NA12760	reg_DEFA	chr8	6877045	G	A	46	48,00%	22	rs11776120		1,00E-012
NA12760	reg_DEFA	chr8	6877291	A	G	47	36,00%	17	rs11786781		1,00E-012
NA12760	reg_DEFA	chr8	6877358	G	C	47	40,00%	19	rs6993492		1,00E-012
NA12760	reg_DEFA	chr8	6877487	T	C	50	36,00%	18	rs34825638		1,00E-012
NA12760	reg_DEFA	chr8	6877608	G	A	54	37,00%	20	rs7824527		1,00E-012
NA12760	reg_DEFA	chr8	6877988	G	T	61	51,00%	31	rs7825124		1,00E-012
NA12760	reg_DEFA	chr8	6878106	C	G	55	44,00%	24			1,00E-012
NA12760	reg_DEFA	chr8	6878312	T	C	34	32,00%	11	rs4490865		1,76E-010
NA12760	reg_DEFA	chr8	6879022	T	G	49	94,00%	46	rs4841816		1,00E-012
NA12760	reg_DEFA	chr8	6879381	C	G	56	41,00%	23	rs4433170		1,00E-012
NA12760	reg_DEFA	chr8	6879828	A	G	34	53,00%	18	rs4602905		1,00E-012
NA12760	reg_DEFA	chr8	6880925	A	G	17	41,00%	7			5,41E-008
NA12760	reg_DEFA	chr8	6881221	C	T	10	60,00%	6		rs11774769	2,87E-008
NA12760	reg_DEFA	chr8	6881365	G	T	25	48,00%	12	rs17078546		1,00E-012
NA12760	reg_DEFA	chr8	6881460	G	A	40	45,00%	18	rs7015200		1,00E-012
NA12760	reg_DEFA	chr8	6881573	G	A	48	40,00%	19	rs11775034		1,00E-012
NA12760	reg_DEFA	chr8	6881816	T	C	55	49,00%	27	rs7004995		1,00E-012

NA12760	reg_DEFA	chr8	6882227	G	T	27	56,00%	15	rs34333583		1,00E-012
NA12760	reg_DEFA	chr8	6882354	T	A	8	50,00%	4	rs35708338		1,82E-005
NA12760	reg_DEFA	chr8	6882359	G	T	8	50,00%	4	rs35866869		1,82E-005
NA12760	reg_DEFA	chr8	6882374	T	C	7	43,00%	3	rs34268546		3,97E-004
NA12760	reg_DEFA	chr8	6882583	C	G	13	46,00%	6		rs2515504	2,21E-007
NA12760	reg_DEFA	chr8	6882620	T	C	13	46,00%	6	rs7009952		2,21E-007
NA12760	reg_DEFA	chr8	6882937	G	C	39	46,00%	18	rs34219797		1,00E-012
NA12760	reg_DEFA	chr8	6883103	C	T	26	50,00%	13	rs11994868		1,00E-012
NA12760	reg_DEFA	chr8	6883131	T	C	21	52,00%	11	rs11985027		1,00E-012
NA12760	reg_DEFA	chr8	6883151	T	G	16	50,00%	8	rs11985030		8,62E-010
NA12760	reg_DEFA	chr8	6883232	T	C	18	44,00%	8	rs11985068		2,79E-009
NA12760	reg_DEFA	chr8	6883286	T	C	23	52,00%	12	rs11985076		1,00E-012
NA12760	reg_DEFA	chr8	6883469	A	C	35	40,00%	14	rs4841817		1,00E-012
NA12760	reg_DEFA	chr8	6883493	G	T	38	37,00%	14	rs4840666		1,00E-012
NA12760	reg_DEFA	chr8	6883556	G	C	48	42,00%	20	rs17078556		1,00E-012
NA12760	reg_DEFA	chr8	6883571	T	G	50	36,00%	18	rs35196527		1,00E-012
NA12760	reg_DEFA	chr8	6883577	A	G	50	36,00%	18	rs34725312		1,00E-012
NA12760	reg_DEFA	chr8	6883643	C	T	66	42,00%	28	rs4621824		1,00E-012
NA12760	reg_DEFA	chr8	6883724	A	G	79	38,00%	30	rs4345578		1,00E-012
NA12760	reg_DEFA	chr8	6883754	T	C	80	39,00%	31	rs4342629		1,00E-012
NA12760	reg_DEFA	chr8	6883823	G	C	84	99,00%	83	rs4342630		1,00E-012
NA12760	reg_DEFA	chr8	6883831	G	C	85	100,00%	85	rs4342631		1,00E-012
NA12760	reg_DEFA	chr8	6883942	C	T	82	41,00%	34	rs4549798		1,00E-012
NA12760	reg_DEFA	chr8	6883982	A	C	75	100,00%	75	rs4342632		1,00E-012
NA12760	reg_DEFA	chr8	6884242	G	T	42	93,00%	39	rs4601339		1,00E-012
NA12760	reg_DEFA	chr8	6884304	G	T	30	100,00%	30	rs4601340		1,00E-012
NA12760	reg_DEFA	chr8	6884370	C	A	32	94,00%	30	rs4642671		1,00E-012
NA12760	reg_DEFA	chr8	6885310	C	T	32	41,00%	13	rs4546682		1,00E-012
NA12760	reg_DEFA	chr8	6885553	C	T	24	67,00%	16	rs6996890		1,00E-012
NA12760	reg_DEFA	chr8	6885796	T	G	39	54,00%	21	rs4841818		1,00E-012
NA12760	reg_DEFA	chr8	6886141	A	G	44	50,00%	22	rs4358823		1,00E-012
NA12760	reg_DEFA	chr8	6887746	G	A	51	49,00%	25	rs10086568		1,00E-012
NA12760	reg_DEFA	chr8	6888052	G	A	31	42,00%	13	rs13257504		1,00E-012
NA12760	reg_DEFA	chr8	6888529	G	C	17	65,00%	11			1,00E-012
NA12760	reg_DEFA	chr8	6889029	C	G	31	65,00%	20	rs4304345		1,00E-012
NA12760	reg_DEFA	chr8	6889086	C	T	34	35,00%	12			1,59E-011
NA12760	reg_DEFA	chr8	6889453	G	A	25	56,00%	14		rs55690609	1,00E-012
NA12760	reg_DEFA	chr8	6890057	T	A	50	62,00%	31	rs4841822		1,00E-012
NA12760	reg_DEFA	chr8	6890276	C	T	40	42,00%	17	rs4260915		1,00E-012
NA12760	reg_DEFA	chr8	6890313	C	G	40	50,00%	20	rs4260916		1,00E-012
NA12760	reg_DEFA	chr8	6890558	G	A	32	56,00%	18	rs4379464		1,00E-012
NA12760	reg_DEFA	chr8	6891017	C	A	40	55,00%	22	rs12682030		1,00E-012
NA12760	reg_DEFA	chr8	6891739	T	C	71	59,00%	42		rs61187659	1,00E-012
NA12760	reg_DEFA	chr8	6891919	A	G	64	59,00%	38	rs6999181		1,00E-012
NA12760	reg_DEFA	chr8	6892711	A	G	35	43,00%	15	rs13249918		1,00E-012
NA12760	reg_DEFA	chr8	6893086	T	C	19	37,00%	7	rs12544774		1,34E-007
NA12760	reg_DEFA	chr8	6893641	G	A	25	52,00%	13			1,00E-012
NA12760	reg_DEFA	chr8	6896339	C	G	11	55,00%	6	rs4446760		6,19E-008
NA12760	reg_DEFA	chr8	6897064	A	C	53	51,00%	27	rs9644778		1,00E-012
NA12760	reg_DEFA	chr8	6898012	G	C	38	47,00%	18	rs6605583		1,00E-012
NA12760	reg_DEFA	chr8	6898076	G	A	41	44,00%	18	rs4841830		1,00E-012
NA12760	reg_DEFA	chr8	6898613	T	G	61	36,00%	22	rs12680521		1,00E-012
NA12760	reg_DEFA	chr8	6899982	G	A	50	60,00%	30	rs4645580		1,00E-012

NA12760	reg_DEFA	chr8	6901260	A	G	42	57,00%	24	-	DEFA5	rs10095331			1,00E-012
NA12760	reg_DEFA	chr8	6902056	T	C	61	31,00%	19			rs4395911			1,00E-012
NA12760	reg_DEFA	chr8	6902827	G	C	33	100,00%	33			rs4415345			1,00E-012
NA12760	reg_DEFA	chr8	6903072	A	G	52	98,00%	51			rs6995789			1,00E-012
NA12760	reg_DEFA	chr8	6903083	C	G	52	100,00%	52			rs6988319			1,00E-012
NA12760	reg_DEFA	chr8	6904688	A	G	24	37,00%	9			rs41471547	rs4841831		1,72E-009
NA12760	reg_DEFA	chr8	6905827	C	G	38	53,00%	20					rs66643008	1,00E-012
NA12760	reg_DEFA	chr8	6907158	G	T	25	48,00%	12			rs7834209			1,00E-012
NA12760	reg_DEFA	chr8	6907549	C	G	72	29,00%	21					rs57992355	1,00E-012
NA12760	reg_DEFA	chr8	6909077	T	G	33	67,00%	22			rs4394417			1,00E-012
NA12760	reg_DEFB	chr8	7225885	T	C	45	64,00%	29						1,00E-012
NA12760	reg_DEFB	chr8	7226233	T	C	117	19,00%	22			rs2719528			1,00E-012
NA12760	reg_DEFB	chr8	7226320	A	G	116	37,00%	43			rs2740644			1,00E-012
NA12760	reg_DEFB	chr8	7226330	C	T	117	38,00%	44			rs4592060			1,00E-012
NA12760	reg_DEFB	chr8	7226617	C	G	75	45,00%	34			rs2719527			1,00E-012
NA12760	reg_DEFB	chr8	7226633	C	G	70	36,00%	25					rs4610778	1,00E-012
NA12760	reg_DEFB	chr8	7226645	T	C	71	51,00%	36					rs2698882	1,00E-012
NA12760	reg_DEFB	chr8	7226650	T	G	71	99,00%	70					rs62636823	1,00E-012
NA12760	reg_DEFB	chr8	7226864	C	A	78	44,00%	34			rs2740643			1,00E-012
NA12760	reg_DEFB	chr8	7227149	T	G	131	50,00%	66					rs2739892	1,00E-012
NA12760	reg_DEFB	chr8	7227202	C	G	117	79,00%	92			rs4110303			1,00E-012
NA12760	reg_DEFB	chr8	7227284	A	C	120	29,00%	35					rs71511231	1,00E-012
NA12760	reg_DEFB	chr8	7227393	T	C	115	47,00%	54					rs58068279	1,00E-012
NA12760	reg_DEFB	chr8	7227554	G	A	114	44,00%	50					rs60497429	1,00E-012
NA12760	reg_DEFB	chr8	7227642	C	T	95	84,00%	80			rs3915363			1,00E-012
NA12760	reg_DEFB	chr8	7227652	C	G	95	45,00%	43			rs2719524			1,00E-012
NA12760	reg_DEFB	chr8	7227677	C	A	90	44,00%	40			rs2719523			1,00E-012
NA12760	reg_DEFB	chr8	7227756	G	C	72	47,00%	34					rs5004606	1,00E-012
NA12760	reg_DEFB	chr8	7227784	C	T	73	45,00%	33					rs4096974	1,00E-012
NA12760	reg_DEFB	chr8	7227789	T	C	69	43,00%	30					rs3915362	1,00E-012
NA12760	reg_DEFB	chr8	7227922	A	T	53	70,00%	37			rs28413583	rs5004605		1,00E-012
NA12760	reg_DEFB	chr8	7227939	C	T	48	23,00%	11			rs2698880			9,81E-009
NA12760	reg_DEFB	chr8	7228194	T	C	64	52,00%	33			rs2719522			1,00E-012
NA12760	reg_DEFB	chr8	7228313	T	C	85	40,00%	34			rs2719521			1,00E-012
NA12760	reg_DEFB	chr8	7228345	G	A	85	44,00%	37			rs2719520			1,00E-012
NA12760	reg_DEFB	chr8	7228356	A	G	83	47,00%	39					rs2739890	1,00E-012
NA12760	reg_DEFB	chr8	7228410	G	A	82	15,00%	12					rs5000920	4,07E-007
NA12760	reg_DEFB	chr8	7228430	A	C	82	44,00%	36			rs2719519			1,00E-012
NA12760	reg_DEFB	chr8	7228471	A	G	88	40,00%	35						1,00E-012
NA12760	reg_DEFB	chr8	7228505	T	C	90	39,00%	35						1,00E-012
NA12760	reg_DEFB	chr8	7228522	G	C	87	39,00%	34			rs2466107			1,00E-012
NA12760	reg_DEFB	chr8	7228560	A	G	88	41,00%	36					rs2463972	1,00E-012
NA12760	reg_DEFB	chr8	7228802	T	G	83	11,00%	9					rs2719518	1,28E-004
NA12760	reg_DEFB	chr8	7229225	A	G	101	18,00%	18					rs2739886	2,43E-011
NA12760	reg_DEFB	chr8	7229247	G	A	104	44,00%	46					rs2719517	1,00E-012
NA12760	reg_DEFB	chr8	7229277	A	G	107	42,00%	45					rs2739885	1,00E-012
NA12760	reg_DEFB	chr8	7229410	C	T	100	33,00%	33					rs71236244	1,00E-012
NA12760	reg_DEFB	chr8	7229511	A	G	91	30,00%	27					rs2739883	1,00E-012
NA12760	reg_DEFB	chr8	7229783	G	A	116	10,00%	12					rs2698877	1,64E-005
NA12760	reg_DEFB	chr8	7229789	T	C	116	41,00%	48			rs2719516			1,00E-012
NA12760	reg_DEFB	chr8	7230035	C	T	85	44,00%	37			rs2739882			1,00E-012
NA12760	reg_DEFB	chr8	7230147	C	T	61	43,00%	26			rs2739881			1,00E-012
NA12760	reg_DEFB	chr8	7230182	T	C	65	18,00%	12					rs3898684	2,84E-008

NA12760	reg_DEFB	chr8	7230346	C	A	56	14,00%	8			4,15E-005
NA12760	reg_DEFB	chr8	7230397	A	C	49	33,00%	16	rs2698876		1,00E-012
NA12760	reg_DEFB	chr8	7230646	C	G	50	80,00%	40		rs4295681	1,00E-012
NA12760	reg_DEFB	chr8	7230690	T	G	50	14,00%	7		rs62636824	1,43E-004
NA12760	reg_DEFB	chr8	7230699	T	C	50	56,00%	28		rs2719514	1,00E-012
NA12760	reg_DEFB	chr8	7230752	G	A	48	27,00%	13		rs71511233	4,99E-011
NA12760	reg_DEFB	chr8	7231074	G	A	52	38,00%	20	rs2740639		1,00E-012
NA12760	reg_DEFB	chr8	7231137	C	A	53	40,00%	21	rs2740638		1,00E-012
NA12760	reg_DEFB	chr8	7231158	G	A	53	17,00%	9	rs2698873		3,19E-006
NA12760	reg_DEFB	chr8	7231435	G	A	97	52,00%	50		rs71511234	1,00E-012
NA12760	reg_DEFB	chr8	7231456	G	A	103	28,00%	29	rs2739878		1,00E-012
NA12760	reg_DEFB	chr8	7231537	C	T	108	36,00%	39	rs2698872		1,00E-012
NA12760	reg_DEFB	chr8	7231549	C	G	110	35,00%	39	rs2719513		1,00E-012
NA12760	reg_DEFB	chr8	7231781	A	G	107	40,00%	43	rs2739877		1,00E-012
NA12760	reg_DEFB	chr8	7232001	C	T	116	46,00%	53	rs2698870		1,00E-012
NA12760	reg_DEFB	chr8	7232162	G	A	102	48,00%	49			1,00E-012
NA12760	reg_DEFB	chr8	7232277	G	T	92	14,00%	13			2,06E-007
NA12760	reg_DEFB	chr8	7232299	C	T	98	48,00%	47	rs2719511		1,00E-012
NA12760	reg_DEFB	chr8	7232309	G	T	99	47,00%	47			1,00E-012
NA12760	reg_DEFB	chr8	7232402	T	G	114	32,00%	36		rs2739876	1,00E-012
NA12760	reg_DEFB	chr8	7232411	T	G	119	49,00%	58	rs2466106		1,00E-012
NA12760	reg_DEFB	chr8	7232542	A	G	113	20,00%	23		rs2739875	1,00E-012
NA12760	reg_DEFB	chr8	7232595	C	T	111	50,00%	56	rs2739874		1,00E-012
NA12760	reg_DEFB	chr8	7232643	A	T	110	80,00%	88	rs3915354		1,00E-012
NA12760	reg_DEFB	chr8	7232788	A	G	90	33,00%	30	rs2698867		1,00E-012
NA12760	reg_DEFB	chr8	7232919	G	A	91	16,00%	15			2,81E-009
NA12760	reg_DEFB	chr8	7232985	C	G	98	51,00%	50		rs2739873	1,00E-012
NA12760	reg_DEFB	chr8	7233075	T	C	108	51,00%	55		rs2740636	1,00E-012
NA12760	reg_DEFB	chr8	7233215	G	A	127	15,00%	19		rs2740635	1,35E-010
NA12760	reg_DEFB	chr8	7233270	T	C	133	14,00%	19		rs2698866	2,93E-010
NA12760	reg_DEFB	chr8	7233352	T	C	120	48,00%	58		rs2740634	1,00E-012
NA12760	reg_DEFB	chr8	7233515	G	A	71	45,00%	32	rs2740633		1,00E-012
NA12760	reg_DEFB	chr8	7233678	A	T	51	14,00%	7		rs2739872	1,62E-004
NA12760	reg_DEFB	chr8	7233706	A	G	53	43,00%	23	rs2739871		1,00E-012
NA12760	reg_DEFB	chr8	7233725	C	T	58	33,00%	19	rs2740632		1,00E-012
NA12760	reg_DEFB	chr8	7233768	G	C	57	40,00%	23	rs2740631		1,00E-012
NA12760	reg_DEFB	chr8	7233949	C	G	85	13,00%	11		rs2739870	4,24E-006
NA12760	reg_DEFB	chr8	7234062	A	C	93	45,00%	42	rs2719510		1,00E-012
NA12760	reg_DEFB	chr8	7234084	G	T	97	43,00%	42	rs28544205		1,00E-012
NA12760	reg_DEFB	chr8	7234115	A	G	107	40,00%	43		rs2739868	1,00E-012
NA12760	reg_DEFB	chr8	7234191	C	T	123	42,00%	52		rs2739867	1,00E-012
NA12760	reg_DEFB	chr8	7234287	C	T	134	43,00%	58		rs2739866	1,00E-012
NA12760	reg_DEFB	chr8	7234433	A	C	131	46,00%	60		rs2698865	1,00E-012
NA12760	reg_DEFB	chr8	7234521	A	T	119	48,00%	57		rs2739865	1,00E-012
NA12760	reg_DEFB	chr8	7234552	T	C	110	53,00%	58		rs2740630	1,00E-012
NA12760	reg_DEFB	chr8	7234557	T	C	110	54,00%	59		rs2740629	1,00E-012
NA12760	reg_DEFB	chr8	7234590	G	A	100	53,00%	53		rs2740628	1,00E-012
NA12760	reg_DEFB	chr8	7234600	G	A	98	12,00%	12		rs2740627	2,84E-006
NA12760	reg_DEFB	chr8	7234601	C	T	96	24,00%	23			1,00E-012
NA12760	reg_DEFB	chr8	7234948	A	T	89	16,00%	14			1,75E-008
NA12760	reg_DEFB	chr8	7234989	C	T	84	52,00%	44		rs2698864	1,00E-012
NA12760	reg_DEFB	chr8	7235018	G	A	87	54,00%	47		rs2740626	1,00E-012
NA12760	reg_DEFB	chr8	7235095	G	A	98	44,00%	43		rs4437677	1,00E-012

NA12760	reg_DEFB	chr8	7235113	T	A	103	49,00%	50		rs4439153	1,00E-012
NA12760	reg_DEFB	chr8	7235127	A	G	106	48,00%	51		rs4633080	1,00E-012
NA12760	reg_DEFB	chr8	7235144	G	A	114	47,00%	54		rs4545129	1,00E-012
NA12760	reg_DEFB	chr8	7235204	C	T	127	47,00%	60		rs4623450	1,00E-012
NA12760	reg_DEFB	chr8	7235255	G	A	135	47,00%	63		rs4545128	1,00E-012
NA12760	reg_DEFB	chr8	7235443	G	C	144	98,00%	141	rs9720375		1,00E-012
NA12760	reg_DEFB	chr8	7235591	C	G	136	44,00%	60	rs2740625		1,00E-012
NA12760	reg_DEFB	chr8	7235889	C	G	83	13,00%	11	rs2698951		3,35E-006
NA12760	reg_DEFB	chr8	7235967	G	T	62	34,00%	21	rs2719506		1,00E-012
NA12760	reg_DEFB	chr8	7236029	G	C	45	36,00%	16		rs2740624	1,00E-012
NA12760	reg_DEFB	chr8	7236128	G	T	34	32,00%	11	rs2719502		1,76E-010
NA12760	reg_DEFB	chr8	7236404	C	G	46	37,00%	17		rs3958986	1,00E-012
NA12760	reg_DEFB	chr8	7236418	C	T	47	36,00%	17		rs3988877	1,00E-012
NA12760	reg_DEFB	chr8	7236506	A	G	61	38,00%	23		rs2719494	1,00E-012
NA12760	reg_DEFB	chr8	7236551	T	C	61	38,00%	23	rs28459499		1,00E-012
NA12760	reg_DEFB	chr8	7236608	C	T	67	34,00%	23		rs2740165	1,00E-012
NA12760	reg_DEFB	chr8	7236670	C	A	62	31,00%	19		rs62636825	1,00E-012
NA12760	reg_DEFB	chr8	7236701	C	T	69	39,00%	27	rs2740164		1,00E-012
NA12760	reg_DEFB	chr8	7236753	G	A	78	44,00%	34	rs2740623		1,00E-012
NA12760	reg_DEFB	chr8	7236764	T	C	77	30,00%	23		rs2740163	1,00E-012
NA12760	reg_DEFB	chr8	7236897	G	C	72	25,00%	18		rs72494251	1,00E-012
NA12760	reg_DEFB	chr8	7237003	A	G	67	43,00%	29		rs2740622	1,00E-012
NA12760	reg_DEFB	chr8	7237030	G	A	70	11,00%	8			2,08E-004
NA12760	reg_DEFB	chr8	7237110	G	A	71	46,00%	33		rs2740621	1,00E-012
NA12760	reg_DEFB	chr8	7237169	C	T	79	46,00%	36		rs2740162	1,00E-012
NA12760	reg_DEFB	chr8	7237229	G	A	73	42,00%	31		rs2740620	1,00E-012
NA12760	reg_DEFB	chr8	7237268	C	T	71	41,00%	29		rs2740161	1,00E-012
NA12760	reg_DEFB	chr8	7237506	C	T	53	41,00%	22	rs2740160		1,00E-012
NA12760	reg_DEFB	chr8	7237549	T	C	47	36,00%	17		rs72626631	1,00E-012
NA12760	reg_DEFB	chr8	7237560	A	G	45	44,00%	20	rs2740618		1,00E-012
NA12760	reg_DEFB	chr8	7237571	A	T	44	23,00%	10		rs55939595	5,02E-008
NA12760	reg_DEFB	chr8	7237869	T	C	48	48,00%	23		rs2740616	1,00E-012
NA12760	reg_DEFB	chr8	7237961	T	C	56	37,00%	21		rs2740615	1,00E-012
NA12760	reg_DEFB	chr8	7238229	G	C	45	18,00%	8	rs2719466		7,89E-006
NA12760	reg_DEFB	chr8	7238361	A	G	31	42,00%	13	rs2719465		1,00E-012
NA12760	reg_DEFB	chr8	7238365	A	G	31	45,00%	14	rs9720329		1,00E-012
NA12760	reg_DEFB	chr8	7238371	A	T	32	44,00%	14		rs2698958	1,00E-012
NA12760	reg_DEFB	chr8	7239140	C	T	35	51,00%	18		rs2740159	1,00E-012
NA12760	reg_DEFB	chr8	7239175	C	T	36	19,00%	7		rs2698959	1,58E-005
NA12760	reg_DEFB	chr8	7239239	T	C	40	37,00%	15		rs2698960	1,00E-012
NA12760	reg_DEFB	chr8	7239336	T	C	46	24,00%	11			6,04E-009
NA12760	reg_DEFB	chr8	7239401	C	G	53	58,00%	31	rs2740613		1,00E-012
NA12760	reg_DEFB	chr8	7239431	A	G	63	56,00%	35	rs2719454		1,00E-012
NA12760	reg_DEFB	chr8	7239620	T	C	102	13,00%	13	rs2740612		6,97E-007
NA12760	reg_DEFB	chr8	7239710	T	G	128	49,00%	63	rs2740611		1,00E-012
NA12760	reg_DEFB	chr8	7240036	G	A	93	29,00%	27		rs2740610	1,00E-012
NA12760	reg_DEFB	chr8	7240395	C	A	51	96,00%	49	rs13260072		1,00E-012
NA12760	reg_DEFB	chr8	7240612	C	A	70	47,00%	33	rs2740609		1,00E-012
NA12760	reg_DEFB	chr8	7240639	C	G	67	12,00%	8		rs2698962	1,52E-004
NA12760	reg_DEFB	chr8	7240641	G	C	67	12,00%	8		rs2719435	1,52E-004
NA12760	reg_DEFB	chr8	7240705	T	C	74	14,00%	10	rs7005466		7,76E-006
NA12760	reg_DEFB	chr8	7240763	G	C	70	13,00%	9		rs2719434	3,30E-005
NA12760	reg_DEFB	chr8	7240831	C	T	56	70,00%	39		rs71250725	1,00E-012

NA12760	reg_DEFB	chr8	7240856	C	A	54	46,00%	25	rs2740608			1,00E-012
NA12760	reg_DEFB	chr8	7240870	C	T	53	15,00%	8			rs2719433	2,76E-005
NA12760	reg_DEFB	chr8	7241049	T	C	28	32,00%	9			rs2698963	8,38E-009
NA12760	reg_DEFB	chr8	7241078	T	C	30	53,00%	16			rs71250726	1,00E-012
NA12760	reg_DEFB	chr8	7241147	C	A	39	49,00%	19			rs2698964	1,00E-012
NA12760	reg_DEFB	chr8	7241218	T	C	41	22,00%	9	rs7009957			3,24E-007
NA12760	reg_DEFB	chr8	7241264	C	T	43	56,00%	24	rs4625055			1,00E-012
NA12760	reg_DEFB	chr8	7241282	A	G	46	26,00%	12			rs2740607	4,14E-010
NA12760	reg_DEFB	chr8	7241285	A	G	45	51,00%	23			rs3958824	1,00E-012
NA12760	reg_DEFB	chr8	7241358	T	C	43	40,00%	17			rs2740157	1,00E-012
NA12760	reg_DEFB	chr8	7241366	G	T	42	38,00%	16			rs2740606	1,00E-012
NA12760	reg_DEFB	chr8	7241581	G	C	50	14,00%	7			rs4540430	1,43E-004
NA12760	reg_DEFB	chr8	7241686	A	G	59	76,00%	45	rs4571754			1,00E-012
NA12760	reg_DEFB	chr8	7241779	A	G	56	32,00%	18			rs2719626	1,00E-012
NA12760	reg_DEFB	chr8	7241782	T	G	59	59,00%	35				1,00E-012
NA12760	reg_DEFB	chr8	7241787	C	T	65	45,00%	29			rs2740605	1,00E-012
NA12760	reg_DEFB	chr8	7241835	T	C	71	18,00%	13	rs2740604			8,42E-009
NA12760	reg_DEFB	chr8	7241903	G	A	95	19,00%	18				8,12E-012
NA12760	reg_DEFB	chr8	7242034	T	A	107	63,00%	67			rs71247376	1,00E-012
NA12760	reg_DEFB	chr8	7242037	G	C	106	63,00%	67			rs71247376	1,00E-012
NA12760	reg_DEFB	chr8	7242252	G	C	109	74,00%	81			rs3988892	1,00E-012
NA12760	reg_DEFB	chr8	7242301	C	G	107	10,00%	11			rs2740750	3,88E-005
NA12760	reg_DEFB	chr8	7242513	T	C	78	74,00%	58			rs3988890	1,00E-012
NA12760	reg_DEFB	chr8	7242527	T	C	76	11,00%	8				3,67E-004
NA12760	reg_DEFB	chr8	7242637	T	G	62	68,00%	42			rs3927359	1,00E-012
NA12760	reg_DEFB	chr8	7242769	A	T	60	13,00%	8			rs72494256	6,90E-005
NA12760	reg_DEFB	chr8	7242795	G	C	68	85,00%	58			rs3928107	1,00E-012
NA12760	reg_DEFB	chr8	7242850	G	A	76	29,00%	22			rs2740749	1,00E-012
NA12760	reg_DEFB	chr8	7242925	C	T	83	57,00%	47	rs2698913			1,00E-012
NA12760	reg_DEFB	chr8	7242996	A	C	98	16,00%	16				9,75E-010
NA12760	reg_DEFB	chr8	7243080	G	A	122	67,00%	82				1,00E-012
NA12760	reg_DEFB	chr8	7243097	A	G	125	66,00%	83				1,00E-012
NA12760	reg_DEFB	chr8	7243226	G	C	145	66,00%	96			rs56017112	1,00E-012
NA12760	reg_DEFB	chr8	7243230	C	T	144	48,00%	69			rs2719607	1,00E-012
NA12760	reg_DEFB	chr8	7243275	T	A	142	46,00%	65			rs2719604	1,00E-012
NA12760	reg_DEFB	chr8	7243307	T	C	140	44,00%	62			rs2719603	1,00E-012
NA12760	reg_DEFB	chr8	7243444	T	G	131	17,00%	22			rs55647651	1,00E-012
NA12760	reg_DEFB	chr8	7243453	C	T	131	60,00%	79	rs4840743			1,00E-012
NA12760	reg_DEFB	chr8	7243457	A	T	126	77,00%	97	rs4840275			1,00E-012
NA12760	reg_DEFB	chr8	7243460	T	C	126	40,00%	50				1,00E-012
NA12760	reg_DEFB	chr8	7243500	A	G	117	16,00%	19	rs2698912			3,87E-011
NA12760	reg_DEFB	chr8	7243502	T	C	120	16,00%	19	rs2740153			4,87E-011
NA12760	reg_DEFB	chr8	7243506	A	T	119	16,00%	19				4,19E-011
NA12760	reg_DEFB	chr8	7243517	G	A	116	28,00%	32			rs2463985	1,00E-012
NA12760	reg_DEFB	chr8	7243526	C	T	113	29,00%	33			rs2466114	1,00E-012
NA12760	reg_DEFB	chr8	7243649	A	G	124	31,00%	38	rs2463984			1,00E-012
NA12760	reg_DEFB	chr8	7243720	C	A	145	17,00%	25	rs2698911			1,00E-012
NA12760	reg_DEFB	chr8	7243739	T	C	145	32,00%	46				1,00E-012
NA12760	reg_DEFB	chr8	7243778	T	C	137	53,00%	73	rs2737539			1,00E-012
NA12760	reg_DEFB	chr8	7243788	G	C	134	38,00%	51	rs2740152			1,00E-012
NA12760	reg_DEFB	chr8	7243809	T	C	130	31,00%	40	rs2977410			1,00E-012
NA12760	reg_DEFB	chr8	7243942	C	A	112	16,00%	18	rs2463983			1,20E-010
NA12760	reg_DEFB	chr8	7243954	C	A	105	18,00%	19	rs2740150	rs34384791		1,41E-011

NA12760	reg_DEFB	chr8	7243999	A	G	91	19,00%	17	rs2740149	rs35454996	3,92E-011
NA12760	reg_DEFB	chr8	7244056	A	T	75	32,00%	24	rs6983203		1,00E-012
NA12760	reg_DEFB	chr8	7244068	T	C	72	21,00%	15			1,03E-010
NA12760	reg_DEFB	chr8	7244089	C	T	62	29,00%	18	rs7013742		1,00E-012
NA12760	reg_DEFB	chr8	7244144	T	A	43	33,00%	14	rs6988073		1,00E-012
NA12760	reg_DEFB	chr8	7244187	T	C	43	30,00%	13	rs9693476		2,49E-011
NA12760	reg_DEFB	chr8	7244256	G	C	45	47,00%	21	rs2954087		1,00E-012
NA12760	reg_DEFB	chr8	7244281	G	A	48	21,00%	10	rs9694285		1,22E-007
NA12760	reg_DEFB	chr8	7244286	C	A	47	21,00%	10	rs9694349		9,84E-008
NA12760	reg_DEFB	chr8	7244326	C	T	52	19,00%	10	rs9694359		2,71E-007
NA12760	reg_DEFB	chr8	7244340	C	T	52	15,00%	8	rs2977415		2,39E-005
NA12760	reg_DEFB	chr8	7244470	T	G	68	18,00%	12			4,82E-008
NA12760	reg_DEFB	chr8	7244486	G	A	68	19,00%	13			4,85E-009
NA12760	reg_DEFB	chr8	7244542	C	A	66	17,00%	11			3,19E-007
NA12760	reg_DEFB	chr8	7244846	T	C	96	31,00%	30	rs34757760		1,00E-012
NA12760	reg_DEFB	chr8	7244847	G	A	95	32,00%	30	rs34757760		1,00E-012
NA12760	reg_DEFB	chr8	7244856	T	G	94	34,00%	32	rs35642932		1,00E-012
NA12760	reg_DEFB	chr8	7244884	C	T	97	22,00%	21	rs35338468		1,00E-012
NA12760	reg_DEFB	chr8	7244931	A	T	102	24,00%	24	rs35669108		1,00E-012
NA12760	reg_DEFB	chr8	7244935	T	A	102	24,00%	24	rs34965334		1,00E-012
NA12760	reg_DEFB	chr8	7245066	G	A	109	15,00%	16	rs2977405		4,82E-009
NA12760	reg_DEFB	chr8	7245081	C	G	116	27,00%	31	rs2980526		1,00E-012
NA12760	reg_DEFB	chr8	7245136	C	T	123	28,00%	34	rs2977406		1,00E-012
NA12760	reg_DEFB	chr8	7245160	T	C	126	26,00%	33	rs2977407		1,00E-012
NA12760	reg_DEFB	chr8	7245210	G	A	124	40,00%	50	rs2977408	rs2977409	1,00E-012
NA12760	reg_DEFB	chr8	7245211	T	C	124	27,00%	33	rs2977409		1,00E-012
NA12760	reg_DEFB	chr8	7245266	C	T	118	26,00%	31	rs2698902		1,00E-012
NA12760	reg_DEFB	chr8	7245290	C	T	118	14,00%	17	rs35021319		2,17E-009
NA12760	reg_DEFB	chr8	7245325	G	A	117	29,00%	34	rs2472432		1,00E-012
NA12760	reg_DEFB	chr8	7245375	C	T	103	31,00%	32	rs2463977		1,00E-012
NA12760	reg_DEFB	chr8	7245556	A	G	78	49,00%	38	rs2740748		1,00E-012
NA12760	reg_DEFB	chr8	7245577	T	C	70	17,00%	12	rs2740148	rs4840745	6,75E-008
NA12760	reg_DEFB	chr8	7245592	T	G	66	27,00%	18	rs2740747		1,00E-012
NA12760	reg_DEFB	chr8	7245649	G	A	57	14,00%	8	rs2740146	rs4840746	4,73E-005
NA12760	reg_DEFB	chr8	7245666	G	A	57	26,00%	15	rs2740145		6,21E-012
NA12760	reg_DEFB	chr8	7245748	C	G	54	13,00%	7			2,34E-004
NA12760	reg_DEFB	chr8	7245898	G	C	72	51,00%	37	rs2719573		1,00E-012
NA12760	reg_DEFB	chr8	7246008	A	G	75	23,00%	17	rs2740746		3,52E-012
NA12760	reg_DEFB	chr8	7246164	C	T	72	24,00%	17	rs2719567		1,00E-012
NA12760	reg_DEFB	chr8	7246177	T	C	69	13,00%	9	rs2698901		2,93E-005
NA12760	reg_DEFB	chr8	7246366	C	G	55	22,00%	12	rs2740144		3,83E-009
NA12760	reg_DEFB	chr8	7246487	C	T	46	37,00%	17	rs2719563		1,00E-012
NA12760	reg_DEFB	chr8	7246593	C	G	37	35,00%	13	rs2247710		2,43E-012
NA12760	reg_DEFB	chr8	7246760	A	G	44	32,00%	14	rs2737552		1,00E-012
NA12760	reg_DEFB	chr8	7246845	T	G	50	26,00%	13	rs2737553		8,84E-011
NA12760	reg_DEFB	chr8	7246914	C	T	51	27,00%	14	rs2951098		1,73E-011
NA12760	reg_DEFB	chr8	7246948	G	A	54	28,00%	15	rs34687479		2,57E-012
NA12760	reg_DEFB	chr8	7246955	C	G	53	28,00%	15	rs35525365		1,89E-012
NA12760	reg_DEFB	chr8	7246956	A	G	54	28,00%	15	rs35525365		2,57E-012
NA12760	reg_DEFB	chr8	7247000	G	C	54	26,00%	14	rs35271627		1,74E-011
NA12760	reg_DEFB	chr8	7247013	C	G	53	25,00%	13	rs34849967		1,80E-010
NA12760	reg_DEFB	chr8	7247046	T	C	56	12,00%	7	rs2951099		2,94E-004
NA12760	reg_DEFB	chr8	7247056	G	A	56	34,00%	19	rs2954062		1,00E-012

NA12760	reg_DEFB	chr8	7247090	T	C	59	24,00%	14	rs34343796			6,50E-011
NA12760	reg_DEFB	chr8	7247188	G	T	57	23,00%	13	rs2977412	rs35145385		4,82E-010
NA12760	reg_DEFB	chr8	7247198	C	A	57	35,00%	20	rs2954061			1,00E-012
NA12760	reg_DEFB	chr8	7247272	T	C	60	20,00%	12	rs35437838	rs2719562		1,10E-008
NA12760	reg_DEFB	chr8	7247289	C	T	55	20,00%	11	rs2719561	rs35165471		4,48E-008
NA12760	reg_DEFB	chr8	7249100	T	C	3	100,00%	3	rs2740138			1,22E-005
NA12760	reg_DEFB	chr8	7249157	C	G	3	100,00%	3				1,22E-005
NA12760	reg_DEFB	chr8	7249212	C	T	3	100,00%	3			rs4324940	1,22E-005
NA12760	reg_DEFB	chr8	7249224	T	C	3	100,00%	3				1,22E-005
NA12760	reg_DEFB	chr8	7249269	T	C	3	100,00%	3			rs62494626	1,22E-005
NA12760	reg_DEFB	chr8	7249320	A	G	3	100,00%	3			rs62530484	1,22E-005
NA12760	reg_DEFB	chr8	7249925	G	A	5	60,00%	3			rs62510592	1,18E-004
NA12760	reg_DEFB	chr8	7250219	T	C	4	75,00%	3				4,78E-005
NA12760	reg_DEFB	chr8	7250371	T	C	5	60,00%	3			rs71521097	1,18E-004
NA12760	reg_DEFB	chr8	7250404	T	C	5	80,00%	4				1,37E-006
NA12760	reg_DEFB	chr8	7250428	T	G	4	75,00%	3			rs28883954	4,78E-005
NA12760	reg_DEFB	chr8	7250939	A	C	6	67,00%	4			rs13275935	4,04E-006
NA12760	reg_DEFB	chr8	7250960	G	C	7	71,00%	5			rs2471963	1,30E-007
NA12760	reg_DEFB	chr8	7251011	T	C	7	43,00%	3			rs62518636	3,97E-004
NA12760	reg_DEFB	chr8	7251070	C	A	5	60,00%	3				1,18E-004
NA12760	reg_DEFB	chr8	7251080	G	T	4	100,00%	4			rs2471964	2,80E-007
NA12760	reg_DEFB	chr8	7251118	T	C	4	100,00%	4	rs2740126			2,80E-007
NA12760	reg_DEFB	chr8	7252395	G	C	5	100,00%	5				6,44E-009
NA12760	reg_DEFB	chr8	7252426	T	C	5	80,00%	4			rs71512308	1,37E-006
NA12760	reg_DEFB	chr8	7252477	T	C	5	100,00%	5	rs2740113			6,44E-009
NA12760	reg_DEFB	chr8	7252507	A	G	6	100,00%	6				1,48E-010
NA12760	reg_DEFB	chr8	7252518	A	G	6	100,00%	6				1,48E-010
NA12760	reg_DEFB	chr8	7252592	A	G	5	100,00%	5			rs7009930	6,44E-009
NA12760	reg_DEFB	chr8	7252655	A	G	3	100,00%	3				1,22E-005
NA12760	reg_DEFB	chr8	7252666	T	G	3	100,00%	3			rs59392872	1,22E-005
NA12760	reg_DEFB	chr8	7253389	T	C	28	61,00%	17	rs2719572			1,00E-012
NA12760	reg_DEFB	chr8	7253589	G	T	34	41,00%	14	rs2698825			1,00E-012
NA12760	reg_DEFB	chr8	7253615	G	A	38	42,00%	16	rs3988853	rs2740112		1,00E-012
NA12760	reg_DEFB	chr8	7253933	A	G	49	24,00%	12	rs2698823			9,21E-010
NA12760	reg_DEFB	chr8	7253959	G	A	53	26,00%	14	rs2740111			1,31E-011
NA12760	reg_DEFB	chr8	7254208	T	C	72	22,00%	16				2,16E-011
NA12760	reg_DEFB	chr8	7254247	A	G	76	97,00%	74	rs3915368			1,00E-012
NA12760	reg_DEFB	chr8	7254287	G	A	82	23,00%	19	rs2740109			1,00E-012
NA12760	reg_DEFB	chr8	7254402	G	A	90	11,00%	10	rs2740108			4,43E-005
NA12760	reg_DEFB	chr8	7254420	T	C	92	22,00%	20	rs2740107			1,00E-012
NA12760	reg_DEFB	chr8	7254732	T	C	137	28,00%	38	rs2740742			1,00E-012
NA12760	reg_DEFB	chr8	7254738	A	G	138	28,00%	39	rs2740105			1,00E-012
NA12760	reg_DEFB	chr8	7254807	C	G	126	21,00%	26	rs2740741			1,00E-012
NA12760	reg_DEFB	chr8	7254922	G	A	126	30,00%	38	rs2740104			1,00E-012
NA12760	reg_DEFB	chr8	7255000	T	C	125	15,00%	19	rs2257803		T	1,02E-010
NA12760	reg_DEFB	chr8	7255018	G	A	132	11,00%	15	rs2257802			4,55E-007
NA12760	reg_DEFB	chr8	7255075	A	G	132	19,00%	25				1,00E-012
NA12760	reg_DEFB	chr8	7255159	G	A	133	32,00%	43	rs2740103			1,00E-012
NA12760	reg_DEFB	chr8	7255375	G	A	102	35,00%	36	rs2719462			1,00E-012
NA12760	reg_DEFB	chr8	7255556	T	G	97	26,00%	25			rs2698822	1,00E-012
NA12760	reg_DEFB	chr8	7255599	G	C	101	20,00%	20				1,00E-012
NA12760	reg_DEFB	chr8	7255820	G	T	119	18,00%	21			rs2737494	1,91E-012
NA12760	reg_DEFB	chr8	7255839	C	T	126	83,00%	105	rs3877964			1,00E-012

NA12760	reg_DEFB	chr8	7255879	G	A	131	18,00%	24			rs2719586	1,00E-012
NA12760	reg_DEFB	chr8	7255946	A	G	137	19,00%	26			rs2698821	1,00E-012
NA12760	reg_DEFB	chr8	7255958	T	C	137	14,00%	19	rs3877963			4,92E-010
NA12760	reg_DEFB	chr8	7255995	A	G	137	16,00%	22	rs2698820			4,22E-012
NA12760	reg_DEFB	chr8	7256236	A	G	125	14,00%	18			rs2257751	7,47E-010
NA12760	reg_DEFB	chr8	7256256	C	T	123	15,00%	18			rs2737497	5,71E-010
NA12760	reg_DEFB	chr8	7256331	C	G	121	32,00%	39	rs2719545			1,00E-012
NA12760	reg_DEFB	chr8	7256384	C	G	117	15,00%	18	rs6983800			2,54E-010
NA12760	reg_DEFB	chr8	7256473	T	C	114	16,00%	18	rs2257742			1,63E-010
NA12760	reg_DEFB	chr8	7256550	G	A	106	11,00%	12			rs3915496	6,50E-006
NA12760	reg_DEFB	chr8	7256564	A	G	102	15,00%	15	rs2740099			1,41E-008
NA12760	reg_DEFB	chr8	7256584	G	A	98	16,00%	16	rs2740098			9,75E-010
NA12760	reg_DEFB	chr8	7256617	G	A	93	99,00%	92	rs28576922	rs3866483		1,00E-012
NA12760	reg_DEFB	chr8	7256695	C	A	70	19,00%	13	rs2737917			7,03E-009
NA12760	reg_DEFB	chr8	7256705	A	C	64	17,00%	11				2,30E-007
NA12760	reg_DEFB	chr8	7256739	C	T	59	20,00%	12			rs7813724	8,98E-009
NA12760	reg_DEFB	chr8	7256789	A	G	42	17,00%	7	rs2740097			4,53E-005
NA12760	reg_DEFB	chr8	7256872	G	A	33	27,00%	9			rs71513067	4,21E-008
NA12760	reg_DEFB	chr8	7256897	T	C	35	17,00%	6	rs2737916			1,35E-004
NA12760	reg_DEFB	chr8	7257104	T	C	65	15,00%	10	rs2740095			2,33E-006
NA12760	reg_DEFB	chr8	7257126	G	A	69	85,00%	59	rs4840277			1,00E-012
NA12760	reg_DEFB	chr8	7257169	C	T	71	13,00%	9			rs55862841	3,70E-005
NA12760	reg_DEFB	chr8	7257175	G	A	69	14,00%	10			rs55897833	4,08E-006
NA12760	reg_DEFB	chr8	7257221	G	A	64	11,00%	7			rs73701026	6,71E-004
NA12760	reg_DEFB	chr8	7257305	C	A	79	15,00%	12			rs71247231	2,68E-007
NA12760	reg_DEFB	chr8	7257338	T	G	90	14,00%	13			rs56007428	1,58E-007
NA12760	reg_DEFB	chr8	7257342	C	A	92	15,00%	14			rs56035080	2,71E-008
NA12760	reg_DEFB	chr8	7257449	A	G	118	18,00%	21				1,00E-012
NA12760	reg_DEFB	chr8	7257475	T	A	130	18,00%	23				1,00E-012
NA12760	reg_DEFB	chr8	7257517	A	C	133	19,00%	25	rs2463965			1,00E-012
NA12760	reg_DEFB	chr8	7257543	C	T	140	14,00%	20				1,05E-010
NA12760	reg_DEFB	chr8	7257703	A	G	155	16,00%	25	rs2698818			1,00E-012
NA12760	reg_DEFB	chr8	7257705	T	A	153	16,00%	24	rs2737915			1,00E-012
NA12760	reg_DEFB	chr8	7257754	G	A	149	17,00%	25	rs2740093			1,00E-012
NA12760	reg_DEFB	chr8	7257896	G	C	162	11,00%	18	rs2740739			4,78E-008
NA12760	reg_DEFB	chr8	7258017	A	C	147	12,00%	18	rs2698817			1,04E-008
NA12760	reg_DEFB	chr8	7258022	A	G	149	13,00%	19				2,07E-009
NA12760	reg_DEFB	chr8	7258165	G	A	144	19,00%	27	rs2740092			1,00E-012
NA12760	reg_DEFB	chr8	7258243	C	G	146	12,00%	18	rs2737527			9,32E-009
NA12760	reg_DEFB	chr8	7258281	C	T	151	14,00%	21	rs2737528			6,12E-011
NA12760	reg_DEFB	chr8	7258328	T	C	143	14,00%	20	rs2740738			1,56E-010
NA12760	reg_DEFB	chr8	7258383	T	C	133	17,00%	23	rs3915367			1,00E-012
NA12760	reg_DEFB	chr8	7258399	G	A	137	14,00%	19			rs71251805	4,92E-010
NA12760	reg_DEFB	chr8	7258464	G	A	124	12,00%	15			rs72626630	2,00E-007
NA12760	reg_DEFB	chr8	7258546	C	T	124	12,00%	15	rs2737529			2,00E-007
NA12760	reg_DEFB	chr8	7258552	T	C	123	11,00%	14	rs2740737			1,07E-006
NA12760	reg_DEFB	chr8	7258587	T	C	123	44,00%	54	rs2737530			1,00E-012
NA12760	reg_DEFB	chr8	7259118	A	C	138	20,00%	28	rs2740736			1,00E-012
NA12760	reg_DEFB	chr8	7259185	G	T	123	18,00%	22	rs3901234			1,00E-012
NA12760	reg_DEFB	chr8	7259453	G	A	101	15,00%	15				1,23E-008
NA12760	reg_DEFB	chr8	7259539	G	A	77	16,00%	12	rs41390446			2,01E-007
NA12760	reg_DEFB	chr8	7259563	G	T	80	14,00%	11	rs41507446			2,31E-006
NA12760	reg_DEFB	chr8	7259739	C	T	81	48,00%	39	rs2737531			1,00E-012

NA12760	reg_DEFB	chr8	7259789	G	C	87	17,00%	15	rs2737912		1,47E-009
NA12760	reg_DEFB	chr8	7259849	G	A	83	22,00%	18	rs2740091		1,89E-012
NA12760	reg_DEFB	chr8	7260004	A	G	85	35,00%	30	rs2740090		1,00E-012
NA12760	reg_DEFB	chr8	7260322	T	C	58	24,00%	14		rs71251804	5,05E-011
NA12760	reg_DEFB	chr8	7261003	T	A	62	18,00%	11	rs2698815		1,64E-007
NA12760	reg_DEFB	chr8	7261175	C	T	102	16,00%	16			1,79E-009
NA12760	reg_DEFB	chr8	7261306	G	T	104	15,00%	16			2,40E-009
NA12760	reg_DEFB	chr8	7261330	G	A	104	13,00%	14			1,32E-007
NA12760	reg_DEFB	chr8	7261371	G	T	94	15,00%	14			3,59E-008
NA12760	reg_DEFB	chr8	7261420	G	T	79	23,00%	18	rs2740089		1,00E-012
NA12760	reg_DEFB	chr8	7261718	A	G	62	97,00%	60		rs71509106	1,00E-012
NA12760	reg_DEFB	chr8	7261867	A	G	62	23,00%	14	rs2740086		1,34E-010
NA12760	reg_DEFB	chr8	7262028	C	G	52	40,00%	21	rs2698827		1,00E-012
NA12760	reg_DEFB	chr8	7262234	G	T	53	26,00%	14	rs2698828		1,31E-011
NA12760	reg_DEFB	chr8	7262270	C	T	50	22,00%	11			1,55E-008
NA12760	reg_DEFB	chr8	7262634	C	T	47	30,00%	14	rs2737535		4,96E-012
NA12760	reg_DEFB	chr8	7262672	G	A	43	16,00%	7		rs71513122	5,30E-005
NA12760	reg_DEFB	chr8	7262683	G	A	43	21,00%	9	rs3762041		5,00E-007
NA12760	reg_DEFB	chr8	7262687	C	T	44	23,00%	10			5,02E-008
NA12760	reg_DEFB	chr8	7262787	C	T	57	19,00%	11	rs3762040		6,62E-008
NA12760	reg_DEFB	chr8	7262795	C	A	59	19,00%	11	rs3762039		9,63E-008
NA12760	reg_DEFB	chr8	7262812	C	T	58	12,00%	7			3,66E-004
NA12760	reg_DEFB	chr8	7263102	T	C	51	80,00%	41	rs4840278		1,00E-012
NA12760	reg_DEFB	chr8	7263191	G	C	46	26,00%	12	rs3762052		4,14E-010
NA12760	reg_DEFB	chr8	7263345	G	A	36	69,00%	25	rs4840751		1,00E-012
NA12760	reg_DEFB	chr8	7263349	G	A	38	16,00%	6	rs3762051		2,17E-004
NA12760	reg_DEFB	chr8	7263407	A	G	40	17,00%	7		rs71244071	3,26E-005
NA12760	reg_DEFB	chr8	7263412	G	A	41	22,00%	9	rs2698830		3,24E-007
NA12760	reg_DEFB	chr8	7263419	A	G	45	27,00%	12	rs2737910		3,12E-010
NA12760	reg_DEFB	chr8	7263436	G	A	48	62,00%	30	rs4840752		1,00E-012
NA12760	reg_DEFB	chr8	7263531	T	C	57	23,00%	13			4,82E-010
NA12760	reg_DEFB	chr8	7263647	A	G	59	22,00%	13			7,64E-010
NA12760	reg_DEFB	chr8	7263683	T	C	55	18,00%	10			4,70E-007
NA12760	reg_DEFB	chr8	7263744	G	A	64	27,00%	17	rs2740083		1,00E-012
NA12760	reg_DEFB	chr8	7263764	G	C	68	16,00%	11	rs2409862		4,36E-007
NA12760	reg_DEFB	chr8	7263877	A	C	54	100,00%	54	rs6651513		1,00E-012
NA12760	reg_DEFB	chr8	7263885	C	T	53	13,00%	7	rs3762038		2,08E-004
NA12760	reg_DEFB	chr8	7264091	G	A	76	20,00%	15	rs3762037		2,04E-010
NA12760	reg_DEFB	chr8	7264095	A	G	77	22,00%	17	rs3988844		5,67E-012
NA12760	reg_DEFB	chr8	7264156	G	C	79	53,00%	42	rs2737536		1,00E-012
NA12760	reg_DEFB	chr8	7264462	A	C	96	12,00%	12		rs71254898	2,28E-006
NA12760	reg_DEFB	chr8	7264470	A	T	95	17,00%	16	rs2737909		6,05E-010
NA12760	reg_DEFB	chr8	7264552	A	C	97	20,00%	19	rs2737538		3,14E-012
NA12760	reg_DEFB	chr8	7264604	G	A	100	17,00%	17		rs71513120	1,56E-010
NA12760	reg_DEFB	chr8	7264610	T	C	98	15,00%	15			8,04E-009
NA12760	reg_DEFB	chr8	7264753	G	C	83	22,00%	18		rs71537819	1,89E-012
NA12760	reg_DEFB	chr8	7264799	C	T	84	19,00%	16	rs3866482		9,04E-011
NA12760	reg_DEFB	chr8	7264836	T	G	76	20,00%	15	rs4118281		2,04E-010
NA12760	reg_DEFB	chr8	7264944	G	C	69	72,00%	50	rs4840753		1,00E-012
NA12760	reg_DEFB	chr8	7264948	G	A	69	17,00%	12	rs3866481		5,71E-008
NA12760	reg_DEFB	chr8	7265160	T	C	53	13,00%	7		rs71513119	2,08E-004
NA12760	reg_DEFB	chr8	7265254	T	C	59	15,00%	9		rs71513117	8,00E-006
NA12760	reg_DEFB	chr8	7265340	T	C	60	17,00%	10	rs2698832		1,09E-006

NA12760	reg_DEFB	chr8	7265354	C	T	61	15,00%	9				1,06E-005
NA12760	reg_DEFB	chr8	7265410	G	A	74	14,00%	10			rs71513116	7,76E-006
NA12760	reg_DEFB	chr8	7265947	G	T	116	14,00%	16	rs3988845			1,21E-008
NA12760	reg_DEFB	chr8	7265983	G	A	123	17,00%	21	rs3866480			3,83E-012
NA12760	reg_DEFB	chr8	7266035	T	C	128	14,00%	18	rs3988846			1,11E-009
NA12760	reg_DEFB	chr8	7266053	C	T	134	31,00%	42	rs2740731			1,00E-012
NA12760	reg_DEFB	chr8	7266297	C	A	116	20,00%	23	rs3988829			1,00E-012
NA12760	reg_DEFB	chr8	7266317	C	T	112	20,00%	22	rs3988830			1,00E-012
NA12760	reg_DEFB	chr8	7266358	C	T	99	20,00%	20	rs3988831			1,00E-012
NA12760	reg_DEFB	chr8	7266438	A	C	90	20,00%	18	rs3988832			8,42E-012
NA12760	reg_DEFB	chr8	7266500	G	A	93	14,00%	13	rs746659			2,35E-007
NA12760	reg_DEFB	chr8	7266687	C	T	80	31,00%	25	rs2740730			1,00E-012
NA12760	reg_DEFB	chr8	7266786	T	C	79	11,00%	9			rs71513115	8,67E-005
NA12760	reg_DEFB	chr8	7266858	C	T	93	13,00%	12			rs71513114	1,62E-006
NA12760	reg_DEFB	chr8	7266868	G	T	99	17,00%	17				1,33E-010
NA12760	reg_DEFB	chr8	7267031	T	G	92	100,00%	92	rs7815816			1,00E-012
NA12760	reg_DEFB	chr8	7267055	A	C	86	21,00%	18			rs71513113	3,72E-012
NA12760	reg_DEFB	chr8	7267305	C	G	70	20,00%	14	rs2737543			6,76E-010
NA12760	reg_DEFB	chr8	7267395	G	T	60	12,00%	7			rs71513110	4,52E-004
NA12760	reg_DEFB	chr8	7267436	A	C	65	15,00%	10			rs71513109	2,33E-006
NA12760	reg_DEFB	chr8	7267461	T	G	60	15,00%	9			rs71513108	9,22E-006
NA12760	reg_DEFB	chr8	7267609	T	G	53	15,00%	8			rs71513107	2,76E-005
NA12760	reg_DEFB	chr8	7267636	C	G	53	19,00%	10			rs71513106	3,27E-007
NA12760	reg_DEFB	chr8	7267855	A	G	68	78,00%	53	rs3958991			1,00E-012
NA12760	reg_DEFB	chr8	7267995	T	C	78	17,00%	13	rs2740729			2,75E-008
NA12760	reg_DEFB	chr8	7268025	T	A	89	20,00%	18	rs28610011	rs3866479		6,89E-012
NA12760	reg_DEFB	chr8	7268520	A	T	116	21,00%	24			rs71513105	1,00E-012
NA12760	reg_DEFB	chr8	7268623	G	T	113	63,00%	71	rs4840754			1,00E-012
NA12760	reg_DEFB	chr8	7268975	C	T	105	14,00%	15	rs3958992			2,10E-008
NA12760	reg_DEFB	chr8	7269055	T	C	101	69,00%	70				1,00E-012
NA12760	reg_DEFB	chr8	7269068	G	C	103	17,00%	18	rs2737546			3,44E-011
NA12760	reg_DEFB	chr8	7269085	G	T	101	65,00%	66				1,00E-012
NA12760	reg_DEFB	chr8	7269368	T	C	70	61,00%	43			rs71511302	1,00E-012
NA12760	reg_DEFB	chr8	7269455	T	C	68	24,00%	16			rs2698835	8,17E-012
NA12760	reg_DEFB	chr8	7269776	G	A	113	46,00%	52			rs2737547	1,00E-012
NA12760	reg_DEFB	chr8	7269817	C	A	116	97,00%	113			rs62636842	1,00E-012
NA12760	reg_DEFB	chr8	7269942	G	T	125	100,00%	125				1,00E-012
NA12760	reg_DEFB	chr8	7269967	T	C	124	98,00%	122				1,00E-012
NA12760	reg_DEFB	chr8	7269989	G	T	124	20,00%	25			rs9720715	1,00E-012
NA12760	reg_DEFB	chr8	7270164	G	A	128	15,00%	19				1,55E-010
NA12760	reg_DEFB	chr8	7270168	G	T	127	65,00%	83				1,00E-012
NA12760	reg_DEFB	chr8	7270508	C	T	149	17,00%	25			rs71511299	1,00E-012
NA12760	reg_DEFB	chr8	7270703	T	C	121	42,00%	51			rs71511298	1,00E-012
NA12760	reg_DEFB	chr8	7270806	C	T	91	20,00%	18	rs2740728			1,03E-011
NA12760	reg_DEFB	chr8	7270961	C	T	63	25,00%	16	rs2740727			2,20E-012
NA12760	reg_DEFB	chr8	7270994	G	A	61	26,00%	16	rs2737548			1,00E-012
NA12760	reg_DEFB	chr8	7271125	G	A	59	19,00%	11	rs2737907			9,63E-008
NA12760	reg_DEFB	chr8	7271226	C	A	59	42,00%	25				1,00E-012
NA12760	reg_DEFB	chr8	7271434	T	C	79	82,00%	65			rs4543549	1,00E-012
NA12760	reg_DEFB	chr8	7271577	A	G	92	11,00%	10			rs11986964	5,35E-005
NA12760	reg_DEFB	chr8	7271654	T	C	119	19,00%	23				1,00E-012
NA12760	reg_DEFB	chr8	7271670	G	C	121	16,00%	19			rs62636843	5,66E-011
NA12760	reg_DEFB	chr8	7271706	G	A	133	17,00%	23			rs62636844	1,00E-012

NA12760	reg_DEFB	chr8	7271837	C	T	144	56,00%	81				rs62636845	1,00E-012
NA12760	reg_DEFB	chr8	7271938	C	T	145	81,00%	117				rs62636846	1,00E-012
NA12760	reg_DEFB	chr8	7272539	G	T	100	13,00%	13			rs2737904		5,53E-007
NA12760	reg_DEFB	chr8	7272799	T	G	137	26,00%	36			rs2737549		1,00E-012
NA12760	reg_DEFB	chr8	7272812	G	T	138	51,00%	70			rs3988903		1,00E-012
NA12760	reg_DEFB	chr8	7272893	G	A	150	83,00%	125			rs3988902		1,00E-012
NA12760	reg_DEFB	chr8	7272905	G	C	147	99,00%	146				rs71509107	1,00E-012
NA12760	reg_DEFB	chr8	7272963	A	G	142	23,00%	33				rs71511249	1,00E-012
NA12760	reg_DEFB	chr8	7273000	T	A	142	23,00%	33			rs2737550		1,00E-012
NA12760	reg_DEFB	chr8	7273160	G	A	96	31,00%	30			rs3988900		1,00E-012
NA12760	reg_DEFB	chr8	7273179	A	G	89	12,00%	11			rs2740725		6,67E-006
NA12760	reg_DEFB	chr8	7273532	G	C	75	55,00%	41			rs3866478		1,00E-012
NA12760	reg_DEFB	chr8	7274151	C	T	106	66,00%	70	-	DEFB103B		rs62636848	1,00E-012
NA12760	reg_DEFB	chr8	7274285	G	T	104	17,00%	18	-	DEFB103B			4,09E-011
NA12760	reg_DEFB	chr8	7274449	C	A	78	12,00%	9	-	DEFB103B		rs62636849	7,85E-005
NA12760	reg_DEFB	chr8	7274576	T	C	40	50,00%	20	-	DEFB103B	rs3789865		1,00E-012
NA12760	reg_DEFB	chr8	7274787	G	C	49	29,00%	14	-	DEFB103B	rs3789862		9,40E-012
NA12760	reg_DEFB	chr8	7274794	G	A	51	20,00%	10	-	DEFB103B		rs73356491	2,23E-007
NA12760	reg_DEFB	chr8	7274798	A	G	51	14,00%	7	-	DEFB103B		rs72709834	1,62E-004
NA12760	reg_DEFB	chr8	7274817	T	C	52	15,00%	8	-	DEFB103B		rs71553678	2,39E-005
NA12760	reg_DEFB	chr8	7274831	A	G	55	13,00%	7	-	DEFB103B		rs13256156	2,62E-004
NA12760	reg_DEFB	chr8	7275507	G	T	72	17,00%	12				rs71511250	9,34E-008
NA12760	reg_DEFB	chr8	7275590	A	G	56	21,00%	12			rs4461923		4,78E-009
NA12760	reg_DEFB	chr8	7275809	A	G	31	84,00%	26				rs71511253	1,00E-012
NA12760	reg_DEFB	chr8	7275837	T	A	28	18,00%	5			rs13439779		4,06E-004
NA12760	reg_DEFB	chr8	7275956	G	A	48	21,00%	10					1,22E-007
NA12760	reg_DEFB	chr8	7276019	A	G	62	23,00%	14			rs4347011		1,34E-010
NA12760	reg_DEFB	chr8	7276276	A	T	117	27,00%	32			rs3762049		1,00E-012
NA12760	reg_DEFB	chr8	7276298	A	G	116	16,00%	19			rs2737902		3,30E-011
NA12760	reg_DEFB	chr8	7276309	G	T	118	12,00%	14			rs2737901		6,41E-007
NA12760	reg_DEFB	chr8	7276674	A	C	115	70,00%	81			rs2737554		1,00E-012
NA12760	reg_DEFB	chr8	7277001	G	A	75	19,00%	14			rs3762046		1,74E-009
NA12760	reg_DEFB	chr8	7277359	T	C	95	69,00%	66				rs71509108	1,00E-012
NA12760	reg_DEFB	chr8	7277387	T	C	92	36,00%	33				rs71511255	1,00E-012
NA12760	reg_DEFB	chr8	7277422	T	A	95	74,00%	70				rs71509109	1,00E-012
NA12760	reg_DEFB	chr8	7277424	C	A	94	74,00%	70				rs71509110	1,00E-012
NA12760	reg_DEFB	chr8	7277451	C	G	100	24,00%	24				rs71511256	1,00E-012
NA12760	reg_DEFB	chr8	7277460	G	A	103	25,00%	26				rs71511257	1,00E-012
NA12760	reg_DEFB	chr8	7277507	T	C	102	48,00%	49					1,00E-012
NA12760	reg_DEFB	chr8	7277521	C	G	105	30,00%	32				rs71511259	1,00E-012
NA12760	reg_DEFB	chr8	7277545	C	T	107	25,00%	27				rs71511260	1,00E-012
NA12760	reg_DEFB	chr8	7277566	G	A	110	25,00%	28				rs71509111	1,00E-012
NA12760	reg_DEFB	chr8	7277662	A	G	113	27,00%	31				rs4999975	1,00E-012
NA12760	reg_DEFB	chr8	7277710	T	C	106	29,00%	31				rs4999974	1,00E-012
NA12760	reg_DEFB	chr8	7277745	A	T	99	22,00%	22				rs62636851	1,00E-012
NA12760	reg_DEFB	chr8	7277755	T	C	103	32,00%	33			rs4999973		1,00E-012
NA12760	reg_DEFB	chr8	7277810	A	G	102	30,00%	31					1,00E-012
NA12760	reg_DEFB	chr8	7277830	C	G	104	30,00%	31					1,00E-012
NA12760	reg_DEFB	chr8	7278010	C	T	112	81,00%	91					1,00E-012
NA12760	reg_DEFB	chr8	7278055	C	T	120	16,00%	19			rs2698834		4,87E-011
NA12760	reg_DEFB	chr8	7278116	C	T	128	32,00%	41				rs4840303	1,00E-012
NA12760	reg_DEFB	chr8	7278208	T	C	138	18,00%	25					1,00E-012
NA12760	reg_DEFB	chr8	7278542	T	A	144	88,00%	127			rs41380147		1,00E-012

NA12760	reg_DEFB	chr8	7278548	A	C	144	33,00%	48		rs4840304	1,00E-012
NA12760	reg_DEFB	chr8	7278563	G	T	144	32,00%	46		rs9693075	1,00E-012
NA12760	reg_DEFB	chr8	7278594	T	C	147	84,00%	123			1,00E-012
NA12760	reg_DEFB	chr8	7278660	C	A	165	30,00%	50		rs71276790	1,00E-012
NA12760	reg_DEFB	chr8	7278727	C	T	168	30,00%	50		rs71276791	1,00E-012
NA12760	reg_DEFB	chr8	7278763	C	T	175	27,00%	47		rs71276792	1,00E-012
NA12760	reg_DEFB	chr8	7278790	G	T	172	24,00%	41			1,00E-012
NA12760	reg_DEFB	chr8	7278926	A	G	176	29,00%	51		rs71276794	1,00E-012
NA12760	reg_DEFB	chr8	7279069	A	C	160	21,00%	34		rs71276795	1,00E-012
NA12760	reg_DEFB	chr8	7279284	C	A	100	25,00%	25	rs2463968		1,00E-012
NA12760	reg_DEFB	chr8	7279289	T	C	97	80,00%	78			1,00E-012
NA12760	reg_DEFB	chr8	7279315	T	C	85	81,00%	69			1,00E-012
NA12760	reg_DEFB	chr8	7279414	G	A	76	39,00%	30		rs4840836	1,00E-012
NA12760	reg_DEFB	chr8	7279450	C	T	77	32,00%	25	rs4840306		1,00E-012
NA12760	reg_DEFB	chr8	7279477	A	G	76	37,00%	28		rs4840307	1,00E-012
NA12760	reg_DEFB	chr8	7279596	C	G	83	31,00%	26		rs4840308	1,00E-012
NA12760	reg_DEFB	chr8	7279621	C	G	92	29,00%	27		rs4840837	1,00E-012
NA12760	reg_DEFB	chr8	7279696	A	T	110	44,00%	48	rs2737899		1,00E-012
NA12760	reg_DEFB	chr8	7279780	C	G	107	61,00%	65		rs71276797	1,00E-012
NA12760	reg_DEFB	chr8	7279877	C	T	105	55,00%	58		rs71276798	1,00E-012
NA12760	reg_DEFB	chr8	7279897	C	G	106	15,00%	16			3,19E-009
NA12760	reg_DEFB	chr8	7280090	T	C	83	83,00%	69			1,00E-012
NA12760	reg_DEFB	chr8	7280221	C	T	61	52,00%	32		rs71299143	1,00E-012
NA12760	reg_DEFB	chr8	7280227	A	T	63	81,00%	51			1,00E-012
NA12760	reg_DEFB	chr8	7280259	T	G	59	27,00%	16	rs2698836		1,00E-012
NA12760	reg_DEFB	chr8	7280364	G	C	65	68,00%	44		rs2737897	1,00E-012
NA12760	reg_DEFB	chr8	7280790	G	A	87	25,00%	22	rs2737896		1,00E-012
NA12760	reg_DEFB	chr8	7281118	T	C	69	16,00%	11	rs2737895		5,08E-007
NA12760	reg_DEFB	chr8	7281292	C	T	61	80,00%	49	rs4840279		1,00E-012
NA12760	reg_DEFB	chr8	7281324	C	A	55	15,00%	8	rs2740075		3,63E-005
NA12760	reg_DEFB	chr8	7281372	T	G	58	53,00%	31	rs4247403		1,00E-012
NA12760	reg_DEFB	chr8	7281483	A	T	50	18,00%	9	rs2740074		1,92E-006
NA12760	reg_DEFB	chr8	7281562	C	T	70	40,00%	28		rs2737894	1,00E-012
NA12760	reg_DEFB	chr8	7281568	A	T	71	18,00%	13	rs2740073		8,42E-009
NA12760	reg_DEFB	chr8	7281649	T	A	66	23,00%	15		rs71509112	2,67E-011
NA12760	reg_DEFB	chr8	7281710	G	T	61	57,00%	35	rs2698838		1,00E-012
NA12760	reg_DEFB	chr8	7282390	A	C	86	19,00%	16			1,31E-010
NA12760	reg_DEFB	chr8	7282443	T	C	96	80,00%	77			1,00E-012
NA12760	reg_DEFB	chr8	7282482	G	A	95	20,00%	19		rs2698840	2,11E-012
NA12760	reg_DEFB	chr8	7282560	C	T	101	29,00%	29		rs71267735	1,00E-012
NA12760	reg_DEFB	chr8	7282592	G	A	100	31,00%	31		rs71267736	1,00E-012
NA12760	reg_DEFB	chr8	7282670	T	C	109	86,00%	94			1,00E-012
NA12760	reg_DEFB	chr8	7282719	A	G	114	12,00%	14		rs2737556	4,19E-007
NA12760	reg_DEFB	chr8	7282985	T	A	115	23,00%	26		rs2737892	1,00E-012
NA12760	reg_DEFB	chr8	7283081	C	T	101	29,00%	29	rs3915374		1,00E-012
NA12760	reg_DEFB	chr8	7283244	C	A	66	21,00%	14		rs2740070	2,94E-010
NA12760	reg_DEFB	chr8	7283283	C	T	64	42,00%	27		rs71267737	1,00E-012
NA12760	reg_DEFB	chr8	7283305	A	G	68	84,00%	57			1,00E-012
NA12760	reg_DEFB	chr8	7283750	C	T	87	24,00%	21		rs62636852	1,00E-012
NA12760	reg_DEFB	chr8	7283805	C	T	120	17,00%	20			7,22E-012
NA12760	reg_DEFB	chr8	7283828	C	G	127	32,00%	41	rs3915371		1,00E-012
NA12760	reg_DEFB	chr8	7283981	G	A	127	33,00%	42		rs3915370	1,00E-012
NA12760	reg_DEFB	chr8	7284058	A	G	126	16,00%	20			1,87E-011

NA12760	reg_DEFB	chr8	7284122	A	G	119	16,00%	19			rs2698841			4,19E-011
NA12760	reg_DEFB	chr8	7284285	G	A	75	29,00%	22			rs2698842			1,00E-012
NA12760	reg_DEFB	chr8	7284343	T	G	66	21,00%	14						2,94E-010
NA12760	reg_DEFB	chr8	7284450	G	A	54	17,00%	9						3,75E-006
NA12760	reg_DEFB	chr8	7285027	C	T	119	34,00%	40			rs2740069			1,00E-012
NA12760	reg_DEFB	chr8	7285028	A	G	118	54,00%	64			rs2740069			1,00E-012
NA12760	reg_DEFB	chr8	7285076	C	T	104	20,00%	21			rs2698844			1,00E-012
NA12760	reg_DEFB	chr8	7285531	G	C	76	43,00%	33			rs2740068	rs2737887		1,00E-012
NA12760	reg_DEFB	chr8	7285565	G	T	72	18,00%	13			rs2737886			1,01E-008
NA12760	reg_DEFB	chr8	7285576	C	G	72	22,00%	16			rs2740067			2,16E-011
NA12760	reg_DEFB	chr8	7285682	T	C	74	18,00%	13					rs2698845	1,42E-008
NA12760	reg_DEFB	chr8	7285744	C	T	74	78,00%	58			rs3988838			1,00E-012
NA12760	reg_DEFB	chr8	7285834	T	A	66	62,00%	41			rs4501606			1,00E-012
NA12760	reg_DEFB	chr8	7285981	C	G	58	72,00%	42					rs3988839	1,00E-012
NA12760	reg_DEFB	chr8	7286036	C	T	60	33,00%	20					rs71267752	1,00E-012
NA12760	reg_DEFB	chr8	7286545	G	A	89	84,00%	75			rs2280948			1,00E-012
NA12760	reg_DEFB	chr8	7286699	A	T	98	33,00%	32			rs2280947			1,00E-012
NA12760	reg_DEFB	chr8	7286812	G	A	92	30,00%	28			rs2280946			1,00E-012
NA12760	reg_DEFB	chr8	7286995	G	C	74	57,00%	42			rs2280945			1,00E-012
NA12760	reg_DEFB	chr8	7287010	G	A	74	20,00%	15			rs2280944			1,37E-010
NA12760	reg_DEFB	chr8	7287421	C	G	40	20,00%	8					rs2740065	3,12E-006
NA12760	reg_DEFB	chr8	7287713	T	C	36	58,00%	21						1,00E-012
NA12760	reg_DEFB	chr8	7287838	G	T	31	26,00%	8					rs71267755	3,85E-007
NA12760	reg_DEFB	chr8	7288153	G	A	36	19,00%	7			rs2740062			1,58E-005
NA12760	reg_DEFB	chr8	7288182	G	A	39	28,00%	11			rs2698848			8,84E-010
NA12760	reg_DEFB	chr8	7288411	T	A	57	19,00%	11			rs2740061			6,62E-008
NA12760	reg_DEFB	chr8	7288723	A	G	73	16,00%	12			rs2740060			1,09E-007
NA12760	reg_DEFB	chr8	7288985	T	C	82	61,00%	50			rs4466423			1,00E-012
NA12760	reg_DEFB	chr8	7289081	C	G	89	18,00%	16						2,25E-010
NA12760	reg_DEFB	chr8	7289545	G	A	83	65,00%	54			rs2740059			1,00E-012
NA12760	reg_DEFB	chr8	7289788	C	T	71	17,00%	12			rs2698849			7,95E-008
NA12760	reg_DEFB	chr8	7290162	T	G	39	64,00%	25			rs2698850			1,00E-012
NA12760	reg_DEFB	chr8	7290257	G	A	48	12,00%	6			rs2740057			7,93E-004
NA12760	reg_DEFB	chr8	7290567	C	T	72	21,00%	15			rs2740056			1,03E-010
NA12760	reg_DEFB	chr8	7290634	G	A	64	14,00%	9			rs2740055			1,58E-005
NA12760	reg_DEFB	chr8	7290637	G	C	64	14,00%	9			rs2740054			1,58E-005
NA12760	reg_DEFB	chr8	7290646	G	A	63	59,00%	37			rs2740053			1,00E-012
NA12760	reg_DEFB	chr8	7290832	C	T	44	16,00%	7					rs3110053	6,18E-005
NA12760	reg_DEFB	chr8	7290907	A	G	52	15,00%	8			rs2740052			2,39E-005
NA12760	reg_DEFB	chr8	7291101	G	A	102	25,00%	26					rs71267758	1,00E-012
NA12760	reg_DEFB	chr8	7291778	C	T	53	17,00%	9			rs2740050			3,19E-006
NA12760	reg_DEFB	chr8	7291811	C	T	50	38,00%	19					rs71267759	1,00E-012
NA12760	reg_DEFB	chr8	7291975	G	T	67	31,00%	21					rs71267760	1,00E-012
NA12760	reg_DEFB	chr8	7292033	G	C	64	31,00%	20					rs71267761	1,00E-012
NA12760	reg_DEFB	chr8	7292224	G	A	60	12,00%	7						4,52E-004
NA12760	reg_DEFB	chr8	7292386	T	C	48	33,00%	16					rs71267762	1,00E-012
NA12760	reg_DEFB	chr8	7292398	G	C	48	15,00%	7			rs2698915			1,10E-004
NA12760	reg_DEFB	chr8	7292605	G	C	30	27,00%	8					rs71267763	2,91E-007
NA12760	reg_DEFB	chr8	7292760	G	A	35	26,00%	9	-	SPAG11B	rs2740048			7,40E-008
NA12760	reg_DEFB	chr8	7292773	T	C	37	100,00%	37	-	SPAG11B			rs4626629	1,00E-012
NA12760	reg_DEFB	chr8	7292820	C	G	39	77,00%	30	-	SPAG11B			rs2740047	1,00E-012
NA12760	reg_DEFB	chr8	7292896	A	C	36	100,00%	36	-	SPAG11B	rs3901154			1,00E-012
NA12760	reg_DEFB	chr8	7293040	A	C	30	20,00%	6	-	SPAG11B	rs2740046			5,46E-005

NA12760	reg_DEFB	chr8	7293741	A	G	7	100,00%	7			-	SPAG11B		rs2740041	3,40E-012
NA12760	reg_DEFB	chr8	7293835	G	A	13	62,00%	8			-	SPAG11B	rs2740040		9,13E-011
NA12760	reg_DEFB	chr8	7293918	T	A	19	42,00%	8			-	SPAG11B		rs71511262	4,72E-009
NA12760	reg_DEFB	chr8	7294380	G	C	43	28,00%	12			-	SPAG11B	rs2737557		1,88E-010
NA12760	reg_DEFB	chr8	7294480	A	C	53	32,00%	17			-	SPAG11B		rs71511263	1,00E-012
NA12760	reg_DEFB	chr8	7294502	C	G	62	45,00%	28			-	SPAG11B	rs2853664		1,00E-012
NA12760	reg_DEFB	chr8	7294800	C	T	107	48,00%	51			-	SPAG11B	rs2853663		1,00E-012
NA12760	reg_DEFB	chr8	7295003	A	G	141	78,00%	110			-	SPAG11B	rs2853661		1,00E-012
NA12760	reg_DEFB	chr8	7295159	G	C	123	100,00%	123			-	SPAG11B		rs3915372	1,00E-012
NA12760	reg_DEFB	chr8	7295311	T	A	123	46,00%	57			-	SPAG11B	rs2853660		1,00E-012
NA12760	reg_DEFB	chr8	7295321	A	T	123	50,00%	62			-	SPAG11B	rs2737558		1,00E-012
NA12760	reg_DEFB	chr8	7295346	G	A	115	15,00%	17			-	SPAG11B			1,45E-009
NA12760	reg_DEFB	chr8	7295375	G	A	114	17,00%	19			-	SPAG11B	rs4089926		2,39E-011
NA12760	reg_DEFB	chr8	7295796	T	C	99	85,00%	84	H	R	-2	SPAG11B	rs1042797		1,00E-012
NA12760	reg_DEFB	chr8	7295813	A	C	97	24,00%	23	I	M	-2	SPAG11B	rs12063		1,00E-012
NA12760	reg_DEFB	chr8	7295820	G	A	97	43,00%	42	P	L	-2	SPAG11B	rs2256100		1,00E-012
NA12760	reg_DEFB	chr8	7295894	C	A	90	46,00%	41			-	SPAG11B	rs2853659		1,00E-012
NA12760	reg_DEFB	chr8	7295903	G	A	87	45,00%	39			-	SPAG11B	rs2738036		1,00E-012
NA12760	reg_DEFB	chr8	7295939	C	T	83	40,00%	33			-	SPAG11B		rs2737559	1,00E-012
NA12760	reg_DEFB	chr8	7296080	T	C	80	44,00%	35	D	G	-2	SPAG11B	rs2738035		1,00E-012
NA12760	reg_DEFB	chr8	7296085	A	G	82	44,00%	36	C	C	-2	SPAG11B		rs61749560	1,00E-012
NA12760	reg_DEFB	chr8	7296092	C	T	83	45,00%	37	R	K	-2	SPAG11B		rs61749561	1,00E-012
NA12760	reg_DEFB	chr8	7296116	C	T	86	43,00%	37	R	Q	-2	SPAG11B	rs2853658		1,00E-012
NA12760	reg_DEFB	chr8	7296139	G	C	90	33,00%	30			-	SPAG11B	rs2294141		1,00E-012
NA12760	reg_DEFB	chr8	7296208	A	G	92	96,00%	88			-	SPAG11B	rs4840280		1,00E-012
NA12760	reg_DEFB	chr8	7296408	T	C	97	40,00%	39			-	SPAG11B	rs12682529		1,00E-012
NA12760	reg_DEFB	chr8	7297111	T	C	106	23,00%	24			-	SPAG11B	rs2853656		1,00E-012
NA12760	reg_DEFB	chr8	7297409	G	T	91	18,00%	16			-	SPAG11B	rs2738034		3,18E-010
NA12760	reg_DEFB	chr8	7297513	G	A	109	19,00%	21			-	SPAG11B	rs2738033		1,00E-012
NA12760	reg_DEFB	chr8	7297645	A	T	105	29,00%	30			-	SPAG11B		rs71511266	1,00E-012
NA12760	reg_DEFB	chr8	7297906	C	T	103	14,00%	14			-	SPAG11B	rs2738032		1,17E-007
NA12760	reg_DEFB	chr8	7297910	G	A	103	16,00%	16			-	SPAG11B	rs2853654		2,07E-009
NA12760	reg_DEFB	chr8	7297951	A	G	96	18,00%	17			-	SPAG11B	rs2740034		7,97E-011
NA12760	reg_DEFB	chr8	7298154	C	T	65	11,00%	7			-	SPAG11B		rs4840309	7,38E-004
NA12760	reg_DEFB	chr8	7298212	T	G	73	48,00%	35			-	SPAG11B	rs2738031		1,00E-012
NA12760	reg_DEFB	chr8	7298298	T	A	86	15,00%	13			-	SPAG11B	rs2738030		9,13E-008
NA12760	reg_DEFB	chr8	7298320	A	G	97	14,00%	14			-	SPAG11B	rs2740033		5,40E-008
NA12760	reg_DEFB	chr8	7298410	C	A	119	13,00%	15			-	SPAG11B	rs2738029		1,16E-007
NA12760	reg_DEFB	chr8	7298525	T	G	110	98,00%	108			-	SPAG11B		rs62636856	1,00E-012
NA12760	reg_DEFB	chr8	7298554	T	C	112	16,00%	18			-	SPAG11B			1,20E-010
NA12760	reg_DEFB	chr8	7298614	T	C	107	12,00%	13			-	SPAG11B	rs2853653		1,21E-006
NA12760	reg_DEFB	chr8	7298676	G	T	91	38,00%	35			-	SPAG11B		rs71511269	1,00E-012
NA12760	reg_DEFB	chr8	7298691	T	G	86	41,00%	35			-	SPAG11B		rs71511270	1,00E-012
NA12760	reg_DEFB	chr8	7298786	T	C	70	23,00%	16			-	SPAG11B	rs2738027		1,34E-011
NA12760	reg_DEFB	chr8	7298840	C	T	60	22,00%	13			-	SPAG11B		rs72056310	9,56E-010
NA12760	reg_DEFB	chr8	7299088	C	A	75	36,00%	27			-	SPAG11B		rs71235968	1,00E-012
NA12760	reg_DEFB	chr8	7299344	T	G	117	15,00%	18			-	SPAG11B	rs2737562		2,54E-010
NA12760	reg_DEFB	chr8	7299529	T	C	123	85,00%	105			-	SPAG11B	rs2738025		1,00E-012
NA12760	reg_DEFB	chr8	7299717	G	C	121	18,00%	22			-	SPAG11B	rs2738024		1,00E-012
NA12760	reg_DEFB	chr8	7299852	A	T	102	18,00%	18			-	SPAG11B		rs71235967	2,90E-011
NA12760	reg_DEFB	chr8	7300402	A	G	68	16,00%	11			-	SPAG11B	rs2266517		4,36E-007
NA12760	reg_DEFB	chr8	7300520	T	C	93	20,00%	19			-	SPAG11B			1,00E-012
NA12760	reg_DEFB	chr8	7300697	C	T	116	13,00%	15			-	SPAG11B	rs2738021		8,21E-008

NA12760	reg_DEFB	chr8	7300781	A	G	108	11,00%	12	-	SPAG11B	rs2740032		7,90E-006
NA12760	reg_DEFB	chr8	7301157	C	G	78	36,00%	28	-	SPAG11B		rs71235965	1,00E-012
NA12760	reg_DEFB	chr8	7301232	G	A	77	16,00%	12	-	SPAG11B		rs71235964	2,01E-007
NA12760	reg_DEFB	chr8	7301270	A	G	75	25,00%	19	-	SPAG11B		rs71235963	1,00E-012
NA12760	reg_DEFB	chr8	7301410	G	T	59	44,00%	26	-	SPAG11B		rs71235962	1,00E-012
NA12760	reg_DEFB	chr8	7301857	A	G	41	83,00%	34	-	SPAG11B	rs2740031		1,00E-012
NA12760	reg_DEFB	chr8	7301960	T	G	48	12,00%	6	-	SPAG11B		rs55978454	7,93E-004
NA12760	reg_DEFB	chr8	7301986	G	A	49	96,00%	47	-	SPAG11B	rs2738017		1,00E-012
NA12760	reg_DEFB	chr8	7302094	G	A	57	28,00%	16	-	SPAG11B		rs71235961	1,00E-012
NA12760	reg_DEFB	chr8	7302098	C	T	57	23,00%	13	-	SPAG11B		rs2740718	4,82E-010
NA12760	reg_DEFB	chr8	7302236	T	C	62	34,00%	21	-	SPAG11B		rs71235960	1,00E-012
NA12760	reg_DEFB	chr8	7302765	C	T	73	62,00%	45	-	SPAG11B	rs4840756		1,00E-012
NA12760	reg_DEFB	chr8	7302817	C	T	68	13,00%	9	-	SPAG11B	rs2740716		2,61E-005
NA12760	reg_DEFB	chr8	7303071	A	G	75	13,00%	10	-	SPAG11B			8,77E-006
NA12760	reg_DEFB	chr8	7303100	G	T	83	16,00%	13	-	SPAG11B			5,92E-008
NA12760	reg_DEFB	chr8	7303169	G	A	92	14,00%	13	-	SPAG11B			2,06E-007
NA12760	reg_DEFB	chr8	7303532	G	A	80	19,00%	15	-	SPAG11B			4,38E-010
NA12760	reg_DEFB	chr8	7303590	C	G	76	26,00%	20	-	SPAG11B	rs2740030		1,00E-012
NA12760	reg_DEFB	chr8	7303683	A	T	75	27,00%	20	-	SPAG11B	rs4532613		1,00E-012
NA12760	reg_DEFB	chr8	7303698	G	A	71	21,00%	15	-	SPAG11B		rs71235959	8,33E-011
NA12760	reg_DEFB	chr8	7303880	C	T	49	63,00%	31	-	SPAG11B		rs62641376	1,00E-012
NA12760	reg_DEFB	chr8	7303899	C	G	48	83,00%	40	-	SPAG11B		rs71526141	1,00E-012
NA12760	reg_DEFB	chr8	7303958	T	C	50	76,00%	38	-	SPAG11B		rs2740712	1,00E-012
NA12760	reg_DEFB	chr8	7303987	G	A	52	81,00%	42	-	SPAG11B		rs34315736	1,00E-012
NA12760	reg_DEFB	chr8	7303999	A	G	51	82,00%	42	-	SPAG11B		rs7464358	1,00E-012
NA12760	reg_DEFB	chr8	7304135	G	A	51	22,00%	11	-	SPAG11B			1,94E-008
NA12760	reg_DEFB	chr8	7304356	C	G	95	15,00%	14	-	SPAG11B		rs71242685	4,12E-008
NA12760	reg_DEFB	chr8	7304384	C	G	98	10,00%	10	-	SPAG11B	rs2738015		9,18E-005
NA12760	reg_DEFB	chr8	7304399	A	C	98	20,00%	20	-	SPAG11B		rs2853665	1,00E-012
NA12760	reg_DEFB	chr8	7304490	A	G	87	16,00%	14	-	SPAG11B		rs2737566	1,29E-008
NA12760	reg_DEFB	chr8	7304912	A	G	47	100,00%	47	-	SPAG11B	rs4840757		1,00E-012
NA12760	reg_DEFB	chr8	7305009	G	A	57	21,00%	12	-	SPAG11B			5,92E-009
NA12760	reg_DEFB	chr8	7305131	G	T	81	16,00%	13	-	SPAG11B			4,39E-008
NA12760	reg_DEFB	chr8	7305501	C	T	112	14,00%	16	-	SPAG11B			7,20E-009
NA12760	reg_DEFB	chr8	7306469	G	T	124	82,00%	102	-	SPAG11B	rs4840282		1,00E-012
NA12760	reg_DEFB	chr8	7306543	G	A	123	15,00%	18	-	SPAG11B			5,71E-010
NA12760	reg_DEFB	chr8	7306637	G	C	122	23,00%	28	-	SPAG11B			1,00E-012
NA12760	reg_DEFB	chr8	7307198	T	C	60	100,00%	60	-	SPAG11B		rs62636859	1,00E-012
NA12760	reg_DEFB	chr8	7307943	G	A	63	90,00%	57	-	SPAG11B	rs2251705		1,00E-012
NA12760	reg_DEFB	chr8	7308164	G	A	66	23,00%	15	-	SPAG11B	rs3817721		2,67E-011
NA12760	reg_DEFB	chr8	7308325	C	A	63	22,00%	14	-	SPAG11B			1,51E-010
NA12760	reg_DEFB	chr8	7308331	A	G	64	37,00%	24	-	SPAG11B	rs2272769		1,00E-012
NA12760	reg_DEFB	chr8	7308457	C	T	64	19,00%	12	-	SPAG11B		rs71242684	2,37E-008
NA12760	reg_DEFB	chr8	7308498	T	A	60	20,00%	12	-	SPAG11B	rs2272768		1,10E-008
NA12760	reg_DEFB	chr8	7308534	G	A	64	20,00%	13	-	SPAG11B			2,21E-009
NA12760	reg_DEFB	chr8	7308849	C	G	131	13,00%	17			rs4521786		1,09E-008
NA12760	reg_DEFB	chr8	7309001	C	T	130	17,00%	22			rs3762045		1,00E-012
NA12760	reg_DEFB	chr8	7309292	G	A	120	22,00%	26			rs17149290		1,00E-012
NA12760	reg_DEFB	chr8	7309563	T	C	94	13,00%	12			rs2740704		1,82E-006
NA12760	reg_DEFB	chr8	7309624	T	G	85	45,00%	38			rs2738013		1,00E-012
NA12760	reg_DEFB	chr8	7309975	G	A	100	99,00%	99					1,00E-012
NA12760	reg_DEFB	chr8	7310156	A	C	122	57,00%	70			rs2740702		1,00E-012
NA12760	reg_DEFB	chr8	7310241	G	T	126	69,00%	87			rs4840283		1,00E-012

NA12760	reg_DEFB	chr8	7310265	T	A	124	50,00%	62					rs2740701		1,00E-012
NA12760	reg_DEFB	chr8	7310309	C	T	130	14,00%	18					rs2740700		1,43E-009
NA12760	reg_DEFB	chr8	7310735	G	A	124	19,00%	24							1,00E-012
NA12760	reg_DEFB	chr8	7310943	T	G	141	19,00%	27							1,00E-012
NA12760	reg_DEFB	chr8	7311305	A	T	114	15,00%	17							1,26E-009
NA12760	reg_DEFB	chr8	7311383	G	A	101	14,00%	14							9,08E-008
NA12760	reg_DEFB	chr8	7311411	C	T	97	13,00%	13							3,87E-007
NA12760	reg_DEFB	chr8	7311441	C	T	81	100,00%	81							1,00E-012
NA12760	reg_DEFB	chr8	7311522	A	T	60	97,00%	58							1,00E-012
NA12760	reg_DEFB	chr8	7311642	G	A	64	11,00%	7						rs3132871	6,71E-004
NA12760	reg_DEFB	chr8	7311904	A	G	117	16,00%	19							3,87E-011
NA12760	reg_DEFB	chr8	7312136	G	T	124	18,00%	22							1,00E-012
NA12760	reg_DEFB	chr8	7312244	T	C	126	19,00%	24							1,00E-012
NA12760	reg_DEFB	chr8	7312534	T	C	128	13,00%	17						rs71242683	7,66E-009
NA12760	reg_DEFB	chr8	7312649	A	G	137	66,00%	90					rs2737570		1,00E-012
NA12760	reg_DEFB	chr8	7312682	G	T	153	38,00%	58					rs28374698		1,00E-012
NA12760	reg_DEFB	chr8	7312925	A	C	156	16,00%	25					rs2737572		1,00E-012
NA12760	reg_DEFB	chr8	7313500	C	G	111	35,00%	39					rs2737573		1,00E-012
NA12760	reg_DEFB	chr8	7313562	C	T	123	11,00%	14							1,07E-006
NA12760	reg_DEFB	chr8	7313761	A	G	108	20,00%	22					rs2737574		1,00E-012
NA12760	reg_DEFB	chr8	7314038	C	T	134	45,00%	60					rs2740026		1,00E-012
NA12760	reg_DEFB	chr8	7314172	A	G	129	17,00%	22					rs2740698		1,00E-012
NA12760	reg_DEFB	chr8	7314381	G	C	112	17,00%	19					rs2737575		1,72E-011
NA12760	reg_DEFB	chr8	7314399	C	A	112	47,00%	53					rs2740025		1,00E-012
NA12760	reg_DEFB	chr8	7314602	G	C	119	17,00%	20						rs71242681	6,12E-012
NA12760	reg_DEFB	chr8	7314802	A	G	170	18,00%	31							1,00E-012
NA12760	reg_DEFB	chr8	7315137	A	G	130	18,00%	23					rs2740697		1,00E-012
NA12760	reg_DEFB	chr8	7315210	C	T	136	16,00%	22					rs2740024		3,62E-012
NA12760	reg_DEFB	chr8	7315223	G	A	134	14,00%	19						rs71242680	3,34E-010
NA12760	reg_DEFB	chr8	7315394	C	T	125	16,00%	20	R	Q	-2	DEFB104A		rs71242679	1,60E-011
NA12760	reg_DEFB	chr8	7315477	A	C	103	47,00%	48			-	DEFB104A		rs2740023	1,00E-012
NA12760	reg_DEFB	chr8	7315525	G	A	97	19,00%	18			-	DEFB104A			1,18E-011
NA12760	reg_DEFB	chr8	7315533	G	A	95	95,00%	90			-	DEFB104A			1,00E-012
NA12760	reg_DEFB	chr8	7315562	G	T	104	17,00%	18			-	DEFB104A	rs2740696		4,09E-011
NA12760	reg_DEFB	chr8	7315606	C	T	116	11,00%	13			-	DEFB104A	rs2740022		3,04E-006
NA12760	reg_DEFB	chr8	7315640	A	G	112	46,00%	52			-	DEFB104A		rs71242678	1,00E-012
NA12760	reg_DEFB	chr8	7315688	A	G	118	19,00%	22			-	DEFB104A		rs71242677	1,00E-012
NA12760	reg_DEFB	chr8	7315783	T	C	121	15,00%	18			-	DEFB104A		rs71242676	4,34E-010
NA12760	reg_DEFB	chr8	7315804	C	A	120	20,00%	24			-	DEFB104A			1,00E-012
NA12760	reg_DEFB	chr8	7315842	G	C	120	12,00%	14			-	DEFB104A			7,88E-007
NA12760	reg_DEFB	chr8	7316076	T	C	84	15,00%	13			-	DEFB104A			6,86E-008
NA12760	reg_DEFB	chr8	7316113	C	T	85	86,00%	73			-	DEFB104A		rs62639764	1,00E-012
NA12760	reg_DEFB	chr8	7316116	A	C	83	49,00%	41			-	DEFB104A	rs2740021		1,00E-012
NA12760	reg_DEFB	chr8	7316428	C	T	97	96,00%	93			-	DEFB104A			1,00E-012
NA12760	reg_DEFB	chr8	7316918	C	A	67	15,00%	10			-	DEFB104A	rs2740020		3,10E-006
NA12760	reg_DEFB	chr8	7316983	C	T	63	51,00%	32			-	DEFB104A	rs2740019		1,00E-012
NA12760	reg_DEFB	chr8	7317035	C	T	71	100,00%	71			-	DEFB104A		rs62639766	1,00E-012
NA12760	reg_DEFB	chr8	7317106	G	T	83	23,00%	19			-	DEFB104A	rs2740018		1,00E-012
NA12760	reg_DEFB	chr8	7317157	G	A	84	48,00%	40			-	DEFB104A		rs62641371	1,00E-012
NA12760	reg_DEFB	chr8	7317224	G	A	89	12,00%	11			-	DEFB104A			6,67E-006
NA12760	reg_DEFB	chr8	7317239	G	A	95	33,00%	31			-	DEFB104A	rs2737576		1,00E-012
NA12760	reg_DEFB	chr8	7317261	C	A	97	97,00%	94			-	DEFB104A			1,00E-012
NA12760	reg_DEFB	chr8	7317363	C	A	98	22,00%	22			-	DEFB104A	rs2740011		1,00E-012

NA12760	reg_DEFB	chr8	7317411	T	C	81	22,00%	18			-	DEFB104A			1,00E-012
NA12760	reg_DEFB	chr8	7317588	G	A	59	19,00%	11			-	DEFB104A	rs6985641		9,63E-008
NA12760	reg_DEFB	chr8	7317813	C	G	17	94,00%	16			-	DEFB104A	rs7001088		1,00E-012
NA12760	reg_DEFB	chr8	7318122	A	T	5	100,00%	5			-	DEFB104A	rs7012750		6,44E-009
NA12760	reg_DEFB	chr8	7318293	T	C	9	33,00%	3			-	DEFB104A		rs73357863	9,21E-004
NA12760	reg_DEFB	chr8	7318591	C	T	31	29,00%	9			-	DEFB104A	rs2680505		2,29E-008
NA12760	reg_DEFB	chr8	7318756	A	T	35	54,00%	19			-	DEFB104A	rs2739991		1,00E-012
NA12760	reg_DEFB	chr8	7318925	T	C	52	62,00%	32			-	DEFB104A	rs2739988		1,00E-012
NA12760	reg_DEFB	chr8	7319055	T	C	81	62,00%	50			-	DEFB104A	rs2739981		1,00E-012
NA12760	reg_DEFB	chr8	7319063	T	C	80	64,00%	51			-	DEFB104A	rs2680506		1,00E-012
NA12760	reg_DEFB	chr8	7319275	A	T	97	44,00%	43			-	DEFB104A	rs2739976		1,00E-012
NA12760	reg_DEFB	chr8	7319333	T	C	98	18,00%	18			-	DEFB104A			1,42E-011
NA12760	reg_DEFB	chr8	7319456	T	C	92	54,00%	50			-	DEFB104A	rs2739969		1,00E-012
NA12760	reg_DEFB	chr8	7319556	A	G	84	52,00%	44			-	DEFB104A		rs2740692	1,00E-012
NA12760	reg_DEFB	chr8	7319599	C	T	84	44,00%	37			-	DEFB104A		rs2739962	1,00E-012
NA12760	reg_DEFB	chr8	7319638	C	T	81	41,00%	33			-	DEFB104A		rs2739960	1,00E-012
NA12760	reg_DEFB	chr8	7319747	A	G	83	86,00%	71			-	DEFB104A	rs4259430		1,00E-012
NA12760	reg_DEFB	chr8	7319771	T	C	85	87,00%	74			-	DEFB104A		rs17843872	1,00E-012
NA12760	reg_DEFB	chr8	7319973	T	C	83	18,00%	15	I	V	-1	DEFB104B	rs2680507		7,41E-010
NA12760	reg_DEFB	chr8	7320136	G	C	55	71,00%	39					rs28590291		1,00E-012
NA12760	reg_DEFB	chr8	7320212	C	G	53	68,00%	36							1,00E-012
NA12760	reg_DEFB	chr8	7320256	C	T	56	64,00%	36							1,00E-012
NA12760	reg_DEFB	chr8	7320389	C	T	64	70,00%	45							1,00E-012
NA12760	reg_DEFB	chr8	7320465	C	T	61	77,00%	47							1,00E-012
NA12760	reg_DEFB	chr8	7320516	A	G	56	75,00%	42						rs71308312	1,00E-012
NA12760	reg_DEFB	chr8	7320524	C	G	57	77,00%	44						rs71308312	1,00E-012
NA12760	reg_DEFB	chr8	7320543	A	G	57	72,00%	41							1,00E-012
NA12760	reg_DEFB	chr8	7320547	A	G	59	75,00%	44							1,00E-012
NA12760	reg_DEFB	chr8	7320699	G	A	58	88,00%	51						rs71272074	1,00E-012
NA12760	reg_DEFB	chr8	7320772	A	C	50	72,00%	36					rs2680508		1,00E-012
NA12760	reg_DEFB	chr8	7320916	A	G	66	95,00%	63						rs61413127	1,00E-012
NA12760	reg_DEFB	chr8	7320949	G	A	76	68,00%	52					rs2740690		1,00E-012
NA12760	reg_DEFB	chr8	7320963	C	T	79	19,00%	15							3,64E-010
NA12760	reg_DEFB	chr8	7320971	C	G	81	21,00%	17					rs2680509		1,41E-011
NA12760	reg_DEFB	chr8	7321565	A	G	47	13,00%	6					rs2739944		7,08E-004
NA12760	reg_DEFB	chr8	7321575	T	A	46	57,00%	26					rs2739943		1,00E-012
NA12760	reg_DEFB	chr8	7321621	C	T	46	57,00%	26						rs2680510	1,00E-012
NA12760	reg_DEFB	chr8	7321847	G	T	76	13,00%	10							9,89E-006
NA12760	reg_DEFB	chr8	7322171	G	A	72	57,00%	41						rs2680512	1,00E-012
NA12760	reg_DEFB	chr8	7322389	A	G	54	74,00%	40						rs2680515	1,00E-012
NA12760	reg_DEFB	chr8	7322414	C	T	49	67,00%	33						rs2680516	1,00E-012
NA12760	reg_DEFB	chr8	7322468	T	C	39	31,00%	12							5,13E-011
NA12760	reg_DEFB	chr8	7322839	C	A	31	65,00%	20					rs28689192		1,00E-012
NA12760	reg_DEFB	chr8	7322882	A	C	32	97,00%	31						rs6472800	1,00E-012
NA12760	reg_DEFB	chr8	7322889	C	T	32	100,00%	32						rs4602913	1,00E-012
NA12760	reg_DEFB	chr8	7322954	C	G	49	100,00%	49					rs28695206		1,00E-012
NA12760	reg_DEFB	chr8	7323020	A	T	68	74,00%	50						rs2739908	1,00E-012
NA12760	reg_DEFB	chr8	7323118	A	G	99	89,00%	88					rs2737579		1,00E-012
NA12760	reg_DEFB	chr8	7323294	A	G	114	31,00%	35					rs2739898		1,00E-012
NA12760	reg_DEFB	chr8	7323491	T	G	98	15,00%	15					rs2739894		8,04E-009
NA12760	reg_DEFB	chr8	7323584	G	A	107	20,00%	21						rs71247893	1,00E-012
NA12760	reg_DEFB	chr8	7323601	G	A	102	13,00%	13						rs2680519	6,97E-007
NA12760	reg_DEFB	chr8	7323645	G	C	100	34,00%	34						rs71247894	1,00E-012

NA12760	reg_DEFB	chr8	7323679	C	T	105	93,00%	98					rs28557092	rs41405850		1,00E-012
NA12760	reg_DEFB	chr8	7323747	T	G	107	14,00%	15					rs2739889			2,73E-008
NA12760	reg_DEFB	chr8	7323827	T	C	102	16,00%	16							rs2737581	1,79E-009
NA12760	reg_DEFB	chr8	7323880	G	A	100	15,00%	15							rs2680521	1,07E-008
NA12760	reg_DEFB	chr8	7323982	C	T	105	16,00%	17								3,47E-010
NA12760	reg_DEFB	chr8	7323985	G	A	107	15,00%	16					rs2680522			3,66E-009
NA12760	reg_DEFB	chr8	7324031	C	A	116	32,00%	37					rs4840760			1,00E-012
NA12760	reg_DEFB	chr8	7324145	G	A	101	15,00%	15							rs2680523	1,23E-008
NA12760	reg_DEFB	chr8	7324233	A	T	108	44,00%	48					rs4467955			1,00E-012
NA12760	reg_DEFB	chr8	7324614	A	G	80	16,00%	13					rs2680525			3,76E-008
NA12760	reg_DEFB	chr8	7324635	T	A	81	11,00%	9					rs2740689			1,06E-004
NA12760	reg_DEFB	chr8	7324672	C	T	77	10,00%	8					rs2680526			4,02E-004
NA12760	reg_DEFB	chr8	7324797	T	G	58	14,00%	8							rs56122448	5,38E-005
NA12760	reg_DEFB	chr8	7324845	T	G	64	14,00%	9							rs55754272	1,58E-005
NA12760	reg_DEFB	chr8	7324880	C	T	65	11,00%	7					rs2954313			7,38E-004
NA12760	reg_DEFB	chr8	7324941	G	C	69	12,00%	8					rs2977416			1,88E-004
NA12760	reg_DEFB	chr8	7324973	G	A	67	10,00%	7							rs2740158	8,86E-004
NA12760	reg_DEFB	chr8	7325028	A	G	81	75,00%	61					rs6651517			1,00E-012
NA12760	reg_DEFB	chr8	7325133	A	G	109	20,00%	22					rs2680528			1,00E-012
NA12760	reg_DEFB	chr8	7325219	A	G	114	18,00%	21							rs2740154	1,00E-012
NA12760	reg_DEFB	chr8	7325548	T	C	81	14,00%	11					rs2737584			2,62E-006
NA12760	reg_DEFB	chr8	7325767	T	C	106	38,00%	40							rs62639773	1,00E-012
NA12760	reg_DEFB	chr8	7325835	A	T	103	15,00%	15								1,61E-008
NA12760	reg_DEFB	chr8	7325839	C	T	105	13,00%	14								1,49E-007
NA12760	reg_DEFB	chr8	7325875	A	G	102	31,00%	32					rs2740137			1,00E-012
NA12760	reg_DEFB	chr8	7325879	A	C	100	16,00%	16					rs2680531			1,33E-009
NA12760	reg_DEFB	chr8	7326033	G	C	93	12,00%	11					rs2737585			1,02E-005
NA12760	reg_DEFB	chr8	7326060	C	T	89	55,00%	49					rs2740125			1,00E-012
NA12760	reg_DEFB	chr8	7326067	C	G	86	13,00%	11					rs2740124			4,76E-006
NA12760	reg_DEFB	chr8	7326472	G	A	115	19,00%	22					rs2680532			1,00E-012
NA12760	reg_DEFB	chr8	7326494	A	G	119	20,00%	24					rs2740102			1,00E-012
NA12760	reg_DEFB	chr8	7326592	T	C	104	22,00%	23							rs2737586	1,00E-012
NA12760	reg_DEFB	chr8	7326628	C	T	92	99,00%	91							rs62639774	1,00E-012
NA12760	reg_DEFB	chr8	7326698	C	T	60	18,00%	11					rs2737587			1,15E-007
NA12760	reg_DEFB	chr8	7326715	A	G	46	24,00%	11					rs2979555			6,04E-009
NA12760	reg_DEFB	chr8	7327125	A	G	37	22,00%	8					rs2980523			1,66E-006
NA12760	reg_DEFB	chr8	7327254	C	T	61	26,00%	16					rs2740084			1,00E-012
NA12760	reg_DEFB	chr8	7327310	T	A	68	22,00%	15					rs2680545			4,26E-011
NA12760	reg_DEFB	chr8	7327328	G	A	72	100,00%	72					rs2293956			1,00E-012
NA12760	reg_DEFB	chr8	7327333	G	A	71	99,00%	70								1,00E-012
NA12760	reg_DEFB	chr8	7327614	A	G	108	12,00%	13	N	N	-2	DEFB106A	rs2293957			1,35E-006
NA12760	reg_DEFB	chr8	7327688	C	T	103	12,00%	12			-	DEFB106B	rs2740082			4,81E-006
NA12760	reg_DEFB	chr8	7327723	T	A	103	16,00%	16			-	DEFB106B				2,07E-009
NA12760	reg_DEFB	chr8	7327733	A	G	107	34,00%	36			-	DEFB106B	rs2244098			1,00E-012
NA12760	reg_DEFB	chr8	7327748	G	A	107	64,00%	68			-	DEFB106B	rs2244096			1,00E-012
NA12760	reg_DEFB	chr8	7327863	C	T	92	15,00%	14			-	DEFB106B				2,71E-008
NA12760	reg_DEFB	chr8	7328121	A	G	76	36,00%	27			-	DEFB106B	rs2243995			1,00E-012
NA12760	reg_DEFB	chr8	7328248	C	T	95	21,00%	20			-	DEFB106B	rs2738002			1,00E-012
NA12760	reg_DEFB	chr8	7328290	T	C	96	61,00%	59			-	DEFB106B	rs2740081			1,00E-012
NA12760	reg_DEFB	chr8	7328305	C	A	95	34,00%	32			-	DEFB106B			rs62641365	1,00E-012
NA12760	reg_DEFB	chr8	7328421	T	C	112	13,00%	15			-	DEFB106B	rs6984237			5,10E-008
NA12760	reg_DEFB	chr8	7328517	A	G	107	15,00%	16			-	DEFB106B	rs2740078			3,66E-009
NA12760	reg_DEFB	chr8	7328700	C	T	46	100,00%	46			-	DEFB106B	rs28548808			1,00E-012

NA12760	reg_DEFB	chr8	7328712	T	C	46	37,00%	17			-	DEFB106B	rs2680547	rs28377333		1,00E-012
NA12760	reg_DEFB	chr8	7328762	A	C	34	32,00%	11			-	DEFB106B	rs2680548			1,76E-010
NA12760	reg_DEFB	chr8	7328769	T	G	31	16,00%	5			-	DEFB106B			rs72626625	6,64E-004
NA12760	reg_DEFB	chr8	7329799	A	C	7	43,00%	3			-	DEFB106B	rs2737589			3,97E-004
NA12760	reg_DEFB	chr8	7329944	A	T	13	85,00%	11			-	DEFB106B	rs2740058			1,00E-012
NA12760	reg_DEFB	chr8	7330145	A	T	15	100,00%	15			-	DEFB106B				1,00E-012
NA12760	reg_DEFB	chr8	7330373	G	C	33	21,00%	7			-	DEFB106B	rs2740039			8,59E-006
NA12760	reg_DEFB	chr8	7330474	G	A	51	25,00%	13			-	DEFB106B	rs2740038			1,16E-010
NA12760	reg_DEFB	chr8	7330551	T	C	58	45,00%	26			-	DEFB106B	rs6605634			1,00E-012
NA12760	reg_DEFB	chr8	7330574	C	G	71	25,00%	18			-	DEFB106B			rs62639775	1,00E-012
NA12760	reg_DEFB	chr8	7330881	T	A	102	28,00%	29			-	DEFB106B			rs62639776	1,00E-012
NA12760	reg_DEFB	chr8	7331105	C	T	64	11,00%	7			-	DEFB106B	rs3748149			6,71E-004
NA12760	reg_DEFB	chr8	7331216	C	G	61	69,00%	42			-	DEFB106B	rs2738000			1,00E-012
NA12760	reg_DEFB	chr8	7331608	C	G	83	100,00%	83								1,00E-012
NA12760	reg_DEFB	chr8	7331822	C	T	91	21,00%	19					rs6999662			1,00E-012
NA12760	reg_DEFB	chr8	7331835	G	A	91	18,00%	16					rs2740027			3,18E-010
NA12760	reg_DEFB	chr8	7332215	T	A	99	17,00%	17					rs6982591			1,33E-010
NA12760	reg_DEFB	chr8	7332633	C	T	104	17,00%	18					rs2737997			4,09E-011
NA12760	reg_DEFB	chr8	7332663	T	A	111	33,00%	37	I	N	1	DEFB105B			rs62639778	1,00E-012
NA12760	reg_DEFB	chr8	7332749	T	C	119	20,00%	24			+	DEFB105A	rs2737996			1,00E-012
NA12760	reg_DEFB	chr8	7333168	G	C	115	100,00%	115			+	DEFB105A				1,00E-012
NA12760	reg_DEFB	chr8	7333507	T	C	125	36,00%	45			+	DEFB105A	rs2737994			1,00E-012
NA12760	reg_DEFB	chr8	7333953	C	T	144	35,00%	50			+	DEFB105A	rs6422765	rs2737593		1,00E-012
NA12760	reg_DEFB	chr8	7334564	C	G	105	36,00%	38							rs62639779	1,00E-012
NA12760	reg_DEFB	chr8	7334688	T	C	62	19,00%	12							rs2737992	1,63E-008
NA12760	reg_DEFB	chr8	7334804	C	T	56	20,00%	11					rs2737594			5,46E-008
NA12760	reg_DEFB	chr8	7334844	G	C	60	35,00%	21							rs62639780	1,00E-012
NA12760	reg_DEFB	chr8	7334996	A	G	112	83,00%	93					rs2737595			1,00E-012
NA12760	reg_DEFB	chr8	7335115	C	T	138	18,00%	25					rs2737596			1,00E-012
NA12760	reg_DEFB	chr8	7335121	T	C	137	100,00%	137								1,00E-012
NA12760	reg_DEFB	chr8	7335165	C	G	145	17,00%	25					rs2737597			1,00E-012
NA12760	reg_DEFB	chr8	7335196	A	G	147	16,00%	24					rs2737598			1,00E-012
NA12760	reg_DEFB	chr8	7335401	A	G	134	99,00%	133					rs2977418			1,00E-012
NA12760	reg_DEFB	chr8	7335410	T	C	136	18,00%	24					rs7462905			1,00E-012
NA12760	reg_DEFB	chr8	7335573	T	C	123	33,00%	41					rs4481626			1,00E-012
NA12760	reg_DEFB	chr8	7336339	A	T	108	13,00%	14					rs2737991			2,13E-007
NA12760	reg_DEFB	chr8	7336570	C	T	61	18,00%	11							rs62639783	1,38E-007
NA12760	reg_DEFB	chr8	7336640	T	G	44	30,00%	13					rs2737599			3,42E-011
NA12760	reg_DEFB	chr8	7336834	A	G	19	21,00%	4					rs2737600			8,22E-004
NA12760	reg_DEFB	chr8	7336931	A	C	26	58,00%	15					rs2737601			1,00E-012
NA12760	reg_DEFB	chr8	7337241	C	T	74	19,00%	14					rs2737602			1,45E-009
NA12760	reg_DEFB	chr8	7337388	G	A	95	18,00%	17								6,70E-011
NA12760	reg_DEFB	chr8	7337863	A	C	51	86,00%	44								1,00E-012
NA12760	reg_DEFB	chr8	7338251	A	C	86	26,00%	22					rs2737989			1,00E-012
NA12760	reg_DEFB	chr8	7338279	T	C	88	99,00%	87					rs2946448			1,00E-012
NA12760	reg_DEFB	chr8	7338381	C	T	94	100,00%	94					rs2977421			1,00E-012
NA12760	reg_DEFB	chr8	7338534	G	T	72	68,00%	49					rs2737604			1,00E-012
NA12760	reg_DEFB	chr8	7338703	C	T	72	24,00%	17					rs2737605			1,00E-012
NA12760	reg_DEFB	chr8	7338734	C	T	77	61,00%	47					rs2680559			1,00E-012
NA12760	reg_DEFB	chr8	7338884	A	G	98	89,00%	87							rs62639786	1,00E-012
NA12760	reg_DEFB	chr8	7338975	G	A	105	17,00%	18								4,84E-011
NA12760	reg_DEFB	chr8	7339053	T	G	108	18,00%	19							rs71236463	8,71E-012
NA12760	reg_DEFB	chr8	7339159	G	T	117	12,00%	14					rs2680560			5,77E-007

NA12760	reg_DEFB	chr8	7339447	C	T	123	98,00%	121						rs62639787	1,00E-012
NA12760	reg_DEFB	chr8	7339485	C	T	124	60,00%	74					rs2680561		1,00E-012
NA12760	reg_DEFB	chr8	7339502	A	G	130	15,00%	20							3,44E-011
NA12760	reg_DEFB	chr8	7339538	C	G	137	100,00%	137					rs2737606		1,00E-012
NA12760	reg_DEFB	chr8	7339593	A	G	142	20,00%	28						rs71236462	1,00E-012
NA12760	reg_DEFB	chr8	7339872	A	G	137	69,00%	95					rs2737608		1,00E-012
NA12760	reg_DEFB	chr8	7339927	A	C	130	70,00%	91					rs2737609		1,00E-012
NA12760	reg_DEFB	chr8	7340022	T	C	108	33,00%	36					rs2737610		1,00E-012
NA12760	reg_DEFB	chr8	7340195	C	A	68	65,00%	44					rs2737988		1,00E-012
NA12760	reg_DEFB	chr8	7340261	C	G	54	19,00%	10						rs62639790	3,93E-007
NA12760	reg_DEFB	chr8	7340654	G	A	68	15,00%	10					rs2737611		3,56E-006
NA12760	reg_DEFB	chr8	7340708	T	C	82	16,00%	13						rs2680425	5,10E-008
NA12760	reg_DEFB	chr8	7340710	G	A	82	100,00%	82							1,00E-012
NA12760	reg_DEFB	chr8	7340890	G	T	95	26,00%	25	V	F	1	DEFB107A	rs2246582		1,00E-012
NA12760	reg_DEFB	chr8	7341300	T	C	40	97,00%	39			+	DEFB107A	rs12682203		1,00E-012
NA12760	reg_DEFB	chr8	7341374	C	G	39	41,00%	16			+	DEFB107A		rs62639791	1,00E-012
NA12760	reg_DEFB	chr8	7341377	A	G	39	38,00%	15			+	DEFB107A		rs73366541	1,00E-012
NA12760	reg_DEFB	chr8	7341389	G	T	36	17,00%	6			+	DEFB107A	rs12675434		1,59E-004
NA12760	reg_DEFB	chr8	7341391	C	G	36	25,00%	9			+	DEFB107A	rs2737612		9,66E-008
NA12760	reg_DEFB	chr8	7341618	G	T	106	20,00%	21			+	DEFB107A		rs62641362	1,00E-012
NA12760	reg_DEFB	chr8	7341639	C	T	117	22,00%	26			+	DEFB107A		rs62641361	1,00E-012
NA12760	reg_DEFB	chr8	7341834	G	C	196	15,00%	29			+	DEFB107A		rs71251575	1,00E-012
NA12760	reg_DEFB	chr8	7342189	A	G	194	17,00%	33			+	DEFB107A	rs2737614		1,00E-012
NA12760	reg_DEFB	chr8	7342213	G	C	197	17,00%	33			+	DEFB107A		rs73199782	1,00E-012
NA12760	reg_DEFB	chr8	7342346	G	A	182	32,00%	58			+	DEFB107A		rs66911494	1,00E-012
NA12760	reg_DEFB	chr8	7343575	C	T	3	100,00%	3			+	DEFB107A		rs4538904	1,22E-005
NA12760	reg_DEFB	chr8	7343596	C	T	3	100,00%	3			+	DEFB107A		rs9774358	1,22E-005
NA12760	reg_DEFB	chr8	7343651	C	T	3	100,00%	3			+	DEFB107A		rs35904342	1,22E-005
NA12760	reg_DEFB	chr8	7343692	C	T	3	100,00%	3			+	DEFB107A		rs62515824	1,22E-005
NA12760	reg_DEFB	chr8	7343714	C	T	3	100,00%	3			+	DEFB107A	rs9720857		1,22E-005
NA12760	reg_DEFB	chr8	7346227	C	T	3	100,00%	3			+	DEFB107A			1,22E-005
NA12760	reg_DEFB	chr8	7347990	G	A	7	71,00%	5			+	DEFB107A			1,30E-007
NA12760	reg_DEFB	chr8	7348074	T	C	7	86,00%	6			+	DEFB107A			1,02E-009
NA12760	reg_DEFB	chr8	7348303	A	G	12	100,00%	12			+	DEFB107A			1,00E-012
NA12760	reg_DEFB	chr8	7348338	C	A	11	100,00%	11			+	DEFB107A			1,00E-012
NA12760	reg_DEFB	chr8	7348349	T	C	10	50,00%	5			+	DEFB107A			1,47E-006
NA12760	reg_DEFB	chr8	7348357	A	G	11	73,00%	8			+	DEFB107A			1,56E-011
NA12760	reg_DEFB	chr8	7348411	A	C	9	44,00%	4			+	DEFB107A			3,21E-005
NA12760	reg_DEFB	chr8	7348467	C	A	6	67,00%	4			+	DEFB107A			4,04E-006
NA12760	reg_DEFB	chr8	7352319	C	G	40	32,00%	13			+	DEFB107A		rs72494253	8,21E-012
NA12760	reg_DEFB	chr8	7352334	T	C	51	33,00%	17			+	DEFB107A		rs73199783	1,00E-012
NA12760	reg_DEFB	chr8	7352362	G	T	59	54,00%	32			+	DEFB107A		rs2737986	1,00E-012
NA12760	reg_DEFB	chr8	7352774	T	A	112	14,00%	16			+	DEFB107A	rs2737616		7,20E-009
NA12760	reg_DEFB	chr8	7352969	T	C	91	98,00%	89			+	DEFB107A	rs12155828		1,00E-012
NA12760	reg_DEFB	chr8	7353013	A	G	108	45,00%	49			+	DEFB107A		rs2737984	1,00E-012
NA12760	reg_DEFB	chr8	7353015	A	G	109	92,00%	100			+	DEFB107A		rs72329447	1,00E-012
NA12760	reg_DEFB	chr8	7353162	C	T	127	26,00%	33			+	DEFB107A		rs2737474	1,00E-012
NA12760	reg_DEFB	chr8	7353297	A	C	149	13,00%	19			+	DEFB107A		rs71511272	2,07E-009
NA12760	reg_DEFB	chr8	7353385	A	G	154	42,00%	65			+	DEFB107A	rs2737983		1,00E-012
NA12760	reg_DEFB	chr8	7353437	A	G	145	19,00%	28			+	DEFB107A	rs2737475		1,00E-012
NA12760	reg_DEFB	chr8	7353557	C	T	135	33,00%	45			+	DEFB107A	rs11775409		1,00E-012
NA12760	reg_DEFB	chr8	7353946	C	T	108	39,00%	42			+	DEFB107A		rs62639796	1,00E-012
NA12760	reg_DEFB	chr8	7353996	T	C	110	46,00%	51			+	DEFB107A	rs2737476		1,00E-012

NA12760	reg_DEFB	chr8	7354227	T	C	140	77,00%	108	+	DEFB107A	rs2737477		1,00E-012
NA12760	reg_DEFB	chr8	7354364	A	G	153	33,00%	50			rs4143089		1,00E-012
NA12760	reg_DEFB	chr8	7354376	T	C	154	32,00%	49			rs4143090		1,00E-012
NA12760	reg_DEFB	chr8	7354417	T	A	152	18,00%	27			rs2680430		1,00E-012
NA12760	reg_DEFB	chr8	7354477	G	A	150	27,00%	41			rs4143091		1,00E-012
NA12760	reg_DEFB	chr8	7354592	A	T	151	15,00%	23				rs2737478	4,21E-012
NA12760	reg_DEFB	chr8	7354673	C	G	132	80,00%	106				rs62639797	1,00E-012
NA12760	reg_DEFB	chr8	7354872	T	G	121	13,00%	16			rs2737479		2,23E-008
NA12760	reg_DEFB	chr8	7355739	G	A	75	100,00%	75				rs71511273	1,00E-012
NA12760	reg_DEFB	chr8	7356482	G	C	88	15,00%	13				rs71511274	1,21E-007
NA12760	reg_DEFB	chr8	7357088	T	C	147	46,00%	68			rs2737481		1,00E-012
NA12760	reg_DEFB	chr8	7357411	A	C	147	95,00%	140			rs4840763		1,00E-012
NA12760	reg_DEFB	chr8	7357553	G	A	173	51,00%	88			rs2737979		1,00E-012
NA12760	reg_DEFB	chr8	7357665	T	G	175	16,00%	28			rs2737482		1,00E-012
NA12760	reg_DEFB	chr8	7357798	C	T	175	34,00%	60				rs2737483	1,00E-012
NA12760	reg_DEFB	chr8	7357880	T	C	164	96,00%	157				rs71509114	1,00E-012
NA12760	reg_DEFB	chr8	7357983	G	A	173	32,00%	55				rs2737978	1,00E-012
NA12760	reg_DEFB	chr8	7358487	A	G	164	32,00%	52			rs2737977		1,00E-012
NA12760	reg_DEFB	chr8	7358618	C	T	181	98,00%	177					1,00E-012
NA12760	reg_DEFB	chr8	7358904	G	A	148	100,00%	148				rs62639798	1,00E-012
NA12760	reg_DEFB	chr8	7358942	C	T	131	38,00%	50			rs2680433		1,00E-012
NA12760	reg_DEFB	chr8	7359000	G	A	111	100,00%	111			rs11984588		1,00E-012
NA12760	reg_DEFB	chr8	7359515	C	A	66	100,00%	66				rs71239493	1,00E-012
NA12760	reg_DEFB	chr8	7359657	T	G	70	100,00%	70				rs62639800	1,00E-012
NA12760	reg_DEFB	chr8	7359721	C	T	61	16,00%	10				rs62640758	1,28E-006
NA12760	reg_DEFB	chr8	7360225	G	A	51	45,00%	23				rs2680434	1,00E-012
NA12760	reg_DEFB	chr8	7360474	C	T	43	14,00%	6			rs2737487		4,35E-004
NA12760	reg_DEFB	chr8	7360635	T	G	82	16,00%	13				rs71259275	5,10E-008
NA12760	reg_DEFB	chr8	7361108	A	G	72	31,00%	22				rs62639801	1,00E-012
NA12760	reg_DEFB	chr8	7361125	T	C	76	32,00%	24				rs62639802	1,00E-012
NA12760	reg_DEFB	chr8	7361697	C	T	80	17,00%	14				rs67213127	4,20E-009
NA12760	reg_DEFB	chr8	7361701	G	T	82	49,00%	40				rs62639803	1,00E-012
NA12760	reg_DEFB	chr8	7361824	G	T	60	18,00%	11				rs71268643	1,15E-007
NA12760	reg_DEFB	chr8	7361829	G	T	60	17,00%	10				rs71268643	1,09E-006
NA12760	reg_DEFB	chr8	7361937	C	T	61	49,00%	30			rs4840765		1,00E-012
NA12760	reg_DEFB	chr8	7361941	A	G	61	15,00%	9					1,06E-005
NA12760	reg_DEFB	chr8	7362037	C	G	50	14,00%	7				rs66478539	1,43E-004
NA12760	reg_DEFB	chr8	7362076	A	G	47	30,00%	14				rs71230560	4,96E-012
NA12760	reg_DEFB	chr8	7362278	G	A	31	16,00%	5				rs71230561	6,64E-004
NA12760	reg_DEFB	chr8	7362617	A	G	30	80,00%	24				rs71213914	1,00E-012
NA12760	reg_DEFB	chr8	7362978	C	T	36	36,00%	13				rs62639805	1,00E-012
NA12760	reg_DEFB	chr8	7362993	C	T	37	30,00%	11			rs2680436		4,70E-010
NA12760	reg_DEFB	chr8	7363131	C	T	25	24,00%	6				rs66602902	1,80E-005
NA12760	reg_DEFB	chr8	7363844	A	T	77	38,00%	29				rs2737488	1,00E-012
NA12760	reg_DEFB	chr8	7363941	A	G	87	37,00%	32				rs2680438	1,00E-012
NA12760	reg_DEFB	chr8	7364133	G	A	111	14,00%	16			rs2680439		6,31E-009
NA12760	reg_DEFB	chr8	7364280	A	G	107	14,00%	15				rs71228231	2,73E-008
NA12760	reg_DEFB	chr8	7364478	C	G	154	71,00%	109			rs2680440		1,00E-012
NA12760	reg_DEFB	chr8	7364562	G	T	149	20,00%	30				rs4590459	1,00E-012
NA12760	reg_DEFB	chr8	7364725	T	A	139	17,00%	24			rs4556104		1,00E-012
NA12760	reg_DEFB	chr8	7364726	T	C	140	21,00%	29					1,00E-012
NA12760	reg_DEFB	chr8	7365153	T	C	139	17,00%	24				rs71228230	1,00E-012
NA12760	reg_DEFB	chr8	7365196	A	G	138	18,00%	25				rs67575026	1,00E-012

NA12760	reg_DEFB	chr8	7365483	G	C	119	10,00%	12			2,13E-005
NA12760	reg_DEFB	chr8	7365664	C	A	125	25,00%	31		rs71511277	1,00E-012
NA12760	reg_DEFB	chr8	7365938	G	A	129	15,00%	19		rs71511278	1,78E-010
NA12760	reg_DEFB	chr8	7366130	G	T	137	15,00%	21		rs71511279	1,19E-011
NA12760	reg_DEFB	chr8	7366324	G	A	115	17,00%	20	rs4584163		8,76E-012
NA12760	reg_DEFB	chr8	7366488	A	G	74	12,00%	9	rs4263787		5,16E-005
NA12760	reg_DEFB	chr8	7366494	A	C	73	49,00%	36	rs4270988		1,00E-012
NA12760	reg_DEFB	chr8	7366516	C	G	71	13,00%	9	rs4446761		3,70E-005
NA12760	reg_DEFB	chr8	7366531	T	G	66	12,00%	8	rs4335141		1,37E-004
NA12760	reg_DEFB	chr8	7367473	C	G	103	20,00%	21	rs4440657		1,00E-012
NA12760	reg_DEFB	chr8	7367494	C	G	101	29,00%	29			1,00E-012
NA12760	reg_DEFB	chr8	7367539	G	C	95	29,00%	28			1,00E-012
NA12760	reg_DEFB	chr8	7367542	G	T	94	26,00%	24			1,00E-012
NA12760	reg_DEFB	chr8	7367818	T	C	21	90,00%	19		rs7842766	1,00E-012
NA12760	reg_DEFB	chr8	7367967	G	A	18	22,00%	4			6,61E-004
NA12760	reg_DEFB	chr8	7368321	C	T	111	12,00%	13	rs2737964		1,85E-006
NA12760	reg_DEFB	chr8	7368407	T	G	130	19,00%	25		rs4599836	1,00E-012
NA12760	reg_DEFB	chr8	7368542	T	A	149	63,00%	94		rs71511292	1,00E-012
NA12760	reg_DEFB	chr8	7368771	A	T	102	30,00%	31	rs2737491		1,00E-012
NA12760	reg_DEFB	chr8	7369708	C	T	148	71,00%	105	rs725058		1,00E-012
NA12760	reg_DEFB	chr8	7369907	A	G	149	16,00%	24	rs2680485		1,00E-012
NA12760	reg_DEFB	chr8	7369959	G	A	138	28,00%	39	rs725057		1,00E-012
NA12760	reg_DEFB	chr8	7370028	T	G	122	32,00%	39	rs2680484		1,00E-012
NA12760	reg_DEFB	chr8	7370074	C	A	113	29,00%	33		rs62639806	1,00E-012
NA12760	reg_DEFB	chr8	7370374	G	C	89	28,00%	25	rs2737961		1,00E-012
NA12760	reg_DEFB	chr8	7370610	A	G	131	64,00%	84		rs2680482	1,00E-012
NA12760	reg_DEFB	chr8	7371168	C	T	83	19,00%	16	rs1002943		7,48E-011
NA12760	reg_DEFB	chr8	7371271	C	A	84	31,00%	26	rs2737492		1,00E-012
NA12760	reg_DEFB	chr8	7371475	C	T	78	71,00%	55	rs4840766		1,00E-012
NA12760	reg_DEFB	chr8	7371487	C	A	73	30,00%	22	rs2737493		1,00E-012
NA12760	reg_DEFB	chr8	7372029	C	T	67	13,00%	9		rs71264712	2,31E-005
NA12760	reg_DEFB	chr8	7372124	C	T	79	28,00%	22	rs2977691		1,00E-012
NA12760	reg_DEFB	chr8	7372488	C	G	155	15,00%	23		rs71264713	7,44E-012
NA12760	reg_DEFB	chr8	7373102	G	A	73	22,00%	16	rs2977399		1,07E-011
NA12760	reg_DEFB	chr8	7373254	C	T	47	55,00%	26	rs1807385		1,00E-012
NA12760	reg_DEFB	chr8	7373360	A	T	29	21,00%	6		rs1985804	4,46E-005
NA12760	reg_DEFB	chr8	7374510	C	T	79	18,00%	14		rs71249119	3,54E-009
NA12760	reg_DEFB	chr8	7374904	G	A	68	22,00%	15	rs2977690		4,26E-011
NA12760	reg_DEFB	chr8	7375439	G	A	90	59,00%	53		rs71249120	1,00E-012
NA12760	reg_DEFB	chr8	7375518	G	A	70	43,00%	30	rs4840769		1,00E-012
NA12760	reg_DEFB	chr8	7375596	C	A	81	38,00%	31	rs11786478		1,00E-012
NA12760	reg_DEFB	chr8	7375735	G	T	82	65,00%	53		rs71249122	1,00E-012
NA12760	reg_DEFB	chr8	7375812	G	A	82	50,00%	41		rs71264915	1,00E-012
NA12760	reg_DEFB	chr8	7375818	C	T	81	36,00%	29		rs71264915	1,00E-012
NA12760	reg_DEFB	chr8	7375977	G	T	79	59,00%	47	rs2737951		1,00E-012
NA12760	reg_DEFB	chr8	7375987	A	G	78	58,00%	45	rs2737950		1,00E-012
NA12760	reg_DEFB	chr8	7376096	T	A	71	48,00%	34	rs2680503		1,00E-012
NA12760	reg_DEFB	chr8	7376598	C	T	119	27,00%	32	rs2737949		1,00E-012
NA12760	reg_DEFB	chr8	7377165	A	G	56	70,00%	39		rs71249124	1,00E-012
NA12760	reg_DEFB	chr8	7377439	G	A	101	35,00%	35	rs2680501		1,00E-012
NA12760	reg_DEFB	chr8	7377464	C	A	101	99,00%	100			1,00E-012
NA12760	reg_DEFB	chr8	7377536	G	C	95	33,00%	31	rs2737947		1,00E-012
NA12760	reg_DEFB	chr8	7378654	T	C	81	96,00%	78		rs71249125	1,00E-012

NA12760	reg_DEFB	chr8	7379026	G	A	66	98,00%	65			rs67847292	1,00E-012
NA12760	reg_DEFB	chr8	7379048	T	C	61	98,00%	60			rs71249126	1,00E-012
NA12760	reg_DEFB	chr8	7380428	G	A	79	99,00%	78				1,00E-012
NA12760	reg_DEFB	chr8	7381443	A	G	128	44,00%	56	rs2737940			1,00E-012
NA12760	reg_DEFB	chr8	7381476	G	A	124	60,00%	74	rs2737939			1,00E-012
NA12760	reg_DEFB	chr8	7381694	T	C	84	25,00%	21	rs2737498			1,00E-012
NA12760	reg_DEFB	chr8	7381963	G	A	52	19,00%	10	rs4311672			2,71E-007
NA12760	reg_DEFB	chr8	7382091	A	C	55	49,00%	27	rs2737936			1,00E-012
NA12760	reg_DEFB	chr8	7382305	C	G	82	65,00%	53	rs2737499			1,00E-012
NA12760	reg_DEFB	chr8	7382355	G	A	89	16,00%	14	rs2737935			1,75E-008
NA12760	reg_DEFB	chr8	7382367	G	C	91	15,00%	14				2,35E-008
NA12760	reg_DEFB	chr8	7382406	C	A	102	34,00%	35	rs4461922			1,00E-012
NA12760	reg_DEFB	chr8	7382462	C	T	105	33,00%	35	rs4633079			1,00E-012
NA12760	reg_DEFB	chr8	7382473	T	G	106	32,00%	34			rs62639815	1,00E-012
NA12760	reg_DEFB	chr8	7382556	T	C	118	31,00%	37			rs62639816	1,00E-012
NA12760	reg_DEFB	chr8	7382833	G	A	154	99,00%	152				1,00E-012
NA12760	reg_DEFB	chr8	7383272	A	C	134	20,00%	27			rs67373661	1,00E-012
NA12760	reg_DEFB	chr8	7383406	C	T	128	34,00%	44	rs4504661			1,00E-012
NA12760	reg_DEFB	chr8	7383498	T	C	124	21,00%	26	rs4392927			1,00E-012
NA12760	reg_DEFB	chr8	7383669	A	G	125	15,00%	19			rs66747636	1,02E-010
NA12760	reg_DEFB	chr8	7383694	G	A	126	19,00%	24				1,00E-012
NA12760	reg_DEFB	chr8	7384348	A	C	184	49,00%	90	rs2737932			1,00E-012
NA12760	reg_DEFB	chr8	7384398	C	A	178	29,00%	52				1,00E-012
NA12760	reg_DEFB	chr8	7384978	T	C	101	54,00%	55	rs2680541			1,00E-012
NA12760	reg_DEFB	chr8	7385749	G	A	56	39,00%	22	rs2737931			1,00E-012
NA12760	reg_DEFB	chr8	7386060	T	A	54	35,00%	19			rs62639820	1,00E-012
NA12760	reg_DEFB	chr8	7386182	T	G	54	52,00%	28	rs2737502			1,00E-012
NA12760	reg_DEFB	chr8	7386319	T	A	46	37,00%	17			rs62639821	1,00E-012
NA12760	reg_DEFB	chr8	7386388	T	C	42	21,00%	9	rs2017768			4,04E-007
NA12760	reg_DEFB	chr8	7386396	G	A	42	45,00%	19			rs62639822	1,00E-012
NA12760	reg_DEFB	chr8	7386641	T	C	35	57,00%	20	rs2737503			1,00E-012
NA12760	reg_DEFB	chr8	7386669	A	G	42	36,00%	15			rs62639824	1,00E-012
NA12760	reg_DEFB	chr8	7386904	T	C	98	11,00%	11				1,69E-005
NA12760	reg_DEFB	chr8	7386935	A	G	103	28,00%	29			rs62639825	1,00E-012
NA12760	reg_DEFB	chr8	7387038	C	A	124	28,00%	35	rs3175182			1,00E-012
NA12760	reg_DEFB	chr8	7387256	A	G	142	13,00%	18	rs1054672			5,98E-009
NA12760	reg_DEFB	chr8	7387328	T	G	133	29,00%	39	rs4840284			1,00E-012
NA12760	reg_DEFB	chr8	7387420	C	G	131	51,00%	67	rs2258428			1,00E-012
NA12760	reg_DEFB	chr8	7387897	G	A	32	19,00%	6	rs2737506			8,02E-005
NA12760	reg_DEFB	chr8	7387908	G	A	32	53,00%	17	rs2737507			1,00E-012
NA12760	reg_DEFB	chr8	7388003	A	G	26	31,00%	8	rs2737508			8,44E-008
NA12760	reg_DEFB	chr8	7388670	T	C	51	22,00%	11	rs2737511	rs2680539		1,94E-008
NA12760	reg_DEFB	chr8	7388966	G	C	40	22,00%	9				2,58E-007
NA12760	reg_DEFB	chr8	7389499	A	G	80	59,00%	47	rs2075896			1,00E-012
NA12760	reg_DEFB	chr8	7389589	G	A	82	43,00%	35	rs2737512			1,00E-012
NA12760	reg_DEFB	chr8	7389644	G	T	98	32,00%	31				1,00E-012
NA12760	reg_DEFB	chr8	7390205	A	G	123	19,00%	23	rs2737513			1,00E-012
NA12760	reg_DEFB	chr8	7390502	G	A	136	34,00%	46	rs2737514	rs4840775		1,00E-012
NA12760	reg_DEFB	chr8	7390503	C	G	137	28,00%	38	rs4840775			1,00E-012
NA12760	reg_DEFB	chr8	7390657	G	A	125	30,00%	38			rs2737889	1,00E-012
NA12760	reg_DEFB	chr8	7390776	G	A	123	80,00%	98	rs2737515			1,00E-012
NA12760	reg_DEFB	chr8	7390893	A	T	112	37,00%	41			rs62639831	1,00E-012
NA12760	reg_DEFB	chr8	7391064	T	G	73	100,00%	73	rs4840776			1,00E-012

NA12760	reg_DEFB	chr8	7391191	T	C	25	68,00%	17							rs71511284	1,00E-012
NA12760	reg_CTRL	chr8	8214235	A	G	23	57,00%	13			-	PRAGMIN	rs6601691			1,00E-012
NA12760	reg_CTRL	chr8	8214527	A	G	48	52,00%	25			-	PRAGMIN	rs6996347			1,00E-012
NA12760	reg_CTRL	chr8	8214897	A	C	43	60,00%	26			-	PRAGMIN	rs4840922			1,00E-012
NA12760	reg_CTRL	chr8	8215271	G	A	24	62,00%	15			-	PRAGMIN	rs2980503			1,00E-012
NA12760	reg_CTRL	chr8	8215510	T	C	33	55,00%	18			-	PRAGMIN	rs13276576			1,00E-012
NA12760	reg_CTRL	chr8	8215633	T	C	37	100,00%	37			-	PRAGMIN	rs1548198			1,00E-012
NA12760	reg_CTRL	chr8	8217059	C	G	30	67,00%	20			-	PRAGMIN	rs4840923			1,00E-012
NA12760	reg_CTRL	chr8	8218156	G	A	28	61,00%	17			-	PRAGMIN	rs12674854			1,00E-012
NA12760	reg_CTRL	chr8	8218928	G	C	43	51,00%	22			-	PRAGMIN	rs867757			1,00E-012
NA12760	reg_CTRL	chr8	8218968	A	T	41	49,00%	20			-	PRAGMIN	rs2945898			1,00E-012
NA12760	reg_CTRL	chr8	8219680	G	C	19	63,00%	12			-	PRAGMIN	rs939069			1,00E-012
NA12760	reg_CTRL	chr8	8223156	T	C	12	50,00%	6	H	R	-2	PRAGMIN			rs56215812	1,21E-007
NA12760	reg_CTRL	chr8	8223577	T	C	62	35,00%	22			-	PRAGMIN	rs2979208			1,00E-012
NA12760	reg_CTRL	chr8	8223747	A	T	52	40,00%	21			-	PRAGMIN	rs2976910			1,00E-012
NA12760	reg_CTRL	chr8	8223842	C	T	39	54,00%	21			-	PRAGMIN	rs10099374			1,00E-012
NA12760	reg_CTRL	chr8	8224229	G	T	13	31,00%	4			-	PRAGMIN	rs2945900			1,69E-004
NA12760	reg_CTRL	chr8	8224259	T	C	15	27,00%	4			-	PRAGMIN				3,12E-004
NA12760	reg_CTRL	chr8	8224912	A	G	40	30,00%	12			-	PRAGMIN	rs2979210			7,20E-011
NA12760	reg_CTRL	chr8	8224960	T	C	34	35,00%	12			-	PRAGMIN	rs2945901			1,59E-011
NA12760	reg_CTRL	chr8	8225163	A	G	26	42,00%	11			-	PRAGMIN	rs2980498	rs2945902		9,51E-012
NA12760	reg_CTRL	chr8	8225164	T	C	24	42,00%	10			-	PRAGMIN	rs2945902			6,17E-011
NA12760	reg_CTRL	chr8	8225230	T	C	21	52,00%	11			-	PRAGMIN	rs2945903			1,00E-012
NA12760	reg_CTRL	chr8	8225641	G	A	29	48,00%	14			-	PRAGMIN	rs2979211			1,00E-012
NA12760	reg_CTRL	chr8	8225874	G	A	31	39,00%	12			-	PRAGMIN	rs2979212			4,09E-012
NA12760	reg_CTRL	chr8	8225954	G	A	44	45,00%	20			-	PRAGMIN	rs2979213			1,00E-012
NA12760	reg_CTRL	chr8	8225986	A	G	45	51,00%	23			-	PRAGMIN	rs2976917			1,00E-012
NA12760	reg_CTRL	chr8	8226005	A	G	45	42,00%	19			-	PRAGMIN	rs2976918			1,00E-012
NA12760	reg_CTRL	chr8	8226193	A	G	54	50,00%	27			-	PRAGMIN	rs2979215			1,00E-012
NA12760	reg_CTRL	chr8	8226315	T	C	66	47,00%	31			-	PRAGMIN	rs2979216			1,00E-012
NA12760	reg_CTRL	chr8	8226494	G	A	71	55,00%	39			-	PRAGMIN	rs2979217			1,00E-012
NA12760	reg_CTRL	chr8	8226774	G	C	55	53,00%	29			-	PRAGMIN	rs2979218			1,00E-012
NA12760	reg_CTRL	chr8	8226802	G	A	55	56,00%	31			-	PRAGMIN	rs2979219			1,00E-012
NA12760	reg_CTRL	chr8	8226809	A	G	56	57,00%	32			-	PRAGMIN	rs2976927			1,00E-012
NA12760	reg_CTRL	chr8	8226848	T	C	58	100,00%	58			-	PRAGMIN	rs2980497			1,00E-012
NA12760	reg_CTRL	chr8	8226912	G	A	48	60,00%	29			-	PRAGMIN	rs2945904			1,00E-012
NA12760	reg_CTRL	chr8	8226996	T	G	44	61,00%	27			-	PRAGMIN	rs2945905			1,00E-012
NA12760	reg_CTRL	chr8	8227475	T	C	35	43,00%	15			-	PRAGMIN	rs2945907			1,00E-012
NA12760	reg_CTRL	chr8	8227809	T	G	62	48,00%	30			-	PRAGMIN	rs1518992			1,00E-012
NA12760	reg_CTRL	chr8	8227825	T	C	63	48,00%	30			-	PRAGMIN	rs1850724			1,00E-012
NA12760	reg_CTRL	chr8	8227900	C	G	70	39,00%	27			-	PRAGMIN	rs1850725			1,00E-012
NA12760	reg_CTRL	chr8	8227975	A	G	67	43,00%	29			-	PRAGMIN	rs1850726			1,00E-012
NA12760	reg_CTRL	chr8	8228199	T	C	48	54,00%	26			-	PRAGMIN	rs2980496			1,00E-012
NA12760	reg_CTRL	chr8	8228481	T	G	64	50,00%	32			-	PRAGMIN	rs2979220			1,00E-012
NA12760	reg_CTRL	chr8	8229144	C	T	25	32,00%	8			-	PRAGMIN	rs2980495			5,97E-008
NA12760	reg_CTRL	chr8	8229293	T	C	19	37,00%	7			-	PRAGMIN	rs2979221			1,34E-007
NA12760	reg_CTRL	chr8	8229301	G	A	18	39,00%	7			-	PRAGMIN	rs2976947			8,67E-008
NA12760	reg_CTRL	chr8	8229639	A	C	38	45,00%	17			-	PRAGMIN	rs2945908			1,00E-012
NA12760	reg_CTRL	chr8	8229935	T	C	32	53,00%	17			-	PRAGMIN	rs2976952			1,00E-012
NA12760	reg_CTRL	chr8	8230241	T	C	35	40,00%	14			-	PRAGMIN	rs2976954			1,00E-012
NA12760	reg_CTRL	chr8	8230250	A	C	35	40,00%	14			-	PRAGMIN	rs2979222			1,00E-012
NA12760	reg_CTRL	chr8	8230631	T	C	17	41,00%	7			-	PRAGMIN	rs2980494			5,41E-008
NA12760	reg_CTRL	chr8	8230864	G	A	28	100,00%	28			-	PRAGMIN	rs2979223			1,00E-012

NA12760	reg_CTRL	chr8	8231138	C	T	47	45,00%	21	-	PRAGMIN	rs2979224			1,00E-012
NA12760	reg_CTRL	chr8	8231195	T	G	50	42,00%	21	-	PRAGMIN	rs2976963			1,00E-012
NA12760	reg_CTRL	chr8	8232030	A	C	14	57,00%	8	-	PRAGMIN	rs2979226			2,09E-010
NA12760	reg_CTRL	chr8	8232142	T	G	21	62,00%	13	-	PRAGMIN	rs9329268	rs9329269		1,00E-012
NA12760	reg_CTRL	chr8	8232143	T	C	23	65,00%	15	-	PRAGMIN	rs9329269			1,00E-012
NA12760	reg_CTRL	chr8	8232156	C	G	25	60,00%	15	-	PRAGMIN	rs9329270			1,00E-012
NA12760	reg_CTRL	chr8	8232408	G	A	52	54,00%	28	-	PRAGMIN	rs2976840			1,00E-012
NA12760	reg_CTRL	chr8	8232580	A	C	36	50,00%	18	-	PRAGMIN	rs2945910			1,00E-012
NA12760	reg_CTRL	chr8	8233348	C	T	66	41,00%	27	-	PRAGMIN	rs2976852			1,00E-012
NA12760	reg_CTRL	chr8	8233599	T	C	83	35,00%	29	-	PRAGMIN	rs2980491			1,00E-012
NA12760	reg_CTRL	chr8	8235132	A	G	89	51,00%	45	-	PRAGMIN	rs2945912			1,00E-012
NA12760	reg_CTRL	chr8	8235635	C	T	63	51,00%	32	-	PRAGMIN	rs2945913			1,00E-012
NA12760	reg_CTRL	chr8	8235716	C	G	55	55,00%	30	-	PRAGMIN	rs4840337			1,00E-012
NA12760	reg_CTRL	chr8	8235760	T	G	55	44,00%	24	-	PRAGMIN	rs2980490			1,00E-012
NA12760	reg_CTRL	chr8	8236281	C	T	63	38,00%	24	-	PRAGMIN	rs2976887			1,00E-012
NA12760	reg_CTRL	chr8	8236848	A	C	8	100,00%	8	-	PRAGMIN	rs2945914			1,00E-012
NA12760	reg_CTRL	chr8	8237074	T	C	5	60,00%	3	-	PRAGMIN			rs67646927	1,18E-004
NA12760	reg_CTRL	chr8	8237374	G	A	37	46,00%	17	-	PRAGMIN			rs62496027	1,00E-012
NA12760	reg_CTRL	chr8	8238677	A	G	24	100,00%	24	-	PRAGMIN	rs2176631			1,00E-012
NA12760	reg_CTRL	chr8	8238782	A	G	28	61,00%	17	-	PRAGMIN	rs2945839			1,00E-012
NA12760	reg_CTRL	chr8	8239704	A	G	21	33,00%	7	-	PRAGMIN	rs2980489			2,98E-007
NA12760	reg_CTRL	chr8	8240257	T	C	21	52,00%	11	-	PRAGMIN				1,00E-012
NA12760	reg_CTRL	chr8	8242420	C	T	56	98,00%	55	-	PRAGMIN	rs11785239			1,00E-012
NA12760	reg_CTRL	chr8	8242644	A	T	42	62,00%	26	-	PRAGMIN	rs13273161			1,00E-012
NA12760	reg_CTRL	chr8	8243226	C	T	67	100,00%	67	-	PRAGMIN	rs6990504			1,00E-012
NA12760	reg_CTRL	chr8	8244163	A	G	21	76,00%	16	-	PRAGMIN	rs7833103			1,00E-012
NA12760	reg_CTRL	chr8	8244364	G	A	11	64,00%	7	-	PRAGMIN				1,04E-009
NA12760	reg_CTRL	chr8	8244749	A	G	42	52,00%	22	-	PRAGMIN	rs17150353			1,00E-012
NA12760	reg_CTRL	chr8	8246169	G	C	37	57,00%	21	-	PRAGMIN	rs11786306			1,00E-012
NA12760	reg_CTRL	chr8	8246601	G	T	92	99,00%	91	-	PRAGMIN	rs4840932			1,00E-012
NA12760	reg_CTRL	chr8	8249567	T	C	29	55,00%	16	-	PRAGMIN	rs34796521			1,00E-012
NA12760	reg_CTRL	chr8	8252759	T	C	42	43,00%	18	-	PRAGMIN	rs4840939			1,00E-012
NA12760	reg_CTRL	chr8	8254396	A	G	51	100,00%	51	-	PRAGMIN	rs4840941			1,00E-012
NA12760	reg_CTRL	chr8	8254926	C	T	36	53,00%	19	-	PRAGMIN	rs11778125			1,00E-012
NA12760	reg_CTRL	chr8	8256007	T	C	36	97,00%	35	-	PRAGMIN	rs7005904			1,00E-012
NA12760	reg_CTRL	chr8	8256108	G	C	29	52,00%	15	-	PRAGMIN	rs10099225			1,00E-012
NA12760	reg_CTRL	chr8	8256279	T	C	19	26,00%	5	-	PRAGMIN	rs7006376			5,71E-005
NA12760	reg_CTRL	chr8	8256377	A	G	21	95,00%	20	-	PRAGMIN	rs4840338			1,00E-012
NA12760	reg_CTRL	chr8	8256592	G	A	60	100,00%	60	-	PRAGMIN	rs724265			1,00E-012
NA12760	reg_CTRL	chr8	8256759	G	A	80	46,00%	37	-	PRAGMIN	rs724266			1,00E-012
NA12760	reg_CTRL	chr8	8257851	T	C	16	62,00%	10	-	PRAGMIN			rs71513189	1,00E-012
NA12760	reg_CTRL	chr8	8258498	C	T	17	59,00%	10	-	PRAGMIN	rs1914826			1,00E-012
NA12760	reg_CTRL	chr8	8258721	C	G	29	48,00%	14	-	PRAGMIN	rs1914825			1,00E-012
NA12760	reg_CTRL	chr8	8258769	G	A	33	45,00%	15	-	PRAGMIN	rs1914824			1,00E-012
NA12760	reg_CTRL	chr8	8259602	T	G	86	45,00%	39	-	PRAGMIN	rs11996133			1,00E-012
NA12760	reg_CTRL	chr8	8259890	T	C	65	100,00%	65	-	PRAGMIN	rs2030279			1,00E-012
NA12760	reg_CTRL	chr8	8261308	T	C	23	57,00%	13	-	PRAGMIN	rs2979237			1,00E-012
NA12760	reg_CTRL	chr8	8261611	A	C	21	62,00%	13	-	PRAGMIN			rs36109453	1,00E-012
NA12760	reg_CTRL	chr8	8261787	T	A	24	58,00%	14	-	PRAGMIN	rs34628823			1,00E-012
NA12760	reg_CTRL	chr8	8261903	C	T	32	56,00%	18	-	PRAGMIN	rs35038563			1,00E-012
NA12760	reg_CTRL	chr8	8264586	C	A	58	57,00%	33	-	PRAGMIN	rs7841735			1,00E-012
NA12760	reg_CTRL	chr8	8265440	C	T	69	45,00%	31	-	PRAGMIN	rs2976972			1,00E-012
NA12760	reg_CTRL	chr8	8265655	C	A	55	58,00%	32	-	PRAGMIN	rs2979166			1,00E-012

NA12760	reg_CTRL	chr8	8265814	C	T	27	26,00%	7			-	PRAGMIN	rs10481460			2,02E-006
NA12760	reg_CTRL	chr8	8267228	C	G	49	61,00%	30			-	PRAGMIN	rs2979134			1,00E-012
NA12760	reg_CTRL	chr8	8268322	G	A	41	59,00%	24			-	PRAGMIN	rs2921009			1,00E-012
NA12760	reg_CTRL	chr8	8269101	A	G	74	55,00%	41			-	PRAGMIN	rs13280051			1,00E-012
NA12760	reg_CTRL	chr8	8269203	A	C	71	45,00%	32			-	PRAGMIN	rs4840946			1,00E-012
NA12760	reg_CTRL	chr8	8269553	T	C	65	57,00%	37			-	PRAGMIN	rs4840947			1,00E-012
NA12760	reg_CTRL	chr8	8269943	C	T	43	37,00%	16			-	PRAGMIN	rs4840948			1,00E-012
NA12760	reg_CTRL	chr8	8270279	G	A	18	50,00%	9			-	PRAGMIN	rs12546045			7,33E-011
NA12760	reg_CTRL	chr8	8270377	C	A	19	53,00%	10			-	PRAGMIN	rs12547958			4,83E-012
NA12760	reg_CTRL	chr8	8270536	G	C	26	46,00%	12			-	PRAGMIN	rs4840950			1,00E-012
NA12760	reg_CTRL	chr8	8270542	G	A	26	50,00%	13			-	PRAGMIN	rs4840951			1,00E-012
NA12760	reg_CTRL	chr8	8271487	G	C	17	59,00%	10	A	A	-1	PRAGMIN	rs4840952			1,00E-012
NA12760	reg_CTRL	chr8	8271602	G	C	15	60,00%	9	S	C	-1	PRAGMIN	rs4840953			1,11E-011
NA12760	reg_CTRL	chr8	8271628	C	T	16	56,00%	9	P	P	-1	PRAGMIN	rs41314930	rs4840954		2,54E-011
NA12760	reg_CTRL	chr8	8271629	G	A	16	56,00%	9	P	L	-1	PRAGMIN	rs4840954	rs4840955		2,54E-011
NA12760	reg_CTRL	chr8	8274087	C	T	34	100,00%	34			-	PRAGMIN	rs2976958			1,00E-012
NA12760	reg_CTRL	chr8	8274496	T	G	75	41,00%	31			-	PRAGMIN	rs3932318			1,00E-012
NA12760	reg_CTRL	chr8	8679890	G	A	36	100,00%	36			-	MFHAS1	rs4841038			1,00E-012
NA12760	reg_CTRL	chr8	8680797	G	A	17	94,00%	16			-	MFHAS1	rs10903311			1,00E-012
NA12760	reg_CTRL	chr8	8680992	C	G	57	100,00%	57			-	MFHAS1	rs2271340			1,00E-012
NA12760	reg_CTRL	chr8	8681135	T	A	71	97,00%	69			-	MFHAS1	rs2271341			1,00E-012
NA12760	reg_CTRL	chr8	8681348	C	T	66	100,00%	66			-	MFHAS1	rs2271342			1,00E-012
NA12760	reg_CTRL	chr8	8681684	G	C	34	97,00%	33			-	MFHAS1	rs12677543			1,00E-012
NA12760	reg_CTRL	chr8	8681732	G	A	35	100,00%	35			-	MFHAS1	rs12677550			1,00E-012
NA12760	reg_CTRL	chr8	8682101	T	C	30	93,00%	28			-	MFHAS1	rs7015606			1,00E-012
NA12760	reg_CTRL	chr8	8683135	G	C	66	98,00%	65			-	MFHAS1	rs2409088			1,00E-012
NA12760	reg_CTRL	chr8	8683656	T	C	21	100,00%	21			-	MFHAS1	rs12682352			1,00E-012
NA12760	reg_CTRL	chr8	8684040	C	T	43	65,00%	28			-	MFHAS1				1,00E-012
NA12760	reg_CTRL	chr8	8686807	T	C	30	100,00%	30			-	MFHAS1	rs2409089			1,00E-012
NA12760	reg_CTRL	chr8	8687291	C	T	34	91,00%	31			-	MFHAS1	rs11249891			1,00E-012
NA12760	reg_CTRL	chr8	8688829	T	C	33	100,00%	33			-	MFHAS1	rs6601732			1,00E-012
NA12760	reg_CTRL	chr8	8690299	G	A	57	96,00%	55			-	MFHAS1	rs7832968			1,00E-012
NA12760	reg_CTRL	chr8	8691268	G	A	47	98,00%	46			-	MFHAS1	rs2409090			1,00E-012
NA12760	reg_CTRL	chr8	8691467	G	A	6	83,00%	5			-	MFHAS1	rs2409091			3,79E-008
NA12760	reg_CTRL	chr8	8691521	C	G	6	100,00%	6			-	MFHAS1	rs7460947			1,48E-010
NA12760	reg_CTRL	chr8	8691937	T	C	13	100,00%	13			-	MFHAS1	rs4841040			1,00E-012
NA12760	reg_CTRL	chr8	8691951	C	G	15	100,00%	15			-	MFHAS1	rs4841041			1,00E-012
NA12760	reg_CTRL	chr8	8692433	C	T	35	94,00%	33			-	MFHAS1	rs3748144			1,00E-012
NA12760	reg_CTRL	chr8	8695314	T	A	66	44,00%	29			-	MFHAS1				1,00E-012
NA12760	reg_CTRL	chr8	8695950	A	G	19	95,00%	18			-	MFHAS1	rs2048419			1,00E-012
NA12760	reg_CTRL	chr8	8696864	C	T	76	50,00%	38			-	MFHAS1			rs62495537	1,00E-012
NA12760	reg_CTRL	chr8	8697085	G	T	74	100,00%	74			-	MFHAS1	rs13282015			1,00E-012
NA12760	reg_CTRL	chr8	8697948	C	A	27	100,00%	27			-	MFHAS1	rs6994038			1,00E-012
NA12760	reg_CTRL	chr8	8698944	T	C	55	100,00%	55			-	MFHAS1	rs12547493			1,00E-012
NA12760	reg_CTRL	chr8	8699091	C	G	79	100,00%	79			-	MFHAS1	rs12544992			1,00E-012
NA12760	reg_CTRL	chr8	8700625	C	T	15	73,00%	11			-	MFHAS1	rs28399241			1,00E-012
NA12760	reg_CTRL	chr8	8701507	C	T	33	97,00%	32			-	MFHAS1	rs9329167			1,00E-012
NA12760	reg_CTRL	chr8	8702026	C	T	26	23,00%	6			-	MFHAS1				2,29E-005
NA12760	reg_CTRL	chr8	8702028	T	A	27	22,00%	6			-	MFHAS1				2,89E-005
NA12760	reg_CTRL	chr8	8702032	G	A	26	96,00%	25			-	MFHAS1	rs4841042			1,00E-012
NA12760	reg_CTRL	chr8	8702089	G	A	35	74,00%	26			-	MFHAS1	rs4841043			1,00E-012
NA12760	reg_CTRL	chr8	8702110	G	A	35	26,00%	9			-	MFHAS1				7,40E-008
NA12760	reg_CTRL	chr8	8702129	C	A	35	26,00%	9			-	MFHAS1				7,40E-008

NA12760	reg_CTRL	chr8	8702133	C	T	34	26,00%	9	-	MFHAS1		rs6982682	5,61E-008
NA12760	reg_CTRL	chr8	8702350	G	A	47	96,00%	45	-	MFHAS1	rs4841044		1,00E-012
NA12760	reg_CTRL	chr8	8702557	G	A	51	94,00%	48	-	MFHAS1	rs11783966		1,00E-012
NA12760	reg_CTRL	chr8	8703143	T	A	9	67,00%	6	-	MFHAS1	rs9644775		1,17E-008
NA12760	reg_CTRL	chr8	8703212	T	C	12	67,00%	8	-	MFHAS1	rs9644776		4,67E-011
NA12760	reg_CTRL	chr8	8704326	C	T	30	100,00%	30	-	MFHAS1	rs6988939		1,00E-012
NA12760	reg_CTRL	chr8	8704329	G	T	30	97,00%	29	-	MFHAS1	rs2175161		1,00E-012
NA12760	reg_CTRL	chr8	8704854	C	T	56	100,00%	56	-	MFHAS1	rs6993494		1,00E-012
NA12760	reg_CTRL	chr8	8705807	A	G	37	97,00%	36	-	MFHAS1	rs7006418		1,00E-012
NA12760	reg_CTRL	chr8	8705896	A	G	38	100,00%	38	-	MFHAS1	rs7006589		1,00E-012
NA12760	reg_CTRL	chr8	8706327	A	C	36	100,00%	36	-	MFHAS1	rs1473029		1,00E-012
NA12760	reg_CTRL	chr8	8707492	G	C	31	100,00%	31	-	MFHAS1	rs4840362		1,00E-012
NA12760	reg_CTRL	chr8	8707587	T	A	30	100,00%	30	-	MFHAS1	rs7823757		1,00E-012
NA12760	reg_CTRL	chr8	8708009	A	G	16	100,00%	16	-	MFHAS1		rs60315134	1,00E-012
NA12760	reg_CTRL	chr8	8708146	C	A	22	100,00%	22	-	MFHAS1		rs59046059	1,00E-012
NA12760	reg_CTRL	chr8	8709372	C	T	46	100,00%	46	-	MFHAS1	rs11784052		1,00E-012
NA12760	reg_CTRL	chr8	8709629	T	A	33	88,00%	29	-	MFHAS1	rs10088933		1,00E-012
NA12760	reg_CTRL	chr8	8709839	G	C	16	94,00%	15	-	MFHAS1	rs11777085		1,00E-012
NA12760	reg_CTRL	chr8	8709989	A	G	12	100,00%	12	-	MFHAS1	rs4841045		1,00E-012
NA12760	reg_CTRL	chr8	8710211	C	T	6	100,00%	6	-	MFHAS1	rs4841046		1,48E-010
NA12760	reg_CTRL	chr8	8710362	A	C	9	89,00%	8	-	MFHAS1	rs4841047		1,00E-012
NA12760	reg_CTRL	chr8	8710730	T	C	52	98,00%	51	-	MFHAS1	rs13265731		1,00E-012
NA12760	reg_CTRL	chr8	8711011	A	C	72	99,00%	71	-	MFHAS1	rs13259216		1,00E-012
NA12760	reg_CTRL	chr8	8711146	T	C	76	99,00%	75	-	MFHAS1	rs35431455		1,00E-012
NA12760	reg_CTRL	chr8	8712586	A	G	17	100,00%	17	-	MFHAS1	rs13260419		1,00E-012
NA12760	reg_CTRL	chr8	8712735	A	T	21	100,00%	21	-	MFHAS1	rs35039922		1,00E-012
NA12760	reg_CTRL	chr8	8713900	T	C	59	53,00%	31	-	MFHAS1	rs950721		1,00E-012
NA12760	reg_CTRL	chr8	8715940	G	A	37	97,00%	36	-	MFHAS1	rs882462		1,00E-012
NA12760	reg_CTRL	chr8	8716307	G	C	50	100,00%	50	-	MFHAS1	rs3827809		1,00E-012
NA12760	reg_CTRL	chr8	8716586	A	G	65	98,00%	64	-	MFHAS1	rs11775523		1,00E-012
NA12760	reg_CTRL	chr8	8716735	G	C	51	98,00%	50	-	MFHAS1	rs28755903		1,00E-012
NA12760	reg_CTRL	chr8	8716866	G	C	30	100,00%	30	-	MFHAS1	rs1039913		1,00E-012
NA12760	reg_CTRL	chr8	8716959	C	T	18	100,00%	18	-	MFHAS1	rs1039914		1,00E-012
NA12760	reg_CTRL	chr8	8717024	T	C	13	100,00%	13	-	MFHAS1	rs1039915		1,00E-012
NA12760	reg_CTRL	chr8	8717493	G	A	3	100,00%	3	-	MFHAS1	rs11779585		1,22E-005
NA12760	reg_CTRL	chr8	8717887	G	A	35	100,00%	35	-	MFHAS1		rs57312668	1,00E-012
NA12760	reg_CTRL	chr8	8718276	C	G	48	100,00%	48	-	MFHAS1	rs4840364		1,00E-012
NA12760	reg_CTRL	chr8	8718775	A	C	41	98,00%	40	-	MFHAS1	rs4841049		1,00E-012
NA12760	reg_CTRL	chr8	8719000	T	C	44	100,00%	44	-	MFHAS1	rs4841050		1,00E-012
NA12760	reg_CTRL	chr8	8719166	T	C	49	100,00%	49	-	MFHAS1	rs1876836		1,00E-012
NA12760	reg_CTRL	chr8	8719602	A	T	36	100,00%	36	-	MFHAS1	rs2409092		1,00E-012
NA12760	reg_CTRL	chr8	8720288	T	C	11	91,00%	10	-	MFHAS1	rs12545499		1,00E-012
NA12760	reg_CTRL	chr8	8720310	G	C	12	100,00%	12	-	MFHAS1	rs2409094		1,00E-012
NA12760	reg_CTRL	chr8	8720681	A	C	36	97,00%	35	-	MFHAS1	rs907179		1,00E-012
NA12760	reg_CTRL	chr8	8722363	G	A	87	100,00%	87	-	MFHAS1	rs1533059		1,00E-012
NA12760	reg_CTRL	chr8	8722600	A	G	50	98,00%	49	-	MFHAS1	rs1533058		1,00E-012
NA12760	reg_CTRL	chr8	8723056	T	C	59	100,00%	59	-	MFHAS1	rs4841051		1,00E-012
NA12760	reg_CTRL	chr8	8723264	A	G	65	100,00%	65	-	MFHAS1	rs1039916		1,00E-012
NA12760	reg_CTRL	chr8	8724090	T	A	37	95,00%	35	-	MFHAS1	rs2409095		1,00E-012
NA12760	reg_CTRL	chr8	8724255	C	A	24	46,00%	11	-	MFHAS1	rs3789850		3,07E-012
NA12760	reg_CTRL	chr8	8724464	G	C	28	61,00%	17	-	MFHAS1	rs3789849		1,00E-012
NA12760	reg_CTRL	chr8	8724497	A	G	32	22,00%	7	-	MFHAS1	rs3789848		6,91E-006
NA12760	reg_CTRL	chr8	8724735	A	G	32	59,00%	19	-	MFHAS1	rs7013471		1,00E-012

NA12760	reg_CTRL	chr8	8725871	T	C	23	48,00%	11	-	MFHAS1	rs13275083		1,66E-012
NA12760	reg_CTRL	chr8	8726186	G	A	48	42,00%	20	-	MFHAS1			1,00E-012
NA12760	reg_CTRL	chr8	8726960	G	A	38	26,00%	10	-	MFHAS1			1,09E-008
NA12760	reg_CTRL	chr8	8726963	C	T	32	66,00%	21	-	MFHAS1			1,00E-012
NA12760	reg_CTRL	chr8	8726976	G	A	28	89,00%	25	-	MFHAS1			1,00E-012
NA12760	reg_CTRL	chr8	8726978	G	A	28	100,00%	28	-	MFHAS1			1,00E-012
NA12760	reg_CTRL	chr8	8726980	G	A	30	93,00%	28	-	MFHAS1			1,00E-012
NA12760	reg_CTRL	chr8	8726982	G	A	29	86,00%	25	-	MFHAS1	rs28821557		1,00E-012
NA12760	reg_CTRL	chr8	8727030	A	G	14	50,00%	7	-	MFHAS1			1,01E-008
NA12760	reg_CTRL	chr8	8727280	T	C	32	62,00%	20	-	MFHAS1		rs57784779	1,00E-012
NA12760	reg_CTRL	chr8	8727376	G	C	53	70,00%	37	-	MFHAS1	rs4840366		1,00E-012
NA12760	reg_CTRL	chr8	8727494	A	C	45	62,00%	28	-	MFHAS1		rs73504221	1,00E-012
NA12760	reg_CTRL	chr8	8727500	G	A	45	60,00%	27	-	MFHAS1		rs73504222	1,00E-012
NA12760	reg_CTRL	chr8	8727803	G	T	35	100,00%	35	-	MFHAS1	rs11995244		1,00E-012
NA12760	reg_CTRL	chr8	8727836	T	C	38	100,00%	38	-	MFHAS1	rs13259619		1,00E-012
NA12760	reg_CTRL	chr8	8728197	C	T	45	49,00%	22	-	MFHAS1	rs13259070		1,00E-012
NA12760	reg_CTRL	chr8	8728321	G	A	57	47,00%	27	-	MFHAS1	rs4840367		1,00E-012
NA12760	reg_CTRL	chr8	8728410	G	A	53	62,00%	33	-	MFHAS1	rs4840368		1,00E-012
NA12760	reg_CTRL	chr8	8728607	T	A	60	47,00%	28	-	MFHAS1	rs4840369		1,00E-012
NA12760	reg_CTRL	chr8	8728688	T	G	52	46,00%	24	-	MFHAS1	rs4840370		1,00E-012
NA12760	reg_CTRL	chr8	8728794	C	G	37	100,00%	37	-	MFHAS1	rs9329169		1,00E-012
NA12760	reg_CTRL	chr8	8729032	T	A	26	73,00%	19	-	MFHAS1	rs13270070		1,00E-012
NA12760	reg_CTRL	chr8	8729476	G	C	47	100,00%	47	-	MFHAS1	rs1510932		1,00E-012
NA12760	reg_CTRL	chr8	8729887	T	C	40	32,00%	13	-	MFHAS1	rs2409096		8,21E-012
NA12760	reg_CTRL	chr8	8729950	C	G	41	100,00%	41	-	MFHAS1	rs1510933		1,00E-012
NA12760	reg_CTRL	chr8	8730145	T	C	48	46,00%	22	-	MFHAS1	rs7820478		1,00E-012
NA12760	reg_CTRL	chr8	8731603	C	G	41	98,00%	40	-	MFHAS1	rs13254903		1,00E-012
NA12760	reg_CTRL	chr8	8732984	C	T	60	100,00%	60	-	MFHAS1	rs6601265		1,00E-012
NA12760	reg_CTRL	chr8	8733859	T	G	58	48,00%	28	-	MFHAS1	rs1510934		1,00E-012
NA12760	reg_CTRL	chr8	8735502	C	T	41	41,00%	17	-	MFHAS1	rs4841054		1,00E-012
NA12760	reg_CTRL	chr8	8736571	A	G	38	97,00%	37	-	MFHAS1	rs4841055		1,00E-012
NA12760	reg_CTRL	chr8	8737167	T	A	28	57,00%	16	-	MFHAS1	rs7820146		1,00E-012
NA12760	reg_CTRL	chr8	8737171	C	T	28	61,00%	17	-	MFHAS1	rs7833171		1,00E-012
NA12760	reg_CTRL	chr8	8737668	G	A	58	36,00%	21	-	MFHAS1		rs73190070	1,00E-012
NA12760	reg_CTRL	chr8	8738012	G	C	21	62,00%	13	-	MFHAS1	rs7017006		1,00E-012
NA12760	reg_CTRL	chr8	8738114	C	G	17	41,00%	7	-	MFHAS1		rs12265954	5,41E-008
NA12760	reg_CTRL	chr8	8738166	C	G	12	33,00%	4	-	MFHAS1		rs11995330	1,19E-004
NA12760	reg_CTRL	chr8	8738261	C	T	7	100,00%	7	-	MFHAS1	rs11249893		3,40E-012
NA12760	reg_CTRL	chr8	8738469	C	T	23	48,00%	11	-	MFHAS1		rs73190071	1,66E-012
NA12760	reg_CTRL	chr8	8739698	G	C	72	47,00%	34	-	MFHAS1			1,00E-012
NA12760	reg_CTRL	chr8	8740017	G	C	67	55,00%	37	-	MFHAS1	rs7820738		1,00E-012
NA12760	reg_CTRL	chr8	8740237	A	G	55	56,00%	31	-	MFHAS1	rs907180		1,00E-012
NA12760	reg_CTRL	chr8	8740285	T	C	53	58,00%	31	-	MFHAS1	rs907181		1,00E-012
NA12760	reg_CTRL	chr8	8741091	C	T	8	100,00%	8	-	MFHAS1	rs6996376		1,00E-012
NA12760	reg_CTRL	chr8	8741740	G	C	72	100,00%	72	-	MFHAS1	rs4481596		1,00E-012
NA12760	reg_CTRL	chr8	8742375	G	T	39	62,00%	24	-	MFHAS1	rs11249894		1,00E-012
NA12760	reg_CTRL	chr8	8742590	C	T	82	48,00%	39	-	MFHAS1	rs11249895		1,00E-012
NA12760	reg_CTRL	chr8	8743742	A	C	37	41,00%	15	-	MFHAS1	rs408459		1,00E-012
NA12760	reg_CTRL	chr8	8743837	A	T	24	67,00%	16	-	MFHAS1			1,00E-012
NA12760	reg_CTRL	chr8	8744430	G	A	34	32,00%	11	-	MFHAS1			1,76E-010
NA12760	reg_CTRL	chr8	8744607	C	G	51	43,00%	22	-	MFHAS1	rs1877119		1,00E-012
NA12760	reg_CTRL	chr8	8745380	C	A	62	40,00%	25	-	MFHAS1	rs387706		1,00E-012
NA12760	reg_CTRL	chr8	8746124	A	C	81	100,00%	81	-	MFHAS1	rs440788		1,00E-012

NA12760	reg_CTRL	chr8	8746384	C	G	89	47,00%	42	-	MFHAS1	rs3925830		1,00E-012
NA12760	reg_CTRL	chr8	8746560	T	G	68	47,00%	32	-	MFHAS1	rs451082		1,00E-012
NA12760	reg_CTRL	chr8	8747166	G	C	21	67,00%	14	-	MFHAS1	rs1964719		1,00E-012
NA12760	reg_CTRL	chr8	8747381	C	T	26	46,00%	12	-	MFHAS1	rs3958877		1,00E-012
NA12760	reg_CTRL	chr8	8748211	G	A	27	100,00%	27	-	MFHAS1	rs437895		1,00E-012
NA12760	reg_CTRL	chr8	8749016	T	A	14	29,00%	4	-	MFHAS1	rs1251003		2,33E-004
NA12760	reg_CTRL	chr8	8749357	A	C	7	100,00%	7	-	MFHAS1	rs435953		3,40E-012
NA12760	reg_CTRL	chr8	8750151	G	A	18	61,00%	11	-	MFHAS1			1,00E-012
NA12760	reg_CTRL	chr8	8750305	A	G	32	56,00%	18	-	MFHAS1	rs4348501		1,00E-012
NA12760	reg_CTRL	chr8	8750416	G	A	32	97,00%	31	-	MFHAS1	rs231188		1,00E-012
NA12760	reg_CTRL	chr8	8750448	C	T	25	96,00%	24	-	MFHAS1	rs4523255		1,00E-012
NA12760	reg_CTRL	chr8	8751363	T	C	47	45,00%	21	-	MFHAS1		rs56073940	1,00E-012
NA12760	reg_CTRL	chr8	8753576	G	T	54	30,00%	16	-	MFHAS1	rs332029		1,00E-012
NA12760	reg_CTRL	chr8	8755553	C	T	72	50,00%	36	-	MFHAS1			1,00E-012
NA12760	reg_CTRL	chr8	8756260	G	A	93	57,00%	53	-	MFHAS1	rs1039917		1,00E-012
NA12760	reg_CTRL	chr8	8756622	A	C	68	53,00%	36	-	MFHAS1			1,00E-012
NA12760	reg_CTRL	chr8	8756923	G	A	54	46,00%	25	-	MFHAS1	rs35900578		1,00E-012
NA12760	reg_CTRL	chr8	8758831	C	T	31	48,00%	15	-	MFHAS1			1,00E-012
NA12760	reg_CTRL	chr8	8758883	G	A	30	100,00%	30	-	MFHAS1	rs4382480		1,00E-012
NA12760	reg_CTRL	chr8	8759937	G	A	32	47,00%	15	-	MFHAS1		rs56367294	1,00E-012
NA12760	reg_CTRL	chr8	8760085	C	T	17	100,00%	17	-	MFHAS1	rs332037		1,00E-012
NA12760	reg_CTRL	chr8	8760419	G	A	54	43,00%	23	-	MFHAS1			1,00E-012
NA12760	reg_CTRL	chr8	8761061	C	G	37	100,00%	37	-	MFHAS1	rs332039		1,00E-012
NA12760	reg_CTRL	chr8	8761328	G	T	21	43,00%	9	-	MFHAS1	rs3789845		4,19E-010
NA12760	reg_CTRL	chr8	8761521	A	G	50	50,00%	25	-	MFHAS1	rs3789844		1,00E-012
NA12760	reg_CTRL	chr8	8761667	C	T	65	100,00%	65	-	MFHAS1	rs3789843		1,00E-012
NA12760	reg_CTRL	chr8	8761686	C	T	64	100,00%	64	-	MFHAS1	rs3827806		1,00E-012
NA12760	reg_CTRL	chr8	8761706	A	C	62	53,00%	33	-	MFHAS1			1,00E-012
NA12760	reg_CTRL	chr8	8761825	C	T	57	98,00%	56	-	MFHAS1		rs60707155	1,00E-012
NA12760	reg_CTRL	chr8	8762536	G	T	19	89,00%	17	-	MFHAS1	rs7017599		1,00E-012
NA12760	reg_CTRL	chr8	8762639	G	A	15	93,00%	14	-	MFHAS1	rs1821007		1,00E-012
NA12760	reg_CTRL	chr8	8762729	G	A	24	92,00%	22	-	MFHAS1	rs1821008		1,00E-012
NA12760	reg_CTRL	chr8	8764214	G	T	92	95,00%	87	-	MFHAS1	rs1567398		1,00E-012
NA12760	reg_CTRL	chr8	8766603	A	C	52	46,00%	24	-	MFHAS1	rs13274028		1,00E-012
NA12760	reg_CTRL	chr8	8766641	C	G	60	52,00%	31	-	MFHAS1		rs73524014	1,00E-012
NA12760	reg_CTRL	chr8	8766707	G	A	68	50,00%	34	-	MFHAS1		rs73524015	1,00E-012
NA12760	reg_CTRL	chr8	8767171	G	C	57	100,00%	57	-	MFHAS1	rs907183		1,00E-012
NA12760	reg_CTRL	chr8	8767310	G	A	49	51,00%	25	-	MFHAS1	rs10098667		1,00E-012
NA12760	reg_CTRL	chr8	8767552	A	G	55	42,00%	23	-	MFHAS1	rs11991673		1,00E-012
NA12760	reg_CTRL	chr8	8767898	G	A	42	100,00%	42	-	MFHAS1	rs332040		1,00E-012
NA12760	reg_CTRL	chr8	8768326	G	C	56	95,00%	53	-	MFHAS1	rs4841058		1,00E-012
NA12760	reg_CTRL	chr8	8768646	G	A	56	41,00%	23	-	MFHAS1		rs61591712	1,00E-012
NA12760	reg_CTRL	chr8	8769293	C	T	47	81,00%	38	-	MFHAS1	rs9644694		1,00E-012
NA12760	reg_CTRL	chr8	8770589	T	C	32	41,00%	13	-	MFHAS1	rs4840372		1,00E-012
NA12760	reg_CTRL	chr8	8770735	G	C	42	100,00%	42	-	MFHAS1	rs2009455		1,00E-012
NA12760	reg_CTRL	chr8	8771021	G	C	85	49,00%	42	-	MFHAS1	rs9644671		1,00E-012
NA12760	reg_CTRL	chr8	8772507	A	C	59	100,00%	59	-	MFHAS1	rs10046783		1,00E-012
NA12760	reg_CTRL	chr8	8772623	A	C	70	97,00%	68	-	MFHAS1	rs10046784		1,00E-012
NA12760	reg_CTRL	chr8	8773796	A	G	57	100,00%	57	-	MFHAS1	rs12679021		1,00E-012
NA12760	reg_CTRL	chr8	8774098	G	A	24	29,00%	7	-	MFHAS1	rs12681432		8,35E-007
NA12760	reg_CTRL	chr8	8774113	A	T	23	74,00%	17	-	MFHAS1	rs7824578		1,00E-012
NA12760	reg_CTRL	chr8	8774196	G	A	27	33,00%	9	-	MFHAS1	rs13261380		5,80E-009
NA12760	reg_CTRL	chr8	8774295	G	T	37	43,00%	16	-	MFHAS1	rs34599909		1,00E-012

NA12760	reg_CTRL	chr8	8774325	G	T	43	53,00%	23			-	MFHAS1			rs60965369	1,00E-012
NA12760	reg_CTRL	chr8	8774535	T	C	70	56,00%	39			-	MFHAS1			rs73192206	1,00E-012
NA12760	reg_CTRL	chr8	8774874	T	G	68	96,00%	65			-	MFHAS1	rs409997			1,00E-012
NA12760	reg_CTRL	chr8	8775015	T	C	60	98,00%	59			-	MFHAS1	rs410487			1,00E-012
NA12760	reg_CTRL	chr8	8775059	C	T	58	57,00%	33			-	MFHAS1	rs1533100			1,00E-012
NA12760	reg_CTRL	chr8	8776018	C	G	38	42,00%	16			-	MFHAS1			rs72626639	1,00E-012
NA12760	reg_CTRL	chr8	8776268	A	G	55	49,00%	27			-	MFHAS1				1,00E-012
NA12760	reg_CTRL	chr8	8780758	G	T	22	95,00%	21			-	MFHAS1	rs435393			1,00E-012
NA12760	reg_CTRL	chr8	8781650	G	A	37	59,00%	22			-	MFHAS1	rs7818276			1,00E-012
NA12760	reg_CTRL	chr8	8784947	T	C	82	98,00%	80			-	MFHAS1	rs399123			1,00E-012
NA12760	reg_CTRL	chr8	8785304	A	G	46	98,00%	45	L	P	-1	MFHAS1	rs429433			1,00E-012
NA12760	reg_CTRL	chr8	11738136	A	C	30	100,00%	30			-	CTSB	rs1142957	rs1142956		1,00E-012
NA12760	reg_CTRL	chr8	11738154	G	C	35	100,00%	35			-	CTSB	rs1736077			1,00E-012
NA12760	reg_CTRL	chr8	11738505	A	C	37	100,00%	37			-	CTSB	rs8005			1,00E-012
NA12760	reg_CTRL	chr8	11738739	G	A	10	100,00%	10			-	CTSB	rs11786618			1,00E-012
NA12760	reg_CTRL	chr8	11739251	A	C	61	97,00%	59			-	CTSB	rs1736078			1,00E-012
NA12760	reg_CTRL	chr8	11739415	A	T	50	100,00%	50			-	CTSB	rs9009			1,00E-012
NA12760	reg_CTRL	chr8	11739613	G	A	26	100,00%	26			-	CTSB	rs6730			1,00E-012
NA12760	reg_CTRL	chr8	11739951	T	C	33	100,00%	33			-	CTSB	rs8898			1,00E-012
NA12760	reg_CTRL	chr8	11743086	C	G	29	100,00%	29			-	CTSB	rs2294140			1,00E-012
NA12760	reg_CTRL	chr8	11744382	T	C	20	100,00%	20			-	CTSB	rs4840586			1,00E-012
NA12760	reg_CTRL	chr8	11744417	C	A	21	95,00%	20			-	CTSB	rs2645423			1,00E-012
NA12760	reg_CTRL	chr8	11755710	T	C	32	47,00%	15			-	CTSB				1,00E-012
NA12760	reg_CTRL	chr8	11760540	A	G	32	100,00%	32			-	CTSB	rs1293307			1,00E-012
NA12760	reg_CTRL	chr8	11761184	G	A	20	35,00%	7			-	CTSB	rs1293309			2,03E-007
NA12760	reg_CTRL	chr8	12624420	G	A	70	100,00%	70			-	LONRF1	rs7005881			1,00E-012
NA12760	reg_CTRL	chr8	12625320	T	C	58	52,00%	30			-	LONRF1			rs73202627	1,00E-012
NA12760	reg_CTRL	chr8	12626051	T	C	64	100,00%	64			-	LONRF1	rs4831767			1,00E-012
NA12760	reg_CTRL	chr8	12626226	C	T	66	53,00%	35			-	LONRF1	rs4831768			1,00E-012
NA12760	reg_CTRL	chr8	12626235	A	G	66	98,00%	65			-	LONRF1	rs4831769			1,00E-012
NA12760	reg_CTRL	chr8	12627074	T	A	57	100,00%	57			-	LONRF1	rs10429335			1,00E-012
NA12760	reg_CTRL	chr8	12627236	A	G	49	59,00%	29			-	LONRF1			rs55752837	1,00E-012
NA12760	reg_CTRL	chr8	12627915	C	T	75	49,00%	37			-	LONRF1	rs11782145			1,00E-012
NA12760	reg_CTRL	chr8	12628025	T	C	78	47,00%	37			-	LONRF1	rs10100866			1,00E-012
NA12760	reg_CTRL	chr8	12628174	G	C	57	44,00%	25			-	LONRF1			rs73202629	1,00E-012
NA12760	reg_CTRL	chr8	12628721	A	G	34	35,00%	12			-	LONRF1	rs9632851			1,59E-011
NA12760	reg_CTRL	chr8	12629393	C	A	30	50,00%	15			-	LONRF1			rs73202633	1,00E-012
NA12760	reg_CTRL	chr8	12629532	A	T	52	96,00%	50			-	LONRF1	rs6530953			1,00E-012
NA12760	reg_CTRL	chr8	12629807	C	G	93	56,00%	52			-	LONRF1	rs7010337			1,00E-012
NA12760	reg_CTRL	chr8	12630196	A	T	87	45,00%	39			-	LONRF1	rs4831770			1,00E-012
NA12760	reg_CTRL	chr8	12630632	A	G	54	50,00%	27			-	LONRF1	rs3802268			1,00E-012
NA12760	reg_CTRL	chr8	12630635	A	C	54	50,00%	27			-	LONRF1			rs73202635	1,00E-012
NA12760	reg_CTRL	chr8	12631165	G	C	49	14,00%	7	T	S	-3	LONRF1				1,25E-004
NA12760	reg_CTRL	chr8	12631166	T	A	49	14,00%	7	T	S	-3	LONRF1				1,25E-004
NA12760	reg_CTRL	chr8	12631700	T	C	44	48,00%	21			-	LONRF1	rs17761564			1,00E-012
NA12760	reg_CTRL	chr8	12631939	T	C	37	59,00%	22			-	LONRF1			rs56114121	1,00E-012
NA12760	reg_CTRL	chr8	12632233	T	A	66	61,00%	40			-	LONRF1	rs17761606			1,00E-012
NA12760	reg_CTRL	chr8	12632654	A	C	48	98,00%	47			-	LONRF1	rs6995647			1,00E-012
NA12760	reg_CTRL	chr8	12633550	C	A	51	100,00%	51			-	LONRF1	rs3802269			1,00E-012
NA12760	reg_CTRL	chr8	12634490	G	C	62	35,00%	22			-	LONRF1	rs4272378			1,00E-012
NA12760	reg_CTRL	chr8	12634940	G	A	27	100,00%	27			-	LONRF1	rs7463601			1,00E-012
NA12760	reg_CTRL	chr8	12634992	A	G	31	97,00%	30			-	LONRF1	rs7461006			1,00E-012
NA12760	reg_CTRL	chr8	12635185	A	G	34	100,00%	34			-	LONRF1	rs6530956			1,00E-012

NA12760	reg_CTRL	chr8	12635399	G	A	37	95,00%	35			-	LONRF1	rs6530958		1,00E-012
NA12760	reg_CTRL	chr8	12635958	A	C	38	95,00%	36			-	LONRF1	rs4258004		1,00E-012
NA12760	reg_CTRL	chr8	12636319	C	T	66	100,00%	66			-	LONRF1	rs6530959		1,00E-012
NA12760	reg_CTRL	chr8	12637327	C	A	57	100,00%	57			-	LONRF1	rs13251315		1,00E-012
NA12760	reg_CTRL	chr8	12637416	A	G	54	94,00%	51			-	LONRF1	rs13272425		1,00E-012
NA12760	reg_CTRL	chr8	12637991	T	C	45	100,00%	45			-	LONRF1	rs9325786		1,00E-012
NA12760	reg_CTRL	chr8	12640631	C	T	56	100,00%	56			-	LONRF1	rs7014187		1,00E-012
NA12760	reg_CTRL	chr8	12641320	A	G	38	42,00%	16			-	LONRF1	rs4831777		1,00E-012
NA12760	reg_CTRL	chr8	12641415	C	G	46	61,00%	28			-	LONRF1		rs73202639	1,00E-012
NA12760	reg_CTRL	chr8	12641627	G	A	71	56,00%	40			-	LONRF1			1,00E-012
NA12760	reg_CTRL	chr8	12642348	C	A	56	66,00%	37			-	LONRF1	rs6530962		1,00E-012
NA12760	reg_CTRL	chr8	12642503	A	C	55	51,00%	28			-	LONRF1	rs4625037		1,00E-012
NA12760	reg_CTRL	chr8	12642559	T	C	57	51,00%	29			-	LONRF1	rs11775169		1,00E-012
NA12760	reg_CTRL	chr8	12642979	C	T	38	100,00%	38			-	LONRF1	rs4831354		1,00E-012
NA12760	reg_CTRL	chr8	12643126	C	T	31	48,00%	15			-	LONRF1	rs7819033		1,00E-012
NA12760	reg_CTRL	chr8	12643453	A	C	48	98,00%	47			-	LONRF1	rs4831780		1,00E-012
NA12760	reg_CTRL	chr8	12643642	A	C	47	94,00%	44			-	LONRF1	rs10098734		1,00E-012
NA12760	reg_CTRL	chr8	12643654	G	A	48	96,00%	46			-	LONRF1	rs10110145		1,00E-012
NA12760	reg_CTRL	chr8	12643838	T	C	48	52,00%	25			-	LONRF1	rs7014429		1,00E-012
NA12760	reg_CTRL	chr8	12644977	T	A	35	46,00%	16			-	LONRF1		rs73202645	1,00E-012
NA12760	reg_CTRL	chr8	12644993	C	T	37	62,00%	23			-	LONRF1	rs7837242		1,00E-012
NA12760	reg_CTRL	chr8	12645091	T	A	48	42,00%	20	I	L	-2	LONRF1	rs1139354		1,00E-012
NA12760	reg_CTRL	chr8	12645405	C	T	52	98,00%	51			-	LONRF1	rs6530964		1,00E-012
NA12760	reg_CTRL	chr8	12645526	A	C	44	52,00%	23			-	LONRF1	rs6530965		1,00E-012
NA12760	reg_CTRL	chr8	12646136	C	G	39	97,00%	38			-	LONRF1	rs7842201		1,00E-012
NA12760	reg_CTRL	chr8	12646342	T	C	38	39,00%	15			-	LONRF1	rs7819248		1,00E-012
NA12760	reg_CTRL	chr8	12646448	A	G	47	34,00%	16			-	LONRF1	rs4831360		1,00E-012
NA12760	reg_CTRL	chr8	12646912	G	C	44	73,00%	32			-	LONRF1	rs4831784		1,00E-012
NA12760	reg_CTRL	chr8	12647447	T	C	77	55,00%	42			-	LONRF1	rs17829381		1,00E-012
NA12760	reg_CTRL	chr8	12647560	C	G	82	45,00%	37			-	LONRF1	rs6985289		1,00E-012
NA12760	reg_CTRL	chr8	12647843	T	C	85	48,00%	41			-	LONRF1	rs17829441		1,00E-012
NA12760	reg_CTRL	chr8	12647982	A	T	93	49,00%	46			-	LONRF1	rs6530966		1,00E-012
NA12760	reg_CTRL	chr8	12648329	G	C	102	98,00%	100			-	LONRF1	rs7838660		1,00E-012
NA12760	reg_CTRL	chr8	12648422	T	C	89	49,00%	44			-	LONRF1	rs7832448		1,00E-012
NA12760	reg_CTRL	chr8	12648780	C	G	72	54,00%	39			-	LONRF1	rs9325792		1,00E-012
NA12760	reg_CTRL	chr8	12648809	T	C	70	47,00%	33			-	LONRF1		rs73202647	1,00E-012
NA12760	reg_CTRL	chr8	12648863	A	G	61	52,00%	32			-	LONRF1	rs9325793		1,00E-012
NA12760	reg_CTRL	chr8	12649062	G	T	56	100,00%	56			-	LONRF1	rs7014516		1,00E-012
NA12760	reg_CTRL	chr8	12649595	G	A	19	100,00%	19			-	LONRF1	rs10441667		1,00E-012
NA12760	reg_CTRL	chr8	12650052	T	C	29	45,00%	13			-	LONRF1	rs17767600		1,00E-012
NA12760	reg_CTRL	chr8	12650145	C	T	31	45,00%	14			-	LONRF1	rs7462166		1,00E-012
NA12760	reg_CTRL	chr8	12650206	G	C	35	49,00%	17			-	LONRF1	rs6530967		1,00E-012
NA12760	reg_CTRL	chr8	12651062	T	C	45	51,00%	23			-	LONRF1	rs4436128		1,00E-012
NA12760	reg_CTRL	chr8	12651131	T	C	51	45,00%	23			-	LONRF1	rs4437649		1,00E-012
NA12760	reg_CTRL	chr8	12651557	C	G	46	39,00%	18			-	LONRF1	rs10503427		1,00E-012
NA12760	reg_CTRL	chr8	12651605	G	A	34	59,00%	20			-	LONRF1	rs6991754		1,00E-012
NA12760	reg_CTRL	chr8	12651680	C	T	46	100,00%	46			-	LONRF1	rs7007056		1,00E-012
NA12760	reg_CTRL	chr8	12652000	C	T	56	98,00%	55			-	LONRF1	rs7007550		1,00E-012
NA12760	reg_CTRL	chr8	12652284	C	G	63	98,00%	62			-	LONRF1	rs6530968		1,00E-012
NA12760	reg_CTRL	chr8	12652948	G	A	62	50,00%	31			-	LONRF1		rs73202653	1,00E-012
NA12760	reg_CTRL	chr8	12653488	C	G	27	96,00%	26			-	LONRF1	rs4831795		1,00E-012
NA12760	reg_CTRL	chr8	12654972	A	G	85	99,00%	84			-	LONRF1	rs6530969		1,00E-012
NA12760	reg_CTRL	chr8	12655147	T	C	88	52,00%	46			-	LONRF1	rs6530970		1,00E-012

MIN	3
MAX	206
AVG	59,2
MED	54,0
