# Supplementary material for: Comprehensive assessment of sequence variation within the copy number variable defensin cluster on 8p23 by target enriched in-depth 454 sequencing
Source: BMC Genomics. 2011 May 18;12:243. doi: 10.1186/1471-2164-12-243 (PMC3118217; doi:10.1186/1471-2164-12-243)
Supplement: Additional file 13 — Verification of discrepancies Sequence Capture/HapMap. PCR verification of 14 discrepancies Sequence Capture/HapMap and 6 HapMap SNPs not identified by Sequence Capture [file 1471-2164-12-243-S13.PDF]

additional file 13: Verification of discrepancies Sequence Capture/HapMap

						HapMap		Sequence Capture					PCR verification		rep	tile	Primer	
DNA	chr	pos	region	SNP_ID	hg18		type	var	type	dep	VAF	P		confirmed			forward	reverse
NA12716	chr8	6.746.980	DEFA	rs2741061	C	CG	het	G	homvar	13	92,00%	1,00E-012	CG	HapMap			GTCAGGGTAAAATTCAGCAG	TGTAGTCCCAGCTACTCAG
NA12716	chr8	12.652.000	LONRF1	rs7007550	C	CT	het	T	homvar	51	98,00%	1,00E-012	TT	SeqCap	IN		CAAACTCTACAAGGACAGG	CCTTACTTATTGTGCAGAGG
NA12716	chr8	6.745.889	DEFA	rs2702930	A	CC	homvar	C	het	125	37,00%	1,00E-012	AC	SeqCap			GATCCAGACTTGGCTTTG	GCAGACCTTGTTTACTGTCC
NA12716	chr8	6.746.389	DEFA	rs2702931	A	CC	homvar	C	het	40	42,00%	1,00E-012	AC	SeqCap	IN		TAAAGACTTGCCCGTGAC	TCACTGAGCCAGACTAAGAC
NA12716	chr8	6.812.906	DEFA	rs11996346	G	CC	homvar	C	het	90	52,00%	1,00E-012	GC	SeqCap			CTTTAAGCTGACACCGATTG	AGATTATACCGGATCTGCTG
NA12716	chr8	6.882.669	DEFA	rs6982904	G	TT	homvar	T	het	16	69,00%	1,00E-012	TT	HapMap	IN	OUT	CTGGTGTGTCTCACTGTTG	CCAAACTGGATCACACATC
NA12716	chr8	6.739.842	DEFA	rs2738149	C	CC	homhg18	T	het	79	57,00%	1,00E-012	CT	SeqCap			CCTGGACTCTCTGTTCTATG	GCTCCATGTCATAGGTTAGG
NA12716	chr8	6.808.715	DEFA	rs4840647	A	AA	homhg18	G	het	106	46,00%	1,00E-012	AG	SeqCap			ACTCAGTCTTGCATGTGTTC	GATCTGAATGTCTCTGAGGAG
NA12716	chr8	6.812.041	DEFA	rs13257112	G	GG	homhg18	T	het	56	55,00%	1,00E-012	GT	SeqCap			CAGTGGCAGTTATAGGAACC	AAATCGTAGCCTTCTTGTTG
NA12716	chr8	6.813.301	DEFA	rs13267882	G	GG	homhg18	A	het	92	42,00%	1,00E-012	GG	HapMap			AGCGACGTTGAAAGAAATG	GATTCCAAGATATGCCTGTG
NA12716	chr8	6.813.385	DEFA	rs13278672	T	TT	homhg18	G	het	74	38,00%	1,00E-012	TT	HapMap			AGCGACGTTGAAAGAAATG	GATTCCAAGATATGCCTGTG
NA12716	chr8	6.865.813	DEFA	rs11781199	T	TT	homhg18	C	het	92	20,00%	1,25E-011	TT	HapMap			CAAGTGGGGTATAGCTGTC	GAAGGAGAGCACAAGACTG
NA12716	chr8	6.865.875	DEFA	rs11781205	T	TT	homhg18	C	het	100	22,00%	1,00E-012	TC	SeqCap			CAAGTGGGGTATAGCTGTC	CTCTGTTCTCCCTCAGCTC
NA12716	chr8	6.886.899	DEFA	rs12716644	C	CC	homhg18	T	het	42	38,00%	1,00E-012	CT	SeqCap		OUT	CCTTGTTCTAAAGCCAGTTC	AGGGGAGGTCCTGTGTATC
NA12716	chr19	60.079.765	FCAR	rs10407012	G	AG	het		low qual/cov				AG	HapMap			GCAAACAGGGATAGTTTGAC	ATGGTGTCGATCTCCTGAC

NA12716	chr8	6.743.992	DEFA	rs13272703	G	CC	homvar		low qual/cov				CG				TATGCCTTCTT CTCTCCATC	CAAAATATCC TCGGGAGAG
NA12716	chr8	6.864.878	DEFA	rs11137085	G	CG	het		low qual/cov				CG	HapMap			CGTAGTTGGT GTCTCTAGTT TG	AGACATCCAC CATCTCAAC
NA12716	chr8	8.259.602	PRAGMIN	rs11996133	T	GT	het		low qual/cov				GT	HapMap			TTGATGAAGT CAAAGCCATC	TCCTTACAGT TCAGGGTCTG
NA12716	chr8	8.716.307	MFHAS1	rs3827809	G	CC	homvar		align probl				CC	HapMap			GCAGGAGGT CAAAGAATC	GAGGTTACTG CTTCATGC
NA12716	chr8	12.651.605	LONRF1	rs6991754	G	AG	het		low qual/cov				AA				GAAATGGGAC ACTTGAATC	GGCTTCTACT TAACCGAGAC
