# Supplementary material for: Comprehensive assessment of sequence variation within the copy number variable defensin cluster on 8p23 by target enriched in-depth 454 sequencing
Source: BMC Genomics. 2011 May 18;12:243. doi: 10.1186/1471-2164-12-243 (PMC3118217; doi:10.1186/1471-2164-12-243)
Supplement: Additional file 14 — Haplotyping candidate regions (HTCR). Haplotyping candidate regions (HTCR) with high density of polymorphic positions used for haplotyping [file 1471-2164-12-243-S14.PDF]

additional file 14: Haplotyping candidate regions (HTCR)

name	DNA	region	hg18	start	end	SNV	#SNV	within (bp)	SNV/kb	
HTCR_14	NA12716	DEFB	chr8	7.277.424	7.278.116	161-176	16	693	23	1
HTCR_15	NA12716	DEFB	chr8	7.280.224	7.280.364	177-180	4	141	28	2
HTCR_19_SPAG11	NA12716	DEFB	chr8	7.295.830	7.296.208	181-189	9	379	24	3
HTCR_20_DEFB104	NA12716	DEFB	chr8	7.315.477	7.315.783	190-195	6	307	20	4
HTCR_23	NA12716	DEFB	chr8	7.320.212	7.320.971	196-209	14	760	18	5
HTCR_24	NA12716	DEFB	chr8	7.322.954	7.323.118	210-214	5	165	30	6
HTCR_25	NA12716	DEFB	chr8	7.354.364	7.354.673	215-218	4	310	13	7
HTCR_26	NA12716	DEFB	chr8	7.362.617	7.362.640	219-222	4	24	167	8
HTCR_00_DEFB4	NA12760	DEFB	chr8	7.259.789	7.260.004	001-003	3	216	14	1
HTCR_01_downstr_B103	NA12760	DEFB	chr8	7.272.799	7.273.179	004-011	8	381	21	2
HTCR_03_SPAG11	NA12760	DEFB	chr8	7.295.159	7.296.408	012-028	17	1.250	14	3
HTCR_04_SPAG11	NA12760	DEFB	chr8	7.298.212	7.298.691	029-035	7	480	15	4
HTCR_05_SPAG11	NA12760	DEFB	chr8	7.301.157	7.302.236	036-045	10	1.080	9	5
HTCR_07_SPAG11	NA12760	DEFB	chr8	7.308.164	7.308.534	046-051	6	371	16	6
HTCR_08_DEFB104	NA12760	DEFB	chr8	7.315.477	7.315.804	052-057	6	328	18	7
HTCR_09_DEFB104	NA12760	DEFB	chr8	7.316.983	7.317.813	058-069	12	831	14	8
HTCR_10_DEFB104	NA12760	DEFB	chr8	7.319.055	7.320.256	070-083	14	1.202	12	9
HTCR_11_DEFB106	NA12760	DEFB	chr8	7.328.121	7.328.769	084-092	9	649	14	10
HTCR_12_DEFB106	NA12760	DEFB	chr8	7.330.373	7.330.574	093-096	4	202	20	11
HTCR_13_DEFB107	NA12760	DEFB	chr8	7.341.300	7.341.639	097-103	7	340	21	12
		total length (bp)		10.089		total	165	10.109	16	
