# Supplementary material for: Comprehensive assessment of sequence variation within the copy number variable defensin cluster on 8p23 by target enriched in-depth 454 sequencing
Source: BMC Genomics. 2011 May 18;12:243. doi: 10.1186/1471-2164-12-243 (PMC3118217; doi:10.1186/1471-2164-12-243)
Supplement: Additional file 15 — SNVs used for haplotyping within HTCRs. List of all SNVs within the HTCRs which were used for haplotyping and CN estimation illustrated in additional file 14 [file 1471-2164-12-243-S15.PDF]

additional file 15: SNVs used for haplotyping within HTCRs

DNA	SNV	HTCR region	chr	pos_hg18	ref	var	#reads	%var				gene	rs
NA12760	SNV001	HTCR_00_DEFB4	chr8	7.259.789	G	C	173	23,00%				DEFB4 (not annotated)	rs2737912
NA12760	SNV002	HTCR_00_DEFB4	chr8	7.259.849	G	A	175	23,00%				DEFB4 (not annotated)	rs2740091
NA12760	SNV003	HTCR_00_DEFB4	chr8	7.260.004	A	G	181	28,00%				DEFB4 (not annotated)	rs2740090
NA12760	SNV004	HTCR_01_downstr_B103	chr8	7.272.799	T	G	209	29,00%					rs2737549
NA12760	SNV005	HTCR_01_downstr_B103	chr8	7.272.812	G	T	212	49,00%					rs3988903
NA12760	SNV006	HTCR_01_downstr_B103	chr8	7.272.893	G	A	216	86,00%					rs3988902
NA12760	SNV007	HTCR_01_downstr_B103	chr8	7.272.905	G	C	223	100,00%					
NA12760	SNV008	HTCR_01_downstr_B103	chr8	7.272.963	A	G	239	28,00%					
NA12760	SNV009	HTCR_01_downstr_B103	chr8	7.273.000	T	A	247	19,00%					rs2737550
NA12760	SNV010	HTCR_01_downstr_B103	chr8	7.273.160	G	A	208	29,00%					rs3988900
NA12760	SNV011	HTCR_01_downstr_B103	chr8	7.273.179	A	G	198	17,00%					rs2740725
NA12760	SNV012	HTCR_03_SPAG11	chr8	7.295.159	G	C	170	100,00%			-	SPAG11B	
NA12760	SNV013	HTCR_03_SPAG11	chr8	7.295.311	T	A	184	44,00%			-	SPAG11B	rs2853660
NA12760	SNV014	HTCR_03_SPAG11	chr8	7.295.321	A	T	184	46,00%			-	SPAG11B	rs2737558
NA12760	SNV015	HTCR_03_SPAG11	chr8	7.295.375	G	A	179	21,00%			-	SPAG11B	rs4089926
NA12760	SNV016	HTCR_03_SPAG11	chr8	7.295.796	T	C	188	85,00%	H	R	-2	SPAG11B	rs1042797
NA12760	SNV017	HTCR_03_SPAG11	chr8	7.295.813	A	C	188	29,00%	I	M	-2	SPAG11B	rs12063
NA12760	SNV018	HTCR_03_SPAG11	chr8	7.295.820	G	A	189	44,00%	P	L	-2	SPAG11B	rs2256100
NA12760	SNV019	HTCR_03_SPAG11	chr8	7.295.894	C	A	182	42,00%			-	SPAG11B	rs2853659
NA12760	SNV020	HTCR_03_SPAG11	chr8	7.295.903	G	A	179	41,00%			-	SPAG11B	rs2738036
NA12760	SNV021	HTCR_03_SPAG11	chr8	7.295.939	C	T	173	37,00%			-	SPAG11B	
NA12760	SNV022	HTCR_03_SPAG11	chr8	7.296.080	T	C	172	40,00%	D	G	-2	SPAG11B	rs2738035
NA12760	SNV023	HTCR_03_SPAG11	chr8	7.296.085	A	G	175	41,00%	C	C	-2	SPAG11B	
NA12760	SNV024	HTCR_03_SPAG11	chr8	7.296.092	C	T	178	42,00%	R	K	-2	SPAG11B	
NA12760	SNV025	HTCR_03_SPAG11	chr8	7.296.116	C	T	182	42,00%	R	Q	-2	SPAG11B	rs2853658
NA12760	SNV026	HTCR_03_SPAG11	chr8	7.296.139	G	C	177	35,00%			-	SPAG11B	rs2294141
NA12760	SNV027	HTCR_03_SPAG11	chr8	7.296.208	A	G	165	95,00%			-	SPAG11B	rs4840280
NA12760	SNV028	HTCR_03_SPAG11	chr8	7.296.408	T	C	173	36,00%			-	SPAG11B	rs12682529
NA12760	SNV029	HTCR_04_SPAG11	chr8	7.298.212	T	G	141	48,00%			-	SPAG11B	rs2738031
NA12760	SNV030	HTCR_04_SPAG11	chr8	7.298.320	A	G	172	20,00%			-	SPAG11B	rs2740033
NA12760	SNV031	HTCR_04_SPAG11	chr8	7.298.410	C	A	201	18,00%			-	SPAG11B	rs2738029
NA12760	SNV032	HTCR_04_SPAG11	chr8	7.298.525	T	G	199	97,00%			-	SPAG11B	
NA12760	SNV033	HTCR_04_SPAG11	chr8	7.298.554	T	C	201	18,00%			-	SPAG11B	
NA12760	SNV034	HTCR_04_SPAG11	chr8	7.298.676	G	T	166	33,00%			-	SPAG11B	
NA12760	SNV035	HTCR_04_SPAG11	chr8	7.298.691	T	G	154	34,00%			-	SPAG11B	
NA12760	SNV036	HTCR_05_SPAG11	chr8	7.301.157	C	G	149	33,00%			-	SPAG11B	
NA12760	SNV037	HTCR_05_SPAG11	chr8	7.301.232	G	A	146	18,00%			-	SPAG11B	
NA12760	SNV038	HTCR_05_SPAG11	chr8	7.301.270	A	G	142	24,00%			-	SPAG11B	
NA12760	SNV039	HTCR_05_SPAG11	chr8	7.301.410	G	T	120	40,00%			-	SPAG11B	
NA12760	SNV040	HTCR_05_SPAG11	chr8	7.301.633	G	C	83	16,00%			-	SPAG11B	rs2738020
NA12760	SNV041	HTCR_05_SPAG11	chr8	7.301.857	A	G	72	86,00%			-	SPAG11B	rs2740031
NA12760	SNV042	HTCR_05_SPAG11	chr8	7.301.960	T	G	75	16,00%			-	SPAG11B	rs2737563
NA12760	SNV043	HTCR_05_SPAG11	chr8	7.301.986	G	A	78	97,00%			-	SPAG11B	rs2738017
NA12760	SNV044	HTCR_05_SPAG11	chr8	7.302.094	G	A	96	32,00%			-	SPAG11B	
NA12760	SNV045	HTCR_05_SPAG11	chr8	7.302.236	T	C	97	35,00%			-	SPAG11B	
NA12760	SNV046	HTCR_07_SPAG11	chr8	7.308.164	G	A	116	21,00%			-	SPAG11B	rs3817721
NA12760	SNV047	HTCR_07_SPAG11	chr8	7.308.325	C	A	116	16,00%			-	SPAG11B	
NA12760	SNV048	HTCR_07_SPAG11	chr8	7.308.331	A	G	118	34,00%			-	SPAG11B	rs2272769
NA12760	SNV049	HTCR_07_SPAG11	chr8	7.308.457	C	T	128	18,00%			-	SPAG11B	

NA12760	SNV050	HTCR_07_SPAG11	chr8	7.308.498	T	A	123	19,00%			-	SPAG11B	rs2272768
NA12760	SNV051	HTCR_07_SPAG11	chr8	7.308.534	G	A	130	18,00%			-	SPAG11B	
NA12760	SNV052	HTCR_08_DEFB104	chr8	7.315.477	A	C	177	43,00%			-	DEFB104A	
NA12760	SNV053	HTCR_08_DEFB104	chr8	7.315.533	G	A	168	96,00%			-	DEFB104A	
NA12760	SNV054	HTCR_08_DEFB104	chr8	7.315.562	G	T	166	21,00%			-	DEFB104A	rs2740696
NA12760	SNV055	HTCR_08_DEFB104	chr8	7.315.640	A	G	169	43,00%			-	DEFB104A	
NA12760	SNV056	HTCR_08_DEFB104	chr8	7.315.688	A	G	164	16,00%			-	DEFB104A	
NA12760	SNV057	HTCR_08_DEFB104	chr8	7.315.804	C	A	174	23,00%			-	DEFB104A	
NA12760	SNV058	HTCR_09_DEFB104	chr8	7.316.983	C	T	153	51,00%			-	DEFB104A	rs2740019
NA12760	SNV059	HTCR_09_DEFB104	chr8	7.317.035	C	T	158	99,00%			-	DEFB104A	
NA12760	SNV060	HTCR_09_DEFB104	chr8	7.317.106	G	T	156	28,00%			-	DEFB104A	rs2740018
NA12760	SNV061	HTCR_09_DEFB104	chr8	7.317.157	G	A	148	51,00%			-	DEFB104A	
NA12760	SNV062	HTCR_09_DEFB104	chr8	7.317.239	G	A	149	36,00%			-	DEFB104A	rs2737576
NA12760	SNV063	HTCR_09_DEFB104	chr8	7.317.261	C	A	142	96,00%			-	DEFB104A	
NA12760	SNV064	HTCR_09_DEFB104	chr8	7.317.363	C	A	142	20,00%			-	DEFB104A	rs2740011
NA12760	SNV065	HTCR_09_DEFB104	chr8	7.317.411	T	C	116	23,00%			-	DEFB104A	
NA12760	SNV066	HTCR_09_DEFB104	chr8	7.317.588	G	A	78	21,00%			-	DEFB104A	rs6985641
NA12760	SNV067	HTCR_09_DEFB104	chr8	7.317.699	A	G	43	21,00%			-	DEFB104A	rs28681639
NA12760	SNV068	HTCR_09_DEFB104	chr8	7.317.749	T	G	32	28,00%			-	DEFB104A	rs2740007
NA12760	SNV069	HTCR_09_DEFB104	chr8	7.317.813	C	G	23	96,00%			-	DEFB104A	rs7001088
NA12760	SNV070	HTCR_10_DEFB104	chr8	7.319.055	T	C	167	68,00%			-	DEFB104A	rs2739981
NA12760	SNV071	HTCR_10_DEFB104	chr8	7.319.063	T	C	164	69,00%			-	DEFB104A	rs2680506
NA12760	SNV072	HTCR_10_DEFB104	chr8	7.319.275	A	T	179	46,00%			-	DEFB104A	rs2739976
NA12760	SNV073	HTCR_10_DEFB104	chr8	7.319.333	T	C	190	16,00%			-	DEFB104A	
NA12760	SNV074	HTCR_10_DEFB104	chr8	7.319.456	T	C	189	50,00%			-	DEFB104A	rs2739969
NA12760	SNV075	HTCR_10_DEFB104	chr8	7.319.556	A	G	187	42,00%			-	DEFB104A	
NA12760	SNV076	HTCR_10_DEFB104	chr8	7.319.599	C	T	186	34,00%			-	DEFB104A	
NA12760	SNV077	HTCR_10_DEFB104	chr8	7.319.638	C	T	191	39,00%			-	DEFB104A	
NA12760	SNV078	HTCR_10_DEFB104	chr8	7.319.747	A	G	162	77,00%			-	DEFB104A	rs4259430
NA12760	SNV079	HTCR_10_DEFB104	chr8	7.319.771	T	C	157	77,00%			-	DEFB104A	
NA12760	SNV080	HTCR_10_DEFB104	chr8	7.319.973	T	C	151	17,00%	I	V	-1	DEFB104B	rs2680507
NA12760	SNV081	HTCR_10_DEFB104	chr8	7.320.136	G	C	132	73,00%					rs28590291
NA12760	SNV082	HTCR_10_DEFB104	chr8	7.320.212	C	G	112	77,00%					
NA12760	SNV083	HTCR_10_DEFB104	chr8	7.320.256	C	T	113	73,00%					
NA12760	SNV084	HTCR_11_DEFB106	chr8	7.328.121	A	G	170	38,00%			-	DEFB106B	rs2243995
NA12760	SNV085	HTCR_11_DEFB106	chr8	7.328.248	C	T	190	19,00%			-	DEFB106B	rs2738002
NA12760	SNV086	HTCR_11_DEFB106	chr8	7.328.290	T	C	185	59,00%			-	DEFB106B	rs2740081
NA12760	SNV087	HTCR_11_DEFB106	chr8	7.328.305	C	A	183	29,00%			-	DEFB106B	
NA12760	SNV088	HTCR_11_DEFB106	chr8	7.328.493	T	A	173	16,00%			-	DEFB106B	rs2740079
NA12760	SNV089	HTCR_11_DEFB106	chr8	7.328.517	A	G	171	19,00%			-	DEFB106B	rs2740078
NA12760	SNV090	HTCR_11_DEFB106	chr8	7.328.712	T	C	84	33,00%			-	DEFB106B	rs2680547, rs2837
NA12760	SNV091	HTCR_11_DEFB106	chr8	7.328.762	A	C	61	34,00%			-	DEFB106B	rs2680548
NA12760	SNV092	HTCR_11_DEFB106	chr8	7.328.769	T	G	59	20,00%			-	DEFB106B	
NA12760	SNV093	HTCR_12_DEFB106	chr8	7.330.373	G	C	112	33,00%			-	DEFB106B	rs2740039
NA12760	SNV094	HTCR_12_DEFB106	chr8	7.330.474	G	A	125	30,00%			-	DEFB106B	rs2740038
NA12760	SNV095	HTCR_12_DEFB106	chr8	7.330.551	T	C	143	43,00%			-	DEFB106B	rs6605634
NA12760	SNV096	HTCR_12_DEFB106	chr8	7.330.574	C	G	155	30,00%			-	DEFB106B	
NA12760	SNV097	HTCR_13_DEFB107	chr8	7.341.300	T	C	47	98,00%			+	DEFB107A	rs12682203
NA12760	SNV098	HTCR_13_DEFB107	chr8	7.341.374	C	G	50	34,00%			+	DEFB107A	
NA12760	SNV099	HTCR_13_DEFB107	chr8	7.341.377	A	G	48	33,00%			+	DEFB107A	
NA12760	SNV100	HTCR_13_DEFB107	chr8	7.341.389	G	T	48	17,00%			+	DEFB107A	rs12675434
NA12760	SNV101	HTCR_13_DEFB107	chr8	7.341.391	C	G	48	33,00%			+	DEFB107A	rs2737612
NA12760	SNV102	HTCR_13_DEFB107	chr8	7.341.618	G	T	122	16,00%			+	DEFB107A	

NA12760	SNV103	HTCR_13_DEFB107	chr8	7.341.639	C	T	132	23,00%			+	DEFB107A	
NA12716	SNV161	HTCR_14	chr8	7.277.424	C	A	62	94,00%					
NA12716	SNV162	HTCR_14	chr8	7.277.451	C	G	64	44,00%					
NA12716	SNV163	HTCR_14	chr8	7.277.460	G	A	63	43,00%					
NA12716	SNV164	HTCR_14	chr8	7.277.506	a	t	68	59,00%				not recognized by RunAssembly, adjacent SNP/SNV	
NA12716	SNV165	HTCR_14	chr8	7.277.507	T	C	68	59,00%					
NA12716	SNV166	HTCR_14	chr8	7.277.521	C	G	68	37,00%					
NA12716	SNV167	HTCR_14	chr8	7.277.545	C	T	68	34,00%					
NA12716	SNV168	HTCR_14	chr8	7.277.566	G	A	62	35,00%					
NA12716	SNV169	HTCR_14	chr8	7.277.662	A	G	60	32,00%					
NA12716	SNV170	HTCR_14	chr8	7.277.710	T	C	55	27,00%					
NA12716	SNV171	HTCR_14	chr8	7.277.744	A	C	57	30,00%					
NA12716	SNV172	HTCR_14	chr8	7.277.755	T	C	56	34,00%					
NA12716	SNV173	HTCR_14	chr8	7.277.810	A	G	59	34,00%					
NA12716	SNV174	HTCR_14	chr8	7.277.830	C	G	54	26,00%					
NA12716	SNV175	HTCR_14	chr8	7.278.010	C	T	84	98,00%					
NA12716	SNV176	HTCR_14	chr8	7.278.116	C	T	97	51,00%					
NA12716	SNV177	HTCR_15	chr8	7.280.224	G	C	64	44,00%					
NA12716	SNV178	HTCR_15	chr8	7.280.227	A	T	64	95,00%					
NA12716	SNV179	HTCR_15	chr8	7.280.259	T	G	60	47,00%					rs2698836
NA12716	SNV180	HTCR_15	chr8	7.280.364	G	C	63	98,00%					
NA12716	SNV181	HTCR_19	chr8	7.295.830	C	T	69	43,00%	D	N	-2	SPAG11B	
NA12716	SNV182	HTCR_19	chr8	7.295.894	C	A	72	40,00%			-	SPAG11B	rs2853659
NA12716	SNV183	HTCR_19	chr8	7.295.903	G	A	72	40,00%			-	SPAG11B	rs2738036
NA12716	SNV184	HTCR_19	chr8	7.295.939	C	T	67	30,00%			-	SPAG11B	
NA12716	SNV185	HTCR_19	chr8	7.296.080	T	C	62	35,00%	D	G	-2	SPAG11B	rs2738035
NA12716	SNV186	HTCR_19	chr8	7.296.085	A	G	61	38,00%	C	C	-2	SPAG11B	
NA12716	SNV187	HTCR_19	chr8	7.296.092	C	T	61	38,00%	R	K	-2	SPAG11B	
NA12716	SNV188	HTCR_19	chr8	7.296.116	C	T	61	36,00%	R	Q	-2	SPAG11B	rs2853658
NA12716	SNV189	HTCR_19	chr8	7.296.208	A	G	59	100,00%			-	SPAG11B	rs4840280
NA12716	SNV190	HTCR_20	chr8	7.315.477	A	C	97	41,00%			-	DEFB104A	
NA12716	SNV191	HTCR_20	chr8	7.315.533	G	A	83	96,00%			-	DEFB104A	
NA12716	SNV192	HTCR_20	chr8	7.315.562	G	T	81	37,00%			-	DEFB104A	rs2740696
NA12716	SNV193	HTCR_20	chr8	7.315.640	A	G	70	49,00%			-	DEFB104A	
NA12716	SNV194	HTCR_20	chr8	7.315.688	A	G	62	47,00%			-	DEFB104A	
NA12716	SNV195	HTCR_20	chr8	7.315.783	T	C	49	43,00%			-	DEFB104A	
NA12716	SNV196	HTCR_23	chr8	7.320.212	C	G	27	96,00%					
NA12716	SNV197	HTCR_23	chr8	7.320.256	C	T	29	97,00%					
NA12716	SNV198	HTCR_23	chr8	7.320.337	G	A	36	44,00%					
NA12716	SNV199	HTCR_23	chr8	7.320.389	C	T	39	97,00%					
NA12716	SNV200	HTCR_23	chr8	7.320.465	C	T	43	100,00%					
NA12716	SNV201	HTCR_23	chr8	7.320.516	A	G	42	100,00%					
NA12716	SNV202	HTCR_23	chr8	7.320.524	C	G	41	100,00%					
NA12716	SNV203	HTCR_23	chr8	7.320.543	A	G	42	98,00%					
NA12716	SNV204	HTCR_23	chr8	7.320.547	A	G	41	100,00%					
NA12716	SNV205	HTCR_23	chr8	7.320.699	G	A	38	97,00%					
NA12716	SNV206	HTCR_23	chr8	7.320.772	A	C	28	96,00%					rs2680508
NA12716	SNV207	HTCR_23	chr8	7.320.916	A	G	35	100,00%					
NA12716	SNV208	HTCR_23	chr8	7.320.949	G	A	41	85,00%					rs2740690
NA12716	SNV209	HTCR_23	chr8	7.320.971	C	G	44	39,00%					rs2680509
NA12716	SNV210	HTCR_24	chr8	7.322.954	C	G	38	97,00%					rs28695206
NA12716	SNV211	HTCR_24	chr8	7.323.016	A	G	48	50,00%					
NA12716	SNV212	HTCR_24	chr8	7.323.020	A	T	49	92,00%					

NA12716	SNV213	HTCR_24	chr8	7.323.062	T	A	68	51,00%					
NA12716	SNV214	HTCR_24	chr8	7.323.118	A	G	82	49,00%					rs2737579
NA12716	SNV215	HTCR_25	chr8	7.354.364	A	G	106	44,00%					rs4143089
NA12716	SNV216	HTCR_25	chr8	7.354.376	T	C	108	44,00%					rs4143090
NA12716	SNV217	HTCR_25	chr8	7.354.477	G	A	114	45,00%					rs4143091
NA12716	SNV218	HTCR_25	chr8	7.354.673	C	G	106	98,00%					
NA12716	SNV219	HTCR_26	chr8	7.362.617	A	G	28	89,00%					
NA12716	SNV220	HTCR_26	chr8	7.362.619	G	A	29	41,00%					
NA12716	SNV221	HTCR_26	chr8	7.362.633	A	G	31	45,00%					
NA12716	SNV222	HTCR_26	chr8	7.362.640	A	G	31	45,00%					

7333
