# Supplementary material for: Comprehensive assessment of sequence variation within the copy number variable defensin cluster on 8p23 by target enriched in-depth 454 sequencing
Source: BMC Genomics. 2011 May 18;12:243. doi: 10.1186/1471-2164-12-243 (PMC3118217; doi:10.1186/1471-2164-12-243)
Supplement: Additional file 16 — DEFB haplotype inference and CN estimation. CN estimation by calculation of the ratios of reads representing the different haplotypes within the HTCRs [file 1471-2164-12-243-S16.PDF]

additional file 16: DEFB haplotype inference and CN estimation

SUMMARY

		# of SNVs per combination						
		2	***3***	***4***	***5***	***6***	***7***	
no. of HTs	NA12716	6	6	6	4			22
	NA12760	30	43	42	26	22	3	166
		36	49	48	30	22	3	188

	SNVs	informative	too few reads
NA12716	62	33	29
NA12760	103	91	12
	165	124	41

a) NA12716 / DEFB (threshold: ≥10 reads)

ratio_reads	cp	#reads																					
				HTCR14																			
			SNV	161	162	163	164	165	166	167	168	169	170	171	172	173	174	175	176				
		21																C	C				
1:1		27	48	SNV 161-174 too few reads														C	T				
				HTCR15				HTCR19															
			SNV	177	178	179	180	181	182	183	184	185	186	187	188	189							
		22		C	T	G	C																
3:2		14	36	G	T	T	C																
		17						C	C	G	C												
3:2		12	29					T	A	A	T												
		9										C	G	T	T	G							
2:3		13	22									T	A	C	C	G							
				HTCR20						HTCR23													
			SNV	190	191	192	193	194	195	196	197	198	199	200	201	202	203	204	205	206	207	208	209
		14		A	A	T																	
1:1		12	26	C	A	G	SNV 193-206 too few reads																
		11																			G	A	C
1:1		10	21																		G	A	G
				HTCR24					HTCR25				HTCR26										
			SNV	210	211	212	213	214	215	216	217	218	219	220	221	222							
		7		G	A	T	T	G				SNV 218 too few reads											
1:2 (3n)		13	20	G	G	T	A	A															
		14					T	G															
1:1		16	30				A	A															
		12							A	T													
1:1		12	24						G	C													
		33							A	T	G												
1:1		28	61						G	C	A												
		12											G	A	G	G							
3:4		16	28										G	G	A	A							
allele 1		172																					
allele 2		173																					
# of reads		345	345																				
avreads		16	31																				

		# of SNVs					
		2	3	4	5	6	
NA12716 DEFB	Number of haplotypes	6	6	6	4	0	22

b) NA12760 / DEFB (threshold: ≥30 reads)

reads ratio	CN	#reads		HTCR00 DEFB4			HTCR 01 downstream of DEFB103															
			SNV	001	002	003	004	005	006	007	008	009	010	011						
		17		C	A															
1:2 (3n)	6	34	51	G	G															
		16		C	A	G														
		62		G	G	A														
1:4:1	6	11	89	G	G	G														
		10					G	T												
		26					T	G												
1:3:1	5	9	45				T	T												
		11					G	T	A	C										
		14					T	G	A	C										
		4					T	G	G	C										
2:3:1:1	7	6	35				T	T	A	C										
		25					G	T	A	C	A	T								
		12					G	T	A	C	G	T								
		19					T	G	A	C	A	T								
		13					T	G	A	C	G	T								
		12					T	G	G	C	A	T								
2:1:1:1:1:2	8	21	102				T	T	A	C	A	A								
		11							A	C	A	A	A	G						
		5							A	C	A	T	A	A						
		15							A	C	A	T	G	A						
		19							A	C	G	T	G	A						
unclear		7	57						G	C	A	T	G	A						
		8									A	A	A	G						
		6									A	T	A	A						
		11									A	T	G	A						
1:1:2:1	5	8	33								G	T	G	A						
		9											A	A						
		8											A	G						
1:1:4	6	47	64										G	A						
				HTCR03 SPAG11																
				012	013	014	015	016	017	018	019	020	021	022	023	024	025	026	027	028
		15		C	A	T														

2:3	5	23	38	C	T	A														
		29		C	A	T	G													
		16		C	T	A	A													
2:1:1	4	15	60	C	T	A	G													
		50			A	T	G													
		16			T	A	A													
3:1:2	6	34	100		T	A	G													
		7						C	A	A										
		12						C	A	G										
		15						C	C	A										
1:2:2:1	6	8	42					T	A	G										
		22						C	A	G	C	G	C							
		11						T	A	G	C	G	C							
		12						C	A	A	A	A	T							
2:1:1:2	6	18	63					C	C	A	A	A	T							
		20												C	G	T	T	G	G	
		27												T	A	C	C	C	G	
3:4:1	8	7	54											T	A	C	C	G	G	
		20												C	G	T	T	G	G	T
		10												T	A	C	C	C	G	C
3:2:1	6	7	37											T	A	C	C	G	G	T
		19																	G	C
1:1 (2n)	6	16	35																G	T
				HTCR04_SPAG11							HTCR05_SPAG11									
			SNV	029	030	031	032	033	034	035	036	037	038	039	040	041	042	043	044	045
		14		G	A	C														
		21		T	A	C														
2:3:2	7	13	48	T	G	A														
		4			A	C	G	C												
		21			A	C	G	T												
1:5:1	7	5	30		G	A	G	T												
		8				C	G	C	G	T										
		14				C	G	T	G	T										
2:3:2	7	10	32			C	G	T	T	G										
		7					G	C	G	T										
		22					G	T	G	T										
1:3:3	7	19	48				G	T	T	G										
		9									C	A	A							
		20									C	G	A							
		6									G	G	A							
1:2:1:1	5	13	48								G	G	G							
		13									C	A	A	T						
		18									C	G	A	G						

2:2:2:1:1	8	13<br>7<br>11	62	C<br>G<br>G	G<br>G<br>G	A<br>A<br>G	T<br>G<br>G				
1:2:3	6	6<br>13<br>15	34				G<br>G<br>T	C<br>G<br>G			
1:2 (3n)	6	12<br>21	33						SNV 41-43too few reads<br>SNV 41-43too few reads	A<br>G	C<br>T

				HTCR07_SPAG11					
				046	047	048	049	050	051
1:1:4	6	6<br>8<br>26	40	A<br>G<br>G	C<br>A<br>C	G<br>G<br>A			
1:1:4:1	7	3<br>4<br>21<br>5	33		A<br>C<br>C<br>C	G<br>A<br>A<br>G	C<br>C<br>C<br>T	T<br>A<br>T<br>T	
1:3:1	5	12<br>33<br>8	53				C<br>C<br>T	A<br>T<br>T	A<br>G<br>G

				HTCR08_DEFB104						HTCR09_DEFB104											
			SNV	052	053	054	055	056	057	058	059	060	061	062	063	064	065	066	067	068	069
3:1:2	6	17<br>7<br>12	36	A<br>A<br>C	A<br>A<br>A	G<br>T<br>G															
2:2:1	5	9<br>11<br>3<br>9<br>5	37	A<br>A<br>C<br>C<br>C	A<br>A<br>A<br>A<br>A	G<br>T<br>G<br>G<br>G	A<br>A<br>A<br>G<br>G	A<br>A<br>A<br>A<br>G													
1:2:2:1	6	10<br>17<br>17<br>9	53				A<br>A<br>G<br>G	A<br>A<br>A<br>G	A<br>C<br>C<br>C												
2:1 (3n)	6	20<br>12	32							C<br>T	T<br>T										
3:2:1	6	13<br>10<br>7	30							C<br>T<br>T	T<br>T<br>T	G<br>G<br>T	G<br>A<br>A								
3:1:1:1	6	14<br>6<br>6<br>4	30							C<br>T<br>T<br>T	T<br>T<br>T<br>T	G<br>G<br>T<br>T	G<br>A<br>A<br>A	G<br>A<br>A<br>G	A<br>A<br>A<br>A	SNVs 64-69 too few reads<br>SNVs 64-69 too few reads<br>SNVs 64-69 too few reads<br>SNVs 64-69 too few reads					

				HTCR10_DEFB104													
			SNV	070	071	072	073	074	075	076	077	078	079	080	081	082	083
3:1	4	63<br>23	86	C<br>T	C<br>T												
		6		C	C	A	T										

		8		C	C	T	C										
		21		C	C	T	T										
1:1:3:3	8	27	62	T	T	A	T										
		13				A	T	C									
		23				A	T	T									
		15				T	C	C									
1:2:1:1	5	14	65			T	T	C									
		6						C	G	C	T	G	C				
		6						C	G	T	C	G	C				
		6						C	G	T	T	G	C				
		5						T	A	C	C	A	T				
1:1:1:1:2	6	8	31					T	A	C	C	G	C				
		10							A	C	C	A	T				
		10							A	C	C	G	C				
		5							G	C	T	G	C				
		3							G	T	C	G	C				
2:2:1:1:2	8	10	38						G	T	T	G	C				
		6										A	T	T			
		7										G	C	C			
1:1:3	5	16	29									G	C	T			
		9												C	C		
		14												T	C		
2:3:2	7	8	31											T	G		
		6														C	C
1:5	6	27	33													G	T
				HTCR11_DEFB106													
				084	085	086	087	088	089	090	091	092					
		32		A	C	C	A										
		12		A	C	C	C										
		18		A	T	C	C										
2:1:1:2	6	38	100	G	C	T	C										
		21			C	C	A	T	A	SNVs 90-92 too few reads							
		13			C	C	C	T	A	SNVs 90-92 too few reads							
		31			C	T	C	T	A	SNVs 90-92 too few reads							
2:1:3:1	7	12	77		T	C	C	A	G	SNVs 90-92 too few reads							
		8						A	G	SNVs 90-92 too few reads							
1:3 (4n)	8	22	30					T	A	SNVs 90-92 too few reads							
				HTCR12_DEFB106				HTCR13_DEFB107									
			SNV	093	094	095	096	097	098	099	100	101	102	103			
		20		C	A	T	C										
		8		G	G	C	C										
		22		G	G	C	G										
2:1:2:1	6	13	63	G	G	T	C										
		17			A	T	C										
		6			G	C	C										
		24			G	C	G										
2:1:3:2	8	14	61		G	T	C										
		7				C	C										

		5				C	G							
1:1:4	6	27	39			T	C							
		5						C	C	A	G	C		
		5						C	C	A	G	G		
		5						C	C	A	T	C		
1:1:1:3	6	13	28					C	G	G	G	C		
		78											G	C
4:1	5	19	97										T	T
avg_cp	6,19													
max_cp	8													
min_cp	4													
STDEV	1,04													
# of CN est	48													
# of reads		2454	2454											
avreads		15	50											
				# of SNVs										
				2	3	4	5	6	7					
NA12760 DEFB			Number of haplotypes	30	43	42	26	22	3	166				
