# Supplementary material for: Comprehensive assessment of sequence variation within the copy number variable defensin cluster on 8p23 by target enriched in-depth 454 sequencing
Source: BMC Genomics. 2011 May 18;12:243. doi: 10.1186/1471-2164-12-243 (PMC3118217; doi:10.1186/1471-2164-12-243)
Supplement: Additional file 17 — SNVs in the DEFB4 promoter region. SNVs in the DEFB4 promoter region in comparison to previous data published in Ref. [25] [file 1471-2164-12-243-S17.PDF]

additional file 17: SNVs in the DEFB4 promoter region

SNV	Ref. [25]	chr	prox prox_hg18	dist hg18	prox type	dist type	NA12760 (dist) ref	var	type	avVAF	NA12716 (dist) ref	var	type	avVAF	
SNV227	49	chr8	7.789.537	7.261.867	Y	R	A	G	R	0,23	A	G	R	0,43	rs2740086
SNV228	48	chr8	7.789.459	7.261.945	R	Y	homoT				homoT				
SNV229	47	chr8	7.789.442	7.261.962	M	K	T	G	K	0,17	homoT				putnovel
SNV230	46	chr8	7.789.376	7.262.028	S	S	C	G	S	0,32	homoC				rs2698827
SNV231	45	chr8	7.789.248	7.262.156	Y	R	homoG				homoG				
SNV232	44	chr8	7.789.170	7.262.234	M	K	G	T	K	0,21	homoG				rs2698828
SNV233	43	chr8	7.789.134	7.262.270	R	Y	C	T	Y	0,17	homoC				putnovel
SNV234	42	chr8	7.789.117	7.262.287	c	g	homoG				homoG				
SNV235	41	chr8	7.788.876	7.262.529	d(A,G,T)	h(A/C/T)	homoC				homoC				
SNV236	40	chr8	7.788.791	7.262.614	R	Y	homoC				homoC				
SNV237	39	chr8	7.788.777	7.262.628	R	Y	homoC				homoC				
SNV238	38	chr8	7.788.771	7.262.634	R	Y	C	T	Y	0,25	homoC				rs2737535
SNV239	37	chr8	7.788.750	7.262.655	V(A/C/G)	B(C/G/T)	homoG				homoG				
SNV240	36	chr8	7.788.738	7.262.667	V(A/C/G)	B(C/G/T)	homoC				homoC				
SNV241	35	chr8	7.788.733	7.262.672	B(C/G/T)	V(A/C/G)	G	A	R	0,15	G	A	R	0,48	putnovel
SNV242	34	chr8	7.788.723	7.262.682	Y	R	homoG				homoG				
SNV243	33	chr8	7.788.722	7.262.683	Y	R	G	A	R	0,21	homoG				rs3762041
SNV244	32	chr8	7.788.718	7.262.687	R	Y	C	T	Y	0,19	homoC				putnovel
SNV245	31	chr8	7.788.618	7.262.787	R	Y	C	T	Y	0,16	C	T	Y	0,47	rs3762040
SNV246	30	chr8	7.788.610	7.262.795	K	M	C	A	M	0,19	homoC				rs3762039
SNV247	29	chr8	7.788.593	7.262.812	R	Y	C	T	Y	0,14	homoC				putnovel
SNV248	28	chr8	7.788.563	7.262.842	R	Y	homoC				homoC				
SNV249	27	chr8	7.788.403	7.263.002	Y	R	homoG				homoG				
SNV250	26	chr8	7.788.390	7.263.015	R	Y	homoC				homoC				
SNV251	25	chr8	7.788.303	7.263.102	R	Y	T	C	Y	0,77	T	C	Y	0,98	rs4840278
SNV252	not identified	chr8	7.788.224	7.263.181	g	c	C	A	M	0.10*)	homoC				putnovel
SNV253	24	chr8	7.788.222	7.263.183	R	Y	homoT				homoT				
SNV254	23	chr8	7.788.214	7.263.191	S	S	G	C	S	0,20	G	C	S	0,52	rs3762052
SNV255	22	chr8	7.788.108	7.263.297	R	Y	homoC				homoC				
SNV256	21	chr8	7.788.060	7.263.345	Y	R	G	A	R	0,55	homoG				rs4840751
SNV257	20	chr8	7.788.056	7.263.349	Y	R	G	A	R	0,16	G	A	R	0,58	rs3762051
SNV258	19	chr8	7.787.997	7.263.407	Y	R	A	G	R	0,17	A	G	R	0,50	putnovel
SNV259	18	chr8	7.787.992	7.263.412	Y	R	G	A	R	0,20	homoG				rs2698830
SNV260	not identified	chr8	7.787.985	7.263.419	t	a	A	G	R	0,24	homoA				rs2737910
SNV261	17	chr8	7.787.968	7.263.436	Y	R	G	A	R	0,45	homoG				rs4840752
SNV262	16	chr8	7.787.957	7.263.448	Y	R	homoA				homoA				
SNV263	15	chr8	7.787.922	7.263.483	W	W	homoT				homoT				
SNV264	14	chr8	7.787.889	7.263.515	R	Y	homoT				homoT				
SNV265	13	chr8	7.787.873	7.263.531	R	Y	T	C	Y	0,20	T	C	Y	0,53	putnovel
SNV266	12	chr8	7.787.799	7.263.605	Y	R	homoA				A	G	R	0,41	putnovel
SNV267	11	chr8	7.787.757	7.263.647	Y	R	A	G	R	0,20	A	G	R	0,37	putnovel
SNV268	10	chr8	7.787.747	7.263.657	W	W	homonucleotide stretch				homonucleotide stretch				
SNV269	9	chr8	7.787.746	7.263.658	a	t	homonucleotide stretch				homonucleotide stretch				
SNV270	8	chr8	7.787.745	7.263.659	a	t	homonucleotide stretch				homonucleotide stretch				
SNV271	7	chr8	7.787.744	7.263.660	a	t	homonucleotide stretch				homonucleotide stretch				
SNV272	6	chr8	7.787.743	7.263.661	y	R	homonucleotide stretch				homonucleotide stretch				
SNV273	5	chr8	7.787.740	7.263.664	W	W	homonucleotide stretch				homonucleotide stretch				
SNV274	4	chr8	7.787.739	7.263.665	R	Y	homonucleotide stretch				homonucleotide stretch				
SNV275	3	chr8	7.787.724	7.263.683	R	Y	T	C	Y	0,16	T	C	Y	0,37	putnovel

SNV276	2	chr8	7.787.663	7.263.744	Y	R	G	A	R	0,28	G	A	R	0,37	rs2740083
SNV277	1	chr8	7.787.643	7.263.764	S	S	G	C	S	0,13	G	C	S	0,34	rs2409862

*) experimentally (PCR, direct sequencing): NOT confirmed, homozygot C
