# Supplementary material for: Comprehensive assessment of sequence variation within the copy number variable defensin cluster on 8p23 by target enriched in-depth 454 sequencing
Source: BMC Genomics. 2011 May 18;12:243. doi: 10.1186/1471-2164-12-243 (PMC3118217; doi:10.1186/1471-2164-12-243)
Supplement: Additional file 18 — DEFB4 Promoter region haplotype inference and CN estimation (NA12760). Haplotype calls (HC) and CN estimation based on distal cluster for NA12760/DEFB4 Promoter region (threshold: ≥30 reads) [file 1471-2164-12-243-S18.PDF]

additional file 18: DEFB4 Promoter region haplotype inference and CN estimation (NA12760)

reads	SNV	227	228	229	230	231	232	233	234	235	236	237	238	239	240	241	242	243	244	245	246	247	248	249	250	251	252	253	254
		1	hom	2	3	hom	4	5	hom	hom	hom	hom	6	hom	hom	7	hom	8	9	10	11	12	hom	hom	hom	13	14	hom	15
29		A	T	T	C	G																							
26		A	T	T	G	G																							
8		G	T	G	C	G																							
6	69	G	T	T	C	G																							
57							G	C	G	C	C	C																	
10							G	T	G	C	C	C																	
16	83						T	C	G	C	C	C																	
9													T	G	C	G	G	G	T	C	C	C							
12													C	G	C	G	G	A	C	C	A	C							
18													C	G	C	G	G	G	C	C	C	C							
7													C	G	C	A	G	G	C	T	C	C							
6	52												C	G	C	G	G	G	C	C	C	T							
8																							C	G	C	C	C	T	C
32																							C	G	C	C	C	T	G
2																							C	G	C	C	A	T	C
1																							C	G	C	T	A	T	C
14	57																						C	G	C	T	C	T	G

reads	SNV	267		275	276	277
		23	nd	24	25	26
13		A	homopymer stretch	T	A	G
55		A		T	G	G
10	78	G		C	A	C
